# Splicing factor SF3B1 promotes endometrial cancer progression via regulating KSR2 RNA maturation

**DOI:** 10.1038/s41419-020-03055-y

**Published:** 2020-10-10

**Authors:** Pooja Popli, Megan M. Richters, Sangappa B. Chadchan, Tae Hoon Kim, Eric Tycksen, Obi Griffith, Premal H. Thaker, Malachi Griffith, Ramakrishna Kommagani

**Affiliations:** 1 Department of Obstetrics and Gynecology, Center for Reproductive Health Sciences, Washington University School of Medicine, St. Louis, MO 63110 USA; 2 Division of Oncology, Department of Medicine, Washington University School of Medicine, St. Louis, MO 63110 USA; 3 Genome Technology Access Center, McDonnell Genome Institute, Washington University School of Medicine, St. Louis, MO 63110 USA; 4 Department of Obstetrics, Gynecology and Reproductive Biology, Michigan State University, Grand Rapids, MI 48824 USA; 5 Department of Genetics, Washington University School of Medicine, St. Louis, MO 63110 USA; 6 Siteman Cancer Center, Washington University School of Medicine, St. Louis, MO 63110 USA; 7 Division of Gynecologic Oncology, Department of Obstetrics and Gynecology, Washington University School of Medicine, St. Louis, MO 63110 USA

**Keywords:** Endometrial cancer, Endometrial cancer

## Abstract

Although endometrial cancer is the most common cancer of the female reproductive tract, we have little understanding of what controls endometrial cancer beyond the transcriptional effects of steroid hormones such as estrogen. As a result, we have limited therapeutic options for the ~62,000 women diagnosed with endometrial cancer each year in the United States. Here, in an attempt to identify new prognostic and therapeutic targets, we focused on a new area for this cancer—alternative mRNA splicing—and investigated whether splicing factor, SF3B1, plays an important role in endometrial cancer pathogenesis. Using a tissue microarray, we found that human endometrial tumors expressed more SF3B1 protein than non-cancerous tissues. Furthermore, *SF3B1* knockdown reduced in vitro proliferation, migration, and invasion of the endometrial cancer cell lines Ishikawa and AN3CA. Similarly, the SF3B1 inhibitor, Pladienolide-B (PLAD-B), reduced the Ishikawa and AN3CA cell proliferation and invasion in vitro. Moreover, PLAD-B reduced tumor growth in an orthotopic endometrial cancer mouse model. Using RNA-Seq approach, we identified ~2000 differentially expressed genes (DEGs) with *SF3B1* knockdown in endometrial cancer cells. Additionally, alternative splicing (AS) events analysis revealed that *SF3B1* depletion led to alteration in multiple categories of AS events including alternative exon skipping (ES), transcript start site usage (TSS), and transcript termination site (TTS) usage. Subsequently, bioinformatics analysis showed *KSR2* as a potential candidate for *SF3B1-*mediated functions in endometrial cancer. Specifically, loss of *SF3B1* led to decrease in KSR2 expression, owing to reduced maturation of *KSR2* pre-mRNA to a mature RNA. Importantly, we found rescuing the *KSR2* expression with *SF3B1* knockdown partially restored the cell growth of endometrial cancer cells. Taken together, our data suggest that *SF3B1* plays a crucial oncogenic role in the tumorigenesis of endometrial cancer and hence may support the development of SF3B1 inhibitors to treat this disease.

## Introduction

In 2020, endometrial cancer is expected to be newly diagnosed in approximately 65,620 patients, and 12,590 women are predicted to die from this disease in the United States, making it the most common gynecologic malignancy^1^. With the rise of obesity—a strong risk factor for endometrial cancer—the number of cases are expected to double by 2030^2^. Although most endometrial cancer patients present with low-grade, estrogen-driven, type 1, early-stage disease and have a favorable prognosis, those with advanced-stage, hormone-independent, type 2, metastatic disease have few treatment options and poor survival outcomes. Currently, major treatment options include endocrine therapy, chemotherapy, immunotherapy, radiotherapy, and hysterectomy. However, endocrine therapy is not widely used because of its limited efficacy, and hysterectomy is not a suitable option for women who wish to preserve fertility. Developing new therapeutic options will require a greater understanding of genes that contribute to endometrial cancer progression.

Recent work in several cancers has revealed that dysregulation of splicing factors can lead to the generation of abnormal mature transcripts^3^ that drive tumorigenesis^4–7^. For example, mutations in *U2AF1*, *SRSF2, ZRSR2*, and *SF3B1* have been identified in myelodysplastic syndromes, chronic lymphocytic leukemia, uveal melanoma, and breast cancer^8–19^. In endometrial cancer, the SF3B1 is one of the frequently mutated splicing factor^20,21^. SF3B1 is a core component of the U2 small nuclear ribonucleoprotein, which specifically recognizes the 3′ splice site at intron–exon junctions^22,23^. In endometrial cancer and several other cancers, *SF3B1* appears to act as an oncogene, as most of the mutations occur in hotspots^20^. Besides, overexpression of SF3B1 has also been reported to drive tumorigenesis in several cancers, including breast cancer, prostate cancer, and myelodysplastic syndromes^24–26^. Moreover, a recent study revealed that knock down of overexpressed *SF3B1* expression reduced breast cancer cell proliferation, migration, and invasion^26^. However, whether SF3B1 overexpression likewise promotes endometrial cancer progression is unknown.

Here, we report that SF3B1 protein is overexpressed in human endometrial tumor samples and endometrial cancer cell lines. Further, we show that knockdown of *SF3B1* or treatment with the SF3B1 inhibitor Pladienolide-B reduces cell viability, migration, and invasion of endometrial cancer cells in vitro and endometrial tumor cell growth in vivo. Finally, we report that knockdown of *SF3B1* alters mRNA maturation of the kinase suppressor of Ras gene *KSR2* and KSR2 acts as a downstream mediator of SF3B1 function(s) in endometrial cancer. Thus, SF3B1 protein expression may be a prognostic biomarker and a therapeutic target for treating endometrial cancer patients.

## Materials and methods

### Mouse and human study approval

Animal studies were performed according to a protocol (#20160227) approved by the Institutional Animal Care and Use Committee of Washington University School of Medicine, Saint Louis, MO, USA. Human volunteers provided written informed consent in accordance with an Institutional Review Board-approved protocol (#201612127) from Washington University School of Medicine and the guidelines of the Declaration of Helsinki.

### Cell culture

The human endometrial cancer cell lines Ishikawa (Sigma, St. Louis, MO) and AN3CA, KLE, and RL-95-2 (all from American Type Culture Collection) were purchased and used within 6–8 months. All cells were maintained in MEM/DMEM medium supplemented with 10% fetal bovine serum at 37 °C and 5% CO_2_.

### Orthotopic endometrial cancer model

Female athymic nude mice (6–8 weeks of age; Jackson Laboratory) were maintained under pathogen-free conditions with food and water provided ad libitum.

The sample size of animal experiment was chosen based on the preliminary experiments and similar well-designed experiments, and no statistical method was used. Investigators were blinded to the treatment groups during data collection and subsequent data analysis. Before surgery, animals received intramuscular 0.1 mg/kg buprenorphine hydrochloride (Temgesic, Reckitt Benckiser, Berkshire, UK) for analgesia. Mice were anesthetized with 100 mg/kg ketamine and 5 mg/kg xylazine and placed on a heating pad in dorsal decubitus. Their dorsal skin was cleaned and sterilized with betadine, and a 0.5-cm incision was made in the right lower flank to expose the right uterine horn. A single-cell suspension of 50 μl of ice-cold Matrigel (BD Matrigel Basement Membrane Matrix, BD Biosciences, San Jose, CA) containing 2 × 10^6^ AN3CA cells was injected into the endometrial cavity through the myometrium by using a 0.3 mm insulin syringe (Omnican 50; B-Braun, Melsungen, Germany). The needle was retracted after waiting for a few seconds to allow the Matrigel cell suspension to polymerize and to prevent leakage into the abdominal cavity. The uterine horn was returned to its original position, and the incision was closed with staples. After the surgery, animals were placed in a warm environment and supervised until full recovery. Three weeks later, mice were randomly assigned to receive either 10 mg/kg PLAD-B (*n* = 4) or vehicle (DMSO) (*n* = 4) intraperitoneally on days 0, 2, 4, and 6. Animals were weighed weekly. Two weeks after the last dose, the mice were sacrificed, and the tumors were imaged, weighed, and processed for histological analysis and immunofluorescence.

### Co-transfection experiment

Lipofectamine 2000 (Invitrogen) was used to co-transfect Ishikawa cells with *SF3B1* siRNA or Control siRNA and pEGFPC1-hKSR2 or pEGFPC1-empty vector. After 36 h, cells were counted and re-plated in 96-well plates, and the relative proliferation rate was evaluated with the MTT proliferation kit at 0, 24, and 48 h. Experiments were performed in triplicates.

### Statistics

A two-tailed paired Student’s *t*-test was used to test for statistical significance, defined as *P* < 0.05. One-way analysis of variance (ANOVA) was used for multiple group comparisons. Asterisks represent the level of significance: *P = 0.05, **P = 0.01, ***P = 0.001, and ****P = 0.0001.

A detailed description of the materials and methods used in this study is available in the online Supplementary Material.

## Results

### SF3B1 expression is elevated in endometrial cancer

**Fig. 1:**
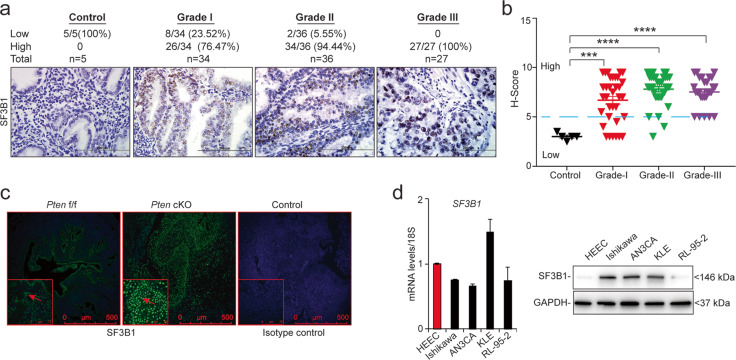
SF3B1 is overexpressed in endometrial cancer cells. **a** Representative images (×40) of immunohistochemical SF3B1 staining (brown) of control and endometrial cancer tissues in an endometrial carcinoma tissue array. **b** Quantitative analysis of SF3B1 expression in normal, grade I, II, and III endometrial tissue samples scored according to staining intensity and number of positive cells [low (score ≤ 5) or high (score ≥ 5)]. Statistical significance was assessed using one-way analyses of variance (ANOVA) and data are presented as mean ± SEM. ***P < 0.001, ****P < 0.0001. **c** Representative immunofluorescence images of SF3B1 staining (green) of uterine tissues from 3-month-old *Pten*^f/f^ or *PR*^Cre/+^*Pten*^f/f^ (*Pten* cKO) mice, which spontaneously develop endometrial carcinoma one month after birth. **d** SF3B1 transcript and protein levels in normal human endometrial epithelial cells (HEEC) and indicated endometrial cancer (EC) cell lines. GAPDH used as a protein loading control.

To investigate whether SF3B1 was involved in endometrial tumorigenesis, we first examined its expression in a tissue microarray containing 102 human endometrial cancer samples of various tumor grades. SF3B1 expression was higher in grades I, II, and III endometrial tumors than in normal endometrial tissues (Fig. 1a, b). Importantly, we observed a grade-dependent increase in SF3B1 expression with the highest expression in Grade III tumors. The distribution of cases with higher SF3B1 expression as follows: Grade I, 26/34 (76%), Grade II, 34/36 (94%), and Grade III, 27/27 (100%). Consistent with this, we found that tumors in a genetic mouse model of endometrial cancer (*PR*^Cre/+^*Pten*^*flox/flox*^) overexpress SF3B1 protein (Fig. 1c). Next, we compared SF3B1 mRNA and protein expression in primary human endometrial epithelial cells (our experimental control) and three Type 1 endometrial cancer cell lines: Ishikawa cells (isolated from a well-differentiated tumor), RL-95-2 cells (from a moderately differentiated tumor), and AN3CA cells (from a metastatic undifferentiated tumor). Additionally, we examined SF3B1 expression in one Type II endometrial cancer cell line, KLE (from a poorly differentiated tumor). Although *SF3B1* mRNA was only elevated in KLE cells, SF3B1 protein was more highly expressed in Ishikawa, AN3CA, and KLE cells than in control cells (Fig. 1d). Importantly, we performed full exon sequencing of *SF3B1* in Ishikawa, AN3CA, and RL-95-2 cells (data not shown) and found no *SF3B1* missense mutations in any of these cell lines. These data indicate that endometrial cancer cell lines overexpress SF3B1 protein in the absence of mutations or elevated mRNA overexpression. Together, these results demonstrate that SF3B1 expression is elevated in endometrial cancer at protein level.

### SF3B1 is required for endometrial cancer cell proliferation and cell cycle progression

**Fig. 2:**
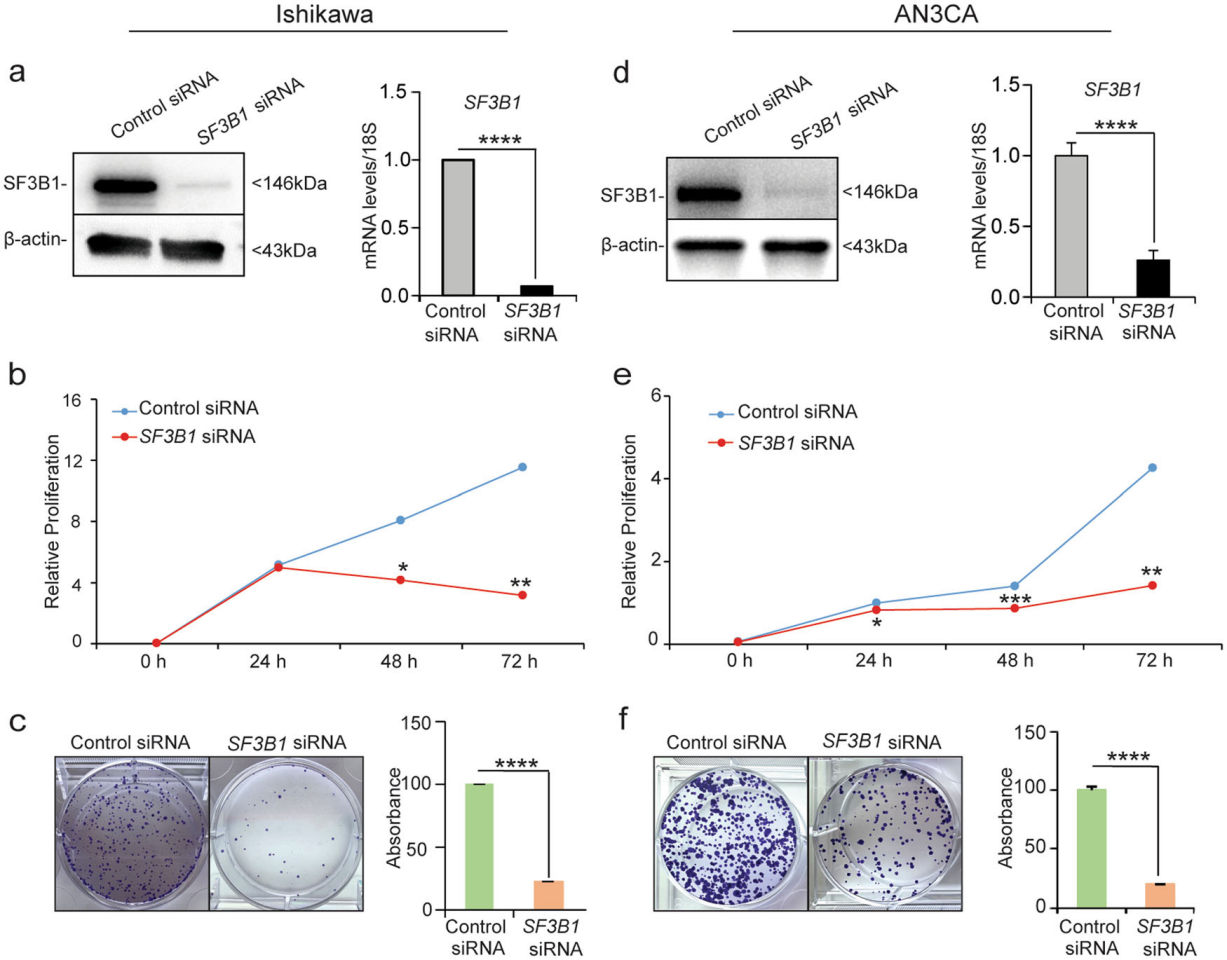
SF3B1 enhances endometrial cancer cell proliferation. **a, d** SF3B1 protein and transcript levels of *SF3B1* in Ishikawa (**a**) and AN3CA (**d**) cells transfected with control siRNA or *SF3B1* siRNA. **b**, **e** Representative MTT proliferation assays in Ishikawa (**b**) and AN3CA (**e**) cells. **c**, **f** Representative clonogenic assays (left) and quantitation (right) in Ishikawa (**c**) and AN3CA (**f**) cells. Data are presented as mean ± SEM. *n* = 3; ***P < 0.001, ****P < 0.0001.

Given that SF3B1 expression was elevated in endometrial cancers, we wondered whether SF3B1 promotes endometrial cancer cell proliferation. To investigate this, we transiently transfected Ishikawa and AN3CA cells (the cell lines in which SF3B1 was most highly expressed) with control or *SF3B1-*targeted small interfering RNA (siRNA). Western blotting and quantitative real-time PCR (Fig. 2a, d) confirmed that the siRNA substantially reduced SF3B1 protein and mRNA expression. After 48 h, we re-plated the cells and followed proliferation for 72 h and found that *SF3B1* siRNA significantly decreased the proliferation of Ishikawa and AN3CA cells (Fig. 2b, e). In addition, clonogenic assays revealed that *SF3B1* knockdown reduced Ishikawa cells proliferation by 84% and AN3CA cell proliferation by 81% (Fig. 2c, f). These results suggested that SF3B1 promotes endometrial cancer cell proliferation.

**Fig. 3:**
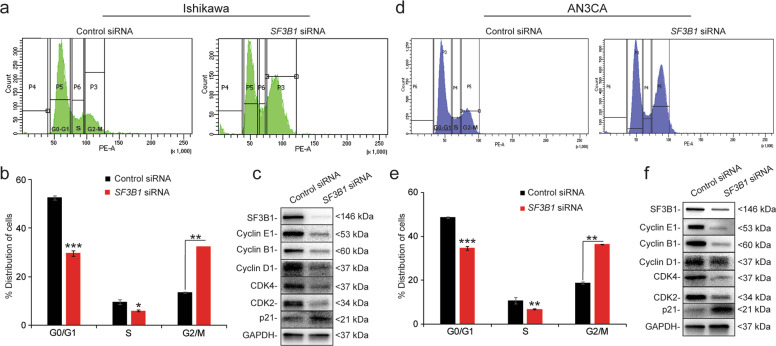
Loss of SF3B1 causes G2/M cell cycle arrest in endometrial cancer cells. **a**, **d** Ishikawa and AN3CA cells transfected with control or *SF3B1* siRNA for 72 h and then subjected to flow cytometry analysis of cell cycle. Histograms depict the percentages of cells in each phase of the cell cycle. **b**, **e** Graphs representing the distribution of cells in indicated phases of the cell cycle. **c**, **f** Western blotting of cell cycle regulatory proteins in Ishikawa (**c**) and AN3CA (**f**) cells transfected with control or *SF3B1* siRNA. Data are presented as mean ± SEM. *P < 0.05, **P < 0.01, ***P < 0.001.

Next, we evaluated the effect of *SF3B1* knockdown on cell cycle progression. Flow cytometry analysis revealed that, compared to cells transfected with control siRNA, a much greater proportion of Ishikawa and AN3CA cells in which *SF3B1* was knocked down were arrested in the G2/M phase of the cell cycle, with a concomitant reduction in the percentage of cells in the G1 phase (Fig. 3a, b, d, e). These results were consistent with findings of Dolatshed et al.^25^ that *SF3B1* knockdown caused G2/M cell cycle arrest in hematopoietic stem and progenitor cells in myelodysplastic syndrome. Next, we examined the expression of the cell cycle regulatory proteins: Cyclin D1 (required throughout the cycle), Cyclin E1 (S-phase transition), Cyclin B1 (transition from G2 to M), p21 (cell cycle inhibitor), CDK4 (progression through G1 phase), and CDK2 (progression through G1 phase)^27^. Expression of Cyclins B1, E1, and D1 was efficiently reduced in cells in which *SF3B1* was knocked down, whereas the expression of p21 was elevated (Fig. 3c, f). These results suggested that *SF3B1* silencing reduced Ishikawa and AN3CA cell proliferation by inducing G2/M cell cycle arrest.

### SF3B1 is required for tumor cell migration and invasion

**Fig. 4:**
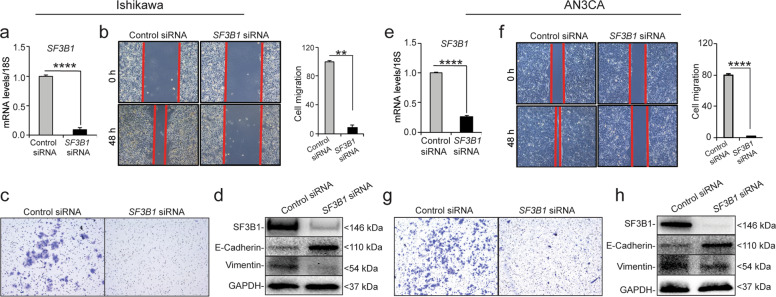
SF3B1 promotes tumor cell migration and invasion. **a**, **e** Confirmation of *SF3B1* mRNA depletion in Ishikawa and AN3CA cells transfected with Control or *SF3B1* siRNA. **b**, **f** Representative scratch assays in Ishikawa (**b**) and AN3CA (**f**) cells transfected with control or *SF3B1* siRNA. **c**, **g** Representative microscopic images (×10) of Ishikawa (**c**) and AN3CA (**g**) cells that invaded through the transwell in the matrigel invasion assay. **d**, **h** Expression analysis of proteins associated with cell migration and invasion. Data are presented as mean ± SEM. **P < 0.01, ****P < 0.0001.

Metastasis, which occurs after cells undergo the epithelial-to-mesenchymal transition (EMT), is the leading cause of most cancer-related deaths^28–31^. To undergo EMT, tumor cells must reduce their expression of E-cadherin and increase the expression of vimentin to become capable of migrating and invading other tissues^28–31^. To determine whether *SF3B1* was required for tumor cell migration, we transfected Ishikawa and AN3CA cells with *SF3B1* siRNA or control siRNA, confirmed that *SF3B1* mRNA expression was efficiently knocked down (Fig. 4a, e), and then performed wound-healing scratch assays. At 48 h post-scratch, Ishikawa and AN3CA cells expressing *SF3B1* siRNA were significantly less migratory than cells expressing control siRNA (Fig. 4b, f). The migration capacity of *SF3B1* depleted Ishikawa and AN3CA when calculated was 70–80% less than their control siRNA cells respectively (Fig. 4b, f).

To evaluate whether SF3B1 was required for invasion, we performed an assay in which cells transfected with *SF3B1* siRNA or Control siRNA were placed into the upper chamber of matrigel-coated transwells. We then stained the cells that invaded through the matrigel into the lower chamber and found that, compared to cells transfected with control siRNA, fewer cells transfected with *SF3B1* siRNA were able to invade through matrigel (Fig. 4c, g). Next, we examined the expression of adhesion markers: E-cadherin and vimentin associated with EMT. We found that cells in which *SF3B1* was knocked down had higher E-cadherin expression and lower vimentin expression than control siRNA-treated cells (Fig. 4d, h). Together, these results suggest that SF3B1 promotes migration and invasion behaviors of endometrial cancer cells.

### The SF3B1 inhibitor Pladienolide-B reduces endometrial cancer cell proliferation, migration, and invasion in vitro

**Fig. 5:**
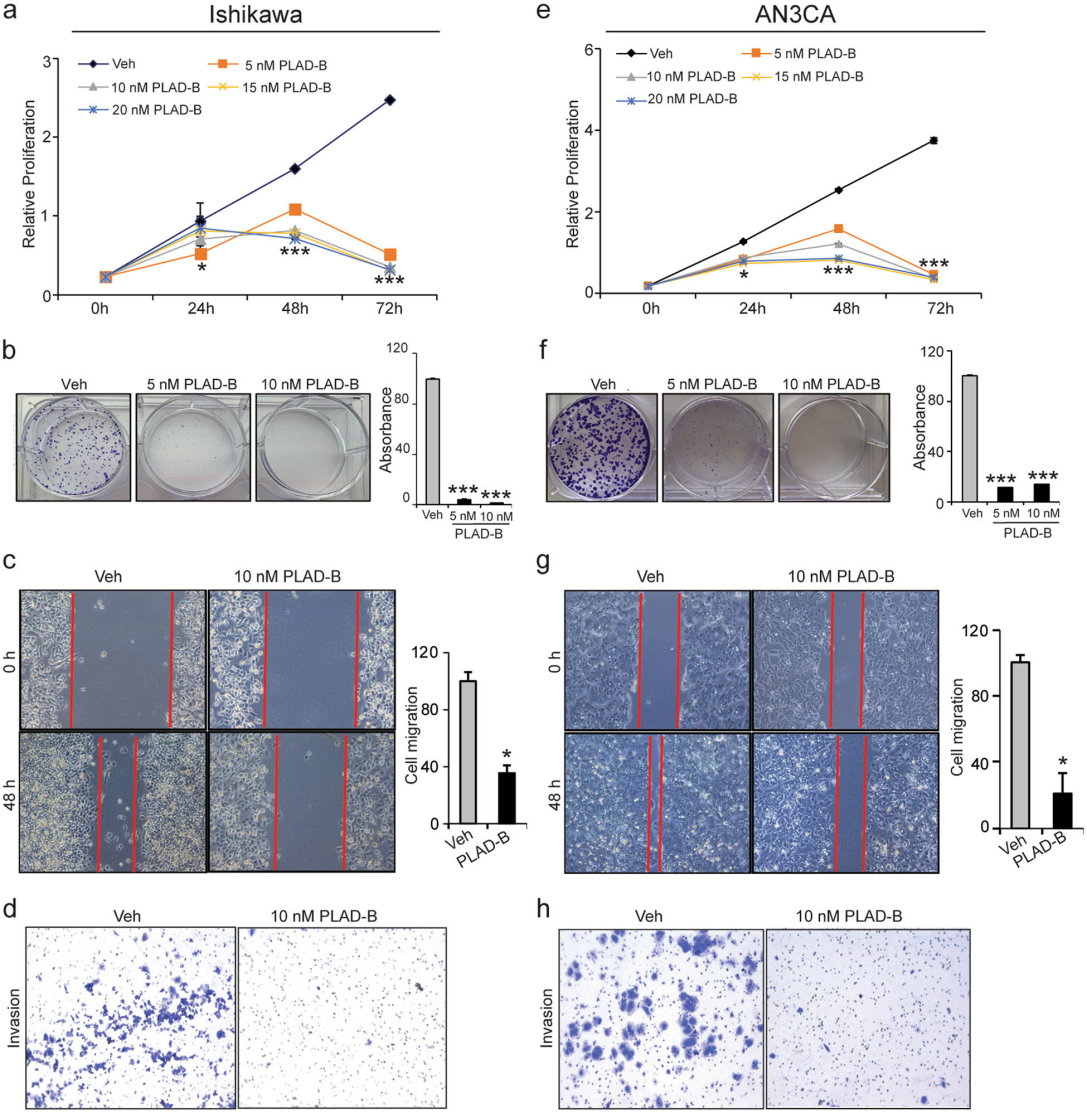
PLAD-B inhibits endometrial cancer cell growth in vitro. **a, e** MTT cell proliferation assay of cells treated with vehicle or increasing concentrations of PLAD-B at indicated time points. **b**, **f** Representative clonogenic assays of cells treated with vehicle or PLAD-B for 10–14 days. The right panel shows quantification of stained colonies by spectrophotometry at 490 nm. **c**, **g** Wound-healing assay of Ishikawa and AN3CA cells treated with vehicle or PLAD-B at 80–90% confluence. The right panel shows quantification of relative migrated area of vehicle- and PLAD-B-treated Ishikawa and AN3CA cells. **d**, **h** Representative microscopic images of Ishikawa and AN3CA cells treated with vehicle or PLAD-B that invaded through the transwell in the matrigel invasion assay. Data are presented as mean ± SEM. *P < 0.05, **P < 0.01, ***P < 0.001, ****P < 0.0001.

PLAD-B is a macrocyclic lactone originally obtained from *Streptomyces platensis* Mer-11107, strain isolated from a soil sample collected in Kanagawa, Japan (accession number FERM P-18144; Bioconsortia Program Laboratory National Institute of Advanced Industrial Science and Technology, Japan)^32–34^. PLAD-B is spliceosome inhibitor that specifically targets the SF3B1 spliceosome subunit^35^ and has anti-tumor activity in multiple cancers^35,36^. For example, when immunocompromised mice were implanted with primary cultured cells from gastric cancer patients, the tumors regressed with PLAD-B treatment^36^. Thus, we determined the effect of PLAD-B on in vitro proliferation, migration, and invasion of endometrial cancer cells. Both Ishikawa and AN3CA cells were treated with vehicle or increasing concentrations of PLAD-B, and cell proliferation was measured 0, 24, 48, and 72 h later. As expected, PLAD-B substantially decreased the proliferation of Ishikawa and AN3CA cells in a dose-dependent manner with IC_50_ of ∼10 nM (*p* < 0.01) (Fig. 5a, e). Similarly, in clonogenic assays, PLAD-B substantially reduced the clonal growth of Ishikawa and AN3CA cells (Fig. 5b, f). Next, we examined the effect of PLAD-B on cell migration and invasion. Scratch assays revealed that PLAD-B reduced the migration of Ishikawa cells by 60% and of AN3CA cells by 80% (Fig. 5c, g). In the invasion assay, Ishikawa and AN3CA cells treated with PLAD-B failed to invade through matrigel (Fig. 5d, h). These data indicate that PLAD-B efficiently reduces the oncogenic potential of SF3B1 in endometrial cancer cells. Additionally, we also evaluated the effect of *SF3B1* knockdown on KLE cells viability, migration, and cell cycle progression and found the similar effects as we observed in Ishikawa and AN3CA cells (Fig. S1). Moreover, consistent with minimal SF3B1 protein levels, RL-95-2 cells showed diminished impact of *SF3B1* knockdown or PLAD-B treatment on cell viability and migration in comparison to other three endometrial cancer cells that have robust SF3B1 protein levels (Fig. S2). Importantly, whereas *SF3B1*-depleted RL-95-2 cells are in a growing phase still at 72 h, three other endometrial cancer cell lines with high SF3B1 levels were in diminished cell growth state (Fig. S2).

### PLAD-B inhibits endometrial tumor growth in vivo

**Fig. 6:**
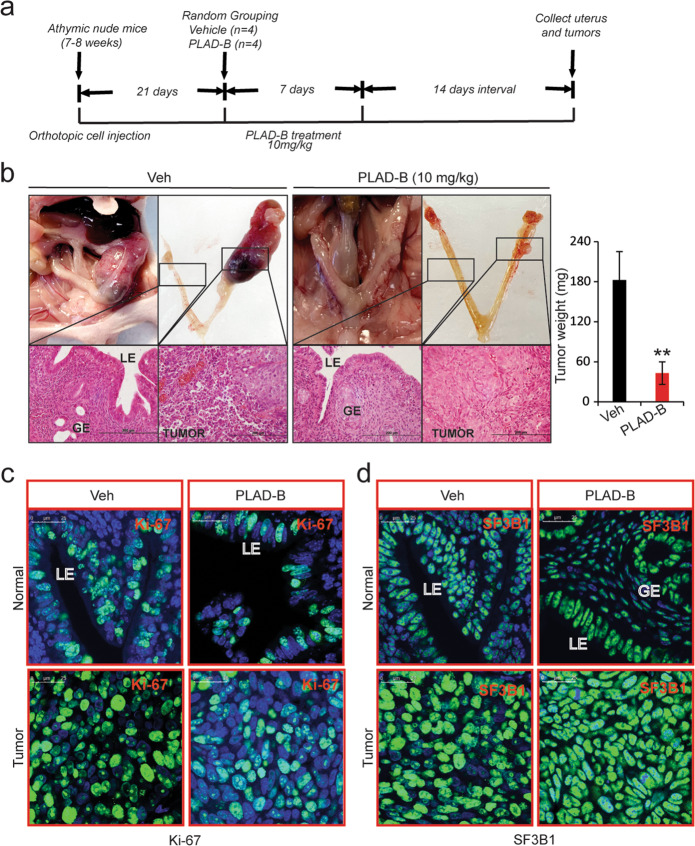
PLAD-B inhibits endometrial tumor growth in vivo. **a** Schematic representation of experimental procedure. **b** Representative images of uteri from mice treated with vehicle or PLAD-B and H&E-stained uterine horns from vehicle- or PLAD-B-treated animals (left). Quantification of tumor weights from vehicle- or PLAD-B-treated mice at endpoint (Right). **c**, **d** Representative confocal immunofluorescent cross-sectional images of uteri from vehicle- or PLAD-B-treated animals stained for Ki-67 and SF3B1; scale bar: 200 μm. LE luminal epithelia, G glandular epithelia. Normal, control un-injected horn; tumor, AN3CA cell-injected uterine horn.

We next sought to assess the activity of PLAD-B in vivo in an orthotopic model of endometrial cancer. AN3CA cells (2 × 10^6^) were injected into the right uterine horns of mice, leaving the left uterine horns untreated as controls. After 3 weeks, mice were randomized to receive either vehicle (DMSO) or 10 mg/kg PLAD-B intraperitoneally every other day for 1 week^36^. Two weeks later, mice were euthanized and uteri were collected (Fig. 6a). In the vehicle group, the tumor-cell-injected uterine horns were ~10 times larger than the control uterine horns (Fig. 6b, left images). Histological evaluation revealed normal histology with intact endometrial glands in the left uterine horn and a solid growing primary tumor in the right uterine horn (Fig. 6b, left images). The tumors showed characteristics of grade 3 endometrioid endometrial cancers, with areas of necrosis and myometrial invasion. In the PLAD-B group, the control and tumor-cell-injected horns were similar in size (Fig. 6b, right images). Additionally, the tumor-cell-injected horns in the PLAD-B group had a typical uterine structure with intact endometrial glands (Fig. 6b, right images). However, we noted the remnants of regressing tumors in the right uteri of PLAD-B-treated mice (Fig. 6b, right images). Quantification analysis showed that tumor weight reduced ~76% in PLAD-B-treated mice compared to vehicle-treated mice (Fig. 6b). These results suggest that PLAD-B effectively reduced the tumor growth. However, PLAD-B appeared to be non-toxic, as we did not notice any differences in behavior, feeding habits, or mobility between vehicle- and PLAD-B-treated mice (data not shown).

To begin to uncover how PLAD-B reduced tumor growth, we examined the expression of proliferation marker Ki-67. In the control uterine horns of vehicle-treated mice, Ki-67-positive cells were evident in the luminal and glandular epithelium. The tumor-cell-injected uterine horns had substantially more Ki-67-positive cells, indicating the growth of a solid tumor (Fig. 6c). The tumor-cell-injected horns in PLAD-B treated mice had substantially fewer Ki-67-positive cells than the corresponding horns in vehicle-treated mice (Fig. 6c) indicating that PLAD-B efficiently reduced tumor cell proliferation. PLAD-B also appeared to inhibit normal cell proliferation, as we saw fewer Ki-67-positive cells in the normal horns of PLAD-B-treated mice than in the corresponding horns of vehicle-treated mice (Fig. 6c, right images).

We wondered whether PLAD-B affected SF3B1 expression and thus stained the uterine horns from the two groups of mice with an SF3B1-specific antibody. In the vehicle-treated mice, the tumor-cell-injected horns had substantially higher SF3B1 expression than the corresponding normal horns (Fig. 6d). SF3B1 expression was mainly restricted to the luminal and glandular epithelium in control uteri (Fig. 6d). As expected, we did not notice any difference in SF3B1 expression between the tumor-cell-injected horns in the vehicle and the PLAD-B-treated mice, indicating that PLAD-B inhibits SF3B1 activity but not expression. Together, these findings suggest that SF3B1 could be targeted to reduce endometrial cancer growth.

### Identification of SF3B1-driven transcriptome and alternative splicing events in endometrial cancer cells

**Fig. 7:**
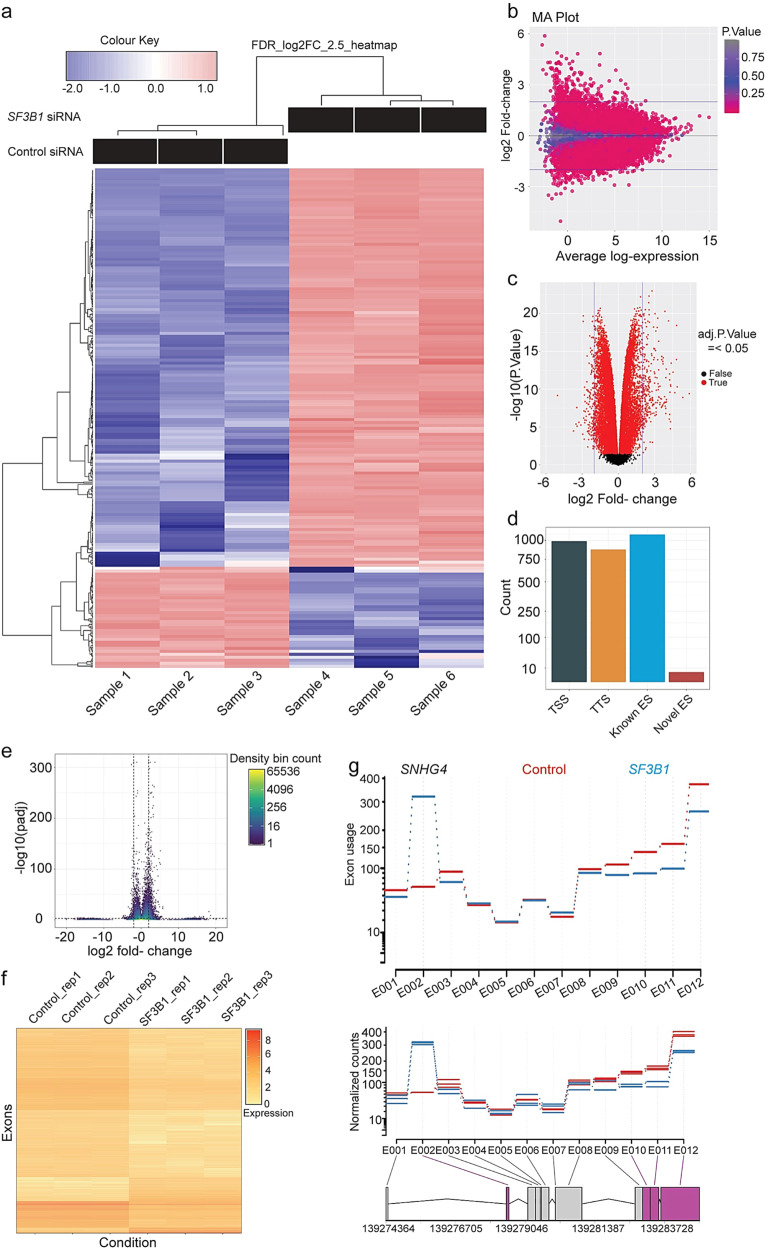
*SF3B1* depletion alters the transcriptome and AS events in EC cells. **a** Heat map of mRNAs differentially expressed between control and *SF3B1* knockdown Ishikawa cells with cutoff of FDR < 0.01 and logFC > 2.5; *n* = 3 each group. **b** MA plot showing the appropriate normalization of RNA-seq data. **c** Volcano plots for all the DEGs in each comparison. The left side indicates downregulated genes and the right side indicates that upregulated DEGs with *p* values < 0.05. **d** Summary of alternative exon usage by alternative transcription category. **e** Volcano density plot of exons’ FDR corrected adjusted *p* value and log2 fold change of *SF3B1* knockdown vs. control. Color represents the density of each bin. Dashed lines represent significance thresholds. **f** Heat map depicting normalized counts for exons with sufficient coverage. Color represents log2(*x* + 1)-transformed normalized counts. **g** Exon usage and normalized counts for control and *SF3B1* knockdown conditions for ENSG00000281398 [SNHG4]. Color (purple) in the gene map indicates significant exons.

Since overexpressed SF3B1 promotes endometrial tumorigenesis both in vitro and in vivo, we next investigated the underlying mechanism. Toward this, we determined the *SF3B1* transcriptome by performing RNA-seq analysis from Ishikawa cells depleted with *SF3B1*. As shown in Fig. 7a, hierarchical clustering revealed a distinct SF3B1-dependent transcriptome in in Ishikawa cells. Using a 2.5-fold cutoff and Benjamini–Hochberg false discovery rate (FDR) of <0.01 threshold for inclusion, we identified 1992 differentially expressed genes (DEGs) between Control and *SF3B1*-depleted Ishikawa cells (Fig. 7c and Supplementary Table 1). Further, Gene Ontology (GO) enrichment analysis revealed that SF3B1 regulated DEGs enriched in several biological processes, including cell adhesion, angiogenesis, and cytokine production involved in immune response, and ATP binding (Supplementary Table 2). Importantly, pathway analysis has been shown that much of the DEGs enriched in calcium signaling pathway, Ras signaling, and metabolic pathways (Supplementary Table 2). This enrichment analysis suggests that SF3B1 overexpression promotes pro-cancer-specific transcriptome in endometrial cancer.

Further, to gain insight into the spectrum of genes that are aberrantly spliced, we used DEXSeq to perform differential exon usage analysis of our RNA-seq data. DEXseq analyzed 6,40,456 exons for differential exon usage. A complete set of DEXseq raw counts, normalized counts, and statistics for every exon is provided as Supplementary Table 3 (annotations for each exon are provided in Supplementary Table 4). Summarization of the exons disrupted suggested that *SF3B1* depletion leads to multiple categories of alternative splicing including alternative exon skipping (ES), transcript start site, and transcript termination site usage (Fig. 7d). A total of 3840 exons exhibited significant loss or gain, determined by FDR corrected adjusted *p* value cutoff of 0.01 and minimum log2 fold change >2, which affected 2160 total gene loci (Fig. 7e). Unsupervised hierarchical clustering was performed on exons with sufficient coverage and variance. Replicates in the control and *SF3B1* knockdown conditions clustered together tightly, indicating a consistent and predictable deregulation of normal splicing upon *SF3B1* knockdown (Fig. 7f). Specific novel alternative splicing events induced by *SF3B1* were readily identifiable and ranged from relative simple alternative ES (e.g. Fig. 7g) to much more complex patterns. Overall, the alternative splicing analysis revealed wide scale disruption of alternative splicing across the transcriptome.

### Validation of DEGs identified by RNA sequencing

**Fig. 8:**
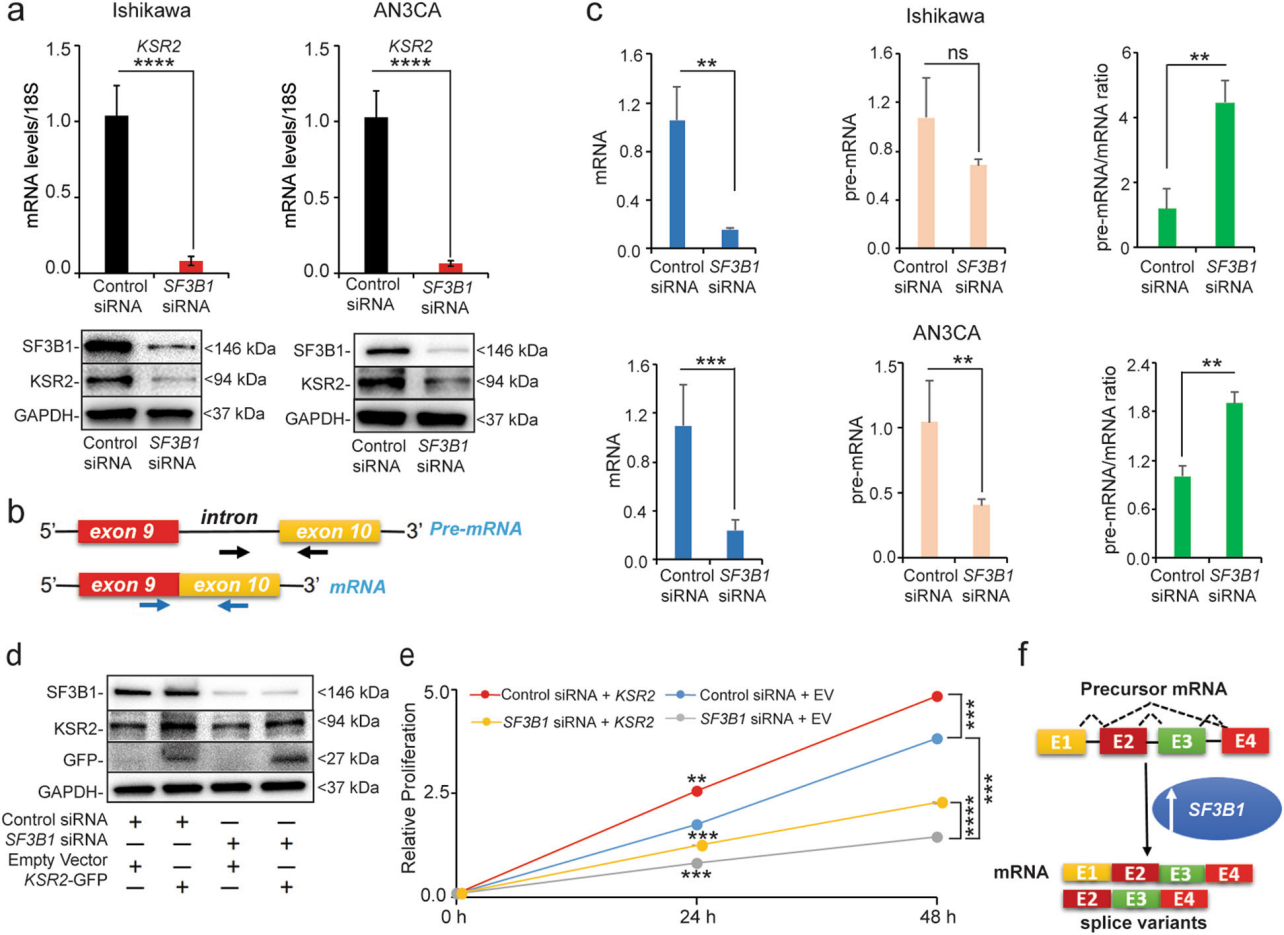
SF3B1 regulates *KSR2* mRNA maturation and *KSR2* mediates SF3B1-driven proliferation. **a** qRT-PCR and western blot analysis to detect mRNA (upper panel) and protein levels (lower panel) of KSR2 in Ishikawa and AN3CA cells transfected with *SF3B1* or Control siRNA. **b** Schematic representation of strategy to identify intron–exon (unspliced, pre-mRNA) and exon–exon (spliced, mature RNA) junction sites using qRT-PCR. Specific primers designed are represented by the two arrow pairs, illustrating their approximate locations. **c** Relative levels of unspliced and spliced *KSR2* transcripts and their ratio in Ishikawa and AN3CA cells transfected with Control or *SF3B1* siRNA. β-actin was used for normalization. Data are presented as mean ± SEM. *P < 0.05, **P < 0.01, ***P < 0.001, ****P < 0.0001. **d** Confirmation of *SF3B1* knockdown and KSR2 overexpression in Ishikawa cells co-transfected with *SF3B1* siRNA or KSR2 plasmids. **e** Cell proliferation assay in Ishikawa cells co-transfected with *SF3B1* siRNA or KSR2 plasmids at indicated times. **f** Schematic illustration of the hypothesis showing how overexpressed SF3B1 might promote endometrial cancer progression by generating transcript variants and mature mRNAs.

Given the wide scale alterations across transcriptome with *SF3B1* knockdown (Fig. 7a), next we aimed to find a specific DEGs that might be a candidate downstream mediators of SF3B1 action. For this, we narrowed seven DEGs based on their biological functions. Out of these seven DEGs, five (*KSR2, GJD3, TRAF1, TBC1D16,* and *DOCK11*) were downregulated and (*FOXQ1, MYLK2*) two were upregulated (Fig. S3). First, we validated the expression of these seven altered genes with *SF3B1* knockdown in both Ishikawa and AN3CA cells (Fig. S3). Since, Ras signaling is the primary mitogenic pathway of endometrial cancer, we decided to focus on *KSR2*, a Ras signaling promoting gene for subsequent studies (Fig. S4). KSR2 is a molecular scaffold protein shown to be critical in the integration of various mitogenic and metabolic pathways^37–39^. As expected, we found SF3B1 regulated the KSR2 expression in endometrial cancer cells both at transcript and protein levels (Fig. 8a).

### KSR2 contributes to SF3B1-dependent proliferation in endometrial cancer cells

SF3B1 is an important regulator of pre-mRNA machinery^24,40,41^ and we found that *KSR2* transcript levels were abrogated with *SF3B1* knockdown (Fig. 8a). Therefore, we wondered whether SF3B1 is required for maturation of *KSR2* pre-mRNA to mature mRNA. To test this, we measured mature (spliced) and immature (unspliced) *KSR2* mRNAs using intron–exon and exon–exon junction-specific primers (Fig. 8b). While knockdown of *SF3B1* abrogated mature *KSR2* mRNAs in both Ishikawa and AN3CA cells, *KSR2* pre-mRNAs remained stable in Ishikawa cells and reduced modestly in AN3CA cells (Fig. 8c). This reduced level of pre-mRNA might be due to splicing coupled transcription defect^42^ or due to reduced stability of pre-mRNA owing to intron retention (splicing defect)^43^. Importantly, the relative ratio of pre-mRNA/mRNA was significantly higher with *SF3B1* knockdown in both the Ishikawa and AN3CA cells, indicating that the reduced splicing efficiency in the absence of SF3B1 lead to an accumulation of pre-mature *KSR2* RNA. Finally, to determine whether KSR2 contributes to overall SF3B1 function, we performed a rescue experiment by co-transfecting the Ishikawa cells with siRNA and human KSR2 plasmid and determined the cell proliferation. As expected, KSR2 protein levels abrogated with *SF3B1* knockdown restored with KSR2-GFP plasmid transfection in Ishikawa cells (Fig. 8d). Importantly, rescuing the KSR2 protein in *SF3B1*-depleted cells partially restored the reduced cell proliferation due to *SF3B1* knockdown (Fig. 8e). Taken together, these data suggest that SF3B1 exerts its functions by generating mature splice variants and KSR2 is one such downstream effectors of SF3B1 function in endometrial cancers (Fig. 8f).

## Discussion

Here, we present several pieces of evidence indicating that the splicing factor SF3B1 plays an important role in endometrial cancer pathogenesis. First, we found increased SF3B1 protein expression in human endometrial tumors and three endometrial cancer cell lines. This is consistent with multiple reports that showed an elevated expression of the other splicing factors in several human cancers^8^. Second, our in vitro data demonstrated that SF3B1 promotes endometrial cancer cell proliferation, cell cycle progression, migration, and invasion. Third, our in vivo data indicated that SF3B1 inhibitor, PLAD-B, reduces endometrial tumor growth in mice. Finally, we found that SF3B1 mediates pro-proliferative function partially by driving *KSR2* mRNA maturation.

In our study, decreased cell viability and associated cell cycle regulators expression in *SF3B1*-depleted cells highlights its pro-proliferative role in endometrial cancer. Interestingly, a recent report suggested that aberrant splicing by mutant *SF3B1* altered the transcriptome, proteome, and metabolome of human cells and caused mis-splicing of various associated metabolic genes^44^. Here, we also found *SF3B1* transcriptome enriched with several metabolic process, suggesting a possible role for *SF3B1* in metabolic programming in cancers. It is well-recognized that SF3B1 regulate alternative splicing of several crucial genes involved in cell survival pathways implying its oncogenic potential in promoting tumorigenesis^24,40,41^. Consistently, we found wide scale disruption of alternative splicing across the transcriptome, suggesting that *SF3B1-*driven alternative splicing has a pathogenic significance in endometrial cancer. Interestingly, we identified differential exon usage in one of the important small nucleolar RNAs (snoRNAs), the *SNHG4*. Previous studies demonstrated the overexpression of numerous snoRNAs in several cancers and their role in promoting proliferation, cell cycle progression, invasion, and metastasis^45–47^. Therefore, investigating the role of snoRNAs in endometrial cancer is quite intriguing; however, in-depth functional studies are required to dissect the role of such potential alternative spliced variants in endometrial cancer tumorigenesis.

Several lines of evidence suggest that *PTEN*, a tumor suppressor gene is frequently mutated in −30 to 50% of endometrial cancers^48–50^. Increased SF3B1 levels in tumors originated due to PTEN loss suggest that overexpression of SF3B1 coupled with PTEN loss might drive the endometrial cancer progression. However, more in-depth studies are required to test whether SF3B1 overexpression and PTEN loss are mutually exclusive in endometrial cancers. Importantly, these studies might provide molecular underpinnings of endometrial cancer initiation and progression.

Previous studies have shown that attenuation in splicing factor-driven RNA maturation promotes tumorigenesis through the accumulation of pre-mRNAs^51,52^. We found decreased expression of KSR2 due to impairment in SF3B1-mediated mRNA maturation and KSR2 partially contributes to SF3B1 pro-proliferative function. Given, multitude of genes impacted by mis-splicing due to splicing factor dysfunctions, the downstream functional impact is unlikely due to the altered splicing of a single gene variant. Instead, splicing factor deregulation may cause broad splicing aberrations resulting in induction of distinct transcriptional signature(s) that promote tumor progression. Thus, the partial contribution of KSR2 to overall SF3B1 function in our study indicates that SF3B1 exerts its functions through numerous downstream effector genes. For example, we found many of the SF3B1-regulated genes associated with metabolic process (Supplementary Table 2). Therefore, future studies dovetailing transcriptomics and metabolomics may provide better understanding on the functional role of SF3B1 in endometrial cancer progression.

SF3B1 is a target of PLAD-B, a natural product with anti-tumor activity in both cancer cell lines and mouse xenograft models^41,53,54^. By impairing the binding of the spliceosome to the branch sequence, PLAD-B causes an accumulation of unspliced mRNA in cells, resulting in reduced viability^35,55^. Here, we found that PLAD-B decreased endometrial cancer cell proliferation, migration, and invasion in vitro. Furthermore, we found that PLAD-B reduced the endometrial cancer burden in our orthotopic endometrial cancer model, suggesting that PLAD-B, or a derivative, might be a viable therapeutic with which to treat endometrial cancer.

In summary, we report that SF3B1 plays an important role in endometrial cancer initiation and progression possibly through regulating *KSR2* mRNA maturation. Thus, uncovering the precise role of SF3B1 in the progression of endometrial cancer will increase our knowledge of spliceosome function in endometrial cancer.

## Supplementary information

Supplemental Material

Fig. S1

Fig. S2

Fig. S3

Fig. S4

Supplementary Table 1

Supplementary Table 2

Supplementary Table 3

Supplementary Table 4

Supplementary Table 5

Supplementary Table 6
