# Supplementary material for: Splicing factor SF3B1 promotes endometrial cancer progression via regulating KSR2 RNA maturation
Source: Cell Death Dis. 2020 Oct 10;11(10):842. doi: 10.1038/s41419-020-03055-y (PMC7548007; doi:10.1038/s41419-020-03055-y)
Supplement: Supplementary file 1 — Supplemental Material [file 41419_2020_3055_MOESM1_ESM.docx]

**Splicing factor SF3B1 promotes endometrial cancer progression via regulating KSR2 RNA maturation**

Pooja Popli^1^, Megan M. Richters^2,3^, Sangappa B. Chadchan^1^, Tae Hoon Kim^7^, Eric Tycksen^3^, Obi Griffith^2,3,4,5^, Premal H. Thaker^6^, Malachi Griffith^2,3,4,5^, and Ramakrishna Kommagani^1,*^

^1^Department of Obstetrics and Gynecology, Center for Reproductive Health Sciences,

^2^Division of Oncology, Department of Medicine,

^3^Genome Technology Access Center, McDonnell Genome Institute,

^4^Department of Genetics,

^5^Siteman Cancer Center,

^6^Division of Gynecologic Oncology, Department of Obstetrics and Gynecology,

Washington University School of Medicine, St. Louis, MO, 63110, USA.

^7^Department of Obstetrics, Gynecology and Reproductive Biology

Grand Rapids, Michigan State University, MI, 48824, USA.

***Correspondence to**

Ramakrishna Kommagani, PhD

Department of Obstetrics & Gynecology

Center for Reproductive Health Sciences

Washington University School of Medicine

BJC Institute of Health - 10th Floor, RM 10606

425 S. Euclid Avenue Campus Box 8064

St. Louis MO 63110

Email: [kommagani@wustl.edu](mailto:kommagani@wustl.edu)

Phone: (314) 273-1638

Fax: (314) 747-0264

**Short title**: Role of SF3B1 in endometrial cancer

The authors have declared that no conflict of interest exists relating to this work.

This PDF file includes:

Supplementary Materials and Methods

Supplementary Legends

References for Supplementary Materials and Methods

**Supplementary materials and methods**

**Human endometrial epithelial cell isolation:** Endometrial biopsies from healthy reproductive-age women were obtained during the proliferative phase (days 8 to 12) of the menstrual cycle. Human endometrial epithelial cells (hEECs) were isolated from endometrial tissue biopsies and cultured as described previously ^1^. Briefly, endometrial biopsy tissue was digested in DMEM/F-12 medium containing 2.5 mg/ml collagenase (Sigma-Aldrich) and 0.5 mg/ml DNase I (Sigma-Aldrich) for 1.5 h at 37°C. Then, epithelial cells were separated from stromal cells by passing through a 0.4 µm cell strainer. Collected hEECs were resuspended in DMEM/F-12 media containing 10% FBS, 100 units/ml penicillin, and 0.1 mg/ml streptomycin and cultured as per standard tissue culture procedures ^1^.

**Tissue microarray immunohistochemistry:** A paraffin-embedded tissue microarray (TMA) from US Biomax containing 102 endometrial cancer specimens of various histologies and normal endometrium was processed as per the manufacturer’s protocol. Briefly, the TMA slide was baked for 30 to 120 minutes at 60°C, deparaffinized, rehydrated, and boiled for antigen retrieval. After blocking with 2.5% goat serum (Vector Laboratories) for 1 h at room temperature, slides were incubated overnight at 4°C with the anti-SF3B1 antibody (1:500, Abcam). Following washing, the slide was incubated for 1 hr with a biotinylated secondary antibody (1:1000, Vector Laboratories), washed, incubated for 45 min. with ABC reagent (Vector Laboratories), developed with DAB, and counter-stained with hematoxylin. SF3B1 expression in normal and cancerous endometrial tissue samples was scored according to staining intensity and number of positive cells (low score ≤5, high score >5) by two independent, blinded investigators.

**Western blotting:** Lysates containing 40 μg of protein were loaded on a 4-15% SDS-polyacrylamide gel (BioRad), separated with 1xTris-Glycine Running Buffer (BioRad), and transferred to PVDF membranes in a wet electro-blotting system (Bio-Rad), all according to the manufacturer’s directions. PVDF membranes were blocked for 1 hour in 5% non-fat milk in TBS-T (Bio-Rad), then incubated overnight at 4^o^C with antibodies listed in Supplementary Table 4 in TBS-T containing 5% BSA. After washing and incubating in horseradish peroxidase-conjugated anti-Rabbit IgG (1:5000, Cell Signaling Technology) in TBS-T plus 5% BSA for 1 hour at room temperature, blots were developed with horseradish peroxidase chemiluminescent substrates (Thermofisher Scientific) and imaged on a BioRad ChemiDoc imaging system.

**Cell proliferation assays:** Cell proliferation was determined by performing the MTT assay (Promega) according to the manufacturer's instructions. Briefly, Ishikawa, AN3CA, KLE or RL-95-2 cells were transfected with *SF3B1* siRNA or control siRNA. Then, 48 h post-transfection, cells were counted and re-plated in 96-well plates, and the relative proliferation rate was evaluated with the MTT proliferation kit at 0 h, 24 h, 48 h and 72 h. Similarly, for PLAD-B experiments, Ishikawa or AN3CA cells were plated in 96-well plates. After 24 h, cells were treated with vehicle (DMSO) or 0 nM, 5 nM, 10 nM, 15 nM, or 20 nM PLAD-B for 0 h, 24 h, 48 h and 72 h. Then, relative cell proliferation was determined by the MTT assay. The IC_50_ value for PLAD-B was calculated by GraphPad Prism. The experiments were performed three times with three replicates in each.

**Immunofluorescence:** Tissues from mouse endometrial orthotopic xenografts were fixed in 4% paraformaldehyde, and sections were subjected to immunofluorescence (n = 4 to 5 per group) as described previously ^1^. Briefly, tissue sections were deparaffinized, rehydrated, and boiled for antigen retrieval. Sections were blocked with PBS containing 2.5% goat-serum (Vector Laboratories) for 1 h, then incubated overnight in primary antibodies against SF3B1 (1:200, Abcam) and Ki-67 (1:200, Abcam). After washing with PBS, sections were incubated with Alexa Fluor 488-conjugated secondary antibodies (Life Technologies) for 1 h at room temperature, and mounted with ProLong Gold Antifade Mountant with DAPI (Thermo Scientific). For H&E staining, tissue sections were fixed, processed, embedded, and sectioned as above. After deparaffinization, sections were stained with hematoxylin and eosin as described previously ^1^. Similarly, uterine sections from 3-month-old *Pten*^f/f^ or *PR*^Cre/+^*Pten* mice (*Pten* cKO) (provided by Dr. Tae Hoon Kim, Michigan State University) were processed for SF3B1 immunofluorescence as described above.

**Clonogenic assay:** Ishikawa or AN3CA cells transfected with siRNA as described above were seeded at a cell density of 1x10^3^/well in a 6-well plate. After 10 (AN3CA) or 14 (Ishikawa) days, the colonies were fixed with 4% paraformaldehyde and stained with 0.1% crystal violet. Stained colonies were photographed and counted before the retained crystal violet stain was removed with 10% acetic acid; the stain eluate was quantitated by spectrophotometry at 490 nm. Similarly, for PLAD-B experiments, 1x10^3^ Ishikawa or AN3CA cells were seeded in a 6-well plate and grown for 24 h. Then, cells were treated with 5 nM or 10 nM PLAD-B or vehicle for 2 days. Afterwards, PLAD-B was removed and fresh media was added. Cells were allowed to form colonies for 14 days (Ishikawa) or 10 days (AN3CA), stained with crystal violet and quantified subsequently as described above.

**Wound-healing assay:** Ishikawa, AN3CA, KLE or RL-95-2 cells were plated in a 6-well plate and allowed to grow in 10% FBS-containing medium to 80-90% confluency. Cells were then transfected with *SF3B1* siRNA or Control siRNA. Six hours post-transfection, cells were scratched with a 10 μl pipette tip, washed twice with media, and incubated in a serum-free medium. Cells were imaged immediately and after 48 h by using a Leica DMi1 microscope. The percentage of migrated area relative to control was calculated. Similarly, for PLAD-B experiments, both Ishikawa and AN3CA cells were grown to 80-90% confluency, treated with 10 nM PLAD or vehicle (DMSO), scratched with a 10 µl pipette tip, and imaged 48 h later as described above.

**Transwell invasion assay:** Ishikawa, AN3CA or KLE cells were transfected with *SF3B1* siRNA or Control siRNA for 48 h and then seeded into the upper chamber of a matrigel-coated transwell (Corning BioCoat™ Matrigel Invasion Chamber). After 36 h, cells on the upper chamber were carefully removed with a cotton swab. The lower chamber was fixed with 4% paraformaldehyde, washed with PBS, and then stained with crystal violet. Invaded cells were imaged on a Leica DMi1 microscope. Similarly, for PLAD-B experiments, Ishikawa (2x10^5^) and AN3CA (1x10^5^) cells were seeded in 10 nM PLAD-B-containing media in a Corning BioCoat™ Matrigel Invasion Chamber. After 36 h, cells in the upper chamber were carefully removed with a cotton swab, and the lower chamber was processed and imaged as explained above.

**Flow cytometry:** Endometrial cancer cells (Ishikawa, AN3CA or KLE cells) were seeded (5x10^5^cells/ml) in 6-well plates and treated with control or *SF3B1* siRNA for 72 h. Post-transfection, cells were harvested, washed with phosphate-buffered saline (PBS), fixed in 70% chilled ethanol, and stained with 50 μg/ml propidium iodide (Sigma) containing 50 μg/ml RNAse. Cell cycle stage analysis was performed by flow cytometry (FACS Canto II) and FACS Diva software (ver. 8.0; BD Biosciences, Franklin Lakes, NJ, USA). The experiments were performed in triplicates.

**Plasmids:** The plasmids pEGFPC1-hKSR2 and pEGFPC1-empty vector ^2^ were kind gifts from Dr. I. Sadaf Farooqi (University of Cambridge Metabolic Research Laboratories and NIHR Cambridge Biomedical Research Centre, Cambridge CB2 0QQ, UK).

**RNA isolation and real-time polymerase chain reaction (qPCR):** Total RNA was isolated from endometrial cancer cells (Ishikawa, AN3CA, KLE or RL-95-2 cells) with the RNeasy total RNA isolation kit (Qiagen). One microgram of total RNA was reverse transcribed to cDNA with the TaqMan Reverse Transcription kit (Applied Biosystems). Quantitative real-time PCR analysis was performed with an ABI Prism 7700 sequence detection system with TaqMan 2X master mix (Applied Biosystems, USA) and validated primers (Applied Biosystems). Ribosomal RNA (18S) was used as an internal control. Primer sequences are shown in Supplementary Table 3.

For RNA maturation study, total RNA was isolated from Ishikawa and AN3CA cells transfected with *SF3B1* siRNA or Control siRNA with Trizol reagents (Qiagen). RNA was then treated with DNase I and reverse transcribed with random hexamers and M-MLV-RTase (Invitrogen). Relative mRNA amounts were quantified by real-time PCR, with β-actin served as a control. Primer sequences used are shown in Supplementary Table 3.

**RNA-sequencing:** Ishikawa cells were transfected with control or *SF3B1* siRNA and then 48 hours later, total RNA was isolated using RNeasy total RNA isolation kit (Qiagen) for RNA-seq. The library preparation and sequence for RNA-seq was performed at the McDonnell Genome Institute at Washington University in St. Louis. The Library was prepared using Kappa RiboErase rRNA removal kit and paired-end sequencing was performed on a Illumina HiSeq 3000 sequencers with 2×150 paired-end reads. Basecalls and demultiplexing were performed with Illumina’s RTA version 1.9 and bcl2fastq2 software with a maximum of one mismatch in the indexing read. RNA-seq reads were then aligned to the Ensembl release 76 primary assembly with STAR version 2.0.4b. Gene counts were derived from the number of uniquely aligned unambiguous reads by Subread:featureCount version 1.4.5.  The counts were then imported into the R/Bioconductor package EdgeR and TMM normalization size factors were calculated to adjust samples for differences in library size. Genes expressed less than one count-per-million in at least three sample were excluded from further analysis and new effective TMM size factors were recalculated. The rescaled matrix of counts were then imported into R/Bioconductor package Limma and weighted likelihoods based on the observed mean-variance relationship of every gene and sample were then calculated for all samples with the voomWithQualityWeights function. Generalized linear models were then created to test for differential expression and the results were then filtered for Benjamini-Hochberg FDR adjusted p-values less than or equal to 0.01 and linear fold change≥ 2.5 fold-change. All gene expression data have been deposited in the public data repository GEO (GSE156775).

Using the same STAR alignments used for differential gene expression analysis we performed differential exon usage analysis using the latest version (v1.34.1) of the alternative splicing analysis BioConductor^3^ tool DEXSeq^4^. To perform exon usage statistics DEXSeq requires raw read counts for individual exons. Human transcript and exon annotations were obtained in Gene Transfer Format (GTF) from build 100 of the Ensembl resource^5^. The exon annotations needed for DEXseq were calculated using the “dexseq_prepare_annotation.py” module from the DEXSeq package using the “--aggregate no” parameter. This parameter avoids the merging of exons from overlapping genes. The resulting annotating exon set in General Feature Format (GFF) contained 640,456 exon features for 59,428 genes. The “dexseq_count.py” module was used to generate exon counts for each of these features. This module uses the “htseq-count” command from the HTSeq package^6^ to perform the raw counting operations. “dexseq_count” was run with default parameters with the exception of: “--stranded no”, “--paired yes”, “--format bam”, and “--order pos”. The DEXseq python modules described above and the latest version of HTSeq (v0.12.4) were installed in a Docker container to perform this analysis. The image for this Docker container was publicly available in DockerHub (‘malachig/htseq-0.12.4’). Exon counts were loaded into the DexSeq package using R (v4.0.2) and RStudio (1.3.1056). The DEXSeq statistical analysis was performed as described in the DEXSeq Bioconductor Vignette provided with the package. To identify exons significantly gained or lost in the cohort, all exons were filtered by FDR corrected adjusted p-value cutoff < 0.01 and a minimum log2 fold change > 2. To subset exons for the heat map, raw counts were filtered to exclude those with low coverage (less than 2 replicates > 10 raw counts) and low variance (coefficient of variance (COV) [Std deviation / mean] < 0.7). Then, unsupervised hierarchical clustering was performed on normalized counts. All code used to perform this analysis and produce visualization for Fig. S3 is made publicly available in GitHub (<https://github.com/griffithlab/sf3b1_ishikawa>).

**Gene ontology and pathway enrichment analysis:** Gene ontology analysis (GO) and pathway enrichment analysis was done by Database for Annotation, Visualization and Integrated Discovery (DAVID) to visualize the DEGs enrichment of biological process (BP), molecular function (MF), cellular component (CC) and Kyoto Encyclopedia of Gene and Genome (KEGG) pathways (P < 0.05).

**Supplementary Legends**

**Fig. S1**: **Effect of *SF3B1* knockdown on proliferation, cell cycle, migration and invasion of KLE cells. a** SF3B1 protein and transcript levels in KLE cells transfected with control siRNA or *SF3B1* siRNA. Data are presented as mean ± SEM. *****P*<0.0001. **b** Representative MTT proliferation assays in KLE *SF3B1* knockdown and control cells **c** Flow cytometry analysis of cell cycle in KLE cells transfected with SF3B1 siRNA or Control siRNA for 72 h. Graphs representing the distribution of cells in indicated phases of the cell cycle. **d** Western blotting of cell cycle regulatory proteins in KLE cells transfected with *SF3B1* siRNA or control siRNA. **e** Representative scratch assays in KLE cells transfected with control or *SF3B1* siRNA. **f** Expression analysis of proteins associated with cell migration and invasion. **g** Representative microscopic images (10X) of KLE *SF3B1* or Control siRNA transfected cells that invaded through the transwell in the Matrigel invasion assay

**Fig. S2: Effect of *SF3B1* knockdown on cell viability and migration of RL-95-2 cells. a** Representative MTT proliferation assays in RL-95-2 *SF3B1* knockdown and control cells. **b** Representative MTT proliferation assays in RL-95-2 cells treated with Vehicle, 5 nM and 10 nM PLAD-B at indicated time points. **c** *SF3B1* transcript levels in RL-95-2 cells transfected with control siRNA or *SF3B1* siRNA. **d** Wound healing assay of RL-95-2 cells treated with control siRNA or *SF3B1* siRNA at 80-90% confluence **e** Expression analysis of proteins associated with cell migration. **f** Comparison of % cell viability and migration potential of different *SF3B1* siRNA depleted or PLAD treated EC cell lines at 72h.

**Fig. S3:** **Validation of DEGs identified by RNA sequencing** **in EC cells.** qRT-PCR analysis to validate selected targets from RNA-sequencing in *SF3B1* depleted Ishikawa and AN3CA cells. 18S was used for normalization. Data are presented as mean ± SEM. **P*<0.05, ***P*<0.01, ****P*<0.001, *****P*<0.0001.

**Fig. S4**: DEGs altered with *SF3B1* knockdown that enriched in Ras signaling pathway.

**Supplementary table 1:** List of DEGs identified by RNA sequencing with *SF3B1* knockdown in Ishikawa cells.

**Supplementary table 2:** Gene Ontology analysis of DEGs altered with *SF3B1* knockdown in Ishikawa cells.

**Supplementary table 3:** List of genes showing differential exon usage between *SF3B1* depleted Ishikawa and control cells, obtained from RNA sequencing data analysis using DEXSeq.

**Supplementary table 4:** A Gene Feature File (GFF) containing exon annotations for all exons summarized.

**Supplementary table 5:** List of primers and TaqMan probes used.

**Supplementary table 6:** List of antibodies used.

**References**

1 Kommagani, R. *et al.* The Promyelocytic Leukemia Zinc Finger Transcription Factor Is Critical for Human Endometrial Stromal Cell Decidualization. *PLoS Genet* **12**, e1005937, doi:10.1371/journal.pgen.1005937 (2016).

2 Pearce, L. R. *et al.* KSR2 mutations are associated with obesity, insulin resistance, and impaired cellular fuel oxidation. *Cell* **155**, 765-777, doi:10.1016/j.cell.2013.09.058 (2013).

3 Huber, W. *et al.* Orchestrating high-throughput genomic analysis with Bioconductor. *Nat Methods* **12**, 115-121, doi:10.1038/nmeth.3252 (2015).

4 Anders, S., Reyes, A. & Huber, W. Detecting differential usage of exons from RNA-seq data. *Genome Res* **22**, 2008-2017, doi:10.1101/gr.133744.111 (2012).

5 Cunningham, F. *et al.* Ensembl 2019. *Nucleic Acids Res* **47**, D745-D751, doi:10.1093/nar/gky1113 (2019).

6 Anders, S., Pyl, P. T. & Huber, W. HTSeq--a Python framework to work with high-throughput sequencing data. *Bioinformatics* **31**, 166-169, doi:10.1093/bioinformatics/btu638 (2015).
