# Supplementary figures and images for: Splicing factor SF3B1 promotes endometrial cancer progression via regulating KSR2 RNA maturation
Source: Cell Death Dis. 2020 Oct 10;11(10):842. doi: 10.1038/s41419-020-03055-y (PMC7548007; doi:10.1038/s41419-020-03055-y)

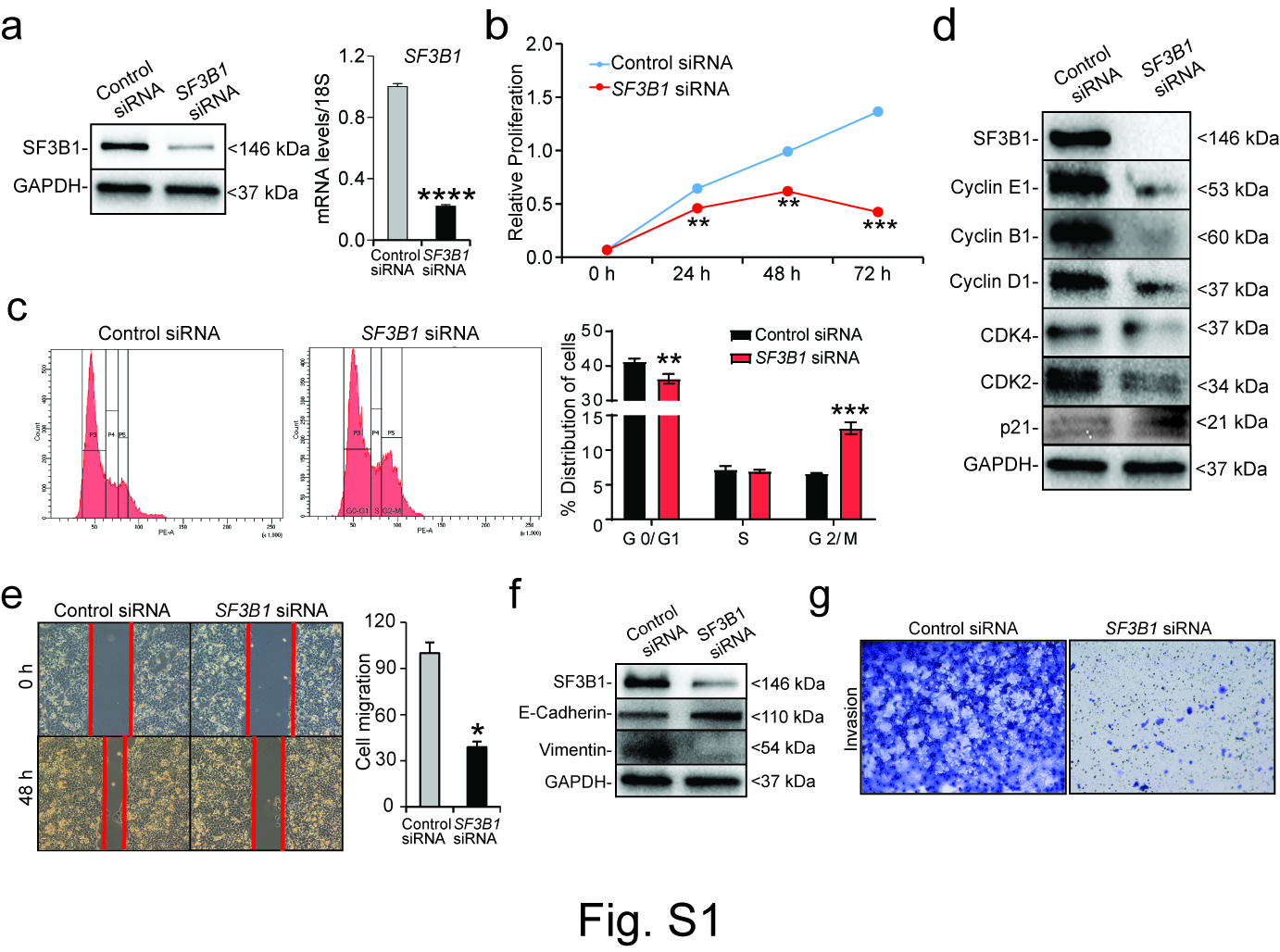

Supplement: Supplementary file 2 — Fig. S1 [file 41419_2020_3055_MOESM2_ESM.tif]

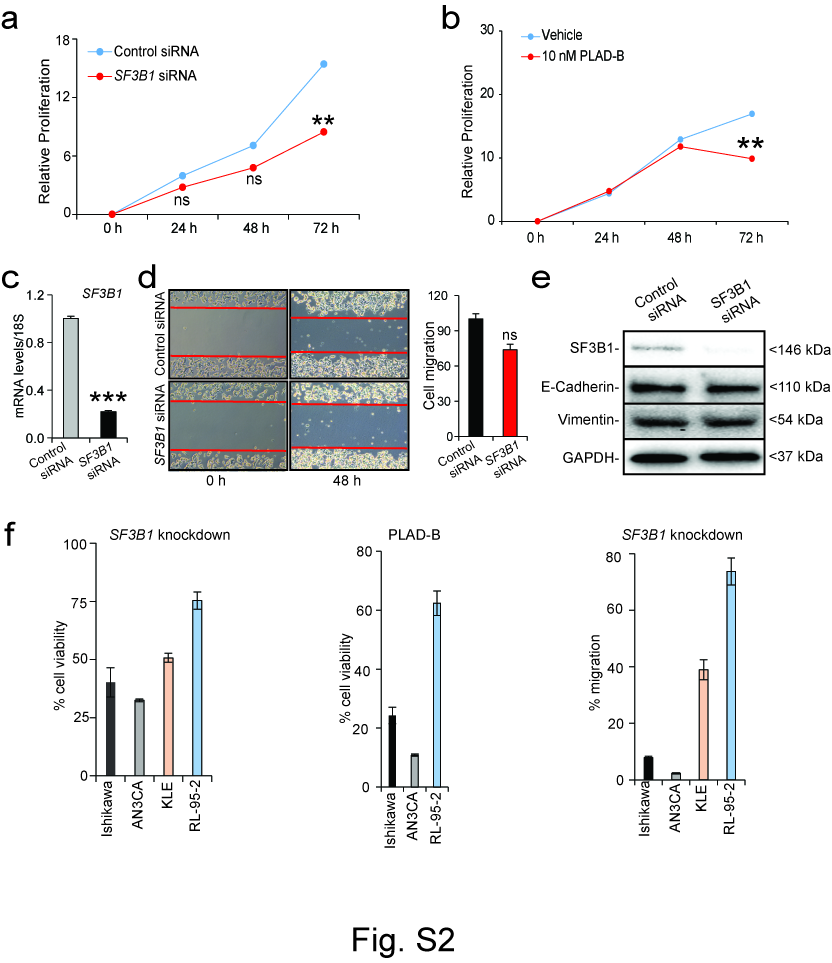

Supplement: Supplementary file 3 — Fig. S2 [file 41419_2020_3055_MOESM3_ESM.tif]

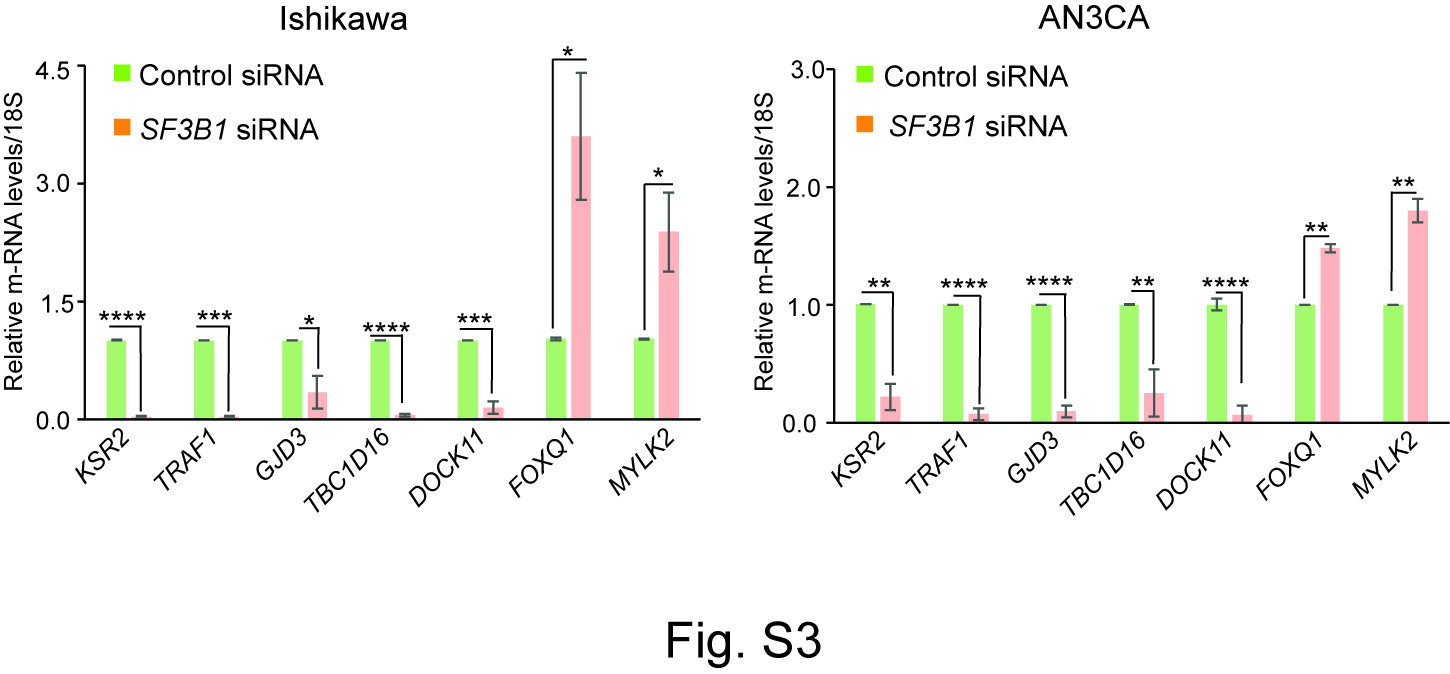

Supplement: Supplementary file 4 — Fig. S3 [file 41419_2020_3055_MOESM4_ESM.tif]

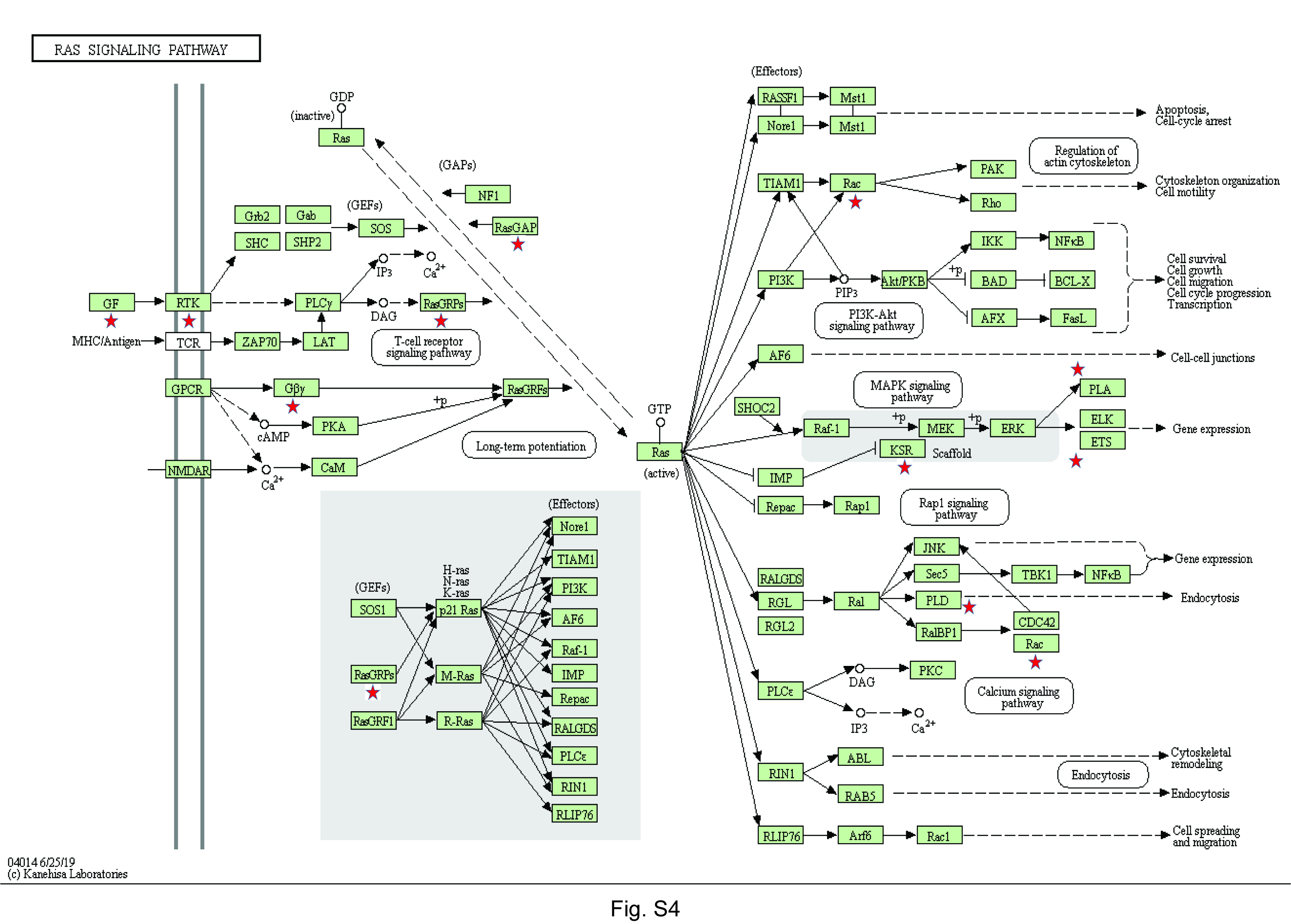

Supplement: Supplementary file 5 — Fig. S4 [file 41419_2020_3055_MOESM5_ESM.tif]
