# Supplementary material for: Splicing factor SF3B1 promotes endometrial cancer progression via regulating KSR2 RNA maturation
Source: Cell Death Dis. 2020 Oct 10;11(10):842. doi: 10.1038/s41419-020-03055-y (PMC7548007; doi:10.1038/s41419-020-03055-y)
Supplement: Supplementary file 6 — Supplementary Table 1 [file 41419_2020_3055_MOESM6_ESM.pdf]

Supplementary Table 1

Feature_ID	entrezgene	external_gene_name	gene_biotype	external_gene_source	transcript_count	description	LINEAR FC
ENSG00000231062	NA	AC103563.2	antisense	Clone-based (Ensembl) gene	1	putative novel transcript	-32.892359
ENSG00000255369	NA	AP000920.1	processed_pseudogene	Clone-based (Ensembl) gene	1	SRY (sex determining region Y)-box 15 (SOX15) pseudogene	-13.089984
ENSG00000102104	6247	RS1	protein_coding	HGNC Symbol	2	retinoschisin 1 [Source:HGNC Symbol;Acc:HGNC:10457]	-11.367566
ENSG00000276191	1394	CRHR1	protein_coding	HGNC Symbol	14	corticotropin releasing hormone receptor 1 [Source:HGNC Symbol;Acc:HGNC:2357]	-10.894619
ENSG00000276191	1.05E+08	CRHR1	protein_coding	HGNC Symbol	14	corticotropin releasing hormone receptor 1 [Source:HGNC Symbol;Acc:HGNC:2357]	-10.894619
ENSG00000230676	NA	AL353803.2	lincRNA	Clone-based (Ensembl) gene	2	novel transcript	-10.68088
ENSG00000181201	NA	HIST3H2BA	unitary_pseudogene	HGNC Symbol	1	histone cluster 3 H2B family member a (pseudogene) [Source:HGNC Symbol;Acc:HGNC:20515	-9.626344
ENSG00000226472	NA	AC008013.1	transcribed_unprocessed_pseudogene	Clone-based (Ensembl) gene	7	tetraspanin family pseudogene	-8.58415
ENSG00000186510	1187	CLCNKA	protein_coding	HGNC Symbol	7	chloride voltage-gated channel Ka [Source:HGNC Symbol;Acc:HGNC:2026]	-7.704644
ENSG00000120457	3762	KCNJ5	protein_coding	HGNC Symbol	3	potassium voltage-gated channel subfamily J member 5 [Source:HGNC Symbol;Acc:HGNC:626	-7.520674
ENSG00000005102	4222	MEOX1	protein_coding	HGNC Symbol	4	mesenchyme homeobox 1 [Source:HGNC Symbol;Acc:HGNC:7013]	-7.480276
ENSG00000089041	5027	P2RX7	protein_coding	HGNC Symbol	13	purinergic receptor P2X 7 [Source:HGNC Symbol;Acc:HGNC:8537]	-7.310552
ENSG00000253981	NA	ALG1L13P	transcribed_unprocessed_pseudogene	HGNC Symbol	4	asparagine-linked glycosylation 1-like 13, pseudogene [Source:HGNC Symbol;Acc:HGNC:4438	-7.175287
ENSG00000230039	NA	AL450487.1	processed_pseudogene	Clone-based (Ensembl) gene	1	cyclin fold protein 1 (CFP1) pseudogene	-7.086752
ENSG00000175785	145270	PRIMA1	protein_coding	HGNC Symbol	4	proline rich membrane anchor 1 [Source:HGNC Symbol;Acc:HGNC:18319]	-6.98436
ENSG00000241322	374286	CDRT1	protein_coding	HGNC Symbol	8	CMT1A duplicated region transcript 1 [Source:HGNC Symbol;Acc:HGNC:14379]	-6.936144
ENSG00000182983	389114	ZNF662	protein_coding	HGNC Symbol	5	zinc finger protein 662 [Source:HGNC Symbol;Acc:HGNC:31930]	-6.875618
ENSG00000188176	342527	SMTNL2	protein_coding	HGNC Symbol	4	smoothelin like 2 [Source:HGNC Symbol;Acc:HGNC:24764]	-6.858949
ENSG00000164089	64850	ETNPPL	protein_coding	HGNC Symbol	10	ethanolamine-phosphate phospho-lyase [Source:HGNC Symbol;Acc:HGNC:14404]	-6.772603
ENSG00000157870	127281	PRXL2B	protein_coding	HGNC Symbol	17	peroxiredoxin like 2B [Source:HGNC Symbol;Acc:HGNC:28390]	-6.644542
ENSG00000228838	NA	AL355483.2	antisense	Clone-based (Ensembl) gene	1	novel transcript	-6.32317
ENSG00000256577	1.02E+08	AC007406.4	antisense	Clone-based (Ensembl) gene	1		-6.299188
ENSG00000116039	525	ATP6V1B1	protein_coding	HGNC Symbol	7	ATPase H+ transporting V1 subunit B1 [Source:HGNC Symbol;Acc:HGNC:853]	-6.214953
ENSG00000160191	5152	PDE9A	protein_coding	HGNC Symbol	28	phosphodiesterase 9A [Source:HGNC Symbol;Acc:HGNC:8795]	-6.162541
ENSG00000151704	3758	KCNJ1	protein_coding	HGNC Symbol	7	potassium voltage-gated channel subfamily J member 1 [Source:HGNC Symbol;Acc:HGNC:625	-6.136679
ENSG00000111181	6539	SLC6A12	protein_coding	HGNC Symbol	16	solute carrier family 6 member 12 [Source:HGNC Symbol;Acc:HGNC:11045]	-6.086785
ENSG00000174370	219833	C11orf45	protein_coding	HGNC Symbol	3	chromosome 11 open reading frame 45 [Source:HGNC Symbol;Acc:HGNC:28584]	-6.003616
ENSG00000272088	NA	AL512413.1	lincRNA	Clone-based (Ensembl) gene	1	novel transcript	-5.877994
ENSG00000104044	4948	OCA2	protein_coding	HGNC Symbol	4	OCA2 melanosomal transmembrane protein [Source:HGNC Symbol;Acc:HGNC:8101]	-5.83651
ENSG00000204161	170371	TMEM273	protein_coding	HGNC Symbol	8	transmembrane protein 273 [Source:HGNC Symbol;Acc:HGNC:27274]	-5.785471
ENSG00000232000	NA	CLCN3P1	transcribed_unprocessed_pseudogene	HGNC Symbol	2	chloride voltage-gated channel 3 pseudogene 1 [Source:HGNC Symbol;Acc:HGNC:49775]	-5.764124
ENSG00000259495	NA	AC016705.2	lincRNA	Clone-based (Ensembl) gene	2	novel transcript	-5.683051
ENSG00000105409	478	ATP1A3	protein_coding	HGNC Symbol	15	ATPase Na+/K+ transporting subunit alpha 3 [Source:HGNC Symbol;Acc:HGNC:801]	-5.678505
ENSG00000232093	NA	DCST1-AS1	antisense	HGNC Symbol	1	DCST1 antisense RNA 1 [Source:HGNC Symbol;Acc:HGNC:41147]	-5.663055
ENSG00000142619	51702	PADI3	protein_coding	HGNC Symbol	1	peptidyl arginine deiminase 3 [Source:HGNC Symbol;Acc:HGNC:18337]	-5.65819
ENSG00000225308	NA	ASS1P11	processed_pseudogene	HGNC Symbol	1	argininosuccinate synthetase 1 pseudogene 11 [Source:HGNC Symbol;Acc:HGNC:761]	-5.483629
ENSG00000215035	NA	FDPSP5	processed_pseudogene	HGNC Symbol	1	farnesyl diphosphate synthase pseudogene 5 [Source:HGNC Symbol;Acc:HGNC:3636]	-5.482655
ENSG00000260423	1.02E+08	LINC02367	lincRNA	HGNC Symbol	1	long intergenic non-protein coding RNA 2367 [Source:HGNC Symbol;Acc:HGNC:53290]	-5.480369
ENSG00000095627	56165	TDRD1	protein_coding	HGNC Symbol	3	tudor domain containing 1 [Source:HGNC Symbol;Acc:HGNC:11712]	-5.462465
ENSG00000189269	51233	DRICH1	protein_coding	HGNC Symbol	2	aspartate rich 1 [Source:HGNC Symbol;Acc:HGNC:28031]	-5.433965
ENSG00000140481	80125	CCDC33	protein_coding	HGNC Symbol	12	coiled-coil domain containing 33 [Source:HGNC Symbol;Acc:HGNC:26552]	-5.388765
ENSG00000259479	653381	SORD2P	transcribed_unprocessed_pseudogene	HGNC Symbol	4	sorbitol dehydrogenase 2, pseudogene [Source:HGNC Symbol;Acc:HGNC:49919]	-5.362181
ENSG00000084710	22979	EFR3B	protein_coding	HGNC Symbol	5	EFR3 homolog B [Source:HGNC Symbol;Acc:HGNC:29155]	-5.3477
ENSG00000158428	375307	CATIP	protein_coding	HGNC Symbol	5	ciliogenesis associated TTC17 interacting protein [Source:HGNC Symbol;Acc:HGNC:25062]	-5.322539
ENSG00000198570	343035	RD3	protein_coding	HGNC Symbol	2	retinal degeneration 3, GUCY2D regulator [Source:HGNC Symbol;Acc:HGNC:19689]	-5.319868
ENSG00000203499	1E+08	IQANK1	protein_coding	HGNC Symbol	5	IQ motif and ankyrin repeat containing 1 [Source:HGNC Symbol;Acc:HGNC:49576]	-5.236168
ENSG00000123453	1757	SARDH	protein_coding	HGNC Symbol	8	sarcosine dehydrogenase [Source:HGNC Symbol;Acc:HGNC:10536]	-5.198461
ENSG00000230535	NA	BASP1P1	processed_pseudogene	HGNC Symbol	1	brain abundant, membrane attached signal protein 1 pseudogene 1 [Source:HGNC Symbol;Ac	-5.189463
ENSG00000107551	83937	RASSF4	protein_coding	HGNC Symbol	12	Ras association domain family member 4 [Source:HGNC Symbol;Acc:HGNC:20793]	-5.187963
ENSG00000138615	8483	CILP	protein_coding	HGNC Symbol	1	cartilage intermediate layer protein [Source:HGNC Symbol;Acc:HGNC:1980]	-5.170271
ENSG00000181652	285973	ATG9B	protein_coding	HGNC Symbol	12	autophagy related 9B [Source:HGNC Symbol;Acc:HGNC:21899]	-5.139846
ENSG00000243244	11037	STON1	protein_coding	HGNC Symbol	5	stonin 1 [Source:HGNC Symbol;Acc:HGNC:17003]	-5.121792
ENSG00000277196	1.03E+08	AC007325.2	protein_coding	Clone-based (Ensembl) gene	2	proline dehydrogenase 1, mitochondrial [Source:NCBI gene;Acc:102724788]	-5.099417
ENSG00000242173	398	ARHGDIG	protein_coding	HGNC Symbol	7	Rho GDP dissociation inhibitor gamma [Source:HGNC Symbol;Acc:HGNC:680]	-5.072287
ENSG00000151224	4143	MAT1A	protein_coding	HGNC Symbol	4	methionine adenosyltransferase 1A [Source:HGNC Symbol;Acc:HGNC:6903]	-5.039512
ENSG00000240694	10687	PNMA2	protein_coding	HGNC Symbol	8	PNMA family member 2 [Source:HGNC Symbol;Acc:HGNC:9159]	-5.029021
ENSG00000154102	404550	C16orf74	protein_coding	HGNC Symbol	7	chromosome 16 open reading frame 74 [Source:HGNC Symbol;Acc:HGNC:23362]	-5.027651
ENSG00000011143	54903	MKS1	protein_coding	HGNC Symbol	13	Meckel syndrome, type 1 [Source:HGNC Symbol;Acc:HGNC:7121]	-4.945722
ENSG00000088002	6820	SULT2B1	protein_coding	HGNC Symbol	4	sulfotransferase family 2B member 1 [Source:HGNC Symbol;Acc:HGNC:11459]	-4.924153
ENSG00000186088	54103	GSAP	protein_coding	HGNC Symbol	13	gamma-secretase activating protein [Source:HGNC Symbol;Acc:HGNC:28042]	-4.91993
ENSG00000179066	NA	AC020907.1	lincRNA	Clone-based (Ensembl) gene	5	novel transcript	-4.903064
ENSG00000198796	115701	ALPK2	protein_coding	HGNC Symbol	6	alpha kinase 2 [Source:HGNC Symbol;Acc:HGNC:20565]	-4.828753
ENSG00000161905	246	ALOX15	protein_coding	HGNC Symbol	7	arachidonate 15-lipoxygenase [Source:HGNC Symbol;Acc:HGNC:433]	-4.759492

ENSG00000159618	221188	ADGRG5	protein_coding	HGNC Symbol	7	adhesion G protein-coupled receptor G5 [Source:HGNC Symbol;Acc:HGNC:19010]	-4.758727
ENSG00000162814	128153	SPATA17	protein_coding	HGNC Symbol	5	spermatogenesis associated 17 [Source:HGNC Symbol;Acc:HGNC:25184]	-4.744064
ENSG00000233850	NA	AC103563.7	antisense	Clone-based (Ensembl) gene	1	novel transcript	-4.717512
ENSG00000124507	29993	PACSIN1	protein_coding	HGNC Symbol	7	protein kinase C and casein kinase substrate in neurons 1 [Source:HGNC Symbol;Acc:HGNC:85	-4.674988
ENSG00000265763	118738	ZNF488	protein_coding	HGNC Symbol	2	zinc finger protein 488 [Source:HGNC Symbol;Acc:HGNC:23535]	-4.671771
ENSG00000171084	1E+08	FAM86JP	transcribed_unprocessed_pseudogene	HGNC Symbol	4	family with sequence similarity 86 member J, pseudogene [Source:HGNC Symbol;Acc:HGNC:4	-4.669938
ENSG00000135205	57639	CCDC146	protein_coding	HGNC Symbol	9	coiled-coil domain containing 146 [Source:HGNC Symbol;Acc:HGNC:29296]	-4.662451
ENSG00000088280	55616	ASAP3	protein_coding	HGNC Symbol	12	ArfGAP with SH3 domain, ankyrin repeat and PH domain 3 [Source:HGNC Symbol;Acc:HGNC:1	-4.646875
ENSG00000158296	64849	SLC13A3	protein_coding	HGNC Symbol	11	solute carrier family 13 member 3 [Source:HGNC Symbol;Acc:HGNC:14430]	-4.641846
ENSG00000139445	121643	FOXN4	protein_coding	HGNC Symbol	4	forkhead box N4 [Source:HGNC Symbol;Acc:HGNC:21399]	-4.624472
ENSG00000149292	54970	TTC12	protein_coding	HGNC Symbol	22	tetratricopeptide repeat domain 12 [Source:HGNC Symbol;Acc:HGNC:23700]	-4.609047
ENSG00000145476	285440	CYP4V2	protein_coding	HGNC Symbol	4	cytochrome P450 family 4 subfamily V member 2 [Source:HGNC Symbol;Acc:HGNC:23198]	-4.602587
ENSG00000159733	57732	ZFYVE28	protein_coding	HGNC Symbol	11	zinc finger FYVE-type containing 28 [Source:HGNC Symbol;Acc:HGNC:29334]	-4.57289
ENSG00000131650	79412	KREMEN2	protein_coding	HGNC Symbol	6	kringle containing transmembrane protein 2 [Source:HGNC Symbol;Acc:HGNC:18797]	-4.546557
ENSG00000112562	64094	SMOC2	protein_coding	HGNC Symbol	6	SPARC related modular calcium binding 2 [Source:HGNC Symbol;Acc:HGNC:20323]	-4.538904
ENSG00000180432	1582	CYP8B1	protein_coding	HGNC Symbol	2	cytochrome P450 family 8 subfamily B member 1 [Source:HGNC Symbol;Acc:HGNC:2653]	-4.496239
ENSG00000008735	23542	MAPK8IP2	protein_coding	HGNC Symbol	2	mitogen-activated protein kinase 8 interacting protein 2 [Source:HGNC Symbol;Acc:HGNC:68	-4.486531
ENSG00000168209	54541	DDIT4	protein_coding	HGNC Symbol	3	DNA damage inducible transcript 4 [Source:HGNC Symbol;Acc:HGNC:24944]	-4.476106
ENSG00000119714	8111	GPR68	protein_coding	HGNC Symbol	4	G protein-coupled receptor 68 [Source:HGNC Symbol;Acc:HGNC:4519]	-4.460494
ENSG00000237886	NA	NALT1	antisense	HGNC Symbol	2	NOTCH1 associated lncRNA in T cell acute lymphoblastic leukemia 1 [Source:HGNC Symbol;Ac	-4.459222
ENSG00000223829	NA	AC004870.2	processed_transcript	Clone-based (Ensembl) gene	3		-4.455987
ENSG00000167291	125058	TBC1D16	protein_coding	HGNC Symbol	9	TBC1 domain family member 16 [Source:HGNC Symbol;Acc:HGNC:28356]	-4.429072
ENSG00000018625	477	ATP1A2	protein_coding	HGNC Symbol	8	ATPase Na+/K+ transporting subunit alpha 2 [Source:HGNC Symbol;Acc:HGNC:800]	-4.409332
ENSG00000160172	645332	FAM86C2P	transcribed_unprocessed_pseudogene	HGNC Symbol	5	family with sequence similarity 86 member C2, pseudogene [Source:HGNC Symbol;Acc:HGNC	-4.392312
ENSG00000157601	4599	MX1	protein_coding	HGNC Symbol	18	MX dynamin like GTPase 1 [Source:HGNC Symbol;Acc:HGNC:7532]	-4.378004
ENSG00000249846	NA	LINC02021	lincRNA	HGNC Symbol	3	long intergenic non-protein coding RNA 2021 [Source:HGNC Symbol;Acc:HGNC:52856]	-4.371178
ENSG00000112494	54346	UNC93A	protein_coding	HGNC Symbol	5	unc-93 homolog A [Source:HGNC Symbol;Acc:HGNC:12570]	-4.360669
ENSG00000133256	5158	PDE6B	protein_coding	HGNC Symbol	12	phosphodiesterase 6B [Source:HGNC Symbol;Acc:HGNC:8786]	-4.355698
ENSG00000151789	79750	ZNF385D	protein_coding	HGNC Symbol	11	zinc finger protein 385D [Source:HGNC Symbol;Acc:HGNC:26191]	-4.344791
ENSG00000189337	23254	KAZN	protein_coding	HGNC Symbol	9	kazrin, periplakin interacting protein [Source:HGNC Symbol;Acc:HGNC:29173]	-4.309811
ENSG00000128655	50940	PDE11A	protein_coding	HGNC Symbol	11	phosphodiesterase 11A [Source:HGNC Symbol;Acc:HGNC:8773]	-4.304226
ENSG00000111796	3820	KLRB1	protein_coding	HGNC Symbol	1	killer cell lectin like receptor B1 [Source:HGNC Symbol;Acc:HGNC:6373]	-4.303085
ENSG00000167741	124975	GGT6	protein_coding	HGNC Symbol	6	gamma-glutamyltransferase 6 [Source:HGNC Symbol;Acc:HGNC:26891]	-4.301309
ENSG00000105289	27134	TJP3	protein_coding	HGNC Symbol	9	tight junction protein 3 [Source:HGNC Symbol;Acc:HGNC:11829]	-4.299968
ENSG00000146966	27147	DENND2A	protein_coding	HGNC Symbol	10	DENN domain containing 2A [Source:HGNC Symbol;Acc:HGNC:22212]	-4.279496
ENSG00000227942	NA	FRMD8P1	processed_pseudogene	HGNC Symbol	1	FERM domain containing 8 pseudogene 1 [Source:HGNC Symbol;Acc:HGNC:24690]	-4.278373
ENSG00000169660	284004	HEXD	protein_coding	HGNC Symbol	17	hexosaminidase D [Source:HGNC Symbol;Acc:HGNC:26307]	-4.272022
ENSG00000158014	7780	SLC30A2	protein_coding	HGNC Symbol	3	solute carrier family 30 member 2 [Source:HGNC Symbol;Acc:HGNC:11013]	-4.264005
ENSG00000217289	NA	AC079776.1	processed_pseudogene	Clone-based (Ensembl) gene	1	single stranded DNA binding protein 3 (SSBP3) pseudogene	-4.245099
ENSG00000103089	79152	FA2H	protein_coding	HGNC Symbol	5	fatty acid 2-hydroxylase [Source:HGNC Symbol;Acc:HGNC:21197]	-4.243403
ENSG00000125864	631	BFSP1	protein_coding	HGNC Symbol	5	beaded filament structural protein 1 [Source:HGNC Symbol;Acc:HGNC:1040]	-4.230368
ENSG00000140986	6123	RPL3L	protein_coding	HGNC Symbol	3	ribosomal protein L3 like [Source:HGNC Symbol;Acc:HGNC:10351]	-4.22742
ENSG00000088543	51161	C3orf18	protein_coding	HGNC Symbol	11	chromosome 3 open reading frame 18 [Source:HGNC Symbol;Acc:HGNC:24837]	-4.224926
ENSG00000056558	7185	TRAF1	protein_coding	HGNC Symbol	3	TNF receptor associated factor 1 [Source:HGNC Symbol;Acc:HGNC:12031]	-4.223212
ENSG00000166436	9866	TRIM66	protein_coding	HGNC Symbol	10	tripartite motif containing 66 [Source:HGNC Symbol;Acc:HGNC:29005]	-4.221241
ENSG00000224383	92340	PRR29	protein_coding	HGNC Symbol	10	proline rich 29 [Source:HGNC Symbol;Acc:HGNC:25673]	-4.217381
ENSG00000173295	286042	FAM86B3P	transcribed_unprocessed_pseudogene	HGNC Symbol	8	family with sequence similarity 86 member B3, pseudogene [Source:HGNC Symbol;Acc:HGNC	-4.203346
ENSG00000182809	1397	CRIP2	protein_coding	HGNC Symbol	11	cysteine rich protein 2 [Source:HGNC Symbol;Acc:HGNC:2361]	-4.195573
ENSG00000105639	3718	JAK3	protein_coding	HGNC Symbol	7	Janus kinase 3 [Source:HGNC Symbol;Acc:HGNC:6193]	-4.179444
ENSG00000116014	84634	KISS1R	protein_coding	HGNC Symbol	3	KISS1 receptor [Source:HGNC Symbol;Acc:HGNC:4510]	-4.174866
ENSG00000258534	NA	AL132712.1	lincRNA	Clone-based (Ensembl) gene	1	novel transcript	-4.163093
ENSG00000147041	94122	SYTL5	protein_coding	HGNC Symbol	2	synaptotagmin like 5 [Source:HGNC Symbol;Acc:HGNC:15589]	-4.150881
ENSG00000231868	NA	AL031848.1	antisense	Clone-based (Ensembl) gene	1	novel transcript, antisense to ESPN	-4.147746
ENSG00000170382	10446	LRRN2	protein_coding	HGNC Symbol	4	leucine rich repeat neuronal 2 [Source:HGNC Symbol;Acc:HGNC:16914]	-4.132347
ENSG00000230490	NA	AL139383.1	lincRNA	Clone-based (Ensembl) gene	3	novel transcript	-4.130352
ENSG00000183044	18	ABAT	protein_coding	HGNC Symbol	16	4-aminobutyrate aminotransferase [Source:HGNC Symbol;Acc:HGNC:23]	-4.120839
ENSG00000126217	23263	MCF2L	protein_coding	HGNC Symbol	32	MCF.2 cell line derived transforming sequence like [Source:HGNC Symbol;Acc:HGNC:14576]	-4.11147
ENSG00000187621	NA	TCL6	processed_transcript	HGNC Symbol	13	T cell leukemia/lymphoma 6 [Source:HGNC Symbol;Acc:HGNC:13463]	-4.108417
ENSG00000002079	NA	MYH16	transcribed_unitary_pseudogene	HGNC Symbol	5	myosin heavy chain 16 pseudogene [Source:HGNC Symbol;Acc:HGNC:31038]	-4.105628
ENSG00000262619	NA	LINC00621	lincRNA	HGNC Symbol	1	long intergenic non-protein coding RNA 621 [Source:HGNC Symbol;Acc:HGNC:44227]	-4.102378
ENSG00000241935	112817	HOGA1	protein_coding	HGNC Symbol	4	4-hydroxy-2-oxoglutarate aldolase 1 [Source:HGNC Symbol;Acc:HGNC:25155]	-4.099928
ENSG00000212712	NA	AP002414.1	processed_pseudogene	Clone-based (Ensembl) gene	1	kinesin family member 1C (KIF1C) pseudogene	-4.097654
ENSG00000205791	503693	LOH12CR2	lincRNA	HGNC Symbol	1	loss of heterozygosity, 12, chromosomal region 2 [Source:HGNC Symbol;Acc:HGNC:26524]	-4.091556
ENSG00000242282	NA	AC108488.1	lincRNA	Clone-based (Ensembl) gene	5	novel transcript	-4.082524
ENSG00000180902	728294	D2HGDH	protein_coding	HGNC Symbol	16	D-2-hydroxyglutarate dehydrogenase [Source:HGNC Symbol;Acc:HGNC:28358]	-4.068024

ENSG00000244723	NA	ASLP1	unprocessed_pseudogene	HGNC Symbol	1	argininosuccinate lyase pseudogene 1 [Source:HGNC Symbol;Acc:HGNC:747]	-4.06249
ENSG00000081277	5317	PKP1	protein_coding	HGNC Symbol	5	plakophilin 1 [Source:HGNC Symbol;Acc:HGNC:9023]	-4.046875
ENSG00000267128	NA	RNF157-AS1	antisense	HGNC Symbol	5	RNF157 antisense RNA 1 [Source:HGNC Symbol;Acc:HGNC:44127]	-4.039296
ENSG00000198720	124930	ANKRD13B	protein_coding	HGNC Symbol	9	ankyrin repeat domain 13B [Source:HGNC Symbol;Acc:HGNC:26363]	-4.038926
ENSG00000135374	2001	ELF5	protein_coding	HGNC Symbol	6	E74 like ETS transcription factor 5 [Source:HGNC Symbol;Acc:HGNC:3320]	-4.035737
ENSG00000172350	64137	ABCG4	protein_coding	HGNC Symbol	6	ATP binding cassette subfamily G member 4 [Source:HGNC Symbol;Acc:HGNC:13884]	-4.024293
ENSG00000074181	4854	NOTCH3	protein_coding	HGNC Symbol	6	notch 3 [Source:HGNC Symbol;Acc:HGNC:7883]	-4.01778
ENSG00000157851	56896	DPYSL5	protein_coding	HGNC Symbol	7	dihydropyrimidinase like 5 [Source:HGNC Symbol;Acc:HGNC:20637]	-4.015509
ENSG00000126500	23769	FLRT1	protein_coding	HGNC Symbol	1	fibronectin leucine rich transmembrane protein 1 [Source:HGNC Symbol;Acc:HGNC:3760]	-4.015134
ENSG00000188051	1E+08	TMEM221	protein_coding	HGNC Symbol	2	transmembrane protein 221 [Source:HGNC Symbol;Acc:HGNC:21943]	-4.001952
ENSG00000187994	126432	RINL	protein_coding	HGNC Symbol	10	Ras and Rab interactor like [Source:HGNC Symbol;Acc:HGNC:24795]	-3.999779
ENSG00000167183	79170	PRR15L	protein_coding	HGNC Symbol	1	proline rich 15 like [Source:HGNC Symbol;Acc:HGNC:28149]	-3.997437
ENSG00000142102	80162	PGGHG	protein_coding	HGNC Symbol	8	protein-glucosylgalactosylhydroxylysine glucosidase [Source:HGNC Symbol;Acc:HGNC:26210]	-3.9841
ENSG00000265511	NA	AC020558.2	antisense	Clone-based (Ensembl) gene	1	novel transcript	-3.978001
ENSG00000125354	23157	6-Sep	protein_coding	HGNC Symbol	9	septin 6 [Source:HGNC Symbol;Acc:HGNC:15848]	-3.975639
ENSG00000186470	11118	BTN3A2	protein_coding	HGNC Symbol	19	butyrophilin subfamily 3 member A2 [Source:HGNC Symbol;Acc:HGNC:1139]	-3.971625
ENSG00000164049	285231	FBXW12	protein_coding	HGNC Symbol	6	F-box and WD repeat domain containing 12 [Source:HGNC Symbol;Acc:HGNC:20729]	-3.969759
ENSG00000225285	NA	LINC01770	lincRNA	HGNC Symbol	4	long intergenic non-protein coding RNA 1770 [Source:HGNC Symbol;Acc:HGNC:52560]	-3.966375
ENSG00000068078	2261	FGFR3	protein_coding	HGNC Symbol	11	fibroblast growth factor receptor 3 [Source:HGNC Symbol;Acc:HGNC:3690]	-3.9648
ENSG00000250479	400916	CHCHD10	protein_coding	HGNC Symbol	5	coiled-coil-helix-coiled-coil-helix domain containing 10 [Source:HGNC Symbol;Acc:HGNC:1555	-3.963141
ENSG00000144648	1238	ACKR2	protein_coding	HGNC Symbol	11	atypical chemokine receptor 2 [Source:HGNC Symbol;Acc:HGNC:1565]	-3.947467
ENSG00000197153	8356	HIST1H3J	protein_coding	HGNC Symbol	2	histone cluster 1 H3 family member j [Source:HGNC Symbol;Acc:HGNC:4774]	-3.946059
ENSG00000157193	7804	LRP8	protein_coding	HGNC Symbol	13	LDL receptor related protein 8 [Source:HGNC Symbol;Acc:HGNC:6700]	-3.932661
ENSG00000065057	4913	NTHL1	protein_coding	HGNC Symbol	10	nth like DNA glycosylase 1 [Source:HGNC Symbol;Acc:HGNC:8028]	-3.927756
ENSG00000171227	140738	TMEM37	protein_coding	HGNC Symbol	4	transmembrane protein 37 [Source:HGNC Symbol;Acc:HGNC:18216]	-3.923916
ENSG00000108813	1748	DLX4	protein_coding	HGNC Symbol	6	distal-less homeobox 4 [Source:HGNC Symbol;Acc:HGNC:2917]	-3.919024
ENSG00000252692	NA	RF00271	snoRNA	RFAM	1		-3.918484
ENSG00000069812	54626	HES2	protein_coding	HGNC Symbol	6	hes family bHLH transcription factor 2 [Source:HGNC Symbol;Acc:HGNC:16005]	-3.907064
ENSG00000135899	3431	SP110	protein_coding	HGNC Symbol	15	SP110 nuclear body protein [Source:HGNC Symbol;Acc:HGNC:5401]	-3.898345
ENSG00000143365	6097	RORC	protein_coding	HGNC Symbol	4	RAR related orphan receptor C [Source:HGNC Symbol;Acc:HGNC:10260]	-3.892972
ENSG00000226355	NA	AL353803.1	lincRNA	Clone-based (Ensembl) gene	1	novel transcript	-3.889205
ENSG00000107317	5730	PTGDS	protein_coding	HGNC Symbol	10	prostaglandin D2 synthase [Source:HGNC Symbol;Acc:HGNC:9592]	-3.887899
ENSG00000178814	26873	OPLAH	protein_coding	HGNC Symbol	4	5-oxoprolinase, ATP-hydrolysing [Source:HGNC Symbol;Acc:HGNC:8149]	-3.887495
ENSG00000149782	5331	PLCB3	protein_coding	HGNC Symbol	4	phospholipase C beta 3 [Source:HGNC Symbol;Acc:HGNC:9056]	-3.887277
ENSG00000237686	1.02E+08	AL109615.3	antisense	Clone-based (Ensembl) gene	2		-3.87399
ENSG00000176438	161176	SYNE3	protein_coding	HGNC Symbol	5	spectrin repeat containing nuclear envelope family member 3 [Source:HGNC Symbol;Acc:HGN	-3.867403
ENSG00000196440	1E+08	ARMCX4	protein_coding	HGNC Symbol	10	armadillo repeat containing X-linked 4 [Source:HGNC Symbol;Acc:HGNC:28615]	-3.859024
ENSG00000009950	51085	MLXIPL	protein_coding	HGNC Symbol	11	MLX interacting protein like [Source:HGNC Symbol;Acc:HGNC:12744]	-3.858779
ENSG00000135144	1840	DTX1	protein_coding	HGNC Symbol	5	deltex E3 ubiquitin ligase 1 [Source:HGNC Symbol;Acc:HGNC:3060]	-3.853164
ENSG00000257534	NA	AC023794.4	lincRNA	Clone-based (Ensembl) gene	1	novel transcript	-3.850358
ENSG00000117016	9783	RIMS3	protein_coding	HGNC Symbol	2	regulating synaptic membrane exocytosis 3 [Source:HGNC Symbol;Acc:HGNC:21292]	-3.847992
ENSG00000119715	2103	ESRRB	protein_coding	HGNC Symbol	8	estrogen related receptor beta [Source:HGNC Symbol;Acc:HGNC:3473]	-3.847862
ENSG00000261456	347688	TUBB8	protein_coding	HGNC Symbol	7	tubulin beta 8 class VIII [Source:HGNC Symbol;Acc:HGNC:20773]	-3.836764
ENSG00000272142	NA	AL359643.3	lincRNA	Clone-based (Ensembl) gene	3		-3.828153
ENSG00000105662	23373	CRTC1	protein_coding	HGNC Symbol	4	CREB regulated transcription coactivator 1 [Source:HGNC Symbol;Acc:HGNC:16062]	-3.816395
ENSG00000276600	338382	RAB7B	protein_coding	HGNC Symbol	5	RAB7B, member RAS oncogene family [Source:HGNC Symbol;Acc:HGNC:30513]	-3.815528
ENSG00000228903	NA	RASA4CP	transcribed_unprocessed_pseudogene	HGNC Symbol	3	RAS p21 protein activator 4C, pseudogene [Source:HGNC Symbol;Acc:HGNC:44185]	-3.808687
ENSG00000198759	25975	EGFL6	protein_coding	HGNC Symbol	3	EGF like domain multiple 6 [Source:HGNC Symbol;Acc:HGNC:3235]	-3.806645
ENSG00000275851	4302	MLLT6	protein_coding	HGNC Symbol	11	MLLT6, PHD finger containing [Source:HGNC Symbol;Acc:HGNC:7138]	-3.803378
ENSG00000188869	342125	TMC3	protein_coding	HGNC Symbol	3	transmembrane channel like 3 [Source:HGNC Symbol;Acc:HGNC:22995]	-3.802723
ENSG00000184363	11187	PKP3	protein_coding	HGNC Symbol	11	plakophilin 3 [Source:HGNC Symbol;Acc:HGNC:9025]	-3.800191
ENSG00000178537	788	SLC25A20	protein_coding	HGNC Symbol	4	solute carrier family 25 member 20 [Source:HGNC Symbol;Acc:HGNC:1421]	-3.800162
ENSG00000010361	80199	FUZ	protein_coding	HGNC Symbol	18	fuzzy planar cell polarity protein [Source:HGNC Symbol;Acc:HGNC:26219]	-3.796092
ENSG00000262681	NA	AC005722.2	lincRNA	Clone-based (Ensembl) gene	1	novel transcript	-3.795696
ENSG00000146090	255426	RASGEF1C	protein_coding	HGNC Symbol	7	RasGEF domain family member 1C [Source:HGNC Symbol;Acc:HGNC:27400]	-3.795171
ENSG00000241288	1.02E+08	LINC02614	processed_transcript	HGNC Symbol	22	long intergenic non-protein coding RNA 2614 [Source:HGNC Symbol;Acc:HGNC:54072]	-3.791491
ENSG00000167136	2021	ENDOG	protein_coding	HGNC Symbol	1	endonuclease G [Source:HGNC Symbol;Acc:HGNC:3346]	-3.789274
ENSG00000130822	139728	PNCK	protein_coding	HGNC Symbol	32	pregnancy up-regulated nonubiquitous CaM kinase [Source:HGNC Symbol;Acc:HGNC:13415]	-3.787417
ENSG00000242950	30816	ERVW-1	protein_coding	HGNC Symbol	5	endogenous retrovirus group W member 1, envelope [Source:HGNC Symbol;Acc:HGNC:13525	-3.786818
ENSG00000152582	79925	SPEF2	protein_coding	HGNC Symbol	16	sperm flagellar 2 [Source:HGNC Symbol;Acc:HGNC:26293]	-3.784715
ENSG00000197496	81031	SLC2A10	protein_coding	HGNC Symbol	3	solute carrier family 2 member 10 [Source:HGNC Symbol;Acc:HGNC:13444]	-3.784395
ENSG00000135502	65012	SLC26A10	protein_coding	HGNC Symbol	10	solute carrier family 26 member 10 [Source:HGNC Symbol;Acc:HGNC:14470]	-3.772858
ENSG00000188158	4810	NHS	protein_coding	HGNC Symbol	6	NHS actin remodeling regulator [Source:HGNC Symbol;Acc:HGNC:7820]	-3.767127
ENSG00000162542	255104	TMCO4	protein_coding	HGNC Symbol	8	transmembrane and coiled-coil domains 4 [Source:HGNC Symbol;Acc:HGNC:27393]	-3.766925
ENSG00000180938	137209	ZNF572	protein_coding	HGNC Symbol	1	zinc finger protein 572 [Source:HGNC Symbol;Acc:HGNC:26758]	-3.75468

ENSG00000105808	10156	RASA4	protein_coding	HGNC Symbol	17	RAS p21 protein activator 4 [Source:HGNC Symbol;Acc:HGNC:23181]	-3.754595
ENSG00000059588	6894	TARBP1	protein_coding	HGNC Symbol	9	TAR (HIV-1) RNA binding protein 1 [Source:HGNC Symbol;Acc:HGNC:11568]	-3.746719
ENSG00000156172	157657	C8orf37	protein_coding	HGNC Symbol	1	chromosome 8 open reading frame 37 [Source:HGNC Symbol;Acc:HGNC:27232]	-3.740618
ENSG00000259370	1.05E+08	AC103740.1	antisense	Clone-based (Ensembl) gene	4		-3.728926
ENSG00000160796	23218	NBEAL2	protein_coding	HGNC Symbol	11	neurobeachin like 2 [Source:HGNC Symbol;Acc:HGNC:31928]	-3.728012
ENSG00000184925	286256	LCN12	protein_coding	HGNC Symbol	15	lipocalin 12 [Source:HGNC Symbol;Acc:HGNC:28733]	-3.721664
ENSG00000197768	441476	STPG3	protein_coding	HGNC Symbol	7	sperm-tail PG-rich repeat containing 3 [Source:HGNC Symbol;Acc:HGNC:37285]	-3.711914
ENSG00000273523	1.02E+08	AL139082.1	antisense	Clone-based (Ensembl) gene	1		-3.705976
ENSG00000186687	90624	LYRM7	protein_coding	HGNC Symbol	4	LYR motif containing 7 [Source:HGNC Symbol;Acc:HGNC:28072]	-3.700469
ENSG00000277639	1.05E+08	AC007906.2	protein_coding	Clone-based (Ensembl) gene	2		-3.696106
ENSG00000240006	1.05E+08	LINC02004	lincRNA	HGNC Symbol	1	long intergenic non-protein coding RNA 2004 [Source:HGNC Symbol;Acc:HGNC:52838]	-3.691468
ENSG00000117266	5129	CDK18	protein_coding	HGNC Symbol	23	cyclin dependent kinase 18 [Source:HGNC Symbol;Acc:HGNC:8751]	-3.688927
ENSG00000166888	6778	STAT6	protein_coding	HGNC Symbol	25	signal transducer and activator of transcription 6 [Source:HGNC Symbol;Acc:HGNC:11368]	-3.686776
ENSG00000278635	NA	AC141557.2	unprocessed_pseudogene	Clone-based (Ensembl) gene	1	WAS protein family homolog 1 (WASH1) pseudogene	-3.686158
ENSG00000171435	283455	KSR2	protein_coding	HGNC Symbol	4	kinase suppressor of ras 2 [Source:HGNC Symbol;Acc:HGNC:18610]	-3.680666
ENSG00000247796	257396	AC008966.1	antisense	Clone-based (Ensembl) gene	3		-3.679142
ENSG00000159714	29800	ZDHHC1	protein_coding	HGNC Symbol	6	zinc finger DHHC-type containing 1 [Source:HGNC Symbol;Acc:HGNC:17916]	-3.672541
ENSG00000165983	9317	PTER	protein_coding	HGNC Symbol	5	phosphotriesterase related [Source:HGNC Symbol;Acc:HGNC:9590]	-3.671888
ENSG00000145002	653333	FAM86B2	protein_coding	HGNC Symbol	4	family with sequence similarity 86 member B2 [Source:HGNC Symbol;Acc:HGNC:32222]	-3.668878
ENSG00000167363	64122	FN3K	protein_coding	HGNC Symbol	5	fructosamine 3 kinase [Source:HGNC Symbol;Acc:HGNC:24822]	-3.667845
ENSG00000105255	79187	FSD1	protein_coding	HGNC Symbol	10	fibronectin type III and SPRY domain containing 1 [Source:HGNC Symbol;Acc:HGNC:13745]	-3.665093
ENSG00000256673	NA	AC141557.1	unprocessed_pseudogene	Clone-based (Ensembl) gene	1	ovostatin (OVOS) pseudogene	-3.664924
ENSG00000258472	NA	AC005726.1	protein_coding	Clone-based (Ensembl) gene	4	novel protein	-3.662548
ENSG00000255753	NA	AC009533.2	processed_pseudogene	Clone-based (Ensembl) gene	1	WAS protein family homolog pseudogene	-3.660819
ENSG00000269896	1E+08	AL513477.1	transcribed_processed_pseudogene	Clone-based (Ensembl) gene	2		-3.660145
ENSG00000275873	256329	LMNTD2	protein_coding	HGNC Symbol	5	lamin tail domain containing 2 [Source:HGNC Symbol;Acc:HGNC:28561]	-3.657983
ENSG00000266524	2662	GDF10	protein_coding	HGNC Symbol	1	growth differentiation factor 10 [Source:HGNC Symbol;Acc:HGNC:4215]	-3.655992
ENSG00000144792	285349	ZNF660	protein_coding	HGNC Symbol	4	zinc finger protein 660 [Source:HGNC Symbol;Acc:HGNC:26720]	-3.653577
ENSG00000129219	5338	PLD2	protein_coding	HGNC Symbol	15	phospholipase D2 [Source:HGNC Symbol;Acc:HGNC:9068]	-3.653146
ENSG00000108479	2584	GALK1	protein_coding	HGNC Symbol	9	galactokinase 1 [Source:HGNC Symbol;Acc:HGNC:4118]	-3.648074
ENSG00000231107	1.02E+08	LINC01508	lincRNA	HGNC Symbol	2	long intergenic non-protein coding RNA 1508 [Source:HGNC Symbol;Acc:HGNC:51190]	-3.642393
ENSG00000143390	5993	RFX5	protein_coding	HGNC Symbol	21	regulatory factor X5 [Source:HGNC Symbol;Acc:HGNC:9986]	-3.641853
ENSG00000225377	NA	NRSN2-AS1	antisense	HGNC Symbol	2	NRSN2 antisense RNA 1 [Source:HGNC Symbol;Acc:HGNC:51222]	-3.633958
ENSG00000180066	NA	C10orf91	lincRNA	HGNC Symbol	3	chromosome 10 open reading frame 91 (putative) [Source:HGNC Symbol;Acc:HGNC:27275]	-3.631645
ENSG00000154269	5169	ENPP3	protein_coding	HGNC Symbol	7	ectonucleotide pyrophosphatase/phosphodiesterase 3 [Source:HGNC Symbol;Acc:HGNC:335	-3.624115
ENSG00000232871	653677	SEC1P	transcribed_unitary_pseudogene	HGNC Symbol	4	secretory blood group 1, pseudogene [Source:HGNC Symbol;Acc:HGNC:44149]	-3.623531
ENSG00000189366	200810	ALG1L	protein_coding	HGNC Symbol	2	ALG1, chitobiosyldiphosphodolichol beta-mannosyltransferase like [Source:HGNC Symbol;Acc	-3.61912
ENSG00000154743	80746	TSEN2	protein_coding	HGNC Symbol	11	tRNA splicing endonuclease subunit 2 [Source:HGNC Symbol;Acc:HGNC:28422]	-3.617864
ENSG00000249348	1.01E+08	UGDH-AS1	antisense	HGNC Symbol	1	UGDH antisense RNA 1 [Source:HGNC Symbol;Acc:HGNC:40601]	-3.613758
ENSG00000005379	9256	TSPOAP1	protein_coding	HGNC Symbol	11	TSPO associated protein 1 [Source:HGNC Symbol;Acc:HGNC:16831]	-3.61096
ENSG00000251669	348926	FAM86EP	transcribed_unprocessed_pseudogene	HGNC Symbol	9	family with sequence similarity 86 member E, pseudogene [Source:HGNC Symbol;Acc:HGNC:2	-3.610241
ENSG00000181035	284439	SLC25A42	protein_coding	HGNC Symbol	6	solute carrier family 25 member 42 [Source:HGNC Symbol;Acc:HGNC:28380]	-3.604535
ENSG00000115107	55240	STEAP3	protein_coding	HGNC Symbol	4	STEAP3 metalloreductase [Source:HGNC Symbol;Acc:HGNC:24592]	-3.600276
ENSG00000267733	NA	AP005264.5	transcribed_processed_pseudogene	Clone-based (Ensembl) gene	5	solute carrier family 5 (low affinity glucose cotransporter), member 4 (SLC5A4) pseudogene	-3.600238
ENSG00000231789	1.02E+08	PIK3CD-AS2	antisense	HGNC Symbol	1	PIK3CD antisense RNA 2 [Source:HGNC Symbol;Acc:HGNC:51334]	-3.594076
ENSG00000110881	41	ASIC1	protein_coding	HGNC Symbol	9	acid sensing ion channel subunit 1 [Source:HGNC Symbol;Acc:HGNC:100]	-3.592624
ENSG00000100341	150379	PNPLA5	protein_coding	HGNC Symbol	5	patatin like phospholipase domain containing 5 [Source:HGNC Symbol;Acc:HGNC:24888]	-3.591899
ENSG00000177106	64787	EPS8L2	protein_coding	HGNC Symbol	32	EPS8 like 2 [Source:HGNC Symbol;Acc:HGNC:21296]	-3.590961
ENSG00000152926	1.1E+08	ZNF117	protein_coding	HGNC Symbol	4	zinc finger protein 117 [Source:HGNC Symbol;Acc:HGNC:12897]	-3.588114
ENSG00000152926	51351	ZNF117	protein_coding	HGNC Symbol	4	zinc finger protein 117 [Source:HGNC Symbol;Acc:HGNC:12897]	-3.588114
ENSG00000168907	255189	PLA2G4F	protein_coding	HGNC Symbol	7	phospholipase A2 group IVF [Source:HGNC Symbol;Acc:HGNC:27396]	-3.58483
ENSG00000255624	NA	AC073585.1	transcribed_processed_pseudogene	Clone-based (Ensembl) gene	2	chromosome 10 open reading frame 88 (C10orf88) pseudogene	-3.580598
ENSG00000183208	390637	GDPGP1	protein_coding	HGNC Symbol	5	GDP-D-glucose phosphorylase 1 [Source:HGNC Symbol;Acc:HGNC:34360]	-3.580496
ENSG00000251537	NA	AC005324.3	protein_coding	Clone-based (Ensembl) gene	1	novel tripartite motif-containing 16 (TRIM16) and CMT1A duplicated region transcript 1 (CDR	-3.575871
ENSG00000153029	3140	MR1	protein_coding	HGNC Symbol	9	major histocompatibility complex, class I-related [Source:HGNC Symbol;Acc:HGNC:4975]	-3.574641
ENSG00000128268	4248	MGAT3	protein_coding	HGNC Symbol	3	mannosyl (beta-1,4-)-glycoprotein beta-1,4-N-acetylglucosaminyltransferase [Source:HGNC Sy	-3.574062
ENSG00000224717	284577	AC098936.1	lincRNA	Clone-based (Ensembl) gene	1	novel transcript	-3.571026
ENSG00000175414	285598	ARL10	protein_coding	HGNC Symbol	4	ADP ribosylation factor like GTPase 10 [Source:HGNC Symbol;Acc:HGNC:22042]	-3.570897
ENSG00000166035	3990	LIPC	protein_coding	HGNC Symbol	7	lipase C, hepatic type [Source:HGNC Symbol;Acc:HGNC:6619]	-3.56819
ENSG00000171462	65989	DLK2	protein_coding	HGNC Symbol	4	delta like non-canonical Notch ligand 2 [Source:HGNC Symbol;Acc:HGNC:21113]	-3.561178
ENSG00000174600	1240	CMKLR1	protein_coding	HGNC Symbol	6	chemerin chemokine-like receptor 1 [Source:HGNC Symbol;Acc:HGNC:2121]	-3.55749
ENSG00000133216	2048	EPHB2	protein_coding	HGNC Symbol	7	EPH receptor B2 [Source:HGNC Symbol;Acc:HGNC:3393]	-3.555322
ENSG00000175643	116028	RMI2	protein_coding	HGNC Symbol	7	RecQ mediated genome instability 2 [Source:HGNC Symbol;Acc:HGNC:28349]	-3.554576
ENSG00000215271	57594	HOMEZ	protein_coding	HGNC Symbol	4	homeobox and leucine zipper encoding [Source:HGNC Symbol;Acc:HGNC:20164]	-3.545717
ENSG00000130653	375775	PNPLA7	protein_coding	HGNC Symbol	7	patatin like phospholipase domain containing 7 [Source:HGNC Symbol;Acc:HGNC:24768]	-3.540587

ENSG00000150873	130813	C2orf50	protein_coding	HGNC Symbol	2	chromosome 2 open reading frame 50 [Source:HGNC Symbol;Acc:HGNC:26324]	-3.532642
ENSG00000127399	65999	LRRC61	protein_coding	HGNC Symbol	5	leucine rich repeat containing 61 [Source:HGNC Symbol;Acc:HGNC:21704]	-3.531611
ENSG00000188747	10811	NOXA1	protein_coding	HGNC Symbol	2	NADPH oxidase activator 1 [Source:HGNC Symbol;Acc:HGNC:10668]	-3.524185
ENSG00000138030	3795	KHK	protein_coding	HGNC Symbol	6	ketohexokinase [Source:HGNC Symbol;Acc:HGNC:6315]	-3.522291
ENSG00000243479	645249	MNX1-AS1	lincRNA	HGNC Symbol	1	MNX1 antisense RNA 1 (head to head) [Source:HGNC Symbol;Acc:HGNC:48954]	-3.521631
ENSG00000226200	NA	SGMS1-AS1	antisense	HGNC Symbol	2	SGMS1 antisense RNA 1 [Source:HGNC Symbol;Acc:HGNC:49683]	-3.518164
ENSG00000266968	NA	AC023421.1	sense_intronic	Clone-based (Ensembl) gene	1	novel transcript	-3.504095
ENSG00000132024	54862	CC2D1A	protein_coding	HGNC Symbol	9	coiled-coil and C2 domain containing 1A [Source:HGNC Symbol;Acc:HGNC:30237]	-3.501975
ENSG00000273831	115399	LRRC56	protein_coding	HGNC Symbol	1	leucine rich repeat containing 56 [Source:HGNC Symbol;Acc:HGNC:25430]	-3.5016
ENSG00000131620	55107	ANO1	protein_coding	HGNC Symbol	10	anoctamin 1 [Source:HGNC Symbol;Acc:HGNC:21625]	-3.501557
ENSG00000117013	9132	KCNQ4	protein_coding	HGNC Symbol	5	potassium voltage-gated channel subfamily Q member 4 [Source:HGNC Symbol;Acc:HGNC:62	-3.489214
ENSG00000276612	NA	FP565260.2	protein_coding	Clone-based (Ensembl) gene	1	novel protein	-3.488892
ENSG00000267480	NA	AP001542.3	antisense	Clone-based (Ensembl) gene	1	novel transcript, antisense IMPA2	-3.484279
ENSG00000248712	283152	CCDC153	protein_coding	HGNC Symbol	4	coiled-coil domain containing 153 [Source:HGNC Symbol;Acc:HGNC:27446]	-3.48322
ENSG00000140263	6652	SORD	protein_coding	HGNC Symbol	7	sorbitol dehydrogenase [Source:HGNC Symbol;Acc:HGNC:11184]	-3.482274
ENSG00000161082	60680	CELF5	protein_coding	HGNC Symbol	8	CUGBP Elav-like family member 5 [Source:HGNC Symbol;Acc:HGNC:14058]	-3.481578
ENSG00000229847	196047	EMX2OS	antisense	HGNC Symbol	7	EMX2 opposite strand/antisense RNA [Source:HGNC Symbol;Acc:HGNC:18511]	-3.480476
ENSG00000278759	6904	TBCD	protein_coding	HGNC Symbol	19	tubulin folding cofactor D [Source:HGNC Symbol;Acc:HGNC:11581]	-3.478438
ENSG00000160200	875	CBS	protein_coding	HGNC Symbol	17	cystathionine-beta-synthase [Source:HGNC Symbol;Acc:HGNC:1550]	-3.477703
ENSG00000110057	81622	UNC93B1	protein_coding	HGNC Symbol	10	unc-93 homolog B1, TLR signaling regulator [Source:HGNC Symbol;Acc:HGNC:13481]	-3.476092
ENSG00000177732	6666	SOX12	protein_coding	HGNC Symbol	1	SRY-box 12 [Source:HGNC Symbol;Acc:HGNC:11198]	-3.465996
ENSG00000142449	84467	FBN3	protein_coding	HGNC Symbol	8	fibrillin 3 [Source:HGNC Symbol;Acc:HGNC:18794]	-3.464988
ENSG00000224713	NA	AC025165.1	antisense	Clone-based (Ensembl) gene	4	novel transcript, antisense to ARHGEF25	-3.46471
ENSG00000173641	27129	HSPB7	protein_coding	HGNC Symbol	7	heat shock protein family B (small) member 7 [Source:HGNC Symbol;Acc:HGNC:5249]	-3.464216
ENSG00000145882	78991	PCYOX1L	protein_coding	HGNC Symbol	7	prenylcysteine oxidase 1 like [Source:HGNC Symbol;Acc:HGNC:28477]	-3.462761
ENSG00000260417	NA	AC092127.1	lincRNA	Clone-based (Ensembl) gene	1	novel transcript	-3.462455
ENSG00000244479	NA	OR2A1-AS1	antisense	HGNC Symbol	10	OR2A1 antisense RNA 1 [Source:HGNC Symbol;Acc:HGNC:49168]	-3.46011
ENSG00000205593	414918	DENND6B	protein_coding	HGNC Symbol	5	DENN domain containing 6B [Source:HGNC Symbol;Acc:HGNC:32690]	-3.457765
ENSG00000158106	114822	RHPN1	protein_coding	HGNC Symbol	3	rhophilin Rho GTPase binding protein 1 [Source:HGNC Symbol;Acc:HGNC:19973]	-3.453524
ENSG00000267385	NA	AC011498.4	protein_coding	Clone-based (Ensembl) gene	1	novel transcript	-3.451355
ENSG00000106688	6505	SLC1A1	protein_coding	HGNC Symbol	3	solute carrier family 1 member 1 [Source:HGNC Symbol;Acc:HGNC:10939]	-3.446054
ENSG00000165240	538	ATP7A	protein_coding	HGNC Symbol	6	ATPase copper transporting alpha [Source:HGNC Symbol;Acc:HGNC:869]	-3.444103
ENSG00000270124	NA	AC092127.2	lincRNA	Clone-based (Ensembl) gene	1	novel transcript	-3.435398
ENSG00000260877	NA	AP005233.2	lincRNA	Clone-based (Ensembl) gene	1	novel transcript	-3.433984
ENSG00000214140	768206	PRCD	protein_coding	HGNC Symbol	13	photoreceptor disc component [Source:HGNC Symbol;Acc:HGNC:32528]	-3.433758
ENSG00000138075	64240	ABCG5	protein_coding	HGNC Symbol	5	ATP binding cassette subfamily G member 5 [Source:HGNC Symbol;Acc:HGNC:13886]	-3.432136
ENSG00000197106	388662	SLC6A17	protein_coding	HGNC Symbol	2	solute carrier family 6 member 17 [Source:HGNC Symbol;Acc:HGNC:31399]	-3.430664
ENSG00000128266	2781	GNAZ	protein_coding	HGNC Symbol	3	G protein subunit alpha z [Source:HGNC Symbol;Acc:HGNC:4395]	-3.428156
ENSG00000233184	NA	AC093157.1	antisense	Clone-based (Ensembl) gene	6		-3.427085
ENSG00000255121	NA	AP003392.4	lincRNA	Clone-based (Ensembl) gene	2	novel transcript	-3.427043
ENSG00000005206	56928	SPPL2B	protein_coding	HGNC Symbol	14	signal peptide peptidase like 2B [Source:HGNC Symbol;Acc:HGNC:30627]	-3.421047
ENSG00000166819	5346	PLIN1	protein_coding	HGNC Symbol	4	perilipin 1 [Source:HGNC Symbol;Acc:HGNC:9076]	-3.420354
ENSG00000140688	64755	C16orf58	protein_coding	HGNC Symbol	11	chromosome 16 open reading frame 58 [Source:HGNC Symbol;Acc:HGNC:25848]	-3.414622
ENSG00000148357	256158	HMCN2	protein_coding	HGNC Symbol	6	hemicentin 2 [Source:HGNC Symbol;Acc:HGNC:21293]	-3.410631
ENSG00000163395	91156	IGFN1	protein_coding	HGNC Symbol	6	immunoglobulin-like and fibronectin type III domain containing 1 [Source:HGNC Symbol;Acc:H	-3.408323
ENSG00000167646	352909	DNAAF3	protein_coding	HGNC Symbol	17	dynein axonemal assembly factor 3 [Source:HGNC Symbol;Acc:HGNC:30492]	-3.4074
ENSG00000107719	27143	PALD1	protein_coding	HGNC Symbol	1	phosphatase domain containing paladin 1 [Source:HGNC Symbol;Acc:HGNC:23530]	-3.40366
ENSG00000100346	8911	CACNA1I	protein_coding	HGNC Symbol	5	calcium voltage-gated channel subunit alpha1 I [Source:HGNC Symbol;Acc:HGNC:1396]	-3.400842
ENSG00000278372	80179	MYO19	protein_coding	HGNC Symbol	27	myosin XIX [Source:HGNC Symbol;Acc:HGNC:26234]	-3.398927
ENSG00000175662	146691	TOM1L2	protein_coding	HGNC Symbol	14	target of myb1 like 2 membrane trafficking protein [Source:HGNC Symbol;Acc:HGNC:11984]	-3.391142
ENSG00000163817	54716	SLC6A20	protein_coding	HGNC Symbol	7	solute carrier family 6 member 20 [Source:HGNC Symbol;Acc:HGNC:30927]	-3.388744
ENSG00000178115	728047	GOLGA8Q	protein_coding	HGNC Symbol	2	golgin A8 family member Q [Source:HGNC Symbol;Acc:HGNC:44408]	-3.388158
ENSG00000178115	727909	GOLGA8Q	protein_coding	HGNC Symbol	2	golgin A8 family member Q [Source:HGNC Symbol;Acc:HGNC:44408]	-3.388158
ENSG00000227121	NA	AC073174.1	lincRNA	Clone-based (Ensembl) gene	1	novel transcript	-3.388052
ENSG00000049239	9563	H6PD	protein_coding	HGNC Symbol	3	hexose-6-phosphate dehydrogenase/glucose 1-dehydrogenase [Source:HGNC Symbol;Acc:HG	-3.38476
ENSG00000065534	4638	MYLK	protein_coding	HGNC Symbol	21	myosin light chain kinase [Source:HGNC Symbol;Acc:HGNC:7590]	-3.379021
ENSG00000168077	51435	SCARA3	protein_coding	HGNC Symbol	2	scavenger receptor class A member 3 [Source:HGNC Symbol;Acc:HGNC:19000]	-3.378024
ENSG00000116151	79906	MORN1	protein_coding	HGNC Symbol	11	MORN repeat containing 1 [Source:HGNC Symbol;Acc:HGNC:25852]	-3.377179
ENSG00000256481	NA	AP006333.2	antisense	Clone-based (Ensembl) gene	1	novel transcript, antisense to MACROD1	-3.374678
ENSG00000104953	79816	TLE6	protein_coding	HGNC Symbol	10	transducin like enhancer of split 6 [Source:HGNC Symbol;Acc:HGNC:30788]	-3.373084
ENSG00000088899	9762	LZTS3	protein_coding	HGNC Symbol	4	leucine zipper tumor suppressor family member 3 [Source:HGNC Symbol;Acc:HGNC:30139]	-3.372712
ENSG00000100271	25809	TTLL1	protein_coding	HGNC Symbol	4	tubulin tyrosine ligase like 1 [Source:HGNC Symbol;Acc:HGNC:1312]	-3.357728
ENSG00000214776	NA	AC092821.1	transcribed_unprocessed_pseudogene	Clone-based (Ensembl) gene	3	ovostatin	-3.356861
ENSG00000119514	79695	GALNT12	protein_coding	HGNC Symbol	4	polypeptide N-acetylgalactosaminyltransferase 12 [Source:HGNC Symbol;Acc:HGNC:19877]	-3.356094
ENSG00000249550	1.01E+08	LINC01234	lincRNA	HGNC Symbol	5	long intergenic non-protein coding RNA 1234 [Source:HGNC Symbol;Acc:HGNC:49757]	-3.351505

ENSG00000162105	22941	SHANK2	protein_coding	HGNC Symbol	22	SH3 and multiple ankyrin repeat domains 2 [Source:HGNC Symbol;Acc:HGNC:14295]	-3.349434
ENSG00000231482	NA	AC105450.1	lincRNA	Clone-based (Ensembl) gene	2	novel transcript	-3.348928
ENSG00000129991	7137	TNNI3	protein_coding	HGNC Symbol	9	troponin I3, cardiac type [Source:HGNC Symbol;Acc:HGNC:11947]	-3.348691
ENSG00000239556	NA	AC004951.2	transcribed_unprocessed_pseudogene	Clone-based (Ensembl) gene	1	uroplakin-like protein (UPLP) pseudogene	-3.348637
ENSG00000168970	8681	JMJD7-PLA2G4B	protein_coding	HGNC Symbol	6	JMJD7-PLA2G4B readthrough [Source:HGNC Symbol;Acc:HGNC:34449]	-3.344686
ENSG00000173898	6712	SPTBN2	protein_coding	HGNC Symbol	12	spectrin beta, non-erythrocytic 2 [Source:HGNC Symbol;Acc:HGNC:11276]	-3.341319
ENSG00000134917	11095	ADAMTS8	protein_coding	HGNC Symbol	2	ADAM metallopeptidase with thrombospondin type 1 motif 8 [Source:HGNC Symbol;Acc:HGN	-3.340499
ENSG00000171631	5031	P2RY6	protein_coding	HGNC Symbol	12	pyrimidinergic receptor P2Y6 [Source:HGNC Symbol;Acc:HGNC:8543]	-3.337705
ENSG00000237476	1.02E+08	LINC01637	lincRNA	HGNC Symbol	1	long intergenic non-protein coding RNA 1637 [Source:HGNC Symbol;Acc:HGNC:52424]	-3.335536
ENSG00000100344	80339	PNPLA3	protein_coding	HGNC Symbol	5	patatin like phospholipase domain containing 3 [Source:HGNC Symbol;Acc:HGNC:18590]	-3.334717
ENSG00000172935	116535	MRGPRF	protein_coding	HGNC Symbol	3	MAS related GPR family member F [Source:HGNC Symbol;Acc:HGNC:24828]	-3.332866
ENSG00000110900	441631	TSPAN11	protein_coding	HGNC Symbol	4	tetraspanin 11 [Source:HGNC Symbol;Acc:HGNC:30795]	-3.329184
ENSG00000163701	132014	IL17RE	protein_coding	HGNC Symbol	11	interleukin 17 receptor E [Source:HGNC Symbol;Acc:HGNC:18439]	-3.328592
ENSG00000137825	3706	ITPKA	protein_coding	HGNC Symbol	4	inositol-trisphosphate 3-kinase A [Source:HGNC Symbol;Acc:HGNC:6178]	-3.319558
ENSG00000101311	55612	FERMT1	protein_coding	HGNC Symbol	4	fermitin family member 1 [Source:HGNC Symbol;Acc:HGNC:15889]	-3.318464
ENSG00000133863	56154	TEX15	protein_coding	HGNC Symbol	4	testis expressed 15, meiosis and synapsis associated [Source:HGNC Symbol;Acc:HGNC:11738]	-3.318178
ENSG00000174705	285590	SH3PXD2B	protein_coding	HGNC Symbol	5	SH3 and PX domains 2B [Source:HGNC Symbol;Acc:HGNC:29242]	-3.31546
ENSG00000103024	4832	NME3	protein_coding	HGNC Symbol	11	NME/NM23 nucleoside diphosphate kinase 3 [Source:HGNC Symbol;Acc:HGNC:7851]	-3.314681
ENSG00000106785	9830	TRIM14	protein_coding	HGNC Symbol	6	tripartite motif containing 14 [Source:HGNC Symbol;Acc:HGNC:16283]	-3.312368
ENSG00000179611	NA	DGKZP1	processed_pseudogene	HGNC Symbol	1	diacylglycerol kinase zeta pseudogene 1 [Source:HGNC Symbol;Acc:HGNC:39263]	-3.311784
ENSG00000142621	114827	FHAD1	protein_coding	HGNC Symbol	22	forkhead associated phosphopeptide binding domain 1 [Source:HGNC Symbol;Acc:HGNC:294	-3.308775
ENSG00000213371	NA	NAP1L1P3	processed_pseudogene	HGNC Symbol	1	nucleosome assembly protein 1 like 1 pseudogene 3 [Source:HGNC Symbol;Acc:HGNC:38007]	-3.307538
ENSG00000166402	7275	TUB	protein_coding	HGNC Symbol	3	tubby bipartite transcription factor [Source:HGNC Symbol;Acc:HGNC:12406]	-3.306075
ENSG00000160183	64699	TMPRSS3	protein_coding	HGNC Symbol	9	transmembrane serine protease 3 [Source:HGNC Symbol;Acc:HGNC:11877]	-3.304861
ENSG00000010318	51533	PHF7	protein_coding	HGNC Symbol	11	PHD finger protein 7 [Source:HGNC Symbol;Acc:HGNC:18458]	-3.301172
ENSG00000196700	57473	ZNF512B	protein_coding	HGNC Symbol	1	zinc finger protein 512B [Source:HGNC Symbol;Acc:HGNC:29212]	-3.296973
ENSG00000176204	80059	LRRTM4	protein_coding	HGNC Symbol	7	leucine rich repeat transmembrane neuronal 4 [Source:HGNC Symbol;Acc:HGNC:19411]	-3.296535
ENSG00000072310	6720	SREBF1	protein_coding	HGNC Symbol	23	sterol regulatory element binding transcription factor 1 [Source:HGNC Symbol;Acc:HGNC:112	-3.293552
ENSG00000184005	256435	ST6GALNAC3	protein_coding	HGNC Symbol	3	ST6 N-acetylgalactosaminide alpha-2,6-sialyltransferase 3 [Source:HGNC Symbol;Acc:HGNC:1	-3.292729
ENSG00000197816	1E+08	CCDC180	protein_coding	HGNC Symbol	13	coiled-coil domain containing 180 [Source:HGNC Symbol;Acc:HGNC:29303]	-3.292173
ENSG00000167925	84514	GHDC	protein_coding	HGNC Symbol	12	GH3 domain containing [Source:HGNC Symbol;Acc:HGNC:24438]	-3.290808
ENSG00000119943	84795	PYROXD2	protein_coding	HGNC Symbol	5	pyridine nucleotide-disulphide oxidoreductase domain 2 [Source:HGNC Symbol;Acc:HGNC:23	-3.288485
ENSG00000006638	6915	TBXA2R	protein_coding	HGNC Symbol	4	thromboxane A2 receptor [Source:HGNC Symbol;Acc:HGNC:11608]	-3.28775
ENSG00000085552	57549	IGSF9	protein_coding	HGNC Symbol	6	immunoglobulin superfamily member 9 [Source:HGNC Symbol;Acc:HGNC:18132]	-3.284711
ENSG00000003400	843	CASP10	protein_coding	HGNC Symbol	13	caspase 10 [Source:HGNC Symbol;Acc:HGNC:1500]	-3.276827
ENSG00000115648	79083	MLPH	protein_coding	HGNC Symbol	23	melanophilin [Source:HGNC Symbol;Acc:HGNC:29643]	-3.273856
ENSG00000062822	5424	POLD1	protein_coding	HGNC Symbol	16	DNA polymerase delta 1, catalytic subunit [Source:HGNC Symbol;Acc:HGNC:9175]	-3.272099
ENSG00000007264	4145	MATK	protein_coding	HGNC Symbol	15	megakaryocyte-associated tyrosine kinase [Source:HGNC Symbol;Acc:HGNC:6906]	-3.269299
ENSG00000233608	117581	TWIST2	protein_coding	HGNC Symbol	2	twist family bHLH transcription factor 2 [Source:HGNC Symbol;Acc:HGNC:20670]	-3.267517
ENSG00000114735	51409	HEMK1	protein_coding	HGNC Symbol	7	HemK methyltransferase family member 1 [Source:HGNC Symbol;Acc:HGNC:24923]	-3.262659
ENSG00000185101	338440	ANO9	protein_coding	HGNC Symbol	8	anoctamin 9 [Source:HGNC Symbol;Acc:HGNC:20679]	-3.260169
ENSG00000142765	84958	SYTL1	protein_coding	HGNC Symbol	11	synaptotagmin like 1 [Source:HGNC Symbol;Acc:HGNC:15584]	-3.258468
ENSG00000238105	55592	GOLGA2P5	transcribed_unprocessed_pseudogene	HGNC Symbol	7	GOLGA2 pseudogene 5 [Source:HGNC Symbol;Acc:HGNC:25315]	-3.257816
ENSG00000111319	6337	SCNN1A	protein_coding	HGNC Symbol	23	sodium channel epithelial 1 alpha subunit [Source:HGNC Symbol;Acc:HGNC:10599]	-3.255732
ENSG00000107902	64077	LHPP	protein_coding	HGNC Symbol	9	phospholysine phosphohistidine inorganic pyrophosphate phosphatase [Source:HGNC Symbo	-3.253233
ENSG00000115524	23451	SF3B1	protein_coding	HGNC Symbol	12	splicing factor 3b subunit 1 [Source:HGNC Symbol;Acc:HGNC:10768]	-3.252253
ENSG00000236305	1.05E+08	SLC12A9-AS1	antisense	HGNC Symbol	1	SLC12A9 antisense RNA 1 [Source:HGNC Symbol;Acc:HGNC:40807]	-3.250801
ENSG00000197879	4641	MYO1C	protein_coding	HGNC Symbol	22	myosin IC [Source:HGNC Symbol;Acc:HGNC:7597]	-3.248263
ENSG00000251615	NA	AC104825.1	lincRNA	Clone-based (Ensembl) gene	1	novel transcript	-3.243663
ENSG00000267147	1.02E+08	LINC01842	lincRNA	HGNC Symbol	1	long intergenic non-protein coding RNA 1842 [Source:HGNC Symbol;Acc:HGNC:52656]	-3.242216
ENSG00000173442	254102	EHBP1L1	protein_coding	HGNC Symbol	11	EH domain binding protein 1 like 1 [Source:HGNC Symbol;Acc:HGNC:30682]	-3.238137
ENSG00000172478	79919	MAB21L4	protein_coding	HGNC Symbol	5	mab-21 like 4 [Source:HGNC Symbol;Acc:HGNC:26216]	-3.235354
ENSG00000182362	54059	YBEY	protein_coding	HGNC Symbol	8	ybeY metalloendoribonuclease [Source:HGNC Symbol;Acc:HGNC:1299]	-3.233844
ENSG00000103197	7249	TSC2	protein_coding	HGNC Symbol	69	TSC complex subunit 2 [Source:HGNC Symbol;Acc:HGNC:12363]	-3.232299
ENSG00000186399	1.01E+08	GOLGA8R	protein_coding	HGNC Symbol	2	golgin A8 family member R [Source:HGNC Symbol;Acc:HGNC:44407]	-3.230934
ENSG00000162836	51205	ACP6	protein_coding	HGNC Symbol	10	acid phosphatase 6, lysophosphatidic [Source:HGNC Symbol;Acc:HGNC:29609]	-3.228487
ENSG00000257698	NA	GIHCG	lincRNA	HGNC Symbol	4	GIHCG, inhibitor of miR-200b/200a/429 expression [Source:HGNC Symbol;Acc:HGNC:52649]	-3.228289
ENSG00000187630	317749	DHRS4L2	protein_coding	HGNC Symbol	11	dehydrogenase/reductase 4 like 2 [Source:HGNC Symbol;Acc:HGNC:19731]	-3.228191
ENSG00000227051	56967	C14orf132	protein_coding	HGNC Symbol	4	chromosome 14 open reading frame 132 [Source:HGNC Symbol;Acc:HGNC:20346]	-3.228148
ENSG00000072571	3161	HMMR	protein_coding	HGNC Symbol	8	hyaluronan mediated motility receptor [Source:HGNC Symbol;Acc:HGNC:5012]	-3.226478
ENSG00000143624	65123	INTS3	protein_coding	HGNC Symbol	11	integrator complex subunit 3 [Source:HGNC Symbol;Acc:HGNC:26153]	-3.226243
ENSG00000226137	440465	BAIAP2-DT	lincRNA	HGNC Symbol	2	BAIAP2 divergent transcript [Source:HGNC Symbol;Acc:HGNC:44342]	-3.224752
ENSG00000075643	55034	MOCOS	protein_coding	HGNC Symbol	2	molybdenum cofactor sulfurase [Source:HGNC Symbol;Acc:HGNC:18234]	-3.224379
ENSG00000267886	NA	AC074135.1	lincRNA	Clone-based (Ensembl) gene	2		-3.223136
ENSG00000181433	55511	SAGE1	protein_coding	HGNC Symbol	3	sarcoma antigen 1 [Source:HGNC Symbol;Acc:HGNC:30369]	-3.221043

ENSG00000102125	6901	TAZ	protein_coding	HGNC Symbol	24	tafazzin [Source:HGNC Symbol;Acc:HGNC:11577]	-3.220631
ENSG00000186193	89958	SAPCD2	protein_coding	HGNC Symbol	1	suppressor APC domain containing 2 [Source:HGNC Symbol;Acc:HGNC:28055]	-3.219259
ENSG00000196372	79754	ASB13	protein_coding	HGNC Symbol	5	ankyrin repeat and SOCS box containing 13 [Source:HGNC Symbol;Acc:HGNC:19765]	-3.215225
ENSG00000263961	440712	RHEX	protein_coding	HGNC Symbol	5	regulator of hemoglobinization and erythroid cell expansion [Source:HGNC Symbol;Acc:HGNC	-3.212651
ENSG00000149476	26007	TKFC	protein_coding	HGNC Symbol	21	triokinase and FMN cyclase [Source:HGNC Symbol;Acc:HGNC:24552]	-3.208289
ENSG00000185818	339983	NAT8L	protein_coding	HGNC Symbol	2	N-acetyltransferase 8 like [Source:HGNC Symbol;Acc:HGNC:26742]	-3.208254
ENSG00000004777	115703	ARHGAP33	protein_coding	HGNC Symbol	13	Rho GTPase activating protein 33 [Source:HGNC Symbol;Acc:HGNC:23085]	-3.208067
ENSG00000187554	7100	TLR5	protein_coding	HGNC Symbol	9	toll like receptor 5 [Source:HGNC Symbol;Acc:HGNC:11851]	-3.206664
ENSG00000167702	90990	KIFC2	protein_coding	HGNC Symbol	9	kinesin family member C2 [Source:HGNC Symbol;Acc:HGNC:29530]	-3.206616
ENSG00000171931	10517	FBXW10	protein_coding	HGNC Symbol	5	F-box and WD repeat domain containing 10 [Source:HGNC Symbol;Acc:HGNC:1211]	-3.204657
ENSG00000196890	128312	HIST3H2BB	protein_coding	HGNC Symbol	1	histone cluster 3 H2B family member b [Source:HGNC Symbol;Acc:HGNC:20514]	-3.203886
ENSG00000120915	2053	EPHX2	protein_coding	HGNC Symbol	12	epoxide hydrolase 2 [Source:HGNC Symbol;Acc:HGNC:3402]	-3.199652
ENSG00000165675	10495	ENOX2	protein_coding	HGNC Symbol	7	ecto-NOX disulfide-thiol exchanger 2 [Source:HGNC Symbol;Acc:HGNC:2259]	-3.196539
ENSG00000260401	NA	AP002761.4	sense_overlapping	Clone-based (Ensembl) gene	1	novel transcript, overlapping to P2RY2	-3.196246
ENSG00000143412	8416	ANXA9	protein_coding	HGNC Symbol	2	annexin A9 [Source:HGNC Symbol;Acc:HGNC:547]	-3.193636
ENSG00000111664	2784	GNB3	protein_coding	HGNC Symbol	9	G protein subunit beta 3 [Source:HGNC Symbol;Acc:HGNC:4400]	-3.19272
ENSG00000142459	115704	EVI5L	protein_coding	HGNC Symbol	7	ecotropic viral integration site 5 like [Source:HGNC Symbol;Acc:HGNC:30464]	-3.189528
ENSG00000229867	1.01E+08	STEAP3-AS1	antisense	HGNC Symbol	1	STEAP3 antisense RNA 1 [Source:HGNC Symbol;Acc:HGNC:41053]	-3.189376
ENSG00000265205	NA	AC010761.3	antisense	Clone-based (Ensembl) gene	1	novel transcript, antisense to SUPT6H	-3.186877
ENSG00000151876	26272	FBXO4	protein_coding	HGNC Symbol	7	F-box protein 4 [Source:HGNC Symbol;Acc:HGNC:13583]	-3.186112
ENSG00000167971	57524	CASKIN1	protein_coding	HGNC Symbol	3	CASK interacting protein 1 [Source:HGNC Symbol;Acc:HGNC:20879]	-3.185053
ENSG00000132361	23277	CLUH	protein_coding	HGNC Symbol	14	clustered mitochondria homolog [Source:HGNC Symbol;Acc:HGNC:29094]	-3.183262
ENSG00000236008	1.02E+08	LINC01814	lincRNA	HGNC Symbol	6	long intergenic non-protein coding RNA 1814 [Source:HGNC Symbol;Acc:HGNC:52618]	-3.183117
ENSG00000231240	NA	KLF2P1	processed_pseudogene	HGNC Symbol	1	Kruppel like factor 2 pseudogene 1 [Source:HGNC Symbol;Acc:HGNC:49280]	-3.179323
ENSG00000272316	NA	AL021368.2	lincRNA	Clone-based (Ensembl) gene	1	novel transcript	-3.178583
ENSG00000128340	5880	RAC2	protein_coding	HGNC Symbol	7	Rac family small GTPase 2 [Source:HGNC Symbol;Acc:HGNC:9802]	-3.177433
ENSG00000162591	1953	MEGF6	protein_coding	HGNC Symbol	7	multiple EGF like domains 6 [Source:HGNC Symbol;Acc:HGNC:3232]	-3.175053
ENSG00000109063	4621	MYH3	protein_coding	HGNC Symbol	5	myosin heavy chain 3 [Source:HGNC Symbol;Acc:HGNC:7573]	-3.169518
ENSG00000172086	51315	KRCC1	protein_coding	HGNC Symbol	1	lysine rich coiled-coil 1 [Source:HGNC Symbol;Acc:HGNC:28039]	-3.169055
ENSG00000198246	55315	SLC29A3	protein_coding	HGNC Symbol	14	solute carrier family 29 member 3 [Source:HGNC Symbol;Acc:HGNC:23096]	-3.168439
ENSG00000276130	5002	SLC22A18	protein_coding	HGNC Symbol	13	solute carrier family 22 member 18 [Source:HGNC Symbol;Acc:HGNC:10964]	-3.167114
ENSG00000185386	5600	MAPK11	protein_coding	HGNC Symbol	4	mitogen-activated protein kinase 11 [Source:HGNC Symbol;Acc:HGNC:6873]	-3.166887
ENSG00000107957	9644	SH3PXD2A	protein_coding	HGNC Symbol	4	SH3 and PX domains 2A [Source:HGNC Symbol;Acc:HGNC:23664]	-3.165818
ENSG00000125246	171425	CLYBL	protein_coding	HGNC Symbol	7	citrate lyase beta like [Source:HGNC Symbol;Acc:HGNC:18355]	-3.16465
ENSG00000131969	145447	ABHD12B	protein_coding	HGNC Symbol	9	abhydrolase domain containing 12B [Source:HGNC Symbol;Acc:HGNC:19837]	-3.164237
ENSG00000234705	NA	HMGA1P4	antisense	HGNC Symbol	1	high mobility group AT-hook 1 pseudogene 4 [Source:HGNC Symbol;Acc:HGNC:39093]	-3.159646
ENSG00000111344	8437	RASAL1	protein_coding	HGNC Symbol	10	RAS protein activator like 1 [Source:HGNC Symbol;Acc:HGNC:9873]	-3.157394
ENSG00000204791	NA	SMPD5	transcribed_unitary_pseudogene	HGNC Symbol	2	sphingomyelin phosphodiesterase 5 [Source:HGNC Symbol;Acc:HGNC:52275]	-3.155039
ENSG00000160285	4047	LSS	protein_coding	HGNC Symbol	11	lanosterol synthase [Source:HGNC Symbol;Acc:HGNC:6708]	-3.152189
ENSG00000169064	79740	ZBBX	protein_coding	HGNC Symbol	13	zinc finger B-box domain containing [Source:HGNC Symbol;Acc:HGNC:26245]	-3.149511
ENSG00000170638	80305	TRABD	protein_coding	HGNC Symbol	6	TraB domain containing [Source:HGNC Symbol;Acc:HGNC:28805]	-3.148168
ENSG00000100304	23170	TTLL12	protein_coding	HGNC Symbol	4	tubulin tyrosine ligase like 12 [Source:HGNC Symbol;Acc:HGNC:28974]	-3.144167
ENSG00000205634	400932	LINC00898	lincRNA	HGNC Symbol	3	long intergenic non-protein coding RNA 898 [Source:HGNC Symbol;Acc:HGNC:48581]	-3.143632
ENSG00000184194	54328	GPR173	protein_coding	HGNC Symbol	2	G protein-coupled receptor 173 [Source:HGNC Symbol;Acc:HGNC:18186]	-3.143126
ENSG00000260220	399693	CCDC187	protein_coding	HGNC Symbol	5	coiled-coil domain containing 187 [Source:HGNC Symbol;Acc:HGNC:30942]	-3.142308
ENSG00000230626	NA	AC011005.1	protein_coding	Clone-based (Ensembl) gene	1	novel protein similar to mitogen-activated protein kinase kinase 2 MAP2K2	-3.138213
ENSG00000226723	NA	AL513175.1	processed_pseudogene	Clone-based (Ensembl) gene	1	pseudogene similar to ATP-binding cassette, sub-family E (OABP), member (ABCE1)	-3.13522
ENSG00000168491	256309	CCDC110	protein_coding	HGNC Symbol	9	coiled-coil domain containing 110 [Source:HGNC Symbol;Acc:HGNC:28504]	-3.133188
ENSG00000257660	1.01E+08	AC117498.2	lincRNA	Clone-based (Ensembl) gene	2		-3.132634
ENSG00000170629	349152	DPY19L2P2	transcribed_unprocessed_pseudogene	HGNC Symbol	6	DPY19L2 pseudogene 2 [Source:HGNC Symbol;Acc:HGNC:21764]	-3.132323
ENSG00000076344	8786	RGS11	protein_coding	HGNC Symbol	8	regulator of G protein signaling 11 [Source:HGNC Symbol;Acc:HGNC:9993]	-3.130269
ENSG00000267533	NA	AP002414.4	processed_pseudogene	Clone-based (Ensembl) gene	1	single stranded DNA binding protein 4 (SSBP4) pseudogene	-3.127108
ENSG00000238278	NA	ALG1L6P	unprocessed_pseudogene	HGNC Symbol	1	asparagine-linked glycosylation 1-like 6, pseudogene [Source:HGNC Symbol;Acc:HGNC:44375]	-3.125656
ENSG00000204381	143903	LAYN	protein_coding	HGNC Symbol	10	layilin [Source:HGNC Symbol;Acc:HGNC:29471]	-3.125314
ENSG00000139428	326625	MMAB	protein_coding	HGNC Symbol	10	metabolism of cobalamin associated B [Source:HGNC Symbol;Acc:HGNC:19331]	-3.123568
ENSG00000121851	84265	POLR3GL	protein_coding	HGNC Symbol	4	RNA polymerase III subunit G like [Source:HGNC Symbol;Acc:HGNC:28466]	-3.122153
ENSG00000183048	1468	SLC25A10	protein_coding	HGNC Symbol	8	solute carrier family 25 member 10 [Source:HGNC Symbol;Acc:HGNC:10980]	-3.120355
ENSG00000224785	NA	AC006026.1	processed_pseudogene	Clone-based (Ensembl) gene	1	ATP-binding cassette, sub-family E (OABP), member 1 (ABCE1) pseudogene	-3.120072
ENSG00000100968	4776	NFATC4	protein_coding	HGNC Symbol	33	nuclear factor of activated T cells 4 [Source:HGNC Symbol;Acc:HGNC:7778]	-3.117646
ENSG00000233080	1.04E+08	LINC01399	transcribed_processed_pseudogene	HGNC Symbol	2	long intergenic non-protein coding RNA 1399 [Source:HGNC Symbol;Acc:HGNC:50680]	-3.116331
ENSG00000254343	NA	AC091563.1	lincRNA	Clone-based (Ensembl) gene	2	novel transcript	-3.116137
ENSG00000067836	79641	ROGDI	protein_coding	HGNC Symbol	16	rogdi homolog [Source:HGNC Symbol;Acc:HGNC:29478]	-3.115943
ENSG00000168427	377007	KLHL30	protein_coding	HGNC Symbol	1	kelch like family member 30 [Source:HGNC Symbol;Acc:HGNC:24770]	-3.114661
ENSG00000073050	7515	XRCC1	protein_coding	HGNC Symbol	9	X-ray repair cross complementing 1 [Source:HGNC Symbol;Acc:HGNC:12828]	-3.112887
ENSG00000220517	NA	ASS1P1	processed_pseudogene	HGNC Symbol	1	argininosuccinate synthetase 1 pseudogene 1 [Source:HGNC Symbol;Acc:HGNC:759]	-3.110508

ENSG00000167524	124923	SGK494	protein_coding	NCBI gene	12	uncharacterized serine/threonine-protein kinase SgK494 [Source:NCBI gene;Acc:124923]	-3.110147
ENSG00000174640	80111	SLCO2A1	protein_coding	HGNC Symbol	9	solute carrier organic anion transporter family member 2A1 [Source:HGNC Symbol;Acc:HGNC	-3.109981
ENSG00000174640	6578	SLCO2A1	protein_coding	HGNC Symbol	9	solute carrier organic anion transporter family member 2A1 [Source:HGNC Symbol;Acc:HGNC	-3.109981
ENSG00000106733	54981	NMRK1	protein_coding	HGNC Symbol	7	nicotinamide riboside kinase 1 [Source:HGNC Symbol;Acc:HGNC:26057]	-3.109567
ENSG00000049540	2006	ELN	protein_coding	HGNC Symbol	34	elastin [Source:HGNC Symbol;Acc:HGNC:3327]	-3.104839
ENSG00000155792	64798	DEPTOR	protein_coding	HGNC Symbol	3	DEP domain containing MTOR interacting protein [Source:HGNC Symbol;Acc:HGNC:22953]	-3.10197
ENSG00000172367	79849	PDZD3	protein_coding	HGNC Symbol	14	PDZ domain containing 3 [Source:HGNC Symbol;Acc:HGNC:19891]	-3.099718
ENSG00000132382	10514	MYBBP1A	protein_coding	HGNC Symbol	13	MYB binding protein 1a [Source:HGNC Symbol;Acc:HGNC:7546]	-3.096378
ENSG00000215788	8718	TNFRSF25	protein_coding	HGNC Symbol	21	TNF receptor superfamily member 25 [Source:HGNC Symbol;Acc:HGNC:11910]	-3.094624
ENSG00000120471	63970	TP53AIP1	protein_coding	HGNC Symbol	6	tumor protein p53 regulated apoptosis inducing protein 1 [Source:HGNC Symbol;Acc:HGNC:2	-3.094207
ENSG00000026950	11119	BTN3A1	protein_coding	HGNC Symbol	11	butyrophilin subfamily 3 member A1 [Source:HGNC Symbol;Acc:HGNC:1138]	-3.09282
ENSG00000076555	32	ACACB	protein_coding	HGNC Symbol	15	acetyl-CoA carboxylase beta [Source:HGNC Symbol;Acc:HGNC:85]	-3.092798
ENSG00000108947	1949	EFNB3	protein_coding	HGNC Symbol	1	ephrin B3 [Source:HGNC Symbol;Acc:HGNC:3228]	-3.089689
ENSG00000101224	994	CDC25B	protein_coding	HGNC Symbol	9	cell division cycle 25B [Source:HGNC Symbol;Acc:HGNC:1726]	-3.089401
ENSG00000143126	1952	CELSR2	protein_coding	HGNC Symbol	4	cadherin EGF LAG seven-pass G-type receptor 2 [Source:HGNC Symbol;Acc:HGNC:3231]	-3.089056
ENSG00000117543	51611	DPH5	protein_coding	HGNC Symbol	15	diphthamide biosynthesis 5 [Source:HGNC Symbol;Acc:HGNC:24270]	-3.088184
ENSG00000187867	342979	PALM3	protein_coding	HGNC Symbol	3	paralemmin 3 [Source:HGNC Symbol;Acc:HGNC:33274]	-3.087463
ENSG00000181031	9501	RPH3AL	protein_coding	HGNC Symbol	22	rabphilin 3A like (without C2 domains) [Source:HGNC Symbol;Acc:HGNC:10296]	-3.08727
ENSG00000245248	NA	USP2-AS1	antisense	HGNC Symbol	5	USP2 antisense RNA 1 (head to head) [Source:HGNC Symbol;Acc:HGNC:48673]	-3.087256
ENSG00000147251	139818	DOCK11	protein_coding	HGNC Symbol	4	dedicator of cytokinesis 11 [Source:HGNC Symbol;Acc:HGNC:23483]	-3.086571
ENSG00000178445	2731	GLDC	protein_coding	HGNC Symbol	22	glycine decarboxylase [Source:HGNC Symbol;Acc:HGNC:4313]	-3.083049
ENSG00000239697	8742	TNFSF12	protein_coding	HGNC Symbol	4	TNF superfamily member 12 [Source:HGNC Symbol;Acc:HGNC:11927]	-3.082425
ENSG00000181790	575	ADGRB1	protein_coding	HGNC Symbol	6	adhesion G protein-coupled receptor B1 [Source:HGNC Symbol;Acc:HGNC:943]	-3.082037
ENSG00000175575	80227	PAAF1	protein_coding	HGNC Symbol	16	proteasomal ATPase associated factor 1 [Source:HGNC Symbol;Acc:HGNC:25687]	-3.081962
ENSG00000117305	3155	HMGCL	protein_coding	HGNC Symbol	9	3-hydroxy-3-methylglutaryl-CoA lyase [Source:HGNC Symbol;Acc:HGNC:5005]	-3.081872
ENSG00000197785	55210	ATAD3A	protein_coding	HGNC Symbol	7	ATPase family, AAA domain containing 3A [Source:HGNC Symbol;Acc:HGNC:25567]	-3.081352
ENSG00000197558	23145	SSPO	protein_coding	HGNC Symbol	11	SCO-spondin [Source:HGNC Symbol;Acc:HGNC:21998]	-3.081281
ENSG00000104812	2997	GYS1	protein_coding	HGNC Symbol	7	glycogen synthase 1 [Source:HGNC Symbol;Acc:HGNC:4706]	-3.077033
ENSG00000262185	1.03E+08	AC005736.1	lincRNA	Clone-based (Ensembl) gene	3		-3.076094
ENSG00000105655	51477	ISYNA1	protein_coding	HGNC Symbol	14	inositol-3-phosphate synthase 1 [Source:HGNC Symbol;Acc:HGNC:29821]	-3.074131
ENSG00000149451	80332	ADAM33	protein_coding	HGNC Symbol	7	ADAM metallopeptidase domain 33 [Source:HGNC Symbol;Acc:HGNC:15478]	-3.071713
ENSG00000183145	53820	RIPPLY3	protein_coding	HGNC Symbol	3	ripply transcriptional repressor 3 [Source:HGNC Symbol;Acc:HGNC:3047]	-3.071533
ENSG00000100321	9145	SYNGR1	protein_coding	HGNC Symbol	7	synaptogyrin 1 [Source:HGNC Symbol;Acc:HGNC:11498]	-3.071091
ENSG00000148341	56904	SH3GLB2	protein_coding	HGNC Symbol	12	SH3 domain containing GRB2 like, endophilin B2 [Source:HGNC Symbol;Acc:HGNC:10834]	-3.070844
ENSG00000184867	9823	ARMCX2	protein_coding	HGNC Symbol	13	armadillo repeat containing X-linked 2 [Source:HGNC Symbol;Acc:HGNC:16869]	-3.070697
ENSG00000186792	8372	HYAL3	protein_coding	HGNC Symbol	7	hyaluronidase 3 [Source:HGNC Symbol;Acc:HGNC:5322]	-3.068254
ENSG00000108352	51195	RAPGEFL1	protein_coding	HGNC Symbol	11	Rap guanine nucleotide exchange factor like 1 [Source:HGNC Symbol;Acc:HGNC:17428]	-3.068037
ENSG00000227188	1.05E+08	MGAT3-AS1	antisense	HGNC Symbol	1	MGAT3 antisense RNA 1 [Source:HGNC Symbol;Acc:HGNC:51356]	-3.06725
ENSG00000110628	5002	SLC22A18	protein_coding	HGNC Symbol	14	solute carrier family 22 member 18 [Source:HGNC Symbol;Acc:HGNC:10964]	-3.067023
ENSG00000214456	440503	PLIN5	protein_coding	HGNC Symbol	6	perilipin 5 [Source:HGNC Symbol;Acc:HGNC:33196]	-3.065972
ENSG00000118971	894	CCND2	protein_coding	HGNC Symbol	4	cyclin D2 [Source:HGNC Symbol;Acc:HGNC:1583]	-3.063914
ENSG00000238000	NA	AC116347.1	processed_pseudogene	Clone-based (Ensembl) gene	1	proteasome (prosome, macropain) activator subunit 2 (PA28 beta) (PSME2) pseudogene	-3.063451
ENSG00000099308	23031	MAST3	protein_coding	HGNC Symbol	5	microtubule associated serine/threonine kinase 3 [Source:HGNC Symbol;Acc:HGNC:19036]	-3.062542
ENSG00000174233	112	ADCY6	protein_coding	HGNC Symbol	9	adenylate cyclase 6 [Source:HGNC Symbol;Acc:HGNC:237]	-3.062319
ENSG00000135119	84900	RNFT2	protein_coding	HGNC Symbol	9	ring finger protein, transmembrane 2 [Source:HGNC Symbol;Acc:HGNC:25905]	-3.060851
ENSG00000142273	23624	CBLC	protein_coding	HGNC Symbol	4	Cbl proto-oncogene C [Source:HGNC Symbol;Acc:HGNC:15961]	-3.060324
ENSG00000108469	9400	RECQL5	protein_coding	HGNC Symbol	19	RecQ like helicase 5 [Source:HGNC Symbol;Acc:HGNC:9950]	-3.060297
ENSG00000073670	4185	ADAM11	protein_coding	HGNC Symbol	5	ADAM metallopeptidase domain 11 [Source:HGNC Symbol;Acc:HGNC:189]	-3.059672
ENSG00000185875	79896	THNSL1	protein_coding	HGNC Symbol	2	threonine synthase like 1 [Source:HGNC Symbol;Acc:HGNC:26160]	-3.054256
ENSG00000159214	149473	CCDC24	protein_coding	HGNC Symbol	11	coiled-coil domain containing 24 [Source:HGNC Symbol;Acc:HGNC:28688]	-3.054201
ENSG00000120925	81790	RNF170	protein_coding	HGNC Symbol	9	ring finger protein 170 [Source:HGNC Symbol;Acc:HGNC:25358]	-3.053214
ENSG00000257556	NA	LINC02298	lincRNA	HGNC Symbol	3	long intergenic non-protein coding RNA 2298 [Source:HGNC Symbol;Acc:HGNC:53216]	-3.051915
ENSG00000260456	1.01E+08	C16orf95	protein_coding	HGNC Symbol	7	chromosome 16 open reading frame 95 [Source:HGNC Symbol;Acc:HGNC:40033]	-3.050824
ENSG00000142233	126147	NTN5	protein_coding	HGNC Symbol	3	netrin 5 [Source:HGNC Symbol;Acc:HGNC:25208]	-3.049755
ENSG00000140057	122481	AK7	protein_coding	HGNC Symbol	5	adenylate kinase 7 [Source:HGNC Symbol;Acc:HGNC:20091]	-3.049574
ENSG00000270276	554313	HIST2H4B	protein_coding	HGNC Symbol	3	histone cluster 2 H4 family member b [Source:HGNC Symbol;Acc:HGNC:29607]	-3.048213
ENSG00000106003	3955	LFNG	protein_coding	HGNC Symbol	7	LFNG O-fucosylpeptide 3-beta-N-acetylglucosaminyltransferase [Source:HGNC Symbol;Acc:H(	-3.046752
ENSG00000153292	266977	ADGRF1	protein_coding	HGNC Symbol	13	adhesion G protein-coupled receptor F1 [Source:HGNC Symbol;Acc:HGNC:18990]	-3.04638
ENSG00000090971	57106	NAT14	protein_coding	HGNC Symbol	5	N-acetyltransferase 14 (putative) [Source:HGNC Symbol;Acc:HGNC:28918]	-3.045424
ENSG00000182272	338707	B4GALNT4	protein_coding	HGNC Symbol	5	beta-1,4-N-acetyl-galactosaminyltransferase 4 [Source:HGNC Symbol;Acc:HGNC:26315]	-3.044043
ENSG00000148288	26301	GBGT1	protein_coding	HGNC Symbol	12	globoside alpha-1,3-N-acetylgalactosaminyltransferase 1 (FORS blood group) [Source:HGNC S	-3.041425
ENSG00000134569	4038	LRP4	protein_coding	HGNC Symbol	5	LDL receptor related protein 4 [Source:HGNC Symbol;Acc:HGNC:6696]	-3.040264
ENSG00000133027	10400	PEMT	protein_coding	HGNC Symbol	13	phosphatidylethanolamine N-methyltransferase [Source:HGNC Symbol;Acc:HGNC:8830]	-3.039347
ENSG00000169418	4881	NPR1	protein_coding	HGNC Symbol	3	natriuretic peptide receptor 1 [Source:HGNC Symbol;Acc:HGNC:7943]	-3.038842
ENSG00000278619	79922	MRM1	protein_coding	HGNC Symbol	2	mitochondrial rRNA methyltransferase 1 [Source:HGNC Symbol;Acc:HGNC:26202]	-3.038757

ENSG00000100867	10202	DHRS2	protein_coding	HGNC Symbol	10	dehydrogenase/reductase 2 [Source:HGNC Symbol;Acc:HGNC:18349]	-3.035109
ENSG00000223396	NA	RPS10P7	transcribed_processed_pseudogene	HGNC Symbol	3	ribosomal protein S10 pseudogene 7 [Source:HGNC Symbol;Acc:HGNC:36423]	-3.031666
ENSG00000166250	79827	CLMP	protein_coding	HGNC Symbol	4	CXADR like membrane protein [Source:HGNC Symbol;Acc:HGNC:24039]	-3.03004
ENSG00000111087	2735	GLI1	protein_coding	HGNC Symbol	8	GLI family zinc finger 1 [Source:HGNC Symbol;Acc:HGNC:4317]	-3.026678
ENSG00000027001	4285	MIPEP	protein_coding	HGNC Symbol	5	mitochondrial intermediate peptidase [Source:HGNC Symbol;Acc:HGNC:7104]	-3.024858
ENSG00000165644	118881	COMTD1	protein_coding	HGNC Symbol	7	catechol-O-methyltransferase domain containing 1 [Source:HGNC Symbol;Acc:HGNC:26309]	-3.022026
ENSG00000103154	54550	NECAB2	protein_coding	HGNC Symbol	5	N-terminal EF-hand calcium binding protein 2 [Source:HGNC Symbol;Acc:HGNC:23746]	-3.020736
ENSG00000130270	148229	ATP8B3	protein_coding	HGNC Symbol	8	ATPase phospholipid transporting 8B3 [Source:HGNC Symbol;Acc:HGNC:13535]	-3.019241
ENSG00000173546	1464	CSPG4	protein_coding	HGNC Symbol	1	chondroitin sulfate proteoglycan 4 [Source:HGNC Symbol;Acc:HGNC:2466]	-3.018555
ENSG00000186523	85002	FAM86B1	protein_coding	HGNC Symbol	19	family with sequence similarity 86 member B1 [Source:HGNC Symbol;Acc:HGNC:28268]	-3.018118
ENSG00000143375	57530	CGN	protein_coding	HGNC Symbol	8	cingulin [Source:HGNC Symbol;Acc:HGNC:17429]	-3.016607
ENSG00000160767	10712	FAM189B	protein_coding	HGNC Symbol	10	family with sequence similarity 189 member B [Source:HGNC Symbol;Acc:HGNC:1233]	-3.0162
ENSG00000144199	151313	FAHD2B	protein_coding	HGNC Symbol	6	fumarylacetoacetate hydrolase domain containing 2B [Source:HGNC Symbol;Acc:HGNC:2531	-3.014863
ENSG00000227630	1.01E+08	LINC01132	lincRNA	HGNC Symbol	3	long intergenic non-protein coding RNA 1132 [Source:HGNC Symbol;Acc:HGNC:49444]	-3.013012
ENSG00000150281	1489	CTF1	protein_coding	HGNC Symbol	2	cardiotrophin 1 [Source:HGNC Symbol;Acc:HGNC:2499]	-3.007121
ENSG00000186496	252884	ZNF396	protein_coding	HGNC Symbol	6	zinc finger protein 396 [Source:HGNC Symbol;Acc:HGNC:18824]	-3.006808
ENSG00000108641	27077	B9D1	protein_coding	HGNC Symbol	20	B9 domain containing 1 [Source:HGNC Symbol;Acc:HGNC:24123]	-3.006462
ENSG00000068400	56850	GRIPAP1	protein_coding	HGNC Symbol	15	GRIP1 associated protein 1 [Source:HGNC Symbol;Acc:HGNC:18706]	-3.00575
ENSG00000107281	56654	NPDC1	protein_coding	HGNC Symbol	6	neural proliferation, differentiation and control 1 [Source:HGNC Symbol;Acc:HGNC:7899]	-3.000836
ENSG00000008710	5310	PKD1	protein_coding	HGNC Symbol	40	polycystin 1, transient receptor potential channel interacting [Source:HGNC Symbol;Acc:HGN	-3.000753
ENSG00000205129	441054	C4orf47	protein_coding	HGNC Symbol	4	chromosome 4 open reading frame 47 [Source:HGNC Symbol;Acc:HGNC:34346]	-2.998925
ENSG00000203999	284751	LINC01270	lincRNA	HGNC Symbol	2	long intergenic non-protein coding RNA 1270 [Source:HGNC Symbol;Acc:HGNC:27658]	-2.993849
ENSG00000107020	55848	PLGRKT	protein_coding	HGNC Symbol	4	plasminogen receptor with a C-terminal lysine [Source:HGNC Symbol;Acc:HGNC:23633]	-2.993514
ENSG00000168135	3761	KCNJ4	protein_coding	HGNC Symbol	1	potassium voltage-gated channel subfamily J member 4 [Source:HGNC Symbol;Acc:HGNC:62	-2.993126
ENSG00000184916	3714	JAG2	protein_coding	HGNC Symbol	5	jagged 2 [Source:HGNC Symbol;Acc:HGNC:6189]	-2.992936
ENSG00000160293	7410	VAV2	protein_coding	HGNC Symbol	5	vav guanine nucleotide exchange factor 2 [Source:HGNC Symbol;Acc:HGNC:12658]	-2.990257
ENSG00000139410	113675	SDSL	protein_coding	HGNC Symbol	6	serine dehydratase like [Source:HGNC Symbol;Acc:HGNC:30404]	-2.988958
ENSG00000180178	NA	FAR2P1	transcribed_unprocessed_pseudogene	HGNC Symbol	6	fatty acyl-CoA reductase 2 pseudogene 1 [Source:HGNC Symbol;Acc:HGNC:49284]	-2.984004
ENSG00000117643	57134	MAN1C1	protein_coding	HGNC Symbol	8	mannosidase alpha class 1C member 1 [Source:HGNC Symbol;Acc:HGNC:19080]	-2.980003
ENSG00000138036	51626	DYNC2LI1	protein_coding	HGNC Symbol	10	dynein cytoplasmic 2 light intermediate chain 1 [Source:HGNC Symbol;Acc:HGNC:24595]	-2.979295
ENSG00000196684	84941	HSH2D	protein_coding	HGNC Symbol	8	hematopoietic SH2 domain containing [Source:HGNC Symbol;Acc:HGNC:24920]	-2.975687
ENSG00000213177	NA	PRDX2P4	processed_pseudogene	HGNC Symbol	1	peroxiredoxin 2 pseudogene 4 [Source:HGNC Symbol;Acc:HGNC:44969]	-2.975652
ENSG00000163884	28999	KLF15	protein_coding	HGNC Symbol	2	Kruppel like factor 15 [Source:HGNC Symbol;Acc:HGNC:14536]	-2.97538
ENSG00000204323	643008	SMIM5	protein_coding	HGNC Symbol	3	small integral membrane protein 5 [Source:HGNC Symbol;Acc:HGNC:40030]	-2.975071
ENSG00000149474	57325	KAT14	protein_coding	HGNC Symbol	7	lysine acetyltransferase 14 [Source:HGNC Symbol;Acc:HGNC:15904]	-2.974553
ENSG00000166558	146167	SLC38A8	protein_coding	HGNC Symbol	4	solute carrier family 38 member 8 [Source:HGNC Symbol;Acc:HGNC:32434]	-2.973131
ENSG00000274276	1.03E+08	CBSL	protein_coding	HGNC Symbol	10	cystathionine-beta-synthase like [Source:HGNC Symbol;Acc:HGNC:51829]	-2.970511
ENSG00000162571	254173	TTLL10	protein_coding	HGNC Symbol	7	tubulin tyrosine ligase like 10 [Source:HGNC Symbol;Acc:HGNC:26693]	-2.970229
ENSG00000156140	9508	ADAMTS3	protein_coding	HGNC Symbol	4	ADAM metallopeptidase with thrombospondin type 1 motif 3 [Source:HGNC Symbol;Acc:HGN	-2.968113
ENSG00000145214	1609	DGKQ	protein_coding	HGNC Symbol	5	diacylglycerol kinase theta [Source:HGNC Symbol;Acc:HGNC:2856]	-2.966489
ENSG00000110931	10645	CAMKK2	protein_coding	HGNC Symbol	16	calcium/calmodulin dependent protein kinase kinase 2 [Source:HGNC Symbol;Acc:HGNC:147	-2.965208
ENSG00000166432	84460	ZMAT1	protein_coding	HGNC Symbol	6	zinc finger matrin-type 1 [Source:HGNC Symbol;Acc:HGNC:29377]	-2.964954
ENSG00000168710	10768	AHCYL1	protein_coding	HGNC Symbol	6	adenosylhomocysteinase like 1 [Source:HGNC Symbol;Acc:HGNC:344]	-2.964769
ENSG00000174327	201232	SLC16A13	protein_coding	HGNC Symbol	2	solute carrier family 16 member 13 [Source:HGNC Symbol;Acc:HGNC:31037]	-2.964378
ENSG00000259570	NA	AC243562.1	unprocessed_pseudogene	Clone-based (Ensembl) gene	1	golgin subfamily A member 2-like (AGSK1) pseudogene	-2.963629
ENSG00000137841	5330	PLCB2	protein_coding	HGNC Symbol	15	phospholipase C beta 2 [Source:HGNC Symbol;Acc:HGNC:9055]	-2.963469
ENSG00000161509	2905	GRIN2C	protein_coding	HGNC Symbol	5	glutamate ionotropic receptor NMDA type subunit 2C [Source:HGNC Symbol;Acc:HGNC:4587	-2.963042
ENSG00000225483	NA	RPL22P4	processed_pseudogene	HGNC Symbol	1	ribosomal protein L22 pseudogene 4 [Source:HGNC Symbol;Acc:HGNC:36299]	-2.962788
ENSG00000278390	1.02E+08	AL354696.2	antisense	Clone-based (Ensembl) gene	4		-2.961551
ENSG00000149499	256364	EML3	protein_coding	HGNC Symbol	18	echinoderm microtubule associated protein like 3 [Source:HGNC Symbol;Acc:HGNC:26666]	-2.961505
ENSG00000235173	51236	HGH1	protein_coding	HGNC Symbol	7	HGH1 homolog [Source:HGNC Symbol;Acc:HGNC:24161]	-2.961271
ENSG00000180616	6752	SSTR2	protein_coding	HGNC Symbol	2	somatostatin receptor 2 [Source:HGNC Symbol;Acc:HGNC:11331]	-2.960814
ENSG00000107331	20	ABCA2	protein_coding	HGNC Symbol	24	ATP binding cassette subfamily A member 2 [Source:HGNC Symbol;Acc:HGNC:32]	-2.960414
ENSG00000144115	55258	THNSL2	protein_coding	HGNC Symbol	10	threonine synthase like 2 [Source:HGNC Symbol;Acc:HGNC:25602]	-2.959645
ENSG00000183833	89876	MAATS1	protein_coding	HGNC Symbol	16	MYCBP associated and testis expressed 1 [Source:HGNC Symbol;Acc:HGNC:24010]	-2.957777
ENSG00000198298	220992	ZNF485	protein_coding	HGNC Symbol	4	zinc finger protein 485 [Source:HGNC Symbol;Acc:HGNC:23440]	-2.957241
ENSG00000144908	10840	ALDH1L1	protein_coding	HGNC Symbol	18	aldehyde dehydrogenase 1 family member L1 [Source:HGNC Symbol;Acc:HGNC:3978]	-2.956851
ENSG00000278817	1.03E+08	AC007325.4	protein_coding	Clone-based (Ensembl) gene	1	protein DGCR6 [Source:NCBI gene;Acc:102724770]	-2.955923
ENSG00000186806	147645	VSIG10L	protein_coding	HGNC Symbol	2	V-set and immunoglobulin domain containing 10 like [Source:HGNC Symbol;Acc:HGNC:27111	-2.953719
ENSG00000158286	388591	RNF207	protein_coding	HGNC Symbol	9	ring finger protein 207 [Source:HGNC Symbol;Acc:HGNC:32947]	-2.953
ENSG00000185189	340371	NRBP2	protein_coding	HGNC Symbol	10	nuclear receptor binding protein 2 [Source:HGNC Symbol;Acc:HGNC:19339]	-2.950775
ENSG00000166183	374569	ASPG	protein_coding	HGNC Symbol	9	asparaginase [Source:HGNC Symbol;Acc:HGNC:20123]	-2.949467
ENSG00000149294	4684	NCAM1	protein_coding	HGNC Symbol	30	neural cell adhesion molecule 1 [Source:HGNC Symbol;Acc:HGNC:7656]	-2.947407
ENSG00000128335	23780	APOL2	protein_coding	HGNC Symbol	9	apolipoprotein L2 [Source:HGNC Symbol;Acc:HGNC:619]	-2.945135
ENSG00000246465	NA	AC138904.1	lincRNA	Clone-based (Ensembl) gene	1	novel transcript LOC100506705	-2.943919

ENSG00000138400	130752	MDH1B	protein_coding	HGNC Symbol	6	malate dehydrogenase 1B [Source:HGNC Symbol;Acc:HGNC:17836]	-2.942864
ENSG00000170899	2941	GSTA4	protein_coding	HGNC Symbol	6	glutathione S-transferase alpha 4 [Source:HGNC Symbol;Acc:HGNC:4629]	-2.941234
ENSG00000008438	8993	PGLYRP1	protein_coding	HGNC Symbol	1	peptidoglycan recognition protein 1 [Source:HGNC Symbol;Acc:HGNC:8904]	-2.940555
ENSG00000187091	5333	PLCD1	protein_coding	HGNC Symbol	9	phospholipase C delta 1 [Source:HGNC Symbol;Acc:HGNC:9060]	-2.940228
ENSG00000163958	131540	ZDHHC19	protein_coding	HGNC Symbol	7	zinc finger DHHC-type containing 19 [Source:HGNC Symbol;Acc:HGNC:20713]	-2.938634
ENSG00000184949	646851	FAM227A	protein_coding	HGNC Symbol	11	family with sequence similarity 227 member A [Source:HGNC Symbol;Acc:HGNC:44197]	-2.93836
ENSG00000116205	127428	TCEANC2	protein_coding	HGNC Symbol	5	transcription elongation factor A N-terminal and central domain containing 2 [Source:HGNC S	-2.937531
ENSG00000262585	1.02E+08	LINC01979	lincRNA	HGNC Symbol	1	long intergenic non-protein coding RNA 1979 [Source:HGNC Symbol;Acc:HGNC:52807]	-2.93722
ENSG00000091622	83394	PITPNM3	protein_coding	HGNC Symbol	5	PITPNM family member 3 [Source:HGNC Symbol;Acc:HGNC:21043]	-2.935585
ENSG00000186862	79955	PDZD7	protein_coding	HGNC Symbol	10	PDZ domain containing 7 [Source:HGNC Symbol;Acc:HGNC:26257]	-2.933931
ENSG00000162882	23498	HAAO	protein_coding	HGNC Symbol	7	3-hydroxyanthranilate 3,4-dioxygenase [Source:HGNC Symbol;Acc:HGNC:4796]	-2.932396
ENSG00000234869	NA	AL021392.1	antisense	Clone-based (Ensembl) gene	1	novel transcript	-2.931448
ENSG00000107819	81855	SFXN3	protein_coding	HGNC Symbol	7	sideroflexin 3 [Source:HGNC Symbol;Acc:HGNC:16087]	-2.929756
ENSG00000161558	55260	TMEM143	protein_coding	HGNC Symbol	13	transmembrane protein 143 [Source:HGNC Symbol;Acc:HGNC:25603]	-2.92828
ENSG00000169884	7480	WNT10B	protein_coding	HGNC Symbol	6	Wnt family member 10B [Source:HGNC Symbol;Acc:HGNC:12775]	-2.924722
ENSG00000141639	5596	MAPK4	protein_coding	HGNC Symbol	6	mitogen-activated protein kinase 4 [Source:HGNC Symbol;Acc:HGNC:6878]	-2.924027
ENSG00000177694	254827	NAALADL2	protein_coding	HGNC Symbol	12	N-acetylated alpha-linked acidic dipeptidase like 2 [Source:HGNC Symbol;Acc:HGNC:23219]	-2.923742
ENSG00000159713	51673	TPPP3	protein_coding	HGNC Symbol	5	tubulin polymerization promoting protein family member 3 [Source:HGNC Symbol;Acc:HGNC:	-2.923131
ENSG00000148218	210	ALAD	protein_coding	HGNC Symbol	9	aminolevulinate dehydratase [Source:HGNC Symbol;Acc:HGNC:395]	-2.921096
ENSG00000128833	55930	MYO5C	protein_coding	HGNC Symbol	9	myosin VC [Source:HGNC Symbol;Acc:HGNC:7604]	-2.919091
ENSG00000275023	4302	MLLT6	protein_coding	HGNC Symbol	11	MLLT6, PHD finger containing [Source:HGNC Symbol;Acc:HGNC:7138]	-2.91896
ENSG00000100307	23492	CBX7	protein_coding	HGNC Symbol	7	chromobox 7 [Source:HGNC Symbol;Acc:HGNC:1557]	-2.918234
ENSG00000167771	283248	RCOR2	protein_coding	HGNC Symbol	3	REST corepressor 2 [Source:HGNC Symbol;Acc:HGNC:27455]	-2.918104
ENSG00000161395	93210	PGAP3	protein_coding	HGNC Symbol	11	post-GPI attachment to proteins 3 [Source:HGNC Symbol;Acc:HGNC:23719]	-2.917736
ENSG00000122367	11155	LDB3	protein_coding	HGNC Symbol	9	LIM domain binding 3 [Source:HGNC Symbol;Acc:HGNC:15710]	-2.91706
ENSG00000101417	11264	PXMP4	protein_coding	HGNC Symbol	3	peroxisomal membrane protein 4 [Source:HGNC Symbol;Acc:HGNC:15920]	-2.916939
ENSG00000071246	22846	VASH1	protein_coding	HGNC Symbol	5	vasohibin 1 [Source:HGNC Symbol;Acc:HGNC:19964]	-2.91585
ENSG00000237276	NA	ANO7L1	transcribed_unprocessed_pseudogene	HGNC Symbol	3	anoctamin 7 like 1 [Source:HGNC Symbol;Acc:HGNC:32248]	-2.913904
ENSG00000260693	NA	AC026150.1	lincRNA	Clone-based (Ensembl) gene	1	novel transcript	-2.913791
ENSG00000275714	8350	HIST1H3A	protein_coding	HGNC Symbol	1	histone cluster 1 H3 family member a [Source:HGNC Symbol;Acc:HGNC:4766]	-2.911683
ENSG00000165140	2203	FBP1	protein_coding	HGNC Symbol	4	fructose-bisphosphatase 1 [Source:HGNC Symbol;Acc:HGNC:3606]	-2.910553
ENSG00000094914	8086	AAAS	protein_coding	HGNC Symbol	20	aladin WD repeat nucleoporin [Source:HGNC Symbol;Acc:HGNC:13666]	-2.907372
ENSG00000271270	1.01E+08	TMCC1-AS1	antisense	HGNC Symbol	4	TMCC1 antisense RNA 1 (head to head) [Source:HGNC Symbol;Acc:HGNC:49060]	-2.905973
ENSG00000184988	113277	TMEM106A	protein_coding	HGNC Symbol	8	transmembrane protein 106A [Source:HGNC Symbol;Acc:HGNC:28288]	-2.904607
ENSG00000178035	3615	IMPDH2	protein_coding	HGNC Symbol	12	inosine monophosphate dehydrogenase 2 [Source:HGNC Symbol;Acc:HGNC:6053]	-2.903333
ENSG00000120899	2185	PTK2B	protein_coding	HGNC Symbol	17	protein tyrosine kinase 2 beta [Source:HGNC Symbol;Acc:HGNC:9612]	-2.901654
ENSG00000249601	NA	LINC01187	lincRNA	HGNC Symbol	2	long intergenic non-protein coding RNA 1187 [Source:HGNC Symbol;Acc:HGNC:49575]	-2.898233
ENSG00000262580	NA	AC087741.1	antisense	Clone-based (Ensembl) gene	5	novel transcript, antisense to CARD14	-2.897083
ENSG00000203808	154442	BVES-AS1	antisense	HGNC Symbol	5	BVES antisense RNA 1 [Source:HGNC Symbol;Acc:HGNC:21223]	-2.895895
ENSG00000248508	1E+08	SRP14-AS1	lincRNA	HGNC Symbol	3	SRP14 antisense RNA1 (head to head) [Source:HGNC Symbol;Acc:HGNC:48619]	-2.895579
ENSG00000186377	260293	CYP4X1	protein_coding	HGNC Symbol	2	cytochrome P450 family 4 subfamily X member 1 [Source:HGNC Symbol;Acc:HGNC:20244]	-2.895252
ENSG00000226321	728763	CROCC2	protein_coding	HGNC Symbol	3	ciliary rootlet coiled-coil, rootletin family member 2 [Source:HGNC Symbol;Acc:HGNC:51677]	-2.894156
ENSG00000049283	55040	EPN3	protein_coding	HGNC Symbol	18	epsin 3 [Source:HGNC Symbol;Acc:HGNC:18235]	-2.890312
ENSG00000068831	10235	RASGRP2	protein_coding	HGNC Symbol	23	RAS guanyl releasing protein 2 [Source:HGNC Symbol;Acc:HGNC:9879]	-2.888882
ENSG00000109066	54868	TMEM104	protein_coding	HGNC Symbol	7	transmembrane protein 104 [Source:HGNC Symbol;Acc:HGNC:25984]	-2.888427
ENSG00000071539	9319	TRIP13	protein_coding	HGNC Symbol	7	thyroid hormone receptor interactor 13 [Source:HGNC Symbol;Acc:HGNC:12307]	-2.887769
ENSG00000171219	55561	CDC42BPG	protein_coding	HGNC Symbol	4	CDC42 binding protein kinase gamma [Source:HGNC Symbol;Acc:HGNC:29829]	-2.886893
ENSG00000115257	54760	PCSK4	protein_coding	HGNC Symbol	13	proprotein convertase subtilisin/kexin type 4 [Source:HGNC Symbol;Acc:HGNC:8746]	-2.884147
ENSG00000141569	201292	TRIM65	protein_coding	HGNC Symbol	7	tripartite motif containing 65 [Source:HGNC Symbol;Acc:HGNC:27316]	-2.88118
ENSG00000168404	197259	MLKL	protein_coding	HGNC Symbol	8	mixed lineage kinase domain like pseudokinase [Source:HGNC Symbol;Acc:HGNC:26617]	-2.880997
ENSG00000204536	54535	CCHCR1	protein_coding	HGNC Symbol	30	coiled-coil alpha-helical rod protein 1 [Source:HGNC Symbol;Acc:HGNC:13930]	-2.880691
ENSG00000114853	92999	ZBTB47	protein_coding	HGNC Symbol	2	zinc finger and BTB domain containing 47 [Source:HGNC Symbol;Acc:HGNC:26955]	-2.879964
ENSG00000235949	NA	AC061961.1	antisense	Clone-based (Ensembl) gene	1	novel transcript	-2.879231
ENSG00000163517	79885	HDAC11	protein_coding	HGNC Symbol	21	histone deacetylase 11 [Source:HGNC Symbol;Acc:HGNC:19086]	-2.879156
ENSG00000181634	9966	TNFSF15	protein_coding	HGNC Symbol	2	TNF superfamily member 15 [Source:HGNC Symbol;Acc:HGNC:11931]	-2.87869
ENSG00000131187	2161	F12	protein_coding	HGNC Symbol	6	coagulation factor XII [Source:HGNC Symbol;Acc:HGNC:3530]	-2.878562
ENSG00000230555	NA	AL450326.1	lincRNA	Clone-based (Ensembl) gene	1	novel transcript	-2.87678
ENSG00000128928	3712	IVD	protein_coding	HGNC Symbol	13	isovaleryl-CoA dehydrogenase [Source:HGNC Symbol;Acc:HGNC:6186]	-2.875202
ENSG00000230392	1.02E+08	AC004835.1	lincRNA	Clone-based (Ensembl) gene	1		-2.874677
ENSG00000091536	51168	MYO15A	protein_coding	HGNC Symbol	23	myosin XVA [Source:HGNC Symbol;Acc:HGNC:7594]	-2.874524
ENSG00000166960	374864	CCDC178	protein_coding	HGNC Symbol	13	coiled-coil domain containing 178 [Source:HGNC Symbol;Acc:HGNC:29588]	-2.872542
ENSG00000165072	256691	MAMDC2	protein_coding	HGNC Symbol	2	MAM domain containing 2 [Source:HGNC Symbol;Acc:HGNC:23673]	-2.870261
ENSG00000076864	5909	RAP1GAP	protein_coding	HGNC Symbol	13	RAP1 GTPase activating protein [Source:HGNC Symbol;Acc:HGNC:9858]	-2.86935
ENSG00000131398	3748	KCNC3	protein_coding	HGNC Symbol	3	potassium voltage-gated channel subfamily C member 3 [Source:HGNC Symbol;Acc:HGNC:62	-2.868548
ENSG00000256433	NA	AC005840.2	lincRNA	Clone-based (Ensembl) gene	1	novel transcript	-2.868355

ENSG00000127585	146330	FBXL16	protein_coding	HGNC Symbol	5	F-box and leucine rich repeat protein 16 [Source:HGNC Symbol;Acc:HGNC:14150]	-2.866545
ENSG00000132874	8170	SLC14A2	protein_coding	HGNC Symbol	3	solute carrier family 14 member 2 [Source:HGNC Symbol;Acc:HGNC:10919]	-2.865417
ENSG00000223783	NA	LINC01983	lincRNA	HGNC Symbol	3	long intergenic non-protein coding RNA 1983 [Source:HGNC Symbol;Acc:HGNC:52813]	-2.862084
ENSG00000064195	1747	DLX3	protein_coding	HGNC Symbol	2	distal-less homeobox 3 [Source:HGNC Symbol;Acc:HGNC:2916]	-2.860358
ENSG00000178078	55620	STAP2	protein_coding	HGNC Symbol	10	signal transducing adaptor family member 2 [Source:HGNC Symbol;Acc:HGNC:30430]	-2.860036
ENSG00000142798	3339	HSPG2	protein_coding	HGNC Symbol	15	heparan sulfate proteoglycan 2 [Source:HGNC Symbol;Acc:HGNC:5273]	-2.85551
ENSG00000155980	3798	KIF5A	protein_coding	HGNC Symbol	3	kinesin family member 5A [Source:HGNC Symbol;Acc:HGNC:6323]	-2.852007
ENSG00000130590	140700	SAMD10	protein_coding	HGNC Symbol	4	sterile alpha motif domain containing 10 [Source:HGNC Symbol;Acc:HGNC:16129]	-2.851699
ENSG00000138316	140766	ADAMTS14	protein_coding	HGNC Symbol	2	ADAM metallopeptidase with thrombospondin type 1 motif 14 [Source:HGNC Symbol;Acc:HG	-2.851376
ENSG00000225889	NA	AC012368.1	antisense	Clone-based (Ensembl) gene	6		-2.848137
ENSG00000214654	NA	B3GNT10	protein_coding	HGNC Symbol	3	UDP-GlcNAc:betaGal beta-1,3-N-acetylglucosaminyltransferase 10 (putative) [Source:HGNC Sy	-2.848107
ENSG00000177494	79413	ZBED2	protein_coding	HGNC Symbol	1	zinc finger BED-type containing 2 [Source:HGNC Symbol;Acc:HGNC:20710]	-2.845805
ENSG00000169733	5986	RFNG	protein_coding	HGNC Symbol	12	RFNG O-fucosylpeptide 3-beta-N-acetylglucosaminyltransferase [Source:HGNC Symbol;Acc:H(	-2.845659
ENSG00000169750	5881	RAC3	protein_coding	HGNC Symbol	4	Rac family small GTPase 3 [Source:HGNC Symbol;Acc:HGNC:9803]	-2.845534
ENSG00000240771	115557	ARHGEF25	protein_coding	HGNC Symbol	8	Rho guanine nucleotide exchange factor 25 [Source:HGNC Symbol;Acc:HGNC:30275]	-2.844799
ENSG00000224189	401022	HAGLR	antisense	HGNC Symbol	16	HOXD antisense growth-associated long non-coding RNA [Source:HGNC Symbol;Acc:HGNC:43	-2.843504
ENSG00000171119	4902	NRTN	protein_coding	HGNC Symbol	1	neurturin [Source:HGNC Symbol;Acc:HGNC:8007]	-2.842816
ENSG00000230074	NA	AL162231.2	antisense	Clone-based (Ensembl) gene	1	novel transcript	-2.842212
ENSG00000256771	56242	ZNF253	protein_coding	HGNC Symbol	5	zinc finger protein 253 [Source:HGNC Symbol;Acc:HGNC:13497]	-2.841428
ENSG00000181577	221416	C6orf223	protein_coding	HGNC Symbol	4	chromosome 6 open reading frame 223 [Source:HGNC Symbol;Acc:HGNC:28692]	-2.840885
ENSG00000233823	NA	AL356311.1	antisense	Clone-based (Ensembl) gene	1	novel transcript	-2.840522
ENSG00000137868	64220	STRA6	protein_coding	HGNC Symbol	22	stimulated by retinoic acid 6 [Source:HGNC Symbol;Acc:HGNC:30650]	-2.839438
ENSG00000198919	9666	DZIP3	protein_coding	HGNC Symbol	6	DAZ interacting zinc finger protein 3 [Source:HGNC Symbol;Acc:HGNC:30938]	-2.839391
ENSG00000163820	79443	FYCO1	protein_coding	HGNC Symbol	5	FYVE and coiled-coil domain containing 1 [Source:HGNC Symbol;Acc:HGNC:14673]	-2.835851
ENSG00000196155	25894	PLEKHG4	protein_coding	HGNC Symbol	15	pleckstrin homology and RhoGEF domain containing G4 [Source:HGNC Symbol;Acc:HGNC:245	-2.83512
ENSG00000154556	8470	SORBS2	protein_coding	HGNC Symbol	65	sorbin and SH3 domain containing 2 [Source:HGNC Symbol;Acc:HGNC:24098]	-2.834837
ENSG00000102048	140462	ASB9	protein_coding	HGNC Symbol	9	ankyrin repeat and SOCS box containing 9 [Source:HGNC Symbol;Acc:HGNC:17184]	-2.833655
ENSG00000025434	10062	NR1H3	protein_coding	HGNC Symbol	35	nuclear receptor subfamily 1 group H member 3 [Source:HGNC Symbol;Acc:HGNC:7966]	-2.831354
ENSG00000137628	55601	DDX60	protein_coding	HGNC Symbol	6	DExD/H-box helicase 60 [Source:HGNC Symbol;Acc:HGNC:25942]	-2.831321
ENSG00000169710	2194	FASN	protein_coding	HGNC Symbol	13	fatty acid synthase [Source:HGNC Symbol;Acc:HGNC:3594]	-2.83059
ENSG00000115604	8809	IL18R1	protein_coding	HGNC Symbol	5	interleukin 18 receptor 1 [Source:HGNC Symbol;Acc:HGNC:5988]	-2.829614
ENSG00000187017	83715	ESPN	protein_coding	HGNC Symbol	18	espin [Source:HGNC Symbol;Acc:HGNC:13281]	-2.827555
ENSG00000185924	146760	RTN4RL1	protein_coding	HGNC Symbol	1	reticulon 4 receptor like 1 [Source:HGNC Symbol;Acc:HGNC:21329]	-2.826613
ENSG00000184908	1188	CLCNKB	protein_coding	HGNC Symbol	4	chloride voltage-gated channel Kb [Source:HGNC Symbol;Acc:HGNC:2027]	-2.826591
ENSG00000146409	116843	SLC18B1	protein_coding	HGNC Symbol	7	solute carrier family 18 member B1 [Source:HGNC Symbol;Acc:HGNC:21573]	-2.826397
ENSG00000166750	162394	SLFN5	protein_coding	HGNC Symbol	3	schlafen family member 5 [Source:HGNC Symbol;Acc:HGNC:28286]	-2.825686
ENSG00000229375	NA	USP24P1	unprocessed_pseudogene	HGNC Symbol	1	ubiquitin specific peptidase 24 pseudogene 1 [Source:HGNC Symbol;Acc:HGNC:42014]	-2.825537
ENSG00000273607	400916	CHCHD10	protein_coding	HGNC Symbol	5	coiled-coil-helix-coiled-coil-helix domain containing 10 [Source:HGNC Symbol;Acc:HGNC:1555	-2.824652
ENSG00000103196	83716	CRISPLD2	protein_coding	HGNC Symbol	11	cysteine rich secretory protein LCCL domain containing 2 [Source:HGNC Symbol;Acc:HGNC:25	-2.823989
ENSG00000249115	23354	HAUS5	protein_coding	HGNC Symbol	9	HAUS augmin like complex subunit 5 [Source:HGNC Symbol;Acc:HGNC:29130]	-2.823637
ENSG00000141576	114804	RNF157	protein_coding	HGNC Symbol	10	ring finger protein 157 [Source:HGNC Symbol;Acc:HGNC:29402]	-2.82307
ENSG00000234196	221527	ZBTB12	protein_coding	HGNC Symbol	1	zinc finger and BTB domain containing 12 [Source:HGNC Symbol;Acc:HGNC:19066]	-2.822922
ENSG00000197183	140688	NOL4L	protein_coding	HGNC Symbol	11	nucleolar protein 4 like [Source:HGNC Symbol;Acc:HGNC:16106]	-2.81937
ENSG00000182796	440104	TMEM198B	transcribed_unitary_pseudogene	HGNC Symbol	15	transmembrane protein 198B (pseudogene) [Source:HGNC Symbol;Acc:HGNC:43629]	-2.819296
ENSG00000277157	8360	HIST1H4D	protein_coding	HGNC Symbol	1	histone cluster 1 H4 family member d [Source:HGNC Symbol;Acc:HGNC:4782]	-2.819123
ENSG00000175938	93129	ORAI3	protein_coding	HGNC Symbol	4	ORAI calcium release-activated calcium modulator 3 [Source:HGNC Symbol;Acc:HGNC:28185]	-2.818613
ENSG00000255346	79400	NOX5	protein_coding	HGNC Symbol	9	NADPH oxidase 5 [Source:HGNC Symbol;Acc:HGNC:14874]	-2.81846
ENSG00000164877	79778	MICALL2	protein_coding	HGNC Symbol	15	MICAL like 2 [Source:HGNC Symbol;Acc:HGNC:29672]	-2.817137
ENSG00000196961	160	AP2A1	protein_coding	HGNC Symbol	8	adaptor related protein complex 2 subunit alpha 1 [Source:HGNC Symbol;Acc:HGNC:561]	-2.816544
ENSG00000148832	196743	PAOX	protein_coding	HGNC Symbol	10	polyamine oxidase [Source:HGNC Symbol;Acc:HGNC:20837]	-2.81636
ENSG00000126603	84662	GLIS2	protein_coding	HGNC Symbol	2	GLIS family zinc finger 2 [Source:HGNC Symbol;Acc:HGNC:29450]	-2.81415
ENSG00000157214	261729	STEAP2	protein_coding	HGNC Symbol	10	STEAP2 metalloreductase [Source:HGNC Symbol;Acc:HGNC:17885]	-2.813428
ENSG00000172508	57571	CARNS1	protein_coding	HGNC Symbol	7	carnosine synthase 1 [Source:HGNC Symbol;Acc:HGNC:29268]	-2.812156
ENSG00000170915	85315	PAQR8	protein_coding	HGNC Symbol	3	progestin and adipoQ receptor family member 8 [Source:HGNC Symbol;Acc:HGNC:15708]	-2.811399
ENSG00000273045	150590	C2orf15	protein_coding	HGNC Symbol	4	chromosome 2 open reading frame 15 [Source:HGNC Symbol;Acc:HGNC:28436]	-2.809141
ENSG00000086205	2346	FOLH1	protein_coding	HGNC Symbol	13	folate hydrolase 1 [Source:HGNC Symbol;Acc:HGNC:3788]	-2.807391
ENSG00000130052	9754	STARD8	protein_coding	HGNC Symbol	5	StAR related lipid transfer domain containing 8 [Source:HGNC Symbol;Acc:HGNC:19161]	-2.807107
ENSG00000131747	7153	TOP2A	protein_coding	HGNC Symbol	5	DNA topoisomerase II alpha [Source:HGNC Symbol;Acc:HGNC:11989]	-2.806949
ENSG00000144843	141	ADPRH	protein_coding	HGNC Symbol	7	ADP-ribosylarginine hydrolase [Source:HGNC Symbol;Acc:HGNC:269]	-2.806656
ENSG00000100191	6527	SLC5A4	protein_coding	HGNC Symbol	1	solute carrier family 5 member 4 [Source:HGNC Symbol;Acc:HGNC:11039]	-2.805553
ENSG00000081138	1005	CDH7	protein_coding	HGNC Symbol	4	cadherin 7 [Source:HGNC Symbol;Acc:HGNC:1766]	-2.805282
ENSG00000158292	387509	GPR153	protein_coding	HGNC Symbol	1	G protein-coupled receptor 153 [Source:HGNC Symbol;Acc:HGNC:23618]	-2.803034
ENSG00000215256	55449	DHRS4-AS1	antisense	HGNC Symbol	6	DHRS4 antisense RNA 1 [Source:HGNC Symbol;Acc:HGNC:23175]	-2.802637
ENSG00000168067	5871	MAP4K2	protein_coding	HGNC Symbol	13	mitogen-activated protein kinase kinase kinase kinase 2 [Source:HGNC Symbol;Acc:HGNC:686	-2.801944
ENSG00000228146	197350	CASP16P	transcribed_unitary_pseudogene	HGNC Symbol	4	caspase 16, pseudogene [Source:HGNC Symbol;Acc:HGNC:27290]	-2.801813

ENSG00000165309	219681	ARMC3	protein_coding	HGNC Symbol	13	armadillo repeat containing 3 [Source:HGNC Symbol;Acc:HGNC:30964]	-2.79974
ENSG00000167680	10501	SEMA6B	protein_coding	HGNC Symbol	3	semaphorin 6B [Source:HGNC Symbol;Acc:HGNC:10739]	-2.799032
ENSG00000146776	222255	ATXN7L1	protein_coding	HGNC Symbol	11	ataxin 7 like 1 [Source:HGNC Symbol;Acc:HGNC:22210]	-2.796972
ENSG00000065054	9351	SLC9A3R2	protein_coding	HGNC Symbol	9	SLC9A3 regulator 2 [Source:HGNC Symbol;Acc:HGNC:11076]	-2.796703
ENSG00000145362	287	ANK2	protein_coding	HGNC Symbol	27	ankyrin 2 [Source:HGNC Symbol;Acc:HGNC:493]	-2.796345
ENSG00000143416	8991	SELENBP1	protein_coding	HGNC Symbol	19	selenium binding protein 1 [Source:HGNC Symbol;Acc:HGNC:10719]	-2.796041
ENSG00000205978	57523	NYNRIN	protein_coding	HGNC Symbol	2	NYN domain and retroviral integrase containing [Source:HGNC Symbol;Acc:HGNC:20165]	-2.795591
ENSG00000175463	374403	TBC1D10C	protein_coding	HGNC Symbol	9	TBC1 domain family member 10C [Source:HGNC Symbol;Acc:HGNC:24702]	-2.794636
ENSG00000165171	155368	METTL27	protein_coding	HGNC Symbol	3	methyltransferase like 27 [Source:HGNC Symbol;Acc:HGNC:19068]	-2.793697
ENSG00000124493	2914	GRM4	protein_coding	HGNC Symbol	14	glutamate metabotropic receptor 4 [Source:HGNC Symbol;Acc:HGNC:4596]	-2.792803
ENSG00000144152	129804	FBLN7	protein_coding	HGNC Symbol	7	fibulin 7 [Source:HGNC Symbol;Acc:HGNC:26740]	-2.791621
ENSG00000219626	375190	FAM228B	protein_coding	HGNC Symbol	10	family with sequence similarity 228 member B [Source:HGNC Symbol;Acc:HGNC:24736]	-2.791378
ENSG00000176055	153364	MBLAC2	protein_coding	HGNC Symbol	2	metallo-beta-lactamase domain containing 2 [Source:HGNC Symbol;Acc:HGNC:33711]	-2.791292
ENSG00000134030	9811	CTIF	protein_coding	HGNC Symbol	11	cap binding complex dependent translation initiation factor [Source:HGNC Symbol;Acc:HGNC	-2.791239
ENSG00000230415	NA	LINC01786	lincRNA	HGNC Symbol	2	long intergenic non-protein coding RNA 1786 [Source:HGNC Symbol;Acc:HGNC:52575]	-2.787707
ENSG00000215440	79716	NPEPL1	protein_coding	HGNC Symbol	9	aminopeptidase like 1 [Source:HGNC Symbol;Acc:HGNC:16244]	-2.787129
ENSG00000047644	55841	WWC3	protein_coding	HGNC Symbol	2	WWC family member 3 [Source:HGNC Symbol;Acc:HGNC:29237]	-2.786636
ENSG00000169683	201255	LRRC45	protein_coding	HGNC Symbol	6	leucine rich repeat containing 45 [Source:HGNC Symbol;Acc:HGNC:28302]	-2.786208
ENSG00000244617	151516	ASPRV1	protein_coding	HGNC Symbol	1	aspartic peptidase retroviral like 1 [Source:HGNC Symbol;Acc:HGNC:26321]	-2.785496
ENSG00000154237	79705	LRRK1	protein_coding	HGNC Symbol	11	leucine rich repeat kinase 1 [Source:HGNC Symbol;Acc:HGNC:18608]	-2.783995
ENSG00000074370	489	ATP2A3	protein_coding	HGNC Symbol	15	ATPase sarcoplasmic/endoplasmic reticulum Ca2+ transporting 3 [Source:HGNC Symbol;Acc:H	-2.783277
ENSG00000127946	3092	HIP1	protein_coding	HGNC Symbol	7	huntingtin interacting protein 1 [Source:HGNC Symbol;Acc:HGNC:4913]	-2.78318
ENSG00000185745	3434	IFIT1	protein_coding	HGNC Symbol	2	interferon induced protein with tetratricopeptide repeats 1 [Source:HGNC Symbol;Acc:HGNC:	-2.782938
ENSG00000184702	5413	5-Sep	protein_coding	HGNC Symbol	13	septin 5 [Source:HGNC Symbol;Acc:HGNC:9164]	-2.782902
ENSG00000250510	27239	GPR162	protein_coding	HGNC Symbol	7	G protein-coupled receptor 162 [Source:HGNC Symbol;Acc:HGNC:16693]	-2.781948
ENSG00000188322	388228	SBK1	protein_coding	HGNC Symbol	1	SH3 domain binding kinase 1 [Source:HGNC Symbol;Acc:HGNC:17699]	-2.781573
ENSG00000102383	158866	ZDHHC15	protein_coding	HGNC Symbol	3	zinc finger DHHC-type containing 15 [Source:HGNC Symbol;Acc:HGNC:20342]	-2.780408
ENSG00000119411	54836	BSPRY	protein_coding	HGNC Symbol	2	B-box and SPRY domain containing [Source:HGNC Symbol;Acc:HGNC:18232]	-2.780119
ENSG00000117245	57576	KIF17	protein_coding	HGNC Symbol	9	kinesin family member 17 [Source:HGNC Symbol;Acc:HGNC:19167]	-2.779096
ENSG00000134245	7482	WNT2B	protein_coding	HGNC Symbol	4	Wnt family member 2B [Source:HGNC Symbol;Acc:HGNC:12781]	-2.778467
ENSG00000196502	6817	SULT1A1	protein_coding	HGNC Symbol	11	sulfotransferase family 1A member 1 [Source:HGNC Symbol;Acc:HGNC:11453]	-2.777729
ENSG00000068024	9759	HDAC4	protein_coding	HGNC Symbol	17	histone deacetylase 4 [Source:HGNC Symbol;Acc:HGNC:14063]	-2.777068
ENSG00000106976	1759	DNM1	protein_coding	HGNC Symbol	29	dynamin 1 [Source:HGNC Symbol;Acc:HGNC:2972]	-2.776053
ENSG00000177706	56975	FAM20C	protein_coding	HGNC Symbol	5	FAM20C, golgi associated secretory pathway kinase [Source:HGNC Symbol;Acc:HGNC:22140]	-2.775933
ENSG00000130701	140893	RBBP8NL	protein_coding	HGNC Symbol	1	RBBP8 N-terminal like [Source:HGNC Symbol;Acc:HGNC:16144]	-2.774742
ENSG00000177640	255082	CASC2	antisense	HGNC Symbol	8	cancer susceptibility 2 [Source:HGNC Symbol;Acc:HGNC:22933]	-2.774719
ENSG00000250067	374887	YJEFN3	protein_coding	HGNC Symbol	3	YjeF N-terminal domain containing 3 [Source:HGNC Symbol;Acc:HGNC:24785]	-2.773458
ENSG00000185100	122622	ADSSL1	protein_coding	HGNC Symbol	12	adenylosuccinate synthase like 1 [Source:HGNC Symbol;Acc:HGNC:20093]	-2.772053
ENSG00000184985	57537	SORCS2	protein_coding	HGNC Symbol	4	sortilin related VPS10 domain containing receptor 2 [Source:HGNC Symbol;Acc:HGNC:16698]	-2.771899
ENSG00000185187	59307	SIGIRR	protein_coding	HGNC Symbol	22	single Ig and TIR domain containing [Source:HGNC Symbol;Acc:HGNC:30575]	-2.77126
ENSG00000100077	157	GRK3	protein_coding	HGNC Symbol	3	G protein-coupled receptor kinase 3 [Source:HGNC Symbol;Acc:HGNC:290]	-2.770049
ENSG00000271254	1.03E+08	AC240274.1	protein_coding	Clone-based (Ensembl) gene	4	neuroblastoma breakpoint family member 1 [Source:NCBI gene;Acc:102724250]	-2.769518
ENSG00000223813	NA	AC007255.1	antisense	Clone-based (Ensembl) gene	2	novel transcript, antisense to CHN2	-2.7688
ENSG00000171792	83695	RHNO1	protein_coding	HGNC Symbol	10	RAD9-HUS1-RAD1 interacting nuclear orphan 1 [Source:HGNC Symbol;Acc:HGNC:28206]	-2.768583
ENSG00000187955	7373	COL14A1	protein_coding	HGNC Symbol	9	collagen type XIV alpha 1 chain [Source:HGNC Symbol;Acc:HGNC:2191]	-2.768076
ENSG00000183117	64478	CSMD1	protein_coding	HGNC Symbol	18	CUB and Sushi multiple domains 1 [Source:HGNC Symbol;Acc:HGNC:14026]	-2.767783
ENSG00000224751	NA	SHMT1P1	processed_pseudogene	HGNC Symbol	1	serine hydroxymethyltransferase 1 (soluble) pseudogene 1 [Source:HGNC Symbol;Acc:HGNC::	-2.767167
ENSG00000227001	NA	NBPF2P	unprocessed_pseudogene	HGNC Symbol	1	NBPF member 2, pseudogene [Source:HGNC Symbol;Acc:HGNC:31987]	-2.766971
ENSG00000258608	NA	DNAJC19P9	processed_pseudogene	HGNC Symbol	1	DnaJ heat shock protein family (Hsp40) member C19 pseudogene 9 [Source:HGNC Symbol;Acc	-2.76585
ENSG00000164362	7015	TERT	protein_coding	HGNC Symbol	6	telomerase reverse transcriptase [Source:HGNC Symbol;Acc:HGNC:11730]	-2.764832
ENSG00000099365	112755	STX1B	protein_coding	HGNC Symbol	3	syntaxin 1B [Source:HGNC Symbol;Acc:HGNC:18539]	-2.764496
ENSG00000266928	NA	AC020905.1	lincRNA	Clone-based (Ensembl) gene	1	novel transcript	-2.761731
ENSG00000181409	9625	AATK	protein_coding	HGNC Symbol	11	apoptosis associated tyrosine kinase [Source:HGNC Symbol;Acc:HGNC:21]	-2.761694
ENSG00000130702	3911	LAMA5	protein_coding	HGNC Symbol	14	laminin subunit alpha 5 [Source:HGNC Symbol;Acc:HGNC:6485]	-2.760079
ENSG00000169231	7059	THBS3	protein_coding	HGNC Symbol	11	thrombospondin 3 [Source:HGNC Symbol;Acc:HGNC:11787]	-2.758191
ENSG00000166165	1152	CKB	protein_coding	HGNC Symbol	17	creatine kinase B [Source:HGNC Symbol;Acc:HGNC:1991]	-2.757467
ENSG00000168765	2948	GSTM4	protein_coding	HGNC Symbol	13	glutathione S-transferase mu 4 [Source:HGNC Symbol;Acc:HGNC:4636]	-2.756763
ENSG00000180264	347088	ADGRD2	protein_coding	HGNC Symbol	7	adhesion G protein-coupled receptor D2 [Source:HGNC Symbol;Acc:HGNC:18651]	-2.756402
ENSG00000088836	83959	SLC4A11	protein_coding	HGNC Symbol	13	solute carrier family 4 member 11 [Source:HGNC Symbol;Acc:HGNC:16438]	-2.755709
ENSG00000064961	10362	HMG20B	protein_coding	HGNC Symbol	16	high mobility group 20B [Source:HGNC Symbol;Acc:HGNC:5002]	-2.75503
ENSG00000175318	196996	GRAMD2A	protein_coding	HGNC Symbol	13	GRAM domain containing 2A [Source:HGNC Symbol;Acc:HGNC:27287]	-2.752408
ENSG00000261787	1E+08	TCF24	protein_coding	HGNC Symbol	2	transcription factor 24 [Source:HGNC Symbol;Acc:HGNC:32275]	-2.751286
ENSG00000163389	56983	POGLUT1	protein_coding	HGNC Symbol	9	protein O-glucosyltransferase 1 [Source:HGNC Symbol;Acc:HGNC:22954]	-2.749531
ENSG00000053371	8574	AKR7A2	protein_coding	HGNC Symbol	5	aldo-keto reductase family 7 member A2 [Source:HGNC Symbol;Acc:HGNC:389]	-2.747599
ENSG00000160221	8209	GATD3A	protein_coding	HGNC Symbol	15	glutamine amidotransferase like class 1 domain containing 3A [Source:HGNC Symbol;Acc:HGN	-2.74664

ENSG00000160221	1.03E+08	GATD3A	protein_coding	HGNC Symbol	15	glutamine amidotransferase like class 1 domain containing 3A [Source:HGNC Symbol;Acc:HGN	-2.74664
ENSG00000204923	554251	FBXO48	protein_coding	HGNC Symbol	1	F-box protein 48 [Source:HGNC Symbol;Acc:HGNC:33857]	-2.746294
ENSG00000089050	10741	RBBP9	protein_coding	HGNC Symbol	3	RB binding protein 9, serine hydrolase [Source:HGNC Symbol;Acc:HGNC:9892]	-2.745065
ENSG00000172296	55304	SPTLC3	protein_coding	HGNC Symbol	6	serine palmitoyltransferase long chain base subunit 3 [Source:HGNC Symbol;Acc:HGNC:16253	-2.744986
ENSG00000125247	84899	TMTC4	protein_coding	HGNC Symbol	11	transmembrane and tetratricopeptide repeat containing 4 [Source:HGNC Symbol;Acc:HGNC:2	-2.744946
ENSG00000187952	NA	HS6ST1P1	processed_pseudogene	HGNC Symbol	1	heparan sulfate 6-O-sulfotransferase 1 pseudogene 1 [Source:HGNC Symbol;Acc:HGNC:31835	-2.743538
ENSG00000235111	NA	Z97192.3	sense_intronic	Clone-based (Ensembl) gene	1	novel transcript	-2.742698
ENSG00000170439	196410	METTL7B	protein_coding	HGNC Symbol	2	methyltransferase like 7B [Source:HGNC Symbol;Acc:HGNC:28276]	-2.741966
ENSG00000164062	327	APEH	protein_coding	HGNC Symbol	16	acylaminoacyl-peptide hydrolase [Source:HGNC Symbol;Acc:HGNC:586]	-2.741118
ENSG00000134198	10100	TSPAN2	protein_coding	HGNC Symbol	4	tetraspanin 2 [Source:HGNC Symbol;Acc:HGNC:20659]	-2.740565
ENSG00000006534	221	ALDH3B1	protein_coding	HGNC Symbol	10	aldehyde dehydrogenase 3 family member B1 [Source:HGNC Symbol;Acc:HGNC:410]	-2.739894
ENSG00000147100	6567	SLC16A2	protein_coding	HGNC Symbol	3	solute carrier family 16 member 2 [Source:HGNC Symbol;Acc:HGNC:10923]	-2.739484
ENSG00000197818	23315	SLC9A8	protein_coding	HGNC Symbol	4	solute carrier family 9 member A8 [Source:HGNC Symbol;Acc:HGNC:20728]	-2.738975
ENSG00000163328	151556	GPR155	protein_coding	HGNC Symbol	6	G protein-coupled receptor 155 [Source:HGNC Symbol;Acc:HGNC:22951]	-2.738436
ENSG00000162482	22977	AKR7A3	protein_coding	HGNC Symbol	1	aldo-keto reductase family 7 member A3 [Source:HGNC Symbol;Acc:HGNC:390]	-2.737685
ENSG00000173153	2101	ESRRA	protein_coding	HGNC Symbol	7	estrogen related receptor alpha [Source:HGNC Symbol;Acc:HGNC:3471]	-2.737158
ENSG00000084774	790	CAD	protein_coding	HGNC Symbol	11	carbamoyl-phosphate synthetase 2, aspartate transcarbamylase, and dihydroorotase [Source:	-2.736993
ENSG00000165424	219654	ZCCHC24	protein_coding	HGNC Symbol	2	zinc finger CCHC-type containing 24 [Source:HGNC Symbol;Acc:HGNC:26911]	-2.736236
ENSG00000213025	NA	COX20P1	processed_pseudogene	HGNC Symbol	1	COX20, cytochrome c oxidase assembly factor pseudogene 1 [Source:HGNC Symbol;Acc:HGN(	-2.734152
ENSG00000196482	2104	ESRRG	protein_coding	HGNC Symbol	24	estrogen related receptor gamma [Source:HGNC Symbol;Acc:HGNC:3474]	-2.733755
ENSG00000183401	126075	CCDC159	protein_coding	HGNC Symbol	20	coiled-coil domain containing 159 [Source:HGNC Symbol;Acc:HGNC:26996]	-2.731476
ENSG00000115993	66008	TRAK2	protein_coding	HGNC Symbol	5	trafficking kinesin protein 2 [Source:HGNC Symbol;Acc:HGNC:13206]	-2.731429
ENSG00000230233	NA	AF240627.1	lincRNA	Clone-based (Ensembl) gene	1	novel transcript	-2.730841
ENSG00000228010	NA	AC073343.2	antisense	Clone-based (Ensembl) gene	3	novel transcript, antisense to ZNF12	-2.729436
ENSG00000233198	643596	RNF224	protein_coding	HGNC Symbol	1	ring finger protein 224 [Source:HGNC Symbol;Acc:HGNC:41912]	-2.72908
ENSG00000127419	84286	TMEM175	protein_coding	HGNC Symbol	22	transmembrane protein 175 [Source:HGNC Symbol;Acc:HGNC:28709]	-2.728646
ENSG00000013016	30845	EHD3	protein_coding	HGNC Symbol	1	EH domain containing 3 [Source:HGNC Symbol;Acc:HGNC:3244]	-2.728305
ENSG00000176387	3291	HSD11B2	protein_coding	HGNC Symbol	4	hydroxysteroid 11-beta dehydrogenase 2 [Source:HGNC Symbol;Acc:HGNC:5209]	-2.727345
ENSG00000175985	400224	PLEKHD1	protein_coding	HGNC Symbol	2	pleckstrin homology and coiled-coil domain containing D1 [Source:HGNC Symbol;Acc:HGNC:2	-2.726771
ENSG00000145029	84276	NICN1	protein_coding	HGNC Symbol	8	nicolin 1 [Source:HGNC Symbol;Acc:HGNC:18317]	-2.725596
ENSG00000157326	10901	DHRS4	protein_coding	HGNC Symbol	8	dehydrogenase/reductase 4 [Source:HGNC Symbol;Acc:HGNC:16985]	-2.724727
ENSG00000150433	219854	TMEM218	protein_coding	HGNC Symbol	17	transmembrane protein 218 [Source:HGNC Symbol;Acc:HGNC:27344]	-2.724712
ENSG00000163462	80128	TRIM46	protein_coding	HGNC Symbol	11	tripartite motif containing 46 [Source:HGNC Symbol;Acc:HGNC:19019]	-2.719865
ENSG00000247708	1.01E+08	STX18-AS1	antisense	HGNC Symbol	7	STX18 antisense RNA 1 (head to head) [Source:HGNC Symbol;Acc:HGNC:48877]	-2.7195
ENSG00000196123	653319	KIAA0895L	protein_coding	HGNC Symbol	12	KIAA0895 like [Source:HGNC Symbol;Acc:HGNC:34408]	-2.719339
ENSG00000144218	3899	AFF3	protein_coding	HGNC Symbol	15	AF4/FMR2 family member 3 [Source:HGNC Symbol;Acc:HGNC:6473]	-2.718816
ENSG00000196167	399948	COLCA1	antisense	HGNC Symbol	5	colorectal cancer associated 1 [Source:HGNC Symbol;Acc:HGNC:33789]	-2.716612
ENSG00000156253	10069	RWDD2B	protein_coding	HGNC Symbol	7	RWD domain containing 2B [Source:HGNC Symbol;Acc:HGNC:1302]	-2.716306
ENSG00000163749	339965	CCDC158	protein_coding	HGNC Symbol	5	coiled-coil domain containing 158 [Source:HGNC Symbol;Acc:HGNC:26374]	-2.714802
ENSG00000101997	28952	CCDC22	protein_coding	HGNC Symbol	3	coiled-coil domain containing 22 [Source:HGNC Symbol;Acc:HGNC:28909]	-2.712659
ENSG00000196189	64218	SEMA4A	protein_coding	HGNC Symbol	16	semaphorin 4A [Source:HGNC Symbol;Acc:HGNC:10729]	-2.711765
ENSG00000196177	36	ACADSB	protein_coding	HGNC Symbol	4	acyl-CoA dehydrogenase short/branched chain [Source:HGNC Symbol;Acc:HGNC:91]	-2.709767
ENSG00000206127	728047	GOLGA8O	protein_coding	HGNC Symbol	2	golgin A8 family member O [Source:HGNC Symbol;Acc:HGNC:44406]	-2.709222
ENSG00000129993	863	CBFA2T3	protein_coding	HGNC Symbol	11	CBFA2/RUNX1 translocation partner 3 [Source:HGNC Symbol;Acc:HGNC:1537]	-2.707419
ENSG00000177380	8541	PPFIA3	protein_coding	HGNC Symbol	13	PTPRF interacting protein alpha 3 [Source:HGNC Symbol;Acc:HGNC:9247]	-2.704178
ENSG00000261801	1E+08	LOXL1-AS1	antisense	HGNC Symbol	13	LOXL1 antisense RNA 1 [Source:HGNC Symbol;Acc:HGNC:44169]	-2.703322
ENSG00000099330	79629	OCEL1	protein_coding	HGNC Symbol	15	occludin/ELL domain containing 1 [Source:HGNC Symbol;Acc:HGNC:26221]	-2.702839
ENSG00000141519	55036	CCDC40	protein_coding	HGNC Symbol	16	coiled-coil domain containing 40 [Source:HGNC Symbol;Acc:HGNC:26090]	-2.702389
ENSG00000181143	94025	MUC16	protein_coding	HGNC Symbol	5	mucin 16, cell surface associated [Source:HGNC Symbol;Acc:HGNC:15582]	-2.701253
ENSG00000167619	284339	TMEM145	protein_coding	HGNC Symbol	5	transmembrane protein 145 [Source:HGNC Symbol;Acc:HGNC:26912]	-2.701092
ENSG00000007541	9091	PIGQ	protein_coding	HGNC Symbol	25	phosphatidylinositol glycan anchor biosynthesis class Q [Source:HGNC Symbol;Acc:HGNC:141	-2.700948
ENSG00000213339	81890	QTRT1	protein_coding	HGNC Symbol	12	queuine tRNA-ribosyltransferase catalytic subunit 1 [Source:HGNC Symbol;Acc:HGNC:23797]	-2.699828
ENSG00000204991	84501	SPIRE2	protein_coding	HGNC Symbol	10	spire type actin nucleation factor 2 [Source:HGNC Symbol;Acc:HGNC:30623]	-2.699572
ENSG00000183317	284656	EPHA10	protein_coding	HGNC Symbol	11	EPH receptor A10 [Source:HGNC Symbol;Acc:HGNC:19987]	-2.698858
ENSG00000257989	NA	AC078864.1	lincRNA	Clone-based (Ensembl) gene	1	novel transcript	-2.696873
ENSG00000101463	79953	SYNDIG1	protein_coding	HGNC Symbol	2	synapse differentiation inducing 1 [Source:HGNC Symbol;Acc:HGNC:15885]	-2.695602
ENSG00000229043	NA	AC091729.3	antisense	Clone-based (Ensembl) gene	3		-2.695408
ENSG00000135636	8291	DYSF	protein_coding	HGNC Symbol	19	dysferlin [Source:HGNC Symbol;Acc:HGNC:3097]	-2.695128
ENSG00000099910	84861	KLHL22	protein_coding	HGNC Symbol	12	kelch like family member 22 [Source:HGNC Symbol;Acc:HGNC:25888]	-2.692905
ENSG00000165816	340706	VWA2	protein_coding	HGNC Symbol	3	von Willebrand factor A domain containing 2 [Source:HGNC Symbol;Acc:HGNC:24709]	-2.692654
ENSG00000126107	79654	HECTD3	protein_coding	HGNC Symbol	7	HECT domain E3 ubiquitin protein ligase 3 [Source:HGNC Symbol;Acc:HGNC:26117]	-2.691743
ENSG00000230091	NA	TMEM254-AS1	antisense	HGNC Symbol	3	TMEM254 antisense RNA 1 [Source:HGNC Symbol;Acc:HGNC:27340]	-2.69037
ENSG00000242193	730102	CRYZL2P	transcribed_unitary_pseudogene	HGNC Symbol	4	crystallin zeta like 2, pseudogene [Source:HGNC Symbol;Acc:HGNC:52164]	-2.689199
ENSG00000198910	3897	L1CAM	protein_coding	HGNC Symbol	16	L1 cell adhesion molecule [Source:HGNC Symbol;Acc:HGNC:6470]	-2.688278
ENSG00000162572	6339	SCNN1D	protein_coding	HGNC Symbol	8	sodium channel epithelial 1 delta subunit [Source:HGNC Symbol;Acc:HGNC:10601]	-2.687694

ENSG00000142973	1580	CYP4B1	protein_coding	HGNC Symbol	15	cytochrome P450 family 4 subfamily B member 1 [Source:HGNC Symbol;Acc:HGNC:2644]	-2.686688
ENSG00000174498	9543	IGDCC3	protein_coding	HGNC Symbol	5	immunoglobulin superfamily DCC subclass member 3 [Source:HGNC Symbol;Acc:HGNC:9700]	-2.686481
ENSG00000235257	1.02E+08	ITGA9-AS1	processed_transcript	HGNC Symbol	19	ITGA9 antisense RNA 1 [Source:HGNC Symbol;Acc:HGNC:49668]	-2.686294
ENSG00000278586	NA	AC005088.1	processed_pseudogene	Clone-based (Ensembl) gene	1	ribosomal protein L10 (RPL10) pseudogene	-2.685422
ENSG00000179855	126326	GIPC3	protein_coding	HGNC Symbol	3	GIPC PDZ domain containing family member 3 [Source:HGNC Symbol;Acc:HGNC:18183]	-2.685275
ENSG00000014123	23376	UFL1	protein_coding	HGNC Symbol	2	UFM1 specific ligase 1 [Source:HGNC Symbol;Acc:HGNC:23039]	-2.684617
ENSG00000014216	823	CAPN1	protein_coding	HGNC Symbol	28	calpain 1 [Source:HGNC Symbol;Acc:HGNC:1476]	-2.684257
ENSG00000136720	9394	HS6ST1	protein_coding	HGNC Symbol	4	heparan sulfate 6-O-sulfotransferase 1 [Source:HGNC Symbol;Acc:HGNC:5201]	-2.684215
ENSG00000165912	29763	PACSIN3	protein_coding	HGNC Symbol	11	protein kinase C and casein kinase substrate in neurons 3 [Source:HGNC Symbol;Acc:HGNC:85	-2.683945
ENSG00000158008	2134	EXTL1	protein_coding	HGNC Symbol	4	exostosin like glycosyltransferase 1 [Source:HGNC Symbol;Acc:HGNC:3515]	-2.682307
ENSG00000123191	540	ATP7B	protein_coding	HGNC Symbol	16	ATPase copper transporting beta [Source:HGNC Symbol;Acc:HGNC:870]	-2.681648
ENSG00000126231	8858	PROZ	protein_coding	HGNC Symbol	3	protein Z, vitamin K dependent plasma glycoprotein [Source:HGNC Symbol;Acc:HGNC:9460]	-2.681639
ENSG00000215217	134121	C5orf49	protein_coding	HGNC Symbol	2	chromosome 5 open reading frame 49 [Source:HGNC Symbol;Acc:HGNC:27028]	-2.680831
ENSG00000064687	10347	ABCA7	protein_coding	HGNC Symbol	19	ATP binding cassette subfamily A member 7 [Source:HGNC Symbol;Acc:HGNC:37]	-2.680626
ENSG00000267201	1.02E+08	LINC01775	lincRNA	HGNC Symbol	1	long intergenic non-protein coding RNA 1775 [Source:HGNC Symbol;Acc:HGNC:52565]	-2.679158
ENSG00000271327	NA	AC010201.2	lincRNA	Clone-based (Ensembl) gene	1	novel transcript	-2.677666
ENSG00000186715	11223	MST1L	transcribed_unprocessed_pseudogene	HGNC Symbol	6	macrophage stimulating 1 like [Source:HGNC Symbol;Acc:HGNC:7390]	-2.67672
ENSG00000152822	2911	GRM1	protein_coding	HGNC Symbol	7	glutamate metabotropic receptor 1 [Source:HGNC Symbol;Acc:HGNC:4593]	-2.673217
ENSG00000270110	NA	AL353593.3	antisense	Clone-based (Ensembl) gene	1	novel transcript, antisense to OBSCN	-2.672698
ENSG00000149043	90019	SYT8	protein_coding	HGNC Symbol	14	synaptotagmin 8 [Source:HGNC Symbol;Acc:HGNC:19264]	-2.67035
ENSG00000126602	10131	TRAP1	protein_coding	HGNC Symbol	17	TNF receptor associated protein 1 [Source:HGNC Symbol;Acc:HGNC:16264]	-2.670166
ENSG00000213347	83463	MXD3	protein_coding	HGNC Symbol	9	MAX dimerization protein 3 [Source:HGNC Symbol;Acc:HGNC:14008]	-2.670145
ENSG00000091592	22861	NLRP1	protein_coding	HGNC Symbol	17	NLR family pyrin domain containing 1 [Source:HGNC Symbol;Acc:HGNC:14374]	-2.669575
ENSG00000091592	728392	NLRP1	protein_coding	HGNC Symbol	17	NLR family pyrin domain containing 1 [Source:HGNC Symbol;Acc:HGNC:14374]	-2.669575
ENSG00000227404	NA	KRT8P20	processed_pseudogene	HGNC Symbol	1	keratin 8 pseudogene 20 [Source:HGNC Symbol;Acc:HGNC:33372]	-2.668884
ENSG00000072954	79041	TMEM38A	protein_coding	HGNC Symbol	3	transmembrane protein 38A [Source:HGNC Symbol;Acc:HGNC:28462]	-2.668362
ENSG00000068903	22933	SIRT2	protein_coding	HGNC Symbol	19	sirtuin 2 [Source:HGNC Symbol;Acc:HGNC:10886]	-2.668227
ENSG00000063854	3029	HAGH	protein_coding	HGNC Symbol	12	hydroxyacylglutathione hydrolase [Source:HGNC Symbol;Acc:HGNC:4805]	-2.66621
ENSG00000137274	670	BPHL	protein_coding	HGNC Symbol	12	biphenyl hydrolase like [Source:HGNC Symbol;Acc:HGNC:1094]	-2.665675
ENSG00000106789	7464	CORO2A	protein_coding	HGNC Symbol	2	coronin 2A [Source:HGNC Symbol;Acc:HGNC:2255]	-2.665658
ENSG00000088881	57593	EBF4	protein_coding	HGNC Symbol	11	EBF family member 4 [Source:HGNC Symbol;Acc:HGNC:29278]	-2.664755
ENSG00000230356	NA	NCAPD2P1	processed_pseudogene	HGNC Symbol	1	non-SMC condensin I complex subunit D2 pseudogene 1 [Source:HGNC Symbol;Acc:HGNC:43	-2.664625
ENSG00000197444	55753	OGDHL	protein_coding	HGNC Symbol	6	oxoglutarate dehydrogenase like [Source:HGNC Symbol;Acc:HGNC:25590]	-2.66412
ENSG00000009780	199870	FAM76A	protein_coding	HGNC Symbol	7	family with sequence similarity 76 member A [Source:HGNC Symbol;Acc:HGNC:28530]	-2.66347
ENSG00000132622	116835	HSPA12B	protein_coding	HGNC Symbol	2	heat shock protein family A (Hsp70) member 12B [Source:HGNC Symbol;Acc:HGNC:16193]	-2.663274
ENSG00000171357	541468	LURAP1	protein_coding	HGNC Symbol	1	leucine rich adaptor protein 1 [Source:HGNC Symbol;Acc:HGNC:32327]	-2.662748
ENSG00000128298	80115	BAIAP2L2	protein_coding	HGNC Symbol	3	BAI1 associated protein 2 like 2 [Source:HGNC Symbol;Acc:HGNC:26203]	-2.662377
ENSG00000072135	26469	PTPN18	protein_coding	HGNC Symbol	16	protein tyrosine phosphatase, non-receptor type 18 [Source:HGNC Symbol;Acc:HGNC:9649]	-2.66177
ENSG00000189144	126231	ZNF573	protein_coding	HGNC Symbol	19	zinc finger protein 573 [Source:HGNC Symbol;Acc:HGNC:26420]	-2.661134
ENSG00000137700	2542	SLC37A4	protein_coding	HGNC Symbol	21	solute carrier family 37 member 4 [Source:HGNC Symbol;Acc:HGNC:4061]	-2.659522
ENSG00000130827	55558	PLXNA3	protein_coding	HGNC Symbol	9	plexin A3 [Source:HGNC Symbol;Acc:HGNC:9101]	-2.658921
ENSG00000162999	142679	DUSP19	protein_coding	HGNC Symbol	3	dual specificity phosphatase 19 [Source:HGNC Symbol;Acc:HGNC:18894]	-2.658376
ENSG00000259051	NA	HNRNPUP1	processed_pseudogene	HGNC Symbol	1	heterogeneous nuclear ribonucleoprotein U pseudogene 1 [Source:HGNC Symbol;Acc:HGNC:	-2.658162
ENSG00000108666	64149	C17orf75	protein_coding	HGNC Symbol	13	chromosome 17 open reading frame 75 [Source:HGNC Symbol;Acc:HGNC:30173]	-2.656718
ENSG00000142609	85452	CFAP74	protein_coding	HGNC Symbol	10	cilia and flagella associated protein 74 [Source:HGNC Symbol;Acc:HGNC:29368]	-2.656289
ENSG00000132793	64900	LPIN3	protein_coding	HGNC Symbol	5	lipin 3 [Source:HGNC Symbol;Acc:HGNC:14451]	-2.653922
ENSG00000204136	2681	GGTA1P	transcribed_unitary_pseudogene	HGNC Symbol	5	glycoprotein, alpha-galactosyltransferase 1 pseudogene [Source:HGNC Symbol;Acc:HGNC:425	-2.653061
ENSG00000258881	NA	AC007040.2	protein_coding	Clone-based (Ensembl) gene	2	novel protein	-2.653023
ENSG00000106804	727	C5	protein_coding	HGNC Symbol	5	complement C5 [Source:HGNC Symbol;Acc:HGNC:1331]	-2.65218
ENSG00000147130	9203	ZMYM3	protein_coding	HGNC Symbol	9	zinc finger MYM-type containing 3 [Source:HGNC Symbol;Acc:HGNC:13054]	-2.651519
ENSG00000230280	NA	HNRNPA1P59	processed_pseudogene	HGNC Symbol	1	heterogeneous nuclear ribonucleoprotein A1 pseudogene 59 [Source:HGNC Symbol;Acc:HGN	-2.651011
ENSG00000116984	4548	MTR	protein_coding	HGNC Symbol	5	5-methyltetrahydrofolate-homocysteine methyltransferase [Source:HGNC Symbol;Acc:HGNC:	-2.647862
ENSG00000095585	29760	BLNK	protein_coding	HGNC Symbol	9	B cell linker [Source:HGNC Symbol;Acc:HGNC:14211]	-2.647023
ENSG00000182612	83882	TSPAN10	protein_coding	HGNC Symbol	5	tetraspanin 10 [Source:HGNC Symbol;Acc:HGNC:29942]	-2.646999
ENSG00000254635	NA	WAC-AS1	antisense	HGNC Symbol	2	WAC antisense RNA 1 (head to head) [Source:HGNC Symbol;Acc:HGNC:27347]	-2.646828
ENSG00000198794	192683	SCAMP5	protein_coding	HGNC Symbol	18	secretory carrier membrane protein 5 [Source:HGNC Symbol;Acc:HGNC:30386]	-2.644839
ENSG00000125746	24139	EML2	protein_coding	HGNC Symbol	31	echinoderm microtubule associated protein like 2 [Source:HGNC Symbol;Acc:HGNC:18035]	-2.644774
ENSG00000182013	55228	PNMA8A	protein_coding	HGNC Symbol	3	PNMA family member 8A [Source:HGNC Symbol;Acc:HGNC:25578]	-2.64474
ENSG00000101213	5753	PTK6	protein_coding	HGNC Symbol	2	protein tyrosine kinase 6 [Source:HGNC Symbol;Acc:HGNC:9617]	-2.643695
ENSG00000167207	64127	NOD2	protein_coding	HGNC Symbol	13	nucleotide binding oligomerization domain containing 2 [Source:HGNC Symbol;Acc:HGNC:533	-2.643315
ENSG00000104884	2068	ERCC2	protein_coding	HGNC Symbol	13	ERCC excision repair 2, TFIIH core complex helicase subunit [Source:HGNC Symbol;Acc:HGNC:	-2.642598
ENSG00000213145	1396	CRIP1	protein_coding	HGNC Symbol	6	cysteine rich protein 1 [Source:HGNC Symbol;Acc:HGNC:2360]	-2.642527
ENSG00000214562	728130	NUTM2D	protein_coding	HGNC Symbol	3	NUT family member 2D [Source:HGNC Symbol;Acc:HGNC:23447]	-2.642198
ENSG00000071242	6196	RPS6KA2	protein_coding	HGNC Symbol	13	ribosomal protein S6 kinase A2 [Source:HGNC Symbol;Acc:HGNC:10431]	-2.640157
ENSG00000025708	1890	TYMP	protein_coding	HGNC Symbol	8	thymidine phosphorylase [Source:HGNC Symbol;Acc:HGNC:3148]	-2.639643

ENSG00000094755	2568	GABRP	protein_coding	HGNC Symbol	10	gamma-aminobutyric acid type A receptor pi subunit [Source:HGNC Symbol;Acc:HGNC:4089]	-2.639282
ENSG00000178752	151176	ERFE	protein_coding	HGNC Symbol	7	erythroferrone [Source:HGNC Symbol;Acc:HGNC:26727]	-2.639226
ENSG00000104147	11339	OIP5	protein_coding	HGNC Symbol	2	Opa interacting protein 5 [Source:HGNC Symbol;Acc:HGNC:20300]	-2.638583
ENSG00000255142	NA	AP006621.2	lincRNA	Clone-based (Ensembl) gene	1	novel transcript	-2.63816
ENSG00000122971	35	ACADS	protein_coding	HGNC Symbol	3	acyl-CoA dehydrogenase short chain [Source:HGNC Symbol;Acc:HGNC:90]	-2.637538
ENSG00000214290	120376	COLCA2	protein_coding	HGNC Symbol	7	colorectal cancer associated 2 [Source:HGNC Symbol;Acc:HGNC:26978]	-2.637031
ENSG00000270127	NA	AC027020.2	lincRNA	Clone-based (Ensembl) gene	1	novel transcript	-2.637027
ENSG00000275074	79873	NUDT18	protein_coding	HGNC Symbol	2	nudix hydrolase 18 [Source:HGNC Symbol;Acc:HGNC:26194]	-2.636709
ENSG00000144554	2177	FANCD2	protein_coding	HGNC Symbol	13	FA complementation group D2 [Source:HGNC Symbol;Acc:HGNC:3585]	-2.636565
ENSG00000117115	11240	PADI2	protein_coding	HGNC Symbol	4	peptidyl arginine deiminase 2 [Source:HGNC Symbol;Acc:HGNC:18341]	-2.634726
ENSG00000244509	27350	APOBEC3C	protein_coding	HGNC Symbol	2	apolipoprotein B mRNA editing enzyme catalytic subunit 3C [Source:HGNC Symbol;Acc:HGNC	-2.634266
ENSG00000271119	NA	AC026412.3	lincRNA	Clone-based (Ensembl) gene	1	novel transcript	-2.633909
ENSG00000112659	23113	CUL9	protein_coding	HGNC Symbol	18	cullin 9 [Source:HGNC Symbol;Acc:HGNC:15982]	-2.632569
ENSG00000265480	284085	KRT18P55	transcribed_unprocessed_pseudogene	HGNC Symbol	3	keratin 18 pseudogene 55 [Source:HGNC Symbol;Acc:HGNC:26874]	-2.632329
ENSG00000074621	9187	SLC24A1	protein_coding	HGNC Symbol	12	solute carrier family 24 member 1 [Source:HGNC Symbol;Acc:HGNC:10975]	-2.63167
ENSG00000144488	339768	ESPNL	protein_coding	HGNC Symbol	6	espin like [Source:HGNC Symbol;Acc:HGNC:27937]	-2.63116
ENSG00000245832	1.02E+08	MIR4300HG	lincRNA	HGNC Symbol	3	MIR4300 host gene [Source:HGNC Symbol;Acc:HGNC:52003]	-2.630896
ENSG00000176182	339344	MYPOP	protein_coding	HGNC Symbol	1	Myb related transcription factor, partner of profilin [Source:HGNC Symbol;Acc:HGNC:20178]	-2.630806
ENSG00000161609	147872	CCDC155	protein_coding	HGNC Symbol	15	coiled-coil domain containing 155 [Source:HGNC Symbol;Acc:HGNC:26520]	-2.630672
ENSG00000154358	84033	OBSCN	protein_coding	HGNC Symbol	14	obscurin, cytoskeletal calmodulin and titin-interacting RhoGEF [Source:HGNC Symbol;Acc:HGI	-2.629201
ENSG00000163617	57577	CCDC191	protein_coding	HGNC Symbol	8	coiled-coil domain containing 191 [Source:HGNC Symbol;Acc:HGNC:29272]	-2.628577
ENSG00000102878	3299	HSF4	protein_coding	HGNC Symbol	25	heat shock transcription factor 4 [Source:HGNC Symbol;Acc:HGNC:5227]	-2.628545
ENSG00000125967	63941	NECAB3	protein_coding	HGNC Symbol	22	N-terminal EF-hand calcium binding protein 3 [Source:HGNC Symbol;Acc:HGNC:15851]	-2.628497
ENSG00000160360	26086	GPSM1	protein_coding	HGNC Symbol	6	G protein signaling modulator 1 [Source:HGNC Symbol;Acc:HGNC:17858]	-2.627947
ENSG00000167861	283987	HID1	protein_coding	HGNC Symbol	14	HID1 domain containing [Source:HGNC Symbol;Acc:HGNC:15736]	-2.626681
ENSG00000187535	9742	IFT140	protein_coding	HGNC Symbol	11	intraflagellar transport 140 [Source:HGNC Symbol;Acc:HGNC:29077]	-2.625698
ENSG00000236383	1.01E+08	CCDC200	protein_coding	HGNC Symbol	16	coiled-coil domain containing 200 [Source:HGNC Symbol;Acc:HGNC:43658]	-2.625284
ENSG00000196172	148213	ZNF681	protein_coding	HGNC Symbol	3	zinc finger protein 681 [Source:HGNC Symbol;Acc:HGNC:26457]	-2.625055
ENSG00000257270	NA	AL928654.2	antisense	Clone-based (Ensembl) gene	1	novel transcript, antisense to MTA1	-2.625043
ENSG00000124343	1E+08	XG	protein_coding	HGNC Symbol	6	Xg glycoprotein (Xg blood group) [Source:HGNC Symbol;Acc:HGNC:12806]	-2.624751
ENSG00000124343	7499	XG	protein_coding	HGNC Symbol	6	Xg glycoprotein (Xg blood group) [Source:HGNC Symbol;Acc:HGNC:12806]	-2.624751
ENSG00000197191	375791	CYSRT1	protein_coding	HGNC Symbol	1	cysteine rich tail 1 [Source:HGNC Symbol;Acc:HGNC:30529]	-2.623534
ENSG00000103152	4350	MPG	protein_coding	HGNC Symbol	5	N-methylpurine DNA glycosylase [Source:HGNC Symbol;Acc:HGNC:7211]	-2.623432
ENSG00000163704	285368	PRRT3	protein_coding	HGNC Symbol	3	proline rich transmembrane protein 3 [Source:HGNC Symbol;Acc:HGNC:26591]	-2.623255
ENSG00000259439	1.08E+08	LINC01833	lincRNA	HGNC Symbol	5	long intergenic non-protein coding RNA 1833 [Source:HGNC Symbol;Acc:HGNC:52644]	-2.62278
ENSG00000227354	1.01E+08	RBM26-AS1	antisense	HGNC Symbol	4	RBM26 antisense RNA 1 [Source:HGNC Symbol;Acc:HGNC:39805]	-2.620086
ENSG00000114268	5210	PFKFB4	protein_coding	HGNC Symbol	15	6-phosphofructo-2-kinase/fructose-2,6-biphosphatase 4 [Source:HGNC Symbol;Acc:HGNC:88	-2.619074
ENSG00000173821	57674	RNF213	protein_coding	HGNC Symbol	20	ring finger protein 213 [Source:HGNC Symbol;Acc:HGNC:14539]	-2.618239
ENSG00000187721	NA	GTF2IP3	unprocessed_pseudogene	HGNC Symbol	1	general transcription factor IIi pseudogene 3 [Source:HGNC Symbol;Acc:HGNC:42640]	-2.615408
ENSG00000198049	553	AVPR1B	protein_coding	HGNC Symbol	2	arginine vasopressin receptor 1B [Source:HGNC Symbol;Acc:HGNC:896]	-2.614692
ENSG00000132394	60678	EEFSEC	protein_coding	HGNC Symbol	4	eukaryotic elongation factor, selenocysteine-tRNA specific [Source:HGNC Symbol;Acc:HGNC:2	-2.614522
ENSG00000162949	92291	CAPN13	protein_coding	HGNC Symbol	7	calpain 13 [Source:HGNC Symbol;Acc:HGNC:16663]	-2.614364
ENSG00000111405	8909	ENDOU	protein_coding	HGNC Symbol	4	endonuclease, poly(U) specific [Source:HGNC Symbol;Acc:HGNC:14369]	-2.614144
ENSG00000184923	728118	NUTM2A	protein_coding	HGNC Symbol	2	NUT family member 2A [Source:HGNC Symbol;Acc:HGNC:23438]	-2.613673
ENSG00000131779	8799	PEX11B	protein_coding	HGNC Symbol	3	peroxisomal biogenesis factor 11 beta [Source:HGNC Symbol;Acc:HGNC:8853]	-2.61074
ENSG00000187583	84069	PLEKHN1	protein_coding	HGNC Symbol	5	pleckstrin homology domain containing N1 [Source:HGNC Symbol;Acc:HGNC:25284]	-2.610626
ENSG00000147394	7739	ZNF185	protein_coding	HGNC Symbol	12	zinc finger protein 185 with LIM domain [Source:HGNC Symbol;Acc:HGNC:12976]	-2.609342
ENSG00000114654	79825	EFCC1	protein_coding	HGNC Symbol	2	EF-hand and coiled-coil domain containing 1 [Source:HGNC Symbol;Acc:HGNC:25692]	-2.609318
ENSG00000138028	10669	CGREF1	protein_coding	HGNC Symbol	9	cell growth regulator with EF-hand domain 1 [Source:HGNC Symbol;Acc:HGNC:16962]	-2.60906
ENSG00000132470	3691	ITGB4	protein_coding	HGNC Symbol	13	integrin subunit beta 4 [Source:HGNC Symbol;Acc:HGNC:6158]	-2.607544
ENSG00000171914	83660	TLN2	protein_coding	HGNC Symbol	11	talin 2 [Source:HGNC Symbol;Acc:HGNC:15447]	-2.604425
ENSG00000260470	NA	AC023794.5	lincRNA	Clone-based (Ensembl) gene	1	novel transcript	-2.604053
ENSG00000143590	1944	EFNA3	protein_coding	HGNC Symbol	3	ephrin A3 [Source:HGNC Symbol;Acc:HGNC:3223]	-2.60322
ENSG00000010310	2696	GIPR	protein_coding	HGNC Symbol	8	gastric inhibitory polypeptide receptor [Source:HGNC Symbol;Acc:HGNC:4271]	-2.603097
ENSG00000225177	NA	AL590617.2	antisense	Clone-based (Ensembl) gene	4		-2.603076
ENSG00000100583	161394	SAMD15	protein_coding	HGNC Symbol	2	sterile alpha motif domain containing 15 [Source:HGNC Symbol;Acc:HGNC:18631]	-2.602921
ENSG00000152766	118932	ANKRD22	protein_coding	HGNC Symbol	2	ankyrin repeat domain 22 [Source:HGNC Symbol;Acc:HGNC:28321]	-2.602842
ENSG00000158483	55199	FAM86C1	protein_coding	HGNC Symbol	6	family with sequence similarity 86 member C1 [Source:HGNC Symbol;Acc:HGNC:25561]	-2.602334
ENSG00000076928	9138	ARHGEF1	protein_coding	HGNC Symbol	23	Rho guanine nucleotide exchange factor 1 [Source:HGNC Symbol;Acc:HGNC:681]	-2.601752
ENSG00000076928	1.01E+08	ARHGEF1	protein_coding	HGNC Symbol	23	Rho guanine nucleotide exchange factor 1 [Source:HGNC Symbol;Acc:HGNC:681]	-2.601752
ENSG00000223820	142913	CFL1P1	transcribed_unprocessed_pseudogene	HGNC Symbol	4	cofilin 1 pseudogene 1 [Source:HGNC Symbol;Acc:HGNC:28560]	-2.60169
ENSG00000100034	9647	PPM1F	protein_coding	HGNC Symbol	10	protein phosphatase, Mg2+/Mn2+ dependent 1F [Source:HGNC Symbol;Acc:HGNC:19388]	-2.601495
ENSG00000111358	2967	GTF2H3	protein_coding	HGNC Symbol	14	general transcription factor IIH subunit 3 [Source:HGNC Symbol;Acc:HGNC:4657]	-2.60123
ENSG00000075461	27092	CACNG4	protein_coding	HGNC Symbol	1	calcium voltage-gated channel auxiliary subunit gamma 4 [Source:HGNC Symbol;Acc:HGNC:14	-2.600325
ENSG00000214300	441272	SPDYE3	protein_coding	HGNC Symbol	2	speedy/RINGO cell cycle regulator family member E3 [Source:HGNC Symbol;Acc:HGNC:35462	-2.599825

ENSG00000100350	80020	FOXRED2	protein_coding	HGNC Symbol	5	FAD dependent oxidoreductase domain containing 2 [Source:HGNC Symbol;Acc:HGNC:26264	-2.599642
ENSG00000074211	5522	PPP2R2C	protein_coding	HGNC Symbol	9	protein phosphatase 2 regulatory subunit Bgamma [Source:HGNC Symbol;Acc:HGNC:9306]	-2.59775
ENSG00000183153	125111	GJD3	protein_coding	HGNC Symbol	1	gap junction protein delta 3 [Source:HGNC Symbol;Acc:HGNC:19147]	-2.597572
ENSG00000203485	64423	INF2	protein_coding	HGNC Symbol	9	inverted formin, FH2 and WH2 domain containing [Source:HGNC Symbol;Acc:HGNC:23791]	-2.59722
ENSG00000230199	NA	AL603825.1	processed_pseudogene	Clone-based (Ensembl) gene	1	ribosomal protein L41 (RPL41) pseudogene	-2.596651
ENSG00000198753	5365	PLXNB3	protein_coding	HGNC Symbol	9	plexin B3 [Source:HGNC Symbol;Acc:HGNC:9105]	-2.595602
ENSG00000240912	NA	AC092896.1	processed_pseudogene	Clone-based (Ensembl) gene	1	histone cluster 1 pseudogene	-2.595581
ENSG00000176715	197322	ACSF3	protein_coding	HGNC Symbol	19	acyl-CoA synthetase family member 3 [Source:HGNC Symbol;Acc:HGNC:27288]	-2.593954
ENSG00000215182	4586	MUC5AC	protein_coding	HGNC Symbol	1	mucin 5AC, oligomeric mucus/gel-forming [Source:HGNC Symbol;Acc:HGNC:7515]	-2.593793
ENSG00000187097	957	ENTPD5	protein_coding	HGNC Symbol	8	ectonucleoside triphosphate diphosphohydrolase 5 [Source:HGNC Symbol;Acc:HGNC:3367]	-2.592694
ENSG00000205356	25851	TECPR1	protein_coding	HGNC Symbol	17	tectonin beta-propeller repeat containing 1 [Source:HGNC Symbol;Acc:HGNC:22214]	-2.592102
ENSG00000172824	283848	CES4A	protein_coding	HGNC Symbol	12	carboxylesterase 4A [Source:HGNC Symbol;Acc:HGNC:26741]	-2.592095
ENSG00000133056	5287	PIK3C2B	protein_coding	HGNC Symbol	8	phosphatidylinositol-4-phosphate 3-kinase catalytic subunit type 2 beta [Source:HGNC Symbo	-2.591932
ENSG00000037280	2324	FLT4	protein_coding	HGNC Symbol	12	fms related tyrosine kinase 4 [Source:HGNC Symbol;Acc:HGNC:3767]	-2.589344
ENSG00000104361	79815	NIPAL2	protein_coding	HGNC Symbol	8	NIPA like domain containing 2 [Source:HGNC Symbol;Acc:HGNC:25854]	-2.589324
ENSG00000113494	5618	PRLR	protein_coding	HGNC Symbol	20	prolactin receptor [Source:HGNC Symbol;Acc:HGNC:9446]	-2.588806
ENSG00000221968	3995	FADS3	protein_coding	HGNC Symbol	13	fatty acid desaturase 3 [Source:HGNC Symbol;Acc:HGNC:3576]	-2.588229
ENSG00000177685	283229	CRACR2B	protein_coding	HGNC Symbol	14	calcium release activated channel regulator 2B [Source:HGNC Symbol;Acc:HGNC:28703]	-2.587914
ENSG00000181218	92815	HIST3H2A	protein_coding	HGNC Symbol	1	histone cluster 3 H2A [Source:HGNC Symbol;Acc:HGNC:20507]	-2.586913
ENSG00000169604	84168	ANTXR1	protein_coding	HGNC Symbol	7	ANTXR cell adhesion molecule 1 [Source:HGNC Symbol;Acc:HGNC:21014]	-2.585933
ENSG00000146648	1956	EGFR	protein_coding	HGNC Symbol	11	epidermal growth factor receptor [Source:HGNC Symbol;Acc:HGNC:3236]	-2.58592
ENSG00000174989	26259	FBXW8	protein_coding	HGNC Symbol	3	F-box and WD repeat domain containing 8 [Source:HGNC Symbol;Acc:HGNC:13597]	-2.585153
ENSG00000197497	79788	ZNF665	protein_coding	HGNC Symbol	6	zinc finger protein 665 [Source:HGNC Symbol;Acc:HGNC:25885]	-2.584966
ENSG00000183150	2842	GPR19	protein_coding	HGNC Symbol	5	G protein-coupled receptor 19 [Source:HGNC Symbol;Acc:HGNC:4473]	-2.584385
ENSG00000141337	22901	ARSG	protein_coding	HGNC Symbol	9	arylsulfatase G [Source:HGNC Symbol;Acc:HGNC:24102]	-2.583393
ENSG00000159374	130951	M1AP	protein_coding	HGNC Symbol	9	meiosis 1 associated protein [Source:HGNC Symbol;Acc:HGNC:25183]	-2.583052
ENSG00000149260	726	CAPN5	protein_coding	HGNC Symbol	9	calpain 5 [Source:HGNC Symbol;Acc:HGNC:1482]	-2.582816
ENSG00000234311	NA	AL451069.3	antisense	Clone-based (Ensembl) gene	1	novel transcript	-2.582151
ENSG00000266373	NA	AP002472.1	processed_pseudogene	Clone-based (Ensembl) gene	1	zinc finger protein 592 (ZNF592) pseudogene	-2.580482
ENSG00000094631	10013	HDAC6	protein_coding	HGNC Symbol	44	histone deacetylase 6 [Source:HGNC Symbol;Acc:HGNC:14064]	-2.579839
ENSG00000257341	NA	AL928654.3	protein_coding	Clone-based (Ensembl) gene	6	novel transcript	-2.579337
ENSG00000269947	NA	AC135178.5	lincRNA	Clone-based (Ensembl) gene	1	novel transcript	-2.579004
ENSG00000164845	NA	FAM86FP	transcribed_unprocessed_pseudogene	HGNC Symbol	2	family with sequence similarity 86 member F, pseudogene [Source:HGNC Symbol;Acc:HGNC:4	-2.577988
ENSG00000199059	442891	MIR135B	miRNA	HGNC Symbol	1	microRNA 135b [Source:HGNC Symbol;Acc:HGNC:31760]	-2.577585
ENSG00000196951	1E+08	SCOC-AS1	antisense	HGNC Symbol	3	SCOC antisense RNA 1 [Source:HGNC Symbol;Acc:HGNC:50601]	-2.576614
ENSG00000128815	57705	WDFY4	protein_coding	HGNC Symbol	6	WDFY family member 4 [Source:HGNC Symbol;Acc:HGNC:29323]	-2.575424
ENSG00000126088	7389	UROD	protein_coding	HGNC Symbol	22	uroporphyrinogen decarboxylase [Source:HGNC Symbol;Acc:HGNC:12591]	-2.573372
ENSG00000263142	NA	LRRC37A17P	transcribed_unprocessed_pseudogene	HGNC Symbol	2	leucine rich repeat containing 37 member A17, pseudogene [Source:HGNC Symbol;Acc:HGNC	-2.573036
ENSG00000272068	NA	AL365181.2	lincRNA	Clone-based (Ensembl) gene	1	novel transcript	-2.572477
ENSG00000099864	5064	PALM	protein_coding	HGNC Symbol	11	paralemmin [Source:HGNC Symbol;Acc:HGNC:8594]	-2.572262
ENSG00000159173	7135	TNNI1	protein_coding	HGNC Symbol	7	troponin I1, slow skeletal type [Source:HGNC Symbol;Acc:HGNC:11945]	-2.572042
ENSG00000188191	5575	PRKAR1B	protein_coding	HGNC Symbol	12	protein kinase cAMP-dependent type I regulatory subunit beta [Source:HGNC Symbol;Acc:HG	-2.571956
ENSG00000107404	1855	DVL1	protein_coding	HGNC Symbol	8	dishevelled segment polarity protein 1 [Source:HGNC Symbol;Acc:HGNC:3084]	-2.57168
ENSG00000254429	NA	AP001972.1	antisense	Clone-based (Ensembl) gene	1	novel transcript	-2.569832
ENSG00000231584	NA	FAHD2CP	transcribed_unprocessed_pseudogene	HGNC Symbol	5	fumarylacetoacetate hydrolase domain containing 2C, pseudogene [Source:HGNC Symbol;Acc	-2.569788
ENSG00000145439	84869	CBR4	protein_coding	HGNC Symbol	7	carbonyl reductase 4 [Source:HGNC Symbol;Acc:HGNC:25891]	-2.569664
ENSG00000115594	3554	IL1R1	protein_coding	HGNC Symbol	13	interleukin 1 receptor type 1 [Source:HGNC Symbol;Acc:HGNC:5993]	-2.568718
ENSG00000127564	9088	PKMYT1	protein_coding	HGNC Symbol	20	protein kinase, membrane associated tyrosine/threonine 1 [Source:HGNC Symbol;Acc:HGNC::	-2.567041
ENSG00000143409	55793	MINDY1	protein_coding	HGNC Symbol	7	MINDY lysine 48 deubiquitinase 1 [Source:HGNC Symbol;Acc:HGNC:25648]	-2.566537
ENSG00000164332	134510	UBLCP1	protein_coding	HGNC Symbol	3	ubiquitin like domain containing CTD phosphatase 1 [Source:HGNC Symbol;Acc:HGNC:28110]	-2.566427
ENSG00000074855	57719	ANO8	protein_coding	HGNC Symbol	4	anoctamin 8 [Source:HGNC Symbol;Acc:HGNC:29329]	-2.565723
ENSG00000139641	23344	ESYT1	protein_coding	HGNC Symbol	10	extended synaptotagmin 1 [Source:HGNC Symbol;Acc:HGNC:29534]	-2.565648
ENSG00000100075	6576	SLC25A1	protein_coding	HGNC Symbol	5	solute carrier family 25 member 1 [Source:HGNC Symbol;Acc:HGNC:10979]	-2.564445
ENSG00000250790	NA	AC127070.2	lincRNA	Clone-based (Ensembl) gene	1	novel transcript	-2.564284
ENSG00000135838	80896	NPL	protein_coding	HGNC Symbol	12	N-acetylneuraminate pyruvate lyase [Source:HGNC Symbol;Acc:HGNC:16781]	-2.563891
ENSG00000221890	23467	NPTXR	protein_coding	HGNC Symbol	2	neuronal pentraxin receptor [Source:HGNC Symbol;Acc:HGNC:7954]	-2.563364
ENSG00000184986	80757	TMEM121	protein_coding	HGNC Symbol	3	transmembrane protein 121 [Source:HGNC Symbol;Acc:HGNC:20511]	-2.563238
ENSG00000197694	6709	SPTAN1	protein_coding	HGNC Symbol	38	spectrin alpha, non-erythrocytic 1 [Source:HGNC Symbol;Acc:HGNC:11273]	-2.562979
ENSG00000185813	5833	PCYT2	protein_coding	HGNC Symbol	18	phosphate cytidylyltransferase 2, ethanolamine [Source:HGNC Symbol;Acc:HGNC:8756]	-2.561084
ENSG00000100897	80344	DCAF11	protein_coding	HGNC Symbol	38	DDB1 and CUL4 associated factor 11 [Source:HGNC Symbol;Acc:HGNC:20258]	-2.560666
ENSG00000115041	30818	KCNIP3	protein_coding	HGNC Symbol	7	potassium voltage-gated channel interacting protein 3 [Source:HGNC Symbol;Acc:HGNC:1552	-2.56056
ENSG00000146243	134728	IRAK1BP1	protein_coding	HGNC Symbol	4	interleukin 1 receptor associated kinase 1 binding protein 1 [Source:HGNC Symbol;Acc:HGNC:	-2.560528
ENSG00000100991	26133	TRPC4AP	protein_coding	HGNC Symbol	2	transient receptor potential cation channel subfamily C member 4 associated protein [Source:	-2.560405
ENSG00000278463	8335	HIST1H2AB	protein_coding	HGNC Symbol	1	histone cluster 1 H2A family member b [Source:HGNC Symbol;Acc:HGNC:4734]	-2.559374
ENSG00000257647	NA	AC124312.2	lincRNA	Clone-based (Ensembl) gene	1	novel transcript	-2.558823

ENSG00000242498	348110	ARPIN	protein_coding	HGNC Symbol	3	actin related protein 2/3 complex inhibitor [Source:HGNC Symbol;Acc:HGNC:28782]	-2.558179
ENSG00000278570	10002	NR2E3	protein_coding	HGNC Symbol	4	nuclear receptor subfamily 2 group E member 3 [Source:HGNC Symbol;Acc:HGNC:7974]	-2.558171
ENSG00000079785	1653	DDX1	protein_coding	HGNC Symbol	8	DEAD-box helicase 1 [Source:HGNC Symbol;Acc:HGNC:2734]	-2.556002
ENSG00000263818	1E+08	RDM1P5	transcribed_processed_pseudogene	HGNC Symbol	4	RDM1 pseudogene 5 [Source:HGNC Symbol;Acc:HGNC:53921]	-2.555611
ENSG00000235092	1.01E+08	ID2-AS1	antisense	HGNC Symbol	7	ID2 antisense RNA 1 [Source:HGNC Symbol;Acc:HGNC:51103]	-2.554092
ENSG00000159792	5681	PSKH1	protein_coding	HGNC Symbol	3	protein serine kinase H1 [Source:HGNC Symbol;Acc:HGNC:9529]	-2.552287
ENSG00000100078	50487	PLA2G3	protein_coding	HGNC Symbol	1	phospholipase A2 group III [Source:HGNC Symbol;Acc:HGNC:17934]	-2.551357
ENSG00000100156	23539	SLC16A8	protein_coding	HGNC Symbol	3	solute carrier family 16 member 8 [Source:HGNC Symbol;Acc:HGNC:16270]	-2.550422
ENSG00000261324	NA	AC010168.2	sense_overlapping	Clone-based (Ensembl) gene	1	novel transcript, overlapping HIST4H4	-2.550138
ENSG00000196535	399687	MYO18A	protein_coding	HGNC Symbol	24	myosin XVIIIA [Source:HGNC Symbol;Acc:HGNC:31104]	-2.549862
ENSG00000214433	NA	GOLGA2P8	transcribed_unprocessed_pseudogene	HGNC Symbol	1	GOLGA2 pseudogene 8 [Source:HGNC Symbol;Acc:HGNC:49922]	-2.548383
ENSG00000251411	NA	AC093827.3	processed_pseudogene	Clone-based (Ensembl) gene	1	actin related protein 2/3 complex, subunit 1A, 41kDa (ARPC1A) pseudogene	-2.54793
ENSG00000177990	283417	DPY19L2	protein_coding	HGNC Symbol	9	dpy-19 like 2 [Source:HGNC Symbol;Acc:HGNC:19414]	-2.547776
ENSG00000143374	80222	TARS2	protein_coding	HGNC Symbol	14	threonyl-tRNA synthetase 2, mitochondrial [Source:HGNC Symbol;Acc:HGNC:30740]	-2.54747
ENSG00000105135	10994	ILVBL	protein_coding	HGNC Symbol	14	ilvB acetolactate synthase like [Source:HGNC Symbol;Acc:HGNC:6041]	-2.546575
ENSG00000146425	6993	DYNLT1	protein_coding	HGNC Symbol	3	dynein light chain Tctex-type 1 [Source:HGNC Symbol;Acc:HGNC:11697]	-2.54633
ENSG00000118162	11133	KPTN	protein_coding	HGNC Symbol	9	kaptin, actin binding protein [Source:HGNC Symbol;Acc:HGNC:6404]	-2.546309
ENSG00000185344	23545	ATP6V0A2	protein_coding	HGNC Symbol	9	ATPase H+ transporting V0 subunit a2 [Source:HGNC Symbol;Acc:HGNC:18481]	-2.545448
ENSG00000205464	92270	ATP6AP1L	protein_coding	HGNC Symbol	6	ATPase H+ transporting accessory protein 1 like [Source:HGNC Symbol;Acc:HGNC:28091]	-2.54484
ENSG00000156521	219743	TYSND1	protein_coding	HGNC Symbol	4	trypsin domain containing 1 [Source:HGNC Symbol;Acc:HGNC:28531]	-2.544687
ENSG00000115297	3196	TLX2	protein_coding	HGNC Symbol	4	T cell leukemia homeobox 2 [Source:HGNC Symbol;Acc:HGNC:5057]	-2.544541
ENSG00000110455	84680	ACCS	protein_coding	HGNC Symbol	13	1-aminocyclopropane-1-carboxylate synthase homolog (inactive) [Source:HGNC Symbol;Acc:H	-2.543909
ENSG00000140854	10300	KATNB1	protein_coding	HGNC Symbol	10	katanin regulatory subunit B1 [Source:HGNC Symbol;Acc:HGNC:6217]	-2.543101
ENSG00000134146	89978	DPH6	protein_coding	HGNC Symbol	9	diphthamine biosynthesis 6 [Source:HGNC Symbol;Acc:HGNC:30543]	-2.540566
ENSG00000101850	4935	GPR143	protein_coding	HGNC Symbol	5	G protein-coupled receptor 143 [Source:HGNC Symbol;Acc:HGNC:20145]	-2.540412
ENSG00000204682	399726	CASC10	protein_coding	HGNC Symbol	1	cancer susceptibility 10 [Source:HGNC Symbol;Acc:HGNC:31448]	-2.540329
ENSG00000159199	516	ATP5MC1	protein_coding	HGNC Symbol	10	ATP synthase membrane subunit c locus 1 [Source:HGNC Symbol;Acc:HGNC:841]	-2.540229
ENSG00000165948	122509	IFI27L1	protein_coding	HGNC Symbol	13	interferon alpha inducible protein 27 like 1 [Source:HGNC Symbol;Acc:HGNC:19754]	-2.539241
ENSG00000177963	60626	RIC8A	protein_coding	HGNC Symbol	17	RIC8 guanine nucleotide exchange factor A [Source:HGNC Symbol;Acc:HGNC:29550]	-2.53818
ENSG00000165186	139411	PTCHD1	protein_coding	HGNC Symbol	2	patched domain containing 1 [Source:HGNC Symbol;Acc:HGNC:26392]	-2.537588
ENSG00000101440	434	ASIP	protein_coding	HGNC Symbol	2	agouti signaling protein [Source:HGNC Symbol;Acc:HGNC:745]	-2.537524
ENSG00000010610	920	CD4	protein_coding	HGNC Symbol	12	CD4 molecule [Source:HGNC Symbol;Acc:HGNC:1678]	-2.536847
ENSG00000255794	196475	RMST	processed_transcript	HGNC Symbol	10	rhabdomyosarcoma 2 associated transcript [Source:HGNC Symbol;Acc:HGNC:29893]	-2.53683
ENSG00000171703	6919	TCEA2	protein_coding	HGNC Symbol	17	transcription elongation factor A2 [Source:HGNC Symbol;Acc:HGNC:11614]	-2.53643
ENSG00000065621	119391	GSTO2	protein_coding	HGNC Symbol	7	glutathione S-transferase omega 2 [Source:HGNC Symbol;Acc:HGNC:23064]	-2.535771
ENSG00000188833	377841	ENTPD8	protein_coding	HGNC Symbol	5	ectonucleoside triphosphate diphosphohydrolase 8 [Source:HGNC Symbol;Acc:HGNC:24860]	-2.534543
ENSG00000157927	55698	RADIL	protein_coding	HGNC Symbol	7	Rap associating with DIL domain [Source:HGNC Symbol;Acc:HGNC:22226]	-2.534034
ENSG00000174740	140886	PABPC5	protein_coding	HGNC Symbol	2	poly(A) binding protein cytoplasmic 5 [Source:HGNC Symbol;Acc:HGNC:13629]	-2.53365
ENSG00000070371	8218	CLTCL1	protein_coding	HGNC Symbol	12	clathrin heavy chain like 1 [Source:HGNC Symbol;Acc:HGNC:2093]	-2.532766
ENSG00000185432	25840	METTL7A	protein_coding	HGNC Symbol	5	methyltransferase like 7A [Source:HGNC Symbol;Acc:HGNC:24550]	-2.53203
ENSG00000130005	2593	GAMT	protein_coding	HGNC Symbol	5	guanidinoacetate N-methyltransferase [Source:HGNC Symbol;Acc:HGNC:4136]	-2.531978
ENSG00000125841	80023	NRSN2	protein_coding	HGNC Symbol	10	neurensin 2 [Source:HGNC Symbol;Acc:HGNC:16229]	-2.531887
ENSG00000196668	1E+08	LINC00173	processed_transcript	HGNC Symbol	4	long intergenic non-protein coding RNA 173 [Source:HGNC Symbol;Acc:HGNC:33791]	-2.530742
ENSG00000127586	63922	CHTF18	protein_coding	HGNC Symbol	20	chromosome transmission fidelity factor 18 [Source:HGNC Symbol;Acc:HGNC:18435]	-2.52943
ENSG00000113946	10686	CLDN16	protein_coding	HGNC Symbol	3	claudin 16 [Source:HGNC Symbol;Acc:HGNC:2037]	-2.529051
ENSG00000126259	84063	KIRREL2	protein_coding	HGNC Symbol	5	kirre like nephrin family adhesion molecule 2 [Source:HGNC Symbol;Acc:HGNC:18816]	-2.528753
ENSG00000100324	10454	TAB1	protein_coding	HGNC Symbol	7	TGF-beta activated kinase 1 (MAP3K7) binding protein 1 [Source:HGNC Symbol;Acc:HGNC:18	-2.528316
ENSG00000136378	11173	ADAMTS7	protein_coding	HGNC Symbol	5	ADAM metallopeptidase with thrombospondin type 1 motif 7 [Source:HGNC Symbol;Acc:HGN	-2.527316
ENSG00000159625	84229	DRC7	protein_coding	HGNC Symbol	10	dynein regulatory complex subunit 7 [Source:HGNC Symbol;Acc:HGNC:25289]	-2.526285
ENSG00000258457	NA	AL132780.2	antisense	Clone-based (Ensembl) gene	3		-2.523619
ENSG00000233834	1.01E+08	AC005083.1	processed_transcript	Clone-based (Ensembl) gene	3		-2.523265
ENSG00000160161	148113	CILP2	protein_coding	HGNC Symbol	3	cartilage intermediate layer protein 2 [Source:HGNC Symbol;Acc:HGNC:24213]	-2.522938
ENSG00000100033	5625	PRODH	protein_coding	HGNC Symbol	15	proline dehydrogenase 1 [Source:HGNC Symbol;Acc:HGNC:9453]	-2.522444
ENSG00000113597	80006	TRAPPC13	protein_coding	HGNC Symbol	12	trafficking protein particle complex 13 [Source:HGNC Symbol;Acc:HGNC:25828]	-2.521706
ENSG00000104983	729440	CCDC61	protein_coding	HGNC Symbol	8	coiled-coil domain containing 61 [Source:HGNC Symbol;Acc:HGNC:33629]	-2.521484
ENSG00000149485	3992	FADS1	protein_coding	HGNC Symbol	21	fatty acid desaturase 1 [Source:HGNC Symbol;Acc:HGNC:3574]	-2.521122
ENSG00000215529	388795	EFCAB8	protein_coding	HGNC Symbol	4	EF-hand calcium binding domain 8 [Source:HGNC Symbol;Acc:HGNC:34532]	-2.520872
ENSG00000160326	11182	SLC2A6	protein_coding	HGNC Symbol	5	solute carrier family 2 member 6 [Source:HGNC Symbol;Acc:HGNC:11011]	-2.520623
ENSG00000167792	4723	NDUFV1	protein_coding	HGNC Symbol	29	NADH:ubiquinone oxidoreductase core subunit V1 [Source:HGNC Symbol;Acc:HGNC:7716]	-2.5197
ENSG00000142530	112703	FAM71E1	protein_coding	HGNC Symbol	6	family with sequence similarity 71 member E1 [Source:HGNC Symbol;Acc:HGNC:25107]	-2.519516
ENSG00000109794	25854	FAM149A	protein_coding	HGNC Symbol	18	family with sequence similarity 149 member A [Source:HGNC Symbol;Acc:HGNC:24527]	-2.519046
ENSG00000246763	503569	RGMB-AS1	antisense	HGNC Symbol	5	RGMB antisense RNA 1 [Source:HGNC Symbol;Acc:HGNC:48666]	-2.518955
ENSG00000186301	NA	MST1P2	unprocessed_pseudogene	HGNC Symbol	1	macrophage stimulating 1 pseudogene 2 [Source:HGNC Symbol;Acc:HGNC:7383]	-2.5189
ENSG00000262583	NA	TMEM231P1	transcribed_unprocessed_pseudogene	HGNC Symbol	2	TMEM231 pseudogene 1 [Source:HGNC Symbol;Acc:HGNC:53870]	-2.51827
ENSG00000016391	55349	CHDH	protein_coding	HGNC Symbol	3	choline dehydrogenase [Source:HGNC Symbol;Acc:HGNC:24288]	-2.517455

ENSG00000117971	1143	CHRNB4	protein_coding	HGNC Symbol	6	cholinergic receptor nicotinic beta 4 subunit [Source:HGNC Symbol;Acc:HGNC:1964]	-2.516733
ENSG00000131584	116983	ACAP3	protein_coding	HGNC Symbol	13	ArfGAP with coiled-coil, ankyrin repeat and PH domains 3 [Source:HGNC Symbol;Acc:HGNC:1(	-2.516723
ENSG00000172731	55222	LRRC20	protein_coding	HGNC Symbol	7	leucine rich repeat containing 20 [Source:HGNC Symbol;Acc:HGNC:23421]	-2.516587
ENSG00000119772	1788	DNMT3A	protein_coding	HGNC Symbol	15	DNA methyltransferase 3 alpha [Source:HGNC Symbol;Acc:HGNC:2978]	-2.516083
ENSG00000172890	55191	NADSYN1	protein_coding	HGNC Symbol	23	NAD synthetase 1 [Source:HGNC Symbol;Acc:HGNC:29832]	-2.514534
ENSG00000128872	29767	TMOD2	protein_coding	HGNC Symbol	6	tropomodulin 2 [Source:HGNC Symbol;Acc:HGNC:11872]	-2.513847
ENSG00000166924	222950	NYAP1	protein_coding	HGNC Symbol	4	neuronal tyrosine phosphorylated phosphoinositide-3-kinase adaptor 1 [Source:HGNC Symbo	-2.513366
ENSG00000242136	NA	AC093904.2	processed_transcript	Clone-based (Ensembl) gene	2	novel transcript	-2.513114
ENSG00000155629	118788	PIK3AP1	protein_coding	HGNC Symbol	6	phosphoinositide-3-kinase adaptor protein 1 [Source:HGNC Symbol;Acc:HGNC:30034]	-2.512301
ENSG00000105707	3249	HPN	protein_coding	HGNC Symbol	9	hepsin [Source:HGNC Symbol;Acc:HGNC:5155]	-2.511549
ENSG00000156509	286151	FBXO43	protein_coding	HGNC Symbol	3	F-box protein 43 [Source:HGNC Symbol;Acc:HGNC:28521]	-2.510764
ENSG00000166126	81693	AMN	protein_coding	HGNC Symbol	7	amnion associated transmembrane protein [Source:HGNC Symbol;Acc:HGNC:14604]	-2.510486
ENSG00000205517	57139	RGL3	protein_coding	HGNC Symbol	17	ral guanine nucleotide dissociation stimulator like 3 [Source:HGNC Symbol;Acc:HGNC:30282]	-2.509835
ENSG00000162620	127255	LRRIQ3	protein_coding	HGNC Symbol	10	leucine rich repeats and IQ motif containing 3 [Source:HGNC Symbol;Acc:HGNC:28318]	-2.509624
ENSG00000274089	145270	PRIMA1	protein_coding	HGNC Symbol	4	proline rich membrane anchor 1 [Source:HGNC Symbol;Acc:HGNC:18319]	-2.508907
ENSG00000224769	NA	MUC20P1	unprocessed_pseudogene	HGNC Symbol	1	mucin 20, cell surface associated pseudogene 1 [Source:HGNC Symbol;Acc:HGNC:51921]	-2.508773
ENSG00000189077	83862	TMEM120A	protein_coding	HGNC Symbol	15	transmembrane protein 120A [Source:HGNC Symbol;Acc:HGNC:21697]	-2.505702
ENSG00000133739	85444	LRRCC1	protein_coding	HGNC Symbol	6	leucine rich repeat and coiled-coil centrosomal protein 1 [Source:HGNC Symbol;Acc:HGNC:29	-2.504506
ENSG00000206457	54535	CCHCR1	protein_coding	HGNC Symbol	16	coiled-coil alpha-helical rod protein 1 [Source:HGNC Symbol;Acc:HGNC:13930]	-2.504299
ENSG00000165238	65268	WNK2	protein_coding	HGNC Symbol	16	WNK lysine deficient protein kinase 2 [Source:HGNC Symbol;Acc:HGNC:14542]	-2.50427
ENSG00000130758	4294	MAP3K10	protein_coding	HGNC Symbol	8	mitogen-activated protein kinase kinase kinase 10 [Source:HGNC Symbol;Acc:HGNC:6849]	-2.503814
ENSG00000172830	54961	SSH3	protein_coding	HGNC Symbol	11	slingshot protein phosphatase 3 [Source:HGNC Symbol;Acc:HGNC:30581]	-2.502165
ENSG00000176533	2788	GNG7	protein_coding	HGNC Symbol	2	G protein subunit gamma 7 [Source:HGNC Symbol;Acc:HGNC:4410]	-2.502092
ENSG00000125434	399512	SLC25A35	protein_coding	HGNC Symbol	7	solute carrier family 25 member 35 [Source:HGNC Symbol;Acc:HGNC:31921]	-2.501366
ENSG00000073331	80216	ALPK1	protein_coding	HGNC Symbol	18	alpha kinase 1 [Source:HGNC Symbol;Acc:HGNC:20917]	-2.500838
ENSG00000156587	9246	UBE2L6	protein_coding	HGNC Symbol	5	ubiquitin conjugating enzyme E2 L6 [Source:HGNC Symbol;Acc:HGNC:12490]	-2.50011
ENSG00000171224	219738	FAM241B	protein_coding	HGNC Symbol	3	family with sequence similarity 241 member B [Source:HGNC Symbol;Acc:HGNC:23519]	2.500005
ENSG00000187079	7003	TEAD1	protein_coding	HGNC Symbol	7	TEA domain transcription factor 1 [Source:HGNC Symbol;Acc:HGNC:11714]	2.502947
ENSG00000173575	1106	CHD2	protein_coding	HGNC Symbol	29	chromodomain helicase DNA binding protein 2 [Source:HGNC Symbol;Acc:HGNC:1917]	2.503817
ENSG00000204778	NA	CBWD4P	unprocessed_pseudogene	HGNC Symbol	1	COBW domain containing 4 pseudogene [Source:HGNC Symbol;Acc:HGNC:18520]	2.506027
ENSG00000270060	NA	AC090589.3	sense_intronic	Clone-based (Ensembl) gene	1	novel transcript	2.507169
ENSG00000198576	23237	ARC	protein_coding	HGNC Symbol	2	activity regulated cytoskeleton associated protein [Source:HGNC Symbol;Acc:HGNC:648]	2.508034
ENSG00000102554	688	KLF5	protein_coding	HGNC Symbol	5	Kruppel like factor 5 [Source:HGNC Symbol;Acc:HGNC:6349]	2.509548
ENSG00000223583	NA	AL513365.1	processed_pseudogene	Clone-based (Ensembl) gene	1	ribosomal protein L17 (RPL17) pseudogene	2.51164
ENSG00000250848	NA	AC021087.2	processed_pseudogene	Clone-based (Ensembl) gene	1	protein phosphatase 4, regulatory subunit 2 (PPP4R2) pseudogene	2.511642
ENSG00000026508	960	CD44	protein_coding	HGNC Symbol	39	CD44 molecule (Indian blood group) [Source:HGNC Symbol;Acc:HGNC:1681]	2.51215
ENSG00000186352	353322	ANKRD37	protein_coding	HGNC Symbol	6	ankyrin repeat domain 37 [Source:HGNC Symbol;Acc:HGNC:29593]	2.512897
ENSG00000112559	4188	MDFI	protein_coding	HGNC Symbol	9	MyoD family inhibitor [Source:HGNC Symbol;Acc:HGNC:6967]	2.513375
ENSG00000250031	NA	AC009927.1	processed_pseudogene	Clone-based (Ensembl) gene	1	protein tyrosine phosphatase, non-receptor type 11 (PTPN11) pseudogene	2.515079
ENSG00000231395	NA	ARL4AP4	processed_pseudogene	HGNC Symbol	1	ADP ribosylation factor like GTPase 4A pseudogene 4 [Source:HGNC Symbol;Acc:HGNC:52373	2.515874
ENSG00000226971	NA	AL606490.2	processed_pseudogene	Clone-based (Ensembl) gene	1	ornithine aminotransferase (gyrate atrophy) (OAT) pseudogene	2.517107
ENSG00000242689	1270	CNTF	protein_coding	HGNC Symbol	1	ciliary neurotrophic factor [Source:HGNC Symbol;Acc:HGNC:2169]	2.520008
ENSG00000136866	7539	ZFP37	protein_coding	HGNC Symbol	3	ZFP37 zinc finger protein [Source:HGNC Symbol;Acc:HGNC:12863]	2.521497
ENSG00000164920	116039	OSR2	protein_coding	HGNC Symbol	10	odd-skipped related transciption factor 2 [Source:HGNC Symbol;Acc:HGNC:15830]	2.522059
ENSG00000086589	55696	RBM22	protein_coding	HGNC Symbol	8	RNA binding motif protein 22 [Source:HGNC Symbol;Acc:HGNC:25503]	2.522845
ENSG00000218418	NA	AL591135.1	processed_pseudogene	Clone-based (Ensembl) gene	1	Rho GTPase activating protein 21 (ARHGAP21) pseudogene	2.523056
ENSG00000275400	NA	AC006001.4	processed_pseudogene	Clone-based (Ensembl) gene	1	translocase of outer mitochondrial membrane 20 homolog (yeast) (TOMM20) pseudogene	2.524726
ENSG00000127774	83460	EMC6	protein_coding	HGNC Symbol	2	ER membrane protein complex subunit 6 [Source:HGNC Symbol;Acc:HGNC:28430]	2.524948
ENSG00000117519	1266	CNN3	protein_coding	HGNC Symbol	7	calponin 3 [Source:HGNC Symbol;Acc:HGNC:2157]	2.525108
ENSG00000105509	3036	HAS1	protein_coding	HGNC Symbol	5	hyaluronan synthase 1 [Source:HGNC Symbol;Acc:HGNC:4818]	2.525175
ENSG00000260743	NA	AC007823.1	lincRNA	Clone-based (Ensembl) gene	1	novel transcript	2.525185
ENSG00000130775	9473	THEMIS2	protein_coding	HGNC Symbol	11	thymocyte selection associated family member 2 [Source:HGNC Symbol;Acc:HGNC:16839]	2.525532
ENSG00000240793	NA	UBA52P8	processed_pseudogene	HGNC Symbol	1	ubiquitin A-52 residue ribosomal protein fusion product 1 pseudogene 8 [Source:HGNC Symb	2.525871
ENSG00000223922	NA	ASS1P2	processed_pseudogene	HGNC Symbol	1	argininosuccinate synthetase 1 pseudogene 2 [Source:HGNC Symbol;Acc:HGNC:765]	2.527001
ENSG00000198746	63906	GPATCH3	protein_coding	HGNC Symbol	3	G-patch domain containing 3 [Source:HGNC Symbol;Acc:HGNC:25720]	2.527146
ENSG00000151376	10873	ME3	protein_coding	HGNC Symbol	12	malic enzyme 3 [Source:HGNC Symbol;Acc:HGNC:6985]	2.527343
ENSG00000089737	57062	DDX24	protein_coding	HGNC Symbol	11	DEAD-box helicase 24 [Source:HGNC Symbol;Acc:HGNC:13266]	2.528554
ENSG00000116903	149371	EXOC8	protein_coding	HGNC Symbol	1	exocyst complex component 8 [Source:HGNC Symbol;Acc:HGNC:24659]	2.52872
ENSG00000278330	NA	AC018529.2	antisense	Clone-based (Ensembl) gene	2	novel transcript, antisense to MBP	2.529019
ENSG00000114019	51421	AMOTL2	protein_coding	HGNC Symbol	13	angiomotin like 2 [Source:HGNC Symbol;Acc:HGNC:17812]	2.529319
ENSG00000204178	55219	MACO1	protein_coding	HGNC Symbol	4	macoilin 1 [Source:HGNC Symbol;Acc:HGNC:25572]	2.529984
ENSG00000188283	163087	ZNF383	protein_coding	HGNC Symbol	8	zinc finger protein 383 [Source:HGNC Symbol;Acc:HGNC:18609]	2.532163
ENSG00000053438	4826	NNAT	protein_coding	HGNC Symbol	6	neuronatin [Source:HGNC Symbol;Acc:HGNC:7860]	2.532769
ENSG00000274386	1E+08	TMEM269	protein_coding	HGNC Symbol	4	transmembrane protein 269 [Source:HGNC Symbol;Acc:HGNC:52381]	2.533287
ENSG00000240445	NA	FOXO3B	processed_pseudogene	HGNC Symbol	1	forkhead box O3B pseudogene [Source:HGNC Symbol;Acc:HGNC:3822]	2.535101

ENSG00000259514	NA	AC027243.1	antisense	Clone-based (Ensembl) gene	1	novel transcript, antisense to ISL2	2.535314
ENSG00000184659	349334	FOXD4L4	protein_coding	HGNC Symbol	1	forkhead box D4 like 4 [Source:HGNC Symbol;Acc:HGNC:23762]	2.535799
ENSG00000259751	NA	AC018868.2	processed_pseudogene	Clone-based (Ensembl) gene	1	tubulin, alpha 1b (TUBA1B) pseudogene	2.536082
ENSG00000075223	10512	SEMA3C	protein_coding	HGNC Symbol	9	semaphorin 3C [Source:HGNC Symbol;Acc:HGNC:10725]	2.536654
ENSG00000139263	121227	LRIG3	protein_coding	HGNC Symbol	9	leucine rich repeats and immunoglobulin like domains 3 [Source:HGNC Symbol;Acc:HGNC:30(	2.537005
ENSG00000244124	NA	ATP1B3-AS1	antisense	HGNC Symbol	1	ATP1B3 antisense RNA 1 [Source:HGNC Symbol;Acc:HGNC:40088]	2.537249
ENSG00000106636	10652	YKT6	protein_coding	HGNC Symbol	7	YKT6 v-SNARE homolog [Source:HGNC Symbol;Acc:HGNC:16959]	2.537294
ENSG00000212607	677826	SNORA3B	snoRNA	HGNC Symbol	1	small nucleolar RNA, H/ACA box 3B [Source:HGNC Symbol;Acc:HGNC:32638]	2.538794
ENSG00000270504	NA	AL391422.4	antisense	Clone-based (Ensembl) gene	1	novel transcript, antisense to PXDC1	2.538974
ENSG00000277728	NA	AC097641.2	antisense	Clone-based (Ensembl) gene	1	novel transcript, antisense to COG1	2.540184
ENSG00000187122	6585	SLIT1	protein_coding	HGNC Symbol	7	slit guidance ligand 1 [Source:HGNC Symbol;Acc:HGNC:11085]	2.540593
ENSG00000213782	51202	DDX47	protein_coding	HGNC Symbol	12	DEAD-box helicase 47 [Source:HGNC Symbol;Acc:HGNC:18682]	2.541615
ENSG00000151929	9531	BAG3	protein_coding	HGNC Symbol	2	BCL2 associated athanogene 3 [Source:HGNC Symbol;Acc:HGNC:939]	2.543237
ENSG00000116701	4688	NCF2	protein_coding	HGNC Symbol	7	neutrophil cytosolic factor 2 [Source:HGNC Symbol;Acc:HGNC:7661]	2.545896
ENSG00000231521	1.05E+08	AL162385.2	antisense	Clone-based (Ensembl) gene	1		2.545963
ENSG00000268015	NA	AC010320.2	antisense	Clone-based (Ensembl) gene	3	novel transcript, antisense to PPP2R1A	2.547457
ENSG00000157306	NA	ZFHX2-AS1	processed_transcript	HGNC Symbol	5	ZFHX2 antisense RNA 1 [Source:HGNC Symbol;Acc:HGNC:52658]	2.548206
ENSG00000189099	345062	PRSS48	protein_coding	HGNC Symbol	2	serine protease 48 [Source:HGNC Symbol;Acc:HGNC:24635]	2.548473
ENSG00000254842	NA	LINC02551	lincRNA	HGNC Symbol	2	long intergenic non-protein coding RNA 2551 [Source:HGNC Symbol;Acc:HGNC:53586]	2.549379
ENSG00000196352	1604	CD55	protein_coding	HGNC Symbol	14	CD55 molecule (Cromer blood group) [Source:HGNC Symbol;Acc:HGNC:2665]	2.551264
ENSG00000196756	388796	SNHG17	processed_transcript	HGNC Symbol	8	small nucleolar RNA host gene 17 [Source:HGNC Symbol;Acc:HGNC:48600]	2.551466
ENSG00000117000	6018	RLF	protein_coding	HGNC Symbol	1	rearranged L-myc fusion [Source:HGNC Symbol;Acc:HGNC:10025]	2.553723
ENSG00000049130	4254	KITLG	protein_coding	HGNC Symbol	7	KIT ligand [Source:HGNC Symbol;Acc:HGNC:6343]	2.55469
ENSG00000150459	10284	SAP18	protein_coding	HGNC Symbol	8	Sin3A associated protein 18 [Source:HGNC Symbol;Acc:HGNC:10530]	2.55548
ENSG00000169991	126917	IFFO2	protein_coding	HGNC Symbol	3	intermediate filament family orphan 2 [Source:HGNC Symbol;Acc:HGNC:27006]	2.555533
ENSG00000248781	NA	PSMC1P4	processed_pseudogene	HGNC Symbol	1	proteasome 26S subunit, ATPase 1 pseudogene 4 [Source:HGNC Symbol;Acc:HGNC:39779]	2.557255
ENSG00000254907	NA	AC087276.2	antisense	Clone-based (Ensembl) gene	1	novel transcript, antisense to API5 and TTC17	2.558894
ENSG00000276488	NA	AC008735.4	antisense	Clone-based (Ensembl) gene	1	novel transcript, antisense to ZNF865	2.559726
ENSG00000127804	79066	METTL16	protein_coding	HGNC Symbol	10	methyltransferase like 16 [Source:HGNC Symbol;Acc:HGNC:28484]	2.559743
ENSG00000087245	4313	MMP2	protein_coding	HGNC Symbol	8	matrix metallopeptidase 2 [Source:HGNC Symbol;Acc:HGNC:7166]	2.559755
ENSG00000248971	NA	KRT8P46	processed_pseudogene	HGNC Symbol	1	keratin 8 pseudogene 46 [Source:HGNC Symbol;Acc:HGNC:39880]	2.559801
ENSG00000126524	51119	SBDS	protein_coding	HGNC Symbol	5	SBDS, ribosome maturation factor [Source:HGNC Symbol;Acc:HGNC:19440]	2.560192
ENSG00000115556	84812	PLCD4	protein_coding	HGNC Symbol	15	phospholipase C delta 4 [Source:HGNC Symbol;Acc:HGNC:9062]	2.560273
ENSG00000260269	NA	AC105036.3	processed_transcript	Clone-based (Ensembl) gene	4	novel transcript	2.560813
ENSG00000215199	NA	YWHAZP6	processed_pseudogene	HGNC Symbol	1	tyrosine 3-monooxygenase/tryptophan 5-monooxygenase activation protein zeta pseudogene	2.561138
ENSG00000228809	NA	AL034428.1	processed_transcript	Clone-based (Ensembl) gene	2	novel transcript, antisense to SNRPB2	2.561702
ENSG00000152443	284309	ZNF776	protein_coding	HGNC Symbol	5	zinc finger protein 776 [Source:HGNC Symbol;Acc:HGNC:26765]	2.561926
ENSG00000230005	NA	SNAP47-AS1	antisense	HGNC Symbol	1	SNAP47 antisense RNA 1 [Source:HGNC Symbol;Acc:HGNC:41501]	2.562531
ENSG00000273151	NA	AC073957.3	antisense	Clone-based (Ensembl) gene	1	novel transcript, antisense to GET4	2.563012
ENSG00000143387	1513	CTSK	protein_coding	HGNC Symbol	3	cathepsin K [Source:HGNC Symbol;Acc:HGNC:2536]	2.56356
ENSG00000225073	7919	DDX39B	protein_coding	HGNC Symbol	22	DExD-box helicase 39B [Source:HGNC Symbol;Acc:HGNC:13917]	2.566101
ENSG00000267491	NA	AC100788.1	antisense	Clone-based (Ensembl) gene	1	novel transcript, antisense to TIMP2	2.56706
ENSG00000269946	NA	MIRLET7A1HG	lincRNA	HGNC Symbol	1	miRlet-7a-1/let-7f-1/let-7d cluster host gene [Source:HGNC Symbol;Acc:HGNC:53970]	2.56724
ENSG00000205085	346653	FAM71F2	protein_coding	HGNC Symbol	7	family with sequence similarity 71 member F2 [Source:HGNC Symbol;Acc:HGNC:27998]	2.568474
ENSG00000175105	55279	ZNF654	protein_coding	HGNC Symbol	4	zinc finger protein 654 [Source:HGNC Symbol;Acc:HGNC:25612]	2.568776
ENSG00000239503	NA	MARK2P8	processed_pseudogene	HGNC Symbol	1	microtubule affinity regulating kinase 2 pseudogene 8 [Source:HGNC Symbol;Acc:HGNC:3979	2.569176
ENSG00000166889	219988	PATL1	protein_coding	HGNC Symbol	2	PAT1 homolog 1, processing body mRNA decay factor [Source:HGNC Symbol;Acc:HGNC:2672	2.569519
ENSG00000212534	692110	SNORD70	snoRNA	HGNC Symbol	1	small nucleolar RNA, C/D box 70 [Source:HGNC Symbol;Acc:HGNC:32731]	2.570282
ENSG00000221883	646450	ARIH2OS	protein_coding	HGNC Symbol	2	ariadne RBR E3 ubiquitin protein ligase 2 opposite strand [Source:HGNC Symbol;Acc:HGNC:34	2.571224
ENSG00000124134	3787	KCNS1	protein_coding	HGNC Symbol	2	potassium voltage-gated channel modifier subfamily S member 1 [Source:HGNC Symbol;Acc:H	2.572112
ENSG00000116514	127544	RNF19B	protein_coding	HGNC Symbol	3	ring finger protein 19B [Source:HGNC Symbol;Acc:HGNC:26886]	2.572664
ENSG00000197808	92283	ZNF461	protein_coding	HGNC Symbol	14	zinc finger protein 461 [Source:HGNC Symbol;Acc:HGNC:21629]	2.572814
ENSG00000100311	5155	PDGFB	protein_coding	HGNC Symbol	4	platelet derived growth factor subunit B [Source:HGNC Symbol;Acc:HGNC:8800]	2.574787
ENSG00000223461	NA	AC004471.1	antisense	Clone-based (Ensembl) gene	1	novel transcript	2.574978
ENSG00000269243	NA	AC008894.2	antisense	Clone-based (Ensembl) gene	2	novel transcript, antisense RAB8A	2.575897
ENSG00000257474	NA	AC027288.1	lincRNA	Clone-based (Ensembl) gene	3	novel transcript	2.576856
ENSG00000073756	5743	PTGS2	protein_coding	HGNC Symbol	5	prostaglandin-endoperoxide synthase 2 [Source:HGNC Symbol;Acc:HGNC:9605]	2.577204
ENSG00000131115	7770	ZNF227	protein_coding	HGNC Symbol	13	zinc finger protein 227 [Source:HGNC Symbol;Acc:HGNC:13020]	2.578474
ENSG00000273447	NA	AC004067.1	antisense	Clone-based (Ensembl) gene	1	novel transcript, antisense to CASP6	2.58156
ENSG00000235079	NA	ZRANB2-AS1	antisense	HGNC Symbol	2	ZRANB2 antisense RNA 1 [Source:HGNC Symbol;Acc:HGNC:43594]	2.583729
ENSG00000173926	115123	3-Mar	protein_coding	HGNC Symbol	5	membrane associated ring-CH-type finger 3 [Source:HGNC Symbol;Acc:HGNC:28728]	2.584111
ENSG00000236199	NA	AL359076.1	antisense	Clone-based (Ensembl) gene	1	novel transcript	2.584406
ENSG00000234883	114614	MIR155HG	lincRNA	HGNC Symbol	1	MIR155 host gene [Source:HGNC Symbol;Acc:HGNC:35460]	2.585748
ENSG00000172602	27289	RND1	protein_coding	HGNC Symbol	6	Rho family GTPase 1 [Source:HGNC Symbol;Acc:HGNC:18314]	2.587311
ENSG00000131503	54882	ANKHD1	protein_coding	HGNC Symbol	21	ankyrin repeat and KH domain containing 1 [Source:HGNC Symbol;Acc:HGNC:24714]	2.591225

ENSG00000232748	NA	AC135050.1	lincRNA	Clone-based (Ensembl) gene	2	novel transcript, antisense to ZNF668	2.59269
ENSG00000240160	NA	RN7SL263P	misc_RNA	HGNC Symbol	1	RNA, 7SL, cytoplasmic 263, pseudogene [Source:HGNC Symbol;Acc:HGNC:46279]	2.593121
ENSG00000261468	NA	AC096921.2	sense_overlapping	Clone-based (Ensembl) gene	1	novel transcript, overlapping to TGFBR2	2.594098
ENSG00000183023	6546	SLC8A1	protein_coding	HGNC Symbol	12	solute carrier family 8 member A1 [Source:HGNC Symbol;Acc:HGNC:11068]	2.594922
ENSG00000264885	NA	AC026271.3	sense_intronic	Clone-based (Ensembl) gene	1	novel transcript	2.596972
ENSG00000196466	90576	ZNF799	protein_coding	HGNC Symbol	4	zinc finger protein 799 [Source:HGNC Symbol;Acc:HGNC:28071]	2.597677
ENSG00000163877	79753	SNIP1	protein_coding	HGNC Symbol	4	Smad nuclear interacting protein 1 [Source:HGNC Symbol;Acc:HGNC:30587]	2.598012
ENSG00000118620	80264	ZNF430	protein_coding	HGNC Symbol	6	zinc finger protein 430 [Source:HGNC Symbol;Acc:HGNC:20808]	2.599174
ENSG00000141469	6563	SLC14A1	protein_coding	HGNC Symbol	20	solute carrier family 14 member 1 (Kidd blood group) [Source:HGNC Symbol;Acc:HGNC:1091	2.601167
ENSG00000142528	25888	ZNF473	protein_coding	HGNC Symbol	9	zinc finger protein 473 [Source:HGNC Symbol;Acc:HGNC:23239]	2.601319
ENSG00000234545	257415	FAM133B	protein_coding	HGNC Symbol	11	family with sequence similarity 133 member B [Source:HGNC Symbol;Acc:HGNC:28629]	2.602659
ENSG00000270429	NA	KNOP1P2	processed_pseudogene	HGNC Symbol	1	lysine rich nucleolar protein 1 pseudogene 2 [Source:HGNC Symbol;Acc:HGNC:48920]	2.603752
ENSG00000180610	NA	ZBTB12BP	processed_pseudogene	HGNC Symbol	1	zinc finger and BTB domain containing 12B, pseudogene [Source:HGNC Symbol;Acc:HGNC:37	2.604452
ENSG00000276523	NA	AC025287.3	antisense	Clone-based (Ensembl) gene	1	novel transcript, antisense to GABARAPL2	2.604574
ENSG00000249628	1E+08	LINC00942	lincRNA	HGNC Symbol	1	long intergenic non-protein coding RNA 942 [Source:HGNC Symbol;Acc:HGNC:48636]	2.605268
ENSG00000261527	NA	AC026464.5	processed_pseudogene	Clone-based (Ensembl) gene	1	transmembrane protein 111 (TMEM111) pseudogene	2.605725
ENSG00000232970	NA	POLHP1	processed_pseudogene	HGNC Symbol	1	DNA polymerase eta pseudogene 1 [Source:HGNC Symbol;Acc:HGNC:43967]	2.610117
ENSG00000169946	23414	ZFPM2	protein_coding	HGNC Symbol	10	zinc finger protein, FOG family member 2 [Source:HGNC Symbol;Acc:HGNC:16700]	2.610411
ENSG00000170631	7564	ZNF16	protein_coding	HGNC Symbol	9	zinc finger protein 16 [Source:HGNC Symbol;Acc:HGNC:12947]	2.61144
ENSG00000199890	NA	RF00019	misc_RNA	RFAM	1		2.611866
ENSG00000113248	56121	PCDHB15	protein_coding	HGNC Symbol	2	protocadherin beta 15 [Source:HGNC Symbol;Acc:HGNC:8686]	2.612496
ENSG00000278243	7264	TSTA3	protein_coding	HGNC Symbol	16	tissue specific transplantation antigen P35B [Source:HGNC Symbol;Acc:HGNC:12390]	2.617547
ENSG00000225581	NA	TRIM53AP	unprocessed_pseudogene	HGNC Symbol	1	tripartite motif containing 53A, pseudogene [Source:HGNC Symbol;Acc:HGNC:19025]	2.617996
ENSG00000235145	NA	RPSAP16	processed_pseudogene	HGNC Symbol	1	ribosomal protein SA pseudogene 16 [Source:HGNC Symbol;Acc:HGNC:35478]	2.618062
ENSG00000083937	25978	CHMP2B	protein_coding	HGNC Symbol	5	charged multivesicular body protein 2B [Source:HGNC Symbol;Acc:HGNC:24537]	2.618325
ENSG00000260213	NA	AC092718.2	antisense	Clone-based (Ensembl) gene	4		2.618842
ENSG00000116954	64121	RRAGC	protein_coding	HGNC Symbol	4	Ras related GTP binding C [Source:HGNC Symbol;Acc:HGNC:19902]	2.621091
ENSG00000163638	56999	ADAMTS9	protein_coding	HGNC Symbol	10	ADAM metallopeptidase with thrombospondin type 1 motif 9 [Source:HGNC Symbol;Acc:HGN	2.621435
ENSG00000179119	144108	SPTY2D1	protein_coding	HGNC Symbol	3	SPT2 chromatin protein domain containing 1 [Source:HGNC Symbol;Acc:HGNC:26818]	2.624134
ENSG00000140600	6457	SH3GL3	protein_coding	HGNC Symbol	6	SH3 domain containing GRB2 like 3, endophilin A3 [Source:HGNC Symbol;Acc:HGNC:10832]	2.625074
ENSG00000275090	NA	RN7SL296P	misc_RNA	HGNC Symbol	1	RNA, 7SL, cytoplasmic 296, pseudogene [Source:HGNC Symbol;Acc:HGNC:46312]	2.6258
ENSG00000088448	55608	ANKRD10	protein_coding	HGNC Symbol	11	ankyrin repeat domain 10 [Source:HGNC Symbol;Acc:HGNC:20265]	2.628167
ENSG00000241120	NA	HMGN1P8	processed_pseudogene	HGNC Symbol	1	high mobility group nucleosome binding domain 1 pseudogene 8 [Source:HGNC Symbol;Acc:H	2.630822
ENSG00000133398	84246	MED10	protein_coding	HGNC Symbol	3	mediator complex subunit 10 [Source:HGNC Symbol;Acc:HGNC:28760]	2.630882
ENSG00000259126	NA	AL161752.1	lincRNA	Clone-based (Ensembl) gene	2	novel transcript	2.631047
ENSG00000118412	9994	CASP8AP2	protein_coding	HGNC Symbol	6	caspase 8 associated protein 2 [Source:HGNC Symbol;Acc:HGNC:1510]	2.631518
ENSG00000179546	3352	HTR1D	protein_coding	HGNC Symbol	1	5-hydroxytryptamine receptor 1D [Source:HGNC Symbol;Acc:HGNC:5289]	2.632234
ENSG00000163435	1999	ELF3	protein_coding	HGNC Symbol	10	E74 like ETS transcription factor 3 [Source:HGNC Symbol;Acc:HGNC:3318]	2.632381
ENSG00000278000	NA	AC139100.2	sense_intronic	Clone-based (Ensembl) gene	1	novel transcript	2.633506
ENSG00000139112	23710	GABARAPL1	protein_coding	HGNC Symbol	19	GABA type A receptor associated protein like 1 [Source:HGNC Symbol;Acc:HGNC:4068]	2.637796
ENSG00000225891	NA	AL513365.2	antisense	Clone-based (Ensembl) gene	1		2.641035
ENSG00000202515	56662	VTRNA1-3	misc_RNA	HGNC Symbol	1	vault RNA 1-3 [Source:HGNC Symbol;Acc:HGNC:12656]	2.643619
ENSG00000088356	81572	PDRG1	protein_coding	HGNC Symbol	1	p53 and DNA damage regulated 1 [Source:HGNC Symbol;Acc:HGNC:16119]	2.643835
ENSG00000254328	NA	AC008429.4	processed_pseudogene	Clone-based (Ensembl) gene	1	coiled-coil domain containing 72 (CCDC72) pseudogene	2.644061
ENSG00000229207	NA	SERPINH1P1	processed_pseudogene	HGNC Symbol	1	serpin family H member 1 pseudogene 1 [Source:HGNC Symbol;Acc:HGNC:19917]	2.644781
ENSG00000177173	NA	NAP1L4P1	processed_pseudogene	HGNC Symbol	1	nucleosome assembly protein 1 like 4 pseudogene 1 [Source:HGNC Symbol;Acc:HGNC:39740]	2.646904
ENSG00000155714	255762	PDZD9	protein_coding	HGNC Symbol	3	PDZ domain containing 9 [Source:HGNC Symbol;Acc:HGNC:28740]	2.647183
ENSG00000149972	53942	CNTN5	protein_coding	HGNC Symbol	12	contactin 5 [Source:HGNC Symbol;Acc:HGNC:2175]	2.649301
ENSG00000178502	55175	KLHL11	protein_coding	HGNC Symbol	1	kelch like family member 11 [Source:HGNC Symbol;Acc:HGNC:19008]	2.650498
ENSG00000162642	148423	C1orf52	protein_coding	HGNC Symbol	4	chromosome 1 open reading frame 52 [Source:HGNC Symbol;Acc:HGNC:24871]	2.652323
ENSG00000272054	NA	AC007390.2	sense_intronic	Clone-based (Ensembl) gene	1	novel transcript	2.657175
ENSG00000249249	NA	AC010226.1	antisense	Clone-based (Ensembl) gene	3		2.657694
ENSG00000107736	64072	CDH23	protein_coding	HGNC Symbol	19	cadherin related 23 [Source:HGNC Symbol;Acc:HGNC:13733]	2.657965
ENSG00000228463	NA	AP006222.1	transcribed_processed_pseudogene	Clone-based (Ensembl) gene	6	ribosomal protein L23a (RPL23A) pseudogene	2.660514
ENSG00000100109	24144	TFIP11	protein_coding	HGNC Symbol	17	tuftelin interacting protein 11 [Source:HGNC Symbol;Acc:HGNC:17165]	2.66058
ENSG00000116604	4209	MEF2D	protein_coding	HGNC Symbol	7	myocyte enhancer factor 2D [Source:HGNC Symbol;Acc:HGNC:6997]	2.661152
ENSG00000230551	NA	AC021078.1	processed_transcript	Clone-based (Ensembl) gene	1	novel transcript	2.66133
ENSG00000055070	26099	SZRD1	protein_coding	HGNC Symbol	10	SUZ RNA binding domain containing 1 [Source:HGNC Symbol;Acc:HGNC:30232]	2.663139
ENSG00000253336	NA	AC018992.1	processed_pseudogene	Clone-based (Ensembl) gene	1	hydroxysteroid (17-beta) dehydrogenase 4 (HSD17B4) pseudogene	2.664527
ENSG00000231587	692093	SNORD62B	snoRNA	HGNC Symbol	1	small nucleolar RNA, C/D box 62B [Source:HGNC Symbol;Acc:HGNC:23031]	2.664771
ENSG00000225190	9842	PLEKHM1	protein_coding	HGNC Symbol	18	pleckstrin homology and RUN domain containing M1 [Source:HGNC Symbol;Acc:HGNC:29017	2.664954
ENSG00000276148	NA	AC084824.5	antisense	Clone-based (Ensembl) gene	1	novel transcript, antisense to DNM1L	2.666871
ENSG00000114757	51555	PEX5L	protein_coding	HGNC Symbol	18	peroxisomal biogenesis factor 5 like [Source:HGNC Symbol;Acc:HGNC:30024]	2.671792
ENSG00000130449	57688	ZSWIM6	protein_coding	HGNC Symbol	1	zinc finger SWIM-type containing 6 [Source:HGNC Symbol;Acc:HGNC:29316]	2.673104
ENSG00000116698	9887	SMG7	protein_coding	HGNC Symbol	16	SMG7, nonsense mediated mRNA decay factor [Source:HGNC Symbol;Acc:HGNC:16792]	2.674579

ENSG00000236824	618	BCYRN1	scRNA	HGNC Symbol	1	brain cytoplasmic RNA 1 [Source:HGNC Symbol;Acc:HGNC:1022]	2.67688
ENSG00000099812	126353	MISP	protein_coding	HGNC Symbol	2	mitotic spindle positioning [Source:HGNC Symbol;Acc:HGNC:27000]	2.678492
ENSG00000237810	NA	AC104852.1	processed_pseudogene	Clone-based (Ensembl) gene	1	polo-like kinase 1 (Drosophila) (PLK1) pseudogene	2.682206
ENSG00000271933	NA	AL603756.1	antisense	Clone-based (Ensembl) gene	1	novel transcript, antisense to GHITM	2.682756
ENSG00000117360	9129	PRPF3	protein_coding	HGNC Symbol	7	pre-mRNA processing factor 3 [Source:HGNC Symbol;Acc:HGNC:17348]	2.687342
ENSG00000276603	NA	AL109614.1	sense_intronic	Clone-based (Ensembl) gene	1	novel transcript, sense intronic to BLCAP	2.68757
ENSG00000173846	1263	PLK3	protein_coding	HGNC Symbol	7	polo like kinase 3 [Source:HGNC Symbol;Acc:HGNC:2154]	2.687954
ENSG00000263823	NA	AC009831.1	antisense	Clone-based (Ensembl) gene	1	novel transcript, antisense to KIAA1012	2.689058
ENSG00000227036	1E+08	LINC00511	processed_transcript	HGNC Symbol	106	long intergenic non-protein coding RNA 511 [Source:HGNC Symbol;Acc:HGNC:43564]	2.689267
ENSG00000227036	400619	LINC00511	processed_transcript	HGNC Symbol	106	long intergenic non-protein coding RNA 511 [Source:HGNC Symbol;Acc:HGNC:43564]	2.689267
ENSG00000133997	10001	MED6	protein_coding	HGNC Symbol	12	mediator complex subunit 6 [Source:HGNC Symbol;Acc:HGNC:19970]	2.689273
ENSG00000205323	84324	SARNP	protein_coding	HGNC Symbol	6	SAP domain containing ribonucleoprotein [Source:HGNC Symbol;Acc:HGNC:24432]	2.692558
ENSG00000148426	254427	PROSER2	protein_coding	HGNC Symbol	5	proline and serine rich 2 [Source:HGNC Symbol;Acc:HGNC:23728]	2.692906
ENSG00000142396	NA	ERVK3-1	protein_coding	HGNC Symbol	8	endogenous retrovirus group K3 member 1 [Source:HGNC Symbol;Acc:HGNC:30466]	2.695011
ENSG00000250033	641364	SLC7A11-AS1	processed_transcript	HGNC Symbol	5	SLC7A11 antisense RNA 1 [Source:HGNC Symbol;Acc:HGNC:44064]	2.696039
ENSG00000149243	283212	KLHL35	protein_coding	HGNC Symbol	5	kelch like family member 35 [Source:HGNC Symbol;Acc:HGNC:26597]	2.698038
ENSG00000186866	23275	POFUT2	protein_coding	HGNC Symbol	14	protein O-fucosyltransferase 2 [Source:HGNC Symbol;Acc:HGNC:14683]	2.701783
ENSG00000260488	NA	AC007216.1	antisense	Clone-based (Ensembl) gene	1	novel transcript, antisense to RUNDC2A	2.702418
ENSG00000261604	NA	AC114947.2	antisense	Clone-based (Ensembl) gene	2	novel transcript, antisense to HMGCS1	2.705434
ENSG00000260751	NA	AC008870.2	sense_intronic	Clone-based (Ensembl) gene	1	novel transcript	2.708269
ENSG00000250764	NA	AC025178.1	antisense	Clone-based (Ensembl) gene	1	novel transcript	2.710005
ENSG00000277151	NA	AL138820.1	lincRNA	Clone-based (Ensembl) gene	1	novel transcript	2.711186
ENSG00000176994	140775	SMCR8	protein_coding	HGNC Symbol	1	Smith-Magenis syndrome chromosome region, candidate 8 [Source:HGNC Symbol;Acc:HGNC:	2.71405
ENSG00000070190	27071	DAPP1	protein_coding	HGNC Symbol	4	dual adaptor of phosphotyrosine and 3-phosphoinositides 1 [Source:HGNC Symbol;Acc:HGNC	2.716505
ENSG00000253754	NA	AC087620.1	antisense	Clone-based (Ensembl) gene	1	novel transcript	2.720225
ENSG00000135914	3357	HTR2B	protein_coding	HGNC Symbol	1	5-hydroxytryptamine receptor 2B [Source:HGNC Symbol;Acc:HGNC:5294]	2.720606
ENSG00000273302	NA	AC016747.3	sense_intronic	Clone-based (Ensembl) gene	1	novel transcript	2.72189
ENSG00000264546	NA	AC008026.3	sense_intronic	Clone-based (Ensembl) gene	1	novel transcript	2.723571
ENSG00000225721	NA	AL592166.1	antisense	Clone-based (Ensembl) gene	2	novel transcript	2.729029
ENSG00000139926	122786	FRMD6	protein_coding	HGNC Symbol	18	FERM domain containing 6 [Source:HGNC Symbol;Acc:HGNC:19839]	2.734621
ENSG00000134256	9398	CD101	protein_coding	HGNC Symbol	4	CD101 molecule [Source:HGNC Symbol;Acc:HGNC:5949]	2.737259
ENSG00000225683	NA	PACRG-AS3	antisense	HGNC Symbol	6	PACRG antisense RNA 3 [Source:HGNC Symbol;Acc:HGNC:52053]	2.737659
ENSG00000261692	NA	AC092120.1	sense_intronic	Clone-based (Ensembl) gene	1	novel transcript	2.738443
ENSG00000109072	7448	VTN	protein_coding	HGNC Symbol	3	vitronectin [Source:HGNC Symbol;Acc:HGNC:12724]	2.739105
ENSG00000115568	7701	ZNF142	protein_coding	HGNC Symbol	7	zinc finger protein 142 [Source:HGNC Symbol;Acc:HGNC:12927]	2.74015
ENSG00000137193	5292	PIM1	protein_coding	HGNC Symbol	3	Pim-1 proto-oncogene, serine/threonine kinase [Source:HGNC Symbol;Acc:HGNC:8986]	2.740525
ENSG00000168314	4336	MOBP	protein_coding	HGNC Symbol	13	myelin-associated oligodendrocyte basic protein [Source:HGNC Symbol;Acc:HGNC:7189]	2.743045
ENSG00000125657	8744	TNFSF9	protein_coding	HGNC Symbol	1	TNF superfamily member 9 [Source:HGNC Symbol;Acc:HGNC:11939]	2.743203
ENSG00000235677	NA	NPM1P26	processed_pseudogene	HGNC Symbol	1	nucleophosmin 1 pseudogene 26 [Source:HGNC Symbol;Acc:HGNC:45205]	2.744677
ENSG00000154898	NA	CCDC144CP	transcribed_processed_pseudogene	HGNC Symbol	3	coiled-coil domain containing 144C, pseudogene [Source:HGNC Symbol;Acc:HGNC:29073]	2.746096
ENSG00000171617	8507	ENC1	protein_coding	HGNC Symbol	7	ectodermal-neural cortex 1 [Source:HGNC Symbol;Acc:HGNC:3345]	2.747072
ENSG00000261526	NA	AC012615.1	lincRNA	Clone-based (Ensembl) gene	1	novel transcript	2.747129
ENSG00000206612	677793	SNORA2A	snoRNA	HGNC Symbol	1	small nucleolar RNA, H/ACA box 2A [Source:HGNC Symbol;Acc:HGNC:32584]	2.750359
ENSG00000227176	NA	AC092641.1	processed_pseudogene	Clone-based (Ensembl) gene	1	TGF beta-inducible nuclear protein 1 (TINP1) pseudogene	2.751499
ENSG00000129152	4654	MYOD1	protein_coding	HGNC Symbol	1	myogenic differentiation 1 [Source:HGNC Symbol;Acc:HGNC:7611]	2.753531
ENSG00000127993	84060	RBM48	protein_coding	HGNC Symbol	3	RNA binding motif protein 48 [Source:HGNC Symbol;Acc:HGNC:21785]	2.755103
ENSG00000012963	55148	UBR7	protein_coding	HGNC Symbol	7	ubiquitin protein ligase E3 component n-recognin 7 (putative) [Source:HGNC Symbol;Acc:HGN	2.757829
ENSG00000162998	2487	FRZB	protein_coding	HGNC Symbol	1	frizzled related protein [Source:HGNC Symbol;Acc:HGNC:3959]	2.757909
ENSG00000242041	NA	RPL35AP28	processed_pseudogene	HGNC Symbol	1	ribosomal protein L35a pseudogene 28 [Source:HGNC Symbol;Acc:HGNC:36480]	2.763659
ENSG00000165006	51271	UBAP1	protein_coding	HGNC Symbol	5	ubiquitin associated protein 1 [Source:HGNC Symbol;Acc:HGNC:12461]	2.764716
ENSG00000164161	64399	HHIP	protein_coding	HGNC Symbol	8	hedgehog interacting protein [Source:HGNC Symbol;Acc:HGNC:14866]	2.765056
ENSG00000165323	120114	FAT3	protein_coding	HGNC Symbol	6	FAT atypical cadherin 3 [Source:HGNC Symbol;Acc:HGNC:23112]	2.765232
ENSG00000162419	10691	GMEB1	protein_coding	HGNC Symbol	4	glucocorticoid modulatory element binding protein 1 [Source:HGNC Symbol;Acc:HGNC:4370]	2.765295
ENSG00000151014	25819	NOCT	protein_coding	HGNC Symbol	3	nocturnin [Source:HGNC Symbol;Acc:HGNC:14254]	2.767226
ENSG00000212452	692109	SNORD69	snoRNA	HGNC Symbol	1	small nucleolar RNA, C/D box 69 [Source:HGNC Symbol;Acc:HGNC:32730]	2.767261
ENSG00000239218	NA	RPS20P22	transcribed_processed_pseudogene	HGNC Symbol	2	ribosomal protein S20 pseudogene 22 [Source:HGNC Symbol;Acc:HGNC:36379]	2.772402
ENSG00000253372	NA	AC016405.1	sense_intronic	Clone-based (Ensembl) gene	2	novel transcript	2.772567
ENSG00000235236	NA	AC137630.2	antisense	Clone-based (Ensembl) gene	2	novel transcript	2.772821
ENSG00000179772	2307	FOXS1	protein_coding	HGNC Symbol	1	forkhead box S1 [Source:HGNC Symbol;Acc:HGNC:3735]	2.773077
ENSG00000265148	1.01E+08	TSPOAP1-AS1	antisense	HGNC Symbol	11	TSPOAP1, SUPT4H1 and RNF43 antisense RNA 1 [Source:HGNC Symbol;Acc:HGNC:44148]	2.775236
ENSG00000239636	NA	AC004865.2	antisense	Clone-based (Ensembl) gene	2		2.776195
ENSG00000261669	NA	AC008731.1	antisense	Clone-based (Ensembl) gene	1	novel transcript, antisense to TNRC6A	2.780461
ENSG00000258891	NA	AC005480.1	antisense	Clone-based (Ensembl) gene	2	novel transcript, antisense to ZNF410	2.780694
ENSG00000169016	1876	E2F6	protein_coding	HGNC Symbol	12	E2F transcription factor 6 [Source:HGNC Symbol;Acc:HGNC:3120]	2.78123
ENSG00000269570	NA	AP001350.1	antisense	Clone-based (Ensembl) gene	1	novel transcript, antisense to ZFP91	2.781765

ENSG00000272072	NA	AC004492.1	antisense	Clone-based (Ensembl) gene	1	novel transcript, antisense to HBP1	2.782598
ENSG00000103429	51283	BFAR	protein_coding	HGNC Symbol	12	bifunctional apoptosis regulator [Source:HGNC Symbol;Acc:HGNC:17613]	2.788668
ENSG00000070495	23210	JMJD6	protein_coding	HGNC Symbol	8	jumonji domain containing 6, arginine demethylase and lysine hydroxylase [Source:HGNC Sym	2.791679
ENSG00000256087	9668	ZNF432	protein_coding	HGNC Symbol	7	zinc finger protein 432 [Source:HGNC Symbol;Acc:HGNC:20810]	2.791821
ENSG00000214189	NA	ZNF788P	transcribed_unprocessed_pseudogene	HGNC Symbol	6	zinc finger family member 788, pseudogene [Source:HGNC Symbol;Acc:HGNC:33112]	2.79396
ENSG00000278576	NA	AL162171.3	sense_intronic	Clone-based (Ensembl) gene	1	novel transcript, sense intronic to ZC3H14	2.796707
ENSG00000156232	123720	WHAMM	protein_coding	HGNC Symbol	2	WAS protein homolog associated with actin, golgi membranes and microtubules [Source:HGN	2.797243
ENSG00000138650	57575	PCDH10	protein_coding	HGNC Symbol	3	protocadherin 10 [Source:HGNC Symbol;Acc:HGNC:13404]	2.798289
ENSG00000236184	NA	TCEA1P4	processed_pseudogene	HGNC Symbol	1	transcription elongation factor A1 pseudogene 4 [Source:HGNC Symbol;Acc:HGNC:31091]	2.798691
ENSG00000189350	165186	TOGARAM2	protein_coding	HGNC Symbol	7	TOG array regulator of axonemal microtubules 2 [Source:HGNC Symbol;Acc:HGNC:33715]	2.799726
ENSG00000275056	NA	AC020663.3	sense_intronic	Clone-based (Ensembl) gene	1	novel transcript, sense intronic to GLYR1	2.80234
ENSG00000106245	8896	BUD31	protein_coding	HGNC Symbol	9	BUD31 homolog [Source:HGNC Symbol;Acc:HGNC:29629]	2.803964
ENSG00000266897	NA	AC005546.1	lincRNA	Clone-based (Ensembl) gene	1	novel transcript	2.804009
ENSG00000221491	677815	SNORA2C	snoRNA	HGNC Symbol	1	small nucleolar RNA, H/ACA box 2C [Source:HGNC Symbol;Acc:HGNC:32624]	2.804746
ENSG00000163545	81788	NUAK2	protein_coding	HGNC Symbol	1	NUAK family kinase 2 [Source:HGNC Symbol;Acc:HGNC:29558]	2.817247
ENSG00000128242	9514	GAL3ST1	protein_coding	HGNC Symbol	22	galactose-3-O-sulfotransferase 1 [Source:HGNC Symbol;Acc:HGNC:24240]	2.819228
ENSG00000166676	780776	TVP23A	protein_coding	HGNC Symbol	12	trans-golgi network vesicle protein 23 homolog A [Source:HGNC Symbol;Acc:HGNC:20398]	2.819453
ENSG00000145888	2741	GLRA1	protein_coding	HGNC Symbol	4	glycine receptor alpha 1 [Source:HGNC Symbol;Acc:HGNC:4326]	2.820989
ENSG00000100796	55671	PPP4R3A	protein_coding	HGNC Symbol	12	protein phosphatase 4 regulatory subunit 3A [Source:HGNC Symbol;Acc:HGNC:20219]	2.82251
ENSG00000247982	283663	LINC00926	lincRNA	HGNC Symbol	4	long intergenic non-protein coding RNA 926 [Source:HGNC Symbol;Acc:HGNC:27514]	2.822718
ENSG00000117281	11126	CD160	protein_coding	HGNC Symbol	5	CD160 molecule [Source:HGNC Symbol;Acc:HGNC:17013]	2.824776
ENSG00000260060	NA	AC009088.1	antisense	Clone-based (Ensembl) gene	1	novel transcript, antisense to PYCARD	2.827477
ENSG00000185246	55015	PRPF39	protein_coding	HGNC Symbol	12	pre-mRNA processing factor 39 [Source:HGNC Symbol;Acc:HGNC:20314]	2.827771
ENSG00000235440	NA	AC011742.3	processed_pseudogene	Clone-based (Ensembl) gene	1	ribosomal protein S2 (RPS2) pseudogene	2.828661
ENSG00000141965	55527	FEM1A	protein_coding	HGNC Symbol	1	fem-1 homolog A [Source:HGNC Symbol;Acc:HGNC:16934]	2.835587
ENSG00000265179	NA	AP000894.2	processed_transcript	Clone-based (Ensembl) gene	4	novel transcript, antisense to ADCYAP1	2.838181
ENSG00000256826	NA	ATP5MFP4	processed_pseudogene	HGNC Symbol	1	ATP synthase membrane subunit f pseudogene 4 [Source:HGNC Symbol;Acc:HGNC:32451]	2.839275
ENSG00000207119	NA	RF00012	snoRNA	RFAM	1		2.840072
ENSG00000253667	NA	AC100821.1	processed_pseudogene	Clone-based (Ensembl) gene	1	ESF1, nucleolar pre-rRNA processing protein, homolog (S. cerevisiae) (ESF1) pseudogene	2.840887
ENSG00000185065	NA	AC000068.1	antisense	Clone-based (Ensembl) gene	1	novel transcript, antisense to C22orf39	2.840968
ENSG00000257315	1E+08	ZBED6	protein_coding	HGNC Symbol	2	zinc finger BED-type containing 6 [Source:HGNC Symbol;Acc:HGNC:33273]	2.844814
ENSG00000100483	79609	VCPKMT	protein_coding	HGNC Symbol	5	valosin containing protein lysine methyltransferase [Source:HGNC Symbol;Acc:HGNC:20352]	2.845199
ENSG00000206532	NA	AC117402.1	lincRNA	Clone-based (Ensembl) gene	2	novel transcript	2.847007
ENSG00000258323	NA	AC073575.1	antisense	Clone-based (Ensembl) gene	1	novel transcript, antisense to NAA25	2.848767
ENSG00000213188	NA	YBX1P4	processed_pseudogene	HGNC Symbol	1	Y-box binding protein 1 pseudogene 4 [Source:HGNC Symbol;Acc:HGNC:42425]	2.849319
ENSG00000134760	1828	DSG1	protein_coding	HGNC Symbol	2	desmoglein 1 [Source:HGNC Symbol;Acc:HGNC:3048]	2.850516
ENSG00000011114	55727	BTBD7	protein_coding	HGNC Symbol	8	BTB domain containing 7 [Source:HGNC Symbol;Acc:HGNC:18269]	2.850835
ENSG00000221500	594838	SNORD100	snoRNA	HGNC Symbol	1	small nucleolar RNA, C/D box 100 [Source:HGNC Symbol;Acc:HGNC:32763]	2.854901
ENSG00000256683	59348	ZNF350	protein_coding	HGNC Symbol	9	zinc finger protein 350 [Source:HGNC Symbol;Acc:HGNC:16656]	2.85763
ENSG00000047662	27146	FAM184B	protein_coding	HGNC Symbol	1	family with sequence similarity 184 member B [Source:HGNC Symbol;Acc:HGNC:29235]	2.857882
ENSG00000267560	NA	AC027514.2	antisense	Clone-based (Ensembl) gene	1	novel transcript, antisense to KIAA1468	2.858253
ENSG00000207181	677802	SNORA14B	snoRNA	HGNC Symbol	1	small nucleolar RNA, H/ACA box 14B [Source:HGNC Symbol;Acc:HGNC:32603]	2.863335
ENSG00000088826	54498	SMOX	protein_coding	HGNC Symbol	12	spermine oxidase [Source:HGNC Symbol;Acc:HGNC:15862]	2.863756
ENSG00000242602	NA	AC008953.1	processed_pseudogene	Clone-based (Ensembl) gene	1	ribosomal protein L35a (RPL35A) pseudogene	2.865276
ENSG00000260648	NA	AC020658.3	sense_intronic	Clone-based (Ensembl) gene	1	novel transcript, sense intronic to PAK6	2.866563
ENSG00000169981	7584	ZNF35	protein_coding	HGNC Symbol	6	zinc finger protein 35 [Source:HGNC Symbol;Acc:HGNC:13099]	2.869278
ENSG00000179168	199720	GGN	protein_coding	HGNC Symbol	5	gametogenetin [Source:HGNC Symbol;Acc:HGNC:18869]	2.869822
ENSG00000213842	NA	SUGT1P2	processed_pseudogene	HGNC Symbol	1	SUGT1 pseudogene 2 [Source:HGNC Symbol;Acc:HGNC:31377]	2.873953
ENSG00000165935	341346	SMCO2	protein_coding	HGNC Symbol	6	single-pass membrane protein with coiled-coil domains 2 [Source:HGNC Symbol;Acc:HGNC:34	2.873978
ENSG00000167130	57171	DOLPP1	protein_coding	HGNC Symbol	5	dolichyldiphosphatase 1 [Source:HGNC Symbol;Acc:HGNC:29565]	2.878888
ENSG00000255566	NA	AC135279.1	processed_pseudogene	Clone-based (Ensembl) gene	1	mitochondrial ribosomal protein S25 (MRPS25) pseudogene	2.881103
ENSG00000130783	84660	CCDC62	protein_coding	HGNC Symbol	6	coiled-coil domain containing 62 [Source:HGNC Symbol;Acc:HGNC:30723]	2.882124
ENSG00000100031	2678	GGT1	protein_coding	HGNC Symbol	27	gamma-glutamyltransferase 1 [Source:HGNC Symbol;Acc:HGNC:4250]	2.88264
ENSG00000213453	NA	FTH1P3	processed_pseudogene	HGNC Symbol	1	ferritin heavy chain 1 pseudogene 3 [Source:HGNC Symbol;Acc:HGNC:3990]	2.884849
ENSG00000136197	79020	C7orf25	protein_coding	HGNC Symbol	6	chromosome 7 open reading frame 25 [Source:HGNC Symbol;Acc:HGNC:21703]	2.886377
ENSG00000277083	NA	PRSS3P3	transcribed_unitary_pseudogene	HGNC Symbol	2	PRSS3 pseudogene 3 [Source:HGNC Symbol;Acc:HGNC:43790]	2.891067
ENSG00000151967	29970	SCHIP1	protein_coding	HGNC Symbol	8	schwannomin interacting protein 1 [Source:HGNC Symbol;Acc:HGNC:15678]	2.892534
ENSG00000121742	10804	GJB6	protein_coding	HGNC Symbol	15	gap junction protein beta 6 [Source:HGNC Symbol;Acc:HGNC:4288]	2.89451
ENSG00000228369	1.04E+08	TXNDC12-AS1	antisense	HGNC Symbol	1	TXNDC12 antisense RNA 1 [Source:HGNC Symbol;Acc:HGNC:30008]	2.90018
ENSG00000125740	2354	FOSB	protein_coding	HGNC Symbol	13	FosB proto-oncogene, AP-1 transcription factor subunit [Source:HGNC Symbol;Acc:HGNC:379	2.902491
ENSG00000181381	91351	DDX60L	protein_coding	HGNC Symbol	16	DExD/H-box 60 like [Source:HGNC Symbol;Acc:HGNC:26429]	2.905744
ENSG00000197019	29950	SERTAD1	protein_coding	HGNC Symbol	1	SERTA domain containing 1 [Source:HGNC Symbol;Acc:HGNC:17932]	2.908473
ENSG00000266498	NA	AC055811.3	lincRNA	Clone-based (Ensembl) gene	1	novel transcript	2.908874
ENSG00000074590	9891	NUAK1	protein_coding	HGNC Symbol	4	NUAK family kinase 1 [Source:HGNC Symbol;Acc:HGNC:14311]	2.91043
ENSG00000226625	NA	RBM17P1	processed_pseudogene	HGNC Symbol	1	RNA binding motif protein 17 pseudogene 1 [Source:HGNC Symbol;Acc:HGNC:50366]	2.912855

ENSG00000101665	4092	SMAD7	protein_coding	HGNC Symbol	8	SMAD family member 7 [Source:HGNC Symbol;Acc:HGNC:6773]	2.917968
ENSG00000275092	NA	AL031710.2	sense_intronic	Clone-based (Ensembl) gene	1	novel transcript, sense intronic to MAPK8IP3	2.91809
ENSG00000184557	9021	SOCS3	protein_coding	HGNC Symbol	2	suppressor of cytokine signaling 3 [Source:HGNC Symbol;Acc:HGNC:19391]	2.920639
ENSG00000227536	NA	SOCS5P4	processed_pseudogene	HGNC Symbol	1	suppressor of cytokine signaling 5 pseudogene 4 [Source:HGNC Symbol;Acc:HGNC:44600]	2.922719
ENSG00000262480	NA	SAMD11P1	processed_pseudogene	HGNC Symbol	1	sterile alpha motif domain containing 11 pseudogene 1 [Source:HGNC Symbol;Acc:HGNC:444	2.925128
ENSG00000176563	124817	CNTD1	protein_coding	HGNC Symbol	7	cyclin N-terminal domain containing 1 [Source:HGNC Symbol;Acc:HGNC:26847]	2.928196
ENSG00000100652	6554	SLC10A1	protein_coding	HGNC Symbol	1	solute carrier family 10 member 1 [Source:HGNC Symbol;Acc:HGNC:10905]	2.928683
ENSG00000200792	677846	SNORA80A	snoRNA	HGNC Symbol	1	small nucleolar RNA, H/ACA box 80A [Source:HGNC Symbol;Acc:HGNC:32666]	2.931488
ENSG00000254510	NA	AP001107.5	processed_transcript	Clone-based (Ensembl) gene	3		2.933431
ENSG00000160799	151903	CCDC12	protein_coding	HGNC Symbol	13	coiled-coil domain containing 12 [Source:HGNC Symbol;Acc:HGNC:28332]	2.93468
ENSG00000143751	163859	SDE2	protein_coding	HGNC Symbol	1	SDE2 telomere maintenance homolog [Source:HGNC Symbol;Acc:HGNC:26643]	2.938657
ENSG00000262228	NA	AC087392.3	lincRNA	Clone-based (Ensembl) gene	2	novel transcript	2.939907
ENSG00000238059	NA	HSPE1P21	processed_pseudogene	HGNC Symbol	1	heat shock protein family E (Hsp10) member 1 pseudogene 21 [Source:HGNC Symbol;Acc:HGI	2.943476
ENSG00000238133	339751	MAP3K20-AS1	antisense	HGNC Symbol	3	MAP3K20 antisense RNA 1 [Source:HGNC Symbol;Acc:HGNC:27935]	2.944704
ENSG00000236194	NA	AC099811.1	sense_intronic	Clone-based (Ensembl) gene	1	novel transcript, sense intronic to STAT5B	2.945191
ENSG00000118985	22936	ELL2	protein_coding	HGNC Symbol	8	elongation factor for RNA polymerase II 2 [Source:HGNC Symbol;Acc:HGNC:17064]	2.945327
ENSG00000124813	860	RUNX2	protein_coding	HGNC Symbol	13	runt related transcription factor 2 [Source:HGNC Symbol;Acc:HGNC:10472]	2.946321
ENSG00000260708	NA	AL118516.1	antisense	Clone-based (Ensembl) gene	1	novel transcript, antisense to TBC1D22A	2.947777
ENSG00000062582	64951	MRPS24	protein_coding	HGNC Symbol	5	mitochondrial ribosomal protein S24 [Source:HGNC Symbol;Acc:HGNC:14510]	2.949135
ENSG00000260898	1E+08	ADPGK-AS1	antisense	HGNC Symbol	2	ADPGK antisense RNA 1 [Source:HGNC Symbol;Acc:HGNC:44144]	2.949417
ENSG00000127824	7277	TUBA4A	protein_coding	HGNC Symbol	10	tubulin alpha 4a [Source:HGNC Symbol;Acc:HGNC:12407]	2.949812
ENSG00000275756	NA	AL354751.3	processed_pseudogene	Clone-based (Ensembl) gene	1	novel pseudogene	2.952201
ENSG00000124091	140687	GCNT7	protein_coding	HGNC Symbol	2	glucosaminyl (N-acetyl) transferase family member 7 [Source:HGNC Symbol;Acc:HGNC:16099	2.953256
ENSG00000104081	90427	BMF	protein_coding	HGNC Symbol	10	Bcl2 modifying factor [Source:HGNC Symbol;Acc:HGNC:24132]	2.954668
ENSG00000150630	7424	VEGFC	protein_coding	HGNC Symbol	2	vascular endothelial growth factor C [Source:HGNC Symbol;Acc:HGNC:12682]	2.955995
ENSG00000248476	NA	BACH1-IT1	sense_intronic	HGNC Symbol	1	BACH1 intronic transcript 1 [Source:HGNC Symbol;Acc:HGNC:40006]	2.956994
ENSG00000278493	NA	AC039056.2	sense_intronic	Clone-based (Ensembl) gene	1	novel transcript, sense intronic to EHD4	2.958475
ENSG00000261997	NA	AC007336.1	lincRNA	Clone-based (Ensembl) gene	1	novel transcript	2.95881
ENSG00000106588	5683	PSMA2	protein_coding	HGNC Symbol	5	proteasome subunit alpha 2 [Source:HGNC Symbol;Acc:HGNC:9531]	2.95946
ENSG00000230069	NA	LRRC37A15P	processed_pseudogene	HGNC Symbol	1	leucine rich repeat containing 37 member A15, pseudogene [Source:HGNC Symbol;Acc:HGNC	2.959745
ENSG00000264895	NA	AC006141.1	sense_intronic	Clone-based (Ensembl) gene	1	novel transcript, sense intronic to MBTD1	2.961136
ENSG00000231329	NA	AL031772.1	processed_transcript	Clone-based (Ensembl) gene	17	novel transcript	2.965655
ENSG00000178381	90637	ZFAND2A	protein_coding	HGNC Symbol	7	zinc finger AN1-type containing 2A [Source:HGNC Symbol;Acc:HGNC:28073]	2.966117
ENSG00000177606	3725	JUN	protein_coding	HGNC Symbol	1	Jun proto-oncogene, AP-1 transcription factor subunit [Source:HGNC Symbol;Acc:HGNC:6204	2.97114
ENSG00000106546	196	AHR	protein_coding	HGNC Symbol	7	aryl hydrocarbon receptor [Source:HGNC Symbol;Acc:HGNC:348]	2.972434
ENSG00000249947	NA	XBP1P1	processed_pseudogene	HGNC Symbol	1	X-box binding protein 1 pseudogene 1 [Source:HGNC Symbol;Acc:HGNC:12802]	2.978406
ENSG00000206754	594837	SNORD101	snoRNA	HGNC Symbol	1	small nucleolar RNA, C/D box 101 [Source:HGNC Symbol;Acc:HGNC:32764]	2.983825
ENSG00000229431	NA	AL139289.1	antisense	Clone-based (Ensembl) gene	1	novel transcript	2.985408
ENSG00000196459	6399	TRAPPC2	protein_coding	HGNC Symbol	8	trafficking protein particle complex 2 [Source:HGNC Symbol;Acc:HGNC:23068]	2.985557
ENSG00000188766	399473	SPRED3	protein_coding	HGNC Symbol	7	sprouty related EVH1 domain containing 3 [Source:HGNC Symbol;Acc:HGNC:31041]	2.985965
ENSG00000172578	89857	KLHL6	protein_coding	HGNC Symbol	4	kelch like family member 6 [Source:HGNC Symbol;Acc:HGNC:18653]	2.989754
ENSG00000162944	130132	RFTN2	protein_coding	HGNC Symbol	4	raftlin family member 2 [Source:HGNC Symbol;Acc:HGNC:26402]	2.995726
ENSG00000266651	NA	AC093484.4	lincRNA	Clone-based (Ensembl) gene	1	novel transcript	2.996188
ENSG00000169047	3667	IRS1	protein_coding	HGNC Symbol	2	insulin receptor substrate 1 [Source:HGNC Symbol;Acc:HGNC:6125]	3.001884
ENSG00000120149	4488	MSX2	protein_coding	HGNC Symbol	2	msh homeobox 2 [Source:HGNC Symbol;Acc:HGNC:7392]	3.00386
ENSG00000232630	NA	PRPS1P2	processed_pseudogene	HGNC Symbol	1	phosphoribosyl pyrophosphate synthetase 1 pseudogene 2 [Source:HGNC Symbol;Acc:HGNC:	3.00512
ENSG00000143507	11221	DUSP10	protein_coding	HGNC Symbol	4	dual specificity phosphatase 10 [Source:HGNC Symbol;Acc:HGNC:3065]	3.006548
ENSG00000225442	NA	MPRIP-AS1	antisense	HGNC Symbol	1	MPRIP antisense RNA 1 [Source:HGNC Symbol;Acc:HGNC:41263]	3.00663
ENSG00000226055	NA	PAICSP1	processed_pseudogene	HGNC Symbol	1	phosphoribosylaminoimidazole carboxylase, phosphoribosylaminoimidazole succinocarboxam	3.012724
ENSG00000180667	55432	YOD1	protein_coding	HGNC Symbol	2	YOD1 deubiquitinase [Source:HGNC Symbol;Acc:HGNC:25035]	3.013605
ENSG00000236296	441046	GUSBP5	transcribed_processed_pseudogene	HGNC Symbol	2	glucuronidase, beta pseudogene 5 [Source:HGNC Symbol;Acc:HGNC:42319]	3.013678
ENSG00000271075	NA	AL139317.4	processed_pseudogene	Clone-based (Ensembl) gene	1	golgi membrane protein 1 (GOLM1) pseudogene	3.014495
ENSG00000270480	NA	AC073413.1	processed_pseudogene	Clone-based (Ensembl) gene	1	PQ loop repeat containing 1 (PQLC1) pseudogene	3.014511
ENSG00000160570	162989	DEDD2	protein_coding	HGNC Symbol	13	death effector domain containing 2 [Source:HGNC Symbol;Acc:HGNC:24450]	3.014601
ENSG00000146707	22932	POMZP3	protein_coding	HGNC Symbol	5	POM121 and ZP3 fusion [Source:HGNC Symbol;Acc:HGNC:9203]	3.015111
ENSG00000228451	157489	SDAD1P1	transcribed_processed_pseudogene	HGNC Symbol	3	SDA1 domain containing 1 pseudogene 1 [Source:HGNC Symbol;Acc:HGNC:31403]	3.018084
ENSG00000198633	147658	ZNF534	protein_coding	HGNC Symbol	5	zinc finger protein 534 [Source:HGNC Symbol;Acc:HGNC:26337]	3.01893
ENSG00000137504	58487	CREBZF	protein_coding	HGNC Symbol	9	CREB/ATF bZIP transcription factor [Source:HGNC Symbol;Acc:HGNC:24905]	3.020296
ENSG00000143367	7286	TUFT1	protein_coding	HGNC Symbol	7	tuftelin 1 [Source:HGNC Symbol;Acc:HGNC:12422]	3.021436
ENSG00000148516	6935	ZEB1	protein_coding	HGNC Symbol	25	zinc finger E-box binding homeobox 1 [Source:HGNC Symbol;Acc:HGNC:11642]	3.022258
ENSG00000260005	NA	AC027601.1	antisense	Clone-based (Ensembl) gene	2		3.022842
ENSG00000188305	374872	PEAK3	protein_coding	HGNC Symbol	1	PEAK family member 3 [Source:HGNC Symbol;Acc:HGNC:24793]	3.023148
ENSG00000213073	729603	AL353625.1	transcribed_processed_pseudogene	Clone-based (Ensembl) gene	2		3.023479
ENSG00000184545	1850	DUSP8	protein_coding	HGNC Symbol	3	dual specificity phosphatase 8 [Source:HGNC Symbol;Acc:HGNC:3074]	3.023958
ENSG00000174738	9975	NR1D2	protein_coding	HGNC Symbol	5	nuclear receptor subfamily 1 group D member 2 [Source:HGNC Symbol;Acc:HGNC:7963]	3.0258

ENSG00000244556	NA	ODCP	processed_pseudogene	HGNC Symbol	1	ornithine decarboxylase pseudogene [Source:HGNC Symbol;Acc:HGNC:8110]	3.029653
ENSG00000261889	NA	AC108134.2	lincRNA	Clone-based (Ensembl) gene	1	novel transcript	3.030442
ENSG00000258413	NA	AL158801.2	lincRNA	Clone-based (Ensembl) gene	2	novel transcript	3.03092
ENSG00000178201	57191	VN1R1	protein_coding	HGNC Symbol	1	vomeronasal 1 receptor 1 [Source:HGNC Symbol;Acc:HGNC:13548]	3.033939
ENSG00000210140	NA	MT-TC	Mt_tRNA	HGNC Symbol	1	mitochondrially encoded tRNA cysteine [Source:HGNC Symbol;Acc:HGNC:7477]	3.035225
ENSG00000171757	151827	LRRC34	protein_coding	HGNC Symbol	12	leucine rich repeat containing 34 [Source:HGNC Symbol;Acc:HGNC:28408]	3.04009
ENSG00000197933	55552	ZNF823	protein_coding	HGNC Symbol	4	zinc finger protein 823 [Source:HGNC Symbol;Acc:HGNC:30936]	3.041223
ENSG00000261542	NA	AC011978.2	lincRNA	Clone-based (Ensembl) gene	1	novel transcript	3.043215
ENSG00000259118	NA	AL139022.1	antisense	Clone-based (Ensembl) gene	4		3.048075
ENSG00000278709	1.05E+08	NKILA	antisense	HGNC Symbol	2	NF-kappaB interacting lncRNA [Source:HGNC Symbol;Acc:HGNC:51599]	3.050031
ENSG00000166401	5271	SERPINB8	protein_coding	HGNC Symbol	9	serpin family B member 8 [Source:HGNC Symbol;Acc:HGNC:8952]	3.050133
ENSG00000215179	NA	MAPK6P4	processed_pseudogene	HGNC Symbol	1	mitogen-activated protein kinase 6 pseudogene 4 [Source:HGNC Symbol;Acc:HGNC:18456]	3.053047
ENSG00000206384	131873	COL6A6	protein_coding	HGNC Symbol	3	collagen type VI alpha 6 chain [Source:HGNC Symbol;Acc:HGNC:27023]	3.056211
ENSG00000081059	6932	TCF7	protein_coding	HGNC Symbol	24	transcription factor 7 [Source:HGNC Symbol;Acc:HGNC:11639]	3.058699
ENSG00000232611	NA	AL683813.1	lincRNA	Clone-based (Ensembl) gene	1	novel transcript	3.059537
ENSG00000181894	79673	ZNF329	protein_coding	HGNC Symbol	5	zinc finger protein 329 [Source:HGNC Symbol;Acc:HGNC:14209]	3.065275
ENSG00000110876	6404	SELPLG	protein_coding	HGNC Symbol	3	selectin P ligand [Source:HGNC Symbol;Acc:HGNC:10722]	3.065328
ENSG00000250303	283140	AP002884.1	lincRNA	Clone-based (Ensembl) gene	5		3.065639
ENSG00000254873	NA	AP001267.1	antisense	Clone-based (Ensembl) gene	2	novel transcript, antisense to ATP5L and UBE4A	3.06821
ENSG00000259657	NA	PIGHP1	processed_pseudogene	HGNC Symbol	1	phosphatidylinositol glycan anchor biosynthesis class H pseudogene 1 [Source:HGNC Symbol;	3.06939
ENSG00000124762	1026	CDKN1A	protein_coding	HGNC Symbol	8	cyclin dependent kinase inhibitor 1A [Source:HGNC Symbol;Acc:HGNC:1784]	3.077181
ENSG00000137814	55142	HAUS2	protein_coding	HGNC Symbol	11	HAUS augmin like complex subunit 2 [Source:HGNC Symbol;Acc:HGNC:25530]	3.084239
ENSG00000242559	NA	RN7SL144P	misc_RNA	HGNC Symbol	1	RNA, 7SL, cytoplasmic 144, pseudogene [Source:HGNC Symbol;Acc:HGNC:46160]	3.101798
ENSG00000232536	NA	AL365436.2	sense_intronic	Clone-based (Ensembl) gene	1	novel transcript	3.102217
ENSG00000132823	51526	OSER1	protein_coding	HGNC Symbol	2	oxidative stress responsive serine rich 1 [Source:HGNC Symbol;Acc:HGNC:16105]	3.104841
ENSG00000163083	3625	INHBB	protein_coding	HGNC Symbol	1	inhibin subunit beta B [Source:HGNC Symbol;Acc:HGNC:6067]	3.104964
ENSG00000213592	NA	PPIAP42	processed_pseudogene	HGNC Symbol	1	peptidylprolyl isomerase A pseudogene 42 [Source:HGNC Symbol;Acc:HGNC:53666]	3.10796
ENSG00000224097	NA	AC021148.1	transcribed_processed_pseudogene	Clone-based (Ensembl) gene	3		3.110675
ENSG00000177453	167359	NIM1K	protein_coding	HGNC Symbol	5	NIM1 serine/threonine protein kinase [Source:HGNC Symbol;Acc:HGNC:28646]	3.111457
ENSG00000249741	NA	AC093890.1	processed_pseudogene	Clone-based (Ensembl) gene	1	salvador homolog 1 (Drosophila) (SAV1) pseudogene	3.116073
ENSG00000165312	220213	OTUD1	protein_coding	HGNC Symbol	1	OTU deubiquitinase 1 [Source:HGNC Symbol;Acc:HGNC:27346]	3.122821
ENSG00000113916	604	BCL6	protein_coding	HGNC Symbol	11	B cell CLL/lymphoma 6 [Source:HGNC Symbol;Acc:HGNC:1001]	3.128249
ENSG00000235748	NA	SEPT14P12	processed_pseudogene	HGNC Symbol	1	septin 14 pseudogene 12 [Source:HGNC Symbol;Acc:HGNC:51699]	3.133978
ENSG00000228212	NA	OFD1P17	processed_pseudogene	HGNC Symbol	1	OFD1 pseudogene 17 [Source:HGNC Symbol;Acc:HGNC:1332]	3.13453
ENSG00000061337	11178	LZTS1	protein_coding	HGNC Symbol	4	leucine zipper tumor suppressor 1 [Source:HGNC Symbol;Acc:HGNC:13861]	3.136823
ENSG00000131471	8639	AOC3	protein_coding	HGNC Symbol	7	amine oxidase, copper containing 3 [Source:HGNC Symbol;Acc:HGNC:550]	3.140425
ENSG00000265261	NA	AC027575.1	processed_pseudogene	Clone-based (Ensembl) gene	1	exportin, tRNA (nuclear export receptor for tRNAs) (XPOT) pseudogene	3.141094
ENSG00000151553	57700	FAM160B1	protein_coding	HGNC Symbol	4	family with sequence similarity 160 member B1 [Source:HGNC Symbol;Acc:HGNC:29320]	3.142694
ENSG00000135100	6927	HNF1A	protein_coding	HGNC Symbol	16	HNF1 homeobox A [Source:HGNC Symbol;Acc:HGNC:11621]	3.145866
ENSG00000236255	NA	AC009404.1	lincRNA	Clone-based (Ensembl) gene	3	novel transcript	3.152975
ENSG00000188710	347148	QRFP	protein_coding	HGNC Symbol	2	pyroglutamylated RFamide peptide [Source:HGNC Symbol;Acc:HGNC:29982]	3.155293
ENSG00000235927	374987	NEXN-AS1	antisense	HGNC Symbol	1	NEXN antisense RNA 1 [Source:HGNC Symbol;Acc:HGNC:31983]	3.163187
ENSG00000118257	8828	NRP2	protein_coding	HGNC Symbol	17	neuropilin 2 [Source:HGNC Symbol;Acc:HGNC:8005]	3.172028
ENSG00000182902	83733	SLC25A18	protein_coding	HGNC Symbol	6	solute carrier family 25 member 18 [Source:HGNC Symbol;Acc:HGNC:10988]	3.173315
ENSG00000234040	NA	RPL10P12	processed_pseudogene	HGNC Symbol	1	ribosomal protein L10 pseudogene 12 [Source:HGNC Symbol;Acc:HGNC:52345]	3.173502
ENSG00000196781	7088	TLE1	protein_coding	HGNC Symbol	6	transducin like enhancer of split 1 [Source:HGNC Symbol;Acc:HGNC:11837]	3.176713
ENSG00000271843	NA	AC012557.1	lincRNA	Clone-based (Ensembl) gene	1	novel transcript	3.176837
ENSG00000273212	NA	AC000068.2	antisense	Clone-based (Ensembl) gene	1	novel transcript, antisense to UFD1L	3.177154
ENSG00000235609	NA	AF127577.4	lincRNA	Clone-based (Ensembl) gene	1	novel transcript	3.179742
ENSG00000257438	NA	AC011595.1	antisense	Clone-based (Ensembl) gene	1	novel transcript, antisense to NUAK1	3.179836
ENSG00000235066	NA	AC009303.2	antisense	Clone-based (Ensembl) gene	10	novel transcript, antisense to CCDC93	3.180947
ENSG00000235635	NA	AC016644.1	antisense	Clone-based (Ensembl) gene	1	novel transcript	3.19185
ENSG00000010818	3097	HIVEP2	protein_coding	HGNC Symbol	5	human immunodeficiency virus type I enhancer binding protein 2 [Source:HGNC Symbol;Acc:	3.192027
ENSG00000130803	57693	ZNF317	protein_coding	HGNC Symbol	6	zinc finger protein 317 [Source:HGNC Symbol;Acc:HGNC:13507]	3.195795
ENSG00000169184	4330	MN1	protein_coding	HGNC Symbol	3	MN1 proto-oncogene, transcriptional regulator [Source:HGNC Symbol;Acc:HGNC:7180]	3.199101
ENSG00000256628	1E+08	ZBTB11-AS1	antisense	HGNC Symbol	1	ZBTB11 antisense RNA 1 [Source:HGNC Symbol;Acc:HGNC:48573]	3.199376
ENSG00000252759	NA	RF00019	misc_RNA	RFAM	1		3.203046
ENSG00000174529	388730	TMEM81	protein_coding	HGNC Symbol	1	transmembrane protein 81 [Source:HGNC Symbol;Acc:HGNC:32349]	3.205708
ENSG00000186790	2301	FOXE3	protein_coding	HGNC Symbol	1	forkhead box E3 [Source:HGNC Symbol;Acc:HGNC:3808]	3.205904
ENSG00000239300	NA	AC080162.1	antisense	Clone-based (Ensembl) gene	2	novel transcript	3.221298
ENSG00000197142	51703	ACSL5	protein_coding	HGNC Symbol	9	acyl-CoA synthetase long chain family member 5 [Source:HGNC Symbol;Acc:HGNC:16526]	3.221402
ENSG00000213608	NA	SLC25A14P1	processed_pseudogene	HGNC Symbol	1	solute carrier family 25 member 14 pseudogene 1 [Source:HGNC Symbol;Acc:HGNC:43856]	3.226999
ENSG00000228363	NA	AC015971.1	antisense	Clone-based (Ensembl) gene	4	novel transcript	3.229255
ENSG00000091656	79776	ZFHX4	protein_coding	HGNC Symbol	12	zinc finger homeobox 4 [Source:HGNC Symbol;Acc:HGNC:30939]	3.231765
ENSG00000165655	84858	ZNF503	protein_coding	HGNC Symbol	1	zinc finger protein 503 [Source:HGNC Symbol;Acc:HGNC:23589]	3.233157

ENSG00000272696	NA	AL359091.4	antisense	Clone-based (Ensembl) gene	1	novel transcript, antisense to TRUB2	3.235941
ENSG00000140941	81631	MAP1LC3B	protein_coding	HGNC Symbol	9	microtubule associated protein 1 light chain 3 beta [Source:HGNC Symbol;Acc:HGNC:13352]	3.24062
ENSG00000269855	646862	RNF225	protein_coding	HGNC Symbol	1	ring finger protein 225 [Source:HGNC Symbol;Acc:HGNC:51249]	3.241869
ENSG00000260325	94086	HSPB9	protein_coding	HGNC Symbol	1	heat shock protein family B (small) member 9 [Source:HGNC Symbol;Acc:HGNC:30589]	3.242379
ENSG00000206897	1.1E+08	SNORA9B	snoRNA	HGNC Symbol	1	small nucleolar RNA, H/ACA box 9B [Source:HGNC Symbol;Acc:HGNC:52223]	3.243876
ENSG00000276867	NA	AC074050.4	lincRNA	Clone-based (Ensembl) gene	1	novel transcript	3.244662
ENSG00000081051	174	AFP	protein_coding	HGNC Symbol	7	alpha fetoprotein [Source:HGNC Symbol;Acc:HGNC:317]	3.248191
ENSG00000196449	79693	YRDC	protein_coding	HGNC Symbol	1	yrdC N6-threonylcarbamoyltransferase domain containing [Source:HGNC Symbol;Acc:HGNC:2	3.250649
ENSG00000110375	7379	UPK2	protein_coding	HGNC Symbol	2	uroplakin 2 [Source:HGNC Symbol;Acc:HGNC:12579]	3.251513
ENSG00000223356	NA	AL590666.1	antisense	Clone-based (Ensembl) gene	1	novel transcript	3.252524
ENSG00000203706	NA	SERTAD4-AS1	antisense	HGNC Symbol	3	SERTAD4 antisense RNA 1 [Source:HGNC Symbol;Acc:HGNC:32019]	3.254497
ENSG00000250027	NA	AC022272.1	antisense	Clone-based (Ensembl) gene	2	novel transcript	3.259231
ENSG00000258359	NA	PCNPP1	processed_pseudogene	HGNC Symbol	1	PEST containing nuclear protein pseudogene 1 [Source:HGNC Symbol;Acc:HGNC:32440]	3.260076
ENSG00000117036	2117	ETV3	protein_coding	HGNC Symbol	3	ETS variant 3 [Source:HGNC Symbol;Acc:HGNC:3492]	3.268585
ENSG00000163071	132671	SPATA18	protein_coding	HGNC Symbol	10	spermatogenesis associated 18 [Source:HGNC Symbol;Acc:HGNC:29579]	3.270727
ENSG00000245970	NA	AP003352.1	antisense	Clone-based (Ensembl) gene	1	novel transcript	3.275315
ENSG00000175213	79797	ZNF408	protein_coding	HGNC Symbol	5	zinc finger protein 408 [Source:HGNC Symbol;Acc:HGNC:20041]	3.27549
ENSG00000181026	64782	AEN	protein_coding	HGNC Symbol	6	apoptosis enhancing nuclease [Source:HGNC Symbol;Acc:HGNC:25722]	3.280313
ENSG00000152137	26353	HSPB8	protein_coding	HGNC Symbol	3	heat shock protein family B (small) member 8 [Source:HGNC Symbol;Acc:HGNC:30171]	3.284407
ENSG00000241529	NA	RN7SL767P	misc_RNA	HGNC Symbol	1	RNA, 7SL, cytoplasmic 767, pseudogene [Source:HGNC Symbol;Acc:HGNC:46783]	3.290775
ENSG00000168878	6439	SFTPB	protein_coding	HGNC Symbol	7	surfactant protein B [Source:HGNC Symbol;Acc:HGNC:10801]	3.29564
ENSG00000224959	NA	AC017002.1	lincRNA	Clone-based (Ensembl) gene	1	novel transcript	3.300623
ENSG00000205763	441212	RP9P	transcribed_unprocessed_pseudogene	HGNC Symbol	2	RP9 pseudogene [Source:HGNC Symbol;Acc:HGNC:33969]	3.307523
ENSG00000238317	692058	SNORD11	snoRNA	HGNC Symbol	1	small nucleolar RNA, C/D box 11 [Source:HGNC Symbol;Acc:HGNC:32707]	3.308489
ENSG00000270890	NA	AL049844.2	processed_pseudogene	Clone-based (Ensembl) gene	1	synaptophysin-like 1 (SYPL1) pseudogene	3.309761
ENSG00000172940	9390	SLC22A13	protein_coding	HGNC Symbol	3	solute carrier family 22 member 13 [Source:HGNC Symbol;Acc:HGNC:8494]	3.315668
ENSG00000171223	3726	JUNB	protein_coding	HGNC Symbol	1	JunB proto-oncogene, AP-1 transcription factor subunit [Source:HGNC Symbol;Acc:HGNC:620	3.317264
ENSG00000168994	221749	PXDC1	protein_coding	HGNC Symbol	4	PX domain containing 1 [Source:HGNC Symbol;Acc:HGNC:21361]	3.318954
ENSG00000181350	388341	LRRC75A	protein_coding	HGNC Symbol	3	leucine rich repeat containing 75A [Source:HGNC Symbol;Acc:HGNC:32403]	3.32423
ENSG00000176971	387758	FIBIN	protein_coding	HGNC Symbol	1	fin bud initiation factor homolog [Source:HGNC Symbol;Acc:HGNC:33747]	3.326331
ENSG00000174564	53833	IL20RB	protein_coding	HGNC Symbol	5	interleukin 20 receptor subunit beta [Source:HGNC Symbol;Acc:HGNC:6004]	3.329618
ENSG00000263220	NA	AC015727.1	antisense	Clone-based (Ensembl) gene	1	novel transcript, antisense to RABEP1	3.330805
ENSG00000228028	NA	AC069257.1	antisense	Clone-based (Ensembl) gene	2	novel transcript	3.331034
ENSG00000271862	NA	AC104118.1	lincRNA	Clone-based (Ensembl) gene	1	novel transcript	3.333866
ENSG00000163874	80149	ZC3H12A	protein_coding	HGNC Symbol	4	zinc finger CCCH-type containing 12A [Source:HGNC Symbol;Acc:HGNC:26259]	3.336542
ENSG00000229334	NA	AC046143.1	antisense	Clone-based (Ensembl) gene	1	novel transcript	3.34073
ENSG00000203734	345930	ECT2L	protein_coding	HGNC Symbol	5	epithelial cell transforming 2 like [Source:HGNC Symbol;Acc:HGNC:21118]	3.341196
ENSG00000129749	57053	CHRNA10	protein_coding	HGNC Symbol	4	cholinergic receptor nicotinic alpha 10 subunit [Source:HGNC Symbol;Acc:HGNC:13800]	3.345848
ENSG00000148584	29974	A1CF	protein_coding	HGNC Symbol	10	APOBEC1 complementation factor [Source:HGNC Symbol;Acc:HGNC:24086]	3.350675
ENSG00000256325	NA	AC025423.1	antisense	Clone-based (Ensembl) gene	1	novel transcript	3.353195
ENSG00000227304	NA	AC067942.1	transcribed_processed_pseudogene	Clone-based (Ensembl) gene	2	ribosomal protein S6 (RPS6) pseudogene	3.359168
ENSG00000233012	NA	HDAC1P2	transcribed_processed_pseudogene	HGNC Symbol	2	histone deacetylase 1 pseudogene 2 [Source:HGNC Symbol;Acc:HGNC:45191]	3.359701
ENSG00000260239	NA	LINC02533	lincRNA	HGNC Symbol	1	long intergenic non-protein coding RNA 2533 [Source:HGNC Symbol;Acc:HGNC:53566]	3.363191
ENSG00000247853	NA	AC006064.2	antisense	Clone-based (Ensembl) gene	1	novel transcript, antisense to CHD4	3.363987
ENSG00000103490	29108	PYCARD	protein_coding	HGNC Symbol	4	PYD and CARD domain containing [Source:HGNC Symbol;Acc:HGNC:16608]	3.364361
ENSG00000214354	NA	AC133644.1	processed_pseudogene	Clone-based (Ensembl) gene	1	platelet-activating factor acetylhydrolase, isoform Ib, alpha subunit 45kDa (PAFAH1B1) pseud	3.364814
ENSG00000170122	2298	FOXD4	protein_coding	HGNC Symbol	1	forkhead box D4 [Source:HGNC Symbol;Acc:HGNC:3805]	3.365439
ENSG00000272644	NA	AC097468.3	lincRNA	Clone-based (Ensembl) gene	1	novel transcript	3.369488
ENSG00000180801	79642	ARSJ	protein_coding	HGNC Symbol	4	arylsulfatase family member J [Source:HGNC Symbol;Acc:HGNC:26286]	3.369524
ENSG00000137502	27314	RAB30	protein_coding	HGNC Symbol	16	RAB30, member RAS oncogene family [Source:HGNC Symbol;Acc:HGNC:9770]	3.370877
ENSG00000116285	54206	ERRFI1	protein_coding	HGNC Symbol	5	ERBB receptor feedback inhibitor 1 [Source:HGNC Symbol;Acc:HGNC:18185]	3.375659
ENSG00000189057	374393	FAM111B	protein_coding	HGNC Symbol	6	family with sequence similarity 111 member B [Source:HGNC Symbol;Acc:HGNC:24200]	3.379287
ENSG00000234789	NA	AL590369.1	antisense	Clone-based (Ensembl) gene	1	novel transcript	3.387113
ENSG00000108255	1411	CRYBA1	protein_coding	HGNC Symbol	2	crystallin beta A1 [Source:HGNC Symbol;Acc:HGNC:2394]	3.388354
ENSG00000231216	1E+08	GS1-600G8.3	antisense	NCBI gene	1	unknown transcript [Source:NCBI gene;Acc:100093698]	3.395847
ENSG00000179909	7710	ZNF154	protein_coding	HGNC Symbol	2	zinc finger protein 154 [Source:HGNC Symbol;Acc:HGNC:12939]	3.398214
ENSG00000054598	2296	FOXC1	protein_coding	HGNC Symbol	2	forkhead box C1 [Source:HGNC Symbol;Acc:HGNC:3800]	3.403549
ENSG00000204103	9935	MAFB	protein_coding	HGNC Symbol	1	MAF bZIP transcription factor B [Source:HGNC Symbol;Acc:HGNC:6408]	3.407707
ENSG00000270184	NA	AC018695.4	antisense	Clone-based (Ensembl) gene	1	novel transcript, antisense to COX4NB	3.408953
ENSG00000260490	NA	MYL12BP1	processed_pseudogene	HGNC Symbol	1	myosin light chain 12B pseudogene 1 [Source:HGNC Symbol;Acc:HGNC:51649]	3.409339
ENSG00000095752	3589	IL11	protein_coding	HGNC Symbol	4	interleukin 11 [Source:HGNC Symbol;Acc:HGNC:5966]	3.410461
ENSG00000189292	285016	ALKAL2	protein_coding	HGNC Symbol	8	ALK and LTK ligand 2 [Source:HGNC Symbol;Acc:HGNC:27683]	3.412059
ENSG00000111729	50856	CLEC4A	protein_coding	HGNC Symbol	5	C-type lectin domain family 4 member A [Source:HGNC Symbol;Acc:HGNC:13257]	3.418111
ENSG00000254463	NA	PPIAP41	processed_pseudogene	HGNC Symbol	1	peptidylprolyl isomerase A pseudogene 41 [Source:HGNC Symbol;Acc:HGNC:53665]	3.434313
ENSG00000188295	79862	ZNF669	protein_coding	HGNC Symbol	5	zinc finger protein 669 [Source:HGNC Symbol;Acc:HGNC:25736]	3.443331

ENSG00000240350	NA	AC017002.3	lincRNA	Clone-based (Ensembl) gene	2		3.443415
ENSG00000259364	NA	AC013356.3	antisense	Clone-based (Ensembl) gene	1	novel transcript, antisense to BAHD1	3.44632
ENSG00000136802	56262	LRRC8A	protein_coding	HGNC Symbol	5	leucine rich repeat containing 8 VRAC subunit A [Source:HGNC Symbol;Acc:HGNC:19027]	3.454547
ENSG00000239653	1.01E+08	PSMD6-AS2	antisense	HGNC Symbol	1	PSMD6 antisense RNA 2 [Source:HGNC Symbol;Acc:HGNC:44125]	3.456044
ENSG00000153094	10018	BCL2L11	protein_coding	HGNC Symbol	25	BCL2 like 11 [Source:HGNC Symbol;Acc:HGNC:994]	3.4598
ENSG00000159885	7673	ZNF222	protein_coding	HGNC Symbol	4	zinc finger protein 222 [Source:HGNC Symbol;Acc:HGNC:13015]	3.463876
ENSG00000270641	9383	TSIX	lincRNA	HGNC Symbol	1	TSIX transcript, XIST antisense RNA [Source:HGNC Symbol;Acc:HGNC:12377]	3.471092
ENSG00000164776	5260	PHKG1	protein_coding	HGNC Symbol	10	phosphorylase kinase catalytic subunit gamma 1 [Source:HGNC Symbol;Acc:HGNC:8930]	3.472617
ENSG00000173334	10221	TRIB1	protein_coding	HGNC Symbol	4	tribbles pseudokinase 1 [Source:HGNC Symbol;Acc:HGNC:16891]	3.473179
ENSG00000228817	NA	BACH1-IT2	lincRNA	HGNC Symbol	3	BACH1 intronic transcript 2 [Source:HGNC Symbol;Acc:HGNC:40007]	3.480274
ENSG00000224818	NA	AC096677.2	3prime_overlapping_ncRNA	Clone-based (Ensembl) gene	1	novel transcript	3.481879
ENSG00000184492	200350	FOXD4L1	protein_coding	HGNC Symbol	1	forkhead box D4 like 1 [Source:HGNC Symbol;Acc:HGNC:18521]	3.485467
ENSG00000242634	NA	RPS24P16	processed_pseudogene	HGNC Symbol	1	ribosomal protein S24 pseudogene 16 [Source:HGNC Symbol;Acc:HGNC:35542]	3.490384
ENSG00000112763	11120	BTN2A1	protein_coding	HGNC Symbol	7	butyrophilin subfamily 2 member A1 [Source:HGNC Symbol;Acc:HGNC:1136]	3.496346
ENSG00000198768	164284	APCDD1L	protein_coding	HGNC Symbol	3	APC down-regulated 1 like [Source:HGNC Symbol;Acc:HGNC:26892]	3.498792
ENSG00000214376	387804	VSTM5	protein_coding	HGNC Symbol	3	V-set and transmembrane domain containing 5 [Source:HGNC Symbol;Acc:HGNC:34443]	3.498861
ENSG00000068079	3430	IFI35	protein_coding	HGNC Symbol	8	interferon induced protein 35 [Source:HGNC Symbol;Acc:HGNC:5399]	3.502028
ENSG00000169067	345651	ACTBL2	protein_coding	HGNC Symbol	1	actin, beta like 2 [Source:HGNC Symbol;Acc:HGNC:17780]	3.502089
ENSG00000248863	NA	AC097376.1	transcribed_processed_pseudogene	Clone-based (Ensembl) gene	2	glucose-fructose oxidoreductase domain containing 2 (GFOD2) pseudogene	3.510163
ENSG00000277895	NA	AC135279.3	antisense	Clone-based (Ensembl) gene	1	novel transcript, antisense to TBK1	3.511507
ENSG00000232118	NA	BACH1-AS1	lincRNA	HGNC Symbol	2	BACH1 antisense RNA 1 [Source:HGNC Symbol;Acc:HGNC:40008]	3.527313
ENSG00000168564	55602	CDKN2AIP	protein_coding	HGNC Symbol	5	CDKN2A interacting protein [Source:HGNC Symbol;Acc:HGNC:24325]	3.535337
ENSG00000267592	NA	AC004134.1	processed_pseudogene	Clone-based (Ensembl) gene	1	transmembrane protein 160 (TMEM160) pseudogene	3.53712
ENSG00000243260	NA	RN7SL558P	misc_RNA	HGNC Symbol	1	RNA, 7SL, cytoplasmic 558, pseudogene [Source:HGNC Symbol;Acc:HGNC:46574]	3.539664
ENSG00000261505	NA	AL031714.1	antisense	Clone-based (Ensembl) gene	2	novel transcript, antisense to UBE2I	3.542059
ENSG00000166856	11318	GPR182	protein_coding	HGNC Symbol	3	G protein-coupled receptor 182 [Source:HGNC Symbol;Acc:HGNC:13708]	3.546507
ENSG00000147174	93953	GCNA	protein_coding	HGNC Symbol	3	germ cell nuclear acidic peptidase [Source:HGNC Symbol;Acc:HGNC:15805]	3.548624
ENSG00000171385	3752	KCND3	protein_coding	HGNC Symbol	3	potassium voltage-gated channel subfamily D member 3 [Source:HGNC Symbol;Acc:HGNC:62	3.553878
ENSG00000138166	1847	DUSP5	protein_coding	HGNC Symbol	2	dual specificity phosphatase 5 [Source:HGNC Symbol;Acc:HGNC:3071]	3.557987
ENSG00000128422	3872	KRT17	protein_coding	HGNC Symbol	10	keratin 17 [Source:HGNC Symbol;Acc:HGNC:6427]	3.558564
ENSG00000249884	1.01E+08	RNF103-CHMP3	protein_coding	HGNC Symbol	2	RNF103-CHMP3 readthrough [Source:HGNC Symbol;Acc:HGNC:38847]	3.559532
ENSG00000206739	NA	RF00019	misc_RNA	RFAM	1		3.567462
ENSG00000091879	285	ANGPT2	protein_coding	HGNC Symbol	4	angiopoietin 2 [Source:HGNC Symbol;Acc:HGNC:485]	3.572934
ENSG00000165188	138065	RNF183	protein_coding	HGNC Symbol	7	ring finger protein 183 [Source:HGNC Symbol;Acc:HGNC:28721]	3.573345
ENSG00000134954	2113	ETS1	protein_coding	HGNC Symbol	9	ETS proto-oncogene 1, transcription factor [Source:HGNC Symbol;Acc:HGNC:3488]	3.580889
ENSG00000229349	NA	ACTG1P9	processed_pseudogene	HGNC Symbol	1	actin gamma 1 pseudogene 9 [Source:HGNC Symbol;Acc:HGNC:154]	3.582583
ENSG00000277879	NA	AL391988.1	antisense	Clone-based (Ensembl) gene	1	novel transcript, antisense to SLC18A2	3.585492
ENSG00000175505	23529	CLCF1	protein_coding	HGNC Symbol	2	cardiotrophin like cytokine factor 1 [Source:HGNC Symbol;Acc:HGNC:17412]	3.589902
ENSG00000255717	23642	SNHG1	processed_transcript	HGNC Symbol	25	small nucleolar RNA host gene 1 [Source:HGNC Symbol;Acc:HGNC:32688]	3.592417
ENSG00000217527	NA	RPS16P5	transcribed_processed_pseudogene	HGNC Symbol	1	ribosomal protein S16 pseudogene 5 [Source:HGNC Symbol;Acc:HGNC:36183]	3.594041
ENSG00000171657	27197	GPR82	protein_coding	HGNC Symbol	2	G protein-coupled receptor 82 [Source:HGNC Symbol;Acc:HGNC:4533]	3.604824
ENSG00000236890	NA	AC016027.5	processed_pseudogene	Clone-based (Ensembl) gene	1	ribosomal protein L18a (RPL18A) pseudogene	3.616852
ENSG00000206633	1E+08	SNORA80B	snoRNA	HGNC Symbol	1	small nucleolar RNA, H/ACA box 80B [Source:HGNC Symbol;Acc:HGNC:34355]	3.62007
ENSG00000134962	152831	KLB	protein_coding	HGNC Symbol	1	klotho beta [Source:HGNC Symbol;Acc:HGNC:15527]	3.625859
ENSG00000249012	NA	AC104819.1	unprocessed_pseudogene	Clone-based (Ensembl) gene	1	trypsin domain contaiing protein pseudogene	3.643934
ENSG00000104327	793	CALB1	protein_coding	HGNC Symbol	11	calbindin 1 [Source:HGNC Symbol;Acc:HGNC:1434]	3.655187
ENSG00000189423	NA	USP32P3	transcribed_unprocessed_pseudogene	HGNC Symbol	4	ubiquitin specific peptidase 32 pseudogene 3 [Source:HGNC Symbol;Acc:HGNC:43576]	3.677521
ENSG00000228446	NA	AC073052.1	processed_pseudogene	Clone-based (Ensembl) gene	1	ankyrin repeat domain 49 (ANKRD49) pseudogene	3.678077
ENSG00000135747	NA	ZNF670-ZNF695	protein_coding	HGNC Symbol	2	ZNF670-ZNF695 readthrough (NMD candidate) [Source:HGNC Symbol;Acc:HGNC:49200]	3.682946
ENSG00000176641	220441	RNF152	protein_coding	HGNC Symbol	5	ring finger protein 152 [Source:HGNC Symbol;Acc:HGNC:26811]	3.684588
ENSG00000258441	283624	LINC00641	processed_transcript	HGNC Symbol	2	long intergenic non-protein coding RNA 641 [Source:HGNC Symbol;Acc:HGNC:27511]	3.69167
ENSG00000184697	9074	CLDN6	protein_coding	HGNC Symbol	3	claudin 6 [Source:HGNC Symbol;Acc:HGNC:2048]	3.692148
ENSG00000170482	9963	SLC23A1	protein_coding	HGNC Symbol	7	solute carrier family 23 member 1 [Source:HGNC Symbol;Acc:HGNC:10974]	3.696172
ENSG00000256533	NA	AP002373.2	processed_pseudogene	Clone-based (Ensembl) gene	1	CCR4 carbon catabolite repression 4-like (S. cerevisiae) (CCRN4L) pseudogene	3.696273
ENSG00000266900	NA	AC027514.1	sense_intronic	Clone-based (Ensembl) gene	1	novel transcript, sense intronic to KIAA1468	3.696842
ENSG00000236754	NA	AC007666.1	antisense	Clone-based (Ensembl) gene	2	novel transcript	3.698881
ENSG00000101082	84174	SLA2	protein_coding	HGNC Symbol	2	Src like adaptor 2 [Source:HGNC Symbol;Acc:HGNC:17329]	3.702058
ENSG00000178776	389336	C5orf46	protein_coding	HGNC Symbol	3	chromosome 5 open reading frame 46 [Source:HGNC Symbol;Acc:HGNC:33768]	3.702702
ENSG00000006194	10127	ZNF263	protein_coding	HGNC Symbol	8	zinc finger protein 263 [Source:HGNC Symbol;Acc:HGNC:13056]	3.713315
ENSG00000263680	NA	AC007639.1	lincRNA	Clone-based (Ensembl) gene	1	novel transcript	3.715153
ENSG00000259030	1.01E+08	FPGT-TNNI3K	protein_coding	HGNC Symbol	7	FPGT-TNNI3K readthrough [Source:HGNC Symbol;Acc:HGNC:42952]	3.718211
ENSG00000268678	NA	AC005261.2	antisense	Clone-based (Ensembl) gene	1	novel transcript, antisense to ZNF304	3.729725
ENSG00000184588	5142	PDE4B	protein_coding	HGNC Symbol	18	phosphodiesterase 4B [Source:HGNC Symbol;Acc:HGNC:8781]	3.72974
ENSG00000236047	NA	AC073410.1	processed_pseudogene	Clone-based (Ensembl) gene	1	ribosomal protein L13a (RPL13A) pseudogene	3.736754
ENSG00000260948	NA	AL390195.2	sense_overlapping	Clone-based (Ensembl) gene	1	novel transcript, sense overlapping WDR77 & OVGP1	3.737312

ENSG00000176761	NA	ZNF285B	unprocessed_pseudogene	HGNC Symbol	1	zinc finger protein 285B (pseudogene) [Source:HGNC Symbol;Acc:HGNC:33262]	3.750955
ENSG00000223547	284391	ZNF844	protein_coding	HGNC Symbol	3	zinc finger protein 844 [Source:HGNC Symbol;Acc:HGNC:25932]	3.754129
ENSG00000180611	151963	MB21D2	protein_coding	HGNC Symbol	1	Mab-21 domain containing 2 [Source:HGNC Symbol;Acc:HGNC:30438]	3.757799
ENSG00000180914	5021	OXTR	protein_coding	HGNC Symbol	4	oxytocin receptor [Source:HGNC Symbol;Acc:HGNC:8529]	3.760683
ENSG00000162614	91624	NEXN	protein_coding	HGNC Symbol	8	nexilin F-actin binding protein [Source:HGNC Symbol;Acc:HGNC:29557]	3.763118
ENSG00000232882	NA	PHKA1P1	processed_pseudogene	HGNC Symbol	1	phosphorylase kinase regulatory subunit alpha 1 pseudogene [Source:HGNC Symbol;Acc:HGN	3.774344
ENSG00000100439	63874	ABHD4	protein_coding	HGNC Symbol	10	abhydrolase domain containing 4 [Source:HGNC Symbol;Acc:HGNC:20154]	3.786381
ENSG00000077616	10003	NAALAD2	protein_coding	HGNC Symbol	10	N-acetylated alpha-linked acidic dipeptidase 2 [Source:HGNC Symbol;Acc:HGNC:14526]	3.787695
ENSG00000229052	NA	AL449283.1	transcribed_processed_pseudogene	Clone-based (Ensembl) gene	2	GAJ protein pseudogene	3.800954
ENSG00000223973	NA	AC068491.1	transcribed_processed_pseudogene	Clone-based (Ensembl) gene	2	platelet-activating factor acetylhydrolase, isoform Ib, alpha subunit 45kDa (PAFAH1B1) pseud	3.82603
ENSG00000198846	9760	TOX	protein_coding	HGNC Symbol	1	thymocyte selection associated high mobility group box [Source:HGNC Symbol;Acc:HGNC:18	3.837474
ENSG00000105443	9266	CYTH2	protein_coding	HGNC Symbol	14	cytohesin 2 [Source:HGNC Symbol;Acc:HGNC:9502]	3.845262
ENSG00000120875	1846	DUSP4	protein_coding	HGNC Symbol	2	dual specificity phosphatase 4 [Source:HGNC Symbol;Acc:HGNC:3070]	3.845985
ENSG00000123358	3164	NR4A1	protein_coding	HGNC Symbol	22	nuclear receptor subfamily 4 group A member 1 [Source:HGNC Symbol;Acc:HGNC:7980]	3.852934
ENSG00000225434	1.01E+08	LINC01504	lincRNA	HGNC Symbol	3	long intergenic non-protein coding RNA 1504 [Source:HGNC Symbol;Acc:HGNC:51185]	3.872598
ENSG00000239686	NA	AL158801.1	processed_pseudogene	Clone-based (Ensembl) gene	1	ribosomal protein L34 (RPL34) pseudogene	3.882687
ENSG00000260317	NA	AC009812.4	lincRNA	Clone-based (Ensembl) gene	1	novel transcript	3.886502
ENSG00000187144	374955	SPATA21	protein_coding	HGNC Symbol	6	spermatogenesis associated 21 [Source:HGNC Symbol;Acc:HGNC:28026]	3.889782
ENSG00000175197	1649	DDIT3	protein_coding	HGNC Symbol	6	DNA damage inducible transcript 3 [Source:HGNC Symbol;Acc:HGNC:2726]	3.892259
ENSG00000269444	NA	AC011491.2	sense_overlapping	Clone-based (Ensembl) gene	1	novel transcript, sense overlapping GTF2F1	3.893811
ENSG00000230701	26226	FBXW4P1	processed_pseudogene	HGNC Symbol	1	F-box and WD repeat domain containing 4 pseudogene 1 [Source:HGNC Symbol;Acc:HGNC:1	3.903971
ENSG00000148841	85450	ITPRIP	protein_coding	HGNC Symbol	5	inositol 1,4,5-trisphosphate receptor interacting protein [Source:HGNC Symbol;Acc:HGNC:29	3.904746
ENSG00000215796	NA	AL512637.1	processed_pseudogene	Clone-based (Ensembl) gene	1	primase polypeptide 1 49kDa (PRIM1) pseudogene	3.911337
ENSG00000234473	NA	ZNF101P2	processed_pseudogene	HGNC Symbol	1	zinc finger protein 101 pseudogene 2 [Source:HGNC Symbol;Acc:HGNC:44942]	3.912982
ENSG00000013588	9052	GPRC5A	protein_coding	HGNC Symbol	6	G protein-coupled receptor class C group 5 member A [Source:HGNC Symbol;Acc:HGNC:9836	3.921436
ENSG00000202314	692075	SNORD6	snoRNA	HGNC Symbol	1	small nucleolar RNA, C/D box 6 [Source:HGNC Symbol;Acc:HGNC:32703]	3.924171
ENSG00000174827	5174	PDZK1	protein_coding	HGNC Symbol	7	PDZ domain containing 1 [Source:HGNC Symbol;Acc:HGNC:8821]	3.9323
ENSG00000120215	2315	MLANA	protein_coding	HGNC Symbol	5	melan-A [Source:HGNC Symbol;Acc:HGNC:7124]	3.945934
ENSG00000259326	NA	AC116158.1	antisense	Clone-based (Ensembl) gene	1	novel transcript, antisense to RASGRP1	3.949122
ENSG00000154027	26289	AK5	protein_coding	HGNC Symbol	13	adenylate kinase 5 [Source:HGNC Symbol;Acc:HGNC:365]	3.959032
ENSG00000143178	9095	TBX19	protein_coding	HGNC Symbol	4	T-box 19 [Source:HGNC Symbol;Acc:HGNC:11596]	3.967285
ENSG00000241217	NA	RN7SL809P	misc_RNA	HGNC Symbol	1	RNA, 7SL, cytoplasmic 809, pseudogene [Source:HGNC Symbol;Acc:HGNC:46825]	3.96851
ENSG00000165194	57526	PCDH19	protein_coding	HGNC Symbol	5	protocadherin 19 [Source:HGNC Symbol;Acc:HGNC:14270]	3.983656
ENSG00000226849	NA	AL109811.1	antisense	Clone-based (Ensembl) gene	1	novel transcript	3.988097
ENSG00000249609	NA	AC080188.1	antisense	Clone-based (Ensembl) gene	1	novel transcript	3.992975
ENSG00000275700	26574	AATF	protein_coding	HGNC Symbol	9	apoptosis antagonizing transcription factor [Source:HGNC Symbol;Acc:HGNC:19235]	3.999932
ENSG00000213693	NA	SEC14L1P1	processed_pseudogene	HGNC Symbol	1	SEC14 like 1 pseudogene 1 [Source:HGNC Symbol;Acc:HGNC:44189]	4.002915
ENSG00000120129	1843	DUSP1	protein_coding	HGNC Symbol	1	dual specificity phosphatase 1 [Source:HGNC Symbol;Acc:HGNC:3064]	4.022517
ENSG00000144655	64651	CSRNP1	protein_coding	HGNC Symbol	2	cysteine and serine rich nuclear protein 1 [Source:HGNC Symbol;Acc:HGNC:14300]	4.022527
ENSG00000235027	NA	AC068580.3	antisense	Clone-based (Ensembl) gene	1	novel transcript	4.030956
ENSG00000250444	NA	CCT5P1	processed_pseudogene	HGNC Symbol	1	chaperonin containing TCP1 subunit 5 pseudogene 1 [Source:HGNC Symbol;Acc:HGNC:35135	4.039382
ENSG00000182511	2242	FES	protein_coding	HGNC Symbol	17	FES proto-oncogene, tyrosine kinase [Source:HGNC Symbol;Acc:HGNC:3657]	4.070321
ENSG00000224316	NA	GTF2IP5	unprocessed_pseudogene	HGNC Symbol	1	general transcription factor IIi pseudogene 5 [Source:HGNC Symbol;Acc:HGNC:51717]	4.074547
ENSG00000246731	85001	MGC16275	antisense	NCBI gene	3	uncharacterized protein MGC16275 [Source:NCBI gene;Acc:85001]	4.076411
ENSG00000277481	342372	PKD1L3	protein_coding	HGNC Symbol	1	polycystin 1 like 3, transient receptor potential channel interacting [Source:HGNC Symbol;Acc	4.07878
ENSG00000273038	NA	AL365203.2	lincRNA	Clone-based (Ensembl) gene	1	novel transcript	4.085196
ENSG00000228366	NA	AL592437.1	processed_pseudogene	Clone-based (Ensembl) gene	1	FUS interacting protein (serine-arginine rich) 1 (FUSIP1) pseudogene	4.090663
ENSG00000255815	NA	KRT8P11	processed_pseudogene	HGNC Symbol	1	keratin 8 pseudogene 11 [Source:HGNC Symbol;Acc:HGNC:31058]	4.091513
ENSG00000228995	NA	MTND1P9	processed_pseudogene	HGNC Symbol	1	MT-ND1 pseudogene 9 [Source:HGNC Symbol;Acc:HGNC:42099]	4.107933
ENSG00000183977	151649	PP2D1	protein_coding	HGNC Symbol	3	protein phosphatase 2C like domain containing 1 [Source:HGNC Symbol;Acc:HGNC:28406]	4.109471
ENSG00000273416	NA	AL732292.2	lincRNA	Clone-based (Ensembl) gene	1	novel transcript	4.114686
ENSG00000250995	NA	AL391280.1	processed_pseudogene	Clone-based (Ensembl) gene	1	novel pseudogene	4.12148
ENSG00000165972	120935	CCDC38	protein_coding	HGNC Symbol	8	coiled-coil domain containing 38 [Source:HGNC Symbol;Acc:HGNC:26843]	4.130596
ENSG00000100285	4744	NEFH	protein_coding	HGNC Symbol	1	neurofilament heavy [Source:HGNC Symbol;Acc:HGNC:7737]	4.131046
ENSG00000269365	NA	AC120349.1	antisense	Clone-based (Ensembl) gene	1	novel transcript	4.133125
ENSG00000233613	NA	DCUN1D2-AS	antisense	HGNC Symbol	2	DCUN1D2 antisense RNA [Source:HGNC Symbol;Acc:HGNC:39889]	4.14323
ENSG00000164082	2912	GRM2	protein_coding	HGNC Symbol	8	glutamate metabotropic receptor 2 [Source:HGNC Symbol;Acc:HGNC:4594]	4.144574
ENSG00000118946	27253	PCDH17	protein_coding	HGNC Symbol	4	protocadherin 17 [Source:HGNC Symbol;Acc:HGNC:14267]	4.149222
ENSG00000142871	3491	CYR61	protein_coding	HGNC Symbol	2	cysteine rich angiogenic inducer 61 [Source:HGNC Symbol;Acc:HGNC:2654]	4.164564
ENSG00000256222	1E+08	MTRNR2L3	protein_coding	HGNC Symbol	1	MT-RNR2 like 3 [Source:HGNC Symbol;Acc:HGNC:37157]	4.167984
ENSG00000165443	84457	PHYHIPL	protein_coding	HGNC Symbol	4	phytanoyl-CoA 2-hydroxylase interacting protein like [Source:HGNC Symbol;Acc:HGNC:29378	4.175198
ENSG00000196159	79633	FAT4	protein_coding	HGNC Symbol	3	FAT atypical cadherin 4 [Source:HGNC Symbol;Acc:HGNC:23109]	4.182717
ENSG00000223861	NA	AL365436.1	processed_pseudogene	Clone-based (Ensembl) gene	1	ribosomal protein S10 (RPS10) pseudogene	4.19053
ENSG00000095739	25805	BAMBI	protein_coding	HGNC Symbol	2	BMP and activin membrane bound inhibitor [Source:HGNC Symbol;Acc:HGNC:30251]	4.207723
ENSG00000225402	NA	AC010878.1	processed_pseudogene	Clone-based (Ensembl) gene	1	serine/threonine kinase receptor associated protein (STRAP) pseudogene	4.208387

ENSG00000227123	NA	RPL12P44	processed_pseudogene	HGNC Symbol	1	ribosomal protein L12 pseudogene 44 [Source:HGNC Symbol;Acc:HGNC:44603]	4.213608
ENSG00000137273	2295	FOXF2	protein_coding	HGNC Symbol	2	forkhead box F2 [Source:HGNC Symbol;Acc:HGNC:3810]	4.218734
ENSG00000272895	NA	AC009303.3	antisense	Clone-based (Ensembl) gene	1	novel transcript, antisense to CCDC93	4.223897
ENSG00000108551	51655	RASD1	protein_coding	HGNC Symbol	2	ras related dexamethasone induced 1 [Source:HGNC Symbol;Acc:HGNC:15828]	4.229381
ENSG00000237101	1.02E+08	AC092809.4	antisense	Clone-based (Ensembl) gene	1		4.235149
ENSG00000272034	26822	SNORD14A	snoRNA	HGNC Symbol	1	small nucleolar RNA, C/D box 14A [Source:HGNC Symbol;Acc:HGNC:10113]	4.261893
ENSG00000235284	26786	SNORD62A	snoRNA	HGNC Symbol	1	small nucleolar RNA, C/D box 62A [Source:HGNC Symbol;Acc:HGNC:10219]	4.264561
ENSG00000274950	6203	RPS9	protein_coding	HGNC Symbol	15	ribosomal protein S9 [Source:HGNC Symbol;Acc:HGNC:10442]	4.277353
ENSG00000174307	23612	PHLDA3	protein_coding	HGNC Symbol	4	pleckstrin homology like domain family A member 3 [Source:HGNC Symbol;Acc:HGNC:8934]	4.289606
ENSG00000198822	2913	GRM3	protein_coding	HGNC Symbol	5	glutamate metabotropic receptor 3 [Source:HGNC Symbol;Acc:HGNC:4595]	4.298629
ENSG00000177519	56475	RPRM	protein_coding	HGNC Symbol	1	reprimo, TP53 dependent G2 arrest mediator homolog [Source:HGNC Symbol;Acc:HGNC:242	4.300505
ENSG00000159251	70	ACTC1	protein_coding	HGNC Symbol	6	actin, alpha, cardiac muscle 1 [Source:HGNC Symbol;Acc:HGNC:143]	4.317705
ENSG00000273951	NA	AL031667.3	sense_intronic	Clone-based (Ensembl) gene	1	novel transcript, sense intronic to CHD6	4.335023
ENSG00000126759	5199	CFP	protein_coding	HGNC Symbol	8	complement factor properdin [Source:HGNC Symbol;Acc:HGNC:8864]	4.336829
ENSG00000150457	26524	LATS2	protein_coding	HGNC Symbol	2	large tumor suppressor kinase 2 [Source:HGNC Symbol;Acc:HGNC:6515]	4.353989
ENSG00000232034	NA	AC092168.2	sense_intronic	Clone-based (Ensembl) gene	1	novel transcript	4.384015
ENSG00000059728	4084	MXD1	protein_coding	HGNC Symbol	6	MAX dimerization protein 1 [Source:HGNC Symbol;Acc:HGNC:6761]	4.384114
ENSG00000109771	55805	LRP2BP	protein_coding	HGNC Symbol	4	LRP2 binding protein [Source:HGNC Symbol;Acc:HGNC:25434]	4.391979
ENSG00000273311	25786	DGCR11	sense_intronic	HGNC Symbol	1	DiGeorge syndrome critical region gene 11 [Source:HGNC Symbol;Acc:HGNC:17226]	4.396922
ENSG00000109927	7007	TECTA	protein_coding	HGNC Symbol	6	tectorin alpha [Source:HGNC Symbol;Acc:HGNC:11720]	4.402448
ENSG00000201457	677834	SNORA55	snoRNA	HGNC Symbol	1	small nucleolar RNA, H/ACA box 55 [Source:HGNC Symbol;Acc:HGNC:32649]	4.407052
ENSG00000276814	NA	AC004801.6	lincRNA	Clone-based (Ensembl) gene	1	novel transcript	4.432587
ENSG00000116819	339488	TFAP2E	protein_coding	HGNC Symbol	2	transcription factor AP-2 epsilon [Source:HGNC Symbol;Acc:HGNC:30774]	4.433762
ENSG00000119508	8013	NR4A3	protein_coding	HGNC Symbol	4	nuclear receptor subfamily 4 group A member 3 [Source:HGNC Symbol;Acc:HGNC:7982]	4.435313
ENSG00000130222	10912	GADD45G	protein_coding	HGNC Symbol	3	growth arrest and DNA damage inducible gamma [Source:HGNC Symbol;Acc:HGNC:4097]	4.445786
ENSG00000274569	NA	AC024884.1	processed_pseudogene	Clone-based (Ensembl) gene	1	apolipoprotein F (APOF) pseudogene	4.45245
ENSG00000081985	3595	IL12RB2	protein_coding	HGNC Symbol	7	interleukin 12 receptor subunit beta 2 [Source:HGNC Symbol;Acc:HGNC:5972]	4.455161
ENSG00000260171	NA	AC008269.2	lincRNA	Clone-based (Ensembl) gene	1	novel transcript	4.485153
ENSG00000241984	NA	RPL7AP2	processed_pseudogene	HGNC Symbol	1	ribosomal protein L7a pseudogene 2 [Source:HGNC Symbol;Acc:HGNC:23552]	4.489009
ENSG00000125997	NA	BPIFB9P	transcribed_unitary_pseudogene	HGNC Symbol	2	BPI fold containing family B member 9, pseudogene [Source:HGNC Symbol;Acc:HGNC:16109]	4.514658
ENSG00000273618	NA	AL135999.2	antisense	Clone-based (Ensembl) gene	1	TEC	4.559167
ENSG00000275956	NA	AC104446.1	processed_pseudogene	Clone-based (Ensembl) gene	1	zinc finger protein 610 (ZNF610) pseudogene	4.560905
ENSG00000136826	9314	KLF4	protein_coding	HGNC Symbol	6	Kruppel like factor 4 [Source:HGNC Symbol;Acc:HGNC:6348]	4.572267
ENSG00000274976	NA	AC087588.2	sense_intronic	Clone-based (Ensembl) gene	1	novel transcript, sense intronic to PKP2	4.574448
ENSG00000008128	728642	CDK11A	protein_coding	HGNC Symbol	23	cyclin dependent kinase 11A [Source:HGNC Symbol;Acc:HGNC:1730]	4.582144
ENSG00000236430	NA	KRT8P29	processed_pseudogene	HGNC Symbol	1	keratin 8 pseudogene 29 [Source:HGNC Symbol;Acc:HGNC:33381]	4.587167
ENSG00000125266	1948	EFNB2	protein_coding	HGNC Symbol	3	ephrin B2 [Source:HGNC Symbol;Acc:HGNC:3227]	4.602376
ENSG00000235602	642559	POU5F1P3	processed_pseudogene	HGNC Symbol	1	POU class 5 homeobox 1 pseudogene 3 [Source:HGNC Symbol;Acc:HGNC:9222]	4.610994
ENSG00000230262	158257	LINC02603	lincRNA	HGNC Symbol	2	long intergenic non-protein coding RNA 2603 [Source:HGNC Symbol;Acc:HGNC:37186]	4.629185
ENSG00000257497	NA	AC121761.1	antisense	Clone-based (Ensembl) gene	1	novel transcript, antisense to GLIPR1	4.632087
ENSG00000224080	NA	UBE2FP1	processed_pseudogene	HGNC Symbol	1	UBE2F pseudogene 1 [Source:HGNC Symbol;Acc:HGNC:44535]	4.633655
ENSG00000234937	NA	AL139128.1	processed_pseudogene	Clone-based (Ensembl) gene	1	proteasome (prosome, macropain) 26S subunit, non-ATPase, 7 (Mov34 homolog) (PSMD7) ps	4.637211
ENSG00000143382	54507	ADAMTSL4	protein_coding	HGNC Symbol	6	ADAMTS like 4 [Source:HGNC Symbol;Acc:HGNC:19706]	4.639271
ENSG00000171189	2897	GRIK1	protein_coding	HGNC Symbol	8	glutamate ionotropic receptor kainate type subunit 1 [Source:HGNC Symbol;Acc:HGNC:4579]	4.650899
ENSG00000146592	9586	CREB5	protein_coding	HGNC Symbol	15	cAMP responsive element binding protein 5 [Source:HGNC Symbol;Acc:HGNC:16844]	4.661245
ENSG00000112599	2979	GUCA1B	protein_coding	HGNC Symbol	1	guanylate cyclase activator 1B [Source:HGNC Symbol;Acc:HGNC:4679]	4.680626
ENSG00000259818	NA	AL606760.3	antisense	Clone-based (Ensembl) gene	1	novel transcript, antisense to C1orf123 & MAGOH	4.701825
ENSG00000232709	NA	MARK2P9	processed_pseudogene	HGNC Symbol	1	microtubule affinity regulating kinase 2 pseudogene 9 [Source:HGNC Symbol;Acc:HGNC:3980	4.708796
ENSG00000188761	440603	BCL2L15	protein_coding	HGNC Symbol	7	BCL2 like 15 [Source:HGNC Symbol;Acc:HGNC:33624]	4.732518
ENSG00000139625	7786	MAP3K12	protein_coding	HGNC Symbol	11	mitogen-activated protein kinase kinase kinase 12 [Source:HGNC Symbol;Acc:HGNC:6851]	4.733485
ENSG00000164683	23462	HEY1	protein_coding	HGNC Symbol	8	hes related family bHLH transcription factor with YRPW motif 1 [Source:HGNC Symbol;Acc:HG	4.744836
ENSG00000038295	7092	TLL1	protein_coding	HGNC Symbol	6	tolloid like 1 [Source:HGNC Symbol;Acc:HGNC:11843]	4.755444
ENSG00000236811	NA	GAPDHP2	processed_pseudogene	HGNC Symbol	1	glyceraldehyde-3-phosphate dehydrogenase pseudogene 2 [Source:HGNC Symbol;Acc:HGNC:	4.764644
ENSG00000119608	283571	PROX2	protein_coding	HGNC Symbol	3	prospero homeobox 2 [Source:HGNC Symbol;Acc:HGNC:26715]	4.768363
ENSG00000104221	55290	BRF2	protein_coding	HGNC Symbol	4	BRF2, RNA polymerase III transcription initiation factor subunit [Source:HGNC Symbol;Acc:HG	4.783022
ENSG00000182183	348378	SHISAL2A	protein_coding	HGNC Symbol	4	shisa like 2A [Source:HGNC Symbol;Acc:HGNC:28757]	4.786448
ENSG00000135604	8676	STX11	protein_coding	HGNC Symbol	1	syntaxin 11 [Source:HGNC Symbol;Acc:HGNC:11429]	4.797435
ENSG00000179921	151306	GPBAR1	protein_coding	HGNC Symbol	4	G protein-coupled bile acid receptor 1 [Source:HGNC Symbol;Acc:HGNC:19680]	4.833102
ENSG00000140795	91807	MYLK3	protein_coding	HGNC Symbol	7	myosin light chain kinase 3 [Source:HGNC Symbol;Acc:HGNC:29826]	4.839631
ENSG00000227456	114036	LINC00310	lincRNA	HGNC Symbol	8	long intergenic non-protein coding RNA 310 [Source:HGNC Symbol;Acc:HGNC:16414]	4.849508
ENSG00000254162	NA	AC009812.3	lincRNA	Clone-based (Ensembl) gene	1	novel transcript	4.85382
ENSG00000113070	1839	HBEGF	protein_coding	HGNC Symbol	3	heparin binding EGF like growth factor [Source:HGNC Symbol;Acc:HGNC:3059]	4.880539
ENSG00000229808	NA	AL391825.1	transcribed_processed_pseudogene	Clone-based (Ensembl) gene	2	proteasome (prosome, macropain) activator subunit 3 (PA28 gamma; Ki) pseudogene	4.90645
ENSG00000230584	NA	CCT5P2	processed_pseudogene	HGNC Symbol	1	chaperonin containing TCP1 subunit 5 pseudogene 2 [Source:HGNC Symbol;Acc:HGNC:35139	4.910758
ENSG00000207088	677797	SNORA7B	snoRNA	HGNC Symbol	1	small nucleolar RNA, H/ACA box 7B [Source:HGNC Symbol;Acc:HGNC:32593]	4.914271

ENSG00000165197	2277	VEGFD	protein_coding	HGNC Symbol	2	vascular endothelial growth factor D [Source:HGNC Symbol;Acc:HGNC:3708]	4.924828
ENSG00000185883	527	ATP6V0C	protein_coding	HGNC Symbol	4	ATPase H+ transporting V0 subunit c [Source:HGNC Symbol;Acc:HGNC:855]	4.925271
ENSG00000258851	NA	AL139300.2	antisense	Clone-based (Ensembl) gene	1	novel transcript, antisense to BAG5	4.930721
ENSG00000260459	NA	FTLP14	processed_pseudogene	HGNC Symbol	1	ferritin light chain pseudogene 14 [Source:HGNC Symbol;Acc:HGNC:37964]	4.959417
ENSG00000139547	8608	RDH16	protein_coding	HGNC Symbol	2	retinol dehydrogenase 16 [Source:HGNC Symbol;Acc:HGNC:29674]	4.969828
ENSG00000256037	NA	MRPL40P1	processed_pseudogene	HGNC Symbol	1	mitochondrial ribosomal protein L40 pseudogene 1 [Source:HGNC Symbol;Acc:HGNC:44532]	5.001058
ENSG00000105146	6795	AURKC	protein_coding	HGNC Symbol	8	aurora kinase C [Source:HGNC Symbol;Acc:HGNC:11391]	5.036561
ENSG00000174776	151790	WDR49	protein_coding	HGNC Symbol	10	WD repeat domain 49 [Source:HGNC Symbol;Acc:HGNC:26587]	5.039414
ENSG00000115963	390	RND3	protein_coding	HGNC Symbol	9	Rho family GTPase 3 [Source:HGNC Symbol;Acc:HGNC:671]	5.051795
ENSG00000100292	3162	HMOX1	protein_coding	HGNC Symbol	4	heme oxygenase 1 [Source:HGNC Symbol;Acc:HGNC:5013]	5.05976
ENSG00000148926	133	ADM	protein_coding	HGNC Symbol	8	adrenomedullin [Source:HGNC Symbol;Acc:HGNC:259]	5.063719
ENSG00000154736	11096	ADAMTS5	protein_coding	HGNC Symbol	1	ADAM metallopeptidase with thrombospondin type 1 motif 5 [Source:HGNC Symbol;Acc:HGN	5.076631
ENSG00000173269	79812	MMRN2	protein_coding	HGNC Symbol	7	multimerin 2 [Source:HGNC Symbol;Acc:HGNC:19888]	5.090807
ENSG00000276334	NA	AL133243.2	sense_intronic	Clone-based (Ensembl) gene	1	novel transcript, sense intronic to BIRC6	5.109009
ENSG00000213234	NA	ST13P10	processed_pseudogene	HGNC Symbol	1	ST13, Hsp70 interacting protein pseudogene 10 [Source:HGNC Symbol;Acc:HGNC:38745]	5.112743
ENSG00000235513	1.01E+08	AL035681.1	antisense	Clone-based (Ensembl) gene	2	novel transcript, antisense to L3MBTL2	5.154187
ENSG00000274928	NA	KRT89P	transcribed_unprocessed_pseudogene	HGNC Symbol	2	keratin 89 pseudogene [Source:HGNC Symbol;Acc:HGNC:30196]	5.172383
ENSG00000252652	NA	RF00019	misc_RNA	RFAM	1		5.176204
ENSG00000175820	643677	CCDC168	protein_coding	HGNC Symbol	1	coiled-coil domain containing 168 [Source:HGNC Symbol;Acc:HGNC:26851]	5.181461
ENSG00000229512	NA	AC068580.1	sense_intronic	Clone-based (Ensembl) gene	1	novel transcript	5.189393
ENSG00000245080	1.05E+08	MIR3150BHG	antisense	HGNC Symbol	2	MIR3150B host gene [Source:HGNC Symbol;Acc:HGNC:52000]	5.226378
ENSG00000249661	NA	TNRC18P1	processed_pseudogene	HGNC Symbol	1	trinucleotide repeat containing 18 pseudogene 1 [Source:HGNC Symbol;Acc:HGNC:43881]	5.250381
ENSG00000135116	8739	HRK	protein_coding	HGNC Symbol	4	harakiri, BCL2 interacting protein [Source:HGNC Symbol;Acc:HGNC:5185]	5.27939
ENSG00000224746	NA	AC015987.1	antisense	Clone-based (Ensembl) gene	1	novel transcript, antisense to MTPN	5.288245
ENSG00000031081	57514	ARHGAP31	protein_coding	HGNC Symbol	2	Rho GTPase activating protein 31 [Source:HGNC Symbol;Acc:HGNC:29216]	5.289316
ENSG00000226800	404665	CACTIN-AS1	antisense	HGNC Symbol	1	CACTIN antisense RNA 1 [Source:HGNC Symbol;Acc:HGNC:31391]	5.298847
ENSG00000240106	NA	RN7SL146P	misc_RNA	HGNC Symbol	1	RNA, 7SL, cytoplasmic 146, pseudogene [Source:HGNC Symbol;Acc:HGNC:46162]	5.302107
ENSG00000227688	NA	HNRNPA3P2	unprocessed_pseudogene	HGNC Symbol	1	heterogeneous nuclear ribonucleoprotein A3 pseudogene 2 [Source:HGNC Symbol;Acc:HGNC	5.311394
ENSG00000153234	4929	NR4A2	protein_coding	HGNC Symbol	10	nuclear receptor subfamily 4 group A member 2 [Source:HGNC Symbol;Acc:HGNC:7981]	5.355787
ENSG00000196114	NA	AL031577.1	processed_pseudogene	Clone-based (Ensembl) gene	1	interferon induced transmembrane protein (IFITM) pseudogene	5.35659
ENSG00000264175	1E+08	MIR3189	miRNA	HGNC Symbol	1	microRNA 3189 [Source:HGNC Symbol;Acc:HGNC:38307]	5.37961
ENSG00000268307	1.11E+08	LINC02560	lincRNA	HGNC Symbol	1	long intergenic non-protein coding RNA 2560 [Source:HGNC Symbol;Acc:HGNC:53600]	5.425144
ENSG00000185758	1E+08	CLDN24	protein_coding	HGNC Symbol	1	claudin 24 [Source:HGNC Symbol;Acc:HGNC:37200]	5.438511
ENSG00000185950	8660	IRS2	protein_coding	HGNC Symbol	1	insulin receptor substrate 2 [Source:HGNC Symbol;Acc:HGNC:6126]	5.463933
ENSG00000276926	1.02E+08	MIR6797	miRNA	HGNC Symbol	1	microRNA 6797 [Source:HGNC Symbol;Acc:HGNC:50169]	5.514903
ENSG00000162552	54361	WNT4	protein_coding	HGNC Symbol	3	Wnt family member 4 [Source:HGNC Symbol;Acc:HGNC:12783]	5.529005
ENSG00000189030	391104	VHLL	protein_coding	HGNC Symbol	1	VHL like [Source:HGNC Symbol;Acc:HGNC:30666]	5.548542
ENSG00000256108	NA	AC135586.1	processed_pseudogene	Clone-based (Ensembl) gene	1	tumor protein D52-like 2 (TPD52L2) pseudogene	5.569455
ENSG00000118514	64577	ALDH8A1	protein_coding	HGNC Symbol	6	aldehyde dehydrogenase 8 family member A1 [Source:HGNC Symbol;Acc:HGNC:15471]	5.604078
ENSG00000144460	57624	NYAP2	protein_coding	HGNC Symbol	2	neuronal tyrosine-phosphorylated phosphoinositide-3-kinase adaptor 2 [Source:HGNC Symbo	5.659198
ENSG00000266708	NA	AP001793.1	antisense	Clone-based (Ensembl) gene	1	novel transcript, antisense to RAB12	5.671243
ENSG00000023445	330	BIRC3	protein_coding	HGNC Symbol	7	baculoviral IAP repeat containing 3 [Source:HGNC Symbol;Acc:HGNC:591]	5.676483
ENSG00000065618	1308	COL17A1	protein_coding	HGNC Symbol	11	collagen type XVII alpha 1 chain [Source:HGNC Symbol;Acc:HGNC:2194]	5.704312
ENSG00000273611	9326	ZNHIT3	protein_coding	HGNC Symbol	12	zinc finger HIT-type containing 3 [Source:HGNC Symbol;Acc:HGNC:12309]	5.718313
ENSG00000213626	81606	LBH	protein_coding	HGNC Symbol	9	limb bud and heart development [Source:HGNC Symbol;Acc:HGNC:29532]	5.754953
ENSG00000258096	NA	AC025031.2	antisense	Clone-based (Ensembl) gene	1	novel transcript, antisense to SLC38A2	5.763637
ENSG00000258951	NA	KRT18P7	processed_pseudogene	HGNC Symbol	1	keratin 18 pseudogene 7 [Source:HGNC Symbol;Acc:HGNC:20283]	5.769145
ENSG00000141448	2627	GATA6	protein_coding	HGNC Symbol	2	GATA binding protein 6 [Source:HGNC Symbol;Acc:HGNC:4174]	5.784475
ENSG00000087494	5744	PTHLH	protein_coding	HGNC Symbol	9	parathyroid hormone like hormone [Source:HGNC Symbol;Acc:HGNC:9607]	5.787987
ENSG00000274677	NA	AC040169.3	sense_intronic	Clone-based (Ensembl) gene	1	novel transcript, sense intronic to MBTPS1	5.800754
ENSG00000143110	128346	C1orf162	protein_coding	HGNC Symbol	3	chromosome 1 open reading frame 162 [Source:HGNC Symbol;Acc:HGNC:28344]	5.812791
ENSG00000075426	2355	FOSL2	protein_coding	HGNC Symbol	4	FOS like 2, AP-1 transcription factor subunit [Source:HGNC Symbol;Acc:HGNC:3798]	5.841027
ENSG00000254248	NA	AC068189.1	antisense	Clone-based (Ensembl) gene	1	novel transcript, antisense to NDUFAF6	5.855949
ENSG00000116717	1647	GADD45A	protein_coding	HGNC Symbol	7	growth arrest and DNA damage inducible alpha [Source:HGNC Symbol;Acc:HGNC:4095]	5.879083
ENSG00000154734	9510	ADAMTS1	protein_coding	HGNC Symbol	6	ADAM metallopeptidase with thrombospondin type 1 motif 1 [Source:HGNC Symbol;Acc:HGN	5.881435
ENSG00000227290	1.01E+08	LINC01364	lincRNA	HGNC Symbol	1	long intergenic non-protein coding RNA 1364 [Source:HGNC Symbol;Acc:HGNC:50599]	5.910852
ENSG00000270499	NA	MKI67P1	processed_pseudogene	HGNC Symbol	1	marker of proliferation Ki-67 pseudogene 1 [Source:HGNC Symbol;Acc:HGNC:49230]	5.93317
ENSG00000185668	5453	POU3F1	protein_coding	HGNC Symbol	1	POU class 3 homeobox 1 [Source:HGNC Symbol;Acc:HGNC:9214]	5.959034
ENSG00000266922	NA	AC008543.3	sense_intronic	Clone-based (Ensembl) gene	1	novel transcript, sense intronic to ZNF823	5.962264
ENSG00000148677	27063	ANKRD1	protein_coding	HGNC Symbol	1	ankyrin repeat domain 1 [Source:HGNC Symbol;Acc:HGNC:15819]	5.966135
ENSG00000143199	55811	ADCY10	protein_coding	HGNC Symbol	5	adenylate cyclase 10 [Source:HGNC Symbol;Acc:HGNC:21285]	6.035932
ENSG00000164411	375519	GJB7	protein_coding	HGNC Symbol	3	gap junction protein beta 7 [Source:HGNC Symbol;Acc:HGNC:16690]	6.045388
ENSG00000274397	NA	AL357153.4	unprocessed_pseudogene	Clone-based (Ensembl) gene	1	ADAM metallopeptidase domain 20 (ADAM20) pseudogene	6.04857
ENSG00000078401	1906	EDN1	protein_coding	HGNC Symbol	1	endothelin 1 [Source:HGNC Symbol;Acc:HGNC:3176]	6.10544
ENSG00000222937	NA	SNORD63B	snoRNA	HGNC Symbol	1	small nucleolar RNA, C/D box 63B [Source:HGNC Symbol;Acc:HGNC:52230]	6.121284

ENSG00000205181	149837	LINC00654	lincRNA	HGNC Symbol	2	long intergenic non-protein coding RNA 654 [Source:HGNC Symbol;Acc:HGNC:27154]	6.137256
ENSG00000106113	1395	CRHR2	protein_coding	HGNC Symbol	8	corticotropin releasing hormone receptor 2 [Source:HGNC Symbol;Acc:HGNC:2358]	6.162853
ENSG00000182963	10052	GJC1	protein_coding	HGNC Symbol	9	gap junction protein gamma 1 [Source:HGNC Symbol;Acc:HGNC:4280]	6.201749
ENSG00000150551	116372	LYPD1	protein_coding	HGNC Symbol	3	LY6/PLAUR domain containing 1 [Source:HGNC Symbol;Acc:HGNC:28431]	6.216509
ENSG00000113249	26762	HAVCR1	protein_coding	HGNC Symbol	6	hepatitis A virus cellular receptor 1 [Source:HGNC Symbol;Acc:HGNC:17866]	6.253475
ENSG00000115896	5334	PLCL1	protein_coding	HGNC Symbol	6	phospholipase C like 1 (inactive) [Source:HGNC Symbol;Acc:HGNC:9063]	6.326631
ENSG00000269560	NA	AC010422.4	sense_intronic	Clone-based (Ensembl) gene	1	novel transcript, sense intronic to ZNF564	6.332945
ENSG00000177238	493829	TRIM72	protein_coding	HGNC Symbol	2	tripartite motif containing 72 [Source:HGNC Symbol;Acc:HGNC:32671]	6.352686
ENSG00000186714	493860	CCDC73	protein_coding	HGNC Symbol	4	coiled-coil domain containing 73 [Source:HGNC Symbol;Acc:HGNC:23261]	6.378435
ENSG00000237781	NA	AL356356.1	antisense	Clone-based (Ensembl) gene	1	novel transcript, antisense to ADAMTSL4	6.392551
ENSG00000237437	NA	ASS1P12	processed_pseudogene	HGNC Symbol	1	argininosuccinate synthetase 1 pseudogene 12 [Source:HGNC Symbol;Acc:HGNC:762]	6.422596
ENSG00000167615	114823	LENG8	protein_coding	HGNC Symbol	9	leukocyte receptor cluster member 8 [Source:HGNC Symbol;Acc:HGNC:15500]	6.444995
ENSG00000086159	363	AQP6	protein_coding	HGNC Symbol	5	aquaporin 6 [Source:HGNC Symbol;Acc:HGNC:639]	6.640243
ENSG00000187950	341350	OVCH1	protein_coding	HGNC Symbol	3	ovochymase 1 [Source:HGNC Symbol;Acc:HGNC:23080]	6.683102
ENSG00000172062	6606	SMN1	protein_coding	HGNC Symbol	11	survival of motor neuron 1, telomeric [Source:HGNC Symbol;Acc:HGNC:11117]	6.751258
ENSG00000118515	6446	SGK1	protein_coding	HGNC Symbol	30	serum/glucocorticoid regulated kinase 1 [Source:HGNC Symbol;Acc:HGNC:10810]	6.872413
ENSG00000107984	22943	DKK1	protein_coding	HGNC Symbol	4	dickkopf WNT signaling pathway inhibitor 1 [Source:HGNC Symbol;Acc:HGNC:2891]	6.915856
ENSG00000087074	23645	PPP1R15A	protein_coding	HGNC Symbol	2	protein phosphatase 1 regulatory subunit 15A [Source:HGNC Symbol;Acc:HGNC:14375]	6.939861
ENSG00000231966	NA	AL359853.2	lincRNA	Clone-based (Ensembl) gene	1	novel transcript	6.963802
ENSG00000178732	2814	GP5	protein_coding	HGNC Symbol	1	glycoprotein V platelet [Source:HGNC Symbol;Acc:HGNC:4443]	6.964008
ENSG00000198881	142689	ASB12	protein_coding	HGNC Symbol	1	ankyrin repeat and SOCS box containing 12 [Source:HGNC Symbol;Acc:HGNC:19763]	7.028119
ENSG00000151365	7069	THRSP	protein_coding	HGNC Symbol	1	thyroid hormone responsive [Source:HGNC Symbol;Acc:HGNC:11800]	7.04874
ENSG00000265206	NA	AC004687.1	antisense	Clone-based (Ensembl) gene	1	novel transcript, MIR142 host	7.07894
ENSG00000100024	51733	UPB1	protein_coding	HGNC Symbol	5	beta-ureidopropionase 1 [Source:HGNC Symbol;Acc:HGNC:16297]	7.134002
ENSG00000276945	NA	AC008498.1	unprocessed_pseudogene	Clone-based (Ensembl) gene	1	protocadherin 19 (PCDH19) pseudogene	7.138945
ENSG00000220557	NA	HMGB1P13	processed_pseudogene	HGNC Symbol	1	high mobility group box 1 pseudogene 13 [Source:HGNC Symbol;Acc:HGNC:13318]	7.196082
ENSG00000275846	NA	AL513548.3	lincRNA	Clone-based (Ensembl) gene	2		7.219053
ENSG00000206989	26785	SNORD63	snoRNA	HGNC Symbol	1	small nucleolar RNA, C/D box 63 [Source:HGNC Symbol;Acc:HGNC:10220]	7.243291
ENSG00000235076	NA	GAPDHP52	processed_pseudogene	HGNC Symbol	1	glyceraldehyde 3 phosphate dehydrogenase pseudogene 52 [Source:HGNC Symbol;Acc:HGNC	7.400904
ENSG00000158445	3745	KCNB1	protein_coding	HGNC Symbol	9	potassium voltage-gated channel subfamily B member 1 [Source:HGNC Symbol;Acc:HGNC:62	7.416489
ENSG00000085465	5016	OVGP1	protein_coding	HGNC Symbol	3	oviductal glycoprotein 1 [Source:HGNC Symbol;Acc:HGNC:8524]	7.602583
ENSG00000101306	85366	MYLK2	protein_coding	HGNC Symbol	3	myosin light chain kinase 2 [Source:HGNC Symbol;Acc:HGNC:16243]	7.647186
ENSG00000117477	57821	CCDC181	protein_coding	HGNC Symbol	7	coiled-coil domain containing 181 [Source:HGNC Symbol;Acc:HGNC:28051]	7.698939
ENSG00000175592	8061	FOSL1	protein_coding	HGNC Symbol	5	FOS like 1, AP-1 transcription factor subunit [Source:HGNC Symbol;Acc:HGNC:13718]	7.706907
ENSG00000106366	5054	SERPINE1	protein_coding	HGNC Symbol	1	serpin family E member 1 [Source:HGNC Symbol;Acc:HGNC:8583]	7.781421
ENSG00000255883	NA	FUNDC2P1	processed_pseudogene	HGNC Symbol	1	FUN14 domain containing 2 pseudogene 1 [Source:HGNC Symbol;Acc:HGNC:17253]	7.789112
ENSG00000260368	NA	AC027373.1	sense_overlapping	Clone-based (Ensembl) gene	1	novel transcript, overlapping YWHAZ	7.875461
ENSG00000108576	6532	SLC6A4	protein_coding	HGNC Symbol	5	solute carrier family 6 member 4 [Source:HGNC Symbol;Acc:HGNC:11050]	7.895941
ENSG00000182329	1.01E+08	KIAA2012	protein_coding	HGNC Symbol	5	KIAA2012 [Source:HGNC Symbol;Acc:HGNC:51250]	8.042549
ENSG00000181016	286006	LSMEM1	protein_coding	HGNC Symbol	5	leucine rich single-pass membrane protein 1 [Source:HGNC Symbol;Acc:HGNC:22036]	8.068104
ENSG00000270681	NA	AC095055.1	antisense	Clone-based (Ensembl) gene	1	novel transcript, antisense to SH3D19	8.108715
ENSG00000269944	NA	AP001267.4	sense_intronic	Clone-based (Ensembl) gene	1	novel transcript	8.207769
ENSG00000130513	9518	GDF15	protein_coding	HGNC Symbol	5	growth differentiation factor 15 [Source:HGNC Symbol;Acc:HGNC:30142]	8.249536
ENSG00000136535	10716	TBR1	protein_coding	HGNC Symbol	6	T-box, brain 1 [Source:HGNC Symbol;Acc:HGNC:11590]	8.330571
ENSG00000182308	285429	DCAF4L1	protein_coding	HGNC Symbol	1	DDB1 and CUL4 associated factor 4 like 1 [Source:HGNC Symbol;Acc:HGNC:27723]	8.471937
ENSG00000262117	400500	BCAR4	lincRNA	HGNC Symbol	7	breast cancer anti-estrogen resistance 4 [Source:HGNC Symbol;Acc:HGNC:22170]	8.51612
ENSG00000122035	387496	RASL11A	protein_coding	HGNC Symbol	3	RAS like family 11 member A [Source:HGNC Symbol;Acc:HGNC:23802]	8.627163
ENSG00000255384	NA	AP001267.2	lincRNA	Clone-based (Ensembl) gene	1	novel transcript	8.764271
ENSG00000134007	8748	ADAM20	protein_coding	HGNC Symbol	1	ADAM metallopeptidase domain 20 [Source:HGNC Symbol;Acc:HGNC:199]	8.777648
ENSG00000227740	NA	AL513329.1	lincRNA	Clone-based (Ensembl) gene	1	novel transcript	8.853138
ENSG00000095110	120400	NXPE1	protein_coding	HGNC Symbol	6	neurexophilin and PC-esterase domain family member 1 [Source:HGNC Symbol;Acc:HGNC:28	8.864571
ENSG00000230736	339666	AL021937.1	lincRNA	Clone-based (Ensembl) gene	1		8.892874
ENSG00000180245	10692	RRH	protein_coding	HGNC Symbol	1	retinal pigment epithelium-derived rhodopsin homolog [Source:HGNC Symbol;Acc:HGNC:104	9.225233
ENSG00000162772	467	ATF3	protein_coding	HGNC Symbol	11	activating transcription factor 3 [Source:HGNC Symbol;Acc:HGNC:785]	9.365725
ENSG00000164778	2020	EN2	protein_coding	HGNC Symbol	1	engrailed homeobox 2 [Source:HGNC Symbol;Acc:HGNC:3343]	9.382804
ENSG00000143107	163479	FNDC7	protein_coding	HGNC Symbol	2	fibronectin type III domain containing 7 [Source:HGNC Symbol;Acc:HGNC:26668]	9.609081
ENSG00000118523	1490	CTGF	protein_coding	HGNC Symbol	1	connective tissue growth factor [Source:HGNC Symbol;Acc:HGNC:2500]	9.653685
ENSG00000243094	NA	AC079203.1	processed_pseudogene	Clone-based (Ensembl) gene	1	ribosomal protein L32 (RPL32) pseudogene	9.700408
ENSG00000261670	NA	AC012213.2	lincRNA	Clone-based (Ensembl) gene	1	novel transcript	9.796575
ENSG00000213028	NA	AL354983.1	processed_pseudogene	Clone-based (Ensembl) gene	1	novel pseudogene	9.896092
ENSG00000215483	646982	LINC00598	lincRNA	HGNC Symbol	16	long intergenic non-protein coding RNA 598 [Source:HGNC Symbol;Acc:HGNC:42770]	10.15561
ENSG00000215483	400123	LINC00598	lincRNA	HGNC Symbol	16	long intergenic non-protein coding RNA 598 [Source:HGNC Symbol;Acc:HGNC:42770]	10.15561
ENSG00000230948	NA	AP001331.1	processed_pseudogene	Clone-based (Ensembl) gene	1	aurora kinase B (AURKB) pseudogene	10.182298
ENSG00000149201	60494	CCDC81	protein_coding	HGNC Symbol	10	coiled-coil domain containing 81 [Source:HGNC Symbol;Acc:HGNC:26281]	10.184813
ENSG00000162733	4921	DDR2	protein_coding	HGNC Symbol	6	discoidin domain receptor tyrosine kinase 2 [Source:HGNC Symbol;Acc:HGNC:2731]	10.214643

ENSG00000243179	NA	AC110769.1	lincRNA	Clone-based (Ensembl) gene	1	novel transcript	10.481277
ENSG00000175967	NA	FO393415.1	antisense	Clone-based (Ensembl) gene	1		10.826758
ENSG00000267717	NA	SRSF10P1	processed_pseudogene	HGNC Symbol	1	serine and arginine rich splicing factor 10 pseudogene 1 [Source:HGNC Symbol;Acc:HGNC:31[illegible]	11.025507
ENSG00000203799	NA	CCDC162P	transcribed_unitary_pseudogene	HGNC Symbol	15	coiled-coil domain containing 162, pseudogene [Source:HGNC Symbol;Acc:HGNC:21565]	11.050017
ENSG00000240441	NA	AC007622.1	processed_pseudogene	Clone-based (Ensembl) gene	1	ribosomal protein L17 (RPL17) pseudogene	11.390128
ENSG00000188771	349633	PLET1	protein_coding	HGNC Symbol	1	placenta expressed transcript 1 [Source:HGNC Symbol;Acc:HGNC:30053]	11.912986
ENSG00000176601	80122	MAP3K19	protein_coding	HGNC Symbol	14	mitogen-activated protein kinase kinase kinase 19 [Source:HGNC Symbol;Acc:HGNC:26249]	11.948612
ENSG00000176697	627	BDNF	protein_coding	HGNC Symbol	19	brain derived neurotrophic factor [Source:HGNC Symbol;Acc:HGNC:1033]	12.128995
ENSG00000261087	NA	AP003469.4	lincRNA	Clone-based (Ensembl) gene	1	novel transcript	12.183911
ENSG00000164266	6690	SPINK1	protein_coding	HGNC Symbol	3	serine peptidase inhibitor, Kazal type 1 [Source:HGNC Symbol;Acc:HGNC:11244]	12.433846
ENSG00000261019	NA	AC010132.4	lincRNA	Clone-based (Ensembl) gene	1	novel transcript	12.869515
ENSG00000164379	94234	FOXQ1	protein_coding	HGNC Symbol	1	forkhead box Q1 [Source:HGNC Symbol;Acc:HGNC:20951]	12.912885
ENSG00000183423	345193	LRIT3	protein_coding	HGNC Symbol	2	leucine rich repeat, Ig-like and transmembrane domains 3 [Source:HGNC Symbol;Acc:HGNC:2[illegible]	13.054067
ENSG00000164007	149461	CLDN19	protein_coding	HGNC Symbol	3	claudin 19 [Source:HGNC Symbol;Acc:HGNC:2040]	13.300555
ENSG00000139287	121278	TPH2	protein_coding	HGNC Symbol	6	tryptophan hydroxylase 2 [Source:HGNC Symbol;Acc:HGNC:20692]	13.677603
ENSG00000163606	131450	CD200R1	protein_coding	HGNC Symbol	5	CD200 receptor 1 [Source:HGNC Symbol;Acc:HGNC:24235]	14.258882
ENSG00000278313	NA	AC100827.5	unprocessed_pseudogene	Clone-based (Ensembl) gene	1	SWI5 recombination repair homolog (yeast) (SWI5) pseudogene	14.290208
ENSG00000099860	4616	GADD45B	protein_coding	HGNC Symbol	7	growth arrest and DNA damage inducible beta [Source:HGNC Symbol;Acc:HGNC:4096]	14.554306
ENSG00000120211	3641	INSL4	protein_coding	HGNC Symbol	1	insulin like 4 [Source:HGNC Symbol;Acc:HGNC:6087]	14.801993
ENSG00000114124	131890	GRK7	protein_coding	HGNC Symbol	1	G protein-coupled receptor kinase 7 [Source:HGNC Symbol;Acc:HGNC:17031]	15.008699
ENSG00000250609	NA	AC110753.1	processed_pseudogene	Clone-based (Ensembl) gene	1	WD repeat domain 77 (WDR77) pseudogene	15.270216
ENSG00000136872	229	ALDOB	protein_coding	HGNC Symbol	10	aldolase, fructose-bisphosphate B [Source:HGNC Symbol;Acc:HGNC:417]	15.312376
ENSG00000240882	NA	AC063952.2	processed_pseudogene	Clone-based (Ensembl) gene	1	prostate tumor overexpressed 1 (PTOV1) pseudogene	15.340107
ENSG00000134539	3824	KLRD1	protein_coding	HGNC Symbol	11	killer cell lectin like receptor D1 [Source:HGNC Symbol;Acc:HGNC:6378]	15.742343
ENSG00000236796	NA	AL157884.3	unprocessed_pseudogene	Clone-based (Ensembl) gene	1	pseudogene similar to part of membrane guanylyl cyclase	15.86072
ENSG00000234199	440900	LINC01191	lincRNA	HGNC Symbol	1	long intergenic non-protein coding RNA 1191 [Source:HGNC Symbol;Acc:HGNC:49595]	15.92966
ENSG00000164309	202333	CMYA5	protein_coding	HGNC Symbol	3	cardiomyopathy associated 5 [Source:HGNC Symbol;Acc:HGNC:14305]	17.943936
ENSG00000254013	NA	MAP2K1P1	processed_pseudogene	HGNC Symbol	1	mitogen-activated protein kinase kinase 1 pseudogene 1 [Source:HGNC Symbol;Acc:HGNC:68[illegible]	18.706908
ENSG00000260672	NA	AC100827.4	lincRNA	Clone-based (Ensembl) gene	1	novel transcript	19.043397
ENSG00000092607	6913	TBX15	protein_coding	HGNC Symbol	3	T-box 15 [Source:HGNC Symbol;Acc:HGNC:11594]	19.198582
ENSG00000230162	NA	CT45A11P	unprocessed_pseudogene	HGNC Symbol	1	cancer/testis antigen family 45 member A11, pseudogene [Source:HGNC Symbol;Acc:HGNC:5[illegible]	19.202966
ENSG00000135077	84868	HAVCR2	protein_coding	HGNC Symbol	6	hepatitis A virus cellular receptor 2 [Source:HGNC Symbol;Acc:HGNC:18437]	19.416372
ENSG00000254479	NA	SLC25A1P1	processed_pseudogene	HGNC Symbol	1	solute carrier family 25 member 1 pseudogene 1 [Source:HGNC Symbol;Acc:HGNC:43845]	19.922426
ENSG00000148156	10880	ACTL7B	protein_coding	HGNC Symbol	1	actin like 7B [Source:HGNC Symbol;Acc:HGNC:162]	20.068903
ENSG00000197140	203102	ADAM32	protein_coding	HGNC Symbol	11	ADAM metallopeptidase domain 32 [Source:HGNC Symbol;Acc:HGNC:15479]	20.126192
ENSG00000259158	317760	ADAM20P1	transcribed_unprocessed_pseudogene	HGNC Symbol	3	ADAM metallopeptidase domain 20 pseudogene 1 [Source:HGNC Symbol;Acc:HGNC:20102]	20.340078
ENSG00000260144	NA	AC100827.2	unprocessed_pseudogene	Clone-based (Ensembl) gene	1	golgin A6 family, member A (GOLGA6A) pseudogene	20.857329
ENSG00000220563	NA	PKMP3	processed_pseudogene	HGNC Symbol	1	pyruvate kinase M1/2 pseudogene 3 [Source:HGNC Symbol;Acc:HGNC:44245]	23.279782
ENSG00000254213	NA	PRXL2AP2	processed_pseudogene	HGNC Symbol	1	peroxiredoxin like 2A pseudogene 2 [Source:HGNC Symbol;Acc:HGNC:52408]	25.82441
ENSG00000224709	NA	OR11M1P	unitary_pseudogene	HGNC Symbol	1	olfactory receptor family 11 subfamily M member 1 pseudogene [Source:HGNC Symbol;Acc:H[illegible]	28.374193
ENSG00000237550	NA	RPL9P9	transcribed_processed_pseudogene	HGNC Symbol	2	ribosomal protein L9 pseudogene 9 [Source:HGNC Symbol;Acc:HGNC:17251]	28.602635
ENSG00000216990	NA	HSPD1P10	processed_pseudogene	HGNC Symbol	1	heat shock protein family D (Hsp60) member 1 pseudogene 10 [Source:HGNC Symbol;Acc:HG[illegible]	29.53624
ENSG00000204564	221545	C6orf136	protein_coding	HGNC Symbol	13	chromosome 6 open reading frame 136 [Source:HGNC Symbol;Acc:HGNC:21301]	40.8378
ENSG00000182585	255324	EPGN	protein_coding	HGNC Symbol	9	epithelial mitogen [Source:HGNC Symbol;Acc:HGNC:17470]	59.008908
