# Supplementary material for: Splicing factor SF3B1 promotes endometrial cancer progression via regulating KSR2 RNA maturation
Source: Cell Death Dis. 2020 Oct 10;11(10):842. doi: 10.1038/s41419-020-03055-y (PMC7548007; doi:10.1038/s41419-020-03055-y)
Supplement: Supplementary file 7 — Supplementary Table 2 [file 41419_2020_3055_MOESM7_ESM.pdf]

Supplementary Table 2

Biological Process

Category	Term	Count	%	PValue	Genes	List Total	Pop Hits	Pop Total	Fold Enrichment	Bonferroni	Benjamini	FDR
GOTERM_BP_DIRECT	GO:0030522~intracellular receptor signaling pathway	11	0.75445816	0.00014576	ESRRA, NR1D2, ESRRB, SLA2, NR4A2, RORC, NR4A1, NR2E3, NR4A3, AHR, NR1H3	1125	38	16792	4.32074854	0.41123151	0.41123151	0.26772529
GOTERM_BP_DIRECT	GO:0007166~cell surface receptor signaling pathway	36	2.4691358	0.00017468	CD101, GPR182, IL1R1, ADGRD2, ADGRF1, TSPAN2, TNFRSF25, CTF1, C5, EDN1, OXTR, IL12RB2, TSPAN11, TSPAN10, PTK2B, CLCF1, CLEC4A, CD4, ADGRB1, KLRD1, EGFR, KLRB1, GPR19, DTX1, CD160, ADGRG5, NPR1, BIRC3, CRHR1, CASP10, CBLC, CRHR2, P2RX7, PRLR, JMJD6, GIPR	1125	274	16792	1.96110949	0.46998848	0.2719811	0.32077559
GOTERM_BP_DIRECT	GO:0071376~cellular response to corticotropin-releasing hormone stimulus	5	0.34293553	0.00026853	CRHR1, CRHR2, NR4A2, NR4A1, NR4A3	1125	6	16792	12.4385185	0.62317637	0.27770816	0.49270972
GOTERM_BP_DIRECT	GO:0035556~intracellular signal transduction	47	3.22359396	0.00027043	GPR182, NUAK2, TUFT1, NUAK1, PLEKHM1, EDN1, MAP4K2, CSPG4, ASB12, ASB13, LATS2, MCF2L, RASAL1, PLCL1, CORO2A, PLCB3, RGS11, NOD2, RAC3, CTGF, HMOX1, PLCD4, PLCD1, NIM1K, ADCY10, PLCB2, RASA4, DEDD2, BLNK, GPR155, SGK1, DGKQ, MLXIPL, NPR1, MAPK11, WNK2, DVL1, ASB9, MAST3, CDC42BPG, KSR2, DUSP1, RPS6KA2, DEPTOR, JAK3, MAP3K12, NRBP2	1125	403	16792	1.7407753	0.62577264	0.2178611	0.49619135
GOTERM_BP_DIRECT	GO:0001525~angiogenesis	30	2.05761317	0.0004507	NRP2, PTGS2, CSPG4, TNFSF12, MMP2, RNF213, EPHB2, MMRN2, TYMP, HEY1, PTK2B, CTGF, HMOX1, SERPINE1, ZC3H12A, HS6ST1, PLCD1, ANGPT2, KLF5, NOX5, HSPG2, VAV2, VASH1, VEGFC, VEGFD, EPGN, LAMA5, JUN, VHLL, SEMA4A	1125	223	16792	2.00801196	0.80567028	0.27937746	0.82564014
GOTERM_BP_DIRECT	GO:0051966~regulation of synaptic transmission, glutamatergic	8	0.54869684	0.00055948	GRM4, GRM3, GRM2, DKK1, GRIK1, MAPK8IP2, ATP1A2, GRM1	1125	23	16792	5.19172947	0.86915317	0.28748524	1.02396003
GOTERM_BP_DIRECT	GO:0007588~excretion	10	0.68587106	0.00058857	CLDN16, ABCG5, SLC22A18, HMOX1, CLCNKA, CLCNKB, AQP6, ATP6V1B1, AMN, KCNJ1	1125	37	16792	4.03411411	0.882286	0.26335006	1.07692602
GOTERM_BP_DIRECT	GO:0060716~labyrinthine layer blood vessel development	7	0.48010974	0.00112918	FBXW8, HEY1, HS6ST1, PLCD1, JUNB, VASH1, CYR61	1125	19	16792	5.4991345	0.98352222	0.40143358	2.05641468
GOTERM_BP_DIRECT	GO:0006468~protein phosphorylation	49	3.36076818	0.0012287	SGK494, MYOD1, CDK18, NUAK2, PDGFB, NUAK1, FAM20C, FPGT-TNNI3K, AURKC, MAP4K2, PKMYT1, FES, ITPKA, LATS2, TRIB1, CAMKK2, PSKH1, SBK1, PTK2B, PTK6, MLKL, NIM1K, MATK, AATK, ERCC2, OBSCN, SGK1, ALPK1, PHKG1, ALPK2, MYLK3, PNCK, PIM1, NPR1, WNK2, CDC25B, P2RX7, CDC42BPG, KSR2, PLK3, CDK11A, MAPK4, PRKAR1B, JAK3, LRRK1, GRK3, MYLK, MAP3K12, NRBP2	1125	456	16792	1.60391423	0.98852805	0.39129881	2.23573899
GOTERM_BP_DIRECT	GO:0006950~response to stress	12	0.82304527	0.00217661	EGFR, TRAP1, SGK1, CGREF1, PTK2B, C5, GADD45G, DUSP10, MAPK11, GADD45B, ERRFI1, GADD45A	1125	61	16792	2.93630601	0.99963602	0.54699046	3.9281576
GOTERM_BP_DIRECT	GO:0042493~response to drug	34	2.33196159	0.00417789	ALAD, SORD, PTGS2, SLC6A4, OXTR, ABCA2, XRCC1, CBX7, GATA6, PTK2B, PEMT, SEMA3C, HTR1D, FOSL1, SREBF1, DNMT3A, SLC8A1, ACTC1, RPH3AL, ATP1A3, FOSB, ACACB, JUNB, HDAC4, VEGFC, P2RX7, CDKN1A, ABCG5, JUN, TBXA2R, HSD11B2, ABAT, LRP8, HTR2B	1125	304	16792	1.66938012	0.99999975	0.74920332	7.41073122
GOTERM_BP_DIRECT	GO:0043401~steroid hormone mediated signaling pathway	11	0.75445816	0.00418943	ESRRA, NR1D2, ESRRB, ESRRG, NR4A2, RORC, NR4A1, PAQR8, NR2E3, NR4A3, NR1H3	1125	57	16792	2.88049903	0.99999976	0.71955091	7.43046422
GOTERM_BP_DIRECT	GO:0000185~activation of MAPKKK activity	5	0.34293553	0.00450265	GADD45G, MAP4K2, GADD45B, TAB1, GADD45A	1125	11	16792	6.78464646	0.99999992	0.71677274	7.96449234
GOTERM_BP_DIRECT	GO:0007507~heart development	23	1.57750343	0.00548103	NRP2, SH3PXD2B, CRIP1, PDGFB, TAZ, EDN1, OXTR, CAD, TNNI3, DVL1, PSKH1, ADM, JMJD6, RPS6KA2, TSC2, GYS1, PKD1, FOXC1, NFATC4, STRA6, RIPPLY3, HTR2B, IFT140	1125	183	16792	1.87597328	1	0.75988361	9.61388195
GOTERM_BP_DIRECT	GO:0036066~protein O-linked fucosylation	5	0.34293553	0.00639952	ADAMTS7, CFP, ADAMTSL4, POFUT2, ADAMTS5	1125	12	16792	6.21925926	1	0.78888952	11.1368484
GOTERM_BP_DIRECT	GO:0008284~positive regulation of cell proliferation	46	3.15500686	0.00846461	FGFR3, PDGFB, CTF1, EDN1, ZNF16, IL11, GLI1, IL12RB2, OSR2, RAC2, CLCF1, CTGF, PTK2B, HSF4, FOSL1, RUNX2, SERTAD1, MATK, KLF5, EGFR, IRS2, WNT10B, KLB, CRIP2, FLT4, EFNB2, MLXIPL, RPS9, ROGDI, IRS1, CDC25B, CAPN1, PTHLH, UFL1, HDAC4, VEGFC, VEGFD, CNTF, ATF3, ADM, EPGN, CCND2, ETS1, HBEGF, BAMBI, HTR2B	1125	466	16792	1.47340391	1	0.85495447	14.4728441
GOTERM_BP_DIRECT	GO:0015677~copper ion import	4	0.27434842	0.00853513	ATP7A, STEAP3, STEAP2, ATP7B	1125	7	16792	8.52926984	1	0.83996138	14.5846431
GOTERM_BP_DIRECT	GO:0050727~regulation of inflammatory response	11	0.75445816	0.00863667	IL1R1, NOD2, PTGS2, FANCD2, NR1D2, PYCARD, BCL6, PIK3AP1, GGT1, BIRC3, NLRP1	1125	63	16792	2.60616578	1	0.82643827	14.7453896
GOTERM_BP_DIRECT	GO:0006629~lipid metabolic process	20	1.37174211	0.00878253	SREBF1, WNT10B, LCN12, FADS1, FADS3, BTN2A1, ABHD4, ABCA2, ALDH3B1, APOL2, PNPLA7, PLCL1, PTGDS, PLIN1, PEMT, LRP8, RDH16, LIPC, THRSP, HMGCL	1125	157	16792	1.90142958	1	0.81496204	14.9757781
GOTERM_BP_DIRECT	GO:0010043~response to zinc ion	8	0.54869684	0.00889761	ATP7A, KHK, P2RX7, ALAD, D2HGDH, CRIP1, HAAO, SLC30A2	1125	36	16792	3.31693827	1	0.80287784	15.1571434
GOTERM_BP_DIRECT	GO:0032259~methylation	12	0.82304527	0.00892811	FAM86B1, FAM86C1, DPH5, MAT1A, FAM86C2P, MTR, PEMT, GAMT, METTL7A, METTL7B, FAM86B2, COMTD1	1125	73	16792	2.45362557	1	0.78816037	15.2051378
GOTERM_BP_DIRECT	GO:0006644~phospholipid metabolic process	10	0.68587106	0.00906937	JMJD7-PLA2G4B, GATA6, ACP6, TAZ, PEMT, ABHD4, PLA2G4F, PLCD1, PLA2G3, PLCB2	1125	54	16792	2.76411523	1	0.77796704	15.427141
GOTERM_BP_DIRECT	GO:0046330~positive regulation of JNK cascade	11	0.75445816	0.01072789	DUSP19, NOD2, CTGF, PTK2B, FLT4, GADD45G, MAP4K2, PYCARD, MAP3K10, GADD45B, GADD45A	1125	65	16792	2.52597607	1	0.81807571	17.9928609
GOTERM_BP_DIRECT	GO:0030324~lung development	12	0.82304527	0.01198463	SREBF1, EGFR, SLC23A1, CCDC40, CRISPLD2, LAMA5, CTGF, JMJD6, STRA6, ZFPM2, TAB1, GLI1	1125	76	16792	2.35677193	1	0.83888453	19.887805
GOTERM_BP_DIRECT	GO:0060346~bone trabecula formation	4	0.27434842	0.01298199	MSX2, WNT10B, MMP2, THBS3	1125	8	16792	7.46311111	1	0.85034434	21.3621163
GOTERM_BP_DIRECT	GO:0040036~regulation of fibroblast growth factor receptor signaling pathway	4	0.27434842	0.01298199	LRIT3, FAM20C, HHIP, RUNX2	1125	8	16792	7.46311111	1	0.85034434	21.3621163
GOTERM_BP_DIRECT	GO:0055088~lipid homeostasis	8	0.54869684	0.01377777	IRS2, ACADSB, NR1D2, IVD, ACADS, PNPLA3, PNPLA5, NR1H3	1125	39	16792	3.06178917	1	0.85616632	22.5200025
GOTERM_BP_DIRECT	GO:0009749~response to glucose	11	0.75445816	0.01455701	KHK, SREBF1, SLC8A1, IRS2, HNF1A, GJD3, CTGF, PTK2B, GIPR, SLC37A4, ANGPT2	1125	68	16792	2.41453595	1	0.86105343	23.6381797
GOTERM_BP_DIRECT	GO:0009612~response to mechanical stimulus	10	0.68587106	0.01592376	INHBB, P2RX7, ETS1, PTK2B, JUN, ENDOG, FOSB, ANGPT2, FOSL1, JUNB	1125	59	16792	2.52986817	1	0.87548233	25.5626555
GOTERM_BP_DIRECT	GO:0043433~negative regulation of sequence-specific DNA binding transcription factor activity	10	0.68587106	0.0176568	HDAC4, SMAD7, GLIS2, HMOX1, PIM1, MAP3K10, DDIT3, TRIB1, FOXS1, SIGIRR	1125	60	16792	2.4877037	1	0.89272354	27.936988
GOTERM_BP_DIRECT	GO:0048661~positive regulation of smooth muscle cell proliferation	10	0.68587106	0.0176568	NOTCH3, EGFR, HDAC4, PDGFB, PTGS2, JUN, HMOX1, EDN1, HBEGF, NR4A3	1125	60	16792	2.4877037	1	0.89272354	27.936988
GOTERM_BP_DIRECT	GO:0050731~positive regulation of peptidyl-tyrosine phosphorylation	12	0.82304527	0.02041035	VEGFC, CD44, PDGFB, PTK2B, CSPG4, KITLG, CD4, LRP8, VTN, LRRK1, LRP4, IL11	1125	82	16792	2.1843252	1	0.91774684	31.5627421
GOTERM_BP_DIRECT	GO:0006357~regulation of transcription from RNA polymerase II promoter	42	2.88065844	0.0206683	MYOD1, FOSL2, HNF1A, ELF3, ELF5, ABCA2, ZEB1, BUD31, FOXS1, KCNIP3, STAT6, FOXQ1, TRAK2, CHD2, HSF4, TCEA2, FOSL1, RUNX2, FOXD4, ETV3, SREBF1, TCF7, DGKQ, RFX5, MAFB, ZMYM3, GMEB1, SOX12, MLXIPL, SAP18, FOSB, ZNF662, JUNB, AHR, TARBP1, MED6, FOXD4L4, ATF3, FOXD4L1, ZBED6, TBX19, FOXE3	1125	441	16792	1.42154497	1	0.913555	31.8934152
GOTERM_BP_DIRECT	GO:0051090~regulation of sequence-specific DNA binding transcription factor activity	6	0.41152263	0.02302212	SGK1, CREBZF, FANCD2, JUN, HMOX1, MAPK11	1125	25	16792	3.58229333	1	0.92899568	34.8418263
GOTERM_BP_DIRECT	GO:0070542~response to fatty acid	5	0.34293553	0.02355018	ALAD, PTGS2, CTGF, GIPR, HMGCL	1125	17	16792	4.39006536	1	0.92751671	35.4864904
GOTERM_BP_DIRECT	GO:0046498~S-adenosylhomocysteine metabolic process	3	0.20576132	0.02452738	DNMT3A, PEMT, GAMT	1125	4	16792	11.1946667	1	0.92964694	36.6636004
GOTERM_BP_DIRECT	GO:0019344~cysteine biosynthetic process	3	0.20576132	0.02452738	CBSL, GGT1, CBS	1125	4	16792	11.1946667	1	0.92964694	36.6636004
GOTERM_BP_DIRECT	GO:0034154~toll-like receptor 7 signaling pathway	3	0.20576132	0.02452738	HAVCR2, UNC93B1, PIK3AP1	1125	4	16792	11.1946667	1	0.92964694	36.6636004
GOTERM_BP_DIRECT	GO:0006000~fructose metabolic process	4	0.27434842	0.025154	KHK, PFKFB4, ALDOB, FBP1	1125	10	16792	5.97048889	1	0.92900266	37.4076992
GOTERM_BP_DIRECT	GO:0016042~lipid catabolic process	12	0.82304527	0.02597266	PLD2, PLCL1, PLCB3, PLIN1, ASPG, ABHD4, PLCD4, PLCD1, PLA2G3, PNPLA3, PLCB2, PNPLA5	1125	85	16792	2.10723137	1	0.92980263	38.3673805
GOTERM_BP_DIRECT	GO:0006970~response to osmotic stress	5	0.34293553	0.02871476	EGFR, PLK3, SORD, LRRC8A, PTK2B	1125	18	16792	4.14617284	1	0.94281927	41.4814792
GOTERM_BP_DIRECT	GO:0001556~oocyte maturation	5	0.34293553	0.02871476	RPS6KA2, PTK2B, TUBB8, CDC25B, TRIP13	1125	18	16792	4.14617284	1	0.94281927	41.4814792
GOTERM_BP_DIRECT	GO:0060048~cardiac muscle contraction	8	0.54869684	0.02877362	SLC8A1, ACTC1, TAZ, ATP1A3, MYLK2, ATP1A2, TNNI3, TNNI1	1125	45	16792	2.65355062	1	0.9387034	41.5466725
GOTERM_BP_DIRECT	GO:0042475~odontogenesis of dentin-containing tooth	9	0.61728395	0.02902079	DLX3, ADM, LAMA5, JAG2, ROGDI, FOXC1, RUNX2, BCL2L11, LRP4	1125	55	16792	2.44247273	1	0.93569748	41.819647

GOTERM_BP_DIRECT	GO:0014065~phosphatidylinositol 3-kinase signaling	6	0.41152263	0.03134145	NYAP2, EDN1, HTR2B, NYAP1, IRS1, SIRT2	1125	27	16792	3.31693827	1	0.94458726	44.324512
GOTERM_BP_DIRECT	GO:1903779~regulation of cardiac conduction	9	0.61728395	0.03194574	SLC8A1, ATP2A3, FPGT-TNNI3K, ATP1A3, NPR1, AHCYL1, ATP1A2, TNNI3, EHD3	1125	56	16792	2.39885714	1	0.94373584	44.9598385
GOTERM_BP_DIRECT	GO:0035970~peptidyl-threonine dephosphorylation	4	0.27434842	0.03289826	PPM1F, DUSP5, DUSP4, DUSP1	1125	11	16792	5.42771717	1	0.94466545	45.9473644
GOTERM_BP_DIRECT	GO:0045820~negative regulation of glycolytic process	4	0.27434842	0.03289826	HDAC4, FBP1, CBFA2T3, DDIT4	1125	11	16792	5.42771717	1	0.94466545	45.9473644
GOTERM_BP_DIRECT	GO:0007175~negative regulation of epidermal growth factor-activated receptor activity	4	0.27434842	0.03289826	CBLC, ZFYVE28, ERRFI1, GPRC5A	1125	11	16792	5.42771717	1	0.94466545	45.9473644
GOTERM_BP_DIRECT	GO:0051968~positive regulation of synaptic transmission, glutamatergic	5	0.34293553	0.03449845	EGFR, PTGS2, PTK2B, OXTR, SHANK2	1125	19	16792	3.92795322	1	0.94854243	47.5687497
GOTERM_BP_DIRECT	GO:0006883~cellular sodium ion homeostasis	5	0.34293553	0.03449845	SGK1, SLC8A1, ATP1A3, ATP1A2, TMPRSS3	1125	19	16792	3.92795322	1	0.94854243	47.5687497
GOTERM_BP_DIRECT	GO:0006805~xenobiotic metabolic process	11	0.75445816	0.03472669	GSTA4, SULT1A1, AKR7A2, EPHX2, AKR7A3, RORC, GGT1, BPHL, GSTO2, AHR, AOC3	1125	78	16792	2.10498006	1	0.94601735	47.7962341
GOTERM_BP_DIRECT	GO:0007565~female pregnancy	12	0.82304527	0.03499218	CRHR1, PTHLH, OVGP1, INSL4, ADM, ETS1, ENDOU, HSD11B2, OXTR, CAD, FOSB, FOSL1	1125	89	16792	2.01252434	1	0.94366495	48.0596703
GOTERM_BP_DIRECT	GO:0007155~cell adhesion	42	2.88065844	0.03617323	NRP2, TLN2, NUAK1, PCDHB15, ITGB4, FERMT1, KITLG, VTN, L1CAM, DDR2, HMCN2, GP5, CGREF1, CD44, COL6A6, SORBS2, CTGF, CLEC4A, AATF, CD4, ADGRB1, KIRREL2, SELPLG, THBS3, RS1, CYR61, FLRT1, CNTN5, EGFL6, LRRN2, EFNB2, PCDH10, PCDH17, SSPO, NCAM1, RND3, PKP1, HAS1, FBLN7, THEMIS2, MUC16, AOC3	1125	459	16792	1.36579811	1	0.94555978	49.2164029
GOTERM_BP_DIRECT	GO:0042517~positive regulation of tyrosine phosphorylation of Stat3 protein	7	0.48010974	0.03894269	FGFR3, CNTF, SOCS3, CLCF1, CTF1, PTK6, STAP2	1125	38	16792	2.74956725	1	0.9536351	51.8340573
GOTERM_BP_DIRECT	GO:0007154~cell communication	7	0.48010974	0.03894269	SLC8A1, GJD3, GJB7, KREMEN2, OIP5, GJB6, GJC1	1125	38	16792	2.74956725	1	0.9536351	51.8340573
GOTERM_BP_DIRECT	GO:0048668~collateral sprouting	3	0.20576132	0.03907895	BDNF, DVL1, HDAC6	1125	5	16792	8.95573333	1	0.95109931	51.959497
GOTERM_BP_DIRECT	GO:0010756~positive regulation of plasminogen activation	3	0.20576132	0.03907895	F12, HPN, PLGRKT	1125	5	16792	8.95573333	1	0.95109931	51.959497
GOTERM_BP_DIRECT	GO:0010656~negative regulation of muscle cell apoptotic process	3	0.20576132	0.03907895	PTK2B, HMOX1, ZC3H12A	1125	5	16792	8.95573333	1	0.95109931	51.959497
GOTERM_BP_DIRECT	GO:2001236~regulation of extrinsic apoptotic signaling pathway	3	0.20576132	0.03907895	TRAF1, DEPTOR, ACSL5	1125	5	16792	8.95573333	1	0.95109931	51.959497
GOTERM_BP_DIRECT	GO:0061624~fructose catabolic process to hydroxyacetone phosphate and glyceraldehyde-3-phosphate	3	0.20576132	0.03907895	KHK, TKFC, ALDOB	1125	5	16792	8.95573333	1	0.95109931	51.959497
GOTERM_BP_DIRECT	GO:0048812~neuron projection morphogenesis	8	0.54869684	0.03932359	ATP7A, EGFR, SGK1, PACSIN1, NYAP2, ZSWIM6, DYNLT1, NYAP1	1125	48	16792	2.4877037	1	0.94896563	52.183931
GOTERM_BP_DIRECT	GO:0001938~positive regulation of endothelial cell proliferation	10	0.68587106	0.03984258	NRP2, VEGFC, VEGFD, PDGFB, JUN, FLT4, PLXNB3, NR4A1, TNFSF12, HTR2B	1125	69	16792	2.16322061	1	0.94792267	52.6567816
GOTERM_BP_DIRECT	GO:0035690~cellular response to drug	10	0.68587106	0.03984258	PPM1F, RBM22, EGFR, ADSSL1, RECQL5, PDE4B, EDN1, FBP1, CAD, ANKRD1	1125	69	16792	2.16322061	1	0.94792267	52.6567816
GOTERM_BP_DIRECT	GO:0042127~regulation of cell proliferation	20	1.37174211	0.04144883	ESRRA, SGK1, TCF7, NUAK1, TNFRSF25, FA2H, JAG2, FES, RBBP9, JUNB, STAT6, LAMA5, PTK2B, JUN, PTK6, BCL6, PLCD1, CHRNA10, TFAP2E, TBX19	1125	185	16792	1.61364565	1	0.95102211	54.0923741
GOTERM_BP_DIRECT	GO:0001706~endoderm formation	4	0.27434842	0.04173117	DUSP5, DUSP4, DKK1, DUSP1	1125	12	16792	4.97540741	1	0.94915463	54.3404229
GOTERM_BP_DIRECT	GO:0034220~ion transmembrane transport	22	1.50891632	0.04262432	SGK1, GJD3, GRIK1, GLRA1, ANO1, CLCNKA, ATP1A3, SFXN3, CLCNKB, ATP1A2, ASIC1, ATP6V1B1, GJC1, ATP7A, ATP6V0C, ATP2A3, SCNN1A, SCNN1D, ATP6V0A2, ANO9, ATP7B, ANO8	1125	210	16792	1.56369947	1	0.94954612	55.1167749
GOTERM_BP_DIRECT	GO:0007254~JNK cascade	8	0.54869684	0.04333448	NOD2, DUSP10, MAP4K2, MAPK8IP2, MAP3K10, TAB1, MAP3K12, TRIB1	1125	49	16792	2.43693424	1	0.94927438	55.7251497
GOTERM_BP_DIRECT	GO:0035914~skeletal muscle cell differentiation	8	0.54869684	0.04333448	KLF5, MYOD1, MEF2D, ATF3, MYLK2, NR4A1, HMG20B, ANKRD1	1125	49	16792	2.43693424	1	0.94927438	55.7251497
GOTERM_BP_DIRECT	GO:0045766~positive regulation of angiogenesis	14	0.96021948	0.04375951	C5, TNFSF12, VEGFC, VEGFD, ADM, PTK2B, GATA6, ETS1, HMOX1, SERPINE1, TBXA2R, ZC3H12A, ANGPT2, TERT	1125	115	16792	1.81710531	1	0.94799835	56.0855182
GOTERM_BP_DIRECT	GO:0043065~positive regulation of apoptotic process	29	1.98902606	0.04692576	PNMA2, PTGS2, ADAMTSL4, ANKRD1, MCF2L, LATS2, HMOX1, PYCARD, BCL6, ADCY10, BMF, PHLDA3, FOSL1, TOP2A, OBSCN, WNT10B, HRK, SAP18, NR4A1, FRZB, VAV2, BCL2L11, ADM, DUSP1, RPS6KA2, GADD45G, MAP3K10, GADD45B, GADD45A	1125	300	16792	1.44286815	1	0.95579474	58.6840951
GOTERM_BP_DIRECT	GO:0060325~face morphogenesis	6	0.41152263	0.04696076	DKK1, CRISPLD2, MYH3, CSRNP1, STRA6, MMP2	1125	30	16792	2.98524444	1	0.95341773	58.7119937
GOTERM_BP_DIRECT	GO:0055117~regulation of cardiac muscle contraction	5	0.34293553	0.04794503	ANK2, SMAD7, FPGT-TNNI3K, RNF207, EHD3	1125	21	16792	3.55386243	1	0.95396739	59.4892009
GOTERM_BP_DIRECT	GO:0006367~transcription initiation from RNA polymerase II promoter	17	1.1659808	0.04806569	ESRRA, ESRRB, ESRRG, NR4A2, GTF2H3, TEAD1, NR4A1, RORC, NR4A3, NR2E3, MED10, NOTCH3, MED6, NR1D2, CTGF, NR1H3, ERCC2	1125	152	16792	1.66938012	1	0.95187895	59.5835217
GOTERM_BP_DIRECT	GO:0048468~cell development	7	0.48010974	0.04841619	INHBB, GATA6, GDF10, HSF4, GDF15, FOXE3, GJC1	1125	40	16792	2.61208889	1	0.95049954	59.8563329
GOTERM_BP_DIRECT	GO:0061029~eyelid development in camera-type eye	4	0.27434842	0.05162256	EGFR, OSR2, JUN, STRA6	1125	13	16792	4.59268376	1	0.9574711	62.2724311
GOTERM_BP_DIRECT	GO:0071277~cellular response to calcium ion	8	0.54869684	0.05212401	ALOX15, JUN, ENDOG, EDN1, RASGRP2, FOSB, RASA4, JUNB	1125	51	16792	2.34136819	1	0.95661505	62.6376246
GOTERM_BP_DIRECT	GO:0043552~positive regulation of phosphatidylinositol 3-kinase activity	6	0.41152263	0.05302799	NOD2, FGFR3, PDGFB, PTK2B, VAV2, IRS1	1125	31	16792	2.88894624	1	0.9568415	63.2875284
GOTERM_BP_DIRECT	GO:0043547~positive regulation of GTPase activity	49	3.36076818	0.05320742	ARHGDIG, NRTN, FGFR3, PDGFB, RAP1GAP, ARHGEF25, RGL3, KITLG, CYTH2, DENND2A, ASAP3, RASGEF1C, GDPGP1, MCF2L, RASAL1, WNT4, RGS11, RINL, GRIN2C, STARD8, RASGRP2, ERRFI1, DOCK11, RASA4, DENND6B, EGFR, OBSCN, ECT2L, IRS2, ARHGEF1, KLB, VAV2, IRS1, RIC8A, NCAM1, ARHGAP31, ARHGAP33, ACAP3, JUN, TBCD, TSC2, SPTBN2, TBXA2R, HBEGF, JAK3, HTR2B, EPS8L2, RAPGEFL1, SPTAN1	1125	565	16792	1.29448653	1	0.95515433	63.4152508
GOTERM_BP_DIRECT	GO:0050896~response to stimulus	9	0.61728395	0.05373768	HMCN2, LRIT3, CLDN19, PRCD, CYP4V2, SCNN1A, SCNN1D, CDH23, RD3	1125	62	16792	2.16670968	1	0.95441092	63.7902513
GOTERM_BP_DIRECT	GO:0045453~bone resorption	5	0.34293553	0.05560666	CTSK, HNF1A, RAC2, PTK2B, LRRK1	1125	22	16792	3.39232323	1	0.95715457	65.0832071
GOTERM_BP_DIRECT	GO:0001957~intramembranous ossification	3	0.20576132	0.05605269	CTSK, MN1, MMP2	1125	6	16792	7.46311111	1	0.95622804	65.3852518
GOTERM_BP_DIRECT	GO:0060137~maternal process involved in parturition	3	0.20576132	0.05605269	CD55, EDN1, OXTR	1125	6	16792	7.46311111	1	0.95622804	65.3852518
GOTERM_BP_DIRECT	GO:0003416~endochondral bone growth	3	0.20576132	0.05605269	MSX2, FGFR3, DDR2	1125	6	16792	7.46311111	1	0.95622804	65.3852518
GOTERM_BP_DIRECT	GO:0038084~vascular endothelial growth factor signaling pathway	3	0.20576132	0.05605269	NRP2, FLT4, FOXC1	1125	6	16792	7.46311111	1	0.95622804	65.3852518
GOTERM_BP_DIRECT	GO:0030031~cell projection assembly	3	0.20576132	0.05605269	RAC2, RAC3, NEFH	1125	6	16792	7.46311111	1	0.95622804	65.3852518
GOTERM_BP_DIRECT	GO:0009636~response to toxic substance	11	0.75445816	0.05711932	DNMT3A, DHRS2, CDKN1A, SLC23A1, SLC6A4, EPHX2, BPHL, HTR1D, PDZD3, AHR, HDAC6	1125	85	16792	1.93162876	1	0.95685425	66.0975673
GOTERM_BP_DIRECT	GO:0015991~ATP hydrolysis coupled proton transport	6	0.41152263	0.05952899	ATP6V0C, ATP1A3, ATP1A2, ATP6AP1L, ATP6V1B1, ATP6V0A2	1125	32	16792	2.79866667	1	0.96053685	67.6561025
GOTERM_BP_DIRECT	GO:0001516~prostaglandin biosynthetic process	4	0.27434842	0.06253139	PTGDS, PTGS2, EDN1, PLA2G4F	1125	14	16792	4.26463492	1	0.96499307	69.5032861
GOTERM_BP_DIRECT	GO:0033137~negative regulation of peptidyl-serine phosphorylation	5	0.34293553	0.06388554	PPM1F, DKK1, SMAD7, MLXIPL, DDIT4	1125	23	16792	3.24483092	1	0.96591785	70.3033548
GOTERM_BP_DIRECT	GO:0030155~regulation of cell adhesion	7	0.48010974	0.06507539	PRLR, LAMA5, NUAK1, PTK2B, JAG2, PKD1, FES	1125	43	16792	2.42985013	1	0.96650117	70.9899256
GOTERM_BP_DIRECT	GO:0045747~positive regulation of Notch signaling pathway	6	0.41152263	0.06646265	NOD2, TSPAN10, JAG2, RFNG, LFNG, POGLUT1	1125	33	16792	2.71385859	1	0.96740648	71.7714536
GOTERM_BP_DIRECT	GO:0022617~extracellular matrix disassembly	10	0.68587106	0.06657952	SH3PXD2B, CTSK, CD44, EXOC8, ELN, HSPG2, MMP2, ADAMTS5, TLL1, CAPN1	1125	76	16792	1.96397661	1	0.96607232	71.8363721
GOTERM_BP_DIRECT	GO:0030198~extracellular matrix organization	20	1.37174211	0.06658371	ELF3, PDGFB, EGFL6, ADAMTSL4, ELN, ITGB4, HSPG2, VTN, DDR2, ATP7A, SMOC2, COL14A1, CD44, CRISPLD2, LAMA5, SERPINE1, FOXF2, VHLL, CYR61, ERCC2	1125	196	16792	1.5230839	1	0.9645144	71.8387
GOTERM_BP_DIRECT	GO:0006469~negative regulation of protein kinase activity	12	0.82304527	0.06664246	PPM1F, FLRT1, DUSP19, RTN4RL1, LRRTM4, SOCS3, TSC2, DEPTOR, GADD45B, GADD45A, TRIB1, DVL1	1125	99	16792	1.80923906	1	0.96303222	71.8712805
GOTERM_BP_DIRECT	GO:0001666~response to hypoxia	18	1.2345679	0.06895132	ALAD, SLC6A4, NR4A2, XRCC1, CBFA2T3, MMP2, DDIT4, VEGFC, VEGFD, ADM, ETS1, PTK2B, HMOX1, HSD11B2, ABAT, VHLL, ANGPT2, ERCC2	1125	172	16792	1.56204651	1	0.96567125	73.1238186

GOTERM_BP_DIRECT	GO:0000165~MAPK cascade	25	1.71467764	0.07351543	NRTN, FGFR3, PDGFB, KITLG, CAMKK2, RASAL1, PTK2B, GRIN2C, RASA4, EGFR, IRS2, KLB, MAPK11, IRS1, NCAM1, DUSP5, PSMA2, MEF2D, EPGN, MAPK4, SPTBN2, MAPK8IP2, HBEGF, JAK3, SPTAN1	1125	262	16792	1.42425785	1	0.97148983	75.4462822
GOTERM_BP_DIRECT	GO:0050919~negative chemotaxis	6	0.41152263	0.07382587	NRP2, PLXNA3, SEMA6B, SEMA3C, SLIT1, SEMA4A	1125	34	16792	2.63403922	1	0.97063285	75.5971496
GOTERM_BP_DIRECT	GO:0030282~bone mineralization	6	0.41152263	0.07382587	ALOX15, SBDS, FGFR3, PTGS2, TUFT1, ERCC2	1125	34	16792	2.63403922	1	0.97063285	75.5971496
GOTERM_BP_DIRECT	GO:1904707~positive regulation of vascular smooth muscle cell proliferation	4	0.27434842	0.07440802	PDGFB, DDX39B, MMP2, TERT	1125	15	16792	3.98032593	1	0.970173	75.8777045
GOTERM_BP_DIRECT	GO:0006646~phosphatidylethanolamine biosynthetic process	4	0.27434842	0.07440802	ETNPPL, ALOX15, PCYT2, LPIN3	1125	15	16792	3.98032593	1	0.970173	75.8777045
GOTERM_BP_DIRECT	GO:0007196~adenylate cyclase-inhibiting G-protein coupled glutamate receptor signaling pathway	3	0.20576132	0.07505972	GRM4, GRM3, GRM2	1125	7	16792	6.39695238	1	0.96982008	76.1881587
GOTERM_BP_DIRECT	GO:0010870~positive regulation of receptor biosynthetic process	3	0.20576132	0.07505972	EDN1, NR1H3, HDAC6	1125	7	16792	6.39695238	1	0.96982008	76.1881587
GOTERM_BP_DIRECT	GO:0034983~peptidyl-lysine deacetylation	3	0.20576132	0.07505972	HDAC4, SIRT2, HDAC6	1125	7	16792	6.39695238	1	0.96982008	76.1881587
GOTERM_BP_DIRECT	GO:0010882~regulation of cardiac muscle contraction by calcium ion signaling	3	0.20576132	0.07505972	HDAC4, SLC8A1, ANK2	1125	7	16792	6.39695238	1	0.96982008	76.1881587
GOTERM_BP_DIRECT	GO:0001755~neural crest cell migration	7	0.48010974	0.07782799	SEMA6B, NRTN, LAMA5, KITLG, SEMA3C, HTR2B, SEMA4A	1125	45	16792	2.32185679	1	0.97242166	77.4652774
GOTERM_BP_DIRECT	GO:0072593~reactive oxygen species metabolic process	6	0.41152263	0.08161361	P2RX7, PDGFB, CTGF, EPHX2, DDIT4, CYR61	1125	35	16792	2.55878095	1	0.97594874	79.1072088
GOTERM_BP_DIRECT	GO:0042594~response to starvation	6	0.41152263	0.08161361	SSTR2, ADSSL1, ADM, CAD, HMGCL, DDIT3	1125	35	16792	2.55878095	1	0.97594874	79.1072088
GOTERM_BP_DIRECT	GO:0030501~positive regulation of bone mineralization	6	0.41152263	0.08161361	P2RX7, SLC8A1, WNT4, WNT10B, OSR2, FAM20C	1125	35	16792	2.55878095	1	0.97594874	79.1072088
GOTERM_BP_DIRECT	GO:0000188~inactivation of MAPK activity	5	0.34293553	0.08224623	DUSP5, DUSP4, DUSP1, DUSP10, DUSP8	1125	25	16792	2.98524444	1	0.97559586	79.3703073
GOTERM_BP_DIRECT	GO:0051591~response to cAMP	7	0.48010974	0.08469492	SREBF1, DUSP1, PTK2B, JUN, FOSB, FOSL1, JUNB	1125	46	16792	2.27138164	1	0.97725804	80.3594602
GOTERM_BP_DIRECT	GO:0007612~learning	8	0.54869684	0.08479874	ARC, AAAS, PTGS2, JUN, STRA6, FOSL1, SHANK2, EPHB2	1125	57	16792	2.09490838	1	0.97634881	80.4003882
GOTERM_BP_DIRECT	GO:1900745~positive regulation of p38MAPK cascade	4	0.27434842	0.08719616	GADD45G, ZC3H12A, GADD45B, GADD45A	1125	16	16792	3.73155556	1	0.977871	81.323429
GOTERM_BP_DIRECT	GO:0006750~glutathione biosynthetic process	4	0.27434842	0.08719616	HAGH, GGT6, OPLAH, GGT1	1125	16	16792	3.73155556	1	0.977871	81.323429
GOTERM_BP_DIRECT	GO:0007340~acrosome reaction	4	0.27434842	0.08719616	GLRA1, SYT8, PLCD4, PCSK4	1125	16	16792	3.73155556	1	0.977871	81.323429
GOTERM_BP_DIRECT	GO:0035994~response to muscle stretch	4	0.27434842	0.08719616	SLC8A1, JUN, EDN1, ANKRD1	1125	16	16792	3.73155556	1	0.977871	81.323429
GOTERM_BP_DIRECT	GO:0051592~response to calcium ion	8	0.54869684	0.09116923	EGFR, P2RX7, D2HGDH, DUSP1, PTK2B, GIPR, AHCYL1, TPH2	1125	58	16792	2.05878927	1	0.98070088	82.76321
GOTERM_BP_DIRECT	GO:0070373~negative regulation of ERK1 and ERK2 cascade	8	0.54869684	0.09116923	DUSP4, ATF3, TBC1D10C, DUSP1, WNK2, ERRFI1, CRYBA1, KLF4	1125	58	16792	2.05878927	1	0.98070088	82.76321
GOTERM_BP_DIRECT	GO:0006814~sodium ion transport	10	0.68587106	0.09127663	SGK1, SLC23A1, SLC5A4, SLC4A11, SLC38A8, SLC13A3, ASIC1, ATP1A2, SCNN1D, SLC10A1	1125	81	16792	1.84274348	1	0.9799227	82.8006348
GOTERM_BP_DIRECT	GO:0007275~multicellular organism development	44	3.01783265	0.09154437	PLXNA3, INSL4, ELF3, TNFRSF25, ANO1, PAQR8, RADIL, FES, DPY19L2, AMN, CCHCR1, WNT4, LBH, FAT3, SPRED3, MN1, TWIST2, TDRD1, UPK2, RS1, OBSCN, WNT10B, EGFL6, ZMYM3, ENC1, DDX1, PIM1, BTBD7, ACKR2, TLE1, EN2, FRZB, MXD1, GGN, WNT2B, APOL2, ADAMTS9, EBF4, PKP1, MEOX1, DLX4, GADD45G, HIVEP2, GADD45B	1125	521	16792	1.26056387	1	0.97927966	82.8935912
GOTERM_BP_DIRECT	GO:0009267~cellular response to starvation	7	0.48010974	0.09188493	INHBB, SREBF1, MYOD1, WNT4, FADS1, PIK3C2B, WNT2B	1125	47	16792	2.22305437	1	0.97869923	83.0111474
GOTERM_BP_DIRECT	GO:0045860~positive regulation of protein kinase activity	7	0.48010974	0.09188493	CDKN1A, DUSP19, PTK2B, CD4, DDR2, CDC25B, CYR61	1125	47	16792	2.22305437	1	0.97869923	83.0111474
GOTERM_BP_DIRECT	GO:0008652~cellular amino acid biosynthetic process	5	0.34293553	0.09229497	CBSL, NAALAD2, FOLH1, ASPG, CBS	1125	26	16792	2.87042735	1	0.97818188	83.1516711
GOTERM_BP_DIRECT	GO:0040016~embryonic cleavage	3	0.20576132	0.09575229	AATF, TOP2A, ERCC2	1125	8	16792	5.59733333	1	0.9804152	84.2935963
GOTERM_BP_DIRECT	GO:0061469~regulation of type B pancreatic cell proliferation	3	0.20576132	0.09575229	NR4A1, NR4A3, ERRFI1	1125	8	16792	5.59733333	1	0.9804152	84.2935963
GOTERM_BP_DIRECT	GO:0034138~toll-like receptor 3 signaling pathway	3	0.20576132	0.09575229	HAVCR2, UNC93B1, SCARA3	1125	8	16792	5.59733333	1	0.9804152	84.2935963
GOTERM_BP_DIRECT	GO:0071493~cellular response to UV-B	3	0.20576132	0.09575229	CDKN1A, CRIP1, HYAL3	1125	8	16792	5.59733333	1	0.9804152	84.2935963
GOTERM_BP_DIRECT	GO:0031659~positive regulation of cyclin-dependent protein serine/threonine kinase activity involved in G1/S transition of mitotic cell cycle	3	0.20576132	0.09575229	EGFR, PIM1, PKD1	1125	8	16792	5.59733333	1	0.9804152	84.2935963
GOTERM_BP_DIRECT	GO:0042135~neurotransmitter catabolic process	3	0.20576132	0.09575229	NAALAD2, ABAT, PRIMA1	1125	8	16792	5.59733333	1	0.9804152	84.2935963
GOTERM_BP_DIRECT	GO:0001945~lymph vessel development	3	0.20576132	0.09575229	FLT4, EFNB2, FOXC1	1125	8	16792	5.59733333	1	0.9804152	84.2935963
GOTERM_BP_DIRECT	GO:0070244~negative regulation of thymocyte apoptotic process	3	0.20576132	0.09575229	RORC, VHLL, JAK3	1125	8	16792	5.59733333	1	0.9804152	84.2935963
GOTERM_BP_DIRECT	GO:0050848~regulation of calcium-mediated signaling	3	0.20576132	0.09575229	CMKLR1, PTK2B, GPR143	1125	8	16792	5.59733333	1	0.9804152	84.2935963
GOTERM_BP_DIRECT	GO:0002903~negative regulation of B cell apoptotic process	3	0.20576132	0.09575229	IRS2, BCL6, HSH2D	1125	8	16792	5.59733333	1	0.9804152	84.2935963
GOTERM_BP_DIRECT	GO:0055114~oxidation-reduction process	49	3.36076818	0.09614652	STEAP3, ALDH8A1, ALDH1L1, ENOX2, PTGS2, GLDC, AKR7A2, AKR7A3, SMOX, HHIP, SARDH, IMPDH2, SH3PXD2B, SH3PXD2A, PCYOX1L, NCF2, FA2H, CBR4, ALDH3B1, CYP4X1, DHRS2, ALOX15, DHRS4, H6PD, HSD11B2, STEAP2, TPH2, PRODH, AOC3, ME3, SORD, DHRS4L2, FASN, HAAO, GSTO2, TSTA3, CHDH, PAOX, NOX5, PYROXD2, FADS1, FOXRED2, FADS3, FAR2P1, CYP4B1, JMJD6, CYP8B1, RDH16, CBS	1125	592	16792	1.23544745	1	0.97991977	84.4190558
GOTERM_BP_DIRECT	GO:0035094~response to nicotine	6	0.41152263	0.09843375	LYPD1, HMOX1, EDN1, CHRNB4, ABAT, ATP1A2	1125	37	16792	2.42046847	1	0.98100928	85.128444
GOTERM_BP_DIRECT	GO:0045597~positive regulation of cell differentiation	6	0.41152263	0.09843375	SOCS3, CTGF, JUN, HSF4, JUNB, CYR61	1125	37	16792	2.42046847	1	0.98100928	85.128444

Cellular Component

Category	Term	Count	%	PValue	Genes	List Total	Pop Hits	Pop Total	Fold Enrichment	Bonferroni	Benjamini	FDR
GOTERM_CC_DI	GO:0016324~apical plasma men	36	2.469136	0.00054676	ABCA7, LZTS1, SLC22A18, SLC22A13, SLC6A20, ANO1, CSPG4, OXTR, AQP6, ATP6V1B1, NHS, AMN, GPR143, DDR2, AMOTL2, P2RY6, SLC23A1, ANK2, SORBS2, SCNN1A, SLC1A1, UPK2, EGFR, HPN, PLET1, GJB6, SHANK2, SLC9A3R2, CD55, ABCG5, AP2A1, DSG1, SPTBN2, AHCYL1, PDZK1, SLC14A2	1220	291	18224	1.847963495	0.25734122	0.257341	0.793741
GOTERM_CC_DI	GO:0005622~intracellular	116	7.956104	0.002478061	ALDH8A1, NRTN, ZNF823, PLEKHM1, TUFT1, ADCY6, RGL3, TNFSF15, RASGEF1C, TRIM46, ARL10, ZNF776, ANK2, PLD2, SOCS3, ZNF670-ZNF695, DDIT4, ASB9, RND3, NME3, RND1, PITPNM3, NYAP2, HSPB8, MAPK4, ZNF383, ZNF432, NYAP1, EEFSEC, LRRK1, IRAK1BP1, ZNHIT3, ZNF844, ZNF430, RAB7B, MAP4K2, ASB12, ASB13, CHMP2B, TRIM65, RASAL1, ZNF227, RAC2, RAC3, ZNF222, BLNK, MDFI, GPR155, GABARAPL1, KLB, SMAD7, KCNB1, ROGDI, RNF103-CHMP3, DVL1, ADPRH, ZNF317, MTR, DEPTOR, SYTL1, CLTCL1, ZNF534, MLPH, ZNF154, ZBBX, MCF2L, CAMKK2, ZNF350, CLCF1, MAP1LC3B, ZNF681, DOCK11, PLCB2, ANO9, ANO8, PIK3C2B, RPH3AL, STX1B, NLRP1, MAST3, GRM4, KSR2, GRM2, CAPN13, MAP3K10, PDE9A, RAPGEFL1, MAP3K12, ZNF485, CDRT1, GPR182, TRIM14, OXTR, PLCL1, RGS11, FAT4, RASGRP2, ZNF473, PLCD4, PLCD1, RASA4, CAPN5, DGKQ, NPR1, ZNF669, ZNF662, CDC25B, CAPN1, FSD1, RAB30, EPGN, TOM1L2, MAPK8IP2, ZNF461, EVI5L, ZNF573	1220	1332	18224	1.300881209	0.740691823	0.490777	3.55072
GOTERM_CC_DI	GO:0012505~endomembrane sy	16	1.097394	0.008959017	SLC2A10, TBC1D10C, SLC6A4, WDFY4, ACACB, ATP6V1B1, SLC9A3R2, BCL2L11, MCF2L, ADPRH, PITPNM3, MAP1LC3B, RAC3, EVI5L, JAK3, NRBP2	1220	114	18224	2.096519988	0.99252109	0.80444	12.28973
GOTERM_CC_DI	GO:0005737~cytoplasm	387	26.54321	0.009235902	MYLK2, MKS1, SSTR2, AAAS, MAPK4, ZNF383, ADAMTS1, MYBBP1A, IRAK1BP1, TRAPPC2, TPPP3, ACP6, CRTC1, DUSP10, CTIF, RAC2, MYO15A, PHYHIPL, DNMT3A, RECQL5, CCDC81, RPS9, CDC42BPG, MUC5AC, LZTS3, LZTS1, HNF1A, NUAK2, ELF3, NUAK1, ELF5, LATS2, NOCT, NOD2, SLC23A1, FBXL16, ERRFI1, SERTAD1, SH3PXD2B, ARHGEF1, SH3PXD2A, GMEB1, TLE1, RFTN2, SMN1, KSR2, FANCD2, FOXC1, TSPOAP1, EXOC8, NNAT, TRIM14, QTRT1, FEM1A, NECAB3, NECAB2, LRRTM4, PTK2B, PKD1, NIM1K, PHLDA3, ZNF263, FLRT1, ACTC1, DGKQ, CAPN5, LRRC45, ANKHD1, CAPN1, FSD1, MEF2D, CNTF, DUSP1, RPS6KA2, CHTF18, GAMT, RNF19B, HTR2B, DUSP8, YKT6, BPHL, IL17RE, IL11, PACSIN1, PACSIN3, OIP5, M1AP, EGFR, ENC1, FBP1, RMI2, PNPLA3, IRS1, DDIT3, DDIT4, PNPLA5, NCAM1, HSPB9, ANKRD13B, PRCD, HSPB8, SPATA18, HSPB7, SPATA17, LRRK1, CRACR2B, TRAF1, ACTBL2, ARHGDIG, LRRCC1, NHS, ISYNA1, FBXW8, PTK6, DRC7, IFT140, OVGP1, LURAP1, MYO1C, SMG7, ARMC3, NR4A2, BFSP1, NR4A1, SHANK2, APEH, HDAC4, P2RX7, PLK3, POLD1, TSC2, MYO19, NAT8L, HDAC6, ANO1, RPRM, FES, CCHCR1, FRMD6, LBH, HEY1, DDX60, OCA2, IMPDH2, SGK1, CNTN5, SLA2, PADI2, LRP2BP, PRPF3, ALDH3B1, UFL1, HAGH, DHRS2, FOLH1, CAPN13, ZFAND2A, TBCD, ZFPM2, UBAP1, STON1, HAUS2, FPGT-TNNI3K, KITLG, ESPN, A1CF, SAPCD2, NFATC4, EHD3, PPP4R3A, ALPK1, ALPK2, FLT4, DPYSL5, TAB1, HID1, CDC25B, IFIT1, CCDC110, GDF15, THEMIS2, CBS, TARS2, DZIP3, SNIP1, EDN1, AURKC, RNF213, GDPGP1, BDNF, CD44, TRAK2, TWIST2, PIM1, WNK2, VASH1, RIC8A, GPBAR1, KRT17, ACTL7B, HAS1, NOL4L, EPS8L2, KAZN, PALM, SLC2A10, ASAP3, GPR143, FUZ, DNAAF3, FBXO4, TDRD1, HIP1, BLNK, MDFI,	1220	5222	18224	1.107026389	0.993575758	0.71689	12.64612
GOTERM_CC_DI	GO:0005667~transcription facto	23	1.577503	0.010040748	MYOD1, TCF7, HNF1A, SMAD7, RCOR2, MAFB, E2F6, MLXIPL, NR4A1, ANKRD1, ZEB1, NR4A3, NR2E3, JUNB, AHR, ETS1, GATA6, JUN, FOXF2, NFATC4, POU3F1, RUNX2, FOXE3	1220	193	18224	1.780141001	0.995871223	0.666447	13.67443
GOTERM_CC_DI	GO:0016459~myosin complex	9	0.617284	0.017078019	MYH16, MYO1C, CGN, MYO15A, MYH3, MYO19, BMF, MYO18A, MYO5C	1220	50	18224	2.688786885	0.999914815	0.790238	22.19739
GOTERM_CC_DI	GO:0005829~cytosol	251	17.21536	0.018193675	CTGF, MAT1A, SULT1A1, SMOX, NUDT18, BCL2L15, NCF2, KIF5A, MYLK3, NOXA1, MYH3, SPIRE2, WNK2, BCL2L11, CARNS1, MKS1, RND1, MAPK4, JUN, TUBA4A, TRAPPC2, PFKFB4, ARHGEF25, RHPN1, ALDOB, DENND2A, PEX5L, IFI35, EPHB2, RAC2, RAC3, GYS1, FBXO4, PPP2R2C, BLNK, SMAD7, UPB1, NPL, RPS9, UBE2L6, ACACB, TNNI3, TNNI1, DVL1, NOTCH3, PLA2G4F, CLTCL1, GNAZ, ENOX2, FERMT1, PDE11A, MCF2L, LATS2, GLI1, CKB, KCNIP3, CBSL, NOD2, TKFC, MAP1LC3B, BAG3, PIK3AP1, ADCY10, DOCK11, THRSP, ERRFI1, ARHGEF1, PALD1, PHKG1, PIK3C2B, TLE1, STX1B, SMN1, ALOX15, CCND2, SERPINB8, RPL3L, GNB3, STEAP2, UROD, MAP3K12, TPH2, TSPOAP1, PPFIA3, SORD, RAP1GAP, EXOC8, HSH2D, HMMR, STAT6, GALK1, PLIN5, DAPP1, PLIN1, PTK2B, FASN, HAAO, TSTA3, RASA4, MICALL2, ACTC1, DGKQ, BAIAP2L2, MAPK11, GJB6, BIRC3, CAPN1, KHK, JMJD6, RPS6KA2, AP2A1, GAMT, RNF19B, YKT6, ALDH1L1, SLC6A4, PKMYT1, ANKRD1, ITPKA, CALB1, TBC1D16, PACSIN1, GSTM4, OPLAH, ANK2, PDE4B, STARD8, AKR7A2, ASPG, AKR7A3, MLKL, MX1, MATK, TUB, IRS2, SOCS3, TAZ, STAP2, MLXIPL, FBP1, IRS1, DDIT3, DDIT4, PNPLA5, PSMA2, NRBP2, TRAF1, ARHGDIG, B9D1, FN3K, ATP6V1B1, CHMP2B, RRAGC, RASAL1, ISYNA1, PTK6, BMF, SREBF1, OBSCN, GABARAPL1, ADSSL1, OVGP1, TBC1D10C, MYO1C, SMG7, EPHX2, AK5, LHPP, AK7, VAV2, RNF103-CHMP3, ATP7A, HDAC4, APEH, CDKN1A, PRKAR1B, TSC2, MTR, DSG1, SPTBN2, AHCYL1, PDZD3, MYLK, HDAC6, DPH5, DPH6, OGDHL, SULT2B1, CAD, FES, PLCB3, HMOX1, WWC3, YOD1, PLCB2, FOSL1, IMPDH2, EFR3B, SGK1, KIF17, PADI3, NADSYN1, PADI2, NLRP1, ALDH3B1, PPM1F, HAGH, UFL1, ARHGAP31, ARHGAP33, ZFYVE28, PDE9A, VHLL, PPP1R15A, UBAP1, HAUS5, TNFRSF25,	1220	3315	18224	1.131029844	0.999954075	0.759955	23.47428
GOTERM_CC_DI	GO:0005887~integral componer	115	7.887517	0.020065836	PLXNA3, GRIK1, TSPAN2, SLC22A13, SLC6A20, EFNA3, SLC6A4, CSPG4, JAG2, TNFSF15, IL17RE, TNFSF12, TLR5, AQP6, SLC26A10, GP5, SLC2A6, CD44, GRIN2C, CLEC4A, ADGRB1, HTR1D, GJD3, EFNB3, EFNB2, PLXNB3, MRGPRF, CRHR1, CRHR2, SSTR2, HAS1, PALM, SLC2A10, IL1R1, FGFR3, SYNDIG1, ENPP3, EPHA10, SLCO2A1, EPHB2, IL12RB2, P2RY6, XG, GAL3ST1, UPK2, KLB, SLC6A12, PLGRKT, SLC6A17, ATP7A, BFAR, CD55, P2RX7, SEMA6B, SLC4A11, ATP2A3, AVPR1B, CHRNB4, TBXA2R, SLC13A3, ATP7B, STEAP3, SLC16A13, IGDCC3, SLC5A4, GLRA1, RRH, DDR2, TSPAN11, KISS1R, SLC23A1, TSPAN10, SLC24A1, HHIP, SLC1A1, MLANA, GPR19, CMKLR1, ACKR2, CACNG4, GRM1, GRM4, FOLH1, GRM3, GRM2, STEAP2, TNFRSF25, CLCNKA, PCDHB15, BTN2A1, CLCNKB, OXTR, GPR68, GPRC5A, COL17A1, SLC18B1, PKD1, HS6ST1, SCNN1A, SELPLG, SLC8A1, HPN, FLRT1, FLT4, NPR1, ASIC1, SLC10A1, SLC16A2, EPGN, SLC16A8, HBEGF, SLC14A1, HTR2B, YKT6, SLC14A2	1220	1415	18224	1.214018421	0.99998374	0.748007	25.57326
GOTERM_CC_DI	GO:0005578~proteinaceous extr	28	1.920439	0.021922608	ADAMTS14, MAMDC2, ELN, MMP2, SMOC2, COL17A1, WNT4, CILP2, COL6A6, CTGF, CRISPLD2, FBN3, CYR61, TECTA, TFIP11, WNT10B, FLRT1, CILP, SLIT1, WNT2B, ADAMTS7, ADAMTS9, COL14A1, ADAMTS8, FBLN7, ADAMTS1, ADAMTS3, ADAMTS5	1220	268	18224	1.560655738	0.999994205	0.738113	27.60191

GOTERM_CC_DI	GO:0005886~plasma membrane	305	20.91907	0.025588896	TRAK2, GRIN2C, CTGF, SERPINE1, CLEC4A, HTR1D, ADGRB1, KLRD1, GNG7, CDH23, KLRB1, PLD2, F12, ARC, EFNB3, EFNB2, PIM1, SPIRE2, WNK2, RIC8A, CRHR1, CRHR2, SSTR2, BTN3A1, RND1, GPBAR1, HAS1, BTN3A2, EPS8L2, PALM, SLC2A10, IL1R1, ARHGEF25, CRTC1, GPR143, EPHB2, KCNJ1, RAC2, RAC3, STX11, BLNK, KLF5, TECTA, SMAD7, PCDH10, CELSR2, PCDH17, ABCG4, PCDH19, DVL1, GPR153, NOTCH3, KCNJ5, KCNJ4, CBLC, CD55, ABCG5, PKP1, PKP3, AVPR1B, CHRNB4, SYTL5, ERVW-1, SYTL1, PDZK1, GRK3, CLTCL1, GNAZ, NRP2, GPR162, GLRA1, GPR82, UNC93A, MCF2L, KCNIP3, NOD2, KISS1R, SLC23A1, BAG3, SLC24A1, STRA6, PIK3AP1, ADCY10, ANGPT2, CD200R1, GPR173, CATIP, ADAM11, ARHGEF1, CLMP, CMKLR1, PIK3C2B, CD160, RPH3AL, CACNG4, ACKR2, RFTN2, GRM1, STX1B, GRM4, GRM3, ALOX15, NAALAD2, GRM2, IL20RB, GIPR, GNB3, STEAP2, PLA2G3, RASD1, MAP3K12, SEMA4A, CLDN16, GPR182, EXOC8, PCDHB15, GPR68, GJC1, HMMR, PLCL1, DAPP1, FAT3, FAT4, FASN, PKD1, RASA4, PHLDA3, MICALL2, IL18R1, FLRT1, SLC8A1, DGKQ, BAIAP2L2, HSPG2, ATP1A3, NPR1, LRRC45, ATP1A2, CLDN24, CAPN1, DKK1, KREMEN2, JMJD6, AP2A1, SLC16A8, HBEGF, LRP8, HTR2B, TJP3, BAMBI, DNM1, ATP6V0A2, PLXNA3, SLC22A18, KCNC3, GRIK1, SLC6A20, SLC6A4, JAG2, TNFSF15, IL17RE, TLR5, TNFSF12, MMP2, SLC2A6, PACSIN1, GP5, WNT4, DYSF, PACSIN3, ANK2, ZNF185, MLKL, SCAMP5, EGFR, TUB, IRS2, KCND3, PLXNB3, STAP2, MRGPRF, HDAC11, IRS1, NCAM1, ANKRD13B, NRSN2, IGSF9, ABCA7, ME3, FGFR3, ITGB4, KATNB1, EPHA10, PAQR8, LRIG3, CHMP2B, SLCO2A1, IL12RB2, PDE6B, RASAL1, KCNS1, P2RY6, ENTPD8, SLC30A2, PRIMA1, BMF, GABRP, TBC1D10C, KLB, MYO1C, KCNB1, SLC6A12, CACNA1I,	1220	4121	18224	1.105556904	0.999999249	0.755895	31.45723
GOTERM_CC_DI	GO:0098793~presynapse	10	0.685871	0.02589049	PLD2, TSPOAP1, P2RX7, SYNDIG1, SLC6A4, SYT8, SYTL5, SYTL1, HIP1, DVL1	1220	64	18224	2.334016393	0.999999365	0.726723	31.7657
GOTERM_CC_DI	GO:0030017~sarcomere	7	0.48011	0.043408656	ACTC1, ARHGEF25, MYH3, MYLK2, ANKRD1, TNNI3, MMP2	1220	39	18224	2.681126524	1	0.866259	47.62032
GOTERM_CC_DI	GO:0030424~axon	22	1.508916	0.068607073	NRP2, PALM, NRTN, KCNC3, RAP1GAP, KCNB1, ATP1A3, KATNB1, CALB1, EPHB2, DVL1, GRM3, CNTF, GRM2, PTK2B, NEFH, LRP8, ADCY10, CHRNA10, IGSF9, MAP3K12, HDAC6	1220	222	18224	1.4803131	1	0.94891	64.49966
GOTERM_CC_DI	GO:0005925~focal adhesion	35	2.400549	0.070218234	FGFR3, CNN3, TLN2, FERMT1, CSPG4, L1CAM, ASAP3, NHS, FES, DDR2, ATP6V0C, CD44, RAC2, PTK2B, SORBS2, ZNF185, STARD8, AATF, EHD3, EGFR, ACTC1, FLRT1, CAPN5, EFNB2, HSPG2, RPS9, MISP, NEXN, SLC9A3R2, CAPN1, ARHGAP31, RND3, LAYN, FBLN7, ATP6V0A2	1220	391	18224	1.337134711	1	0.940929	65.38404
GOTERM_CC_DI	GO:0043197~dendritic spine	12	0.823045	0.070350612	PALM, SLC8A1, ARC, GRM3, LZTS1, SYNDIG1, CNN3, PDE4B, ATP1A2, SHANK2, ITPKA, DVL1	1220	100	18224	1.79252459	1	0.929035	65.45579
GOTERM_CC_DI	GO:0005811~lipid particle	9	0.617284	0.072342171	TRAP1, PLIN5, ALOX15, PLIN1, ABHD4, LSS, METTL7A, PNPLA3, PNPLA5	1220	66	18224	2.036959762	1	0.922163	66.51856
GOTERM_CC_DI	GO:0043025~neuronal cell body	29	1.989026	0.075596228	RAP1GAP, GLRA1, CNN3, CRTC1, KATNB1, CAD, CALB1, EPHB2, RAC3, PTK2B, PYCARD, ADCY10, HPN, KIF5A, ENC1, DPYSL5, SHANK2, TMPRSS3, NLRP1, DVL1, ATP7A, P2RX7, PLK3, MAPK8IP2, SPTBN2, NRSN2, LRP8, YKT6, LRP4	1220	315	18224	1.375217278	1	0.91917	68.18975
GOTERM_CC_DI	GO:0005604~basement membra	10	0.685871	0.080593736	HMCN2, SMOC2, COL17A1, LAMA5, EGFL6, HSPG2, ADAMTS1, VTN, VWA2, MMRN2	1220	79	18224	1.890848724	1	0.921093	70.60571
GOTERM_CC_DI	GO:0043190~ATP-binding casset	3	0.205761	0.095639571	ABCA7, ABCG5, ABCA2	1220	8	18224	5.601639344	1	0.943767	76.88745

Molecular function			
**ID**	**Gene Name**	**Species**	**GOTERM_MF_DIRECT**
ACCS	1-aminocyclopropane-1-carboxylate synthase homolog (inactive)(ACCS)	Homo sapiens	GO:0003824~catalytic activity,GO:0005515~protein binding,GO:0016847~1-aminocyclopropane-1-carboxylate synthase activity,GO:0030170~pyridoxal phosphate binding,GO:0042802~identical protein binding,GO:0042803~protein homodimerization activity,
HAAO	3-hydroxyanthranilate 3,4-dioxygenase(HAAO)	Homo sapiens	GO:0000334~3-hydroxyanthranilate 3,4-dioxygenase activity,GO:0005506~iron ion binding,GO:0005515~protein binding,GO:0008198~ferrous iron binding,GO:0009055~electron carrier activity,
HMGCL	3-hydroxymethyl-3-methylglutaryl-CoA lyase(HMGCL)	Homo sapiens	GO:0000062~fatty-acyl-CoA binding,GO:0000287~magnesium ion binding,GO:0004419~hydroxymethylglutaryl-CoA lyase activity,GO:0005102~receptor binding,GO:0016829~lyase activity,GO:0030145~manganese ion binding,GO:0031406~carboxylic acid binding,GO:0042803~protein homodimerization activity,GO:0046872~metal ion binding,
ABAT	4-aminobutyrate aminotransferase(ABAT)	Homo sapiens	GO:0003867~4-aminobutyrate transaminase activity,GO:0008483~transaminase activity,GO:0030170~pyridoxal phosphate binding,GO:0032145~succinate-semialdehyde dehydrogenase binding,GO:0042803~protein homodimerization activity,GO:0046872~metal ion binding,GO:0047298~(S)-3-amino-2-methylpropionate transaminase activity,GO:0051536~iron-sulfur cluster binding,
HOGA1	4-hydroxy-2-oxoglutarate aldolase 1(HOGA1)	Homo sapiens	GO:0008700~4-hydroxy-2-oxoglutarate aldolase activity,GO:0016829~lyase activity,GO:0042803~protein homodimerization activity,
HTR1D	5-hydroxytryptamine receptor 1D(HTR1D)	Homo sapiens	GO:0004993~G-protein coupled serotonin receptor activity,GO:0030594~neurotransmitter receptor activity,GO:0051378~serotonin binding,
HTR2B	5-hydroxytryptamine receptor 2B(HTR2B)	Homo sapiens	GO:0001965~G-protein alpha-subunit binding,GO:0004993~G-protein coupled serotonin receptor activity,GO:0005096~GTPase activator activity,GO:0008144~drug binding,GO:0030594~neurotransmitter receptor activity,GO:0051378~serotonin binding,
MTR	5-methyltetrahydrofolate-homocysteine methyltransferase(MTR)	Homo sapiens	GO:0005515~protein binding,GO:0008270~zinc ion binding,GO:0008705~methionine synthase activity,GO:0008898~S-adenosylmethionine-homocysteine S-methyltransferase activity,GO:0031419~cobalamin binding,
OPLAH	5-oxoprolinase (ATP-hydrolysing)(OPLAH)	Homo sapiens	GO:0005524~ATP binding,GO:0016787~hydrolase activity,GO:0017168~5-oxoprolinase (ATP-hydrolyzing) activity,
PFKFB4	6-phosphofructo-2-kinase/fructose-2,6-biphosphatase 4(PFKFB4)	Homo sapiens	GO:0003824~catalytic activity,GO:0003873~6-phosphofructo-2-kinase activity,GO:0004331~fructose-2,6-bisphosphate 2-phosphatase activity,GO:0005524~ATP binding,
ADAM11	ADAM metallopeptidase domain 11(ADAM11)	Homo sapiens	GO:0004222~metalloendopeptidase activity,GO:0005178~integrin binding,GO:0008237~metallopeptidase activity,
ADAM20	ADAM metallopeptidase domain 20(ADAM20)	Homo sapiens	GO:0004222~metalloendopeptidase activity,GO:0008237~metallopeptidase activity,GO:0046872~metal ion binding,
ADAM32	ADAM metallopeptidase domain 32(ADAM32)	Homo sapiens	GO:0004222~metalloendopeptidase activity,
ADAM33	ADAM metallopeptidase domain 33(ADAM33)	Homo sapiens	GO:0004222~metalloendopeptidase activity,GO:0008237~metallopeptidase activity,GO:0008270~zinc ion binding,
ADAMTS1	ADAM metallopeptidase with thrombospondin type 1 motif 1(ADAMTS1)	Homo sapiens	GO:0004222~metalloendopeptidase activity,GO:0008201~heparin binding,GO:0008237~metallopeptidase activity,GO:0008270~zinc ion binding,
ADAMTS1	ADAM metallopeptidase with thrombospondin type 1 motif 14(ADAMTS14)	Homo sapiens	GO:0004222~metalloendopeptidase activity,GO:0008270~zinc ion binding,
ADAMTS3	ADAM metallopeptidase with thrombospondin type 1 motif 3(ADAMTS3)	Homo sapiens	GO:0004175~endopeptidase activity,GO:0004222~metalloendopeptidase activity,GO:0005515~protein binding,GO:0008201~heparin binding,GO:0008233~peptidase activity,GO:0008270~zinc ion binding,
ADAMTS5	ADAM metallopeptidase with thrombospondin type 1 motif 5(ADAMTS5)	Homo sapiens	GO:0004222~metalloendopeptidase activity,GO:0005178~integrin binding,GO:0005515~protein binding,GO:0008201~heparin binding,GO:0008237~metallopeptidase activity,GO:0008270~zinc ion binding,GO:0050840~extracellular matrix binding,
ADAMTS7	ADAM metallopeptidase with thrombospondin type 1 motif 7(ADAMTS7)	Homo sapiens	GO:0004222~metalloendopeptidase activity,GO:0005515~protein binding,GO:0008233~peptidase activity,GO:0008237~metallopeptidase activity,GO:0008270~zinc ion binding,
ADAMTS8	ADAM metallopeptidase with thrombospondin type 1 motif 8(ADAMTS8)	Homo sapiens	GO:0004222~metalloendopeptidase activity,GO:0005178~integrin binding,GO:0008201~heparin binding,GO:0008237~metallopeptidase activity,GO:0008270~zinc ion binding,GO:0009673~low-affinity phosphate transmembrane transporter activity,
ADAMTS9	ADAM metallopeptidase with thrombospondin type 1 motif 9(ADAMTS9)	Homo sapiens	GO:0004222~metalloendopeptidase activity,GO:0008237~metallopeptidase activity,GO:0008270~zinc ion binding,
ADAMTSL	ADAMTS like 4(ADAMTSL4)	Homo sapiens	GO:0002020~protease binding,GO:0004222~metalloendopeptidase activity,GO:0005515~protein binding,GO:0008233~peptidase activity,
ARL10	ADP ribosylation factor like GTPase 10(ARL10)	Homo sapiens	GO:0005525~GTP binding,
ADPRH	ADP-ribosylarginine hydrolase(ADPRH)	Homo sapiens	GO:0000287~magnesium ion binding,GO:0003875~ADP-ribosylarginine hydrolase activity,GO:0005096~GTPase activator activity,GO:0005515~protein binding,GO:0016787~hydrolase activity,GO:0017137~Rab GTPase binding,
AFF3	AF4/FMR2 family member 3(AFF3)	Homo sapiens	GO:0003690~double-stranded DNA binding,
ALG1L	ALG1, chitobiosyldiphosphodolichol beta-mannosyltransferase like(ALG1L)	Homo sapiens	GO:0000030~mannosyltransferase activity,GO:0016757~transferase activity, transferring glycosyl groups,
A1CF	APOBEC1 complementation factor(A1CF)	Homo sapiens	GO:0000166~nucleotide binding,GO:0003723~RNA binding,GO:0003725~double-stranded RNA binding,GO:0003727~single-stranded RNA binding,GO:0005515~protein binding,
ABCA2	ATP binding cassette subfamily A member 2(ABCA2)	Homo sapiens	GO:0000166~nucleotide binding,GO:0005215~transporter activity,GO:0005524~ATP binding,GO:0016887~ATPase activity,GO:0042626~ATPase activity, coupled to transmembrane movement of substances,
ABCA7	ATP binding cassette subfamily A member 7(ABCA7)	Homo sapiens	GO:0005215~transporter activity,GO:0005524~ATP binding,GO:0016887~ATPase activity,GO:0034188~apolipoprotein A-I receptor activity,GO:0042626~ATPase activity, coupled to transmembrane movement of substances,GO:0090554~phosphatidylcholine-translocating ATPase activity,GO:0090556~phosphatidylserine-translocating ATPase activity,
ABCG4	ATP binding cassette subfamily G member 4(ABCG4)	Homo sapiens	GO:0005515~protein binding,GO:0005524~ATP binding,GO:0016887~ATPase activity,GO:0017127~cholesterol transporter activity,GO:0034041~sterol-transporting ATPase activity,GO:0042803~protein homodimerization activity,GO:0046982~protein heterodimerization activity,
ABCG5	ATP binding cassette subfamily G member 5(ABCG5)	Homo sapiens	GO:0005515~protein binding,GO:0005524~ATP binding,GO:0016887~ATPase activity,GO:0017127~cholesterol transporter activity,GO:0042626~ATPase activity, coupled to transmembrane movement of substances,GO:0046982~protein heterodimerization activity,
ATP6V0A	ATPase H+ transporting V0 subunit a2(ATP6V0A2)	Homo sapiens	GO:0005515~protein binding,GO:0015078~hydrogen ion transmembrane transporter activity,GO:0046961~proton-transporting ATPase activity, rotational mechanism,GO:0051117~ATPase binding,
ATP6V0C	ATPase H+ transporting V0 subunit c(ATP6V0C)	Homo sapiens	GO:0005515~protein binding,GO:0015078~hydrogen ion transmembrane transporter activity,GO:0031625~ubiquitin protein ligase binding,GO:0046933~proton-transporting ATP synthase activity, rotational mechanism,GO:0046961~proton-transporting ATPase activity, rotational mechanism,

ATP6V1B	ATPase H+ transporting V1 subunit B1(ATP6V1B1)	Homo sapiens	GO:0005524~ATP binding,GO:0015078~hydrogen ion transmembrane transporter activity,GO:0016820~hydrolase activity, acting on acid anhydrides, catalyzing transmembrane movement of substances,GO:0032403~protein complex binding,
ATP6AP1I	ATPase H+ transporting accessory protein 1 like(ATP6AP1L)	Homo sapiens	GO:0046933~proton-transporting ATP synthase activity, rotational mechanism,GO:0046961~proton-transporting ATPase activity, rotational mechanism,
ATP1A2	ATPase Na+/K+ transporting subunit alpha 2(ATP1A2)	Homo sapiens	GO:0000166~nucleotide binding,GO:0005391~sodium:potassium-exchanging ATPase activity,GO:0005515~protein binding,GO:0005524~ATP binding,GO:0008144~drug binding,GO:0016887~ATPase activity,GO:0030955~potassium ion binding,GO:0031402~sodium ion binding,GO:0046872~metal ion binding,GO:0051087~chaperone binding,GO:1990239~steroid hormone binding,
ATP1A3	ATPase Na+/K+ transporting subunit alpha 3(ATP1A3)	Homo sapiens	GO:0000166~nucleotide binding,GO:0005391~sodium:potassium-exchanging ATPase activity,GO:0005524~ATP binding,GO:0031748~D1 dopamine receptor binding,GO:0043395~heparan sulfate proteoglycan binding,GO:0046872~metal ion binding,GO:0051087~chaperone binding,GO:0086037~sodium:potassium-exchanging ATPase activity involved in regulation of cardiac muscle cell membrane potential,GO:1990239~steroid hormone binding,
ATP7A	ATPase copper transporting alpha(ATP7A)	Homo sapiens	GO:0000166~nucleotide binding,GO:0004008~copper-exporting ATPase activity,GO:0005375~copper ion transmembrane transporter activity,GO:0005507~copper ion binding,GO:0005515~protein binding,GO:0005524~ATP binding,GO:0016532~superoxide dismutase copper chaperone activity,GO:0019829~cation-transporting ATPase activity,GO:0032767~copper-dependent protein binding,
ATP7B	ATPase copper transporting beta(ATP7B)	Homo sapiens	GO:0000166~nucleotide binding,GO:0004008~copper-exporting ATPase activity,GO:0005507~copper ion binding,GO:0005515~protein binding,GO:0005524~ATP binding,GO:0019829~cation-transporting ATPase activity,GO:0043682~copper-transporting ATPase activity,GO:0046872~metal ion binding,
ATAD3A	ATPase family, AAA domain containing 3A(ATAD3A)	Homo sapiens	GO:0005524~ATP binding,
ATP8B3	ATPase phospholipid transporting 8B3(ATP8B3)	Homo sapiens	GO:0000287~magnesium ion binding,GO:0004012~phospholipid-translocating ATPase activity,GO:0005524~ATP binding,
ATP2A3	ATPase sarcoplasmic/endoplasmic reticulum Ca2+ transporting 3(ATP2A3)	Homo sapiens	GO:0005388~calcium-transporting ATPase activity,GO:0005524~ATP binding,GO:0046872~metal ion binding,
ASAP3	ArfGAP with SH3 domain, ankyrin repeat and PH domain 3(ASAP3)	Homo sapiens	GO:0005096~GTPase activator activity,GO:0005515~protein binding,GO:0046872~metal ion binding,
ACAP3	ArfGAP with coiled-coil, ankyrin repeat and PH domains 3(ACAP3)	Homo sapiens	GO:0005096~GTPase activator activity,GO:0046872~metal ion binding,
BSPRY	containing(BSPRY)	Homo sapiens	GO:0008270~zinc ion binding,
BCL6	B-cell CLL/lymphoma 6(BCL6)	Homo sapiens	GO:0000977~RNA polymerase II regulatory region sequence-specific DNA binding,GO:0001161~intronic transcription regulatory region sequence-specific DNA binding,GO:0001227~transcriptional repressor activity, RNA polymerase II transcription regulatory region sequence-specific binding,GO:0003676~nucleic acid binding,GO:0003682~chromatin binding,GO:0003700~transcription factor activity, sequence-specific DNA binding,GO:0005515~protein binding,GO:0031490~chromatin DNA binding,GO:0042802~identical protein binding,GO:0043565~sequence-specific DNA binding,GO:0046872~metal ion binding,
BLNK	B-cell linker(BLNK)	Homo sapiens	GO:0005068~transmembrane receptor protein tyrosine kinase adaptor activity,GO:0005070~SH3/SH2 adaptor activity,GO:0005515~protein binding,
B9D1	B9 domain containing 1(B9D1)	Homo sapiens	GO:0008158~hedgehog receptor activity,
BAIAP2L2	BAI1 associated protein 2 like 2(BAIAP2L2)	Homo sapiens	GO:0005543~phospholipid binding,GO:0008093~cytoskeletal adaptor activity,
BAG3	BCL2 associated athanogene 3(BAG3)	Homo sapiens	GO:0000774~adenyl-nucleotide exchange factor activity,GO:0005515~protein binding,GO:0032403~protein complex binding,GO:0051087~chaperone binding,GO:0098641~cadherin binding involved in cell-cell adhesion,
BCL2L11	BCL2 like 11(BCL2L11)	Homo sapiens	GO:0005515~protein binding,GO:0008017~microtubule binding,GO:0045502~dynein binding,
BCL2L15	BCL2 like 15(BCL2L15)	Homo sapiens	GO:0005515~protein binding,
BAMBI	BMP and activin membrane bound inhibitor(BAMBI)	Homo sapiens	GO:0005109~frizzled binding,GO:0005114~type II transforming growth factor beta receptor binding,
BRF2	BRF2, RNA polymerase III transcription initiation factor 50 kDa subunit(BRF2)	Homo sapiens	GO:0001007~transcription factor activity, RNA polymerase III transcription factor binding,GO:0001032~RNA polymerase III type 3 promoter DNA binding,GO:0008270~zinc ion binding,
BUD31	BUD31 homolog(BUD31)	Homo sapiens	GO:0003700~transcription factor activity, sequence-specific DNA binding,GO:0005515~protein binding,GO:0030374~ligand-dependent nuclear receptor transcription coactivator activity,GO:0035257~nuclear hormone receptor binding,
BMF	Bcl2 modifying factor(BMF)	Homo sapiens	GO:0005515~protein binding,
CLEC4A	C-type lectin domain family 4 member A(CLEC4A)	Homo sapiens	GO:0004888~transmembrane signaling receptor activity,GO:0030246~carbohydrate binding,
CASKIN1	CASK interacting protein 1(CASKIN1)	Homo sapiens	GO:0005515~protein binding,
CBFA2T3	CBFA2/RUNX1 translocation partner 3(CBFA2T3)	Homo sapiens	GO:0003700~transcription factor activity, sequence-specific DNA binding,GO:0003714~transcription corepressor activity,GO:0005515~protein binding,GO:0046872~metal ion binding,
CD101	CD101 molecule(CD101)	Homo sapiens	GO:0016812~hydrolase activity, acting on carbon-nitrogen (but not peptide) bonds, in cyclic amides,
CD160	CD160 molecule(CD160)	Homo sapiens	GO:0004872~receptor activity,GO:0005102~receptor binding,GO:0005515~protein binding,GO:0032393~MHC class I receptor activity,
CD200R1	CD200 receptor 1(CD200R1)	Homo sapiens	GO:0004872~receptor activity,GO:0005515~protein binding,
CD4	CD4 molecule(CD4)	Homo sapiens	GO:0001618~virus receptor activity,GO:0001948~glycoprotein binding,GO:0004872~receptor activity,GO:0004888~transmembrane signaling receptor activity,GO:0005201~extracellular matrix structural constituent,GO:0005515~protein binding,GO:0008270~zinc ion binding,GO:0015026~coreceptor activity,GO:0019865~immunoglobulin binding,GO:0019899~enzyme binding,GO:0019901~protein kinase binding,GO:0042289~MHC class II protein binding,GO:0042803~protein homodimerization activity,GO:1990782~protein tyrosine kinase binding,
CD44	CD44 molecule (Indian blood group)(CD44)	Homo sapiens	GO:0004896~cytokine receptor activity,GO:0005515~protein binding,GO:0005518~collagen binding,GO:0005540~hyaluronic acid binding,
CD55	CD55 molecule (Cromer blood group)(CD55)	Homo sapiens	GO:0001618~virus receptor activity,GO:0004857~enzyme inhibitor activity,GO:0005515~protein binding,GO:0008289~lipid binding,
CDC42BP(	CDC42 binding protein kinase gamma(CDC42BPG)	Homo sapiens	GO:0000287~magnesium ion binding,GO:0004674~protein serine/threonine kinase activity,GO:0005515~protein binding,GO:0005524~ATP binding,
CDKN2AII	CDKN2A interacting protein(CDKN2AIP)	Homo sapiens	GO:0002039~p53 binding,GO:0003723~RNA binding,GO:0005515~protein binding,
CDRT1	CMT1A duplicated region transcript 1(CDRT1)	Homo sapiens	GO:0008270~zinc ion binding,
CRTC1	CREB regulated transcription coactivator 1(CRTC1)	Homo sapiens	GO:0005515~protein binding,GO:0008140~cAMP response element binding protein binding,
CREBZF	CREB/ATF bZIP transcription factor(CREBZF)	Homo sapiens	GO:0003677~DNA binding,GO:0003700~transcription factor activity, sequence-specific DNA binding,GO:0005515~protein binding,GO:0042802~identical protein binding,GO:0043565~sequence-specific DNA binding,
CELF5	CUGBP, Elav-like family member 5(CELF5)	Homo sapiens	GO:0000166~nucleotide binding,GO:0003676~nucleic acid binding,GO:0003723~RNA binding,

CBLC	Cbl proto-oncogene C(CBLC)	Homo sapiens	GO:0001784~phosphotyrosine binding,GO:0004871~signal transducer activity,GO:0005154~epidermal growth factor receptor binding,GO:0005509~calcium ion binding,GO:0008270~zinc ion binding,GO:0016874~ligase activity,GO:0017124~SH3 domain binding,GO:0030971~receptor tyrosine kinase binding,GO:0061630~ubiquitin protein ligase activity,
D2HGDH	D-2-hydroxyglutarate dehydrogenase(D2HGDH)	Homo sapiens	GO:0003824~catalytic activity,GO:0004458~D-lactate dehydrogenase (cytochrome) activity,GO:0016614~oxidoreductase activity, acting on CH-OH group of donors,GO:0050660~flavin adenine dinucleotide binding,GO:0051990~(R)-2-hydroxyglutarate dehydrogenase activity,GO:0071949~FAD binding,
DZIP3	DAZ interacting zinc finger protein 3(DZIP3)	Homo sapiens	GO:0003723~RNA binding,GO:0004842~ubiquitin-protein transferase activity,GO:0005515~protein binding,GO:0008270~zinc ion binding,GO:0016874~ligase activity,GO:0019902~phosphatase binding,GO:0031593~polyubiquitin binding,GO:0044822~poly(A) RNA binding,GO:0061630~ubiquitin protein ligase activity,
DCAF11	DDB1 and CUL4 associated factor 11(DCAF11)	Homo sapiens	GO:0005515~protein binding,
DDX1	DEAD-box helicase 1(DDX1)	Homo sapiens	GO:0003676~nucleic acid binding,GO:0003677~DNA binding,GO:0003682~chromatin binding,GO:0003712~transcription cofactor activity,GO:0003724~RNA helicase activity,GO:0003725~double-stranded RNA binding,GO:0004004~ATP-dependent RNA helicase activity,GO:0004386~helicase activity,GO:0004518~nuclease activity,GO:0004527~exonuclease activity,GO:0005515~protein binding,GO:0005524~ATP binding,GO:0008026~ATP-dependent helicase activity,GO:0008143~poly(A) binding,GO:0033677~DNA/RNA helicase activity,GO:0044822~poly(A) RNA binding,
DDX24	DEAD-box helicase 24(DDX24)	Homo sapiens	GO:0003676~nucleic acid binding,GO:0003724~RNA helicase activity,GO:0004004~ATP-dependent RNA helicase activity,GO:0004386~helicase activity,GO:0005524~ATP binding,GO:0044822~poly(A) RNA binding,
DDX47	DEAD-box helicase 47(DDX47)	Homo sapiens	GO:0003676~nucleic acid binding,GO:0004004~ATP-dependent RNA helicase activity,GO:0004386~helicase activity,GO:0005515~protein binding,GO:0005524~ATP binding,GO:0044822~poly(A) RNA binding,
DDX60L	DEAD-box helicase 60-like(DDX60L)	Homo sapiens	GO:0003676~nucleic acid binding,GO:0003723~RNA binding,GO:0004386~helicase activity,GO:0005524~ATP binding,
DENND2A	DENN domain containing 2A(DENND2A)	Homo sapiens	GO:0017112~Rab guanyl-nucleotide exchange factor activity,
DENND6E	DENN domain containing 6B(DENND6B)	Homo sapiens	GO:0017112~Rab guanyl-nucleotide exchange factor activity,
DEPTOR	DEP domain containing MTOR-interacting protein(DEPTOR)	Homo sapiens	GO:0005515~protein binding,
DDX39B	DExD-box helicase 39B(DDX39B)	Homo sapiens	GO:0003676~nucleic acid binding,GO:0004004~ATP-dependent RNA helicase activity,GO:0005515~protein binding,GO:0005524~ATP binding,GO:0008186~RNA-dependent ATPase activity,GO:0016887~ATPase activity,GO:0017070~U6 snRNA binding,GO:0030621~U4 snRNA binding,GO:0032403~protein complex binding,GO:0042802~identical protein binding,GO:0043008~ATP-dependent protein binding,GO:0044822~poly(A) RNA binding,
DDX60	DExD/H-box helicase 60(DDX60)	Homo sapiens	GO:0003690~double-stranded DNA binding,GO:0003725~double-stranded RNA binding,GO:0003727~single-stranded RNA binding,GO:0004386~helicase activity,GO:0005515~protein binding,GO:0005524~ATP binding,
DDIT3	DNA damage inducible transcript 3(DDIT3)	Homo sapiens	GO:0000976~transcription regulatory region sequence-specific DNA binding,GO:0000978~RNA polymerase II core promoter proximal region sequence-specific DNA binding,GO:0001077~transcriptional activator activity, RNA polymerase II core promoter proximal region sequence-specific binding,GO:0003677~DNA binding,GO:0003700~transcription factor activity, sequence-specific DNA binding,GO:0003714~transcription corepressor activity,GO:0005515~protein binding,GO:0008134~transcription factor binding,GO:0008140~cAMP response element binding protein binding,GO:0042803~protein homodimerization activity,GO:0043522~leucine zipper domain binding,GO:0043565~sequence-specific DNA binding,GO:0044212~transcription regulatory region DNA binding,GO:0046982~protein heterodimerization activity,
DDIT4	4(DDIT4)	Homo sapiens	GO:0071889~14-3-3 protein binding,
DNMT3A	DNA methyltransferase 3 alpha(DNMT3A)	Homo sapiens	GO:0003677~DNA binding,GO:0003682~chromatin binding,GO:0003886~DNA (cytosine-5-)-methyltransferase activity,GO:0005515~protein binding,GO:0009008~DNA-methyltransferase activity,GO:0042802~identical protein binding,GO:0045322~unmethylated CpG binding,GO:0046872~metal ion binding,GO:0051718~DNA (cytosine-5-)-methyltransferase activity, acting on CpG substrates,
POLD1	DNA polymerase delta 1, catalytic subunit(POLD1)	Homo sapiens	GO:0000166~nucleotide binding,GO:0003676~nucleic acid binding,GO:0003677~DNA binding,GO:0003682~chromatin binding,GO:0003684~damaged DNA binding,GO:0003887~DNA-directed DNA polymerase activity,GO:0005515~protein binding,GO:0008296~3'-5'-exodeoxyribonuclease activity,GO:0008408~3'-5' exonuclease activity,GO:0019899~enzyme binding,GO:0046872~metal ion binding,GO:0051539~4 iron, 4 sulfur cluster binding,
DPY19L2F	DPY19L2 pseudogene 2(DPY19L2P2)	Homo sapiens	GO:0000030~mannosyltransferase activity,
E2F6	E2F transcription factor 6(E2F6)	Homo sapiens	GO:0003677~DNA binding,GO:0003700~transcription factor activity, sequence-specific DNA binding,GO:0003714~transcription corepressor activity,GO:0005515~protein binding,GO:0046983~protein dimerization activity,
ELF3	E74 like ETS transcription factor 3(ELF3)	Homo sapiens	GO:0000978~RNA polymerase II core promoter proximal region sequence-specific DNA binding,GO:0000981~RNA polymerase II transcription factor activity, sequence-specific DNA binding,GO:0001077~transcriptional activator activity, RNA polymerase II core promoter proximal region sequence-specific binding,GO:0003700~transcription factor activity, sequence-specific DNA binding,GO:0003713~transcription coactivator activity,GO:0005515~protein binding,GO:0043565~sequence-specific DNA binding,
ELF5	E74 like ETS transcription factor 5(ELF5)	Homo sapiens	GO:0000977~RNA polymerase II regulatory region sequence-specific DNA binding,GO:0000981~RNA polymerase II transcription factor activity, sequence specific DNA binding,GO:0001228~transcriptional activator activity, RNA polymerase II transcription regulatory region sequence-specific binding,GO:0043565~sequence-specific DNA binding,
EFCC1	EF-hand and coiled-coil domain containing 1(EFCC1)	Homo sapiens	GO:0005509~calcium ion binding,
EFCAB8	EF-hand calcium binding domain 8(EFCAB8)	Homo sapiens	GO:0005509~calcium ion binding,
EFR3B	EFR3 homolog B(EFR3B)	Homo sapiens	GO:0005515~protein binding,
EGFL6	EGF like domain multiple 6(EGFL6)	Homo sapiens	GO:0005178~integrin binding,GO:0005509~calcium ion binding,
EHD3	EH domain containing 3(EHD3)	Homo sapiens	GO:0003676~nucleic acid binding,GO:0005509~calcium ion binding,GO:0005515~protein binding,GO:0005524~ATP binding,GO:0005525~GTP binding,
EPHA10	EPH receptor A10(EPHA10)	Homo sapiens	GO:0004672~protein kinase activity,GO:0005003~ephrin receptor activity,GO:0005005~transmembrane-ephrin receptor activity,GO:0005515~protein binding,GO:0005524~ATP binding,

EPHB2	EPH receptor B2(EPHB2)	Homo sapiens	GO:0004713~protein tyrosine kinase activity,GO:0005003~ephrin receptor activity,GO:0005005~transmembrane-ephrin receptor activity,GO:0005102~receptor binding,GO:0005515~protein binding,GO:0005524~ATP binding,GO:0008046~axon guidance receptor activity,GO:0042802~identical protein binding,
EPS8L2	EPS8 like 2(EPS8L2)	Homo sapiens	GO:0003779~actin binding,GO:0005089~Rho guanyl-nucleotide exchange factor activity,GO:0030676~Rac guanyl-nucleotide exchange factor activity,GO:0098641~cadherin binding involved in cell-cell adhesion,
EMC6	ER membrane protein complex subunit 6(EMC6)	Homo sapiens	GO:0005515~protein binding,
ERRFI1	ERBB receptor feedback inhibitor 1(ERRFI1)	Homo sapiens	GO:0005096~GTPase activator activity,GO:0005515~protein binding,GO:0017124~SH3 domain binding,GO:0019901~protein kinase binding,GO:0031267~small GTPase binding,
ERCC2	ERCC excision repair 2, TFIIH core complex helicase subunit(ERCC2)	Homo sapiens	GO:0003677~DNA binding,GO:0004003~ATP-dependent DNA helicase activity,GO:0004672~protein kinase activity,GO:0005515~protein binding,GO:0005524~ATP binding,GO:0008022~protein C-terminus binding,GO:0008094~DNA-dependent ATPase activity,GO:0008353~RNA polymerase II carboxy-terminal domain kinase activity,GO:0043139~5'-3' DNA helicase activity,GO:0046872~metal ion binding,GO:0047485~protein N-terminus binding,GO:0051539~4 iron, 4 sulfur cluster binding,
ETS1	ETS proto-oncogene 1, transcription factor(ETS1)	Homo sapiens	specific DNA binding,GO:0000982~transcription factor activity, RNA polymerase II core promoter proximal region sequence-specific binding,GO:0003677~DNA binding,GO:0003700~transcription factor activity, sequence-specific DNA binding,GO:0005515~protein binding,GO:0008134~transcription factor binding,GO:0035035~histone acetyltransferase binding,GO:0042802~identical protein binding,GO:0043565~sequence-specific DNA binding,
ETV3	ETS variant 3(ETV3)	Homo sapiens	GO:0000977~RNA polymerase II regulatory region sequence-specific DNA binding,GO:0000981~RNA polymerase II transcription factor activity, sequence specific DNA binding,GO:0001227~transcriptional repressor activity, RNA polymerase II transcription regulatory region sequence-specific binding,GO:0017151~DEAD/H-box RNA helicase binding,
FBXW8	F-box and WD repeat domain containing 8(FBXW8)	Homo sapiens	GO:0004842~ubiquitin-protein transferase activity,GO:0005515~protein binding,
FBXO4	F-box protein 4(FBXO4)	Homo sapiens	GO:0004842~ubiquitin-protein transferase activity,GO:0005515~protein binding,GO:0042803~protein homodimerization activity,GO:0061630~ubiquitin protein ligase activity,
FBXO43	F-box protein 43(FBXO43)	Homo sapiens	GO:0046872~metal ion binding,
FOXRED2	FAD dependent oxidoreductase domain containing 2(FOXRED2)	Homo sapiens	GO:0001948~glycoprotein binding,GO:0004497~monooxygenase activity,GO:0005515~protein binding,GO:0016491~oxidoreductase activity,GO:0050660~flavin adenine dinucleotide binding,
FAM20C	FAM20C, golgi associated secretory pathway kinase(FAM20C)	Homo sapiens	GO:0004674~protein serine/threonine kinase activity,GO:0005509~calcium ion binding,GO:0005515~protein binding,GO:0005524~ATP binding,GO:0030145~manganese ion binding,
FAT3	FAT atypical cadherin 3(FAT3)	Homo sapiens	GO:0005509~calcium ion binding,
FAT4	FAT atypical cadherin 4(FAT4)	Homo sapiens	GO:0005509~calcium ion binding,GO:0005515~protein binding,
FRMD6	FERM domain containing 6(FRMD6)	Homo sapiens	GO:0005200~structural constituent of cytoskeleton,GO:0005515~protein binding,
FES	FES proto-oncogene, tyrosine kinase(FES)	Homo sapiens	GO:0004713~protein tyrosine kinase activity,GO:0004715~non-membrane spanning protein tyrosine kinase activity,GO:0005515~protein binding,GO:0005524~ATP binding,GO:0008017~microtubule binding,GO:0034987~immunoglobulin receptor binding,GO:0035091~phosphatidylinositol binding,
FOSL1	FOS like 1, AP-1 transcription factor subunit(FOSL1)	Homo sapiens	GO:0000978~RNA polymerase II core promoter proximal region sequence-specific DNA binding,GO:0000982~transcription factor activity, RNA polymerase II core promoter proximal region sequence-specific binding,GO:0001077~transcriptional activator activity, RNA polymerase II core promoter proximal region sequence-specific binding,GO:0003677~DNA binding,GO:0003700~transcription factor activity, sequence-specific DNA binding,GO:0005515~protein binding,
FOSL2	FOS like 2, AP-1 transcription factor subunit(FOSL2)	Homo sapiens	GO:0000978~RNA polymerase II core promoter proximal region sequence-specific DNA binding,GO:0000982~transcription factor activity, RNA polymerase II core promoter proximal region sequence-specific binding,GO:0003682~chromatin binding,GO:0003700~transcription factor activity, sequence-specific DNA binding,GO:0005515~protein binding,GO:0043565~sequence-specific DNA binding,
FPGT-TNN	FPGT-TNNI3K readthrough(FPGT-TNNI3K)	Homo sapiens	GO:0004672~protein kinase activity,GO:0004674~protein serine/threonine kinase activity,GO:0005524~ATP binding,GO:0008022~protein C-terminus binding,GO:0031013~troponin I binding,GO:0046872~metal ion binding,
FYCO1	FYVE and coiled-coil domain containing 1(FYCO1)	Homo sapiens	GO:0005515~protein binding,GO:0046872~metal ion binding,
FANCD2	Fanconi anemia complementation group D2(FANCD2)	Homo sapiens	GO:0005515~protein binding,GO:0070182~DNA polymerase binding,
FOSB	FosB proto-oncogene, AP-1 transcription factor subunit(FOSB)	Homo sapiens	GO:0000978~RNA polymerase II core promoter proximal region sequence-specific DNA binding,GO:0000982~transcription factor activity, RNA polymerase II core promoter proximal region sequence-specific binding,GO:0001077~transcriptional activator activity, RNA polymerase II core promoter proximal region sequence-specific binding,GO:0003677~DNA binding,GO:0003700~transcription factor activity, sequence-specific DNA binding,GO:0008134~transcription factor binding,GO:0043565~sequence-specific DNA binding,
GNAZ	G protein subunit alpha z(GNAZ)	Homo sapiens	GO:0003924~GTPase activity,GO:0004871~signal transducer activity,GO:0005057~receptor signaling protein activity,GO:0005525~GTP binding,GO:0031683~G-protein beta/gamma-subunit complex binding,GO:0031821~G-protein coupled serotonin receptor binding,GO:0046872~metal ion binding,
GNB3	G protein subunit beta 3(GNB3)	Homo sapiens	GO:0003924~GTPase activity,GO:0004871~signal transducer activity,GO:0005515~protein binding,GO:0030507~spectrin binding,GO:0051020~GTPase binding,
GNG7	G protein subunit gamma 7(GNG7)	Homo sapiens	GO:0004871~signal transducer activity,
GPBAR1	G protein-coupled bile acid receptor 1(GPBAR1)	Homo sapiens	GO:0038181~bile acid receptor activity,GO:0038182~G-protein coupled bile acid receptor activity,
GPR143	G protein-coupled receptor 143(GPR143)	Homo sapiens	GO:0004930~G-protein coupled receptor activity,GO:0005515~protein binding,GO:0035240~dopamine binding,GO:0035643~L-DOPA receptor activity,GO:0072544~L-DOPA binding,GO:0072545~tyrosine binding,
GPR153	G protein-coupled receptor 153(GPR153)	Homo sapiens	GO:0004930~G-protein coupled receptor activity,
GPR162	G protein-coupled receptor 162(GPR162)	Homo sapiens	GO:0004930~G-protein coupled receptor activity,
GPR173	G protein-coupled receptor 173(GPR173)	Homo sapiens	GO:0004930~G-protein coupled receptor activity,GO:0004968~gonadotropin-releasing hormone receptor activity,
GPR182	G protein-coupled receptor 182(GPR182)	Homo sapiens	GO:0004888~transmembrane signaling receptor activity,GO:0004930~G-protein coupled receptor activity,
GPR19	G protein-coupled receptor 19(GPR19)	Homo sapiens	GO:0004930~G-protein coupled receptor activity,
GPR68	G protein-coupled receptor 68(GPR68)	Homo sapiens	GO:0004930~G-protein coupled receptor activity,
GPR82	G protein-coupled receptor 82(GPR82)	Homo sapiens	GO:0004930~G-protein coupled receptor activity,
GPRC5A	G protein-coupled receptor class C group 5 member A(GPRC5A)	Homo sapiens	GO:0004930~G-protein coupled receptor activity,GO:0005515~protein binding,GO:0098641~cadherin binding involved in cell-cell adhesion,
GRK3	G protein-coupled receptor kinase 3(GRK3)	Homo sapiens	GO:0004672~protein kinase activity,GO:0004703~G-protein coupled receptor kinase activity,GO:0005524~ATP binding,GO:0047696~beta-adrenergic receptor kinase activity,

GRK7	G protein-coupled receptor kinase 7(GRK7)	Homo sapiens	GO:0004703~G-protein coupled receptor kinase activity,GO:0005524~ATP binding,GO:0050254~rhodopsin kinase activity,
GPATCH3	G-patch domain containing 3(GPATCH3)	Homo sapiens	GO:0003676~nucleic acid binding,
GPSM1	G-protein signaling modulator 1(GPSM1)	Homo sapiens	GO:0005092~GDP-dissociation inhibitor activity,
GABARAP	GABA type A receptor associated protein like 1(GABARAPL1)	Homo sapiens	GO:0005515~protein binding,GO:0030957~Tat protein binding,GO:0031625~ubiquitin protein ligase binding,GO:0048487~beta-tubulin binding,GO:0050811~GABA receptor binding,
GATA6	GATA binding protein 6(GATA6)	Homo sapiens	GO:0000977~RNA polymerase II regulatory region sequence-specific DNA binding,GO:0000979~RNA polymerase II core promoter sequence-specific DNA binding,GO:0000981~RNA polymerase II transcription factor activity, sequence-specific DNA binding,GO:0001085~RNA polymerase II transcription factor binding,GO:0001103~RNA polymerase II repressing transcription factor binding,GO:0001228~transcriptional activator activity, RNA polymerase II transcription regulatory region sequence-specific binding,GO:0003677~DNA binding,GO:0003682~chromatin binding,GO:0003700~transcription factor activity, sequence-specific DNA binding,GO:0003705~transcription factor activity, RNA polymerase II distal enhancer sequence-specific binding,GO:0005515~protein binding,GO:0008134~transcription factor binding,GO:0008270~zinc ion binding,GO:0019901~protein kinase binding,GO:0044212~transcription regulatory region DNA binding,
GDPGP1	GDP-D-glucose phosphorylase 1(GDPGP1)	Homo sapiens	GO:0000166~nucleotide binding,GO:0005085~guanyl-nucleotide exchange factor activity,GO:0016779~nucleotidyltransferase activity,GO:0016787~hydrolase activity,GO:0080048~GDP-D-glucose phosphorylase activity,
GLI1	GLI family zinc finger 1(GLI1)	Homo sapiens	GO:0000977~RNA polymerase II regulatory region sequence-specific DNA binding,GO:0000978~RNA polymerase II core promoter proximal region sequence-specific DNA binding,GO:0003677~DNA binding,GO:0003682~chromatin binding,GO:0003705~transcription factor activity, RNA polymerase II distal enhancer sequence-specific binding,GO:0005515~protein binding,GO:0008017~microtubule binding,GO:0043565~sequence-specific DNA binding,GO:0044212~transcription regulatory region DNA binding,GO:0046872~metal ion binding,
GLIS2	GLIS family zinc finger 2(GLIS2)	Homo sapiens	GO:0000977~RNA polymerase II regulatory region sequence-specific DNA binding,GO:0001077~transcriptional activator activity, RNA polymerase II core promoter proximal region sequence-specific binding,GO:0003676~nucleic acid binding,GO:0005515~protein binding,GO:0044212~transcription regulatory region DNA binding,GO:0046872~metal ion binding,
GRIPAP1	GRIP1 associated protein 1(GRIPAP1)	Homo sapiens	GO:0005515~protein binding,
HECTD3	HECT domain E3 ubiquitin protein ligase 3(HECTD3)	Homo sapiens	GO:0004842~ubiquitin-protein transferase activity,GO:0005515~protein binding,GO:0016874~ligase activity,GO:0019905~syntaxin binding,
HID1	HID1 domain containing(HID1)	Homo sapiens	GO:0005515~protein binding,
HNF1A	HNF1 homeobox A(HNF1A)	Homo sapiens	GO:0000977~RNA polymerase II regulatory region sequence-specific DNA binding,GO:0000979~RNA polymerase II core promoter sequence-specific DNA binding,GO:0001077~transcriptional activator activity, RNA polymerase II core promoter proximal region sequence-specific binding,GO:0003677~DNA binding,GO:0003700~transcription factor activity, sequence-specific DNA binding,GO:0005515~protein binding,GO:0042803~protein homodimerization activity,GO:0044212~transcription regulatory region DNA binding,GO:0046982~protein heterodimerization activity,GO:0046983~protein dimerization activity,
HEMK1	HemK methyltransferase family member 1(HEMK1)	Homo sapiens	GO:0003677~DNA binding,GO:0005515~protein binding,GO:0008170~N-methyltransferase activity,GO:0008276~protein methyltransferase activity,
JMJD7-PL	JMJD7-PLA2G4B readthrough(JMJD7-PLA2G4B)	Homo sapiens	GO:0004622~lysophospholipase activity,GO:0004623~phospholipase A2 activity,GO:0005509~calcium ion binding,GO:0005544~calcium-dependent phospholipid binding,GO:0008970~phosphatidylcholine 1-acylhydrolase activity,GO:0047498~calcium-dependent phospholipase A2 activity,
JAK3	Janus kinase 3(JAK3)	Homo sapiens	GO:0004713~protein tyrosine kinase activity,GO:0004715~non-membrane spanning protein tyrosine kinase activity,GO:0005088~Ras guanyl-nucleotide exchange factor activity,GO:0005102~receptor binding,GO:0005515~protein binding,GO:0005524~ATP binding,GO:0019903~protein phosphatase binding,
JUN	Jun proto-oncogene, AP-1 transcription factor subunit(JUN)	Homo sapiens	GO:0000978~RNA polymerase II core promoter proximal region sequence-specific DNA binding,GO:0000980~RNA polymerase II distal enhancer sequence-specific DNA binding,GO:0000981~RNA polymerase II transcription factor activity, sequence-specific DNA binding,GO:0000982~transcription factor activity, RNA polymerase II core promoter proximal region sequence-specific binding,GO:0001077~transcriptional activator activity, RNA polymerase II core promoter proximal region sequence-specific binding,GO:0001102~RNA polymerase II activating transcription factor binding,GO:0001190~transcriptional activator activity, RNA polymerase II transcription factor binding,GO:0003677~DNA binding,GO:0003682~chromatin binding,GO:0003700~transcription factor activity, sequence-specific DNA binding,GO:0003705~transcription factor activity, RNA polymerase II distal enhancer sequence-specific binding,GO:0003713~transcription coactivator activity,GO:0005096~GTPase activator activity,GO:0005515~protein binding,GO:0008134~transcription factor binding,GO:0019899~enzyme binding,GO:0035497~cAMP response element binding,GO:0042802~identical protein binding,GO:0042803~protein homodimerization activity,GO:0043565~sequence-specific DNA binding,GO:0044212~transcription regulatory region DNA binding,GO:0044822~poly(A) RNA binding,GO:0046982~protein heterodimerization activity,GO:0070412~R-SMAD binding,GO:0071837~HMG box domain binding,
JUNB	JunB proto-oncogene, AP-1 transcription factor subunit(JUNB)	Homo sapiens	GO:0000978~RNA polymerase II core promoter proximal region sequence-specific DNA binding,GO:0000981~RNA polymerase II transcription factor activity, sequence-specific DNA binding,GO:0001077~transcriptional activator activity, RNA polymerase II core promoter proximal region sequence-specific binding,GO:0003677~DNA binding,GO:0003713~transcription coactivator activity,GO:0003714~transcription corepressor activity,GO:0003723~RNA binding,GO:0004523~RNA-DNA hybrid ribonuclease activity,GO:0005515~protein binding,GO:0008134~transcription factor binding,
KISS1R	KISS1 receptor(KISS1R)	Homo sapiens	GO:0004930~G-protein coupled receptor activity,GO:0005515~protein binding,GO:0008188~neuropeptide receptor activity,
KITLG	KIT ligand(KITLG)	Homo sapiens	GO:0005088~Ras guanyl-nucleotide exchange factor activity,GO:0005125~cytokine activity,GO:0005173~stem cell factor receptor binding,GO:0005515~protein binding,GO:0008083~growth factor activity,GO:0046934~phosphatidylinositol-4,5-bisphosphate 3-kinase activity,
KLF15	Kruppel like factor 15(KLF15)	Homo sapiens	GO:0001077~transcriptional activator activity, RNA polymerase II core promoter proximal region sequence-specific binding,GO:0005515~protein binding,GO:0044212~transcription regulatory region DNA binding,GO:0046872~metal ion binding,

KLF4	Kruppel like factor 4(KLF4)	Homo sapiens	GO:0000987~core promoter proximal region sequence-specific DNA binding,GO:0001010~transcription factor activity, sequence-specific DNA binding transcription factor recruiting,GO:0001047~core promoter binding,GO:0001077~transcriptional activator activity, RNA polymerase II core promoter proximal region sequence-specific binding,GO:0001085~RNA polymerase II transcription factor binding,GO:0001190~transcriptional activator activity, RNA polymerase II transcription factor binding,GO:0001221~transcription cofactor binding,GO:0003676~nucleic acid binding,GO:0003700~transcription factor activity, sequence-specific DNA binding,GO:0005515~protein binding,GO:0008013~beta-catenin binding,GO:0008270~zinc ion binding,GO:0035014~phosphatidylinositol 3-kinase regulator activity,GO:0042826~histone deacetylase binding,GO:0044212~transcription regulatory region DNA binding,GO:0046872~metal ion binding,
KLF5	Kruppel like factor 5(KLF5)	Homo sapiens	GO:0000978~RNA polymerase II core promoter proximal region sequence-specific DNA binding,GO:0001077~transcriptional activator activity, RNA polymerase II core promoter proximal region sequence-specific binding,GO:0003700~transcription factor activity, sequence-specific DNA binding,GO:0005515~protein binding,GO:0008134~transcription factor binding,GO:0043565~sequence-specific DNA binding,GO:0046872~metal ion binding,
LRP4	LDL receptor related protein 4(LRP4)	Homo sapiens	GO:0005509~calcium ion binding,GO:0005515~protein binding,GO:0030971~receptor tyrosine kinase binding,GO:0034185~apolipoprotein binding,GO:0042803~protein homodimerization activity,GO:0097110~scaffold protein binding,
LRP8	LDL receptor related protein 8(LRP8)	Homo sapiens	GO:0004888~transmembrane signaling receptor activity,GO:0005041~low-density lipoprotein receptor activity,GO:0005509~calcium ion binding,GO:0005515~protein binding,GO:0008035~high-density lipoprotein particle binding,GO:0019894~kinesin binding,GO:0030229~very-low-density lipoprotein particle receptor activity,GO:0034185~apolipoprotein binding,GO:0038025~reelin receptor activity,
LFNG	LFNG O-fucosylpeptide 3-beta-N-acetylglucosaminyltransferase(LFNG)	Homo sapiens	GO:0016757~transferase activity, transferring glycosyl groups,GO:0033829~O-fucosylpeptide 3-beta-N-acetylglucosaminyltransferase activity,GO:0046872~metal ion binding,
LDB3	LIM domain binding 3(LDB3)	Homo sapiens	GO:0005080~protein kinase C binding,GO:0005515~protein binding,GO:0008092~cytoskeletal protein binding,GO:0008270~zinc ion binding,GO:0051371~muscle alpha-actinin binding,
LRP2BP	LRP2 binding protein(LRP2BP)	Homo sapiens	GO:0005515~protein binding,
LYPD1	LY6/PLAUR domain containing 1(LYPD1)	Homo sapiens	GO:0030550~acetylcholine receptor inhibitor activity,GO:0033130~acetylcholine receptor binding,
MAFB	MAF bZIP transcription factor B(MAFB)	Homo sapiens	specific DNA binding,GO:0001077~transcriptional activator activity, RNA polymerase II core promoter proximal region sequence-specific binding,GO:0001228~transcriptional activator activity, RNA polymerase II transcription regulatory region sequence-specific binding,GO:0005515~protein binding,GO:0008134~transcription factor binding,
MRGPRF	MAS related GPR family member F(MRGPRF)	Homo sapiens	GO:0004930~G-protein coupled receptor activity,
MXD1	MAX dimerization protein 1(MXD1)	Homo sapiens	GO:0000978~RNA polymerase II core promoter proximal region sequence-specific DNA binding,GO:0001078~transcriptional repressor activity, RNA polymerase II core promoter proximal region sequence-specific binding,GO:0003700~transcription factor activity, sequence-specific DNA binding,GO:0003712~transcription cofactor activity,GO:0003714~transcription corepressor activity,GO:0005515~protein binding,GO:0046983~protein dimerization activity,
MXD3	MAX dimerization protein 3(MXD3)	Homo sapiens	GO:0003677~DNA binding,GO:0005515~protein binding,GO:0046983~protein dimerization activity,
MCF2L	MCF.2 cell line derived transforming sequence like(MCF2L)	Homo sapiens	GO:0005085~guanyl-nucleotide exchange factor activity,GO:0005089~Rho guanyl-nucleotide exchange factor activity,GO:0035091~phosphatidylinositol binding,
MICALL2	MICAL like 2(MICALL2)	Homo sapiens	GO:0005515~protein binding,GO:0008270~zinc ion binding,GO:0017137~Rab GTPase binding,GO:0031005~filamin binding,GO:0042805~actinin binding,GO:0051015~actin filament binding,
MLLT6	MLLT6, PHD finger domain containing(MLLT6)	Homo sapiens	GO:0005515~protein binding,GO:0008270~zinc ion binding,
MLXIPL	MLX interacting protein like(MLXIPL)	Homo sapiens	GO:0000978~RNA polymerase II core promoter proximal region sequence-specific DNA binding,GO:0001078~transcriptional repressor activity, RNA polymerase II core promoter proximal region sequence-specific binding,GO:0003677~DNA binding,GO:0003700~transcription factor activity, sequence-specific DNA binding,GO:0008134~transcription factor binding,GO:0035538~carbohydrate response element binding,GO:0042803~protein homodimerization activity,GO:0046982~protein heterodimerization activity,
MX1	MX dynamin like GTPase 1(MX1)	Homo sapiens	GO:0003924~GTPase activity,GO:0005515~protein binding,GO:0005525~GTP binding,GO:0008017~microtubule binding,
MYBBP1A	MYB binding protein 1a(MYBBP1A)	Homo sapiens	GO:0001047~core promoter binding,GO:0003677~DNA binding,GO:0003714~transcription corepressor activity,GO:0003887~DNA-directed DNA polymerase activity,GO:0005515~protein binding,GO:0008134~transcription factor binding,GO:0043565~sequence-specific DNA binding,GO:0044822~poly(A) RNA binding,
MAATS1	MYCBP associated and testis expressed 1(MAATS1)	Homo sapiens	GO:0005515~protein binding,
MB21D2	Mab-21 domain containing 2(MB21D2)	Homo sapiens	GO:0032403~protein complex binding,GO:0098641~cadherin binding involved in cell-cell adhesion,
MKS1	Meckel syndrome, type 1(MKS1)	Homo sapiens	GO:0005515~protein binding,
MYPOP	Myb related transcription factor, partner of profilin(MYPOP)	Homo sapiens	GO:0000978~RNA polymerase II core promoter proximal region sequence-specific DNA binding,GO:0001078~transcriptional repressor activity, RNA polymerase II core promoter proximal region sequence-specific binding,GO:0042803~protein homodimerization activity,
MDFI	MyoD family inhibitor(MDFI)	Homo sapiens	GO:0005515~protein binding,GO:0008134~transcription factor binding,GO:0042802~identical protein binding,
NAALAD2	N-acetylated alpha-linked acidic dipeptidase 2(NAALAD2)	Homo sapiens	GO:0004180~carboxypeptidase activity,GO:0008236~serine-type peptidase activity,GO:0008237~metallopeptidase activity,GO:0008239~dipeptidyl-peptidase activity,GO:0016805~dipeptidase activity,GO:0046872~metal ion binding,GO:0050129~N-formylglutamate deformylase activity,
NAALADL	N-acetylated alpha-linked acidic dipeptidase like 2(NAALADL2)	Homo sapiens	GO:0005515~protein binding,
NPL	N-acetylneuraminate pyruvate lyase(NPL)	Homo sapiens	GO:0005515~protein binding,GO:0008747~N-acetylneuraminate lyase activity,GO:0016829~lyase activity,GO:0042802~identical protein binding,
NAT14	N-acetyltransferase 14 (putative)(NAT14)	Homo sapiens	GO:0003677~DNA binding,GO:0008080~N-acetyltransferase activity,
NAT8L	N-acetyltransferase 8 like(NAT8L)	Homo sapiens	GO:0008080~N-acetyltransferase activity,GO:0017188~aspartate N-acetyltransferase activity,
MPG	N-methylpurine DNA glycosylase(MPG)	Homo sapiens	GO:0003677~DNA binding,GO:0003684~damaged DNA binding,GO:0003905~alkylbase DNA N-glycosylase activity,GO:0005515~protein binding,GO:0008725~DNA-3-methyladenine glycosylase activity,GO:0019104~DNA N-glycosylase activity,GO:0043916~DNA 7-methylguanine glycosylase activity,GO:0052821~DNA-7-methyladenine glycosylase activity,GO:0052822~DNA-3-methylguanine glycosylase activity,

NECAB2	N-terminal EF-hand calcium binding protein 2(NECAB2)	Homo sapiens	GO:0005509~calcium ion binding,GO:0005515~protein binding,GO:0042802~identical protein binding,
NECAB3	N-terminal EF-hand calcium binding protein 3(NECAB3)	Homo sapiens	GO:0005509~calcium ion binding,GO:0005515~protein binding,
NADSYN1	NAD synthetase 1(NADSYN1)	Homo sapiens	GO:0003952~NAD+ synthase (glutamine-hydrolyzing) activity,GO:0004359~glutaminase activity,GO:0005515~protein binding,GO:0005524~ATP binding,GO:0016810~hydrolase activity, acting on carbon-nitrogen (but not peptide) bonds,
NDUFV1	NADH:ubiquinone oxidoreductase core subunit V1(NDUFV1)	Homo sapiens	GO:0003954~NADH dehydrogenase activity,GO:0008137~NADH dehydrogenase (ubiquinone) activity,GO:0010181~FMN binding,GO:0046872~metal ion binding,GO:0051287~NAD binding,GO:0051539~4 iron, 4 sulfur cluster binding,
NOX5	NADPH oxidase 5(NOX5)	Homo sapiens	GO:0005509~calcium ion binding,GO:0015252~hydrogen ion channel activity,GO:0016175~superoxide-generating NADPH oxidase activity,GO:0020037~heme binding,GO:0050660~flavin adenine dinucleotide binding,GO:0050661~NADP binding,
NOXA1	NADPH oxidase activator 1(NOXA1)	Homo sapiens	GO:0005515~protein binding,GO:0016176~superoxide-generating NADPH oxidase activator activity,GO:0017124~SH3 domain binding,GO:0019899~enzyme binding,GO:0048365~Rac GTPase binding,
NIM1K	NIM1 serine/threonine protein kinase(NIM1K)	Homo sapiens	GO:0000287~magnesium ion binding,GO:0004672~protein kinase activity,GO:0004674~protein serine/threonine kinase activity,GO:0005524~ATP binding,
NIPAL2	NIPA like domain containing 2(NIPAL2)	Homo sapiens	GO:0015095~magnesium ion transmembrane transporter activity,
NLRP1	NLR family pyrin domain containing 1(NLRP1)	Homo sapiens	GO:0005515~protein binding,GO:0005524~ATP binding,GO:0008656~cysteine-type endopeptidase activator activity involved in apoptotic process,GO:0019899~enzyme binding,GO:0019904~protein domain specific binding,GO:0097110~scaffold protein binding,
NME3	NME/NM23 nucleoside diphosphate kinase 3(NME3)	Homo sapiens	GO:0004550~nucleoside diphosphate kinase activity,GO:0005515~protein binding,GO:0005524~ATP binding,GO:0046872~metal ion binding,
NUAK1	NUAK family kinase 1(NUAK1)	Homo sapiens	GO:0002039~p53 binding,GO:0004672~protein kinase activity,GO:0004674~protein serine/threonine kinase activity,GO:0005515~protein binding,GO:0005524~ATP binding,GO:0046872~metal ion binding,
NUAK2	NUAK family kinase 2(NUAK2)	Homo sapiens	GO:0000287~magnesium ion binding,GO:0004674~protein serine/threonine kinase activity,GO:0005515~protein binding,GO:0005524~ATP binding,
NYNRIN	NYN domain and retroviral integrase containing(NYNRIN)	Homo sapiens	GO:0003676~nucleic acid binding,
OCA2	OCA2 melanosomal transmembrane protein(OCA2)	Homo sapiens	GO:0005215~transporter activity,GO:0005302~L-tyrosine transmembrane transporter activity,GO:0005515~protein binding,
ORAI3	ORAI calcium release-activated calcium modulator 3(ORAI3)	Homo sapiens	GO:0005515~protein binding,GO:0015279~store-operated calcium channel activity,
OTUD1	OTU deubiquitinase 1(OTUD1)	Homo sapiens	GO:0004843~thiol-dependent ubiquitin-specific protease activity,
OIP5	Opa interacting protein 5(OIP5)	Homo sapiens	GO:0005515~protein binding,GO:0046872~metal ion binding,
PATL1	PAT1 homolog 1, processing body mRNA decay factor(PATL1)	Homo sapiens	GO:0003723~RNA binding,GO:0005515~protein binding,GO:0008266~poly(U) RNA binding,GO:0034046~poly(G) binding,GO:0044822~poly(A) RNA binding,
PDZK1	PDZ domain containing 1(PDZK1)	Homo sapiens	GO:0005124~scavenger receptor binding,GO:0005215~transporter activity,GO:0005515~protein binding,GO:0030165~PDZ domain binding,GO:0032403~protein complex binding,
PDZD3	PDZ domain containing 3(PDZD3)	Homo sapiens	GO:0005515~protein binding,GO:0008022~protein C-terminus binding,GO:0008200~ion channel inhibitor activity,GO:0030251~guanylate cyclase inhibitor activity,
PDZD7	PDZ domain containing 7(PDZD7)	Homo sapiens	GO:0005515~protein binding,GO:0042803~protein homodimerization activity,GO:0046982~protein heterodimerization activity,
PHF7	PHD finger protein 7(PHF7)	Homo sapiens	GO:0005515~protein binding,GO:0008270~zinc ion binding,
PITPNM3	PITPNM family member 3(PITPNM3)	Homo sapiens	GO:0005509~calcium ion binding,GO:0005515~protein binding,GO:0008289~lipid binding,GO:0008526~phosphatidylinositol transporter activity,GO:0030971~receptor tyrosine kinase binding,GO:0046872~metal ion binding,
POMZP3	POM121 and ZP3 fusion(POMZP3)	Homo sapiens	GO:0032190~acrosin binding,
POU3F1	POU class 3 homeobox 1(POU3F1)	Homo sapiens	GO:0001105~RNA polymerase II transcription coactivator activity,GO:0003700~transcription factor activity, sequence-specific DNA binding,GO:0043565~sequence-specific DNA binding,
PPFIA3	PTPRF interacting protein alpha 3(PPFIA3)	Homo sapiens	GO:0005515~protein binding,
PXDC1	PX domain containing 1(PXDC1)	Homo sapiens	GO:0035091~phosphatidylinositol binding,
PYCARD	PYD and CARD domain containing(PYCARD)	Homo sapiens	GO:0002020~protease binding,GO:0005138~interleukin-6 receptor binding,GO:0005515~protein binding,GO:0005523~tropomyosin binding,GO:0008656~cysteine-type endopeptidase activator activity involved in apoptotic process,GO:0017024~myosin I binding,GO:0019899~enzyme binding,GO:0032090~Pyrin domain binding,GO:0042802~identical protein binding,GO:0042803~protein homodimerization activity,GO:0070700~BMP receptor binding,GO:0097153~cysteine-type endopeptidase activity involved in apoptotic process,
PIM1	Pim-1 proto-oncogene, serine/threonine kinase(PIM1)	Homo sapiens	GO:0004674~protein serine/threonine kinase activity,GO:0005515~protein binding,GO:0005524~ATP binding,GO:0008134~transcription factor binding,GO:0030145~manganese ion binding,GO:0043024~ribosomal small subunit binding,
RAB30	RAB30, member RAS oncogene family(RAB30)	Homo sapiens	GO:0003924~GTPase activity,GO:0005525~GTP binding,
RAB7B	RAB7B, member RAS oncogene family(RAB7B)	Homo sapiens	GO:0005525~GTP binding,
RHNO1	RAD9-HUS1-RAD1 interacting nuclear orphan 1(RHNO1)	Homo sapiens	GO:0005515~protein binding,
RAP1GAP	RAP1 GTPase activating protein(RAP1GAP)	Homo sapiens	GO:0003924~GTPase activity,GO:0005096~GTPase activator activity,GO:0005515~protein binding,GO:0017016~Ras GTPase binding,GO:0030695~GTPase regulator activity,GO:0042803~protein homodimerization activity,
RORC	RAR related orphan receptor C(RORC)	Homo sapiens	GO:0003677~DNA binding,GO:0003700~transcription factor activity, sequence-specific DNA binding,GO:0003707~steroid hormone receptor activity,GO:0004879~RNA polymerase II transcription factor activity, ligand-activated sequence-specific DNA binding,GO:0005515~protein binding,GO:0008142~oxysterol binding,GO:0008270~zinc ion binding,GO:0043565~sequence-specific DNA binding,GO:0098531~transcription factor activity, direct ligand regulated sequence-specific DNA binding,
RASGRP2	RAS guanyl releasing protein 2(RASGRP2)	Homo sapiens	GO:0005085~guanyl-nucleotide exchange factor activity,GO:0005509~calcium ion binding,GO:0008289~lipid binding,GO:0019992~diacylglycerol binding,
RASL11A	RAS like family 11 member A(RASL11A)	Homo sapiens	GO:0005525~GTP binding,
RASA4	RAS p21 protein activator 4(RASA4)	Homo sapiens	GO:0005096~GTPase activator activity,GO:0046872~metal ion binding,
RASAL1	RAS protein activator like 1(RASAL1)	Homo sapiens	GO:0005096~GTPase activator activity,GO:0005543~phospholipid binding,GO:0046872~metal ion binding,
RBBP9	RB binding protein 9, serine hydrolase(RBBP9)	Homo sapiens	GO:0005515~protein binding,GO:0016787~hydrolase activity,
RCOR2	REST corepressor 2(RCOR2)	Homo sapiens	GO:0003677~DNA binding,GO:0003700~transcription factor activity, sequence-specific DNA binding,GO:0003714~transcription corepressor activity,GO:0008134~transcription factor binding,GO:0044212~transcription regulatory region DNA binding,
RFNG	RFNG O-fucosylpeptide 3-beta-N-acetylglucosaminyltransferase(RFNG)	Homo sapiens	GO:0016757~transferase activity, transferring glycosyl groups,GO:0033829~O-fucosylpeptide 3-beta-N-acetylglucosaminyltransferase activity,GO:0046872~metal ion binding,

RIC8A	RIC8 guanine nucleotide exchange factor A(RIC8A)	Homo sapiens	GO:0001965~G-protein alpha-subunit binding,GO:0005085~guanyl-nucleotide exchange factor activity,GO:0005096~GTPase activator activity,GO:0005515~protein binding,
RBM22	RNA binding motif protein 22(RBM22)	Homo sapiens	GO:0000166~nucleotide binding,GO:0005487~nucleocytoplasmic transporter activity,GO:0005515~protein binding,GO:0017070~U6 snRNA binding,GO:0036002~pre-mRNA binding,GO:0044822~poly(A) RNA binding,GO:0046872~metal ion binding,GO:0048306~calcium-dependent protein binding,
RBM48	RNA binding motif protein 48(RBM48)	Homo sapiens	GO:0000166~nucleotide binding,GO:0003723~RNA binding,
POLR3GL	RNA polymerase III subunit G like(POLR3GL)	Homo sapiens	GO:0001056~RNA polymerase III activity,GO:0005515~protein binding,
RNF103-C	RNF103-CHMP3 readthrough(RNF103-CHMP3)	Homo sapiens	GO:0031210~phosphatidylcholine binding,GO:0042803~protein homodimerization activity,GO:1990381~ubiquitin-specific protease binding,
RWDD2B	RWD domain containing 2B(RWDD2B)	Homo sapiens	GO:0005515~protein binding,
RADIL	Rap associating with DIL domain(RADIL)	Homo sapiens	GO:0005515~protein binding,
RAPGEFL1	Rap guanine nucleotide exchange factor like 1(RAPGEFL1)	Homo sapiens	GO:0005057~receptor signaling protein activity,GO:0005085~guanyl-nucleotide exchange factor activity,
RINL	Ras and Rab interactor like(RINL)	Homo sapiens	GO:0005085~guanyl-nucleotide exchange factor activity,GO:0005096~GTPase activator activity,
RASSF4	Ras association domain family member 4(RASSF4)	Homo sapiens	GO:0005515~protein binding,
RRAGC	Ras related GTP binding C(RRAGC)	Homo sapiens	GO:0000287~magnesium ion binding,GO:0003924~GTPase activity,GO:0005515~protein binding,GO:0005525~GTP binding,GO:0019003~GDP binding,GO:0046982~protein heterodimerization activity,
RASGEF1C	RasGEF domain family member 1C(RASGEF1C)	Homo sapiens	GO:0005085~guanyl-nucleotide exchange factor activity,
RECQL5	RecQ like helicase 5(RECQL5)	Homo sapiens	GO:0000993~RNA polymerase II core binding,GO:0003676~nucleic acid binding,GO:0003677~DNA binding,GO:0003678~DNA helicase activity,GO:0005524~ATP binding,GO:0008026~ATP-dependent helicase activity,GO:0009378~four-way junction helicase activity,GO:0043140~ATP-dependent 3'-5' DNA helicase activity,
RMI2	RecQ mediated genome instability 2(RMI2)	Homo sapiens	GO:0003677~DNA binding,
ARHGDIG	Rho GDP dissociation inhibitor gamma(ARHGDIG)	Homo sapiens	GO:0005094~Rho GDP-dissociation inhibitor activity,GO:0005096~GTPase activator activity,GO:0005515~protein binding,
ARHGAP3	Rho GTPase activating protein 31(ARHGAP31)	Homo sapiens	GO:0005096~GTPase activator activity,GO:0017124~SH3 domain binding,
ARHGAP3	Rho GTPase activating protein 33(ARHGAP33)	Homo sapiens	GO:0005096~GTPase activator activity,GO:0005515~protein binding,GO:0035091~phosphatidylinositol binding,
RND1	Rho family GTPase 1(RND1)	Homo sapiens	GO:0003924~GTPase activity,GO:0005102~receptor binding,GO:0005515~protein binding,GO:0005525~GTP binding,
RND3	Rho family GTPase 3(RND3)	Homo sapiens	GO:0003924~GTPase activity,GO:0005515~protein binding,GO:0005525~GTP binding,
ARHGEF1	Rho guanine nucleotide exchange factor 1(ARHGEF1)	Homo sapiens	GO:0005089~Rho guanyl-nucleotide exchange factor activity,GO:0005096~GTPase activator activity,GO:0005515~protein binding,GO:0044822~poly(A) RNA binding,
ARHGEF2	Rho guanine nucleotide exchange factor 25(ARHGEF25)	Homo sapiens	GO:0005089~Rho guanyl-nucleotide exchange factor activity,
SARNP	SAP domain containing ribonucleoprotein(SARNP)	Homo sapiens	GO:0003677~DNA binding,GO:0005515~protein binding,GO:0044822~poly(A) RNA binding,
SBDS	SBDS ribosome assembly guanine nucleotide exchange factor(SBDS)	Homo sapiens	GO:0005515~protein binding,GO:0008017~microtubule binding,GO:0019843~rRNA binding,GO:0043022~ribosome binding,GO:0044822~poly(A) RNA binding,
SSPO	SCO-spondin(SSPO)	Homo sapiens	GO:0030414~peptidase inhibitor activity,
SERTAD1	SERTA domain containing 1(SERTAD1)	Homo sapiens	GO:0005515~protein binding,
SH3PXD2A	SH3 and PX domains 2A(SH3PXD2A)	Homo sapiens	GO:0005515~protein binding,GO:0016176~superoxide-generating NADPH oxidase activator activity,GO:0035091~phosphatidylinositol binding,
SH3PXD2B	SH3 and PX domains 2B(SH3PXD2B)	Homo sapiens	GO:0005515~protein binding,GO:0010314~phosphatidylinositol-5-phosphate binding,GO:0016176~superoxide-generating NADPH oxidase activator activity,GO:0032266~phosphatidylinositol-3-phosphate binding,GO:0035091~phosphatidylinositol binding,GO:0042169~SH2 domain binding,GO:0070273~phosphatidylinositol-4-phosphate binding,GO:0080025~phosphatidylinositol-3,5-bisphosphate binding,
SHANK2	SH3 and multiple ankyrin repeat domains 2(SHANK2)	Homo sapiens	GO:0005515~protein binding,GO:0017124~SH3 domain binding,GO:0030160~GKAP/Homer scaffold activity,GO:0035255~ionotropic glutamate receptor binding,
SBK1	SH3 domain binding kinase 1(SBK1)	Homo sapiens	GO:0004674~protein serine/threonine kinase activity,GO:0005524~ATP binding,
SH3GL3	SH3 domain containing GRB2 like 3, endophilin A3(SH3GL3)	Homo sapiens	GO:0005515~protein binding,GO:0008289~lipid binding,GO:0042802~identical protein binding,
SH3GLB2	SH3 domain containing GRB2 like endophilin B2(SH3GLB2)	Homo sapiens	GO:0005515~protein binding,GO:0042802~identical protein binding,GO:0098641~cadherin binding involved in cell-cell adhesion,
SLC9A3R2	SLC9A3 regulator 2(SLC9A3R2)	Homo sapiens	GO:0005102~receptor binding,GO:0005515~protein binding,GO:0008013~beta-catenin binding,GO:0008022~protein C-terminus binding,GO:0019902~phosphatase binding,GO:0032947~protein complex scaffold,GO:0098641~cadherin binding involved in cell-cell adhesion,
SMAD7	SMAD family member 7(SMAD7)	Homo sapiens	GO:0003700~transcription factor activity, sequence-specific DNA binding,GO:0005515~protein binding,GO:0005518~collagen binding,GO:0008013~beta-catenin binding,GO:0030617~transforming growth factor beta receptor, inhibitory cytoplasmic mediator activity,GO:0031625~ubiquitin protein ligase binding,GO:0034713~type I transforming growth factor beta receptor binding,GO:0044212~transcription regulatory region DNA binding,GO:0046872~metal ion binding,GO:0048185~activin binding,GO:0070411~I-SMAD binding,
SMG7	SMG7, nonsense mediated mRNA decay factor(SMG7)	Homo sapiens	GO:0004540~ribonuclease activity,GO:0005515~protein binding,GO:0042162~telomeric DNA binding,GO:0043021~ribonucleoprotein complex binding,GO:0051721~protein phosphatase 2A binding,GO:0070034~telomerase RNA binding,
SP110	SP110 nuclear body protein(SP110)	Homo sapiens	GO:0003677~DNA binding,GO:0004871~signal transducer activity,GO:0008270~zinc ion binding,
SMOC2	SPARC related modular calcium binding 2(SMOC2)	Homo sapiens	GO:0005509~calcium ion binding,GO:0008201~heparin binding,
SPTY2D1	SPT2 chromatin protein domain containing 1(SPTY2D1)	Homo sapiens	GO:0001042~RNA polymerase I core binding,GO:0003677~DNA binding,GO:0042393~histone binding,
SOX12	SRY-box 12(SOX12)	Homo sapiens	GO:0000976~transcription regulatory region sequence-specific DNA binding,GO:0001077~transcriptional activator activity, RNA polymerase II core promoter proximal region sequence-specific binding,GO:0001105~RNA polymerase II transcription coactivator activity,GO:0003677~DNA binding,
ST6GALN	ST6 N-acetylgalactosaminide alpha-2,6-sialyltransferase 3(ST6GALNAC3)	Homo sapiens	GO:0001665~alpha-N-acetylgalactosaminide alpha-2,6-sialyltransferase activity,GO:0008373~sialyltransferase activity,
STEAP2	STEAP2 metalloreductase(STEAP2)	Homo sapiens	GO:0005215~transporter activity,GO:0008823~cupric reductase activity,GO:0046872~metal ion binding,GO:0052851~ferric-chelate reductase (NADPH) activity,
STEAP3	STEAP3 metalloreductase(STEAP3)	Homo sapiens	GO:0005515~protein binding,GO:0008823~cupric reductase activity,GO:0016723~oxidoreductase activity, oxidizing metal ions, NAD or NADP as acceptor,GO:0046872~metal ion binding,GO:0052851~ferric-chelate reductase (NADPH) activity,

SAP18	Sin3A associated protein 18(SAP18)	Homo sapiens	GO:0003714~transcription corepressor activity,GO:0004407~histone deacetylase activity,GO:0005515~protein binding,GO:0044822~poly(A) RNA binding,
SNIP1	Smad nuclear interacting protein 1(SNIP1)	Homo sapiens	GO:0003729~mRNA binding,GO:0005515~protein binding,GO:0044822~poly(A) RNA binding,
SMCR8	Smith-Magenis syndrome chromosome region, candidate 8(SMCR8)	Homo sapiens	GO:0005515~protein binding,
SLA2	Src like adaptor 2(SLA2)	Homo sapiens	GO:0005070~SH3/SH2 adaptor activity,GO:0005515~protein binding,GO:0047485~protein N-terminus binding,
STARD8	StAR related lipid transfer domain containing 8(STARD8)	Homo sapiens	GO:0005096~GTPase activator activity,GO:0008289~lipid binding,
TBX15	T-box 15(TBX15)	Homo sapiens	GO:0000978~RNA polymerase II core promoter proximal region sequence-specific DNA binding,GO:0001078~transcriptional repressor activity, RNA polymerase II core promoter proximal region sequence-specific binding,GO:0001106~RNA polymerase II transcription corepressor activity,GO:0003700~transcription factor activity, sequence-specific DNA binding,GO:0005515~protein binding,GO:0042803~protein homodimerization activity,GO:0046982~protein heterodimerization activity,
TBX19	T-box 19(TBX19)	Homo sapiens	GO:0001158~enhancer sequence-specific DNA binding,GO:0003677~DNA binding,GO:0003700~transcription factor activity, sequence-specific DNA binding,
TBR1	T-box, brain 1(TBR1)	Homo sapiens	GO:0000979~RNA polymerase II core promoter sequence-specific DNA binding,GO:0003677~DNA binding,GO:0003700~transcription factor activity, sequence-specific DNA binding,
TLX2	T-cell leukemia homeobox 2(TLX2)	Homo sapiens	GO:0003677~DNA binding,GO:0043565~sequence-specific DNA binding,
TARBP1	TAR (HIV-1) RNA binding protein 1(TARBP1)	Homo sapiens	GO:0003723~RNA binding,GO:0016423~tRNA (guanine) methyltransferase activity,
TBC1D10(	TBC1 domain family member 10C(TBC1D10C)	Homo sapiens	GO:0005096~GTPase activator activity,GO:0005515~protein binding,GO:0017137~Rab GTPase binding,
TBC1D16	TBC1 domain family member 16(TBC1D16)	Homo sapiens	GO:0005096~GTPase activator activity,GO:0005515~protein binding,GO:0017137~Rab GTPase binding,
TEAD1	TEA domain transcription factor 1(TEAD1)	Homo sapiens	GO:0000978~RNA polymerase II core promoter proximal region sequence-specific DNA binding,GO:0000982~transcription factor activity, RNA polymerase II core promoter proximal region sequence-specific binding,GO:0001077~transcriptional activator activity, RNA polymerase II core promoter proximal region sequence-specific binding,GO:0001159~core promoter proximal region DNA binding,GO:0003677~DNA binding,GO:0003700~transcription factor activity, sequence-specific DNA binding,GO:0005515~protein binding,
TAB1	TGF-beta activated kinase 1 (MAP3K7) binding protein 1(TAB1)	Homo sapiens	GO:0004722~protein serine/threonine phosphatase activity,GO:0005515~protein binding,GO:0008047~enzyme activator activity,GO:0016301~kinase activity,GO:0019209~kinase activator activity,GO:0032403~protein complex binding,GO:0048273~mitogen-activated protein kinase p38 binding,
TRAF1	TNF receptor associated factor 1(TRAF1)	Homo sapiens	GO:0005164~tumor necrosis factor receptor binding,GO:0005515~protein binding,GO:0008270~zinc ion binding,GO:0031625~ubiquitin protein ligase binding,GO:0031996~thioesterase binding,GO:0042802~identical protein binding,
TRAP1	TNF receptor associated protein 1(TRAP1)	Homo sapiens	GO:0005164~tumor necrosis factor receptor binding,GO:0005515~protein binding,GO:0005524~ATP binding,GO:0019901~protein kinase binding,GO:0044822~poly(A) RNA binding,GO:0051082~unfolded protein binding,
TNFRSF25	TNF receptor superfamily member 25(TNFRSF25)	Homo sapiens	GO:0004872~receptor activity,GO:0005031~tumor necrosis factor-activated receptor activity,
TSPOAP1	TSPO associated protein 1(TSPOAP1)	Homo sapiens	GO:0005515~protein binding,GO:0030156~benzodiazepine receptor binding,
UFL1	UFM1 specific ligase 1(UFL1)	Homo sapiens	GO:0005515~protein binding,GO:0016874~ligase activity,GO:0071568~UFM1 transferase activity,
WHAMM	WAS protein homolog associated with actin, golgi membranes and microtubules(WHAMM)	Homo sapiens	GO:0003779~actin binding,GO:0008017~microtubule binding,GO:0017049~GTP-Rho binding,GO:0071933~Arp2/3 complex binding,
WDR49	WD repeat domain 49(WDR49)	Homo sapiens	GO:0005509~calcium ion binding,
WDFY4	WDFY family member 4(WDFY4)	Homo sapiens	GO:0005543~phospholipid binding,
WNK2	WNK lysine deficient protein kinase 2(WNK2)	Homo sapiens	GO:0004674~protein serine/threonine kinase activity,GO:0005524~ATP binding,
WWC3	WWC family member 3(WWC3)	Homo sapiens	GO:0019900~kinase binding,GO:0032947~protein complex scaffold,
WNT10B	Wnt family member 10B(WNT10B)	Homo sapiens	GO:0005109~frizzled binding,GO:0048018~receptor agonist activity,
WNT2B	Wnt family member 2B(WNT2B)	Homo sapiens	GO:0005109~frizzled binding,
WNT4	Wnt family member 4(WNT4)	Homo sapiens	GO:0003714~transcription corepressor activity,GO:0005109~frizzled binding,GO:0048018~receptor agonist activity,
XRCC1	X-ray repair cross complementing 1(XRCC1)	Homo sapiens	GO:0003684~damaged DNA binding,GO:0003909~DNA ligase activity,GO:0005515~protein binding,GO:0019899~enzyme binding,
YKT6	YKT6 v-SNARE homolog (S. cerevisiae)(YKT6)	Homo sapiens	GO:0000149~SNARE binding,GO:0005484~SNAP receptor activity,GO:0019706~protein-cysteine S-palmitoyltransferase activity,GO:0098641~cadherin binding involved in cell-cell adhesion,
YOD1	YOD1 deubiquitinase(YOD1)	Homo sapiens	GO:0004843~thiol-dependent ubiquitin-specific protease activity,GO:0005515~protein binding,GO:0031625~ubiquitin protein ligase binding,GO:0046872~metal ion binding,GO:0061578~Lys63-specific deubiquitinase activity,GO:1904265~ubiquitin-specific protease activity involved in negative regulation of retrograde protein transport, ER to cytosol,GO:1990380~Lys48-specific deubiquitinase activity,
YJEFN3	YjeF N-terminal domain containing 3(YJEFN3)	Homo sapiens	GO:0052856~NADHX epimerase activity,GO:0052857~NADPHX epimerase activity,
ZFP37	ZFP37 zinc finger protein(ZFP37)	Homo sapiens	GO:0003677~DNA binding,GO:0003700~transcription factor activity, sequence-specific DNA binding,GO:0008270~zinc ion binding,
ZNF670-Z	ZNF670-ZNF695 readthrough (NMD candidate)(ZNF670-ZNF695)	Homo sapiens	GO:0003676~nucleic acid binding,
ABHD12B	abhydrolase domain containing 12B(ABHD12B)	Homo sapiens	GO:0016787~hydrolase activity,
ABHD4	abhydrolase domain containing 4(ABHD4)	Homo sapiens	GO:0016787~hydrolase activity,GO:0052689~carboxylic ester hydrolase activity,
ACACB	acetyl-CoA carboxylase beta(ACACB)	Homo sapiens	GO:0003989~acetyl-CoA carboxylase activity,GO:0004075~biotin carboxylase activity,GO:0005515~protein binding,GO:0005524~ATP binding,GO:0009374~biotin binding,GO:0046872~metal ion binding,
ACP6	acid phosphatase 6, lysophosphatidic(ACP6)	Homo sapiens	GO:0003993~acid phosphatase activity,GO:0052642~lysophosphatidic acid phosphatase activity,
ASIC1	acid sensing ion channel subunit 1(ASIC1)	Homo sapiens	GO:0005272~sodium channel activity,GO:0005515~protein binding,GO:0015280~ligand-gated sodium channel activity,GO:0022839~ion gated channel activity,GO:0044736~acid-sensing ion channel activity,
ACTL7B	actin like 7B(ACTL7B)	Homo sapiens	GO:0005200~structural constituent of cytoskeleton,
ACTC1	actin, alpha, cardiac muscle 1(ACTC1)	Homo sapiens	GO:0005524~ATP binding,GO:0016887~ATPase activity,GO:0017022~myosin binding,
ACTBL2	actin, beta like 2(ACTBL2)	Homo sapiens	GO:0005515~protein binding,GO:0005524~ATP binding,
ARPIN	actin-related protein 2/3 complex inhibitor(ARPIN)	Homo sapiens	GO:0005515~protein binding,

ATF3	activating transcription factor 3(ATF3)	Homo sapiens	GO:0000976~transcription regulatory region sequence-specific DNA binding,GO:0000977~RNA polymerase II regulatory region sequence-specific DNA binding,GO:0000978~RNA polymerase II core promoter proximal region sequence-specific DNA binding,GO:0000982~transcription factor activity, RNA polymerase II core promoter proximal region sequence-specific binding,GO:0001078~transcriptional repressor activity, RNA polymerase II core promoter proximal region sequence-specific binding,GO:0001228~transcriptional activator activity, RNA polymerase II transcription regulatory region sequence-specific binding,GO:0003700~transcription factor activity, sequence-specific DNA binding,GO:0003714~transcription corepressor activity,GO:0005515~protein binding,GO:0042802~identical protein binding,GO:0042803~protein homodimerization activity,GO:0043565~sequence-specific DNA binding,GO:0044212~transcription regulatory region DNA binding,GO:0046982~protein heterodimerization activity,
ACADS	acyl-CoA dehydrogenase, C-2 to C-3 short chain(ACADS)	Homo sapiens	GO:0000062~fatty-acyl-CoA binding,GO:0003995~acyl-CoA dehydrogenase activity,GO:0004085~butyryl-CoA dehydrogenase activity,GO:0009055~electron carrier activity,GO:0050660~flavin adenine dinucleotide binding,
ACADSB	acyl-CoA dehydrogenase, short/branched chain(ACADSB)	Homo sapiens	GO:0000062~fatty-acyl-CoA binding,GO:0003995~acyl-CoA dehydrogenase activity,GO:0009055~electron carrier activity,GO:0050660~flavin adenine dinucleotide binding,GO:0052890~oxidoreductase activity, acting on the CH-CH group of donors, with a flavin as acceptor,
ACSF3	acyl-CoA synthetase family member 3(ACSF3)	Homo sapiens	GO:0003824~catalytic activity,GO:0005524~ATP binding,GO:0016878~acid-thiol ligase activity,GO:0031957~very long-chain fatty acid-CoA ligase activity,GO:0090409~malonyl-CoA synthetase activity,
ACSL5	acyl-CoA synthetase long-chain family member 5(ACSL5)	Homo sapiens	GO:0004467~long-chain fatty acid-CoA ligase activity,GO:0005524~ATP binding,GO:0102391~decanoate--CoA ligase activity,
APEH	acylaminoacyl-peptide hydrolase(APEH)	Homo sapiens	GO:0004252~serine-type endopeptidase activity,GO:0005515~protein binding,GO:0008242~omega peptidase activity,GO:0042802~identical protein binding,GO:0044822~poly(A) RNA binding,
AP2A1	adaptor related protein complex 2 alpha 1 subunit(AP2A1)	Homo sapiens	GO:0005515~protein binding,GO:0008022~protein C-terminus binding,GO:0008565~protein transporter activity,GO:0019901~protein kinase binding,GO:0032403~protein complex binding,GO:0035615~clathrin adaptor activity,
AHCYL1	adenosylhomocysteinase like 1(AHCYL1)	Homo sapiens	GO:0003723~RNA binding,GO:0004013~adenosylhomocysteinase activity,GO:0005515~protein binding,GO:0016787~hydrolase activity,GO:0042802~identical protein binding,GO:0051287~NAD binding,
ADCY10	adenylate cyclase 10, soluble(ADCY10)	Homo sapiens	GO:0000287~magnesium ion binding,GO:0004016~adenylate cyclase activity,GO:0005524~ATP binding,GO:0030145~manganese ion binding,GO:0051117~ATPase binding,GO:0071890~bicarbonate binding,
ADCY6	adenylate cyclase 6(ADCY6)	Homo sapiens	GO:0004016~adenylate cyclase activity,GO:0005515~protein binding,GO:0005524~ATP binding,GO:0019901~protein kinase binding,GO:0046872~metal ion binding,
AK5	adenylate kinase 5(AK5)	Homo sapiens	GO:0004017~adenylate kinase activity,GO:0004550~nucleoside diphosphate kinase activity,GO:0005524~ATP binding,GO:0019205~nucleobase-containing compound kinase activity,GO:0019206~nucleoside kinase activity,
AK7	adenylate kinase 7(AK7)	Homo sapiens	GO:0004017~adenylate kinase activity,GO:0004127~cytidylate kinase activity,GO:0004550~nucleoside diphosphate kinase activity,GO:0005524~ATP binding,GO:0019205~nucleobase-containing compound kinase activity,GO:0019206~nucleoside kinase activity,
ADSSL1	adenylosuccinate synthase like 1(ADSSL1)	Homo sapiens	GO:0000287~magnesium ion binding,GO:0003924~GTPase activity,GO:0004019~adenylosuccinate synthase activity,GO:0005525~GTP binding,GO:0042301~phosphate ion binding,GO:0042803~protein homodimerization activity,GO:0051015~actin filament binding,
ADGRB1	adhesion G protein-coupled receptor B1(ADGRB1)	Homo sapiens	GO:0004930~G-protein coupled receptor activity,
ADGRD2	adhesion G protein-coupled receptor D2(ADGRD2)	Homo sapiens	GO:0004930~G-protein coupled receptor activity,
ADGRF1	adhesion G protein-coupled receptor F1(ADGRF1)	Homo sapiens	GO:0004930~G-protein coupled receptor activity,
ADGRG5	adhesion G protein-coupled receptor G5(ADGRG5)	Homo sapiens	GO:0004930~G-protein coupled receptor activity,
ADM	adrenomedullin(ADM)	Homo sapiens	GO:0005102~receptor binding,GO:0005179~hormone activity,GO:0005515~protein binding,GO:0031700~adrenomedullin receptor binding,
ASIP	agouti signaling protein(ASIP)	Homo sapiens	GO:0005102~receptor binding,GO:0031781~type 3 melanocortin receptor binding,GO:0031782~type 4 melanocortin receptor binding,
ALDH1L1	aldehyde dehydrogenase 1 family member L1(ALDH1L1)	Homo sapiens	GO:0003824~catalytic activity,GO:0004029~aldehyde dehydrogenase (NAD) activity,GO:0016155~formyltetrahydrofolate dehydrogenase activity,GO:0016491~oxidoreductase activity,GO:0016620~oxidoreductase activity, acting on the aldehyde or oxo group of donors, NAD or NADP as acceptor,GO:0016742~hydroxymethyl-, formyl- and related transferase activity,GO:0019145~aminobutyraldehyde dehydrogenase activity,GO:0047105~4-trimethylammoniobutyraldehyde dehydrogenase activity,
ALDH3B1	aldehyde dehydrogenase 3 family member B1(ALDH3B1)	Homo sapiens	GO:0004028~3-chloroallyl aldehyde dehydrogenase activity,GO:0004029~aldehyde dehydrogenase (NAD) activity,GO:0004030~aldehyde dehydrogenase [NAD(P)+] activity,GO:0005515~protein binding,GO:0016491~oxidoreductase activity,GO:0016620~oxidoreductase activity, acting on the aldehyde or oxo group of donors, NAD or NADP as acceptor,
ALDH8A1	aldehyde dehydrogenase 8 family member A1(ALDH8A1)	Homo sapiens	GO:0001758~retinal dehydrogenase activity,GO:0004029~aldehyde dehydrogenase (NAD) activity,GO:0016620~oxidoreductase activity, acting on the aldehyde or oxo group of donors, NAD or NADP as acceptor,
AKR7A2	aldo-keto reductase family 7 member A2(AKR7A2)	Homo sapiens	activity,GO:0004032~alditol:NADP+ 1-oxidoreductase activity,GO:0004033~aldo-keto reductase (NADP) activity,GO:0004448~isocitrate dehydrogenase activity,GO:0004495~mevaldate reductase activity,GO:0005515~protein binding,GO:0008875~gluconate dehydrogenase activity,GO:0009055~electron carrier activity,GO:0018451~epoxide dehydrogenase activity,GO:0018452~5-exo-hydroxycamphor dehydrogenase activity,GO:0018453~2-hydroxytetrahydrofuran dehydrogenase activity,GO:0019119~phenanthrene-9,10-epoxide hydrolase activity,GO:0019152~acetoin dehydrogenase activity,GO:0032442~phenylcoumaran benzylic ether reductase activity,GO:0032866~D-xylose:NADP reductase activity,GO:0032867~L-arabinose:NADP reductase activity,GO:0033709~D-arabinitol dehydrogenase, D-ribulose forming (NADP+) activity,GO:0033765~steroid dehydrogenase activity, acting on the CH-CH group of donors,GO:0034831~(R)-(-)-1,2,3,4-tetrahydronaphthol dehydrogenase activity,GO:0034840~3-hydroxymenthone dehydrogenase activity,GO:0035380~very long-chain-3-hydroxyacyl-CoA dehydrogenase activity,GO:0035410~dihydrotestosterone 17-beta-dehydrogenase activity,GO:0043713~(R)-2-hydroxyisocaproate dehydrogenase activity,GO:0044103~L-arabinose 1-dehydrogenase (NADP+) activity,GO:0044105~L-xylulose reductase (NAD+) activity,GO:0048258~3-ketoglucose-reductase activity,GO:0051990~(R)-2-hydroxyglutarate dehydrogenase activity,GO:0052677~D-arabinitol dehydrogenase, D-xylulose forming (NADP+) activity,

AKR7A3	aldo-keto reductase family 7 member A3(AKR7A3)	Homo sapiens	GO:0004033~aldo-keto reductase (NADP) activity,GO:0005515~protein binding,GO:0009055~electron carrier activity,GO:0042802~identical protein binding,
ALDOB	aldolase, fructose-bisphosphate B(ALDOB)	Homo sapiens	GO:0004332~fructose-bisphosphate aldolase activity,GO:0005515~protein binding,GO:0008092~cytoskeletal protein binding,GO:0042802~identical protein binding,GO:0051117~ATPase binding,GO:0061609~fructose-1-phosphate aldolase activity,GO:0070061~fructose binding,
AFP	alpha fetoprotein(AFP)	Homo sapiens	GO:0005515~protein binding,GO:0046872~metal ion binding,
ALPK1	alpha kinase 1(ALPK1)	Homo sapiens	GO:0004674~protein serine/threonine kinase activity,GO:0005524~ATP binding,GO:0016301~kinase activity,
ALPK2	alpha kinase 2(ALPK2)	Homo sapiens	GO:0004674~protein serine/threonine kinase activity,GO:0005524~ATP binding,
AOC3	amine oxidase, copper containing 3(AOC3)	Homo sapiens	GO:0005507~copper ion binding,GO:0005509~calcium ion binding,GO:0005515~protein binding,GO:0008131~primary amine oxidase activity,GO:0042803~protein homodimerization activity,GO:0046982~protein heterodimerization activity,GO:0048038~quinone binding,GO:0052593~tryptamine:oxygen oxidoreductase (deaminating) activity,GO:0052594~aminoacetone:oxygen oxidoreductase(deaminating) activity,GO:0052595~aliphatic-amine oxidase activity,GO:0052596~phenethylamine:oxygen oxidoreductase (deaminating) activity,
ALAD	aminolevulinate dehydratase(ALAD)	Homo sapiens	GO:0003824~catalytic activity,GO:0004655~porphobilinogen synthase activity,GO:0008270~zinc ion binding,GO:0032791~lead ion binding,GO:0042802~identical protein binding,GO:0046872~metal ion binding,
NPEPL1	aminopeptidase-like 1(NPEPL1)	Homo sapiens	GO:0004177~aminopeptidase activity,GO:0008235~metalloexopeptidase activity,GO:0030145~manganese ion binding,
AMN	amnion associated transmembrane protein(AMN)	Homo sapiens	GO:0005102~receptor binding,
AMOTL2	angiomotin like 2(AMOTL2)	Homo sapiens	GO:0005515~protein binding,GO:0042802~identical protein binding,
ANGPT2	angiopoietin 2(ANGPT2)	Homo sapiens	GO:0005102~receptor binding,GO:0005515~protein binding,GO:0030971~receptor tyrosine kinase binding,GO:0046872~metal ion binding,
ANK2	ankyrin 2(ANK2)	Homo sapiens	GO:0005200~structural constituent of cytoskeleton,GO:0005515~protein binding,GO:0008093~cytoskeletal adaptor activity,GO:0019899~enzyme binding,GO:0019901~protein kinase binding,GO:0030507~spectrin binding,GO:0030674~protein binding, bridging,GO:0044325~ion channel binding,GO:0051117~ATPase binding,
ANKHD1	ankyrin repeat and KH domain containing 1(ANKHD1)	Homo sapiens	GO:0003723~RNA binding,GO:0005515~protein binding,GO:0044822~poly(A) RNA binding,
ASB13	ankyrin repeat and SOCS box containing 13(ASB13)	Homo sapiens	GO:0005515~protein binding,
ASB9	ankyrin repeat and SOCS box containing 9(ASB9)	Homo sapiens	GO:0005515~protein binding,
ANKRD1	ankyrin repeat domain 1(ANKRD1)	Homo sapiens	GO:0001085~RNA polymerase II transcription factor binding,GO:0001105~RNA polymerase II transcription coactivator activity,GO:0002039~p53 binding,GO:0003677~DNA binding,GO:0003714~transcription corepressor activity,GO:0005515~protein binding,GO:0031432~titin binding,GO:0042826~histone deacetylase binding,GO:0070412~R-SMAD binding,
ANKRD10	ankyrin repeat domain 10(ANKRD10)	Homo sapiens	GO:0005515~protein binding,
ANXA9	annexin A9(ANXA9)	Homo sapiens	GO:0001786~phosphatidylserine binding,GO:0005509~calcium ion binding,GO:0005515~protein binding,GO:0005543~phospholipid binding,GO:0005544~calcium-dependent phospholipid binding,GO:0015464~acetylcholine receptor activity,GO:0042803~protein homodimerization activity,
ANO1	anoctamin 1(ANO1)	Homo sapiens	GO:0005227~calcium activated cation channel activity,GO:0005229~intracellular calcium activated chloride channel activity,GO:0005247~voltage-gated chloride channel activity,GO:0005254~chloride channel activity,GO:0005515~protein binding,GO:0015111~iodide transmembrane transporter activity,GO:0046983~protein dimerization activity,
ANO8	anoctamin 8(ANO8)	Homo sapiens	GO:0005229~intracellular calcium activated chloride channel activity,
ANO9	anoctamin 9(ANO9)	Homo sapiens	GO:0005229~intracellular calcium activated chloride channel activity,GO:0017128~phospholipid scramblase activity,
ANTXR1	anthrax toxin receptor 1(ANTXR1)	Homo sapiens	GO:0004872~receptor activity,GO:0004888~transmembrane signaling receptor activity,GO:0005515~protein binding,GO:0005518~collagen binding,GO:0046872~metal ion binding,GO:0051015~actin filament binding,
APOBEC3	apolipoprotein B mRNA editing enzyme catalytic subunit 3C(APOBEC3C)	Homo sapiens	GO:0005515~protein binding,GO:0008270~zinc ion binding,GO:0016814~hydrolase activity, acting on carbon-nitrogen (but not peptide) bonds, in cyclic amidines,GO:0044822~poly(A) RNA binding,
APOL2	apolipoprotein L2(APOL2)	Homo sapiens	GO:0005102~receptor binding,GO:0008035~high-density lipoprotein particle binding,GO:0008289~lipid binding,
AATF	apoptosis antagonizing transcription factor(AATF)	Homo sapiens	GO:0003700~transcription factor activity, sequence-specific DNA binding,GO:0005515~protein binding,GO:0043522~leucine zipper domain binding,GO:0044822~poly(A) RNA binding,GO:0048156~tau protein binding,
AATK	apoptosis associated tyrosine kinase(AATK)	Homo sapiens	GO:0004672~protein kinase activity,GO:0004674~protein serine/threonine kinase activity,GO:0005524~ATP binding,
AEN	apoptosis enhancing nuclease(AEN)	Homo sapiens	GO:0003676~nucleic acid binding,GO:0004527~exonuclease activity,GO:0005515~protein binding,
AQP6	aquaporin 6(AQP6)	Homo sapiens	GO:0005215~transporter activity,GO:0005253~anion channel activity,GO:0015112~nitrate transmembrane transporter activity,GO:0015250~water channel activity,GO:0015254~glycerol channel activity,
ALOX15	arachidonate 15-lipoxygenase(ALOX15)	Homo sapiens	GO:0004052~arachidonate 12-lipoxygenase activity,GO:0005506~iron ion binding,GO:0005515~protein binding,GO:0005546~phosphatidylinositol-4,5-bisphosphate binding,GO:0016702~oxidoreductase activity, acting on single donors with incorporation of molecular oxygen, incorporation of two atoms of oxygen,GO:0047977~hepoxilin-epoxide hydrolase activity,GO:0050473~arachidonate 15-lipoxygenase activity,GO:0051120~hepoxilin A3 synthase activity,GO:0097260~eoxin A4 synthase activity,
JMJD6	arginine demethylase and lysine hydroxylase(JMJD6)	Homo sapiens	GO:0003723~RNA binding,GO:0003727~single-stranded RNA binding,GO:0004872~receptor activity,GO:0005506~iron ion binding,GO:0005515~protein binding,GO:0008168~methyltransferase activity,GO:0032452~histone demethylase activity,GO:0033746~histone demethylase activity (H3-R2 specific),GO:0033749~histone demethylase activity (H4-R3 specific),GO:0042802~identical protein binding,GO:0042803~protein homodimerization activity,GO:0070815~peptidyl-lysine 5-dioxygenase activity,
AVPR1B	arginine vasopressin receptor 1B(AVPR1B)	Homo sapiens	GO:0005000~vasopressin receptor activity,GO:0005080~protein kinase C binding,GO:0042277~peptide binding,

AHR	aryl hydrocarbon receptor(AHR)	Homo sapiens	GO:0003677~DNA binding,GO:0003700~transcription factor activity, sequence-specific DNA binding,GO:0004874~aryl hydrocarbon receptor activity,GO:0004879~RNA polymerase II transcription factor activity, ligand-activated sequence-specific DNA binding,GO:0005515~protein binding,GO:0008134~transcription factor binding,GO:0035326~enhancer binding,GO:0044212~transcription regulatory region DNA binding,GO:0046982~protein heterodimerization activity,GO:0046983~protein dimerization activity,GO:0051879~Hsp90 protein binding,GO:0070888~E-box binding,
ARSG	arylsulfatase G(ARSG)	Homo sapiens	GO:0004065~arylsulfatase activity,GO:0008484~sulfuric ester hydrolase activity,GO:0046872~metal ion binding,
ARSJ	arylsulfatase family member J(ARSJ)	Homo sapiens	GO:0004065~arylsulfatase activity,GO:0008484~sulfuric ester hydrolase activity,GO:0046872~metal ion binding,
ASPG	asparaginase(ASPG)	Homo sapiens	GO:0003847~1-alkyl-2-acetylglycerophosphocholine esterase activity,GO:0004067~asparaginase activity,GO:0004622~lysophospholipase activity,
DRICH1	aspartate rich 1(DRICH1)	Homo sapiens	GO:0005515~protein binding,
ASPRV1	aspartic peptidase, retroviral-like 1(ASPRV1)	Homo sapiens	GO:0004190~aspartic-type endopeptidase activity,
ATXN7L1	ataxin 7 like 1(ATXN7L1)	Homo sapiens	GO:0005515~protein binding,
ACKR2	atypical chemokine receptor 2(ACKR2)	Homo sapiens	GO:0004950~chemokine receptor activity,GO:0005044~scavenger receptor activity,GO:0016493~C-C chemokine receptor activity,GO:0019957~C-C chemokine binding,
AURKC	aurora kinase C(AURKC)	Homo sapiens	GO:0004672~protein kinase activity,GO:0004674~protein serine/threonine kinase activity,GO:0004712~protein serine/threonine/tyrosine kinase activity,GO:0005524~ATP binding,GO:0035174~histone serine kinase activity,
BIRC3	baculoviral IAP repeat containing 3(BIRC3)	Homo sapiens	GO:0004842~ubiquitin-protein transferase activity,GO:0005515~protein binding,GO:0008270~zinc ion binding,GO:0016740~transferase activity,GO:0016874~ligase activity,GO:0043027~cysteine-type endopeptidase inhibitor activity involved in apoptotic process,
BFSP1	beaded filament structural protein 1(BFSP1)	Homo sapiens	GO:0005200~structural constituent of cytoskeleton,GO:0005212~structural constituent of eye lens,GO:0005515~protein binding,
B4GALNT	beta-1,4-N-acetyl-galactosaminyltransferase 4(B4GALNT4)	Homo sapiens	GO:0008376~acetylgalactosaminyltransferase activity,GO:0033842~N-acetyl-beta-glucosaminyl-glycoprotein 4-beta-N-acetylgalactosaminyltransferase activity,
UPB1	beta-ureidopropionase 1(UPB1)	Homo sapiens	GO:0003837~beta-ureidopropionase activity,GO:0016810~hydrolase activity, acting on carbon-nitrogen (but not peptide) bonds,GO:0046872~metal ion binding,
BFAR	bifunctional apoptosis regulator(BFAR)	Homo sapiens	GO:0005515~protein binding,GO:0008270~zinc ion binding,GO:0030674~protein binding, bridging,GO:0061630~ubiquitin protein ligase activity,GO:0089720~caspase binding,
BPHL	biphenyl hydrolase like(BPHL)	Homo sapiens	GO:0004649~poly(ADP-ribose) glycohydrolase activity,GO:0016787~hydrolase activity,GO:0047658~alpha-amino-acid esterase activity,
BDNF	brain derived neurotrophic factor(BDNF)	Homo sapiens	GO:0005169~neurotrophin TRKB receptor binding,GO:0008083~growth factor activity,
BTN3A1	butyrophilin subfamily 3 member A1(BTN3A1)	Homo sapiens	GO:0005515~protein binding,
CREB5	cAMP responsive element binding protein 5(CREB5)	Homo sapiens	GO:0003676~nucleic acid binding,GO:0003700~transcription factor activity, sequence-specific DNA binding,GO:0005515~protein binding,GO:0043565~sequence-specific DNA binding,GO:0046872~metal ion binding,
CDH7	cadherin 7(CDH7)	Homo sapiens	GO:0005509~calcium ion binding,
CELSR2	cadherin EGF LAG seven-pass G-type receptor 2(CELSR2)	Homo sapiens	GO:0004930~G-protein coupled receptor activity,GO:0005509~calcium ion binding,
CDH23	cadherin related 23(CDH23)	Homo sapiens	GO:0005509~calcium ion binding,GO:0005515~protein binding,
CALB1	calbindin 1(CALB1)	Homo sapiens	GO:0005499~vitamin D binding,GO:0005509~calcium ion binding,GO:0005515~protein binding,GO:0008270~zinc ion binding,
CRACR2B	calcium release activated channel regulator 2B(CRACR2B)	Homo sapiens	GO:0005509~calcium ion binding,GO:0005515~protein binding,
CACNG4	calcium voltage-gated channel auxiliary subunit gamma 4(CACNG4)	Homo sapiens	GO:0005245~voltage-gated calcium channel activity,GO:0005262~calcium channel activity,GO:0016247~channel regulator activity,
CACNA1I	calcium voltage-gated channel subunit alpha1 I(CACNA1I)	Homo sapiens	GO:0005245~voltage-gated calcium channel activity,GO:0005515~protein binding,GO:0008332~low voltage-gated calcium channel activity,
CAMKK2	calcium/calmodulin dependent protein kinase kinase 2(CAMKK2)	Homo sapiens	GO:0004674~protein serine/threonine kinase activity,GO:0004683~calmodulin-dependent protein kinase activity,GO:0004713~protein tyrosine kinase activity,GO:0005509~calcium ion binding,GO:0005516~calmodulin binding,GO:0005524~ATP binding,
CAPN1	calpain 1(CAPN1)	Homo sapiens	GO:0004198~calcium-dependent cysteine-type endopeptidase activity,GO:0005509~calcium ion binding,GO:0005515~protein binding,
CAPN13	calpain 13(CAPN13)	Homo sapiens	GO:0004198~calcium-dependent cysteine-type endopeptidase activity,GO:0005509~calcium ion binding,
CAPN5	calpain 5(CAPN5)	Homo sapiens	GO:0004198~calcium-dependent cysteine-type endopeptidase activity,
CNN3	calponin 3(CNN3)	Homo sapiens	GO:0003779~actin binding,GO:0005516~calmodulin binding,GO:0008017~microtubule binding,GO:0098641~cadherin binding involved in cell-cell adhesion,
CTIF	cap binding complex dependent translation initiation factor(CTIF)	Homo sapiens	GO:0003723~RNA binding,GO:0005515~protein binding,
CAD	carbamoyl-phosphate synthetase 2, aspartate transcarbamylase, and dihydroorotase(CAD)	Homo sapiens	GO:0004070~aspartate carbamoyltransferase activity,GO:0004087~carbamoyl-phosphate synthase (ammonia) activity,GO:0004088~carbamoyl-phosphate synthase (glutamine-hydrolyzing) activity,GO:0004151~dihydroorotase activity,GO:0004672~protein kinase activity,GO:0005524~ATP binding,GO:0008270~zinc ion binding,GO:0016597~amino acid binding,GO:0016743~carboxyl- or carbamoyltransferase activity,GO:0016810~hydrolase activity, acting on carbon-nitrogen (but not peptide) bonds,GO:0016812~hydrolase activity, acting on carbon-nitrogen (but not peptide) bonds, in cyclic amides,GO:0019899~enzyme binding,GO:0042802~identical protein binding,GO:0046872~metal ion binding,GO:0070335~aspartate binding,
CBR4	carbonyl reductase 4(CBR4)	Homo sapiens	GO:0003955~NAD(P)H dehydrogenase (quinone) activity,GO:0005515~protein binding,GO:0008753~NADPH dehydrogenase (quinone) activity,GO:0016491~oxidoreductase activity,GO:0047025~3-oxoacyl-[acyl-carrier-protein] reductase (NADH) activity,GO:0048038~quinone binding,GO:0070402~NADPH binding,
CES4A	carboxylesterase 4A(CES4A)	Homo sapiens	GO:0016787~hydrolase activity,GO:0052689~carboxylic ester hydrolase activity,
CMYA5	cardiomyopathy associated 5(CMYA5)	Homo sapiens	GO:0005515~protein binding,
CTF1	cardiotrophin 1(CTF1)	Homo sapiens	GO:0005125~cytokine activity,GO:0005146~leukemia inhibitory factor receptor binding,GO:0005515~protein binding,
CLCF1	cardiotrophin-like cytokine factor 1(CLCF1)	Homo sapiens	GO:0005102~receptor binding,GO:0005125~cytokine activity,GO:0005127~ciliary neurotrophic factor receptor binding,GO:0005515~protein binding,GO:0008083~growth factor activity,GO:0046982~protein heterodimerization activity,
CARNS1	carnosine synthase 1(CARNS1)	Homo sapiens	GO:0005524~ATP binding,GO:0016887~ATPase activity,GO:0046872~metal ion binding,GO:0047730~carnosine synthase activity,
CILP2	cartilage intermediate layer protein 2(CILP2)	Homo sapiens	GO:0004035~alkaline phosphatase activity,GO:0004551~nucleotide diphosphatase activity,
CILP	cartilage intermediate layer protein(CILP)	Homo sapiens	GO:0004035~alkaline phosphatase activity,GO:0004551~nucleotide diphosphatase activity,

CASP10	caspase 10(CASP10)	Homo sapiens	GO:0004197~cysteine-type endopeptidase activity,GO:0005515~protein binding,GO:0031625~ubiquitin protein ligase binding,GO:0035877~death effector domain binding,GO:0097199~cysteine-type endopeptidase activity involved in apoptotic signaling pathway,
CASP8AP2	caspase 8 associated protein 2(CASP8AP2)	Homo sapiens	GO:0003677~DNA binding,GO:0003714~transcription corepressor activity,GO:0005123~death receptor binding,GO:0005515~protein binding,GO:0008656~cysteine-type endopeptidase activator activity involved in apoptotic process,GO:0016505~peptidase activator activity involved in apoptotic process,GO:0032184~SUMO polymer binding,
COMTD1	catechol-O-methyltransferase domain containing 1(COMTD1)	Homo sapiens	GO:0005515~protein binding,GO:0008171~O-methyltransferase activity,
CTSK	cathepsin K(CTSK)	Homo sapiens	GO:0001968~fibronectin binding,GO:0004197~cysteine-type endopeptidase activity,GO:0004252~serine-type endopeptidase activity,GO:0005515~protein binding,GO:0005518~collagen binding,GO:0008234~cysteine-type peptidase activity,GO:0043394~proteoglycan binding,
CDC25B	cell division cycle 25B(CDC25B)	Homo sapiens	GO:0004721~phosphoprotein phosphatase activity,GO:0004725~protein tyrosine phosphatase activity,GO:0005515~protein binding,GO:0019901~protein kinase binding,
CGREF1	cell growth regulator with EF-hand domain 1(CGREF1)	Homo sapiens	GO:0005509~calcium ion binding,
CHMP2B	charged multivesicular body protein 2B(CHMP2B)	Homo sapiens	GO:0005515~protein binding,GO:0019904~protein domain specific binding,GO:0098641~cadherin binding involved in cell-cell adhesion,
CMKLR1	chemerin chemokine-like receptor 1(CMKLR1)	Homo sapiens	GO:0004872~receptor activity,GO:0004930~G-protein coupled receptor activity,GO:0004950~chemokine receptor activity,GO:0005515~protein binding,
CLCNKA	chloride voltage-gated channel Ka(CLCNKA)	Homo sapiens	GO:0005247~voltage-gated chloride channel activity,GO:0031404~chloride ion binding,GO:0046872~metal ion binding,
CLCNKB	chloride voltage-gated channel Kb(CLCNKB)	Homo sapiens	GO:0005247~voltage-gated chloride channel activity,GO:0031404~chloride ion binding,GO:0046872~metal ion binding,
CHDH	choline dehydrogenase(CHDH)	Homo sapiens	GO:0005515~protein binding,GO:0008812~choline dehydrogenase activity,GO:0016614~oxidoreductase activity, acting on CH-OH group of donors,GO:0050660~flavin adenine dinucleotide binding,
CHRNA10	cholinergic receptor nicotinic alpha 10 subunit(CHRNA10)	Homo sapiens	GO:0004889~acetylcholine-activated cation-selective channel activity,GO:0005102~receptor binding,GO:0005230~extracellular ligand-gated ion channel activity,GO:0005262~calcium channel activity,GO:0015464~acetylcholine receptor activity,GO:0042166~acetylcholine binding,
CHRNB4	cholinergic receptor nicotinic beta 4 subunit(CHRNB4)	Homo sapiens	GO:0004889~acetylcholine-activated cation-selective channel activity,GO:0005230~extracellular ligand-gated ion channel activity,GO:0008144~drug binding,GO:0015276~ligand-gated ion channel activity,GO:0015464~acetylcholine receptor activity,GO:0042166~acetylcholine binding,GO:0046982~protein heterodimerization activity,
CSPG4	chondroitin sulfate proteoglycan 4(CSPG4)	Homo sapiens	GO:0004871~signal transducer activity,GO:0019901~protein kinase binding,
CBX7	chromobox 7(CBX7)	Homo sapiens	GO:0003682~chromatin binding,GO:0003727~single-stranded RNA binding,GO:0005515~protein binding,GO:0035064~methylated histone binding,
CHD2	chromodomain helicase DNA binding protein 2(CHD2)	Homo sapiens	GO:0001046~core promoter sequence-specific DNA binding,GO:0003677~DNA binding,GO:0004003~ATP-dependent DNA helicase activity,GO:0004386~helicase activity,GO:0005515~protein binding,GO:0005524~ATP binding,GO:0042393~histone binding,GO:0044822~poly(A) RNA binding,
C1orf52	chromosome 1 open reading frame 52(C1orf52)	Homo sapiens	GO:0044822~poly(A) RNA binding,
C2orf15	chromosome 2 open reading frame 15(C2orf15)	Homo sapiens	GO:0044822~poly(A) RNA binding,
C2orf50	chromosome 2 open reading frame 50(C2orf50)	Homo sapiens	GO:0005515~protein binding,
C7orf25	chromosome 7 open reading frame 25(C7orf25)	Homo sapiens	GO:0005515~protein binding,
C8orf37	chromosome 8 open reading frame 37(C8orf37)	Homo sapiens	GO:0005515~protein binding,
CHTF18	chromosome transmission fidelity factor 18(CHTF18)	Homo sapiens	GO:0003677~DNA binding,GO:0003689~DNA clamp loader activity,GO:0005515~protein binding,GO:0005524~ATP binding,GO:0043142~single-stranded DNA-dependent ATPase activity,
CNTF	ciliary neurotrophic factor(CNTF)	Homo sapiens	GO:0005125~cytokine activity,GO:0005127~ciliary neurotrophic factor receptor binding,GO:0005138~interleukin-6 receptor binding,GO:0005515~protein binding,GO:0008083~growth factor activity,
CATIP	ciliogenesis associated TTC17 interacting protein(CATIP)	Homo sapiens	GO:0005515~protein binding,
CGN	cingulin(CGN)	Homo sapiens	GO:0003774~motor activity,GO:0003779~actin binding,GO:0005515~protein binding,GO:0098641~cadherin binding involved in cell-cell adhesion,
CLYBL	citrate lyase beta like(CLYBL)	Homo sapiens	GO:0000287~magnesium ion binding,GO:0003824~catalytic activity,GO:0004474~malate synthase activity,GO:0016829~lyase activity,GO:0046872~metal ion binding,
CLTCL1	clathrin heavy chain like 1(CLTCL1)	Homo sapiens	GO:0004871~signal transducer activity,GO:0005198~structural molecule activity,GO:0005515~protein binding,
CLDN16	claudin 16(CLDN16)	Homo sapiens	GO:0005198~structural molecule activity,GO:0005515~protein binding,GO:0015095~magnesium ion transmembrane transporter activity,GO:0042802~identical protein binding,
CLDN19	claudin 19(CLDN19)	Homo sapiens	GO:0005198~structural molecule activity,GO:0042802~identical protein binding,
CLDN24	claudin 24(CLDN24)	Homo sapiens	GO:0005198~structural molecule activity,
CLDN6	claudin 6(CLDN6)	Homo sapiens	GO:0005198~structural molecule activity,GO:0042802~identical protein binding,
CLUH	clustered mitochondria homolog(CLUH)	Homo sapiens	GO:0003729~mRNA binding,
F12	coagulation factor XII(F12)	Homo sapiens	GO:0004252~serine-type endopeptidase activity,GO:0005509~calcium ion binding,GO:0005515~protein binding,GO:0051787~misfolded protein binding,
CC2D1A	coiled-coil and C2 domain containing 1A(CC2D1A)	Homo sapiens	GO:0000978~RNA polymerase II core promoter proximal region sequence-specific DNA binding,GO:0000981~RNA polymerase II transcription factor activity, sequence-specific DNA binding,GO:0001078~transcriptional repressor activity, RNA polymerase II core promoter proximal region sequence-specific binding,GO:0004871~signal transducer activity,GO:0005515~protein binding,GO:0098641~cadherin binding involved in cell-cell adhesion,
CCDC110	coiled-coil domain containing 110(CCDC110)	Homo sapiens	GO:0005515~protein binding,
CCDC12	coiled-coil domain containing 12(CCDC12)	Homo sapiens	GO:0005515~protein binding,
CCDC153	coiled-coil domain containing 153(CCDC153)	Homo sapiens	GO:0005515~protein binding,
CCDC155	coiled-coil domain containing 155(CCDC155)	Homo sapiens	GO:0005515~protein binding,GO:0042802~identical protein binding,GO:0070840~dynein complex binding,
CCDC22	coiled-coil domain containing 22(CCDC22)	Homo sapiens	GO:0005515~protein binding,GO:0097602~cullin family protein binding,
CCDC24	coiled-coil domain containing 24(CCDC24)	Homo sapiens	GO:0005515~protein binding,
CCDC33	coiled-coil domain containing 33(CCDC33)	Homo sapiens	GO:0005515~protein binding,
CCDC62	coiled-coil domain containing 62(CCDC62)	Homo sapiens	GO:0030331~estrogen receptor binding,GO:0030374~ligand-dependent nuclear receptor transcription coactivator activity,
COL14A1	collagen type XIV alpha 1 chain(COL14A1)	Homo sapiens	GO:0005201~extracellular matrix structural constituent,GO:0005518~collagen binding,GO:0030674~protein binding, bridging,GO:0044822~poly(A) RNA binding,

COL17A1	collagen type XVII alpha 1 chain(COL17A1)	Homo sapiens	GO:0005515~protein binding,
C5	complement C5(C5)	Homo sapiens	GO:0004866~endopeptidase inhibitor activity,GO:0005102~receptor binding,GO:0005515~protein binding,GO:0008009~chemokine activity,
CTGF	connective tissue growth factor(CTGF)	Homo sapiens	GO:0001968~fibronectin binding,GO:0005178~integrin binding,GO:0005515~protein binding,GO:0005520~insulin-like growth factor binding,GO:0008022~protein C-terminus binding,GO:0008083~growth factor activity,GO:0008201~heparin binding,
CORO2A	coronin 2A(CORO2A)	Homo sapiens	GO:0051015~actin filament binding,
CRHR1	corticotropin releasing hormone receptor 1(CRHR1)	Homo sapiens	GO:0004930~G-protein coupled receptor activity,GO:0005515~protein binding,GO:0015056~corticotrophin-releasing factor receptor activity,
CRHR2	corticotropin releasing hormone receptor 2(CRHR2)	Homo sapiens	GO:0004930~G-protein coupled receptor activity,GO:0005515~protein binding,GO:0015056~corticotrophin-releasing factor receptor activity,
CKB	creatine kinase B(CKB)	Homo sapiens	GO:0004111~creatine kinase activity,GO:0005515~protein binding,GO:0005524~ATP binding,GO:0016301~kinase activity,GO:0031625~ubiquitin protein ligase binding,
CRYBA1	crystallin beta A1(CRYBA1)	Homo sapiens	GO:0005212~structural constituent of eye lens,GO:0005515~protein binding,
CUL9	cullin 9(CUL9)	Homo sapiens	GO:0005515~protein binding,GO:0005524~ATP binding,GO:0008270~zinc ion binding,GO:0031625~ubiquitin protein ligase binding,
CCND2	cyclin D2(CCND2)	Homo sapiens	GO:0005515~protein binding,GO:0019901~protein kinase binding,
CDK11A	cyclin dependent kinase 11A(CDK11A)	Homo sapiens	GO:0004672~protein kinase activity,GO:0004674~protein serine/threonine kinase activity,GO:0004693~cyclin-dependent protein serine/threonine kinase activity,GO:0005524~ATP binding,
CDK18	cyclin dependent kinase 18(CDK18)	Homo sapiens	GO:0004672~protein kinase activity,GO:0004674~protein serine/threonine kinase activity,GO:0004693~cyclin-dependent protein serine/threonine kinase activity,GO:0005515~protein binding,GO:0005524~ATP binding,
CDKN1A	cyclin dependent kinase inhibitor 1A(CDKN1A)	Homo sapiens	GO:0004861~cyclin-dependent protein serine/threonine kinase inhibitor activity,GO:0005515~protein binding,GO:0016301~kinase activity,GO:0019912~cyclin-dependent protein kinase activating kinase activity,GO:0030332~cyclin binding,GO:0031625~ubiquitin protein ligase binding,GO:0032403~protein complex binding,GO:0046872~metal ion binding,
CBSL	cystathionine-beta-synthase like(CBSL)	Homo sapiens	GO:0004122~cystathionine beta-synthase activity,GO:0004124~cysteine synthase activity,GO:0019825~oxygen binding,GO:0019899~enzyme binding,GO:0020037~heme binding,GO:0030170~pyridoxal phosphate binding,GO:0031625~ubiquitin protein ligase binding,GO:0042803~protein homodimerization activity,GO:0046872~metal ion binding,GO:0050421~nitrite reductase (NO-forming) activity,GO:0070025~carbon monoxide binding,GO:0070026~nitric oxide binding,GO:0072341~modified amino acid binding,GO:1904047~S-adenosyl-L-methionine binding,
CBS	cystathionine-beta-synthase(CBS)	Homo sapiens	GO:0004122~cystathionine beta-synthase activity,GO:0004124~cysteine synthase activity,GO:0005515~protein binding,GO:0019825~oxygen binding,GO:0019899~enzyme binding,GO:0020037~heme binding,GO:0030170~pyridoxal phosphate binding,GO:0031625~ubiquitin protein ligase binding,GO:0042802~identical protein binding,GO:0042803~protein homodimerization activity,GO:0046872~metal ion binding,GO:0050421~nitrite reductase (NO-forming) activity,GO:0070025~carbon monoxide binding,GO:0070026~nitric oxide binding,GO:0072341~modified amino acid binding,GO:1904047~S-adenosyl-L-methionine binding,
CSRNP1	cysteine and serine rich nuclear protein 1(CSRNP1)	Homo sapiens	GO:0001228~transcriptional activator activity, RNA polymerase II transcription regulatory region sequence-specific binding,GO:0003700~transcription factor activity, sequence-specific DNA binding,GO:0005515~protein binding,GO:0043565~sequence-specific DNA binding,
CYR61	cysteine rich angiogenic inducer 61(CYR61)	Homo sapiens	GO:0000166~nucleotide binding,GO:0005178~integrin binding,GO:0005520~insulin-like growth factor binding,GO:0008201~heparin binding,GO:0050840~extracellular matrix binding,
CRIP1	cysteine rich protein 1(CRIP1)	Homo sapiens	GO:0003680~AT DNA binding,GO:0008270~zinc ion binding,GO:0008301~DNA binding, bending,GO:0042277~peptide binding,
CRIP2	cysteine rich protein 2(CRIP2)	Homo sapiens	GO:0008270~zinc ion binding,
CRISPLD2	cysteine rich secretory protein LCCL domain containing 2(CRISPLD2)	Homo sapiens	GO:0008201~heparin binding,
CYP4B1	cytochrome P450 family 4 subfamily B member 1(CYP4B1)	Homo sapiens	GO:0004497~monooxygenase activity,GO:0005506~iron ion binding,GO:0008144~drug binding,GO:0015643~toxic substance binding,GO:0016705~oxidoreductase activity, acting on paired donors, with incorporation or reduction of molecular oxygen,GO:0018585~fluorene oxygenase activity,GO:0019825~oxygen binding,GO:0020037~heme binding,GO:0070330~aromatase activity,
CYP4V2	cytochrome P450 family 4 subfamily V member 2(CYP4V2)	Homo sapiens	GO:0004497~monooxygenase activity,GO:0005506~iron ion binding,GO:0016705~oxidoreductase activity, acting on paired donors, with incorporation or reduction of molecular oxygen,GO:0020037~heme binding,
CYP4X1	cytochrome P450 family 4 subfamily X member 1(CYP4X1)	Homo sapiens	GO:0005506~iron ion binding,GO:0020037~heme binding,GO:0070330~aromatase activity,
CYP8B1	cytochrome P450 family 8 subfamily B member 1(CYP8B1)	Homo sapiens	GO:0004497~monooxygenase activity,GO:0005506~iron ion binding,GO:0008397~sterol 12-alpha-hydroxylase activity,GO:0016705~oxidoreductase activity, acting on paired donors, with incorporation or reduction of molecular oxygen,GO:0019825~oxygen binding,GO:0020037~heme binding,GO:0033778~7alpha-hydroxycholest-4-en-3-one 12alpha-hydroxylase activity,
CYTH2	cytohesin 2(CYTH2)	Homo sapiens	GO:0005086~ARF guanyl-nucleotide exchange factor activity,GO:0005515~protein binding,GO:0008289~lipid binding,GO:0070679~inositol 1,4,5 trisphosphate binding,
DEDD2	death effector domain containing 2(DEDD2)	Homo sapiens	GO:0003677~DNA binding,GO:0005515~protein binding,GO:0030159~receptor signaling complex scaffold activity,
DOCK11	dedicator of cytokinesis 11(DOCK11)	Homo sapiens	GO:0005085~guanyl-nucleotide exchange factor activity,GO:0005089~Rho guanyl-nucleotide exchange factor activity,GO:0005515~protein binding,GO:0017048~Rho GTPase binding,
DHRS2	dehydrogenase/reductase 2(DHRS2)	Homo sapiens	GO:0004090~carbonyl reductase (NADPH) activity,GO:0005515~protein binding,GO:0016491~oxidoreductase activity,
DHRS4L2	dehydrogenase/reductase 4 like 2(DHRS4L2)	Homo sapiens	GO:0004090~carbonyl reductase (NADPH) activity,GO:0016491~oxidoreductase activity,
DHRS4	dehydrogenase/reductase 4(DHRS4)	Homo sapiens	GO:0000253~3-keto sterol reductase activity,GO:0004090~carbonyl reductase (NADPH) activity,GO:0005102~receptor binding,GO:0016655~oxidoreductase activity, acting on NAD(P)H, quinone or similar compound as acceptor,GO:0018455~alcohol dehydrogenase [NAD(P)+] activity,GO:0052650~NADP-retinol dehydrogenase activity,
DLK2	delta like non-canonical Notch ligand 2(DLK2)	Homo sapiens	GO:0005509~calcium ion binding,GO:0042803~protein homodimerization activity,
DTX1	deltex E3 ubiquitin ligase 1(DTX1)	Homo sapiens	GO:0003713~transcription coactivator activity,GO:0005112~Notch binding,GO:0005515~protein binding,GO:0008270~zinc ion binding,GO:0016874~ligase activity,GO:0017124~SH3 domain binding,GO:0031625~ubiquitin protein ligase binding,
DSG1	desmoglein 1(DSG1)	Homo sapiens	GO:0005509~calcium ion binding,GO:0005515~protein binding,GO:0015643~toxic substance binding,GO:0045295~gamma-catenin binding,
DGKQ	diacylglycerol kinase theta(DGKQ)	Homo sapiens	GO:0004143~diacylglycerol kinase activity,GO:0005515~protein binding,GO:0005524~ATP binding,GO:0019900~kinase binding,GO:0033613~activating transcription factor binding,GO:0043274~phospholipase binding,GO:0046872~metal ion binding,

DKK1	dickkopf WNT signaling pathway inhibitor 1(DKK1)	Homo sapiens	GO:0004871~signal transducer activity,GO:0005515~protein binding,GO:0008083~growth factor activity,GO:0039706~co-receptor binding,GO:0048019~receptor antagonist activity,GO:0050750~low-density lipoprotein particle receptor binding,
DPYSL5	dihydropyrimidinase like 5(DPYSL5)	Homo sapiens	GO:0005515~protein binding,GO:0008017~microtubule binding,GO:0016810~hydrolase activity, acting on carbon-nitrogen (but not peptide) bonds,
DPH5	diphthamide biosynthesis 5(DPH5)	Homo sapiens	GO:0004164~diphthine synthase activity,
DPH6	diphthamine biosynthesis 6(DPH6)	Homo sapiens	GO:0005524~ATP binding,GO:0017178~diphthine-ammonia ligase activity,
DDR2	discoidin domain receptor tyrosine kinase 2(DDR2)	Homo sapiens	GO:0004714~transmembrane receptor protein tyrosine kinase activity,GO:0005515~protein binding,GO:0005518~collagen binding,GO:0005524~ATP binding,GO:0038062~protein tyrosine kinase collagen receptor activity,
DVL1	dishevelled segment polarity protein 1(DVL1)	Homo sapiens	GO:0005109~frizzled binding,GO:0005515~protein binding,GO:0008013~beta-catenin binding,GO:0019899~enzyme binding,GO:0019901~protein kinase binding,GO:0042802~identical protein binding,GO:0048365~Rac GTPase binding,
DLX3	distal-less homeobox 3(DLX3)	Homo sapiens	GO:0001077~transcriptional activator activity, RNA polymerase II core promoter proximal region sequence-specific binding,GO:0003682~chromatin binding,GO:0003700~transcription factor activity, sequence-specific DNA binding,GO:0043565~sequence-specific DNA binding,
DLX4	distal-less homeobox 4(DLX4)	Homo sapiens	GO:0000978~RNA polymerase II core promoter proximal region sequence-specific DNA binding,GO:0001078~transcriptional repressor activity, RNA polymerase II core promoter proximal region sequence-specific binding,GO:0003700~transcription factor activity, sequence-specific DNA binding,GO:0005515~protein binding,GO:0043565~sequence-specific DNA binding,
DOLPP1	dolichyldiphosphatase 1(DOLPP1)	Homo sapiens	GO:0047874~dolichyldiphosphatase activity,
DPY19L2	dpy-19 like 2(DPY19L2)	Homo sapiens	GO:0000030~mannosyltransferase activity,
DAPP1	dual adaptor of phosphotyrosine and 3-phosphoinositides 1(DAPP1)	Homo sapiens	GO:0005515~protein binding,GO:0005543~phospholipid binding,GO:0005547~phosphatidylinositol-3,4,5-trisphosphate binding,GO:0043325~phosphatidylinositol-3,4-bisphosphate binding,
DUSP1	dual specificity phosphatase 1(DUSP1)	Homo sapiens	GO:0004725~protein tyrosine phosphatase activity,GO:0004726~non-membrane spanning protein tyrosine phosphatase activity,GO:0005515~protein binding,GO:0008330~protein tyrosine/threonine phosphatase activity,GO:0017017~MAP kinase tyrosine/serine/threonine phosphatase activity,GO:0019838~growth factor binding,
DUSP10	dual specificity phosphatase 10(DUSP10)	Homo sapiens	GO:0004721~phosphoprotein phosphatase activity,GO:0004725~protein tyrosine phosphatase activity,GO:0016791~phosphatase activity,GO:0017017~MAP kinase tyrosine/serine/threonine phosphatase activity,GO:0033549~MAP kinase phosphatase activity,
DUSP19	dual specificity phosphatase 19(DUSP19)	Homo sapiens	GO:0004725~protein tyrosine phosphatase activity,GO:0004860~protein kinase inhibitor activity,GO:0005078~MAP-kinase scaffold activity,GO:0005515~protein binding,GO:0008579~JUN kinase phosphatase activity,GO:0030295~protein kinase activator activity,GO:0031435~mitogen-activated protein kinase kinase kinase binding,
DUSP4	dual specificity phosphatase 4(DUSP4)	Homo sapiens	GO:0004725~protein tyrosine phosphatase activity,GO:0008330~protein tyrosine/threonine phosphatase activity,GO:0016791~phosphatase activity,GO:0017017~MAP kinase tyrosine/serine/threonine phosphatase activity,GO:1990439~MAP kinase threonine phosphatase activity,
DUSP5	dual specificity phosphatase 5(DUSP5)	Homo sapiens	GO:0004725~protein tyrosine phosphatase activity,GO:0005515~protein binding,GO:0008138~protein tyrosine/serine/threonine phosphatase activity,GO:0016791~phosphatase activity,GO:0017017~MAP kinase tyrosine/serine/threonine phosphatase activity,
DUSP8	dual specificity phosphatase 8(DUSP8)	Homo sapiens	GO:0004725~protein tyrosine phosphatase activity,GO:0016791~phosphatase activity,GO:0017017~MAP kinase tyrosine/serine/threonine phosphatase activity,
DNM1	dynamin 1(DNM1)	Homo sapiens	GO:0003924~GTPase activity,GO:0005515~protein binding,GO:0005525~GTP binding,GO:0008017~microtubule binding,GO:0008022~protein C-terminus binding,GO:0019901~protein kinase binding,GO:0031749~D2 dopamine receptor binding,GO:0032403~protein complex binding,GO:0042802~identical protein binding,GO:0044822~poly(A) RNA binding,GO:0046983~protein dimerization activity,GO:0050998~nitric-oxide synthase binding,
DYNC2LI1	dynein cytoplasmic 2 light intermediate chain 1(DYNC2LI1)	Homo sapiens	GO:0003774~motor activity,GO:0005515~protein binding,GO:0045504~dynein heavy chain binding,
DYNLT1	dynein light chain Tctex-type 1(DYNLT1)	Homo sapiens	GO:0003774~motor activity,GO:0005515~protein binding,GO:0042802~identical protein binding,
DRC7	dynein regulatory complex subunit 7(DRC7)	Homo sapiens	GO:0005515~protein binding,
DYSF	dysferlin(DYSF)	Homo sapiens	GO:0005509~calcium ion binding,GO:0005515~protein binding,GO:0005543~phospholipid binding,GO:0005544~calcium-dependent phospholipid binding,
EBF4	early B-cell factor 4(EBF4)	Homo sapiens	GO:0000977~RNA polymerase II regulatory region sequence-specific DNA binding,GO:0001077~transcriptional activator activity, RNA polymerase II core promoter proximal region sequence-specific binding,GO:0001228~transcriptional activator activity, RNA polymerase II transcription regulatory region sequence-specific binding,GO:0003677~DNA binding,GO:0046872~metal ion binding,
EML2	echinoderm microtubule associated protein like 2(EML2)	Homo sapiens	GO:0005102~receptor binding,GO:0005515~protein binding,GO:0008017~microtubule binding,GO:0008022~protein C-terminus binding,GO:0015631~tubulin binding,
EML3	echinoderm microtubule associated protein like 3(EML3)	Homo sapiens	GO:0008017~microtubule binding,
EVI5L	ecotropic viral integration site 5 like(EVI5L)	Homo sapiens	GO:0005096~GTPase activator activity,GO:0005515~protein binding,GO:0017137~Rab GTPase binding,
ENOX2	ecto-NOX disulfide-thiol exchanger 2(ENOX2)	Homo sapiens	GO:0000166~nucleotide binding,GO:0003676~nucleic acid binding,GO:0015035~protein disulfide oxidoreductase activity,
ENC1	ectodermal-neural cortex 1(ENC1)	Homo sapiens	GO:0003779~actin binding,GO:0005515~protein binding,
ENTPD5	ectonucleoside triphosphate diphosphohydrolase 5(ENTPD5)	Homo sapiens	GO:0004382~guanosine-diphosphatase activity,GO:0005515~protein binding,GO:0016787~hydrolase activity,GO:0045134~uridine-diphosphatase activity,
ENTPD8	ectonucleoside triphosphate diphosphohydrolase 8(ENTPD8)	Homo sapiens	GO:0005524~ATP binding,GO:0016787~hydrolase activity,GO:0017110~nucleoside-diphosphatase activity,GO:0017111~nucleoside-triphosphatase activity,GO:0046872~metal ion binding,
ENPP3	ectonucleotide pyrophosphatase/phosphodiesterase 3(ENPP3)	Homo sapiens	GO:0003676~nucleic acid binding,GO:0004528~phosphodiesterase I activity,GO:0004551~nucleotide diphosphatase activity,GO:0005044~scavenger receptor activity,GO:0030247~polysaccharide binding,GO:0035529~NADH pyrophosphatase activity,GO:0046872~metal ion binding,GO:0047429~nucleoside-triphosphate diphosphatase activity,
ELN	elastin(ELN)	Homo sapiens	GO:0005201~extracellular matrix structural constituent,GO:0005515~protein binding,GO:0050840~extracellular matrix binding,
ELL2	elongation factor for RNA polymerase II 2(ELL2)	Homo sapiens	GO:0003746~translation elongation factor activity,
ENDOG	endonuclease G(ENDOG)	Homo sapiens	GO:0003676~nucleic acid binding,GO:0004519~endonuclease activity,GO:0004536~deoxyribonuclease activity,GO:0005515~protein binding,GO:0046872~metal ion binding,

ENDOU	endonuclease, poly(U) specific(ENDOU)	Homo sapiens	GO:0003723~RNA binding,GO:0004521~endoribonuclease activity,GO:0005044~scavenger receptor activity,GO:0008083~growth factor activity,GO:0008236~serine-type peptidase activity,GO:0030145~manganese ion binding,GO:0030247~polysaccharide binding,
EDN1	endothelin 1(EDN1)	Homo sapiens	GO:0005125~cytokine activity,GO:0005179~hormone activity,GO:0005515~protein binding,GO:0031707~endothelin A receptor binding,GO:0031708~endothelin B receptor binding,
EN2	engrailed homeobox 2(EN2)	Homo sapiens	GO:0043565~sequence-specific DNA binding,
EFNA3	ephrin A3(EFNA3)	Homo sapiens	GO:0005005~transmembrane-ephrin receptor activity,GO:0005515~protein binding,GO:0046875~ephrin receptor binding,
EFNB2	ephrin B2(EFNB2)	Homo sapiens	GO:0001618~virus receptor activity,GO:0005515~protein binding,GO:0046875~ephrin receptor binding,
EFNB3	ephrin B3(EFNB3)	Homo sapiens	GO:0001618~virus receptor activity,GO:0005005~transmembrane-ephrin receptor activity,GO:0046875~ephrin receptor binding,
EGFR	epidermal growth factor receptor(EGFR)	Homo sapiens	GO:0001948~glycoprotein binding,GO:0003682~chromatin binding,GO:0003690~double-stranded DNA binding,GO:0004672~protein kinase activity,GO:0004709~MAP kinase kinase kinase activity,GO:0004713~protein tyrosine kinase activity,GO:0004714~transmembrane receptor protein tyrosine kinase activity,GO:0004716~receptor signaling protein tyrosine kinase activity,GO:0004888~transmembrane signaling receptor activity,GO:0005006~epidermal growth factor-activated receptor activity,GO:0005088~Ras guanyl-nucleotide exchange factor activity,GO:0005178~integrin binding,GO:0005515~protein binding,GO:0005516~calmodulin binding,GO:0005524~ATP binding,GO:0019899~enzyme binding,GO:0019901~protein kinase binding,GO:0019903~protein phosphatase binding,GO:0030235~nitric-oxide synthase regulator activity,GO:0031625~ubiquitin protein ligase binding,GO:0042802~identical protein binding,GO:0046934~phosphatidylinositol-4,5-bisphosphate 3-kinase activity,GO:0046982~protein heterodimerization activity,GO:0048408~epidermal growth factor binding,GO:0051015~actin filament binding,GO:0098641~cadherin binding involved in cell-cell adhesion,
ECT2L	epithelial cell transforming 2 like(ECT2L)	Homo sapiens	GO:0005089~Rho guanyl-nucleotide exchange factor activity,
EPGN	epithelial mitogen(EPGN)	Homo sapiens	GO:0005154~epidermal growth factor receptor binding,GO:0008083~growth factor activity,
EPHX2	epoxide hydrolase 2(EPHX2)	Homo sapiens	GO:0000287~magnesium ion binding,GO:0003824~catalytic activity,GO:0004301~epoxide hydrolase activity,GO:0005102~receptor binding,GO:0015643~toxic substance binding,GO:0016787~hydrolase activity,GO:0016791~phosphatase activity,GO:0033885~10-hydroxy-9-(phosphonooxy)octadecanoate phosphatase activity,GO:0042577~lipid phosphatase activity,GO:0042803~protein homodimerization activity,
EPN3	epsin 3(EPN3)	Homo sapiens	GO:0005515~protein binding,GO:0008289~lipid binding,GO:1990175~EH domain binding,
ERFE	erythroferrone(ERFE)	Homo sapiens	GO:0005179~hormone activity,GO:0042803~protein homodimerization activity,
ESPN	espin(ESPN)	Homo sapiens	GO:0017124~SH3 domain binding,GO:0051015~actin filament binding,
ESRRA	estrogen related receptor alpha(ESRRA)	Homo sapiens	GO:0000978~RNA polymerase II core promoter proximal region sequence-specific DNA binding,GO:0001077~transcriptional activator activity, RNA polymerase II core promoter proximal region sequence-specific binding,GO:0001078~transcriptional repressor activity, RNA polymerase II core promoter proximal region sequence-specific binding,GO:0003677~DNA binding,GO:0003700~transcription factor activity, sequence-specific DNA binding,GO:0003707~steroid hormone receptor activity,GO:0004879~RNA polymerase II transcription factor activity, ligand-activated sequence-specific DNA binding,GO:0005496~steroid binding,GO:0005515~protein binding,GO:0008270~zinc ion binding,GO:0019904~protein domain specific binding,GO:0043565~sequence-specific DNA binding,
ESRRB	estrogen related receptor beta(ESRRB)	Homo sapiens	GO:0000980~RNA polymerase II distal enhancer sequence-specific DNA binding,GO:0003700~transcription factor activity, sequence-specific DNA binding,GO:0003707~steroid hormone receptor activity,GO:0003713~transcription coactivator activity,GO:0004879~RNA polymerase II transcription factor activity, ligand-activated sequence-specific DNA binding,GO:0005496~steroid binding,GO:0008134~transcription factor binding,GO:0008270~zinc ion binding,GO:0043565~sequence-specific DNA binding,
ESRRG	estrogen related receptor gamma(ESRRG)	Homo sapiens	GO:0000977~RNA polymerase II regulatory region sequence-specific DNA binding,GO:0001228~transcriptional activator activity, RNA polymerase II transcription regulatory region sequence-specific binding,GO:0003677~DNA binding,GO:0003700~transcription factor activity, sequence-specific DNA binding,GO:0003707~steroid hormone receptor activity,GO:0003708~retinoic acid receptor activity,GO:0005496~steroid binding,GO:0005515~protein binding,GO:0008270~zinc ion binding,GO:0043565~sequence-specific DNA binding,GO:0050682~AF-2 domain binding,
ETNPPL	ethanolamine-phosphate phospho-lyase(ETNPPL)	Homo sapiens	GO:0008483~transaminase activity,GO:0030170~pyridoxal phosphate binding,GO:0042802~identical protein binding,GO:0050459~ethanolamine-phosphate phospho-lyase activity,
EEFSEC	eukaryotic elongation factor, selenocysteine-tRNA specific(EEFSEC)	Homo sapiens	GO:0000049~tRNA binding,GO:0003746~translation elongation factor activity,GO:0003924~GTPase activity,GO:0005525~GTP binding,GO:0035368~selenocysteine insertion sequence binding,GO:0043021~ribonucleoprotein complex binding,
EXOC8	exocyst complex component 8(EXOC8)	Homo sapiens	GO:0005515~protein binding,GO:0017160~Ral GTPase binding,
EXTL1	exostosin like glycosyltransferase 1(EXTL1)	Homo sapiens	GO:0050508~glucuronosyl-N-acetylglucosaminyl-proteoglycan 4-alpha-N-acetylglucosaminyltransferase activity,
ESYT1	extended synaptotagmin 1(ESYT1)	Homo sapiens	GO:0005515~protein binding,GO:0008289~lipid binding,GO:0046872~metal ion binding,
FAM111B	family with sequence similarity 111 member B(FAM111B)	Homo sapiens	GO:0005515~protein binding,
FAM133B	family with sequence similarity 133 member B(FAM133B)	Homo sapiens	GO:0044822~poly(A) RNA binding,
FAM189B	family with sequence similarity 189 member B(FAM189B)	Homo sapiens	GO:0005515~protein binding,GO:0050699~WW domain binding,
FAM76A	family with sequence similarity 76 member A(FAM76A)	Homo sapiens	GO:0004175~endopeptidase activity,GO:0016929~SUMO-specific protease activity,
FAM86B1	family with sequence similarity 86 member B1(FAM86B1)	Homo sapiens	GO:0008168~methyltransferase activity,
FAM86B2	family with sequence similarity 86 member B2(FAM86B2)	Homo sapiens	GO:0008168~methyltransferase activity,
FAM86C1	family with sequence similarity 86 member C1(FAM86C1)	Homo sapiens	GO:0005515~protein binding,GO:0008168~methyltransferase activity,
FAM86C2	family with sequence similarity 86, member A pseudogene(FAM86C2P)	Homo sapiens	GO:0008168~methyltransferase activity,
FA2H	fatty acid 2-hydroxylase(FA2H)	Homo sapiens	GO:0005506~iron ion binding,GO:0020037~heme binding,GO:0046872~metal ion binding,GO:0080132~fatty acid alpha-hydroxylase activity,
FADS1	fatty acid desaturase 1(FADS1)	Homo sapiens	GO:0000248~C-5 sterol desaturase activity,GO:0016491~oxidoreductase activity,GO:0016717~oxidoreductase activity, acting on paired donors, with oxidation of a pair of donors resulting in the reduction of molecular oxygen to two molecules of water,GO:0045485~omega-6 fatty acid desaturase activity,
FADS3	fatty acid desaturase 3(FADS3)	Homo sapiens	GO:0016491~oxidoreductase activity,

FASN	fatty acid synthase(FASN)	Homo sapiens	GO:0003824~catalytic activity,GO:0004312~fatty acid synthase activity,GO:0004313~[acyl-carrier-protein] S-acetyltransferase activity,GO:0004314~[acyl-carrier-protein] S-malonyltransferase activity,GO:0004315~3-oxoacyl-[acyl-carrier-protein] synthase activity,GO:0004316~3-oxoacyl-[acyl-carrier-protein] reductase (NADPH) activity,GO:0004317~3-hydroxypalmitoyl-[acyl-carrier-protein] dehydratase activity,GO:0004319~enoyl-[acyl-carrier-protein] reductase (NADPH, B-specific) activity,GO:0004320~oleoyl-[acyl-carrier-protein] hydrolase activity,GO:0005515~protein binding,GO:0008144~drug binding,GO:0016295~myristoyl-[acyl-carrier-protein] hydrolase activity,GO:0016296~palmitoyl-[acyl-carrier-protein] hydrolase activity,GO:0016491~oxidoreductase activity,GO:0031177~phosphopantetheine binding,GO:0042803~protein homodimerization activity,GO:0044822~poly(A) RNA binding,GO:0047117~enoyl-[acyl-carrier-protein] reductase (NADPH, A-specific) activity,GO:0047451~3-hydroxyoctanoyl-[acyl-carrier-protein] dehydratase activity,GO:0070402~NADPH binding,GO:0098641~cadherin binding involved in cell-cell adhesion,GO:0102132~3-oxo-pimeloyl-[acp] methyl ester reductase activity,
FAR2P1	fatty acyl-CoA reductase 2 pseudogene 1(FAR2P1)	Homo sapiens	GO:0080019~fatty-acyl-CoA reductase (alcohol-forming) activity,
FEM1A	fem-1 homolog A(FEM1A)	Homo sapiens	GO:0004842~ubiquitin-protein transferase activity,GO:0031867~EP4 subtype prostaglandin E2 receptor binding,
FBN3	fibrillin 3(FBN3)	Homo sapiens	GO:0005201~extracellular matrix structural constituent,GO:0005509~calcium ion binding,
FGFR3	fibroblast growth factor receptor 3(FGFR3)	Homo sapiens	GO:0004713~protein tyrosine kinase activity,GO:0005007~fibroblast growth factor-activated receptor activity,GO:0005088~Ras guanyl-nucleotide exchange factor activity,GO:0005515~protein binding,GO:0005524~ATP binding,GO:0016303~1-phosphatidylinositol-3-kinase activity,GO:0017134~fibroblast growth factor binding,GO:0046934~phosphatidylinositol-4,5-bisphosphate 3-kinase activity,
FLRT1	fibronectin leucine rich transmembrane protein 1(FLRT1)	Homo sapiens	GO:0004860~protein kinase inhibitor activity,GO:0005057~receptor signaling protein activity,GO:0005104~fibroblast growth factor receptor binding,GO:0030674~protein binding, bridging,
FBLN7	fibulin 7(FBLN7)	Homo sapiens	GO:0005509~calcium ion binding,GO:0008201~heparin binding,
FLT4	fms related tyrosine kinase 4(FLT4)	Homo sapiens	GO:0004714~transmembrane receptor protein tyrosine kinase activity,GO:0005021~vascular endothelial growth factor-activated receptor activity,GO:0005515~protein binding,GO:0005524~ATP binding,GO:0019838~growth factor binding,GO:0019903~protein phosphatase binding,
FOLH1	folate hydrolase 1(FOLH1)	Homo sapiens	GO:0004181~metallocarboxypeptidase activity,GO:0008233~peptidase activity,GO:0016805~dipeptidase activity,GO:0046872~metal ion binding,GO:1904492~Ac-Asp-Glu binding,GO:1904493~tetrahydrofolyl-poly(glutamate) polymer binding,
FOXC1	forkhead box C1(FOXC1)	Homo sapiens	GO:0000977~RNA polymerase II regulatory region sequence-specific DNA binding,GO:0000981~RNA polymerase II transcription factor activity, sequence specific DNA binding,GO:0001228~transcriptional activator activity, RNA polymerase II transcription regulatory region sequence-specific binding,GO:0003677~DNA binding,GO:0003700~transcription factor activity, sequence-specific DNA binding,GO:0003705~transcription factor activity, RNA polymerase II distal enhancer sequence-specific binding,GO:0005515~protein binding,GO:0008134~transcription factor binding,GO:0008301~DNA binding, bending,GO:0043565~sequence-specific DNA binding,GO:0044212~transcription regulatory region DNA binding,
FOXD4	forkhead box D4(FOXD4)	Homo sapiens	GO:0000981~RNA polymerase II transcription factor activity, sequence-specific DNA binding,GO:0003700~transcription factor activity, sequence-specific DNA binding,GO:0008301~DNA binding, bending,GO:0043565~sequence-specific DNA binding,
FOXD4L1	forkhead box D4-like 1(FOXD4L1)	Homo sapiens	GO:0000981~RNA polymerase II transcription factor activity, sequence-specific DNA binding,GO:0003700~transcription factor activity, sequence-specific DNA binding,GO:0043565~sequence-specific DNA binding,
FOXD4L4	forkhead box D4-like 4(FOXD4L4)	Homo sapiens	GO:0000981~RNA polymerase II transcription factor activity, sequence-specific DNA binding,GO:0043565~sequence-specific DNA binding,
FOXE3	forkhead box E3(FOXE3)	Homo sapiens	GO:0000981~RNA polymerase II transcription factor activity, sequence-specific DNA binding,GO:0003677~DNA binding,GO:0003700~transcription factor activity, sequence-specific DNA binding,GO:0043565~sequence-specific DNA binding,
FOXF2	forkhead box F2(FOXF2)	Homo sapiens	GO:0000977~RNA polymerase II regulatory region sequence-specific DNA binding,GO:0001228~transcriptional activator activity, RNA polymerase II transcription regulatory region sequence-specific binding,GO:0003677~DNA binding,GO:0003700~transcription factor activity, sequence-specific DNA binding,GO:0003705~transcription factor activity, RNA polymerase II distal enhancer sequence-specific binding,GO:0008134~transcription factor binding,GO:0043565~sequence-specific DNA binding,
FOXN4	forkhead box N4(FOXN4)	Homo sapiens	GO:0001158~enhancer sequence-specific DNA binding,GO:0003682~chromatin binding,GO:0003700~transcription factor activity, sequence-specific DNA binding,GO:0005515~protein binding,GO:0043565~sequence-specific DNA binding,
FOXQ1	forkhead box Q1(FOXQ1)	Homo sapiens	GO:0000981~RNA polymerase II transcription factor activity, sequence-specific DNA binding,GO:0043565~sequence-specific DNA binding,
FOXS1	forkhead box S1(FOXS1)	Homo sapiens	GO:0000981~RNA polymerase II transcription factor activity, sequence-specific DNA binding,GO:0003677~DNA binding,GO:0003700~transcription factor activity, sequence-specific DNA binding,GO:0043565~sequence-specific DNA binding,
FRZB	frizzled-related protein(FRZB)	Homo sapiens	GO:0004930~G-protein coupled receptor activity,GO:0017147~Wnt-protein binding,GO:0042813~Wnt-activated receptor activity,
FN3K	fructosamine 3 kinase(FN3K)	Homo sapiens	GO:0016301~kinase activity,GO:0030387~fructosamine-3-kinase activity,
FBP1	fructose-bisphosphatase 1(FBP1)	Homo sapiens	GO:0005515~protein binding,GO:0016208~AMP binding,GO:0042132~fructose 1,6-bisphosphate 1-phosphatase activity,GO:0042802~identical protein binding,GO:0046872~metal ion binding,GO:0048029~monosaccharide binding,
FAHD2B	fumarylacetoacetate hydrolase domain containing 2B(FAHD2B)	Homo sapiens	GO:0016787~hydrolase activity,GO:0046872~metal ion binding,
FUZ	fuzzy planar cell polarity protein(FUZ)	Homo sapiens	GO:0005515~protein binding,
GALK1	galactokinase 1(GALK1)	Homo sapiens	GO:0004335~galactokinase activity,GO:0005524~ATP binding,GO:0005534~galactose binding,
GAL3ST1	galactose-3-O-sulfotransferase 1(GAL3ST1)	Homo sapiens	GO:0001733~galactosylceramide sulfotransferase activity,GO:0008146~sulfotransferase activity,
GGN	gametogenetin(GGN)	Homo sapiens	GO:0005515~protein binding,
GABRP	gamma-aminobutyric acid type A receptor pi subunit(GABRP)	Homo sapiens	GO:0004890~GABA-A receptor activity,GO:0005230~extracellular ligand-gated ion channel activity,GO:0008068~extracellular-glutamate-gated chloride channel activity,
GGT1	gamma-glutamyltransferase 1(GGT1)	Homo sapiens	GO:0003840~gamma-glutamyltransferase activity,GO:0005515~protein binding,GO:0036374~glutathione hydrolase activity,
GGT6	gamma-glutamyltransferase 6(GGT6)	Homo sapiens	GO:0003840~gamma-glutamyltransferase activity,GO:0036374~glutathione hydrolase activity,
GSAP	protein(GSAP)	Homo sapiens	GO:0001540~beta-amyloid binding,

GJD3	gap junction protein delta 3(GJD3)	Homo sapiens	GO:0005216~ion channel activity,GO:0005243~gap junction channel activity,GO:0005515~protein binding,GO:0086075~gap junction channel activity involved in cardiac conduction electrical coupling,
GJC1	gap junction protein gamma 1(GJC1)	Homo sapiens	GO:0005216~ion channel activity,GO:0005243~gap junction channel activity,GO:0086020~gap junction channel activity involved in SA node cell-atrial cardiac muscle cell electrical coupling,GO:0086077~gap junction channel activity involved in AV node cell-bundle of His cell electrical coupling,
GIPR	gastric inhibitory polypeptide receptor(GIPR)	Homo sapiens	GO:0004888~transmembrane signaling receptor activity,GO:0016519~gastric inhibitory peptide receptor activity,GO:0017046~peptide hormone binding,
GTF2H3	general transcription factor IIH subunit 3(GTF2H3)	Homo sapiens	GO:0003684~damaged DNA binding,GO:0003700~transcription factor activity, sequence-specific DNA binding,GO:0004672~protein kinase activity,GO:0005515~protein binding,GO:0008094~DNA-dependent ATPase activity,GO:0008135~translation factor activity, RNA binding,GO:0008353~RNA polymerase II carboxy-terminal domain kinase activity,GO:0046872~metal ion binding,GO:0047485~protein N-terminus binding,
GBGT1	globoside alpha-1,3-N-acetylgalactosaminyltransferase 1(GBGT1)	Homo sapiens	GO:0016758~transferase activity, transferring hexosyl groups,GO:0046872~metal ion binding,GO:0047277~globoside alpha-N-acetylgalactosaminyltransferase activity,
GMEB1	glucocorticoid modulatory element binding protein 1(GMEB1)	Homo sapiens	GO:0000978~RNA polymerase II core promoter proximal region sequence-specific DNA binding,GO:0001077~transcriptional activator activity, RNA polymerase II core promoter proximal region sequence-specific binding,GO:0003677~DNA binding,GO:0003713~transcription coactivator activity,GO:0046872~metal ion binding,
GCNT7	glucosaminyl (N-acetyl) transferase family member 7(GCNT7)	Homo sapiens	GO:0008375~acetylglucosaminyltransferase activity,
GRIN2C	glutamate ionotropic receptor NMDA type subunit 2C(GRIN2C)	Homo sapiens	GO:0004970~ionotropic glutamate receptor activity,GO:0004972~NMDA glutamate receptor activity,GO:0005088~Ras guanyl-nucleotide exchange factor activity,GO:0005234~extracellular-glutamate-gated ion channel activity,GO:0005261~cation channel activity,GO:0005515~protein binding,
GRIK1	glutamate ionotropic receptor kainate type subunit 1(GRIK1)	Homo sapiens	GO:0004970~ionotropic glutamate receptor activity,GO:0005234~extracellular-glutamate-gated ion channel activity,GO:0015276~ligand-gated ion channel activity,GO:0015277~kainate selective glutamate receptor activity,
GRM1	glutamate metabotropic receptor 1(GRM1)	Homo sapiens	GO:0004930~G-protein coupled receptor activity,GO:0005515~protein binding,GO:0008066~glutamate receptor activity,
GRM2	glutamate metabotropic receptor 2(GRM2)	Homo sapiens	GO:0001641~group II metabotropic glutamate receptor activity,GO:0004930~G-protein coupled receptor activity,GO:0005246~calcium channel regulator activity,GO:0005515~protein binding,GO:0008066~glutamate receptor activity,
GRM3	glutamate metabotropic receptor 3(GRM3)	Homo sapiens	GO:0001641~group II metabotropic glutamate receptor activity,GO:0004930~G-protein coupled receptor activity,GO:0005246~calcium channel regulator activity,GO:0008066~glutamate receptor activity,
GRM4	glutamate metabotropic receptor 4(GRM4)	Homo sapiens	GO:0001642~group III metabotropic glutamate receptor activity,GO:0004930~G-protein coupled receptor activity,GO:0008066~glutamate receptor activity,
GSTA4	glutathione S-transferase alpha 4(GSTA4)	Homo sapiens	GO:0004364~glutathione transferase activity,GO:0005515~protein binding,GO:0042802~identical protein binding,GO:0042803~protein homodimerization activity,
GSTM4	glutathione S-transferase mu 4(GSTM4)	Homo sapiens	GO:0004364~glutathione transferase activity,GO:0005515~protein binding,GO:0019899~enzyme binding,GO:0042803~protein homodimerization activity,GO:0043295~glutathione binding,
GSTO2	glutathione S-transferase omega 2(GSTO2)	Homo sapiens	GO:0004364~glutathione transferase activity,GO:0005515~protein binding,GO:0016491~oxidoreductase activity,GO:0042802~identical protein binding,GO:0045174~glutathione dehydrogenase (ascorbate) activity,GO:0050610~methylarsonate reductase activity,
GLDC	glycine decarboxylase(GLDC)	Homo sapiens	GO:0004375~glycine dehydrogenase (decarboxylating) activity,GO:0009055~electron carrier activity,GO:0016594~glycine binding,GO:0016829~lyase activity,GO:0019899~enzyme binding,GO:0030170~pyridoxal phosphate binding,GO:0046983~protein dimerization activity,
GLRA1	glycine receptor alpha 1(GLRA1)	Homo sapiens	GO:0005515~protein binding,GO:0008270~zinc ion binding,GO:0016594~glycine binding,GO:0016934~extracellular-glycine-gated chloride channel activity,GO:0022824~transmitter-gated ion channel activity,GO:0030977~taurine binding,
GYS1	glycogen synthase 1(GYS1)	Homo sapiens	GO:0004373~glycogen (starch) synthase activity,GO:0005515~protein binding,GO:0005536~glucose binding,GO:0019901~protein kinase binding,GO:0061547~glycogen synthase activity, transferring glucose-1-phosphate,
GP5	glycoprotein V platelet(GP5)	Homo sapiens	GO:0005515~protein binding,
GGTA1P	glycoprotein, alpha-galactosyltransferase 1 pseudogene(GGTA1P)	Homo sapiens	GO:0003840~gamma-glutamyltransferase activity,GO:0016758~transferase activity, transferring hexosyl groups,
GOLGA8C	golgin A8 family member O(GOLGA8O)	Homo sapiens	GO:0005515~protein binding,
GADD45A	growth arrest and DNA damage inducible alpha(GADD45A)	Homo sapiens	GO:0001047~core promoter binding,GO:0005515~protein binding,
GADD45B	growth arrest and DNA damage inducible beta(GADD45B)	Homo sapiens	GO:0005515~protein binding,
GADD45C	growth arrest and DNA damage inducible gamma(GADD45G)	Homo sapiens	GO:0005515~protein binding,
GDF10	growth differentiation factor 10(GDF10)	Homo sapiens	GO:0005125~cytokine activity,GO:0005160~transforming growth factor beta receptor binding,GO:0008083~growth factor activity,
GDF15	growth differentiation factor 15(GDF15)	Homo sapiens	GO:0005125~cytokine activity,GO:0005160~transforming growth factor beta receptor binding,GO:0005515~protein binding,GO:0008083~growth factor activity,
GAMT	guanidinoacetate N-methyltransferase(GAMT)	Homo sapiens	GO:0008168~methyltransferase activity,GO:0030731~guanidinoacetate N-methyltransferase activity,
GUCA1B	guanylate cyclase activator 1B(GUCA1B)	Homo sapiens	GO:0005198~structural molecule activity,GO:0005509~calcium ion binding,GO:0008048~calcium sensitive guanylate cyclase activator activity,
HRK	harakiri, BCL2 interacting protein(HRK)	Homo sapiens	GO:0005515~protein binding,
HSPA12B	heat shock protein family A (Hsp70) member 12B(HSPA12B)	Homo sapiens	GO:0005515~protein binding,GO:0005524~ATP binding,
HSPB7	heat shock protein family B (small) member 7(HSPB7)	Homo sapiens	GO:0005515~protein binding,GO:0008022~protein C-terminus binding,GO:0031005~filamin binding,
HSPB8	heat shock protein family B (small) member 8(HSPB8)	Homo sapiens	GO:0004672~protein kinase activity,GO:0005515~protein binding,GO:0042802~identical protein binding,
HSPB9	heat shock protein family B (small) member 9(HSPB9)	Homo sapiens	GO:0005515~protein binding,
HSF4	heat shock transcription factor 4(HSF4)	Homo sapiens	GO:0003700~transcription factor activity, sequence-specific DNA binding,GO:0003714~transcription corepressor activity,GO:0019903~protein phosphatase binding,GO:0043565~sequence-specific DNA binding,
HHIP	hedgehog interacting protein(HHIP)	Homo sapiens	GO:0005515~protein binding,GO:0008270~zinc ion binding,GO:0016901~oxidoreductase activity, acting on the CH-OH group of donors, quinone or similar compound as acceptor,GO:0048038~quinone binding,GO:0097108~hedgehog family protein binding,
HSH2D	hematopoietic SH2 domain containing(HSH2D)	Homo sapiens	GO:0005070~SH3/SH2 adaptor activity,GO:0005515~protein binding,

HMOX1	heme oxygenase 1(HMOX1)	Homo sapiens	GO:0004392~heme oxygenase (decyclizing) activity,GO:0004630~phospholipase D activity,GO:0004871~signal transducer activity,GO:0005515~protein binding,GO:0019899~enzyme binding,GO:0020037~heme binding,GO:0042803~protein homodimerization activity,GO:0046872~metal ion binding,
HMCN2	hemicentin 2(HMCN2)	Homo sapiens	GO:0005509~calcium ion binding,
HS6ST1	heparan sulfate 6-O-sulfotransferase 1(HS6ST1)	Homo sapiens	GO:0008146~sulfotransferase activity,GO:0017095~heparan sulfate 6-O-sulfotransferase activity,
HSPG2	heparan sulfate proteoglycan 2(HSPG2)	Homo sapiens	GO:0005509~calcium ion binding,GO:0005515~protein binding,GO:0008022~protein C-terminus binding,
HBEGF	heparin binding EGF like growth factor(HBEGF)	Homo sapiens	GO:0004713~protein tyrosine kinase activity,GO:0005088~Ras guanyl-nucleotide exchange factor activity,GO:0005154~epidermal growth factor receptor binding,GO:0008083~growth factor activity,GO:0008201~heparin binding,GO:0046934~phosphatidylinositol-4,5-bisphosphate 3-kinase activity,
HAVCR1	hepatitis A virus cellular receptor 1(HAVCR1)	Homo sapiens	GO:0001618~virus receptor activity,
HAVCR2	hepatitis A virus cellular receptor 2(HAVCR2)	Homo sapiens	GO:0005515~protein binding,GO:0046872~metal ion binding,
HPN	hepsin(HPN)	Homo sapiens	GO:0004252~serine-type endopeptidase activity,GO:0005044~scavenger receptor activity,GO:0005515~protein binding,GO:0008233~peptidase activity,GO:0008236~serine-type peptidase activity,GO:0015269~calcium-activated potassium channel activity,GO:0070008~serine-type exopeptidase activity,
HES2	hes family bHLH transcription factor 2(HES2)	Homo sapiens	GO:0003690~double-stranded DNA binding,GO:0008134~transcription factor binding,GO:0046983~protein dimerization activity,
HEY1	hes related family bHLH transcription factor with YRPW motif 1(HEY1)	Homo sapiens	GO:0000983~transcription factor activity, RNA polymerase II core promoter sequence-specific,GO:0000988~transcription factor activity, protein binding,GO:0003677~DNA binding,GO:0003700~transcription factor activity, sequence-specific DNA binding,GO:0005515~protein binding,GO:0008134~transcription factor binding,GO:0035939~microsatellite binding,GO:0046983~protein dimerization activity,
H6PD	hexose-6-phosphate dehydrogenase/glucose 1-dehydrogenase(H6PD)	Homo sapiens	GO:0004345~glucose-6-phosphate dehydrogenase activity,GO:0017057~6-phosphogluconolactonase activity,GO:0030246~carbohydrate binding,GO:0047936~glucose 1-dehydrogenase [NAD(P)] activity,GO:0050661~NADP binding,
HMG20B	high mobility group 20B(HMG20B)	Homo sapiens	GO:0003677~DNA binding,GO:0004407~histone deacetylase activity,GO:0005515~protein binding,GO:0046982~protein heterodimerization activity,
HIST1H2A	histone cluster 1 H2A family member b(HIST1H2AB)	Homo sapiens	GO:0003677~DNA binding,GO:0046982~protein heterodimerization activity,
HIST1H3A	histone cluster 1 H3 family member a(HIST1H3A)	Homo sapiens	binding,GO:0042393~histone binding,GO:0046982~protein heterodimerization activity,GO:0098641~cadherin binding involved in cell-cell adhesion,
HIST1H3J	histone cluster 1 H3 family member j(HIST1H3J)	Homo sapiens	binding,GO:0042393~histone binding,GO:0046982~protein heterodimerization activity,GO:0098641~cadherin binding involved in cell-cell adhesion,
HIST1H4D	histone cluster 1 H4 family member d(HIST1H4D)	Homo sapiens	GO:0003677~DNA binding,GO:0005515~protein binding,GO:0019904~protein domain specific binding,GO:0042393~histone binding,GO:0044822~poly(A) RNA binding,GO:0046982~protein heterodimerization activity,
HIST2H4B	histone cluster 2 H4 family member b(HIST2H4B)	Homo sapiens	GO:0003677~DNA binding,GO:0005515~protein binding,GO:0019904~protein domain specific binding,GO:0042393~histone binding,GO:0044822~poly(A) RNA binding,GO:0046982~protein heterodimerization activity,
HIST3H2A	histone cluster 3 H2A(HIST3H2A)	Homo sapiens	GO:0003677~DNA binding,GO:0046982~protein heterodimerization activity,
HIST3H2B	histone cluster 3 H2B family member b(HIST3H2BB)	Homo sapiens	GO:0003677~DNA binding,GO:0046982~protein heterodimerization activity,
HDAC11	histone deacetylase 11(HDAC11)	Homo sapiens	GO:0004407~histone deacetylase activity,GO:0008134~transcription factor binding,GO:0016787~hydrolase activity,GO:0032041~NAD-dependent histone deacetylase activity (H3-K14 specific),
HDAC4	histone deacetylase 4(HDAC4)	Homo sapiens	GO:0001025~RNA polymerase III transcription factor binding,GO:0001047~core promoter binding,GO:0003682~chromatin binding,GO:0003714~transcription corepressor activity,GO:0004407~histone deacetylase activity,GO:0005515~protein binding,GO:0008134~transcription factor binding,GO:0008270~zinc ion binding,GO:0019901~protein kinase binding,GO:0030955~potassium ion binding,GO:0032041~NAD-dependent histone deacetylase activity (H3-K14 specific),GO:0033558~protein deacetylase activity,GO:0033613~activating transcription factor binding,GO:0042826~histone deacetylase binding,GO:0043565~sequence-specific DNA binding,GO:0044212~transcription regulatory region DNA binding,GO:0070491~repressing transcription factor binding,
HDAC6	histone deacetylase 6(HDAC6)	Homo sapiens	GO:0001047~core promoter binding,GO:0003779~actin binding,GO:0004407~histone deacetylase activity,GO:0005515~protein binding,GO:0008013~beta-catenin binding,GO:0008017~microtubule binding,GO:0008270~zinc ion binding,GO:0016787~hydrolase activity,GO:0019899~enzyme binding,GO:0031593~polyubiquitin binding,GO:0031625~ubiquitin protein ligase binding,GO:0032041~NAD-dependent histone deacetylase activity (H3-K14 specific),GO:0042826~histone deacetylase binding,GO:0042903~tubulin deacetylase activity,GO:0043014~alpha-tubulin binding,GO:0048156~tau protein binding,GO:0048487~beta-tubulin binding,GO:0051787~misfolded protein binding,GO:0051879~Hsp90 protein binding,GO:0070840~dynein complex binding,
HOMEZ	homeobox and leucine zipper encoding(HOMEZ)	Homo sapiens	GO:0003677~DNA binding,GO:0003700~transcription factor activity, sequence-specific DNA binding,GO:0003714~transcription corepressor activity,
HIVEP2	human immunodeficiency virus type I enhancer binding protein 2(HIVEP2)	Homo sapiens	GO:0003677~DNA binding,GO:0003700~transcription factor activity, sequence-specific DNA binding,GO:0043565~sequence-specific DNA binding,GO:0044212~transcription regulatory region DNA binding,GO:0046872~metal ion binding,
HIP1	huntingtin interacting protein 1(HIP1)	Homo sapiens	GO:0005154~epidermal growth factor receptor binding,GO:0005200~structural constituent of cytoskeleton,GO:0005515~protein binding,GO:0005546~phosphatidylinositol-4,5-bisphosphate binding,GO:0030276~clathrin binding,GO:0032051~clathrin light chain binding,GO:0032266~phosphatidylinositol-3-phosphate binding,GO:0035091~phosphatidylinositol binding,GO:0035254~glutamate receptor binding,GO:0035612~AP-2 adaptor complex binding,GO:0035615~clathrin adaptor activity,GO:0042803~protein homodimerization activity,GO:0043325~phosphatidylinositol-3,4-bisphosphate binding,GO:0046982~protein heterodimerization activity,GO:0051015~actin filament binding,GO:0080025~phosphatidylinositol-3,5-bisphosphate binding,
HMMR	hyaluronan mediated motility receptor(HMMR)	Homo sapiens	GO:0005515~protein binding,GO:0005540~hyaluronic acid binding,
HAS1	hyaluronan synthase 1(HAS1)	Homo sapiens	GO:0050501~hyaluronan synthase activity,
HYAL3	hyaluronoglucosaminidase 3(HYAL3)	Homo sapiens	GO:0001618~virus receptor activity,GO:0004415~hyalurononglucosaminidase activity,GO:0005515~protein binding,GO:0033906~hyaluronoglucuronidase activity,
HAGH	hydroxyacylglutathione hydrolase(HAGH)	Homo sapiens	GO:0004416~hydroxyacylglutathione hydrolase activity,GO:0005515~protein binding,GO:0046872~metal ion binding,
HSD11B2	hydroxysteroid 11-beta dehydrogenase 2(HSD11B2)	Homo sapiens	GO:0003845~11-beta-hydroxysteroid dehydrogenase [NAD(P)] activity,GO:0005496~steroid binding,GO:0051287~NAD binding,

ILVBL	ilvB acetolactate synthase like(ILVBL)	Homo sapiens	GO:0000287~magnesium ion binding,GO:0003824~catalytic activity,GO:0005515~protein binding,GO:0016740~transferase activity,GO:0030976~thiamine pyrophosphate binding,
IGSF9	immunoglobulin superfamily member 9(IGSF9)	Homo sapiens	GO:0098632~protein binding involved in cell-cell adhesion,
IGFN1	immunoglobulin-like and fibronectin type III domain containing 1(IGFN1)	Homo sapiens	GO:0005515~protein binding,
INHBB	inhibin beta B subunit(INHBB)	Homo sapiens	GO:0005125~cytokine activity,GO:0005160~transforming growth factor beta receptor binding,GO:0005179~hormone activity,GO:0005515~protein binding,GO:0008083~growth factor activity,GO:0042803~protein homodimerization activity,GO:0046789~host cell surface receptor binding,
IMPDH2	inosine monophosphate dehydrogenase 2(IMPDH2)	Homo sapiens	GO:0000166~nucleotide binding,GO:0003677~DNA binding,GO:0003723~RNA binding,GO:0003824~catalytic activity,GO:0003938~IMP dehydrogenase activity,GO:0005515~protein binding,GO:0016491~oxidoreductase activity,GO:0046872~metal ion binding,
ISYNA1	inositol-3-phosphate synthase 1(ISYNA1)	Homo sapiens	GO:0004512~inositol-3-phosphate synthase activity,GO:0005515~protein binding,
ITPKA	inositol-trisphosphate 3-kinase A(ITPKA)	Homo sapiens	GO:0004683~calmodulin-dependent protein kinase activity,GO:0005516~calmodulin binding,GO:0005524~ATP binding,GO:0008440~inositol-1,4,5-trisphosphate 3-kinase activity,GO:0048365~Rac GTPase binding,
INSL4	insulin like 4(INSL4)	Homo sapiens	GO:0005102~receptor binding,GO:0005159~insulin-like growth factor receptor binding,GO:0005179~hormone activity,
IRS1	insulin receptor substrate 1(IRS1)	Homo sapiens	GO:0004871~signal transducer activity,GO:0005068~transmembrane receptor protein tyrosine kinase adaptor activity,GO:0005080~protein kinase C binding,GO:0005088~Ras guanyl-nucleotide exchange factor activity,GO:0005158~insulin receptor binding,GO:0005159~insulin-like growth factor receptor binding,GO:0005515~protein binding,GO:0016303~1-phosphatidylinositol-3-kinase activity,GO:0042169~SH2 domain binding,GO:0043548~phosphatidylinositol 3-kinase binding,GO:0046934~phosphatidylinositol-4,5-bisphosphate 3-kinase activity,
IRS2	insulin receptor substrate 2(IRS2)	Homo sapiens	GO:0004871~signal transducer activity,GO:0005088~Ras guanyl-nucleotide exchange factor activity,GO:0005158~insulin receptor binding,GO:0005515~protein binding,GO:0016303~1-phosphatidylinositol-3-kinase activity,GO:0019901~protein kinase binding,GO:0019903~protein phosphatase binding,GO:0019904~protein domain specific binding,GO:0043548~phosphatidylinositol 3-kinase binding,GO:0046934~phosphatidylinositol-4,5-bisphosphate 3-kinase activity,GO:0071889~14-3-3 protein binding,
INTS3	integrator complex subunit 3(INTS3)	Homo sapiens	GO:0005515~protein binding,
ITGB4	integrin subunit beta 4(ITGB4)	Homo sapiens	GO:0001664~G-protein coupled receptor binding,GO:0004872~receptor activity,GO:0005515~protein binding,GO:0031994~insulin-like growth factor I binding,GO:0038132~neuregulin binding,
IFI35	interferon induced protein 35(IFI35)	Homo sapiens	GO:0005515~protein binding,
IFIT1	interferon induced protein with tetratricopeptide repeats 1(IFIT1)	Homo sapiens	GO:0003723~RNA binding,GO:0005515~protein binding,
IRAK1BP1	interleukin 1 receptor associated kinase 1 binding protein 1(IRAK1BP1)	Homo sapiens	GO:0005515~protein binding,
IL1R1	interleukin 1 receptor type 1(IL1R1)	Homo sapiens	GO:0002020~protease binding,GO:0004871~signal transducer activity,GO:0004888~transmembrane signaling receptor activity,GO:0004908~interleukin-1 receptor activity,GO:0004909~interleukin-1, Type I, activating receptor activity,GO:0005161~platelet-derived growth factor receptor binding,GO:0005515~protein binding,GO:0019966~interleukin-1 binding,
IL11	interleukin 11(IL11)	Homo sapiens	GO:0005125~cytokine activity,GO:0005142~interleukin-11 receptor binding,GO:0005515~protein binding,GO:0008083~growth factor activity,
IL12RB2	interleukin 12 receptor subunit beta 2(IL12RB2)	Homo sapiens	GO:0004896~cytokine receptor activity,GO:0019901~protein kinase binding,
IL17RE	interleukin 17 receptor E(IL17RE)	Homo sapiens	GO:0005515~protein binding,GO:0030368~interleukin-17 receptor activity,
IL18R1	interleukin 18 receptor 1(IL18R1)	Homo sapiens	GO:0004872~receptor activity,GO:0004908~interleukin-1 receptor activity,GO:0005515~protein binding,GO:0042008~interleukin-18 receptor activity,
IL20RB	interleukin 20 receptor subunit beta(IL20RB)	Homo sapiens	GO:0004896~cytokine receptor activity,GO:0042015~interleukin-20 binding,
IFFO2	intermediate filament family orphan 2(IFFO2)	Homo sapiens	GO:0005198~structural molecule activity,
INF2	inverted formin, FH2 and WH2 domain containing(INF2)	Homo sapiens	GO:0003779~actin binding,GO:0017048~Rho GTPase binding,
IVD	isovaleryl-CoA dehydrogenase(IVD)	Homo sapiens	GO:0000062~fatty-acyl-CoA binding,GO:0003995~acyl-CoA dehydrogenase activity,GO:0008470~isovaleryl-CoA dehydrogenase activity,GO:0009055~electron carrier activity,GO:0016627~oxidoreductase activity, acting on the CH-CH group of donors,GO:0050660~flavin adenine dinucleotide binding,GO:0052890~oxidoreductase activity, acting on the CH-CH group of donors, with a flavin as acceptor,
JAG2	jagged 2(JAG2)	Homo sapiens	GO:0005112~Notch binding,GO:0005509~calcium ion binding,GO:0005515~protein binding,GO:0008083~growth factor activity,
KPTN	kaptin, actin binding protein(KPTN)	Homo sapiens	GO:0003779~actin binding,
KATNB1	katanin regulatory subunit B1(KATNB1)	Homo sapiens	GO:0008017~microtubule binding,GO:0008568~microtubule-severing ATPase activity,GO:0045502~dynein binding,GO:0046982~protein heterodimerization activity,
KAZN	kazrin, periplakin interacting protein(KAZN)	Homo sapiens	GO:0005515~protein binding,
KLHL11	kelch like family member 11(KLHL11)	Homo sapiens	GO:0031625~ubiquitin protein ligase binding,
KLHL22	kelch like family member 22(KLHL22)	Homo sapiens	GO:0004842~ubiquitin-protein transferase activity,GO:0005515~protein binding,
KLHL30	kelch like family member 30(KLHL30)	Homo sapiens	GO:0004842~ubiquitin-protein transferase activity,
KLHL35	kelch like family member 35(KLHL35)	Homo sapiens	GO:0005515~protein binding,
KLHL6	kelch like family member 6(KLHL6)	Homo sapiens	GO:0005515~protein binding,
KRT17	keratin 17(KRT17)	Homo sapiens	GO:0005198~structural molecule activity,GO:0005200~structural constituent of cytoskeleton,GO:0005515~protein binding,GO:0032395~MHC class II receptor activity,GO:0042289~MHC class II protein binding,
KHK	ketohexokinase(KHK)	Homo sapiens	GO:0004454~ketohexokinase activity,GO:0005515~protein binding,GO:0005524~ATP binding,
KLRB1	killer cell lectin like receptor B1(KLRB1)	Homo sapiens	GO:0004888~transmembrane signaling receptor activity,GO:0030246~carbohydrate binding,
KLRD1	killer cell lectin like receptor D1(KLRD1)	Homo sapiens	GO:0004888~transmembrane signaling receptor activity,GO:0005515~protein binding,GO:0023024~MHC class I protein complex binding,GO:0023030~MHC class Ib protein binding, via antigen binding groove,GO:0030246~carbohydrate binding,GO:1990405~protein antigen binding,
KIRREL2	kin of IRRE like 2 (Drosophila)(KIRREL2)	Homo sapiens	GO:0005515~protein binding,
KSR2	kinase suppressor of ras 2(KSR2)	Homo sapiens	GO:0004672~protein kinase activity,GO:0004674~protein serine/threonine kinase activity,GO:0005524~ATP binding,GO:0046872~metal ion binding,
KIF17	kinesin family member 17(KIF17)	Homo sapiens	GO:0003777~microtubule motor activity,GO:0005524~ATP binding,GO:0008017~microtubule binding,GO:0008574~ATP-dependent microtubule motor activity, plus-end-directed,
KIF5A	kinesin family member 5A(KIF5A)	Homo sapiens	GO:0003774~motor activity,GO:0003777~microtubule motor activity,GO:0005515~protein binding,GO:0005524~ATP binding,GO:0008017~microtubule binding,GO:0008574~ATP-dependent microtubule motor activity, plus-end-directed,GO:0019894~kinesin binding,

KIFC2	kinesin family member C2(KIFC2)	Homo sapiens	GO:0003777~microtubule motor activity,GO:0005524~ATP binding,GO:0008017~microtubule binding,GO:0016887~ATPase activity,
KLB	klotho beta(KLB)	Homo sapiens	GO:0004553~hydrolase activity, hydrolyzing O-glycosyl compounds,GO:0004871~signal transducer activity,GO:0005088~Ras guanyl-nucleotide exchange factor activity,GO:0005104~fibroblast growth factor receptor binding,GO:0005515~protein binding,GO:0008422~beta-glucosidase activity,GO:0016303~1-phosphatidylinositol-3-kinase activity,GO:0017134~fibroblast growth factor binding,GO:0046934~phosphatidylinositol-4,5-bisphosphate 3-kinase activity,
LAMA5	laminin subunit alpha 5(LAMA5)	Homo sapiens	GO:0005178~integrin binding,GO:0005198~structural molecule activity,
LSS	lanosterol synthase (2,3-oxidosqualene-lanosterol cyclase)(LSS)	Homo sapiens	GO:0000250~lanosterol synthase activity,GO:0016866~intramolecular transferase activity,
LATS2	large tumor suppressor kinase 2(LATS2)	Homo sapiens	GO:0004674~protein serine/threonine kinase activity,GO:0005515~protein binding,GO:0005524~ATP binding,GO:0046872~metal ion binding,
LAYN	layilin(LAYN)	Homo sapiens	GO:0005540~hyaluronic acid binding,GO:0030246~carbohydrate binding,
LURAP1	leucine rich adaptor protein 1(LURAP1)	Homo sapiens	GO:0005515~protein binding,
LRRC20	leucine rich repeat containing 20(LRRC20)	Homo sapiens	GO:0005515~protein binding,
LRRC45	leucine rich repeat containing 45(LRRC45)	Homo sapiens	GO:0005515~protein binding,
LRRC8A	leucine rich repeat containing 8 family member A(LRRC8A)	Homo sapiens	GO:0005225~volume-sensitive anion channel activity,GO:0005253~anion channel activity,GO:0005515~protein binding,
LRRK1	leucine rich repeat kinase 1(LRRK1)	Homo sapiens	GO:0004672~protein kinase activity,GO:0004674~protein serine/threonine kinase activity,GO:0005515~protein binding,GO:0005524~ATP binding,GO:0005525~GTP binding,GO:0042802~identical protein binding,GO:0046872~metal ion binding,
LRRN2	leucine rich repeat neuronal 2(LRRN2)	Homo sapiens	GO:0004872~receptor activity,
LRRTM4	leucine rich repeat transmembrane neuronal 4(LRRTM4)	Homo sapiens	GO:0004860~protein kinase inhibitor activity,
LRRIQ3	leucine rich repeats and IQ motif containing 3(LRRIQ3)	Homo sapiens	GO:0005515~protein binding,
LSMEM1	leucine rich single-pass membrane protein 1(LSMEM1)	Homo sapiens	GO:0005515~protein binding,
LZTS1	leucine zipper tumor suppressor 1(LZTS1)	Homo sapiens	GO:0003677~DNA binding,GO:0003700~transcription factor activity, sequence-specific DNA binding,GO:0005515~protein binding,GO:0008017~microtubule binding,
LENG8	leukocyte receptor cluster member 8(LENG8)	Homo sapiens	GO:0005515~protein binding,
LIPC	lipase C, hepatic type(LIPC)	Homo sapiens	GO:0004620~phospholipase activity,GO:0004806~triglyceride lipase activity,GO:0008201~heparin binding,GO:0030169~low-density lipoprotein particle binding,GO:0034185~apolipoprotein binding,
LPIN3	lipin 3(LPIN3)	Homo sapiens	GO:0003713~transcription coactivator activity,GO:0008195~phosphatidate phosphatase activity,
LCN12	lipocalin 12(LCN12)	Homo sapiens	GO:0001972~retinoic acid binding,GO:0005215~transporter activity,
KAT14	lysine acetyltransferase 14(KAT14)	Homo sapiens	GO:0004402~histone acetyltransferase activity,GO:0005515~protein binding,GO:0008080~N-acetyltransferase activity,GO:0030274~LIM domain binding,
MST1L	macrophage stimulating 1 like(MST1L)	Homo sapiens	GO:0004252~serine-type endopeptidase activity,
MR1	major histocompatibility complex, class I-related(MR1)	Homo sapiens	GO:0003823~antigen binding,GO:0005515~protein binding,GO:0032393~MHC class I receptor activity,
MDH1B	malate dehydrogenase 1B(MDH1B)	Homo sapiens	GO:0016615~malate dehydrogenase activity,GO:0016616~oxidoreductase activity, acting on the CH-OH group of donors, NAD or NADP as acceptor,GO:0030060~L-malate dehydrogenase activity,
ME3	malic enzyme 3(ME3)	Homo sapiens	GO:0004470~malic enzyme activity,GO:0004471~malate dehydrogenase (decarboxylating) (NAD+) activity,GO:0004473~malate dehydrogenase (decarboxylating) (NADP+) activity,GO:0005509~calcium ion binding,GO:0008948~oxaloacetate decarboxylase activity,GO:0046872~metal ion binding,GO:0048037~cofactor binding,GO:0051287~NAD binding,
MAN1C1	mannosidase alpha class 1C member 1(MAN1C1)	Homo sapiens	GO:0004571~mannosyl-oligosaccharide 1,2-alpha-mannosidase activity,GO:0005509~calcium ion binding,
MGAT3	mannosyl (beta-1,4-)-glycoprotein beta-1,4-N-acetylglucosaminyltransferase(MGAT3)	Homo sapiens	GO:0003830~beta-1,4-mannosylglycoprotein 4-beta-N-acetylglucosaminyltransferase activity,
MMP2	matrix metallopeptidase 2(MMP2)	Homo sapiens	GO:0004222~metalloendopeptidase activity,GO:0004252~serine-type endopeptidase activity,GO:0005515~protein binding,GO:0008237~metallopeptidase activity,GO:0008270~zinc ion binding,
MED10	mediator complex subunit 10(MED10)	Homo sapiens	GO:0001104~RNA polymerase II transcription cofactor activity,GO:0005515~protein binding,GO:0061630~ubiquitin protein ligase activity,
MED6	mediator complex subunit 6(MED6)	Homo sapiens	GO:0001104~RNA polymerase II transcription cofactor activity,GO:0001128~RNA polymerase II transcription coactivator activity involved in preinitiation complex assembly,GO:0003713~transcription coactivator activity,GO:0005515~protein binding,GO:0008134~transcription factor binding,GO:0061630~ubiquitin protein ligase activity,
MATK	megakaryocyte-associated tyrosine kinase(MATK)	Homo sapiens	GO:0004713~protein tyrosine kinase activity,GO:0004715~non-membrane spanning protein tyrosine kinase activity,GO:0005102~receptor binding,GO:0005515~protein binding,GO:0005524~ATP binding,
M1AP	meiosis 1 associated protein(M1AP)	Homo sapiens	GO:0005515~protein binding,GO:0042802~identical protein binding,
MLANA	melan-A(MLANA)	Homo sapiens	GO:0005515~protein binding,
MLPH	melanophilin(MLPH)	Homo sapiens	GO:0003779~actin binding,GO:0005515~protein binding,GO:0017022~myosin binding,GO:0017137~Rab GTPase binding,GO:0046872~metal ion binding,
MEOX1	mesenchyme homeobox 1(MEOX1)	Homo sapiens	GO:0000978~RNA polymerase II core promoter proximal region sequence-specific DNA binding,GO:0001046~core promoter sequence-specific DNA binding,GO:0001077~transcriptional activator activity, RNA polymerase II core promoter proximal region sequence-specific binding,GO:0003677~DNA binding,GO:0003682~chromatin binding,GO:0003700~transcription factor activity, sequence-specific DNA binding,GO:0005515~protein binding,GO:0043565~sequence-specific DNA binding,GO:0071837~HMG box domain binding,
MBLAC2	metallo-beta-lactamase domain containing 2(MBLAC2)	Homo sapiens	GO:0016787~hydrolase activity,GO:0046872~metal ion binding,
MAT1A	methionine adenosyltransferase 1A(MAT1A)	Homo sapiens	GO:0004478~methionine adenosyltransferase activity,GO:0005524~ATP binding,GO:0046872~metal ion binding,GO:0098601~selenomethionine adenosyltransferase activity,
MMAB	methylmalonic aciduria (cobalamin deficiency) cblB type(MMAB)	Homo sapiens	GO:0005524~ATP binding,GO:0008817~cob(I)yrinic acid a,c-diamide adenosyltransferase activity,
METTL16	methyltransferase like 16(METTL16)	Homo sapiens	GO:0008168~methyltransferase activity,GO:0044822~poly(A) RNA binding,GO:0052907~23S rRNA (adenine(1618)-N(6))-methyltransferase activity,
METTL7A	methyltransferase like 7A(METTL7A)	Homo sapiens	GO:0008168~methyltransferase activity,GO:0008757~S-adenosylmethionine-dependent methyltransferase activity,
METTL7B	methyltransferase like 7B(METTL7B)	Homo sapiens	GO:0008168~methyltransferase activity,GO:0008757~S-adenosylmethionine-dependent methyltransferase activity,
MAP1LC3B	microtubule associated protein 1 light chain 3 beta(MAP1LC3B)	Homo sapiens	GO:0005515~protein binding,GO:0008017~microtubule binding,GO:0031625~ubiquitin protein ligase binding,
MAST3	microtubule associated serine/threonine kinase 3(MAST3)	Homo sapiens	GO:0000287~magnesium ion binding,GO:0004674~protein serine/threonine kinase activity,GO:0005515~protein binding,GO:0005524~ATP binding,
MIPEP	mitochondrial intermediate peptidase(MIPEP)	Homo sapiens	GO:0004222~metalloendopeptidase activity,GO:0046872~metal ion binding,

MRM1	mitochondrial rRNA methyltransferase 1(MRM1)	Homo sapiens	GO:0003723~RNA binding,GO:0005515~protein binding,GO:0008173~RNA methyltransferase activity,GO:0044822~poly(A) RNA binding,GO:0070039~rRNA (guanosine-2'-O-)-methyltransferase activity,
MRPS24	mitochondrial ribosomal protein S24(MRPS24)	Homo sapiens	GO:0003735~structural constituent of ribosome,GO:0044822~poly(A) RNA binding,
MAPK11	mitogen-activated protein kinase 11(MAPK11)	Homo sapiens	GO:0004672~protein kinase activity,GO:0004674~protein serine/threonine kinase activity,GO:0004707~MAP kinase activity,GO:0005515~protein binding,GO:0005524~ATP binding,
MAPK4	mitogen-activated protein kinase 4(MAPK4)	Homo sapiens	GO:0004674~protein serine/threonine kinase activity,GO:0004707~MAP kinase activity,GO:0005515~protein binding,GO:0005524~ATP binding,GO:0019901~protein kinase binding,GO:0042803~protein homodimerization activity,GO:0046982~protein heterodimerization activity,
MAPK8IP2	mitogen-activated protein kinase 8 interacting protein 2(MAPK8IP2)	Homo sapiens	GO:0001540~beta-amyloid binding,GO:0005078~MAP-kinase scaffold activity,GO:0005198~structural molecule activity,GO:0005515~protein binding,GO:0016301~kinase activity,GO:0019894~kinesin binding,GO:0019901~protein kinase binding,GO:0030295~protein kinase activator activity,GO:0032403~protein complex binding,
MAP3K10	mitogen-activated protein kinase kinase kinase 10(MAP3K10)	Homo sapiens	GO:0003714~transcription corepressor activity,GO:0004672~protein kinase activity,GO:0004674~protein serine/threonine kinase activity,GO:0004706~JUN kinase kinase kinase activity,GO:0004709~MAP kinase kinase kinase activity,GO:0004713~protein tyrosine kinase activity,GO:0005524~ATP binding,GO:0042803~protein homodimerization activity,GO:0043425~bHLH transcription factor binding,
MAP3K12	mitogen-activated protein kinase kinase kinase 12(MAP3K12)	Homo sapiens	GO:0004672~protein kinase activity,GO:0004674~protein serine/threonine kinase activity,GO:0004709~MAP kinase kinase kinase activity,GO:0005515~protein binding,GO:0005524~ATP binding,GO:0019901~protein kinase binding,GO:0042803~protein homodimerization activity,
MAP3K19	mitogen-activated protein kinase kinase kinase 19(MAP3K19)	Homo sapiens	GO:0004672~protein kinase activity,GO:0004702~receptor signaling protein serine/threonine kinase activity,GO:0005524~ATP binding,
MAP4K2	mitogen-activated protein kinase kinase kinase kinase 2(MAP4K2)	Homo sapiens	GO:0004672~protein kinase activity,GO:0004674~protein serine/threonine kinase activity,GO:0004702~receptor signaling protein serine/threonine kinase activity,GO:0005515~protein binding,GO:0005524~ATP binding,GO:0008349~MAP kinase kinase kinase kinase activity,GO:0031435~mitogen-activated protein kinase kinase kinase binding,
MISP	mitotic spindle positioning(MISP)	Homo sapiens	GO:0003779~actin binding,
MLKL	mixed lineage kinase domain like(MLKL)	Homo sapiens	GO:0004672~protein kinase activity,GO:0005515~protein binding,GO:0005524~ATP binding,GO:0019901~protein kinase binding,GO:0032403~protein complex binding,
MOCOS	molybdenum cofactor sulfurase(MOCOS)	Homo sapiens	GO:0005515~protein binding,GO:0008265~Mo-molybdopterin cofactor sulfurase activity,GO:0016740~transferase activity,GO:0016829~lyase activity,GO:0030151~molybdenum ion binding,GO:0030170~pyridoxal phosphate binding,
MSX2	msh homeobox 2(MSX2)	Homo sapiens	GO:0000977~RNA polymerase II regulatory region sequence-specific DNA binding,GO:0001227~transcriptional repressor activity, RNA polymerase II transcription regulatory region sequence-specific binding,GO:0003712~transcription cofactor activity,GO:0005515~protein binding,GO:0008134~transcription factor binding,GO:0043565~sequence-specific DNA binding,GO:0044212~transcription regulatory region DNA binding,
MUC16	mucin 16, cell surface associated(MUC16)	Homo sapiens	GO:0005515~protein binding,
MUC5AC	mucin 5AC, oligomeric mucus/gel-forming(MUC5AC)	Homo sapiens	GO:0005201~extracellular matrix structural constituent,GO:0030197~extracellular matrix constituent, lubricant activity,
MMRN2	multimerin 2(MMRN2)	Homo sapiens	GO:0005515~protein binding,
MEGF6	multiple EGF like domains 6(MEGF6)	Homo sapiens	GO:0005509~calcium ion binding,GO:0005515~protein binding,
MOBP	myelin-associated oligodendrocyte basic protein(MOBP)	Homo sapiens	GO:0003779~actin binding,GO:0005515~protein binding,GO:0017022~myosin binding,GO:0017137~Rab GTPase binding,GO:0019911~structural constituent of myelin sheath,
MEF2D	myocyte enhancer factor 2D(MEF2D)	Homo sapiens	GO:0000977~RNA polymerase II regulatory region sequence-specific DNA binding,GO:0000978~RNA polymerase II core promoter proximal region sequence-specific DNA binding,GO:0000981~RNA polymerase II transcription factor activity, sequence-specific DNA binding,GO:0001077~transcriptional activator activity, RNA polymerase II core promoter proximal region sequence-specific binding,GO:0003700~transcription factor activity, sequence-specific DNA binding,GO:0005515~protein binding,GO:0033613~activating transcription factor binding,GO:0042803~protein homodimerization activity,GO:0042826~histone deacetylase binding,GO:0046982~protein heterodimerization activity,
MYOD1	myogenic differentiation 1(MYOD1)	Homo sapiens	GO:0000977~RNA polymerase II regulatory region sequence-specific DNA binding,GO:0000978~RNA polymerase II core promoter proximal region sequence-specific DNA binding,GO:0001047~core promoter binding,GO:0001228~transcriptional activator activity, RNA polymerase II transcription regulatory region sequence-specific binding,GO:0003682~chromatin binding,GO:0003705~transcription factor activity, RNA polymerase II distal enhancer sequence-specific binding,GO:0003713~transcription coactivator activity,GO:0005515~protein binding,GO:0008134~transcription factor binding,GO:0031490~chromatin DNA binding,GO:0031625~ubiquitin protein ligase binding,GO:0035257~nuclear hormone receptor binding,GO:0046982~protein heterodimerization activity,GO:0070888~E-box binding,
MYO1C	myosin IC(MYO1C)	Homo sapiens	GO:0000146~microfilament motor activity,GO:0003774~motor activity,GO:0003779~actin binding,GO:0005102~receptor binding,GO:0005515~protein binding,GO:0005516~calmodulin binding,GO:0005524~ATP binding,GO:0008022~protein C-terminus binding,GO:0017160~Ral GTPase binding,GO:0030898~actin-dependent ATPase activity,
MYO5C	myosin VC(MYO5C)	Homo sapiens	GO:0003774~motor activity,GO:0003779~actin binding,GO:0005516~calmodulin binding,GO:0005524~ATP binding,
MYO19	myosin XIX(MYO19)	Homo sapiens	GO:0003774~motor activity,GO:0003779~actin binding,GO:0005524~ATP binding,GO:0016887~ATPase activity,GO:0032027~myosin light chain binding,GO:0060002~plus-end directed microfilament motor activity,
MYO15A	myosin XVA(MYO15A)	Homo sapiens	GO:0003774~motor activity,GO:0003779~actin binding,GO:0005516~calmodulin binding,GO:0005524~ATP binding,
MYO18A	myosin XVIIIA(MYO18A)	Homo sapiens	GO:0003677~DNA binding,GO:0003774~motor activity,GO:0005515~protein binding,GO:0005524~ATP binding,GO:0008094~DNA-dependent ATPase activity,GO:0016887~ATPase activity,GO:0043531~ADP binding,GO:0044822~poly(A) RNA binding,GO:0051015~actin filament binding,
MYH16	myosin heavy chain 16 pseudogene(MYH16)	Homo sapiens	GO:0003774~motor activity,
MYH3	myosin heavy chain 3(MYH3)	Homo sapiens	GO:0000146~microfilament motor activity,GO:0003774~motor activity,GO:0005516~calmodulin binding,GO:0005524~ATP binding,GO:0017018~myosin phosphatase activity,GO:0042623~ATPase activity, coupled,GO:0051015~actin filament binding,
MYLK2	myosin light chain kinase 2(MYLK2)	Homo sapiens	GO:0004683~calmodulin-dependent protein kinase activity,GO:0004687~myosin light chain kinase activity,GO:0005515~protein binding,GO:0005516~calmodulin binding,GO:0005524~ATP binding,
MYLK3	myosin light chain kinase 3(MYLK3)	Homo sapiens	GO:0004683~calmodulin-dependent protein kinase activity,GO:0004687~myosin light chain kinase activity,GO:0005524~ATP binding,

MYLK	myosin light chain kinase(MYLK)	Homo sapiens	GO:0003779~actin binding,GO:0004687~myosin light chain kinase activity,GO:0005515~protein binding,GO:0005516~calmodulin binding,GO:0005524~ATP binding,GO:0016301~kinase activity,GO:0046872~metal ion binding,
NPR1	natriuretic peptide receptor 1(NPR1)	Homo sapiens	GO:0000166~nucleotide binding,GO:0004016~adenylate cyclase activity,GO:0004383~guanylate cyclase activity,GO:0004672~protein kinase activity,GO:0005524~ATP binding,GO:0005525~GTP binding,GO:0008528~G-protein coupled peptide receptor activity,GO:0016849~phosphorus-oxygen lyase activity,GO:0016941~natriuretic peptide receptor activity,GO:0017046~peptide hormone binding,GO:0019901~protein kinase binding,GO:0042562~hormone binding,
NCAM1	neural cell adhesion molecule 1(NCAM1)	Homo sapiens	GO:0001618~virus receptor activity,GO:0005088~Ras guanyl-nucleotide exchange factor activity,
NPDC1	neural proliferation, differentiation and control 1(NPDC1)	Homo sapiens	GO:0005515~protein binding,
NBEAL2	neurobeachin like 2(NBEAL2)	Homo sapiens	GO:0005543~phospholipid binding,
NEFH	neurofilament heavy polypeptide(NEFH)	Homo sapiens	GO:0005198~structural molecule activity,GO:0005200~structural constituent of cytoskeleton,GO:0008017~microtubule binding,GO:0019894~kinesin binding,GO:0019901~protein kinase binding,GO:0030674~protein binding, bridging,GO:0045502~dynein binding,
NPTXR	neuronal pentraxin receptor(NPTXR)	Homo sapiens	GO:0046872~metal ion binding,
NYAP2	neuronal tyrosine-phosphorylated phosphoinositide-3-kinase adaptor 2(NYAP2)	Homo sapiens	GO:0005515~protein binding,
NRP2	neuropilin 2(NRP2)	Homo sapiens	GO:0004872~receptor activity,GO:0005021~vascular endothelial growth factor-activated receptor activity,GO:0008201~heparin binding,GO:0017154~semaphorin receptor activity,GO:0019838~growth factor binding,GO:0019955~cytokine binding,GO:0046872~metal ion binding,
NRTN	neurturin(NRTN)	Homo sapiens	GO:0005088~Ras guanyl-nucleotide exchange factor activity,GO:0005102~receptor binding,GO:0008083~growth factor activity,
NCF2	neutrophil cytosolic factor 2(NCF2)	Homo sapiens	GO:0005515~protein binding,GO:0008022~protein C-terminus binding,GO:0009055~electron carrier activity,GO:0016175~superoxide-generating NADPH oxidase activity,GO:0048365~Rac GTPase binding,
NEXN	nexilin F-actin binding protein(NEXN)	Homo sapiens	GO:0008307~structural constituent of muscle,GO:0051015~actin filament binding,
NMRK1	nicotinamide riboside kinase 1(NMRK1)	Homo sapiens	GO:0005515~protein binding,GO:0005524~ATP binding,GO:0016301~kinase activity,GO:0046872~metal ion binding,GO:0050262~ribosylnicotinamide kinase activity,
NOCT	nocturnin(NOCT)	Homo sapiens	GO:0003700~transcription factor activity, sequence-specific DNA binding,GO:0003729~mRNA binding,GO:0004535~poly(A)-specific ribonuclease activity,GO:0046872~metal ion binding,
NOTCH3	notch 3(NOTCH3)	Homo sapiens	GO:0004872~receptor activity,GO:0005509~calcium ion binding,GO:0005515~protein binding,GO:0019899~enzyme binding,GO:0098641~cadherin binding involved in cell-cell adhesion,
NTHL1	nth like DNA glycosylase 1(NTHL1)	Homo sapiens	GO:0000703~oxidized pyrimidine nucleobase lesion DNA N-glycosylase activity,GO:0003677~DNA binding,GO:0003690~double-stranded DNA binding,GO:0003824~catalytic activity,GO:0003906~DNA-(apurinic or apyrimidinic site) lyase activity,GO:0004519~endonuclease activity,GO:0005515~protein binding,GO:0008534~oxidized purine nucleobase lesion DNA N-glycosylase activity,GO:0019104~DNA N-glycosylase activity,GO:0046872~metal ion binding,GO:0051539~4 iron, 4 sulfur cluster binding,
NFATC4	nuclear factor of activated T-cells 4(NFATC4)	Homo sapiens	GO:0000978~RNA polymerase II core promoter proximal region sequence-specific DNA binding,GO:0001078~transcriptional repressor activity, RNA polymerase II core promoter proximal region sequence-specific binding,GO:0001227~transcriptional repressor activity, RNA polymerase II transcription regulatory region sequence-specific binding,GO:0003677~DNA binding,GO:0003700~transcription factor activity, sequence-specific DNA binding,GO:0003713~transcription coactivator activity,GO:0005515~protein binding,GO:0008134~transcription factor binding,GO:0042975~peroxisome proliferator activated receptor binding,
NRBP2	nuclear receptor binding protein 2(NRBP2)	Homo sapiens	GO:0004672~protein kinase activity,GO:0004674~protein serine/threonine kinase activity,GO:0005524~ATP binding,
NR1D2	nuclear receptor subfamily 1 group D member 2(NR1D2)	Homo sapiens	GO:0001046~core promoter sequence-specific DNA binding,GO:0003700~transcription factor activity, sequence-specific DNA binding,GO:0003707~steroid hormone receptor activity,GO:0004879~RNA polymerase II transcription factor activity, ligand-activated sequence-specific DNA binding,GO:0005515~protein binding,GO:0008270~zinc ion binding,GO:0043565~sequence-specific DNA binding,
NR1H3	nuclear receptor subfamily 1 group H member 3(NR1H3)	Homo sapiens	GO:0000978~RNA polymerase II core promoter proximal region sequence-specific DNA binding,GO:0001077~transcriptional activator activity, RNA polymerase II core promoter proximal region sequence-specific binding,GO:0003677~DNA binding,GO:0003700~transcription factor activity, sequence-specific DNA binding,GO:0003707~steroid hormone receptor activity,GO:0003713~transcription coactivator activity,GO:0004879~RNA polymerase II transcription factor activity, ligand-activated sequence-specific DNA binding,GO:0005515~protein binding,GO:0008270~zinc ion binding,GO:0015485~cholesterol binding,GO:0032810~sterol response element binding,GO:0043565~sequence-specific DNA binding,GO:0044212~transcription regulatory region DNA binding,
NR2E3	nuclear receptor subfamily 2 group E member 3(NR2E3)	Homo sapiens	GO:0003700~transcription factor activity, sequence-specific DNA binding,GO:0003707~steroid hormone receptor activity,GO:0004879~RNA polymerase II transcription factor activity, ligand-activated sequence-specific DNA binding,GO:0005515~protein binding,GO:0008270~zinc ion binding,GO:0043565~sequence-specific DNA binding,
NR4A1	nuclear receptor subfamily 4 group A member 1(NR4A1)	Homo sapiens	GO:0001077~transcriptional activator activity, RNA polymerase II core promoter proximal region sequence-specific binding,GO:0003677~DNA binding,GO:0003707~steroid hormone receptor activity,GO:0004879~RNA polymerase II transcription factor activity, ligand-activated sequence-specific DNA binding,GO:0005515~protein binding,GO:0008270~zinc ion binding,GO:0043565~sequence-specific DNA binding,GO:0046982~protein heterodimerization activity,
NR4A2	nuclear receptor subfamily 4 group A member 2(NR4A2)	Homo sapiens	GO:0000979~RNA polymerase II core promoter sequence-specific DNA binding,GO:0001077~transcriptional activator activity, RNA polymerase II core promoter proximal region sequence-specific binding,GO:0003677~DNA binding,GO:0003707~steroid hormone receptor activity,GO:0004879~RNA polymerase II transcription factor activity, ligand-activated sequence-specific DNA binding,GO:0005515~protein binding,GO:0008013~beta-catenin binding,GO:0008270~zinc ion binding,GO:0035259~glucocorticoid receptor binding,GO:0042803~protein homodimerization activity,GO:0043565~sequence-specific DNA binding,GO:0046965~retinoid X receptor binding,GO:0046982~protein heterodimerization activity,

NR4A3	nuclear receptor subfamily 4 group A member 3(NR4A3)	Homo sapiens	GO:0000978~RNA polymerase II core promoter proximal region sequence-specific DNA binding,GO:0001046~core promoter sequence-specific DNA binding,GO:0001077~transcriptional activator activity, RNA polymerase II core promoter proximal region sequence-specific binding,GO:0001223~transcription coactivator binding,GO:0003677~DNA binding,GO:0003707~steroid hormone receptor activity,GO:0004879~RNA polymerase II transcription factor activity, ligand-activated sequence-specific DNA binding,GO:0004887~thyroid hormone receptor activity,GO:0005515~protein binding,GO:0008270~zinc ion binding,GO:0019901~protein kinase binding,GO:0035035~histone acetyltransferase binding,GO:0035259~glucocorticoid receptor binding,GO:0042803~protein homodimerization activity,GO:0043565~sequence-specific DNA binding,
NOL4L	nucleolar protein 4 like(NOL4L)	Homo sapiens	GO:0003723~RNA binding,GO:0005515~protein binding,
NOD2	nucleotide binding oligomerization domain containing 2(NOD2)	Homo sapiens	GO:0003779~actin binding,GO:0005515~protein binding,GO:0005524~ATP binding,GO:0019899~enzyme binding,GO:0019901~protein kinase binding,GO:0030544~Hsp70 protein binding,GO:0032500~muramyl dipeptide binding,GO:0042834~peptidoglycan binding,GO:0050700~CARD domain binding,GO:0051879~Hsp90 protein binding,
NUDT18	nudix hydrolase 18(NUDT18)	Homo sapiens	GO:0000287~magnesium ion binding,GO:0005515~protein binding,GO:0016787~hydrolase activity,GO:0044715~8-oxo-dGDP phosphatase activity,GO:0044716~8-oxo-GDP phosphatase activity,GO:0044717~8-hydroxy-dADP phosphatase activity,
OBSCN	obscurin, cytoskeletal calmodulin and titin-interacting RhoGEF(OBSCN)	Homo sapiens	GO:0004672~protein kinase activity,GO:0004674~protein serine/threonine kinase activity,GO:0005085~guanyl-nucleotide exchange factor activity,GO:0005089~Rho guanyl-nucleotide exchange factor activity,GO:0005515~protein binding,GO:0005516~calmodulin binding,GO:0005524~ATP binding,GO:0008307~structural constituent of muscle,GO:0030506~ankyrin binding,GO:0031432~titin binding,GO:0046872~metal ion binding,
OSR2	odd-skipped related transciption factor 2(OSR2)	Homo sapiens	GO:0000977~RNA polymerase II regulatory region sequence-specific DNA binding,GO:0001228~transcriptional activator activity, RNA polymerase II transcription regulatory region sequence-specific binding,GO:0003676~nucleic acid binding,GO:0005515~protein binding,GO:0043565~sequence-specific DNA binding,GO:0046872~metal ion binding,
OVGP1	oviductal glycoprotein 1(OVGP1)	Homo sapiens	GO:0004568~chitinase activity,GO:0008061~chitin binding,
OVCH1	ovochymase 1(OVCH1)	Homo sapiens	GO:0004252~serine-type endopeptidase activity,GO:0046872~metal ion binding,
OGDHL	oxoglutarate dehydrogenase-like(OGDHL)	Homo sapiens	GO:0004591~oxoglutarate dehydrogenase (succinyl-transferring) activity,GO:0005515~protein binding,GO:0030976~thiamine pyrophosphate binding,GO:0046872~metal ion binding,
OXTR	oxytocin receptor(OXTR)	Homo sapiens	GO:0004990~oxytocin receptor activity,GO:0005000~vasopressin receptor activity,GO:0017046~peptide hormone binding,GO:0042277~peptide binding,
PDRG1	p53 and DNA damage regulated 1(PDRG1)	Homo sapiens	GO:0051082~unfolded protein binding,
PALM3	paralemmin 3(PALM3)	Homo sapiens	GO:0005515~protein binding,GO:0005524~ATP binding,
PALM	paralemmin(PALM)	Homo sapiens	GO:0005515~protein binding,GO:0031750~D3 dopamine receptor binding,
PNMA2	paraneoplastic Ma antigen 2(PNMA2)	Homo sapiens	GO:0005515~protein binding,
PTHLH	parathyroid hormone like hormone(PTHLH)	Homo sapiens	GO:0005179~hormone activity,GO:0051428~peptide hormone receptor binding,
PNPLA3	patatin like phospholipase domain containing 3(PNPLA3)	Homo sapiens	GO:0004465~lipoprotein lipase activity,GO:0004623~phospholipase A2 activity,GO:0004806~triglyceride lipase activity,GO:0016411~acylglycerol O-acyltransferase activity,GO:0016787~hydrolase activity,GO:0051264~mono-olein transacylation activity,GO:0051265~diolein transacylation activity,
PNPLA5	patatin like phospholipase domain containing 5(PNPLA5)	Homo sapiens	GO:0004806~triglyceride lipase activity,GO:0016787~hydrolase activity,
PNPLA7	patatin like phospholipase domain containing 7(PNPLA7)	Homo sapiens	GO:0004622~lysophospholipase activity,
PGLYRP1	peptidoglycan recognition protein 1(PGLYRP1)	Homo sapiens	GO:0008270~zinc ion binding,GO:0008745~N-acetylmuramoyl-L-alanine amidase activity,GO:0016019~peptidoglycan receptor activity,GO:0042834~peptidoglycan binding,
PADI2	peptidyl arginine deiminase 2(PADI2)	Homo sapiens	GO:0004668~protein-arginine deiminase activity,GO:0005509~calcium ion binding,GO:0030331~estrogen receptor binding,
PADI3	peptidyl arginine deiminase 3(PADI3)	Homo sapiens	GO:0004668~protein-arginine deiminase activity,GO:0005509~calcium ion binding,GO:0042802~identical protein binding,
PLIN1	perilipin 1(PLIN1)	Homo sapiens	GO:0008289~lipid binding,
PLIN5	perilipin 5(PLIN5)	Homo sapiens	GO:0035473~lipase binding,GO:0042802~identical protein binding,
PEX11B	peroxisomal biogenesis factor 11 beta(PEX11B)	Homo sapiens	GO:0005515~protein binding,GO:0042803~protein homodimerization activity,
PEX5L	peroxisomal biogenesis factor 5 like(PEX5L)	Homo sapiens	GO:0000268~peroxisome targeting sequence binding,GO:0005052~peroxisome matrix targeting signal-1 binding,GO:0031267~small GTPase binding,
PXMP4	peroxisomal membrane protein 4(PXMP4)	Homo sapiens	GO:0005515~protein binding,
PALD1	phosphatase domain containing, paladin 1(PALD1)	Homo sapiens	GO:0004725~protein tyrosine phosphatase activity,GO:0005515~protein binding,
PCYT2	phosphate cytidylyltransferase 2, ethanolamine(PCYT2)	Homo sapiens	GO:0003824~catalytic activity,GO:0004306~ethanolamine-phosphate cytidylyltransferase activity,GO:0016779~nucleotidyltransferase activity,
PEMT	phosphatidylethanolamine N-methyltransferase(PEMT)	Homo sapiens	GO:0000773~phosphatidyl-N-methylethanolamine N-methyltransferase activity,GO:0004608~phosphatidylethanolamine N-methyltransferase activity,GO:0008170~N-methyltransferase activity,GO:0008429~phosphatidylethanolamine binding,GO:0080101~phosphatidyl-N-dimethylethanolamine N-methyltransferase activity,
PIGQ	phosphatidylinositol glycan anchor biosynthesis class Q(PIGQ)	Homo sapiens	GO:0017176~phosphatidylinositol N-acetylglucosaminyltransferase activity,
PIK3C2B	phosphatidylinositol-4-phosphate 3-kinase catalytic subunit type 2 beta(PIK3C2B)	Homo sapiens	GO:0001727~lipid kinase activity,GO:0005515~protein binding,GO:0005524~ATP binding,GO:0016301~kinase activity,GO:0016303~1-phosphatidylinositol-3-kinase activity,GO:0035005~1-phosphatidylinositol-4-phosphate 3-kinase activity,GO:0035091~phosphatidylinositol binding,
PDE11A	phosphodiesterase 11A(PDE11A)	Homo sapiens	GO:0004112~cyclic-nucleotide phosphodiesterase activity,GO:0004114~3',5'-cyclic-nucleotide phosphodiesterase activity,GO:0004115~3',5'-cyclic-AMP phosphodiesterase activity,GO:0004118~cGMP-stimulated cyclic-nucleotide phosphodiesterase activity,GO:0030552~cAMP binding,GO:0030553~cGMP binding,GO:0046872~metal ion binding,GO:0047555~3',5'-cyclic-GMP phosphodiesterase activity,
PDE4B	phosphodiesterase 4B(PDE4B)	Homo sapiens	GO:0004114~3',5'-cyclic-nucleotide phosphodiesterase activity,GO:0004115~3',5'-cyclic-AMP phosphodiesterase activity,GO:0030552~cAMP binding,GO:0044325~ion channel binding,GO:0046872~metal ion binding,
PDE6B	phosphodiesterase 6B(PDE6B)	Homo sapiens	GO:0004114~3',5'-cyclic-nucleotide phosphodiesterase activity,GO:0046872~metal ion binding,GO:0047555~3',5'-cyclic-GMP phosphodiesterase activity,
PDE9A	phosphodiesterase 9A(PDE9A)	Homo sapiens	GO:0004114~3',5'-cyclic-nucleotide phosphodiesterase activity,GO:0005515~protein binding,GO:0046872~metal ion binding,GO:0047555~3',5'-cyclic-GMP phosphodiesterase activity,
PIK3AP1	phosphoinositide-3-kinase adaptor protein 1(PIK3AP1)	Homo sapiens	GO:0036312~phosphatidylinositol 3-kinase regulatory subunit binding,GO:0042802~identical protein binding,
PLA2G3	phospholipase A2 group III(PLA2G3)	Homo sapiens	GO:0004623~phospholipase A2 activity,GO:0046872~metal ion binding,GO:0047498~calcium-dependent phospholipase A2 activity,

PLA2G4F	phospholipase A2 group IVF(PLA2G4F)	Homo sapiens	GO:0004620~phospholipase activity,GO:0004622~lysophospholipase activity,GO:0004623~phospholipase A2 activity,GO:0008970~phosphatidylcholine 1-acylhydrolase activity,GO:0046872~metal ion binding,GO:0047498~calcium-dependent phospholipase A2 activity,
PLCB2	phospholipase C beta 2(PLCB2)	Homo sapiens	GO:0004435~phosphatidylinositol phospholipase C activity,GO:0004629~phospholipase C activity,GO:0004871~signal transducer activity,GO:0005509~calcium ion binding,
PLCB3	phospholipase C beta 3(PLCB3)	Homo sapiens	GO:0004435~phosphatidylinositol phospholipase C activity,GO:0004629~phospholipase C activity,GO:0004871~signal transducer activity,GO:0005509~calcium ion binding,GO:0005516~calmodulin binding,GO:0098641~cadherin binding involved in cell-cell adhesion,
PLCD1	phospholipase C delta 1(PLCD1)	Homo sapiens	GO:0001786~phosphatidylserine binding,GO:0004435~phosphatidylinositol phospholipase C activity,GO:0004871~signal transducer activity,GO:0005509~calcium ion binding,GO:0032794~GTPase activating protein binding,GO:0070300~phosphatidic acid binding,GO:1901981~phosphatidylinositol phosphate binding,
PLCD4	phospholipase C delta 4(PLCD4)	Homo sapiens	GO:0004435~phosphatidylinositol phospholipase C activity,GO:0004629~phospholipase C activity,GO:0004871~signal transducer activity,GO:0005509~calcium ion binding,
PLCL1	phospholipase C like 1(PLCL1)	Homo sapiens	GO:0004435~phosphatidylinositol phospholipase C activity,GO:0004629~phospholipase C activity,GO:0004871~signal transducer activity,GO:0050811~GABA receptor binding,GO:0070679~inositol 1,4,5 trisphosphate binding,
PLD2	phospholipase D2(PLD2)	Homo sapiens	GO:0003824~catalytic activity,GO:0004630~phospholipase D activity,GO:0005515~protein binding,GO:0035091~phosphatidylinositol binding,GO:0070290~N-acylphosphatidylethanolamine-specific phospholipase D activity,
LHPP	phospholysine phosphohistidine inorganic pyrophosphate phosphatase(LHPP)	Homo sapiens	GO:0000287~magnesium ion binding,GO:0004427~inorganic diphosphatase activity,GO:0008969~phosphohistidine phosphatase activity,GO:0042803~protein homodimerization activity,
PHKG1	phosphorylase kinase catalytic subunit gamma 1(PHKG1)	Homo sapiens	GO:0004672~protein kinase activity,GO:0004674~protein serine/threonine kinase activity,GO:0004689~phosphorylase kinase activity,GO:0005516~calmodulin binding,GO:0005524~ATP binding,GO:0019899~enzyme binding,GO:0050321~tau-protein kinase activity,
PTER	phosphotriesterase related(PTER)	Homo sapiens	GO:0008270~zinc ion binding,GO:0016788~hydrolase activity, acting on ester bonds,
PKP1	plakophilin 1(PKP1)	Homo sapiens	GO:0004871~signal transducer activity,GO:0005515~protein binding,GO:0005521~lamin binding,GO:0019215~intermediate filament binding,GO:0030280~structural constituent of epidermis,
PKP3	plakophilin 3(PKP3)	Homo sapiens	GO:0045294~alpha-catenin binding,GO:0050839~cell adhesion molecule binding,GO:0098641~cadherin binding involved in cell-cell adhesion,
PLGRKT	plasminogen receptor with a C-terminal lysine(PLGRKT)	Homo sapiens	GO:0005515~protein binding,
PDGFB	platelet derived growth factor subunit B(PDGFB)	Homo sapiens	GO:0005088~Ras guanyl-nucleotide exchange factor activity,GO:0005161~platelet-derived growth factor receptor binding,GO:0005515~protein binding,GO:0005518~collagen binding,GO:0008083~growth factor activity,GO:0016176~superoxide-generating NADPH oxidase activator activity,GO:0042056~chemoattractant activity,GO:0042802~identical protein binding,GO:0042803~protein homodimerization activity,GO:0046934~phosphatidylinositol-4,5-bisphosphate 3-kinase activity,GO:0046982~protein heterodimerization activity,GO:0048407~platelet-derived growth factor binding,
PLEKHM1	pleckstrin homology and RUN domain containing M1(PLEKHM1)	Homo sapiens	GO:0046872~metal ion binding,
PLEKHG4	pleckstrin homology and RhoGEF domain containing G4(PLEKHG4)	Homo sapiens	GO:0005089~Rho guanyl-nucleotide exchange factor activity,GO:0005515~protein binding,
PLEKHN1	pleckstrin homology domain containing N1(PLEKHN1)	Homo sapiens	GO:0005515~protein binding,
PHLDA3	pleckstrin homology like domain family A member 3(PHLDA3)	Homo sapiens	GO:0005546~phosphatidylinositol-4,5-bisphosphate binding,GO:0005547~phosphatidylinositol-3,4,5-trisphosphate binding,GO:0010314~phosphatidylinositol-5-phosphate binding,GO:0032266~phosphatidylinositol-3-phosphate binding,GO:0043325~phosphatidylinositol-3,4-bisphosphate binding,GO:0080025~phosphatidylinositol-3,5-bisphosphate binding,
PLXNA3	plexin A3(PLXNA3)	Homo sapiens	GO:0004888~transmembrane signaling receptor activity,GO:0005515~protein binding,GO:0017154~semaphorin receptor activity,
PLXNB3	plexin B3(PLXNB3)	Homo sapiens	GO:0005515~protein binding,GO:0017154~semaphorin receptor activity,GO:0019904~protein domain specific binding,GO:0051022~Rho GDP-dissociation inhibitor binding,GO:0098632~protein binding involved in cell-cell adhesion,
PLK3	polo like kinase 3(PLK3)	Homo sapiens	GO:0002039~p53 binding,GO:0004674~protein serine/threonine kinase activity,GO:0005515~protein binding,GO:0005524~ATP binding,
PABPC5	poly(A) binding protein cytoplasmic 5(PABPC5)	Homo sapiens	GO:0000166~nucleotide binding,GO:0003676~nucleic acid binding,GO:0003723~RNA binding,
PAOX	polyamine oxidase(PAOX)	Homo sapiens	GO:0005102~receptor binding,GO:0016491~oxidoreductase activity,GO:0046592~polyamine oxidase activity,GO:0052899~N(1),N(12)-diacetylspermine:oxygen oxidoreductase (3-acetamidopropanal-forming) activity,GO:0052901~spermine:oxygen oxidoreductase (spermidine-forming) activity,GO:0052902~spermidine:oxygen oxidoreductase (3-aminopropanal-forming) activity,GO:0052903~N1-acetylspermine:oxygen oxidoreductase (3-acetamidopropanal-forming) activity,GO:0052904~N1-acetylspermidine:oxygen oxidoreductase (3-acetamidopropanal-forming) activity,
PKD1L3	polycystin 1 like 3, transient receptor potential channel interacting(PKD1L3)	Homo sapiens	GO:0005261~cation channel activity,GO:0005262~calcium channel activity,GO:0005515~protein binding,GO:0008324~cation transmembrane transporter activity,GO:0030246~carbohydrate binding,GO:0033040~sour taste receptor activity,
PKD1	polycystin 1, transient receptor potential channel interacting(PKD1)	Homo sapiens	GO:0005262~calcium channel activity,GO:0005515~protein binding,GO:0019901~protein kinase binding,GO:0019904~protein domain specific binding,GO:0030246~carbohydrate binding,GO:0044325~ion channel binding,
GALNT12	polypeptide N-acetylgalactosaminyltransferase 12(GALNT12)	Homo sapiens	GO:0004653~polypeptide N-acetylgalactosaminyltransferase activity,GO:0016740~transferase activity,GO:0030246~carbohydrate binding,GO:0046872~metal ion binding,
PGAP3	post-GPI attachment to proteins 3(PGAP3)	Homo sapiens	GO:0016788~hydrolase activity, acting on ester bonds,
KCNIP3	potassium voltage-gated channel interacting protein 3(KCNIP3)	Homo sapiens	GO:0000978~RNA polymerase II core promoter proximal region sequence-specific DNA binding,GO:0001078~transcriptional repressor activity, RNA polymerase II core promoter proximal region sequence-specific binding,GO:0003677~DNA binding,GO:0003714~transcription corepressor activity,GO:0005244~voltage-gated ion channel activity,GO:0005267~potassium channel activity,GO:0005509~calcium ion binding,GO:0005515~protein binding,GO:0015459~potassium channel regulator activity,GO:0043565~sequence-specific DNA binding,GO:0044325~ion channel binding,
KCNS1	potassium voltage-gated channel modifier subfamily S member 1(KCNS1)	Homo sapiens	GO:0005249~voltage-gated potassium channel activity,GO:0005251~delayed rectifier potassium channel activity,GO:0005515~protein binding,GO:0015459~potassium channel regulator activity,

KCNB1	potassium voltage-gated channel subfamily B member 1(KCNB1)	Homo sapiens	GO:0005249~voltage-gated potassium channel activity,GO:0005251~delayed rectifier potassium channel activity,GO:0005515~protein binding,GO:0032182~ubiquitin-like protein binding,GO:0044325~ion channel binding,GO:0046982~protein heterodimerization activity,
KCNC3	potassium voltage-gated channel subfamily C member 3(KCNC3)	Homo sapiens	GO:0005249~voltage-gated potassium channel activity,GO:0005251~delayed rectifier potassium channel activity,
KCND3	potassium voltage-gated channel subfamily D member 3(KCND3)	Homo sapiens	GO:0005249~voltage-gated potassium channel activity,GO:0005250~A-type (transient outward) potassium channel activity,GO:0005251~delayed rectifier potassium channel activity,GO:0005515~protein binding,GO:0044325~ion channel binding,GO:0046872~metal ion binding,GO:0086008~voltage-gated potassium channel activity involved in cardiac muscle cell action potential repolarization,GO:1902282~voltage-gated potassium channel activity involved in ventricular cardiac muscle cell action potential repolarization,
KCNJ1	potassium voltage-gated channel subfamily J member 1(KCNJ1)	Homo sapiens	GO:0005242~inward rectifier potassium channel activity,GO:0005524~ATP binding,GO:0005546~phosphatidylinositol-4,5-bisphosphate binding,GO:0015272~ATP-activated inward rectifier potassium channel activity,
KCNJ4	potassium voltage-gated channel subfamily J member 4(KCNJ4)	Homo sapiens	GO:0005242~inward rectifier potassium channel activity,GO:0005515~protein binding,GO:0015467~G-protein activated inward rectifier potassium channel activity,GO:0030165~PDZ domain binding,
KCNJ5	potassium voltage-gated channel subfamily J member 5(KCNJ5)	Homo sapiens	GO:0005242~inward rectifier potassium channel activity,GO:0005515~protein binding,GO:0015467~G-protein activated inward rectifier potassium channel activity,GO:0086089~voltage-gated potassium channel activity involved in atrial cardiac muscle cell action potential repolarization,GO:1902282~voltage-gated potassium channel activity involved in ventricular cardiac muscle cell action potential repolarization,
KCNQ4	potassium voltage-gated channel subfamily Q member 4(KCNQ4)	Homo sapiens	GO:0005249~voltage-gated potassium channel activity,GO:0005251~delayed rectifier potassium channel activity,GO:0005267~potassium channel activity,GO:0005515~protein binding,
PRPF3	pre-mRNA processing factor 3(PRPF3)	Homo sapiens	GO:0005515~protein binding,GO:0042802~identical protein binding,GO:0044822~poly(A) RNA binding,
PRPF39	pre-mRNA processing factor 39(PRPF39)	Homo sapiens	GO:0030627~pre-mRNA 5'-splice site binding,
PNCK	pregnancy up-regulated nonubiquitous CaM kinase(PNCK)	Homo sapiens	GO:0004672~protein kinase activity,GO:0004674~protein serine/threonine kinase activity,GO:0004683~calmodulin-dependent protein kinase activity,GO:0005516~calmodulin binding,GO:0005524~ATP binding,
PCYOX1L	prenylcysteine oxidase 1 like(PCYOX1L)	Homo sapiens	GO:0001735~prenylcysteine oxidase activity,
PAQR8	progestin and adipoQ receptor family member 8(PAQR8)	Homo sapiens	GO:0003707~steroid hormone receptor activity,GO:0005496~steroid binding,
PRLR	prolactin receptor(PRLR)	Homo sapiens	GO:0004925~prolactin receptor activity,GO:0005515~protein binding,GO:0017046~peptide hormone binding,GO:0042803~protein homodimerization activity,GO:0042978~ornithine decarboxylase activator activity,GO:0046872~metal ion binding,
PROSER2	proline and serine rich 2(PROSER2)	Homo sapiens	GO:0005515~protein binding,
PRODH	proline dehydrogenase 1(PRODH)	Homo sapiens	GO:0004657~proline dehydrogenase activity,GO:0071949~FAD binding,
PCSK4	proprotein convertase subtilisin/kexin type 4(PCSK4)	Homo sapiens	GO:0004252~serine-type endopeptidase activity,GO:0005515~protein binding,
PROX2	prospero homeobox 2(PROX2)	Homo sapiens	GO:0001078~transcriptional repressor activity, RNA polymerase II core promoter proximal region sequence-specific binding,GO:0003677~DNA binding,
PTGDS	prostaglandin D2 synthase(PTGDS)	Homo sapiens	GO:0004667~prostaglandin-D synthase activity,GO:0005215~transporter activity,GO:0005501~retinoid binding,GO:0005504~fatty acid binding,GO:0005515~protein binding,GO:0036094~small molecule binding,
PTGS2	prostaglandin-endoperoxide synthase 2(PTGS2)	Homo sapiens	GO:0004601~peroxidase activity,GO:0004666~prostaglandin-endoperoxide synthase activity,GO:0005515~protein binding,GO:0008289~lipid binding,GO:0019899~enzyme binding,GO:0020037~heme binding,GO:0042803~protein homodimerization activity,GO:0046872~metal ion binding,GO:0050473~arachidonate 15-lipoxygenase activity,
PRSS48	protease, serine 48(PRSS48)	Homo sapiens	GO:0004252~serine-type endopeptidase activity,
PAAF1	proteasomal ATPase associated factor 1(PAAF1)	Homo sapiens	GO:0005515~protein binding,
PSMA2	proteasome subunit alpha 2(PSMA2)	Homo sapiens	GO:0004298~threonine-type endopeptidase activity,GO:0005515~protein binding,
POFUT2	protein O-fucosyltransferase 2(POFUT2)	Homo sapiens	GO:0016757~transferase activity, transferring glycosyl groups,GO:0046922~peptide-O-fucosyltransferase activity,
POGLUT1	protein O-glucosyltransferase 1(POGLUT1)	Homo sapiens	GO:0030158~protein xylosyltransferase activity,GO:0035251~UDP-glucosyltransferase activity,GO:0035252~UDP-xylosyltransferase activity,GO:0046527~glucosyltransferase activity,
PROZ	protein Z, vitamin K dependent plasma glycoprotein(PROZ)	Homo sapiens	GO:0004252~serine-type endopeptidase activity,GO:0005509~calcium ion binding,
PACSIN1	protein kinase C and casein kinase substrate in neurons 1(PACSIN1)	Homo sapiens	GO:0005515~protein binding,GO:0005543~phospholipid binding,GO:0008092~cytoskeletal protein binding,GO:0016301~kinase activity,
PACSIN3	protein kinase C and casein kinase substrate in neurons 3(PACSIN3)	Homo sapiens	GO:0005515~protein binding,GO:0008092~cytoskeletal protein binding,GO:0008289~lipid binding,GO:0016301~kinase activity,GO:0019855~calcium channel inhibitor activity,
PRKAR1B	protein kinase cAMP-dependent type I regulatory subunit beta(PRKAR1B)	Homo sapiens	GO:0004862~cAMP-dependent protein kinase inhibitor activity,GO:0005515~protein binding,GO:0008603~cAMP-dependent protein kinase regulator activity,GO:0016301~kinase activity,GO:0030552~cAMP binding,GO:0034236~protein kinase A catalytic subunit binding,
PKMYT1	protein kinase, membrane associated tyrosine/threonine 1(PKMYT1)	Homo sapiens	GO:0004672~protein kinase activity,GO:0004674~protein serine/threonine kinase activity,GO:0005515~protein binding,GO:0005524~ATP binding,GO:0016301~kinase activity,GO:0046872~metal ion binding,
PPP1R15A	protein phosphatase 1 regulatory subunit 15A(PPP1R15A)	Homo sapiens	GO:0005515~protein binding,GO:0008157~protein phosphatase 1 binding,GO:0008599~protein phosphatase type 1 regulator activity,GO:0019888~protein phosphatase regulator activity,GO:0019901~protein kinase binding,GO:0071862~protein phosphatase type 1 activator activity,GO:0072542~protein phosphatase activator activity,
PPP2R2C	protein phosphatase 2 regulatory subunit Bgamma(PPP2R2C)	Homo sapiens	GO:0004722~protein serine/threonine phosphatase activity,GO:0005515~protein binding,GO:0008601~protein phosphatase type 2A regulator activity,GO:0019888~protein phosphatase regulator activity,
PP2D1	protein phosphatase 2C like domain containing 1(PP2D1)	Homo sapiens	GO:0004722~protein serine/threonine phosphatase activity,
PPM1F	protein phosphatase, Mg2+/Mn2+ dependent 1F(PPM1F)	Homo sapiens	GO:0004722~protein serine/threonine phosphatase activity,GO:0005515~protein binding,GO:0016791~phosphatase activity,GO:0033192~calmodulin-dependent protein phosphatase activity,GO:0043169~cation binding,GO:0046872~metal ion binding,
PSKH1	protein serine kinase H1(PSKH1)	Homo sapiens	GO:0004674~protein serine/threonine kinase activity,GO:0005515~protein binding,GO:0005524~ATP binding,
PTK2B	protein tyrosine kinase 2 beta(PTK2B)	Homo sapiens	GO:0004683~calmodulin-dependent protein kinase activity,GO:0004713~protein tyrosine kinase activity,GO:0004715~non-membrane spanning protein tyrosine kinase activity,GO:0004871~signal transducer activity,GO:0004972~NMDA glutamate receptor activity,GO:0005102~receptor binding,GO:0005515~protein binding,GO:0005524~ATP binding,GO:0032403~protein complex binding,GO:0043423~3-phosphoinositide-dependent protein kinase binding,
PTK6	protein tyrosine kinase 6(PTK6)	Homo sapiens	GO:0004713~protein tyrosine kinase activity,GO:0004715~non-membrane spanning protein tyrosine kinase activity,GO:0005102~receptor binding,GO:0005515~protein binding,GO:0005524~ATP binding,GO:0042802~identical protein binding,

PTPN18	protein tyrosine phosphatase, non-receptor type 18(PTPN18)	Homo sapiens	GO:0004725~protein tyrosine phosphatase activity,GO:0004726~non-membrane spanning protein tyrosine phosphatase activity,GO:0005515~protein binding,
PGGHG	protein-glucosylgalactosylhydroxylysine glucosidase(PGGHG)	Homo sapiens	GO:0003824~catalytic activity,GO:0016787~hydrolase activity,GO:0016798~hydrolase activity, acting on glycosyl bonds,
PCDH10	protocadherin 10(PCDH10)	Homo sapiens	GO:0005509~calcium ion binding,
PCDH17	protocadherin 17(PCDH17)	Homo sapiens	GO:0005509~calcium ion binding,GO:0005515~protein binding,
PCDH19	protocadherin 19(PCDH19)	Homo sapiens	GO:0005509~calcium ion binding,
PCDHB15	protocadherin beta 15(PCDHB15)	Homo sapiens	GO:0005509~calcium ion binding,
P2RX7	purinergic receptor P2X 7(P2RX7)	Homo sapiens	GO:0001530~lipopolysaccharide binding,GO:0001614~purinergic nucleotide receptor activity,GO:0004931~extracellular ATP-gated cation channel activity,GO:0005102~receptor binding,GO:0005216~ion channel activity,GO:0005515~protein binding,GO:0005524~ATP binding,GO:0042803~protein homodimerization activity,
PYROXD2	pyridine nucleotide-disulphide oxidoreductase domain 2(PYROXD2)	Homo sapiens	GO:0005515~protein binding,GO:0016491~oxidoreductase activity,
P2RY6	pyrimidinergic receptor P2Y6(P2RY6)	Homo sapiens	GO:0004930~G-protein coupled receptor activity,GO:0005515~protein binding,GO:0045028~G-protein coupled purinergic nucleotide receptor activity,GO:0045029~UDP-activated nucleotide receptor activity,
QRFP	pyroglutamylated RFamide peptide(QRFP)	Homo sapiens	GO:0005184~neuropeptide hormone activity,GO:0031854~orexigenic neuropeptide QRFP receptor binding,
QTRT1	queuine tRNA-ribosyltransferase catalytic subunit 1(QTRT1)	Homo sapiens	GO:0008479~queuine tRNA-ribosyltransferase activity,GO:0042803~protein homodimerization activity,GO:0046872~metal ion binding,GO:0046982~protein heterodimerization activity,
RPH3AL	rabphilin 3A-like (without C2 domains)(RPH3AL)	Homo sapiens	GO:0005509~calcium ion binding,GO:0005515~protein binding,GO:0005544~calcium-dependent phospholipid binding,GO:0008092~cytoskeletal protein binding,GO:0017137~Rab GTPase binding,GO:0019905~syntaxin binding,GO:0030274~LIM domain binding,GO:0030276~clathrin binding,GO:0046872~metal ion binding,
RGL3	ral guanine nucleotide dissociation stimulator like 3(RGL3)	Homo sapiens	GO:0008321~Ral guanyl-nucleotide exchange factor activity,GO:0017016~Ras GTPase binding,
RASD1	ras related dexamethasone induced 1(RASD1)	Homo sapiens	GO:0003924~GTPase activity,GO:0005515~protein binding,GO:0005525~GTP binding,
RAC2	ras-related C3 botulinum toxin substrate 2 (rho family, small GTP binding protein Rac2)(RAC2)	Homo sapiens	GO:0003924~GTPase activity,GO:0005525~GTP binding,GO:0019887~protein kinase regulator activity,
RAC3	ras-related C3 botulinum toxin substrate 3 (rho family, small GTP binding protein Rac3)(RAC3)	Homo sapiens	GO:0003924~GTPase activity,GO:0005515~protein binding,GO:0005525~GTP binding,GO:0048306~calcium-dependent protein binding,
RLF	rearranged L-myc fusion(RLF)	Homo sapiens	GO:0003677~DNA binding,GO:0005515~protein binding,GO:0008270~zinc ion binding,
RIMS3	regulating synaptic membrane exocytosis 3(RIMS3)	Homo sapiens	GO:0044325~ion channel binding,
RGS11	regulator of G-protein signaling 11(RGS11)	Homo sapiens	GO:0004871~signal transducer activity,GO:0005096~GTPase activator activity,GO:0031681~G-protein beta-subunit binding,
RFX5	regulatory factor X5(RFX5)	Homo sapiens	GO:0000978~RNA polymerase II core promoter proximal region sequence-specific DNA binding,GO:0003677~DNA binding,GO:0003700~transcription factor activity, sequence-specific DNA binding,GO:0005515~protein binding,
RPRM	reprimo, TP53 dependent G2 arrest mediator candidate(RPRM)	Homo sapiens	GO:0005515~protein binding,
RTN4RL1	reticulon 4 receptor like 1(RTN4RL1)	Homo sapiens	GO:0004860~protein kinase inhibitor activity,GO:0004872~receptor activity,GO:0005515~protein binding,
RD3	retinal degeneration 3(RD3)	Homo sapiens	GO:0005515~protein binding,
RRH	retinal pigment epithelium-derived rhodopsin homolog(RRH)	Homo sapiens	GO:0004930~G-protein coupled receptor activity,GO:0008020~G-protein coupled photoreceptor activity,
RDH16	retinol dehydrogenase 16 (all-trans)(RDH16)	Homo sapiens	GO:0004745~retinol dehydrogenase activity,GO:0009055~electron carrier activity,
RS1	retinoschisin 1(RS1)	Homo sapiens	GO:0001786~phosphatidylserine binding,GO:0005546~phosphatidylinositol-4,5-bisphosphate binding,GO:0005547~phosphatidylinositol-3,4,5-trisphosphate binding,GO:0010314~phosphatidylinositol-5-phosphate binding,GO:0032266~phosphatidylinositol-3-phosphate binding,GO:0043325~phosphatidylinositol-3,4-bisphosphate binding,GO:0070273~phosphatidylinositol-4-phosphate binding,GO:0080025~phosphatidylinositol-3,5-bisphosphate binding,
RHPN1	rhophilin Rho GTPase binding protein 1(RHPN1)	Homo sapiens	GO:0005515~protein binding,
RPL3L	ribosomal protein L3 like(RPL3L)	Homo sapiens	GO:0003723~RNA binding,GO:0003735~structural constituent of ribosome,
RPS6KA2	ribosomal protein S6 kinase A2(RPS6KA2)	Homo sapiens	GO:0000287~magnesium ion binding,GO:0004672~protein kinase activity,GO:0004674~protein serine/threonine kinase activity,GO:0004711~ribosomal protein S6 kinase activity,GO:0004712~protein serine/threonine/tyrosine kinase activity,GO:0005515~protein binding,GO:0005524~ATP binding,GO:0016301~kinase activity,
RPS9	ribosomal protein S9(RPS9)	Homo sapiens	GO:0003735~structural constituent of ribosome,GO:0005515~protein binding,GO:0019843~rRNA binding,GO:0044822~poly(A) RNA binding,GO:0045182~translation regulator activity,
RNF152	ring finger protein 152(RNF152)	Homo sapiens	GO:0004842~ubiquitin-protein transferase activity,GO:0008270~zinc ion binding,GO:0016874~ligase activity,GO:0031267~small GTPase binding,GO:0061630~ubiquitin protein ligase activity,
RNF157	ring finger protein 157(RNF157)	Homo sapiens	GO:0008270~zinc ion binding,
RNF170	ring finger protein 170(RNF170)	Homo sapiens	GO:0005515~protein binding,GO:0008270~zinc ion binding,GO:0016874~ligase activity,
RNF183	ring finger protein 183(RNF183)	Homo sapiens	GO:0005515~protein binding,GO:0008270~zinc ion binding,
RNF19B	ring finger protein 19B(RNF19B)	Homo sapiens	GO:0004842~ubiquitin-protein transferase activity,GO:0005515~protein binding,GO:0008270~zinc ion binding,GO:0016874~ligase activity,GO:0031624~ubiquitin conjugating enzyme binding,GO:0061630~ubiquitin protein ligase activity,
RNF207	ring finger protein 207(RNF207)	Homo sapiens	GO:0005515~protein binding,GO:0008270~zinc ion binding,GO:0030544~Hsp70 protein binding,GO:0044325~ion channel binding,GO:0051087~chaperone binding,
RNF213	ring finger protein 213(RNF213)	Homo sapiens	GO:0004842~ubiquitin-protein transferase activity,GO:0008270~zinc ion binding,GO:0016874~ligase activity,GO:0016887~ATPase activity,
RNF224	ring finger protein 224(RNF224)	Homo sapiens	GO:0008270~zinc ion binding,
RNF225	ring finger protein 225(RNF225)	Homo sapiens	GO:0008270~zinc ion binding,
RNFT2	ring finger protein, transmembrane 2(RNFT2)	Homo sapiens	GO:0008270~zinc ion binding,
RUNX2	runt related transcription factor 2(RUNX2)	Homo sapiens	GO:0000978~RNA polymerase II core promoter proximal region sequence-specific DNA binding,GO:0000979~RNA polymerase II core promoter sequence-specific DNA binding,GO:0000981~RNA polymerase II transcription factor activity, sequence-specific DNA binding,GO:0001077~transcriptional activator activity, RNA polymerase II core promoter proximal region sequence-specific binding,GO:0003677~DNA binding,GO:0003682~chromatin binding,GO:0003700~transcription factor activity, sequence-specific DNA binding,GO:0005515~protein binding,GO:0005524~ATP binding,GO:0019904~protein domain specific binding,GO:0043425~bHLH transcription factor binding,GO:0070491~repressing transcription factor binding,
SARDH	sarcosine dehydrogenase(SARDH)	Homo sapiens	GO:0008480~sarcosine dehydrogenase activity,GO:0016491~oxidoreductase activity,

SCARA3	scavenger receptor class A member 3(SCARA3)	Homo sapiens	GO:0005044~scavenger receptor activity,GO:0005198~structural molecule activity,GO:0005515~protein binding,
SLFN5	schlafen family member 5(SLFN5)	Homo sapiens	GO:0005524~ATP binding,
SCHIP1	schwannomin interacting protein 1(SCHIP1)	Homo sapiens	GO:0005515~protein binding,GO:0042802~identical protein binding,
SCAMP5	secretory carrier membrane protein 5(SCAMP5)	Homo sapiens	GO:0005515~protein binding,
SELPLG	selectin P ligand(SELPLG)	Homo sapiens	GO:0001618~virus receptor activity,GO:0005102~receptor binding,GO:0005515~protein binding,
SELENBP1	selenium binding protein 1(SELENBP1)	Homo sapiens	GO:0005515~protein binding,GO:0008430~selenium binding,
SEMA3C	semaphorin 3C(SEMA3C)	Homo sapiens	GO:0030215~semaphorin receptor binding,GO:0038191~neuropilin binding,GO:0045499~chemorepellent activity,
SEMA4A	semaphorin 4A(SEMA4A)	Homo sapiens	GO:0005515~protein binding,GO:0030215~semaphorin receptor binding,GO:0038191~neuropilin binding,GO:0045499~chemorepellent activity,
SEMA6B	semaphorin 6B(SEMA6B)	Homo sapiens	GO:0030215~semaphorin receptor binding,GO:0045499~chemorepellent activity,
SDSL	serine dehydratase like(SDSL)	Homo sapiens	GO:0003941~L-serine ammonia-lyase activity,GO:0004794~L-threonine ammonia-lyase activity,GO:0030170~pyridoxal phosphate binding,GO:0042802~identical protein binding,
SPTLC3	serine palmitoyltransferase long chain base subunit 3(SPTLC3)	Homo sapiens	GO:0003824~catalytic activity,GO:0004758~serine C-palmitoyltransferase activity,GO:0008483~transaminase activity,GO:0016740~transferase activity,GO:0030170~pyridoxal phosphate binding,
SPINK1	serine peptidase inhibitor, Kazal type 1(SPINK1)	Homo sapiens	GO:0004866~endopeptidase inhibitor activity,GO:0004867~serine-type endopeptidase inhibitor activity,GO:0005515~protein binding,
SERPINB8	serpin family B member 8(SERPINB8)	Homo sapiens	GO:0004867~serine-type endopeptidase inhibitor activity,GO:0005515~protein binding,
SERPINE1	serpin family E member 1(SERPINE1)	Homo sapiens	GO:0002020~protease binding,GO:0004867~serine-type endopeptidase inhibitor activity,GO:0005102~receptor binding,GO:0005515~protein binding,
SGK1	serum/glucocorticoid regulated kinase 1(SGK1)	Homo sapiens	GO:0004674~protein serine/threonine kinase activity,GO:0004712~protein serine/threonine/tyrosine kinase activity,GO:0005246~calcium channel regulator activity,GO:0005515~protein binding,GO:0005524~ATP binding,GO:0015459~potassium channel regulator activity,GO:0017080~sodium channel regulator activity,GO:0017081~chloride channel regulator activity,GO:0035091~phosphatidylinositol binding,
SFXN3	sideroflexin 3(SFXN3)	Homo sapiens	GO:0015075~ion transmembrane transporter activity,
SPPL2B	signal peptide peptidase like 2B(SPPL2B)	Homo sapiens	GO:0005515~protein binding,GO:0042500~aspartic endopeptidase activity, intramembrane cleaving,GO:0042803~protein homodimerization activity,
STAT6	signal transducer and activator of transcription 6(STAT6)	Homo sapiens	GO:0000979~RNA polymerase II core promoter sequence-specific DNA binding,GO:0003677~DNA binding,GO:0003700~transcription factor activity, sequence-specific DNA binding,GO:0004871~signal transducer activity,GO:0005515~protein binding,GO:0019903~protein phosphatase binding,GO:0042802~identical protein binding,
STAP2	signal transducing adaptor family member 2(STAP2)	Homo sapiens	GO:0005515~protein binding,
SIGIRR	single Ig and TIR domain containing(SIGIRR)	Homo sapiens	GO:0005515~protein binding,
SIRT2	sirtuin 2(SIRT2)	Homo sapiens	GO:0003682~chromatin binding,GO:0003950~NAD+ ADP-ribosyltransferase activity,GO:0004407~histone deacetylase activity,GO:0005515~protein binding,GO:0008134~transcription factor binding,GO:0008270~zinc ion binding,GO:0016811~hydrolase activity, acting on carbon-nitrogen (but not peptide) bonds, in linear amides,GO:0017136~NAD-dependent histone deacetylase activity,GO:0033558~protein deacetylase activity,GO:0034979~NAD-dependent protein deacetylase activity,GO:0035035~histone acetyltransferase binding,GO:0042826~histone deacetylase binding,GO:0042903~tubulin deacetylase activity,GO:0043130~ubiquitin binding,GO:0046970~NAD-dependent histone deacetylase activity (H4-K16 specific),GO:0048487~beta-tubulin binding,GO:0070403~NAD+ binding,
SSH3	slingshot protein phosphatase 3(SSH3)	Homo sapiens	GO:0003677~DNA binding,GO:0003779~actin binding,GO:0004725~protein tyrosine phosphatase activity,GO:0008138~protein tyrosine/serine/threonine phosphatase activity,
SLIT1	slit guidance ligand 1(SLIT1)	Homo sapiens	GO:0005509~calcium ion binding,GO:0008201~heparin binding,GO:0048495~Roundabout binding,
SCNN1A	sodium channel epithelial 1 alpha subunit(SCNN1A)	Homo sapiens	GO:0005272~sodium channel activity,GO:0005515~protein binding,GO:0015280~ligand-gated sodium channel activity,GO:0050699~WW domain binding,
SCNN1D	sodium channel epithelial 1 delta subunit(SCNN1D)	Homo sapiens	GO:0005272~sodium channel activity,GO:0005515~protein binding,GO:0015280~ligand-gated sodium channel activity,
SLC1A1	solute carrier family 1 member 1(SLC1A1)	Homo sapiens	GO:0005313~L-glutamate transmembrane transporter activity,GO:0005314~high-affinity glutamate transmembrane transporter activity,GO:0005515~protein binding,GO:0015171~amino acid transmembrane transporter activity,GO:0015293~symporter activity,GO:0015501~glutamate:sodium symporter activity,GO:0016595~glutamate binding,GO:0033229~cysteine transmembrane transporter activity,
SLC10A1	solute carrier family 10 member 1(SLC10A1)	Homo sapiens	GO:0001618~virus receptor activity,GO:0008508~bile acid:sodium symporter activity,
SLC13A3	solute carrier family 13 member 3(SLC13A3)	Homo sapiens	GO:0005215~transporter activity,GO:0015137~citrate transmembrane transporter activity,GO:0015141~succinate transmembrane transporter activity,GO:0015362~high-affinity sodium:dicarboxylate symporter activity,GO:0017153~sodium:dicarboxylate symporter activity,
SLC14A1	solute carrier family 14 member 1 (Kidd blood group)(SLC14A1)	Homo sapiens	GO:0005372~water transmembrane transporter activity,GO:0015204~urea transmembrane transporter activity,GO:0015265~urea channel activity,
SLC14A2	solute carrier family 14 member 2(SLC14A2)	Homo sapiens	GO:0015204~urea transmembrane transporter activity,GO:0015265~urea channel activity,GO:0050839~cell adhesion molecule binding,
SLC16A13	solute carrier family 16 member 13(SLC16A13)	Homo sapiens	GO:0015129~lactate transmembrane transporter activity,GO:0015293~symporter activity,
SLC16A2	solute carrier family 16 member 2(SLC16A2)	Homo sapiens	GO:0005215~transporter activity,GO:0008028~monocarboxylic acid transmembrane transporter activity,GO:0015293~symporter activity,GO:0015349~thyroid hormone transmembrane transporter activity,
SLC16A8	solute carrier family 16 member 8(SLC16A8)	Homo sapiens	GO:0008028~monocarboxylic acid transmembrane transporter activity,GO:0015129~lactate transmembrane transporter activity,GO:0015293~symporter activity,
SLC18B1	solute carrier family 18 member B1(SLC18B1)	Homo sapiens	GO:0022857~transmembrane transporter activity,
SLC2A10	solute carrier family 2 member 10(SLC2A10)	Homo sapiens	GO:0005351~sugar:proton symporter activity,GO:0005355~glucose transmembrane transporter activity,GO:0022857~transmembrane transporter activity,GO:0055056~D-glucose transmembrane transporter activity,
SLC2A6	solute carrier family 2 member 6(SLC2A6)	Homo sapiens	GO:0005351~sugar:proton symporter activity,GO:0005355~glucose transmembrane transporter activity,GO:0022857~transmembrane transporter activity,GO:0055056~D-glucose transmembrane transporter activity,
SLC22A13	solute carrier family 22 member 13(SLC22A13)	Homo sapiens	GO:0015101~organic cation transmembrane transporter activity,GO:0022891~substrate-specific transmembrane transporter activity,GO:0090416~nicotinate transporter activity,
SLC22A18	solute carrier family 22 member 18(SLC22A18)	Homo sapiens	GO:0005215~transporter activity,GO:0015238~drug transmembrane transporter activity,GO:0015293~symporter activity,GO:0015307~drug:proton antiporter activity,GO:0031625~ubiquitin protein ligase binding,

SLC23A1	solute carrier family 23 member 1(SLC23A1)	Homo sapiens	GO:0005515~protein binding,GO:0008520~L-ascorbate:sodium symporter activity,GO:0015081~sodium ion transmembrane transporter activity,GO:0015205~nucleobase transmembrane transporter activity,GO:0015229~L-ascorbic acid transporter activity,GO:0033300~dehydroascorbic acid transporter activity,GO:0070890~sodium-dependent L-ascorbate transmembrane transporter activity,
SLC24A1	solute carrier family 24 member 1(SLC24A1)	Homo sapiens	GO:0005262~calcium channel activity,GO:0005509~calcium ion binding,GO:0005515~protein binding,GO:0008273~calcium, potassium:sodium antiporter activity,GO:0015293~symporter activity,GO:0030955~potassium ion binding,GO:0031402~sodium ion binding,
SLC25A1	solute carrier family 25 member 1(SLC25A1)	Homo sapiens	GO:0003735~structural constituent of ribosome,GO:0015137~citrate transmembrane transporter activity,GO:0015142~tricarboxylic acid transmembrane transporter activity,
SLC25A10	solute carrier family 25 member 10(SLC25A10)	Homo sapiens	GO:0003735~structural constituent of ribosome,GO:0005310~dicarboxylic acid transmembrane transporter activity,GO:0005515~protein binding,
SLC25A18	solute carrier family 25 member 18(SLC25A18)	Homo sapiens	GO:0003735~structural constituent of ribosome,GO:0005515~protein binding,GO:0015293~symporter activity,
SLC25A20	solute carrier family 25 member 20(SLC25A20)	Homo sapiens	GO:0003735~structural constituent of ribosome,GO:0015227~acyl carnitine transmembrane transporter activity,
SLC25A35	solute carrier family 25 member 35(SLC25A35)	Homo sapiens	GO:0003735~structural constituent of ribosome,
SLC25A42	solute carrier family 25 member 42(SLC25A42)	Homo sapiens	GO:0003735~structural constituent of ribosome,GO:0005347~ATP transmembrane transporter activity,GO:0015217~ADP transmembrane transporter activity,GO:0015228~coenzyme A transmembrane transporter activity,GO:0043262~adenosine-diphosphatase activity,GO:0080122~AMP transmembrane transporter activity,
SLC26A10	solute carrier family 26 member 10(SLC26A10)	Homo sapiens	GO:0005254~chloride channel activity,GO:0008271~secondary active sulfate transmembrane transporter activity,GO:0015106~bicarbonate transmembrane transporter activity,GO:0015116~sulfate transmembrane transporter activity,GO:0015301~anion:anion antiporter activity,GO:0019531~oxalate transmembrane transporter activity,
SLC29A3	solute carrier family 29 member 3(SLC29A3)	Homo sapiens	GO:0005337~nucleoside transmembrane transporter activity,
SLC30A2	solute carrier family 30 member 2(SLC30A2)	Homo sapiens	GO:0005385~zinc ion transmembrane transporter activity,GO:0005515~protein binding,
SLC37A4	solute carrier family 37 member 4(SLC37A4)	Homo sapiens	GO:0005215~transporter activity,GO:0015152~glucose-6-phosphate transmembrane transporter activity,GO:0061513~glucose 6-phosphate:inorganic phosphate antiporter activity,
SLC38A8	solute carrier family 38 member 8(SLC38A8)	Homo sapiens	GO:0015171~amino acid transmembrane transporter activity,
SLC4A11	solute carrier family 4 member 11(SLC4A11)	Homo sapiens	GO:0005272~sodium channel activity,GO:0005452~inorganic anion exchanger activity,GO:0015106~bicarbonate transmembrane transporter activity,GO:0015252~hydrogen ion channel activity,GO:0015293~symporter activity,GO:0015301~anion:anion antiporter activity,GO:0046715~borate transmembrane transporter activity,GO:0046983~protein dimerization activity,
SLC5A4	solute carrier family 5 member 4(SLC5A4)	Homo sapiens	GO:0005215~transporter activity,GO:0005412~glucose:sodium symporter activity,
SLC6A12	solute carrier family 6 member 12(SLC6A12)	Homo sapiens	GO:0005328~neurotransmitter:sodium symporter activity,GO:0005332~gamma-aminobutyric acid:sodium symporter activity,GO:0005515~protein binding,GO:0015171~amino acid transmembrane transporter activity,
SLC6A17	solute carrier family 6 member 17(SLC6A17)	Homo sapiens	GO:0005328~neurotransmitter:sodium symporter activity,GO:0015171~amino acid transmembrane transporter activity,
SLC6A20	solute carrier family 6 member 20(SLC6A20)	Homo sapiens	GO:0005328~neurotransmitter:sodium symporter activity,GO:0005515~protein binding,GO:0015171~amino acid transmembrane transporter activity,GO:0015375~glycine:sodium symporter activity,
SLC6A4	solute carrier family 6 member 4(SLC6A4)	Homo sapiens	GO:0005328~neurotransmitter:sodium symporter activity,GO:0005335~serotonin:sodium symporter activity,GO:0005515~protein binding,GO:0008504~monoamine transmembrane transporter activity,GO:0015222~serotonin transmembrane transporter activity,GO:0017022~myosin binding,GO:0017075~syntaxin-1 binding,GO:0017137~Rab GTPase binding,GO:0019811~cocaine binding,GO:0042803~protein homodimerization activity,GO:0046872~metal ion binding,GO:0050998~nitric-oxide synthase binding,GO:0051015~actin filament binding,
SLC8A1	solute carrier family 8 member A1(SLC8A1)	Homo sapiens	GO:0005432~calcium:sodium antiporter activity,GO:0005509~calcium ion binding,GO:0005515~protein binding,GO:0005516~calmodulin binding,GO:0008092~cytoskeletal protein binding,GO:0030506~ankyrin binding,GO:0044325~ion channel binding,
SLC9A8	solute carrier family 9 member A8(SLC9A8)	Homo sapiens	GO:0015385~sodium:proton antiporter activity,GO:0015386~potassium:proton antiporter activity,
SLCO2A1	solute carrier organic anion transporter family member 2A1(SLCO2A1)	Homo sapiens	GO:0005215~transporter activity,GO:0005319~lipid transporter activity,GO:0015132~prostaglandin transmembrane transporter activity,GO:0015347~sodium-independent organic anion transmembrane transporter activity,
SSTR2	somatostatin receptor 2(SSTR2)	Homo sapiens	GO:0004994~somatostatin receptor activity,GO:0005515~protein binding,GO:0030165~PDZ domain binding,GO:0042923~neuropeptide binding,
SORBS2	sorbin and SH3 domain containing 2(SORBS2)	Homo sapiens	GO:0005200~structural constituent of cytoskeleton,GO:0005515~protein binding,GO:0008093~cytoskeletal adaptor activity,GO:0008307~structural constituent of muscle,GO:0044822~poly(A) RNA binding,GO:0046872~metal ion binding,
SORD	sorbitol dehydrogenase(SORD)	Homo sapiens	GO:0003939~L-iditol 2-dehydrogenase activity,GO:0008270~zinc ion binding,GO:0016491~oxidoreductase activity,GO:0030246~carbohydrate binding,GO:0042802~identical protein binding,GO:0046526~D-xylulose reductase activity,GO:0051287~NAD binding,
SORCS2	sortilin related VPS10 domain containing receptor 2(SORCS2)	Homo sapiens	GO:0008188~neuropeptide receptor activity,
SPTAN1	spectrin alpha, non-erythrocytic 1(SPTAN1)	Homo sapiens	GO:0003779~actin binding,GO:0005088~Ras guanyl-nucleotide exchange factor activity,GO:0005200~structural constituent of cytoskeleton,GO:0005509~calcium ion binding,GO:0005515~protein binding,GO:0005516~calmodulin binding,GO:0098641~cadherin binding involved in cell-cell adhesion,
SPTBN2	spectrin beta, non-erythrocytic 2(SPTBN2)	Homo sapiens	GO:0003779~actin binding,GO:0005088~Ras guanyl-nucleotide exchange factor activity,GO:0005200~structural constituent of cytoskeleton,GO:0005543~phospholipid binding,GO:0098641~cadherin binding involved in cell-cell adhesion,
SYNE3	spectrin repeat containing nuclear envelope family member 3(SYNE3)	Homo sapiens	GO:0005515~protein binding,GO:0051015~actin filament binding,
SPDYE3	speedy/RINGO cell cycle regulator family member E3(SPDYE3)	Homo sapiens	GO:0019901~protein kinase binding,
SPATA17	spermatogenesis associated 17(SPATA17)	Homo sapiens	GO:0005516~calmodulin binding,
SPATA18	spermatogenesis associated 18(SPATA18)	Homo sapiens	GO:0005515~protein binding,
SPATA21	spermatogenesis associated 21(SPATA21)	Homo sapiens	GO:0005509~calcium ion binding,

SMOX	spermine oxidase(SMOX)	Homo sapiens	GO:0016491~oxidoreductase activity,GO:0046592~polyamine oxidase activity,GO:0052894~norspermine:oxygen oxidoreductase activity,GO:0052895~N1-acetylspermine:oxygen oxidoreductase (N1-acetylspermidine-forming) activity,GO:0052901~spermine:oxygen oxidoreductase (spermidine-forming) activity,
SPIRE2	spire type actin nucleation factor 2(SPIRE2)	Homo sapiens	GO:0003779~actin binding,
SF3B1	splicing factor 3b subunit 1(SF3B1)	Homo sapiens	GO:0003729~mRNA binding,GO:0005515~protein binding,GO:0044822~poly(A) RNA binding,
SREBF1	sterol regulatory element binding transcription factor 1(SREBF1)	Homo sapiens	GO:0000978~RNA polymerase II core promoter proximal region sequence-specific DNA binding,GO:0001077~transcriptional activator activity, RNA polymerase II core promoter proximal region sequence-specific binding,GO:0003677~DNA binding,GO:0003682~chromatin binding,GO:0003700~transcription factor activity, sequence-specific DNA binding,GO:0005515~protein binding,GO:0019901~protein kinase binding,GO:0032403~protein complex binding,GO:0032810~sterol response element binding,GO:0046983~protein dimerization activity,
STRA6	stimulated by retinoic acid 6(STRA6)	Homo sapiens	GO:0004872~receptor activity,GO:0051183~vitamin transporter activity,
SULT1A1	sulfotransferase family 1A member 1(SULT1A1)	Homo sapiens	GO:0004062~aryl sulfotransferase activity,GO:0005515~protein binding,GO:0008146~sulfotransferase activity,GO:0047894~flavonol 3-sulfotransferase activity,GO:0050294~steroid sulfotransferase activity,
SULT2B1	sulfotransferase family 2B member 1(SULT2B1)	Homo sapiens	GO:0004027~alcohol sulfotransferase activity,GO:0005515~protein binding,GO:0050294~steroid sulfotransferase activity,
SOCS3	suppressor of cytokine signaling 3(SOCS3)	Homo sapiens	GO:0004860~protein kinase inhibitor activity,GO:0005515~protein binding,
SFTPB	surfactant protein B(SFTPB)	Homo sapiens	GO:0001664~G-protein coupled receptor binding,GO:0008047~enzyme activator activity,
SMN1	survival of motor neuron 1, telomeric(SMN1)	Homo sapiens	GO:0003723~RNA binding,GO:0005515~protein binding,GO:0042802~identical protein binding,
SYNDIG1	synapse differentiation inducing 1(SYNDIG1)	Homo sapiens	GO:0005515~protein binding,GO:0035254~glutamate receptor binding,GO:0042803~protein homodimerization activity,
SYNGR1	synaptogyrin 1(SYNGR1)	Homo sapiens	GO:0005515~protein binding,
SYT8	synaptotagmin 8(SYT8)	Homo sapiens	GO:0005509~calcium ion binding,GO:0005544~calcium-dependent phospholipid binding,GO:0019905~syntaxin binding,GO:0030276~clathrin binding,GO:0048306~calcium-dependent protein binding,
SYTL1	synaptotagmin like 1(SYTL1)	Homo sapiens	GO:0005509~calcium ion binding,GO:0005515~protein binding,GO:0005544~calcium-dependent phospholipid binding,GO:0017137~Rab GTPase binding,GO:0019905~syntaxin binding,GO:0030276~clathrin binding,GO:0042043~neurexin family protein binding,
SYTL5	synaptotagmin like 5(SYTL5)	Homo sapiens	GO:0005509~calcium ion binding,GO:0005515~protein binding,GO:0005544~calcium-dependent phospholipid binding,GO:0017137~Rab GTPase binding,GO:0019905~syntaxin binding,GO:0030276~clathrin binding,
STX11	syntaxin 11(STX11)	Homo sapiens	GO:0000149~SNARE binding,GO:0005484~SNAP receptor activity,GO:0005515~protein binding,
STX1B	syntaxin 1B(STX1B)	Homo sapiens	GO:0000149~SNARE binding,GO:0005484~SNAP receptor activity,GO:0019901~protein kinase binding,GO:0019904~protein domain specific binding,
TSEN2	tRNA splicing endonuclease subunit 2(TSEN2)	Homo sapiens	GO:0000213~tRNA-intron endonuclease activity,GO:0003676~nucleic acid binding,GO:0005515~protein binding,GO:0016829~lyase activity,
TAZ	tafazzin(TAZ)	Homo sapiens	GO:0003841~1-acylglycerol-3-phosphate O-acyltransferase activity,GO:0008374~O-acyltransferase activity,GO:0016746~transferase activity, transferring acyl groups,GO:0047184~1-acylglycerophosphocholine O-acyltransferase activity,
TLN2	talin 2(TLN2)	Homo sapiens	GO:0003779~actin binding,GO:0005198~structural molecule activity,GO:0005200~structural constituent of cytoskeleton,GO:0005515~protein binding,GO:0051015~actin filament binding,
TOM1L2	target of myb1 like 2 membrane trafficking protein(TOM1L2)	Homo sapiens	GO:0005515~protein binding,GO:0019901~protein kinase binding,GO:0030276~clathrin binding,
TECPR1	tectonin beta-propeller repeat containing 1(TECPR1)	Homo sapiens	GO:0005515~protein binding,GO:0032266~phosphatidylinositol-3-phosphate binding,
TECTA	tectorin alpha(TECTA)	Homo sapiens	GO:0005201~extracellular matrix structural constituent,
TERT	telomerase reverse transcriptase(TERT)	Homo sapiens	GO:0000049~tRNA binding,GO:0001223~transcription coactivator binding,GO:0003677~DNA binding,GO:0003720~telomerase activity,GO:0003721~telomerase RNA reverse transcriptase activity,GO:0003723~RNA binding,GO:0003964~RNA-directed DNA polymerase activity,GO:0003968~RNA-directed RNA polymerase activity,GO:0005515~protein binding,GO:0016779~nucleotidyltransferase activity,GO:0042162~telomeric DNA binding,GO:0042803~protein homodimerization activity,GO:0046872~metal ion binding,GO:0070034~telomerase RNA binding,
TSPAN10	tetraspanin 10(TSPAN10)	Homo sapiens	GO:0019899~enzyme binding,
TSPAN2	tetraspanin 2(TSPAN2)	Homo sapiens	GO:0005515~protein binding,
THNSL1	threonine synthase like 1(THNSL1)	Homo sapiens	GO:0004795~threonine synthase activity,GO:0030170~pyridoxal phosphate binding,
THNSL2	threonine synthase like 2(THNSL2)	Homo sapiens	GO:0004795~threonine synthase activity,GO:0005125~cytokine activity,GO:0016829~lyase activity,GO:0030170~pyridoxal phosphate binding,GO:0070905~serine binding,
TARS2	threonyl-tRNA synthetase 2, mitochondrial (putative)(TARS2)	Homo sapiens	GO:0003723~RNA binding,GO:0004829~threonine-tRNA ligase activity,GO:0005524~ATP binding,
THBS3	thrombospondin 3(THBS3)	Homo sapiens	GO:0005509~calcium ion binding,GO:0008201~heparin binding,
TBXA2R	thromboxane A2 receptor(TBXA2R)	Homo sapiens	GO:0004960~thromboxane receptor activity,GO:0004961~thromboxane A2 receptor activity,GO:0005085~guanyl-nucleotide exchange factor activity,GO:0005515~protein binding,
TYMP	thymidine phosphorylase(TYMP)	Homo sapiens	GO:0004645~phosphorylase activity,GO:0008083~growth factor activity,GO:0009032~thymidine phosphorylase activity,GO:0016154~pyrimidine-nucleoside phosphorylase activity,GO:0016763~transferase activity, transferring pentosyl groups,
THEMIS2	thymocyte selection associated family member 2(THEMIS2)	Homo sapiens	GO:0005515~protein binding,
TOX	thymocyte selection associated high mobility group box(TOX)	Homo sapiens	GO:0003677~DNA binding,
TRIP13	thyroid hormone receptor interactor 13(TRIP13)	Homo sapiens	GO:0003712~transcription cofactor activity,GO:0005515~protein binding,GO:0005524~ATP binding,GO:0042802~identical protein binding,
THRSP	thyroid hormone responsive(THRSP)	Homo sapiens	GO:0042802~identical protein binding,GO:0042803~protein homodimerization activity,
TJP3	tight junction protein 3(TJP3)	Homo sapiens	GO:0005515~protein binding,
TSTA3	tissue specific transplantation antigen P35B(TSTA3)	Homo sapiens	GO:0003824~catalytic activity,GO:0009055~electron carrier activity,GO:0016491~oxidoreductase activity,GO:0016853~isomerase activity,GO:0042356~GDP-4-dehydro-D-rhamnose reductase activity,GO:0050577~GDP-L-fucose synthase activity,GO:0050662~coenzyme binding,
TLR5	toll like receptor 5(TLR5)	Homo sapiens	GO:0004872~receptor activity,GO:0004888~transmembrane signaling receptor activity,GO:0005149~interleukin-1 receptor binding,
TLL1	tolloid like 1(TLL1)	Homo sapiens	GO:0004222~metalloendopeptidase activity,GO:0004252~serine-type endopeptidase activity,GO:0005509~calcium ion binding,GO:0008270~zinc ion binding,

TOP2A	topoisomerase (DNA) II alpha(TOP2A)	Homo sapiens	GO:0000287~magnesium ion binding,GO:0003677~DNA binding,GO:0003682~chromatin binding,GO:0003918~DNA topoisomerase type II (ATP-hydrolyzing) activity,GO:0005080~protein kinase C binding,GO:0005515~protein binding,GO:0005524~ATP binding,GO:0008022~protein C-terminus binding,GO:0008094~DNA-dependent ATPase activity,GO:0008144~drug binding,GO:0008301~DNA binding, bending,GO:0019899~enzyme binding,GO:0042803~protein homodimerization activity,GO:0042826~histone deacetylase binding,GO:0043130~ubiquitin binding,GO:0044822~poly(A) RNA binding,GO:0046982~protein heterodimerization activity,
TRAK2	trafficking kinesin protein 2(TRAK2)	Homo sapiens	GO:0005102~receptor binding,GO:0005515~protein binding,GO:0019899~enzyme binding,GO:0050811~GABA receptor binding,
TRAPPC1	trafficking protein particle complex 13(TRAPPC13)	Homo sapiens	GO:0005515~protein binding,
TRAPPC2	trafficking protein particle complex 2(TRAPPC2)	Homo sapiens	GO:0005515~protein binding,GO:0008134~transcription factor binding,GO:0044325~ion channel binding,
TCEANC2	transcription elongation factor A N-terminal and central domain containing 2(TCEANC2)	Homo sapiens	GO:0003677~DNA binding,GO:0005515~protein binding,
TCEA2	transcription elongation factor A2(TCEA2)	Homo sapiens	GO:0003677~DNA binding,GO:0003746~translation elongation factor activity,GO:0005515~protein binding,GO:0008270~zinc ion binding,
TCF24	transcription factor 24(TCF24)	Homo sapiens	GO:0003677~DNA binding,GO:0046983~protein dimerization activity,
TCF7	transcription factor 7 (T-cell specific, HMG-box)(TCF7)	Homo sapiens	GO:0001047~core promoter binding,GO:0001227~transcriptional repressor activity, RNA polymerase II transcription regulatory region sequence-specific binding,GO:0005515~protein binding,GO:0008013~beta-catenin binding,GO:0043565~sequence-specific DNA binding,GO:0044212~transcription regulatory region DNA binding,
TFAP2E	transcription factor AP-2 epsilon(TFAP2E)	Homo sapiens	GO:0000977~RNA polymerase II regulatory region sequence-specific DNA binding,GO:0000978~RNA polymerase II core promoter proximal region sequence-specific DNA binding,GO:0000981~RNA polymerase II transcription factor activity, sequence-specific DNA binding,GO:0001077~transcriptional activator activity, RNA polymerase II core promoter proximal region sequence-specific binding,GO:0042803~protein homodimerization activity,GO:0046982~protein heterodimerization activity,
TLE1	transducin like enhancer of split 1(TLE1)	Homo sapiens	GO:0001106~RNA polymerase II transcription corepressor activity,GO:0005515~protein binding,GO:0008134~transcription factor binding,GO:0042802~identical protein binding,
TRPC4AP	transient receptor potential cation channel subfamily C member 4 associated protein(TRPC4AP)	Homo sapiens	GO:0005262~calcium channel activity,GO:0005515~protein binding,GO:0019902~phosphatase binding,
TMPRSS3	transmembrane protease, serine 3(TMPRSS3)	Homo sapiens	GO:0004252~serine-type endopeptidase activity,GO:0005044~scavenger receptor activity,GO:0008233~peptidase activity,GO:0017080~sodium channel regulator activity,
TMEM17	transmembrane protein 175(TMEM175)	Homo sapiens	GO:0005267~potassium channel activity,GO:0022841~potassium ion leak channel activity,
TMEM21	transmembrane protein 218(TMEM218)	Homo sapiens	GO:0005515~protein binding,
TMEM37	transmembrane protein 37(TMEM37)	Homo sapiens	GO:0005244~voltage-gated ion channel activity,GO:0005262~calcium channel activity,
TMEM38	transmembrane protein 38A(TMEM38A)	Homo sapiens	GO:0005261~cation channel activity,GO:0005267~potassium channel activity,GO:0015269~calcium-activated potassium channel activity,
TRIB1	tribbles pseudokinase 1(TRIB1)	Homo sapiens	GO:0004672~protein kinase activity,GO:0004860~protein kinase inhibitor activity,GO:0005515~protein binding,GO:0005524~ATP binding,GO:0008134~transcription factor binding,GO:0031434~mitogen-activated protein kinase kinase binding,GO:0031625~ubiquitin protein ligase binding,GO:0055106~ubiquitin-protein transferase regulator activity,
TKFC	triokinase and FMN cyclase(TKFC)	Homo sapiens	GO:0004371~glycerone kinase activity,GO:0005515~protein binding,GO:0005524~ATP binding,GO:0034012~FAD-AMP lyase (cyclizing) activity,GO:0046872~metal ion binding,GO:0050354~triokinase activity,
TRIM14	tripartite motif containing 14(TRIM14)	Homo sapiens	GO:0008270~zinc ion binding,
TRIM46	tripartite motif containing 46(TRIM46)	Homo sapiens	GO:0008270~zinc ion binding,
TRIM65	tripartite motif containing 65(TRIM65)	Homo sapiens	GO:0008270~zinc ion binding,
TRIM66	tripartite motif containing 66(TRIM66)	Homo sapiens	GO:0008270~zinc ion binding,
TRIM72	tripartite motif containing 72(TRIM72)	Homo sapiens	GO:0001786~phosphatidylserine binding,GO:0005515~protein binding,GO:0008270~zinc ion binding,GO:0031624~ubiquitin conjugating enzyme binding,GO:0061630~ubiquitin protein ligase activity,
TMOD2	tropomodulin 2(TMOD2)	Homo sapiens	GO:0003779~actin binding,GO:0005523~tropomyosin binding,
TNNI1	troponin I1, slow skeletal type(TNNI1)	Homo sapiens	GO:0003779~actin binding,GO:0005515~protein binding,GO:0046872~metal ion binding,
TNNI3	troponin I3, cardiac type(TNNI3)	Homo sapiens	GO:0003779~actin binding,GO:0005515~protein binding,GO:0019855~calcium channel inhibitor activity,GO:0019901~protein kinase binding,GO:0019904~protein domain specific binding,GO:0030172~troponin C binding,GO:0031014~troponin T binding,GO:0046872~metal ion binding,GO:0048306~calcium-dependent protein binding,
TYSND1	trypsin domain containing 1(TYSND1)	Homo sapiens	GO:0002020~protease binding,GO:0004252~serine-type endopeptidase activity,
TPH2	tryptophan hydroxylase 2(TPH2)	Homo sapiens	GO:0004497~monooxygenase activity,GO:0004510~tryptophan 5-monooxygenase activity,GO:0005506~iron ion binding,GO:0016597~amino acid binding,GO:0016714~oxidoreductase activity, acting on paired donors, with incorporation or reduction of molecular oxygen, reduced pteridine as one donor, and incorporation of one atom of oxygen,
TUB	tubby bipartite transcription factor(TUB)	Homo sapiens	GO:0032403~protein complex binding,GO:0035091~phosphatidylinositol binding,
TSC2	tuberous sclerosis 2(TSC2)	Homo sapiens	GO:0005096~GTPase activator activity,GO:0005515~protein binding,GO:0019902~phosphatase binding,GO:0031267~small GTPase binding,GO:0042803~protein homodimerization activity,
TUBA4A	tubulin alpha 4a(TUBA4A)	Homo sapiens	GO:0003924~GTPase activity,GO:0005200~structural constituent of cytoskeleton,GO:0005515~protein binding,GO:0005525~GTP binding,GO:0019899~enzyme binding,
TUBB8	tubulin beta 8 class VIII(TUBB8)	Homo sapiens	GO:0003924~GTPase activity,GO:0005200~structural constituent of cytoskeleton,GO:0005525~GTP binding,
TBCD	tubulin folding cofactor D(TBCD)	Homo sapiens	GO:0005096~GTPase activator activity,GO:0005515~protein binding,GO:0048487~beta-tubulin binding,GO:0051087~chaperone binding,
TPPP3	tubulin polymerization promoting protein family member 3(TPPP3)	Homo sapiens	GO:0015631~tubulin binding,
TTLL1	tubulin tyrosine ligase like 1(TTLL1)	Homo sapiens	GO:0005524~ATP binding,GO:0016874~ligase activity,GO:0070740~tubulin-glutamic acid ligase activity,
TTLL10	tubulin tyrosine ligase like 10(TTLL10)	Homo sapiens	GO:0005515~protein binding,GO:0005524~ATP binding,GO:0070735~protein-glycine ligase activity,
TTLL12	tubulin tyrosine ligase like 12(TTLL12)	Homo sapiens	GO:0005524~ATP binding,GO:0016874~ligase activity,
TDRD1	tudor domain containing 1(TDRD1)	Homo sapiens	GO:0046872~metal ion binding,
TUFT1	tuftelin 1(TUFT1)	Homo sapiens	GO:0005515~protein binding,GO:0030345~structural constituent of tooth enamel,
TFIP11	tuftelin interacting protein 11(TFIP11)	Homo sapiens	GO:0003676~nucleic acid binding,GO:0003677~DNA binding,GO:0005515~protein binding,
TNFSF12	tumor necrosis factor superfamily member 12(TNFSF12)	Homo sapiens	GO:0005102~receptor binding,GO:0005125~cytokine activity,GO:0005164~tumor necrosis factor receptor binding,GO:0005515~protein binding,
TNFSF15	tumor necrosis factor superfamily member 15(TNFSF15)	Homo sapiens	GO:0005102~receptor binding,GO:0005125~cytokine activity,GO:0005164~tumor necrosis factor receptor binding,

TNFSF9	tumor necrosis factor superfamily member 9(TNFSF9)	Homo sapiens	GO:0005102~receptor binding,GO:0005125~cytokine activity,GO:0005164~tumor necrosis factor receptor binding,GO:0032813~tumor necrosis factor receptor superfamily binding,
TWIST2	twist family bHLH transcription factor 2(TWIST2)	Homo sapiens	GO:0003677~DNA binding,GO:0005515~protein binding,GO:0046983~protein dimerization activity,
UBAP1	ubiquitin associated protein 1(UBAP1)	Homo sapiens	GO:0005515~protein binding,GO:0043130~ubiquitin binding,
UBE2L6	ubiquitin conjugating enzyme E2 L6(UBE2L6)	Homo sapiens	GO:0004842~ubiquitin-protein transferase activity,GO:0005515~protein binding,GO:0005524~ATP binding,GO:0042296~ISG15 transferase activity,GO:0043130~ubiquitin binding,
UBLCP1	ubiquitin like domain containing CTD phosphatase 1(UBLCP1)	Homo sapiens	GO:0004722~protein serine/threonine phosphatase activity,GO:0005515~protein binding,
UBR7	ubiquitin protein ligase E3 component n-recognin 7 (putative)(UBR7)	Homo sapiens	GO:0008270~zinc ion binding,GO:0016874~ligase activity,
UNC93B1	unc-93 homolog B1 (C. elegans)(UNC93B1)	Homo sapiens	GO:0035325~Toll-like receptor binding,
SGK494	uncharacterized serine/threonine-protein kinase SgK494(SGK494)	Homo sapiens	GO:0004672~protein kinase activity,GO:0004674~protein serine/threonine kinase activity,GO:0005524~ATP binding,
UPK2	uroplakin 2(UPK2)	Homo sapiens	GO:0005515~protein binding,
UROD	uroporphyrinogen decarboxylase(UROD)	Homo sapiens	GO:0004853~uroporphyrinogen decarboxylase activity,GO:0005515~protein binding,
VCPKMT	valosin containing protein lysine methyltransferase(VCPKMT)	Homo sapiens	GO:0005515~protein binding,GO:0016279~protein-lysine N-methyltransferase activity,
VEGFC	vascular endothelial growth factor C(VEGFC)	Homo sapiens	GO:0005515~protein binding,GO:0008083~growth factor activity,GO:0042056~chemoattractant activity,GO:0043185~vascular endothelial growth factor receptor 3 binding,
VEGFD	vascular endothelial growth factor D(VEGFD)	Homo sapiens	GO:0005161~platelet-derived growth factor receptor binding,GO:0005172~vascular endothelial growth factor receptor binding,GO:0005515~protein binding,GO:0008083~growth factor activity,GO:0042056~chemoattractant activity,GO:0042803~protein homodimerization activity,GO:0043185~vascular endothelial growth factor receptor 3 binding,
VASH1	vasohibin 1(VASH1)	Homo sapiens	GO:0005515~protein binding,
VAV2	vav guanine nucleotide exchange factor 2(VAV2)	Homo sapiens	GO:0005085~guanyl-nucleotide exchange factor activity,GO:0005089~Rho guanyl-nucleotide exchange factor activity,GO:0005154~epidermal growth factor receptor binding,GO:0005515~protein binding,GO:0046872~metal ion binding,
VTN	vitronectin(VTN)	Homo sapiens	GO:0005044~scavenger receptor activity,GO:0005178~integrin binding,GO:0005515~protein binding,GO:0005518~collagen binding,GO:0008201~heparin binding,GO:0030247~polysaccharide binding,GO:0042802~identical protein binding,GO:0050840~extracellular matrix binding,
VN1R1	vomeronasal 1 receptor 1(VN1R1)	Homo sapiens	GO:0016503~pheromone receptor activity,
VHLL	von Hippel-Lindau tumor suppressor like(VHLL)	Homo sapiens	GO:0005515~protein binding,GO:0061630~ubiquitin protein ligase activity,
VWA2	von Willebrand factor A domain containing 2(VWA2)	Homo sapiens	GO:0005509~calcium ion binding,GO:0005515~protein binding,GO:0042802~identical protein binding,
YBEY	ybeY metallopeptidase (putative)(YBEY)	Homo sapiens	GO:0004222~metalloendopeptidase activity,GO:0046872~metal ion binding,
YRDC	yrdC N6-threonylcarbamoyltransferase domain containing(YRDC)	Homo sapiens	GO:0000049~tRNA binding,GO:0003725~double-stranded RNA binding,GO:0016779~nucleotidyltransferase activity,
ZFAND2A	zinc finger AN1-type containing 2A(ZFAND2A)	Homo sapiens	GO:0008270~zinc ion binding,
ZBBX	zinc finger B-box domain containing(ZBBX)	Homo sapiens	GO:0008270~zinc ion binding,
ZBED2	zinc finger BED-type containing 2(ZBED2)	Homo sapiens	GO:0003677~DNA binding,GO:0046872~metal ion binding,
ZBED6	zinc finger BED-type containing 6(ZBED6)	Homo sapiens	GO:0000977~RNA polymerase II regulatory region sequence-specific DNA binding,GO:0003700~transcription factor activity, sequence-specific DNA binding,GO:0046872~metal ion binding,GO:0046983~protein dimerization activity,
ZC3H12A	zinc finger CCCH-type containing 12A(ZC3H12A)	Homo sapiens	GO:0003677~DNA binding,GO:0003682~chromatin binding,GO:0003723~RNA binding,GO:0003729~mRNA binding,GO:0003730~mRNA 3'-UTR binding,GO:0004521~endoribonuclease activity,GO:0004532~exoribonuclease activity,GO:0004540~ribonuclease activity,GO:0004843~thiol-dependent ubiquitin-specific protease activity,GO:0005515~protein binding,GO:0035198~miRNA binding,GO:0035613~RNA stem-loop binding,GO:0035925~mRNA 3'-UTR AU-rich region binding,GO:0043022~ribosome binding,GO:0046872~metal ion binding,
ZCCHC24	zinc finger CCHC-type containing 24(ZCCHC24)	Homo sapiens	GO:0003676~nucleic acid binding,GO:0008270~zinc ion binding,GO:0044822~poly(A) RNA binding,
ZDHHC1	zinc finger DHHC-type containing 1(ZDHHC1)	Homo sapiens	GO:0003677~DNA binding,GO:0008270~zinc ion binding,GO:0016409~palmitoyltransferase activity,GO:0019706~protein-cysteine S-palmitoyltransferase activity,
ZDHHC15	zinc finger DHHC-type containing 15(ZDHHC15)	Homo sapiens	GO:0008270~zinc ion binding,GO:0016409~palmitoyltransferase activity,GO:0019706~protein-cysteine S-palmitoyltransferase activity,
ZDHHC19	zinc finger DHHC-type containing 19(ZDHHC19)	Homo sapiens	GO:0008270~zinc ion binding,GO:0019706~protein-cysteine S-palmitoyltransferase activity,
ZEB1	zinc finger E-box binding homeobox 1(ZEB1)	Homo sapiens	GO:0001227~transcriptional repressor activity, RNA polymerase II transcription regulatory region sequence-specific binding,GO:0003676~nucleic acid binding,GO:0003677~DNA binding,GO:0003682~chromatin binding,GO:0003700~transcription factor activity, sequence-specific DNA binding,GO:0003713~transcription coactivator activity,GO:0003714~transcription corepressor activity,GO:0005515~protein binding,GO:0008134~transcription factor binding,GO:0008270~zinc ion binding,GO:0046872~metal ion binding,GO:0070888~E-box binding,
ZFYVE28	zinc finger FYVE-type containing 28(ZFYVE28)	Homo sapiens	GO:0005515~protein binding,GO:0032266~phosphatidylinositol-3-phosphate binding,GO:0046872~metal ion binding,
ZNHIT3	zinc finger HIT-type containing 3(ZNHIT3)	Homo sapiens	GO:0005515~protein binding,GO:0046872~metal ion binding,GO:0046966~thyroid hormone receptor binding,
ZMYM3	zinc finger MYM-type containing 3(ZMYM3)	Homo sapiens	GO:0000981~RNA polymerase II transcription factor activity, sequence-specific DNA binding,GO:0003677~DNA binding,GO:0008270~zinc ion binding,
ZSWIM6	zinc finger SWIM-type containing 6(ZSWIM6)	Homo sapiens	GO:0008270~zinc ion binding,
ZBTB12	zinc finger and BTB domain containing 12(ZBTB12)	Homo sapiens	GO:0003677~DNA binding,GO:0046872~metal ion binding,
ZBTB47	zinc finger and BTB domain containing 47(ZBTB47)	Homo sapiens	GO:0003676~nucleic acid binding,GO:0003677~DNA binding,GO:0046872~metal ion binding,
ZFHX4	zinc finger homeobox 4(ZFHX4)	Homo sapiens	GO:0008270~zinc ion binding,GO:0043565~sequence-specific DNA binding,GO:0046872~metal ion binding,
ZMAT1	zinc finger matrin-type 1(ZMAT1)	Homo sapiens	GO:0002039~p53 binding,GO:0003677~DNA binding,GO:0003723~RNA binding,GO:0008270~zinc ion binding,GO:0046872~metal ion binding,
ZNF117	zinc finger protein 117(ZNF117)	Homo sapiens	GO:0000978~RNA polymerase II core promoter proximal region sequence-specific DNA binding,GO:0003676~nucleic acid binding,GO:0003700~transcription factor activity, sequence-specific DNA binding,GO:0008270~zinc ion binding,GO:0046872~metal ion binding,
ZNF142	zinc finger protein 142(ZNF142)	Homo sapiens	GO:0003676~nucleic acid binding,GO:0003677~DNA binding,GO:0046872~metal ion binding,
ZNF154	zinc finger protein 154(ZNF154)	Homo sapiens	GO:0000977~RNA polymerase II regulatory region sequence-specific DNA binding,GO:0003676~nucleic acid binding,GO:0003700~transcription factor activity, sequence-specific DNA binding,GO:0046872~metal ion binding,

ZNF16	zinc finger protein 16(ZNF16)	Homo sapiens	GO:0003677~DNA binding,GO:0003700~transcription factor activity, sequence-specific DNA binding,GO:0005515~protein binding,GO:0046872~metal ion binding,
ZNF185	zinc finger protein 185 (LIM domain)(ZNF185)	Homo sapiens	GO:0008270~zinc ion binding,
ZNF222	zinc finger protein 222(ZNF222)	Homo sapiens	GO:0003676~nucleic acid binding,GO:0003677~DNA binding,GO:0046872~metal ion binding,
ZNF227	zinc finger protein 227(ZNF227)	Homo sapiens	GO:0003676~nucleic acid binding,GO:0003677~DNA binding,GO:0003700~transcription factor activity, sequence-specific DNA binding,GO:0005515~protein binding,GO:0046872~metal ion binding,
ZNF253	zinc finger protein 253(ZNF253)	Homo sapiens	GO:0000978~RNA polymerase II core promoter proximal region sequence-specific DNA binding,GO:0003676~nucleic acid binding,GO:0008270~zinc ion binding,GO:0046872~metal ion binding,
ZNF263	zinc finger protein 263(ZNF263)	Homo sapiens	GO:0003676~nucleic acid binding,GO:0003700~transcription factor activity, sequence-specific DNA binding,GO:0005515~protein binding,GO:0043565~sequence-specific DNA binding,GO:0046872~metal ion binding,
ZNF317	zinc finger protein 317(ZNF317)	Homo sapiens	GO:0003676~nucleic acid binding,GO:0003677~DNA binding,GO:0003700~transcription factor activity, sequence-specific DNA binding,GO:0005515~protein binding,GO:0046872~metal ion binding,
ZNF329	zinc finger protein 329(ZNF329)	Homo sapiens	GO:0003676~nucleic acid binding,GO:0003677~DNA binding,GO:0005515~protein binding,GO:0046872~metal ion binding,
ZNF35	zinc finger protein 35(ZNF35)	Homo sapiens	GO:0003677~DNA binding,GO:0003700~transcription factor activity, sequence-specific DNA binding,GO:0043565~sequence-specific DNA binding,GO:0046872~metal ion binding,
ZNF350	zinc finger protein 350(ZNF350)	Homo sapiens	GO:0001162~RNA polymerase II intronic transcription regulatory region sequence-specific DNA binding,GO:0001227~transcriptional repressor activity, RNA polymerase II transcription regulatory region sequence-specific binding,GO:0003676~nucleic acid binding,GO:0003677~DNA binding,GO:0005515~protein binding,GO:0046872~metal ion binding,
ZNF383	zinc finger protein 383(ZNF383)	Homo sapiens	GO:0003676~nucleic acid binding,GO:0003677~DNA binding,GO:0003700~transcription factor activity, sequence-specific DNA binding,GO:0046872~metal ion binding,
ZNF385D	zinc finger protein 385D(ZNF385D)	Homo sapiens	GO:0002039~p53 binding,GO:0003723~RNA binding,GO:0008270~zinc ion binding,
ZNF396	zinc finger protein 396(ZNF396)	Homo sapiens	GO:0003700~transcription factor activity, sequence-specific DNA binding,GO:0043565~sequence-specific DNA binding,GO:0046872~metal ion binding,
ZNF408	zinc finger protein 408(ZNF408)	Homo sapiens	GO:0003676~nucleic acid binding,GO:0003677~DNA binding,GO:0005515~protein binding,GO:0042802~identical protein binding,GO:0046872~metal ion binding,
ZNF430	zinc finger protein 430(ZNF430)	Homo sapiens	GO:0000978~RNA polymerase II core promoter proximal region sequence-specific DNA binding,GO:0003676~nucleic acid binding,GO:0046872~metal ion binding,
ZNF432	zinc finger protein 432(ZNF432)	Homo sapiens	GO:0003676~nucleic acid binding,GO:0003677~DNA binding,GO:0003700~transcription factor activity, sequence-specific DNA binding,GO:0046872~metal ion binding,
ZNF461	zinc finger protein 461(ZNF461)	Homo sapiens	GO:0003676~nucleic acid binding,GO:0003677~DNA binding,GO:0003700~transcription factor activity, sequence-specific DNA binding,GO:0046872~metal ion binding,
ZNF473	zinc finger protein 473(ZNF473)	Homo sapiens	GO:0003676~nucleic acid binding,GO:0003677~DNA binding,GO:0005515~protein binding,GO:0046872~metal ion binding,
ZNF485	zinc finger protein 485(ZNF485)	Homo sapiens	GO:0003676~nucleic acid binding,GO:0003677~DNA binding,GO:0046872~metal ion binding,
ZNF488	zinc finger protein 488(ZNF488)	Homo sapiens	GO:0003677~DNA binding,GO:0005515~protein binding,GO:0046872~metal ion binding,
ZNF503	zinc finger protein 503(ZNF503)	Homo sapiens	GO:0003676~nucleic acid binding,GO:0046872~metal ion binding,
ZNF512B	zinc finger protein 512B(ZNF512B)	Homo sapiens	GO:0003677~DNA binding,GO:0005515~protein binding,GO:0046872~metal ion binding,
ZNF534	zinc finger protein 534(ZNF534)	Homo sapiens	GO:0003676~nucleic acid binding,GO:0003677~DNA binding,GO:0003700~transcription factor activity, sequence-specific DNA binding,GO:0046872~metal ion binding,
ZNF572	zinc finger protein 572(ZNF572)	Homo sapiens	GO:0003676~nucleic acid binding,GO:0003677~DNA binding,GO:0005515~protein binding,GO:0046872~metal ion binding,
ZNF573	zinc finger protein 573(ZNF573)	Homo sapiens	GO:0003676~nucleic acid binding,GO:0003677~DNA binding,GO:0003700~transcription factor activity, sequence-specific DNA binding,GO:0046872~metal ion binding,
ZNF654	zinc finger protein 654(ZNF654)	Homo sapiens	GO:0003677~DNA binding,GO:0046872~metal ion binding,
ZNF660	zinc finger protein 660(ZNF660)	Homo sapiens	GO:0000977~RNA polymerase II regulatory region sequence-specific DNA binding,GO:0003700~transcription factor activity, sequence-specific DNA binding,GO:0046872~metal ion binding,
ZNF662	zinc finger protein 662(ZNF662)	Homo sapiens	GO:0003676~nucleic acid binding,GO:0003677~DNA binding,GO:0003712~transcription cofactor activity,GO:0046872~metal ion binding,
ZNF665	zinc finger protein 665(ZNF665)	Homo sapiens	GO:0003677~DNA binding,GO:0046872~metal ion binding,
ZNF669	zinc finger protein 669(ZNF669)	Homo sapiens	GO:0003676~nucleic acid binding,GO:0003677~DNA binding,GO:0046872~metal ion binding,
ZNF681	zinc finger protein 681(ZNF681)	Homo sapiens	GO:0003676~nucleic acid binding,GO:0003677~DNA binding,GO:0003700~transcription factor activity, sequence-specific DNA binding,GO:0046872~metal ion binding,
ZNF776	zinc finger protein 776(ZNF776)	Homo sapiens	GO:0003676~nucleic acid binding,GO:0003677~DNA binding,GO:0046872~metal ion binding,
ZNF799	zinc finger protein 799(ZNF799)	Homo sapiens	GO:0003676~nucleic acid binding,GO:0003677~DNA binding,GO:0046872~metal ion binding,
ZNF823	zinc finger protein 823(ZNF823)	Homo sapiens	GO:0003676~nucleic acid binding,GO:0003677~DNA binding,GO:0046872~metal ion binding,
ZNF844	zinc finger protein 844(ZNF844)	Homo sapiens	GO:0003676~nucleic acid binding,GO:0003677~DNA binding,GO:0005515~protein binding,GO:0046872~metal ion binding,
ZFPM2	zinc finger protein, FOG family member 2(ZFPM2)	Homo sapiens	GO:0001078~transcriptional repressor activity, RNA polymerase II core promoter proximal region sequence-specific binding,GO:0001085~RNA polymerase II transcription factor binding,GO:0001105~RNA polymerase II transcription coactivator activity,GO:0003676~nucleic acid binding,GO:0003677~DNA binding,GO:0003714~transcription corepressor activity,GO:0005515~protein binding,GO:0008134~transcription factor binding,GO:0008270~zinc ion binding,GO:0046872~metal ion binding,

KEGG Analysis

Category	Term	Count	%	PValue	Genes	List Total	Pop Hits	Pop Total	Fold Enrichment	Bonferroni	Benjamini	FDR
KEGG_PATHWAY	hsa04010:MAPK signaling pathway	30	2.05761317	0.00342898	IL1R1, FGFR3, PDGFB, DUSP10, MAP4K2, JMJD7-PLA2G4B, BDNF, RAC2, RAC3, RASGRP2, EGFR, CACNA1I, NR4A1, CACNG4, MAPK11, TAB1, DDIT3, CDC25B, DUSP5, DUSP4, DUSP1, RPS6KA2, JUN, GADD45G, MAPK8IP2, PLA2G4F, GADD45B, DUSP8, GADD45A, MAP3K12	467	253	6879	1.74666317	0.59618822	0.59618822	4.40069386
KEGG_PATHWAY	hsa04724:Glutamatergic synapse	17	1.1659808	0.00406422	PLD2, GRIK1, ADCY6, GRM1, SHANK2, GRM4, GRM3, PLCB3, JMJD7-PLA2G4B, GRM2, GRIN2C, PLA2G4F, GNB3, SLC1A1, GRK3, PLCB2, GNG7	467	114	6879	2.19660769	0.65874997	0.4158339	5.19603759
KEGG_PATHWAY	hsa04020:Calcium signaling pathway	22	1.50891632	0.00925921	EGFR, SLC8A1, PHKG1, MYLK3, CACNA1I, OXTR, MYLK2, GRM1, ITPKA, ORAI3, P2RX7, PLCB3, GRIN2C, PTK2B, ATP2A3, AVPR1B, TBXA2R, PLCD4, PLCD1, HTR2B, PLCB2, MYLK	467	179	6879	1.81041475	0.91420706	0.55895404	11.4746998
KEGG_PATHWAY	hsa04961:Endocrine and other factor-regulated calcium reabsorption	9	0.61728395	0.00973718	PLCB3, AP2A1, ADCY6, ATP1A3, ATP1A2, CALB1, CLTCL1, PLCB2, DNM1	467	45	6879	2.94603854	0.92446912	0.47575881	12.0326434
KEGG_PATHWAY	hsa04360:Axon guidance	17	1.1659808	0.0114569	PLXNA3, EFNB3, EFNB2, EFNA3, PLXNB3, DPYSL5, L1CAM, FES, SLIT1, EPHB2, SEMA6B, RND1, RAC2, RAC3, SEMA3C, NFATC4, SEMA4A	467	127	6879	1.97175808	0.95226458	0.45578795	14.0133309
KEGG_PATHWAY	hsa04921:Oxytocin signaling pathway	19	1.30315501	0.01232455	EGFR, PTGS2, MYLK3, ADCY6, CACNG4, OXTR, NPR1, MYLK2, CAMKK2, KCNJ5, KCNJ4, PLCB3, CDKN1A, JMJD7-PLA2G4B, JUN, PLA2G4F, NFATC4, PLCB2, MYLK	467	150	6879	1.86582441	0.96214138	0.42053466	14.9969327
KEGG_PATHWAY	hsa04115:p53 signaling pathway	11	0.75445816	0.01397948	STEAP3, CDKN1A, CCND2, GADD45G, TSC2, SERPINE1, RPRM, GADD45B, ADGRB1, GADD45A, TP53AIP1	467	67	6879	2.41838985	0.97568404	0.41195161	16.844284
KEGG_PATHWAY	hsa04960:Aldosterone-regulated sodium reabsorption	8	0.54869684	0.01448127	SGK1, ATP1A3, HSD11B2, ATP1A2, SCNN1A, IRS1, SLC9A3R2, KCNJ1	467	39	6879	3.02157799	0.97874166	0.38206731	17.3970404
KEGG_PATHWAY	hsa00260:Glycine, serine and threonine metabolism	8	0.54869684	0.01448127	CBSL, CHDH, SDSL, GAMT, SARDH, CBS, AOC3, GLDC	467	39	6879	3.02157799	0.97874166	0.38206731	17.3970404
KEGG_PATHWAY	hsa04668:TNF signaling pathway	14	0.96021948	0.02804271	TRAF1, IL18R1, PTGS2, SOCS3, EDN1, CREB5, MAPK11, BIRC3, TAB1, JUNB, CASP10, NOD2, JUN, MLKL	467	107	6879	1.92731494	0.9994519	0.56583915	31.1109723
KEGG_PATHWAY	hsa04726:Serotonergic synapse	14	0.96021948	0.03644902	PTGS2, SLC6A4, KCNJ5, CYP4X1, JMJD7-PLA2G4B, PLCB3, ALOX15, PLA2G4F, GNB3, HTR1D, HTR2B, PLCB2, GNG7, TPH2	467	111	6879	1.85786214	0.99994468	0.62477478	38.521684
KEGG_PATHWAY	hsa00270:Cysteine and methionine metabolism	7	0.48010974	0.04065604	CBSL, DNMT3A, MAT1A, MTR, SDSL, AHCYL1, CBS	467	38	6879	2.71345655	0.99998257	0.63069577	41.947219
KEGG_PATHWAY	hsa01100:Metabolic pathways	98	6.72153635	0.04207223	ETNPPL, ALAD, PTGS2, GBGT1, OGDHL, GGT1, CAD, LSS, ITPKA, NMRK1, CKB, GLDC, CBSL, ST6GALNAC3, PLCB3, TRAK2, TKFC, MAT1A, PCYT2, SARDH, PLCB2, IMPDH2, HMGCL, PLD2, HYAL3, ACADS, SPTLC3, PIK3C2B, FBP1, NADSYN1, PIGQ, PNPLA3, ALDH3B1, LPIN3, ALOX15, MGAT3, FOLH1, NME3, DHRS4, PTGDS, H6PD, ABAT, PLA2G3, UROD, AOC3, PRODH, TPH2, ME3, ACADSB, SORD, ENPP3, ALDOB, DHRS4L2, EXTL1, ATP6V1B1, ATP6V0C, GALK1, JMJD7-PLA2G4B, ISYNA1, TYMP, IVD, HAAO, PEMT, FASN, PLCD4, PLCD1, TSTA3, GALNT12, GAL3ST1, ACSL5, DNMT3A, CHDH, ADSSL1, DGKQ, UPB1, HOGA1, SDSL, EPHX2, AK5, POLR3GL, ACACB, AK7, MAN1C1, MMAB, KHK, GGT6, POLD1, NDUFV1, MTR, PLA2G4F, AHCYL1, GAMT, RDH16, CYP8B1, LIPC, ATP6V0A2, NAT8L, CBS	467	1219	6879	1.18421566	0.9999882	0.61156281	43.060079
KEGG_PATHWAY	hsa04912:GnRH signaling pathway	12	0.82304527	0.04262103	EGFR, PLD2, JMJD7-PLA2G4B, PLCB3, PTK2B, JUN, ADCY6, PLA2G4F, HBEGF, MAPK11, PLCB2, MMP2	467	91	6879	1.942443	0.99998986	0.58708817	43.4860108
KEGG_PATHWAY	hsa05030:Cocaine addiction	8	0.54869684	0.04538527	GRM3, BDNF, GRM2, GRIN2C, JUN, GPSM1, CREB5, FOSB	467	49	6879	2.40492942	0.99999527	0.58349886	45.5869881
KEGG_PATHWAY	hsa04024:cAMP signaling pathway	21	1.44032922	0.04623366	PLD2, ADCY6, ATP1A3, OXTR, NPR1, CREB5, ATP1A2, VAV2, TNNI3, GLI1, BDNF, SSTR2, RAC2, GRIN2C, RAC3, JUN, PDE4B, GIPR, HHIP, ADCY10, HTR1D	467	198	6879	1.56229317	0.99999626	0.56531063	46.2171968
KEGG_PATHWAY	hsa00410:beta-Alanine metabolism	6	0.41152263	0.05507894	UPB1, ABAT, SMOX, ALDH3B1, AOC3, CARNS1	467	31	6879	2.85100504	0.99999968	0.60733139	52.397958
KEGG_PATHWAY	hsa04971:Gastric acid secretion	10	0.68587106	0.05686542	PLCB3, SSTR2, MYLK3, ADCY6, ATP1A3, MYLK2, ATP1A2, PLCB2, MYLK, KCNJ1	467	73	6879	2.01783462	0.99999981	0.59714938	53.5637232
KEGG_PATHWAY	hsa00051:Fructose and mannose metabolism	6	0.41152263	0.06180577	KHK, SORD, PFKFB4, ALDOB, FBP1, TSTA3	467	32	6879	2.76191113	0.99999995	0.60769206	56.6516811
KEGG_PATHWAY	hsa04068:FoxO signaling pathway	15	1.02880658	0.06874298	EGFR, IRS2, GABARAPL1, SGK1, MAPK11, IRS1, GRM1, BCL2L11, CDKN1A, PLK3, CCND2, GADD45G, BCL6, GADD45B, GADD45A	467	134	6879	1.64890217	0.99999999	0.62826826	60.6684521
KEGG_PATHWAY	hsa04310:Wnt signaling pathway	15	1.02880658	0.08319989	WNT10B, TCF7, DVL1, WNT2B, WNT4, PLCB3, DKK1, RAC2, RAC3, CCND2, JUN, NFATC4, BAMBI, FOSL1, PLCB2	467	138	6879	1.6011079	1	0.68229396	67.9585304
KEGG_PATHWAY	hsa04014:Ras signaling pathway	22	1.50891632	0.08547337	EGFR, PLD2, FGFR3, PDGFB, FLT4, EFNA3, KITLG, RASAL1, VEGFC, VEGFD, JMJD7-PLA2G4B, KSR2, RAC2, RAC3, ETS1, RASGRP2, PLA2G4F, GNB3, PLA2G3, RASA4, ANGPT2, GNG7	467	226	6879	1.43391257	1	0.67477585	68.9841083
