# Supplementary material for: Splicing factor SF3B1 promotes endometrial cancer progression via regulating KSR2 RNA maturation
Source: Cell Death Dis. 2020 Oct 10;11(10):842. doi: 10.1038/s41419-020-03055-y (PMC7548007; doi:10.1038/s41419-020-03055-y)
Supplement: Supplementary file 10 — Supplementary Table 5 [file 41419_2020_3055_MOESM10_ESM.pdf]

Supplementary Table 5: List of primers and probes

Gene name	Species	Application, Chemistry	Company	Sequence/Cat. No.
SF3B1	Human	qPCR, Taqman	ABI	MM00473105_m1
KSR2	Human	qPCR, Taqman	ABI	Hs00543277_m1
TRAF1	Human	qPCR, Taqman	ABI	Hs01090170_m1
GJD3	Human	qPCR, Taqman	ABI	Hs00987388_s1
DOCK11	Human	qPCR, Taqman	ABI	Hs00376176_m1
TBC1D16	Human	qPCR, Taqman	ABI	Hs00292666_m1
FOXQ1	Human	qPCR, Taqman	ABI	Hs00536425_s1
MYLK2	Human	qPCR, Taqman	ABI	Hs00263888_m1
18S	Human	qPCR, Taqman	ABI	4318839
KSR2 unspliced mRNA	Human	qPCR, Sybergreen	Sigma	Forward, 5'-GCTGAGGAAAGGGCAAGAA-3'<br>Reverse, 5'-TTCTTCTGCTGCCGTGTG-3'
KSR2 spliced mRNA	Human	qPCR, Sybergreen	Sigma	Forward, 5'-CCGTTCCGTGTGACATCAA-3'<br>Reverse, 5'-CGTGGCACTAGGAGGGA-3'
β-actin	Human	qPCR, Sybergreen	Sigma	Forward, 5'- AATGRGGCCGAGGACTTTGATTGC-3'<br>Reverse, 5'- AGGATGGCAAGGGACTTCCTGTAA-3'

*all primer sequences are written 5' to 3'
ABI-applied biosystems
IDT-integrated DNA technologies
