# Supplementary material for: Splicing factor SF3B1 promotes endometrial cancer progression via regulating KSR2 RNA maturation
Source: Cell Death Dis. 2020 Oct 10;11(10):842. doi: 10.1038/s41419-020-03055-y (PMC7548007; doi:10.1038/s41419-020-03055-y)
Supplement: Supplementary file 11 — Supplementary Table 6 [file 41419_2020_3055_MOESM11_ESM.pdf]

Table 6. List of antibodies

Antibody	Company, Catalogue number	Application
SF3B1	Abcam, ab172634	Immunoblotting, Immunofluorescence
Cyclin D1	Santa Cruz, sc-246	Immunoblotting
Cyclin E1	Santa Cruz, sc-377100	Immunoblotting,
Cyclin B1	Santa Cruz, sc-245	Immunoblotting
CDK2	Santa Cruz, sc-6248	Immunoblotting
CDK4	Santa Cruz, sc-56277	Immunoblotting
p21	Santa Cruz, sc-6246	Immunoblotting
KSR2	Santa Cruz, sc-100421	Immunoblotting
GFP	CST, #2956	Immunoblotting
E-Cadherin	Abcam,ab1416	Immunoblotting
Vimentin	Abcam,ab28028	Immunoblotting
GAPDH	CST, #2118	Immunoblotting
Anti-rabbit IgG, HRP-linked	CST, #7074	Immunoblotting
Anti-mouse IgG, HRP-linked	CST, #7076	Immunoblotting
Goat anti-Rabbit IgG (H+L)	Thermofisher Scientific, A32731	Immunofluorescence
Ki-67	ab16667	Immunofluorescence
